# Multicomponent Direct Assembly of *N*-Heterospirocycles Facilitated by Visible-Light-Driven Photocatalysis

**DOI:** 10.1021/acs.joc.2c01684

**Published:** 2022-09-14

**Authors:** Oliver M. Griffiths, Steven V. Ley

**Affiliations:** Yusuf Hamied Department of Chemistry, University of Cambridge, Cambridge CB2 1EW, U.K.

## Abstract

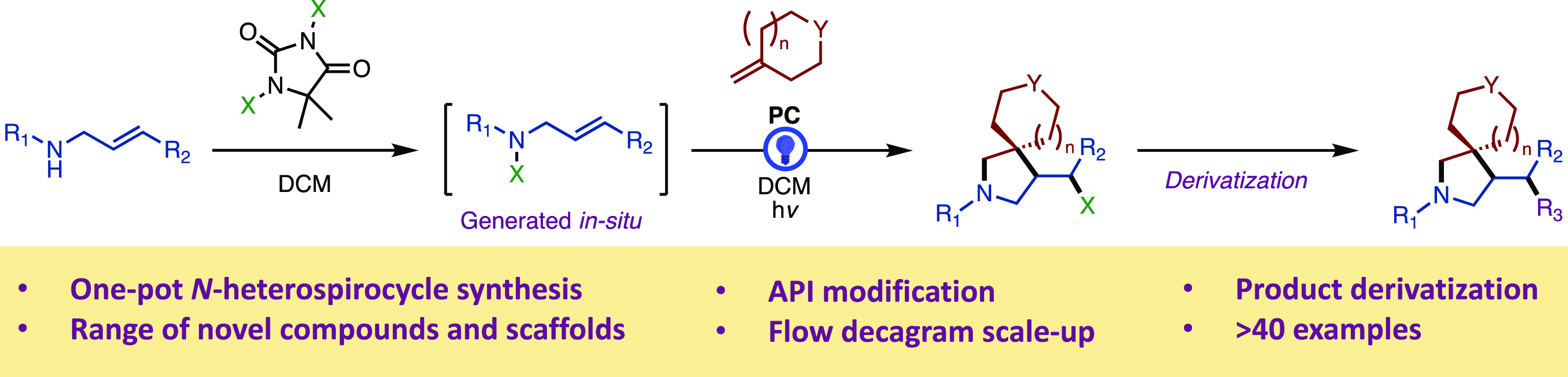

*N*-heterospirocycles are interesting structural units found in both natural products and medicinal compounds but have relatively few reliable methods for their synthesis. Here, we enlist the photocatalytic generation of *N*-centered radicals to construct β-spirocyclic pyrrolidines from *N*-allylsulfonamides and alkenes. A variety of β-spirocyclic pyrrolidines have been constructed, including drug derivatives, in moderate to very good yields. Further derivatization of the products has also been demonstrated as has a viable scale-up procedure, making use of flow chemistry techniques.

## Introduction

Developing new and versatile routes to desirable organic architectures constitutes an important aspect of the molecular assembly of pharmaceutically and agrochemically relevant molecules. One such novel class is spirocyclic derivatives, which feature in natural products and are increasingly both modeled as and observed in medicinal chemistry compounds.^1^ Heterospirocycles are particularly interesting for several reasons such as being isosteric alternatives to hydrophobic aromatic motifs,^2^ and they are also amenable to wide-ranging derivatization and heteroatom incorporation to adjust biological profiles.^3^

**Figure 1:**
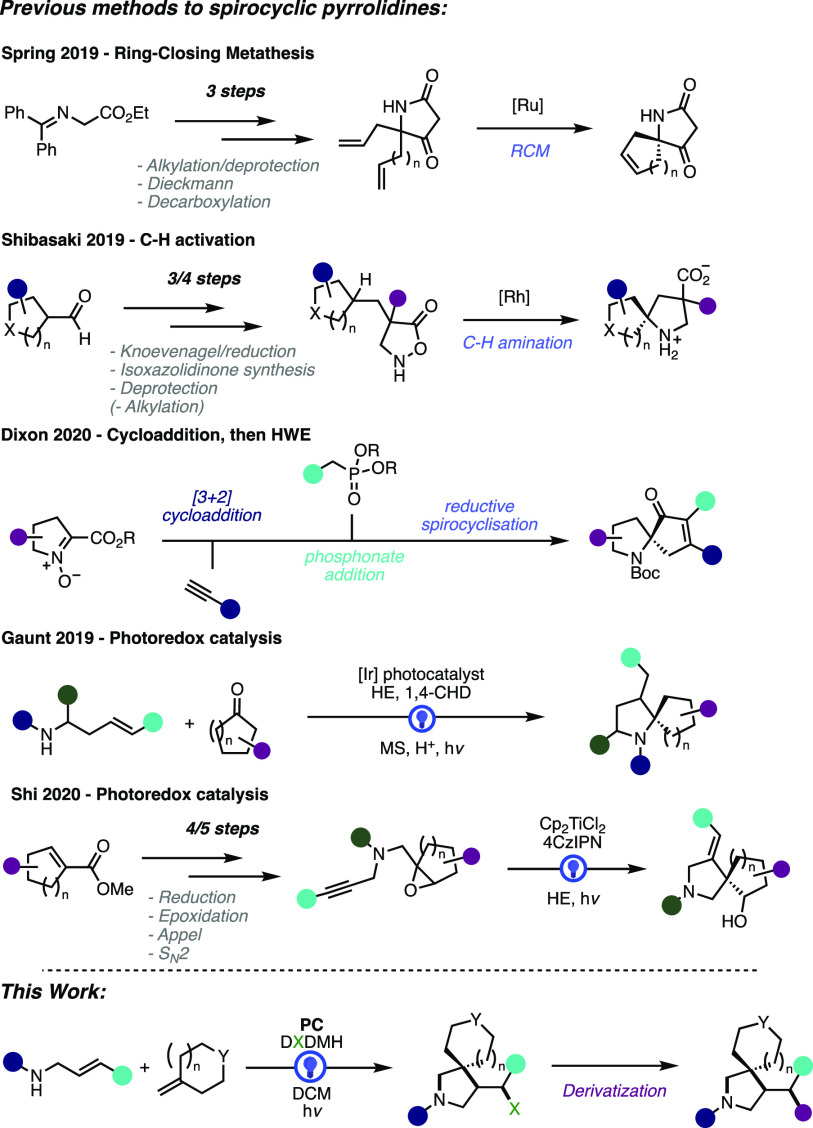
Strategies Developed to Access Spirocyclic Pyrrolidines.

Of the methods developed to prepare azaheterospirocycles in recent years, most require multistep procedures;^3a,3c,3d,4,5^ however, the latest applications of photocatalysis have made this possible in few synthetic steps.^6^ Indeed, the development of highly efficient light sources, especially LEDs, has triggered a renaissance of interest in photochemical reactions.^7^ As such, photocatalysis has arisen as a valuable tool that enables the construction of complex molecular structures in a modular fashion under mild conditions via the intermediacy of highly reactive species.^8^ Of the methods developed to prepare spirocyclic pyrrolidines, most have enabled the preparation of α-spirocyclic pyrrolidines, overcoming some of the difficulties associated with preparing α-tertiary amines (Figure 1).^3c,4b−4d,4g,4i,6a^ β-spirocyclic pyrrolidine scaffolds, despite their increased uptake as medicinal targets, have by comparison received less attention and thus often rely on multistep syntheses.^3a−3c,4f,4h^

The generation of nitrogen-centered radicals (NCRs) has been heavily influenced by the emergence of photocatalysis, facilitating several modes of activation of free N–H and N–X bonds.^9^ The utility of NCRs is enhanced by the ability to attach different groups to the nitrogen atom, conveniently modulating their reactivity.^9b,10^ As such, many NCR strategies have been shown to successfully incorporate nitrogen into aliphatic substrates and also to construct nitrogen-containing heterocycles, scaffolds, and readily decorated fragments.^9^ Of these modes, homolytic cleavage of N–X bonds has proved useful and can be executed, among other methods, by direct photoexcitation with UV light or by photosensitization.^9^ Our group has taken interest in this area and has previously disclosed a one-pot, two-step pyrrolidine ring-forming reaction using electron-rich or activated alkenes, a chlorinating agent, *N*-allylsulfonamides, and a photosensitizer (either organic or inorganic).^11^ Here, we report development on this strategy that makes use of the matched electrophilicity of arylsulfonamides and exocyclic olefins that enables access to a variety of β-spirocyclic pyrrolidines in moderate to excellent yields. Further derivatization and scale-up by the use of flow chemistry have also been explored.

## Results and Discussion

Adopting *N*-allyltoluenesulfonamide (**1a**), 1,3-dichloro-5,5′-dimethylhydantoin, and methylenecyclohexane (**2a**) as model substrates and (Ir[dF(CF_3_)ppy]_2_(dtbpy))PF_6_ (PC1) as the photocatalyst in the CH_2_Cl_2_ solvent, we set about finding viable reaction conditions (Table 1). We were pleased to find that spirocycle **3a** was formed in 80% yield by NMR and was isolated in 76% yield (entry 1). *N*-Boc, *N*-methyl, and *N*-acetamide-protected allylamine were all unsuccessful when investigated as alternatives to the sulfonamide-protected allylamine. Likewise, *N*-chlorosuccinimide and trichloroisocyanuric acid alternative chlorinating reagents gave inferior yields of **1a**. As expected, removal of the photocatalyst from the reaction mixture decreased the yield of the product substantially with a large quantity of the N–Cl intermediate remaining unconverted, evidencing its function as a photocatalyst (entry 2). Removal of the light source almost completely prevented any conversion of the intermediate (entry 3). Interestingly, performing the reaction under air only mildly decreased the yield of **3a** (entry 4). Switching the solvent to acetonitrile failed to deliver any identifiable spirocyclic product **3a**, instead producing **1a** as the sole product presumably by hydrogen atom transfer (HAT) of the nitrogen-centered radical from the solvent (entry 5). Using 1,2-dichloroethane (DCE) as the reaction solvent did however give **3a** in 62% yield (entry 6). Both increasing and decreasing the reaction concentration for the photochemical step made little impact on the conversion to the product (entries 7 and 8). Reducing the stoichiometry of **2a** did decrease the yield (entry 9) and increased the proportion of **1a** recovered from the reaction mixture, likely by increasing the rate of HAT from the solvent relative to olefin addition. Increasing the quantity of **2a** only had a marginal improvement on the yield (entry 10) and so was not adopted for the remainder of the experiments so as not to unnecessarily expend other potentially valuable olefins. Adopting only a 5 min prestir for *N*-chlorination in step 1 still gave spirocycle **3a** in 76% isolated yield (entry 11). Adopting an organic photocatalyst (4CzIPN) instead of (Ir[dF(CF_3_)ppy]_2_(dtbpy))PF_6_ was also able to deliver **3a** in 72% yield, demonstrating the option of switching out expensive inorganic photocatalysts for easily prepared organic alternatives (entry 12).

**Table 1:**
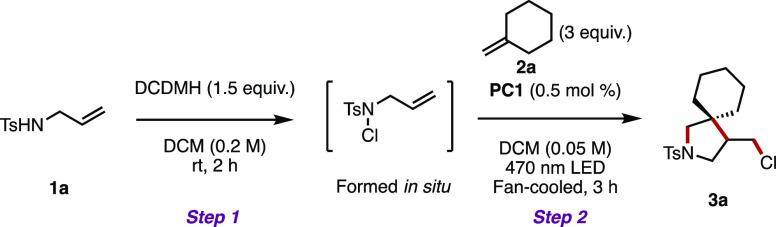
Reaction Optimization

entry	deviation from standard conditions	yield (%)^a^
1	none	80 (76)^b^
2	no photocatalyst	14^c^
3	no light	<5^c^
4	under air	72
5	MeCN solvent	0^d^
6	DCE solvent	62
7	0.1 M (step 2)	80
8	0.025 M (step 2)	78
9	2 equiv **2a**	64
10	5 equiv **2a**	82
11	5 min (step 1)	76^b^
12	4CzIPN used as PC	72^b^

a Reaction conditions: 0.2 mmol, 1.0 equiv of *N*-allyltoluenesulfonamide, 1.5 equiv of 1,3-dichloro-5,5′-dimethylhydantoin, 3.0 equiv of methylenecyclohexane, 0.5 mol % (Ir[dF(CF_3_)ppy]_2_(dtbpy))PF_6_ (**PC1**), 4 mL total (step 2) of dichloromethane (DCM). ^1^H NMR conversion to the product.

b Isolated yield.

c Unreacted N–Cl intermediate observed as the major component by ^1^H NMR.

d *N*-allyltoluenesulfonamide alone observed by ^1^H NMR.

With the new reaction conditions established, we set about evaluating the suitability of a range of *N*-allylsulfonamides (Table 2). The common amine-protecting group (*N*-tosyl)allylamine underwent spirocyclization in 76% isolated yield to **3a**. *N*-(4-Bromobenzenesulfonyl)allylamine (**1b**) was less well-suited to the conditions, providing **3b** in modest yield. Electron-withdrawn aromatic sulfonamides were well tolerated under the conditions, producing **3c** and **3d** in very good and good yields, respectively. Heterocyclic sulfonamide **3e** was tolerated, albeit modestly, as was 4-(trifluoromethoxy)benzenesulfonamide **3f**. Sulfone-containing spirocycle **3g** was produced in very good yield. *N*-allylsulfonamides **1h** and **1i** produced spirocycles **3h** and **3i**, respectively, in good and moderate yields, demonstrating the opportunity to have variants of the methyl substituent on the pyrrolidine ring. Alternative halogenating agents were able to yield the desired spirocyclic scaffolds but with alternative halogen atom substituents on the pyrrolidine methyl substituent, providing alkyl bromide **3j** and alkyl iodide **3k** in very good yields. *N*-methanesulfonylallylamine (**1l**) gave no conversion to the product, instead giving full return of the starting material.

**Table 2:**
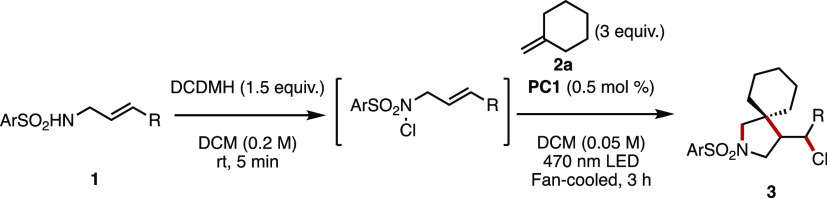

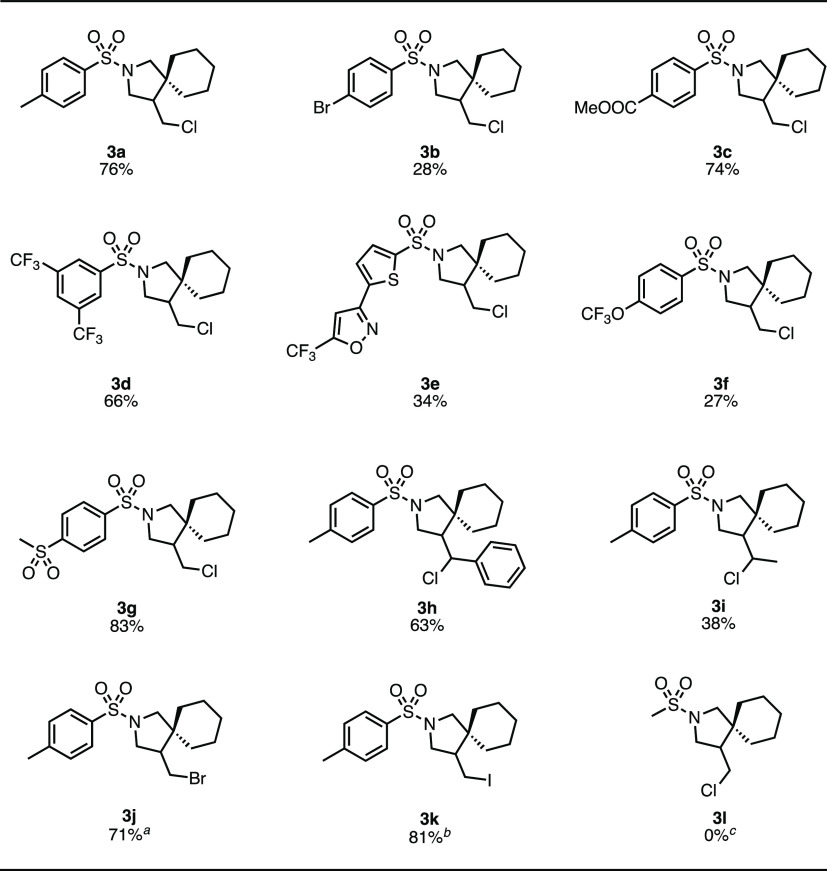
*N*-Allylsulfonamide Scope^a^

a Reaction conditions: 0.2 mmol, 1.0 equiv of *N*-allyltoluenesulfonamide, 1.5 equiv of 1,3-dichloro-5,5′-dimethylhydantoin, 3.0 equiv of methylenecyclohexane, 0.5 mol % (Ir[dF(CF_3_)ppy]_2_(dtbpy))PF_6_, 4 mL total (step 2) of DCM. ^a^1.5 equiv of 1,3-dibromo-5,5′-dimethylhydantoin was used instead of 1,3-dichloro-5,5′-dimethylhydantoin.

b 1.5 equiv of 1,3-diiodo-5,5′-dimethylhydantoin was used instead of 1,3-dichloro-5,5′-dimethylhydantoin.

c Starting material **1l** was the sole observed product in the crude ^1^H NMR spectrum.

We subsequently turned our attention to the olefin component (Table 3). Aliphatic and aromatic substituted olefins were tolerated well under the reaction conditions, producing separable mixtures of *N*-heterospirocycle diastereomers (**3m** and **3n**, respectively). The presence of a large number of benzylic protons in the olefin component (**2o**) however led to a diminished yield of **3o** and a large recovery of the HAT product **1a**. Spirocyclization of 2-methyleneadamantane (**2p**) was successful, showing tolerance for α-hindered olefins to produce **3p** in a decent yield. Other medicinally and synthetically useful moieties such as *gem*-difluoro- and acetal-protected ketones were well tolerated, producing **3q** and **3r** in good yields. Aliphatic rings of varying sizes were also tolerated from small rings (**3s**), through medium-sized rings (**3a** and **3t**), to macrocyclic rings (**3u** and **3v**), all of which were obtained in good to very good yields. Most noteworthy of these are the macrocyclic rings **3u** and **3v** (spirocyclic 12- and 15-membered aliphatic rings respectively), which would pose synthetic challenges by other means. Spirocycles constituting two heterocycles were then our main point of focus for novel structures and it indeed proved fruitful. Oxo-heterocycles oxetane-containing *bis*-spirocycle **3w** and tetrahydropyran-containing spirocycle **3x** were obtained in 55 and 46% yields, respectively, from their corresponding alkenes. The unusual *bis*-spirocyclic oxazoline **3y** was accessed in an acceptable 39% yield and sulfone-containing heterocycle **3z** was also well-suited. Observing recent interest in *bis*-azaheterocycles for applications such as bioavailable strained-type linkers,^3^ we next turned our focus to preparing a selection of *bis*-azaheterospirocycles with orthogonal protecting groups and with both chloromethyl and bromomethyl moieties. Azetidine **3aa** was prepared in reasonable yield as was *bis*-pyrrolidine spirocycle **3ab**, though the latter was an inseparable 1:1 mixture of diastereomers. Piperidine ring-containing spirocycles could be successfully prepared in good to very good yields with or without orthogonal protecting groups and with either chloromethyl or bromomethyl substituents (**3ac**–**3af**) and also demonstrated the potential to switch out the expensive [Ir] photocatalyst for an easily prepared organic photocatalyst, with no diminishment in yield (**3af**). Finally, *bis*-azaheterospirocycles **3ag** and **3ah** were prepared in moderate and good yields, respectively.

**Table 3:**
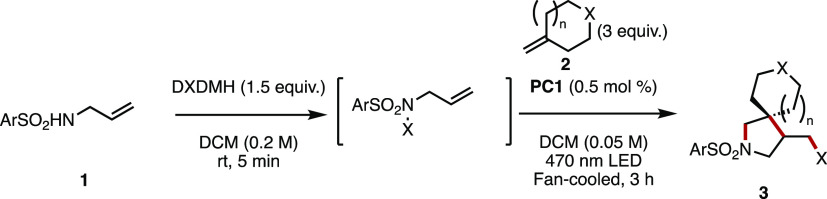

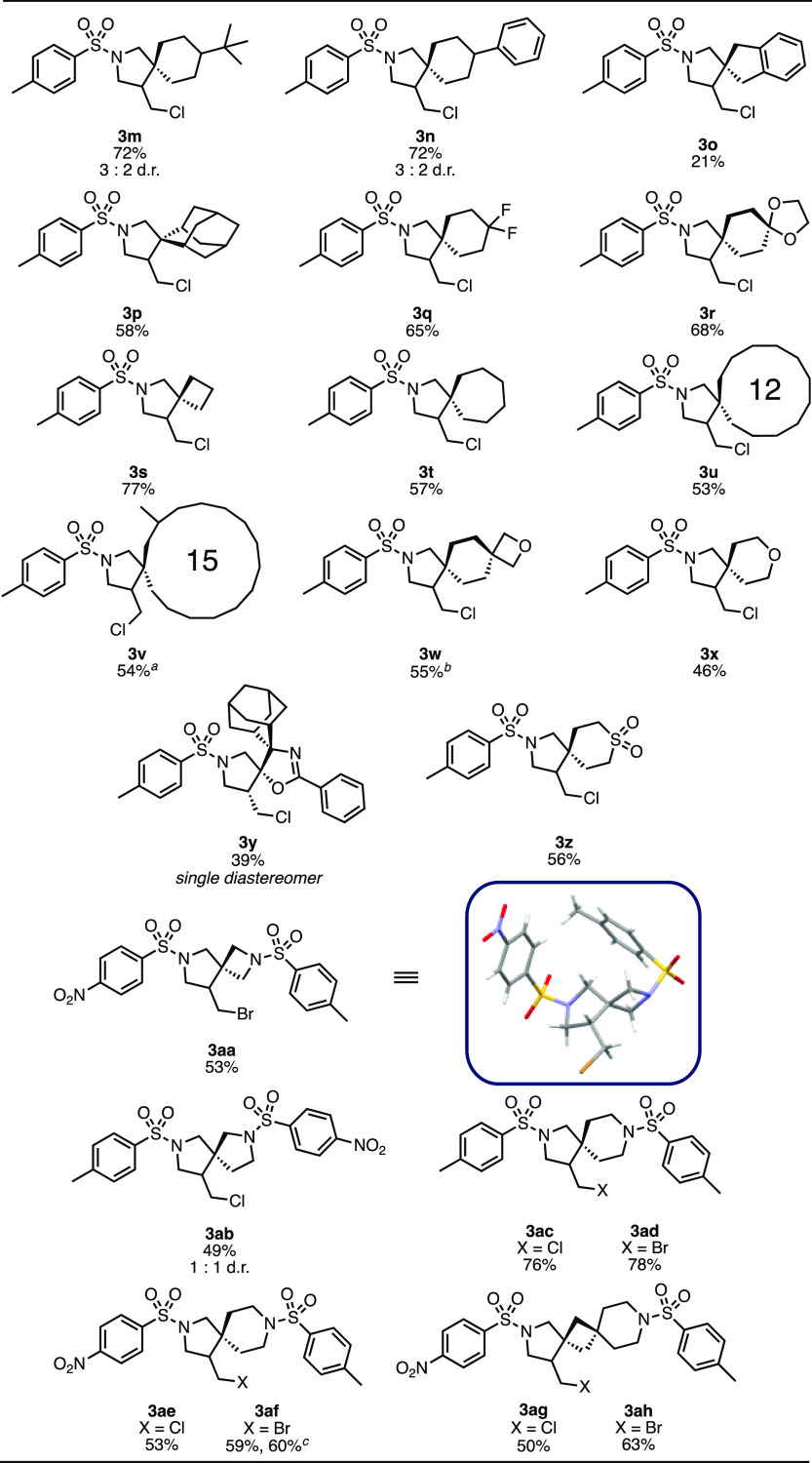
Exocyclic Olefin Scope^a^

a Reaction conditions: 0.2 mmol, 1.0 equiv of *N*-allyltoluenesulfonamide, 1.5 equiv of 1,3-dihalo-5,5′-dimethylhydantoin, 3.0 equiv of olefin, 0.5 mol % (Ir[dF(CF_3_)ppy]_2_(dtbpy))PF_6_, 4 mL total (step 2) of DCM. ^a^Diastereomeric ratios not able to be determined due to inseparability and substantial overlapping of peaks in the ^1^H NMR spectra of the isolated diastereomers.

b 2 equiv of olefin component used.

c 0.5 mol % 4CzIPN used as photocatalyst instead.

The inevitable formation of the pendant halomethyl group on the pyrrolidine ring opens up opportunities for further diversification of the products. We have previously shown how a pyrrolidine chloromethyl substituent can be altered by nucleophilic substitution and elimination; however, to open up other modes of activation such as cross-coupling, an sp^2^-Cl moiety was required.^11^ The realization that other halogens can be installed instead of just chlorine (as found previously) serves to facilitate other derivatization options for the products. As such, we set about to demonstrate a few of these options (Table 4). First, *in situ* hydrodehalogenation of the alkyl halide using (tristrimethylsilyl)silane (TTMSS) produced derivatives **4a–c** in good yields (Table 4a). The hydrodehalogenation reaction to **4a** from alkyl bromide **3j** was notably slower than from alkyl iodide **3k**, requiring 24 h of 470 nm LED irradiation to achieve full conversion (though producing **4a** in superior yield). This poses an attractive one-pot (double-photochemical), three-step telescoped method to the hydrodehalogenated β-spirocyclic pyrrolidine products. Alkyl bromide **3ad** was further derivatized by an unoptimized Kornblum oxidation to aldehyde **5** in 40% yield and was also amenable to a previously reported photoredox-catalyzed cross-electrophile coupling reaction^12^ with 3-bromopyridine, forming *tris-*azaheterocycle **6** in 32% yield (Table 4b).

**Table 4:**
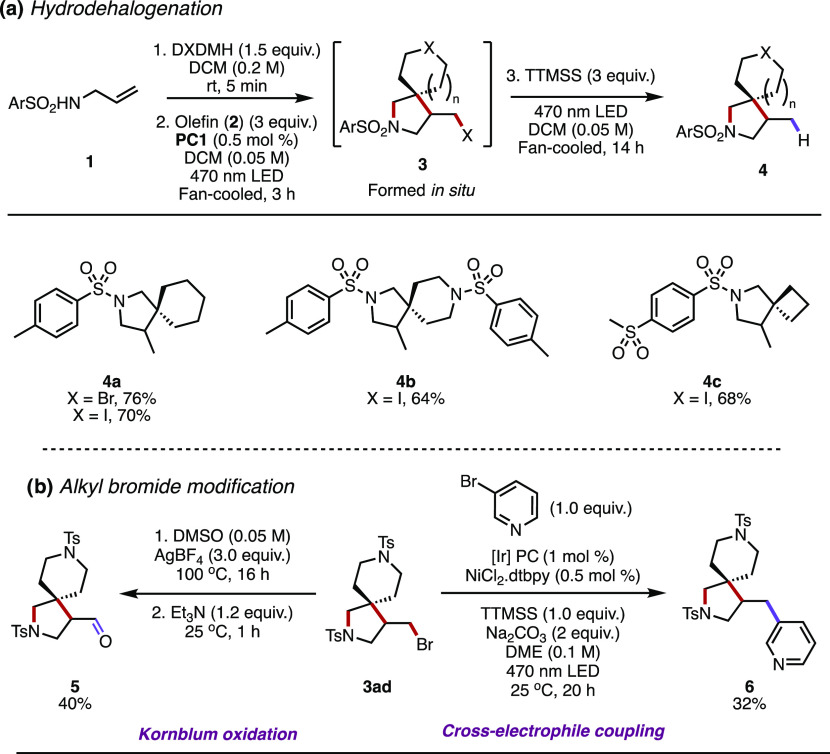
Halomethyl Derivatizations

Recognizing the ability to easily construct interesting *N*-heterospirocycles using this method, we became interested in its possible application toward drug derivatization. Sulfonamides are frequently found among APIs, agrochemicals, and natural products and are, therefore, highly desirable targets for selective modification.^13^ The opportunity to install spirocyclized pyrrolidine rings on sulfonamide-containing drugs using this method is particularly attractive due to the tethered halogen substituent. This can be removed, cross-coupled, or transformed to a plethora of other desirable functional groups, thus opening up a wider range of chemical space to be explored for biological activity. We were therefore pleased to find that a selection of drug molecules could be derivatized in moderate to excellent yields (Table 5). Celecoxib derivatives **3ai** and **3aj** were prepared in moderate and very good yields, respectively, showing that a variety of spirocyclic compounds from sulfonamide-containing drugs are possible. Valdecoxib, methazolamide, and ethoxzolamide derivatives **3ak**, **3aj**, and **3am** were also successfully prepared. Using the telescoped one-pot hydrodehalogenation procedure (Table 4a), probenecid analogue **4d** was obtained in excellent yield. Examples **3ak**, **3aj, 3am**, and **4d** are all compliant with Lipinski’s rule of 5, relating to the favorable physicochemical properties of drug molecules.^14^

**Table 5:**
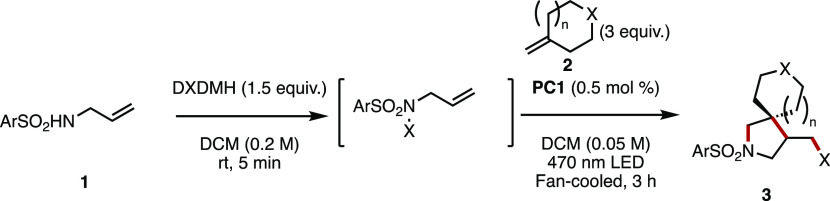

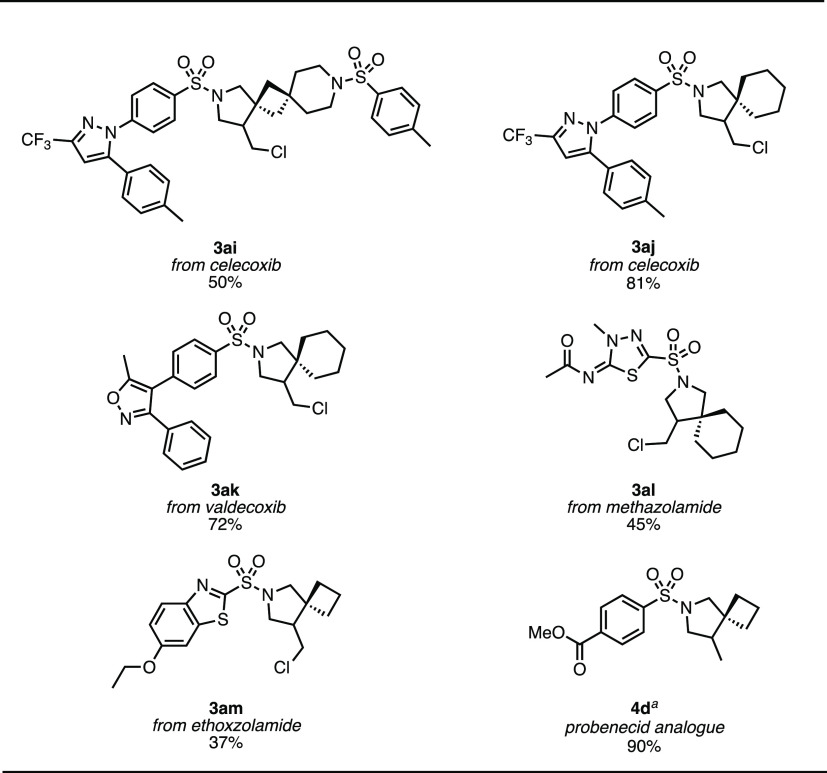
Drug Derivatives^a^

a Reaction conditions: 0.2 mmol, 1.0 equiv of *N*-allyltoluenesulfonamide, 1.5 equiv of 1,3-dihalo-5,5′-dimethylhydantoin, 3.0 equiv of olefin, 0.5 mol % Ir[dF(CF_3_)ppy]_2_(dtbpy)PF_6_, 4 mL total (step 2) of DCM. ^a^1,3-diiodo-5,5′-dimethylhydantoin used and the resulting alkyl iodide subjected to hydrodehalogenation conditions with 4.0 equiv TTMSS *in situ* as in Table 4a.

With the application of this method to make a variety of *N*-heterospirocyclic structures and derivatives illustrated, we turned our attention to addressing the scalability of the reaction. Despite the attraction of photochemistry for facilitating the construction of novel compounds, photochemical reactions are notorious for their poor efficiency, reproducibility, and scalability.^15^ Many groups, including ours, have found that adopting continuous flow platforms can have several advantages over their batch counterparts by mitigating some of the issues faced with photochemistry such as superior heat distribution, mixing, and photon flux as a result of the small cross section of tubing used in flow setups.^7c,16^

Perhaps the most attractive aspect of continuous processing, however, particularly in industrial settings, is safety and hazard containment.^17^ Continuous flow setups have been employed to avoid the build-up of large quantities of hazardous intermediates by generating them *in situ*, performing the reaction and then quenching any left unreacted, all within a short period of time and in a relatively small volume of tubing.^18^ This reduces chemical hazards associated with mechanical failure of the reactor and/or leakage, while also providing greater control over thermal runaway pathways due to the high surface-area/volume ratio of flow tubing compared to batch reactors.^18,19^*N*-Haloamines, despite their utility in synthesis,^20^ are often overlooked for large-scale applications due to concerns around toxicity and instability. Indeed, continuous flow platforms have been applied to handling *N*-chloramines in the synthesis successfully to mitigate some of their associated challenges.^21^ As such, we saw the potential to tackle both the photochemical scale-up and the *N*-haloamine instability using a one-flow reaction to execute a decagram scale-up of one particular spirocycle as a demonstration of the process.

**Scheme 1:**
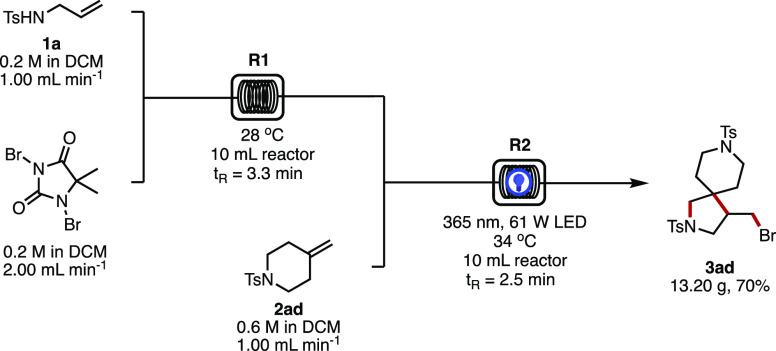
Multigram Continuous Flow Construction of β-Spirocyclic Pyrrolidine **3ad**

Previously, we have made efforts to reduce the overall cost of multigram-scale photocatalytic reactions by employing a photocatalyst recycling method.^16g^ To further such efforts, we reasoned here that the use of a high-power UV lamp for the scaled procedure would make the presence of a photocatalyst unnecessary by facilitating direct photocleavage of the N–X bond. Transferring the batch procedure into flow with the use of a 61 W 365 nm LED, we found the reaction to be equally successful both with and without a photocatalyst. By employing 1.0 equiv of **1a**, 2.0 equiv of 1,3-dibromo-5,5′-dimethylhydantoin, 3.0 equiv of olefin **2ad**, and a residence time of just 2.5 min in the photoreactor, we were able to prepare *bis*-azaheterospirocycle **3ad** in 71% isolated yield on a 0.2 mmol scale. We then set about performing the scaled-up one-flow two-step reaction so as to generate the N–Br intermediate *in situ* and consume it directly from the output feed (Scheme 1). With a residence time of 3.33 min at room temperature, the output of reactor R1 was then mixed at a Y-piece with another inlet delivering a solution of olefin **2ad**. The resulting solution was then flowed into a UV-150 photoreactor at 34 °C with a residence time of 2.50 min and the output collected, dried, and purified by chromatography to give 13.20 g of **3ad** (70% yield) in 3 h. Using this reactor setup, a throughput of 105.6 g day^–1^ can be achieved for this particular spirocycle.

In summary, we have described a new method to prepare a range of β-spirocyclic pyrrolidines from *N*-allylsulfonamides, halogenating agents, and exocyclic olefins. Furthermore, we have shown how these products can be further derivatized *via* the pendant halomethyl group present in the product and also the formation of spirocyclic drug derivatives using this method. Finally, we have demonstrated that this transformation can be executed efficiently in a continuous flow platform on a decagram scale using high-power LEDs, while also mitigating potential hazards surrounding the handling of *N*-halo intermediates.

## Experimental Section

### General Methods

All procedures below were conducted under inert nitrogen atmosphere unless stated otherwise. Reagents were supplied by Sigma-Aldrich, Alfa Aesar, Acros, TCI, and Fluorochem and were used as received. Dichloromethane (extra) dry was purchased from Acros Organics and used for all spirocyclization reactions. All substrates were synthesized using the batch photochemical setup (as shown in the Supporting Information (SI)), consisting of a coiled LED strip (8–9 W total power output) on the inside of the tubing (4 cm in height, 8 cm in diameter). Each reaction used commercially available 6 mL microwave vials, cleaned and oven-dried before use. The vials were placed individually in the center of the photoreactor tube and were fan-cooled from above (under these conditions, the reaction temperature did not exceed 35 °C). Flow reactions and scale-up experiment were performed using a Vapourtec E-series comprising three peristaltic pumps and a photoreactor, with an 8 bar BPR at the output. The light source used was a commercially available 61 W radiant power 365 nm (peak intensity) LED from Vapourtec with an irradiation band ranging 350–400 nm. The reactor coil constituted 10 mL total volume of FEP tubing (ID = 1.0 mm). Work-up solvents were obtained from commercial sources and distilled prior to use. Petroleum ether (Pet ether) refers to the fractions of petrol collected between 40 and 60 °C b.p. Flash column chromatography was conducted either using a Biotage SPX system with single-use disposable silica columns of appropriate size (SiliaSep Flash Cartridges 12 or 25 g 40–60 μm ISO04/012) or manually with silica gel 60 (230–400 mesh particle size, 40–63 μm particle size). Thin-layer chromatography analysis was carried out using silica gel 60 F254 precoated glass-backed plates and visualized under UV light (254 nm) or with permanganate or vanillin stains. ^1^H NMR, ^13^C{^1^H} NMR, and ^19^F NMR were obtained using a 500 MHz Bruker Avance III (Smart Probe 500 MHz) Spectrometer or a Bruker AV400 (Avance 400 MHz) Spectrometer. Chemical shifts (d) are referenced to residual CDCl_3_ in parts per million (ppm). Coupling constants *J* are quoted in hertz (Hz). Proton and carbon multiplicity is recorded as singlet (s), doublet (d), double of doublets (dd), triplet (t), quartet (q), pentet/quintet (p), heptet (hept.) multiplet (m), and broad (br), or combinations thereof. All compounds examined were dried *in vacuo* to remove the residual solvents. ^1^H NMR signals are reported to two decimal places and ^13^C{^1^H} signals to one decimal place. Assignments are made according to the labeling of structures in the experimental section and derived from two-dimensional (2D) spectra, all of which are accessible by request from the authors. The operating frequency used for all nuclei in the included 2D spectra (see the SI) are those reported in the corresponding ^1^H and ^13^C NMR spectra captions immediately above the 2D spectra. High-resolution mass spectra (HRMS) were obtained on a Waters Vion IMS QTOF spectrometer. Infrared spectra were recorded neat on a PerkinElmer Spectrum One Fourier transform infrared (FTIR) spectrometer with a universal attenuated total reflection (ATR) sampling accessory, and selected peaks are reported.

### General Procedure A: Preparation of *N*-Allylsulfonamides **1a, 1b, 1c, 1d, 1e, 1f, 1g, 1l, 1aa**, and **1ak** (Schotten–Baumann-Like Conditions)

*N*-Allylamine (1.1 equiv) and dry Et_3_N (1.1 equiv) were dissolved in DCM (0.25 M) under N_2_. The mixture was then stirred and cooled to 0 °C. The corresponding sulfonyl chloride (1.0 equiv) was added dropwise if liquid, or in small portions if solid. The resulting reaction mixture was then stirred at 0 °C for 1 h and then allowed to warm to room temperature and continue to react for a further 15 h. The crude solution was then added to sat. NaHCO_3_, the layers were separated, and the aqueous layer was extracted with DCM (×3). The combined organic phases were then washed with brine, dried (MgSO_4_), filtered, and then concentrated *in vacuo* to yield the desired *N*-allylsulfonamide. The literature data for compounds **1a**,^11^**1b**,^22^**1d**,^22^**1e**,^11^**1f**,^23^**1h**,^24^**1i**,^25^**1l**,^22^ and **1aa**(22) matched the data obtained using this procedure.
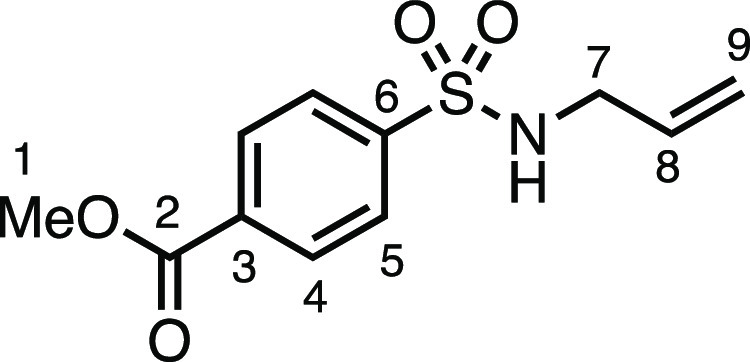


#### Methyl 4-(*N*-(But-3-en-1-yl)sulfamoyl)benzoate (**1c**)

*N*-Allylamine (165 μL, 2.2 mmol), dry Et_3_N (307 μL, 2.2 mmol), DCM (8 mL), and methyl 4-(chlorosulfonyl)benzoate (469 mg, 2.0 mmol) were subjected to General Procedure A to give compound **1c** as a white solid (444 mg, 87%). ^1^H NMR (400 MHz, CDCl_3_) δ 8.17 (app d, *J* = 8.6 Hz, 2H, H_4_), 7.94 (app d, *J* = 8.6 Hz, 2H, H_5_), 5.76–5.60 (m, 1H, H_8_), 5.15 (dd, 1H, *J* = 17.1, 1.2 Hz, H_9_), 5.09 (dd, 1H, *J* = 10.2, 1.2 Hz, H_9′_), 4.80 (t, *J* = 6.1 Hz, 1H, N–H), 3.96 (s, 3H, H_1_), 3.63 (tt, *J* = 6.1, 1.4 Hz, 2H, H_7_). ^13^C{^1^H} NMR (101 MHz, CDCl_3_) δ 165.8 (C_2_), 144.2 (C_3_), 134.0 (C_6_), 132.8 (C_8_), 130.5 (C_4_), 127.2 (C_5_), 118.2 (C_9_), 52.8 (C_1_), 45.9 (C_7_). Mp: 80.0–81.9 °C. IR: ν_max_ 3231, 2920, 1701, 1434, 1333, 1287, 1156 cm^–1^. HRMS: *m*/*z* calculated for C_11_H_14_NO_4_S^+^, 256.0638 [M + H]^+^. Found *m*/*z* 256.0632, Δ = −2.3 ppm.
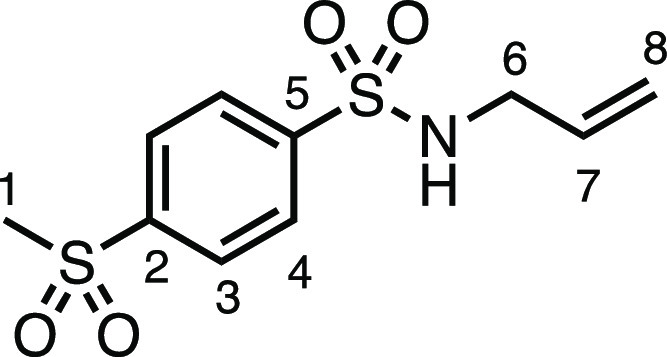


#### *N*-Allyl-4-(methylsulfonyl)benzenesulfonamide (**1g**)

*N*-Allylamine (330 μL, 4.4 mmol), dry Et_3_N (614 μL, 4.4 mmol), DCM (16 mL), and 4-(methylsulfonyl)benzenesulfonyl chloride (1.02 g, 4.0 mmol) were subjected to General Procedure A to give compound **1c** as a white solid (809 mg, 74%). ^1^H NMR (400 MHz, CDCl_3_) δ 8.09 (m, *J* = 8.5 Hz, 4H, H_3_ + H_4_), 5.72 (ddt, *J* = 16.5, 11.2, 5.9 Hz, 1H, H_7_), 5.19 (d, *J* = 17.2 Hz, 1H, H_8_), 5.14 (d, *J* = 10.2 Hz, 1H, H_8′_), 4.64 (t, *J* = 5.9 Hz, 1H, N–H), 3.68 (t, *J* = 6.0 Hz, 2H, H_6_), 3.10 (s, 3H, H_1_).^13^C{^1^H} NMR (101 MHz, CDCl_3_) δ 145.7 (C_5_), 144.5 (C_2_), 132.6 (C_7_), 128.6 (C_3_ or C_4_), 128.3 (C_3_ or C_4_), 118.5 (C_8_), 46.0 (C_6_), 44.5 (C_1_). Mp: 147.0–148.6 °C. IR: ν_max_ 3268, 2922, 1435, 1386, 1326, 1308, 1282, 1147 cm^–1^. HRMS: *m*/*z* calculated for C_10_H_14_NO_4_S_2_^+^, 276.0359 [M + H]^+^. Found *m*/*z* 276.0354, Δ = −1.7 ppm. Rf (30% EtOAc in Pet ether) = 0.21.
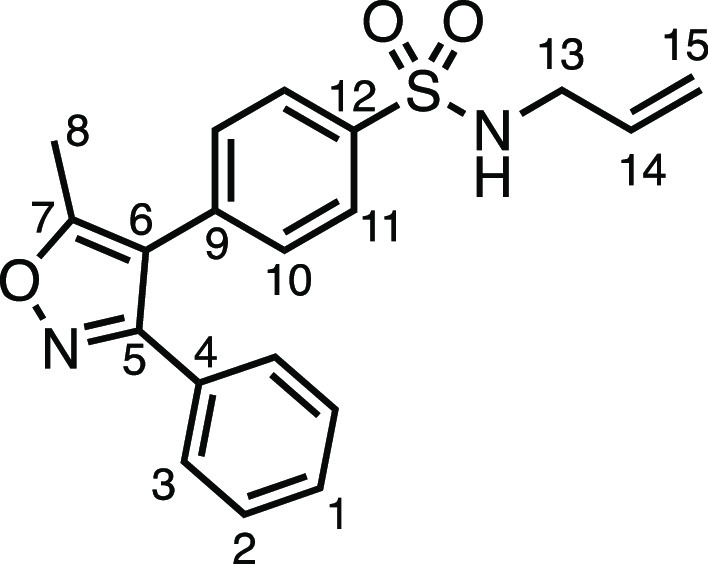


#### *N*-Allyl-4-(5-methyl-3-phenylisoxazol-4-yl)benzenesulfonamide (**1ak**)

*N*-Allylamine (124 μL, 1.65 mmol), dry Et_3_N (229 μL, 1.65 mmol), DCM (6 mL), and 4-(methylsulfonyl)benzenesulfonyl chloride (500 mg, 1.5 mmol) were subjected to General Procedure A to give compound **1c** as a white solid (531 mg, 99%). ^1^H NMR (400 MHz, CDCl_3_) δ 7.86 (app d, *J* = 7.7 Hz, 2H, H_11_), 7.36 (m, 7H, H_1_ + H_2_ + H_3_ + H_10_), 5.73 (ddt, *J* = 16.4, 11.0, 5.7 Hz, 1H, H_14_), 5.17 (d, *J* = 17.2 Hz, 1H, H_15_), 5.11 (d, *J* = 10.2 Hz, 1H, H_15′_), 4.69 (bs, 1H, N–H), 3.67 (bs, 2H, H_13_), 2.49 (s, 3H, H_8_). ^13^C{^1^H} NMR (101 MHz, CDCl_3_) δ 167.4 (C_7_), 161.2 (C_5_), 139.4 (C_9_), 135.4 (C_12_), 133.0 (C_14_), 130.5 (C_10_), 129.9 (C_1_), 128.8 (C_2_ or C_3_), 128.6 (C_2_ or C_3_ + C_4_), 127.6 (C_11_), 117.9 (C_15_), 114.6 (C_6_), 45.9 (C_13_), 11.9 (C_8_). Mp: 91.0–93.3 °C. IR: ν_max_ 3293, 1623, 1412, 1394, 1329, 1164 cm^–1^. HRMS: *m*/*z* calculated for C_19_H_19_N_2_O_3_S^+^, 355.1111 [M + H]^+^. Found *m*/*z* 355.1109, Δ = −0.5 ppm. Rf (30% EtOAc in Pet ether) = 0.21.

### General Procedure B: Preparation of Sulfonamides **1h, 1i, 1ai**, and **1al** (S_N_2 Conditions)

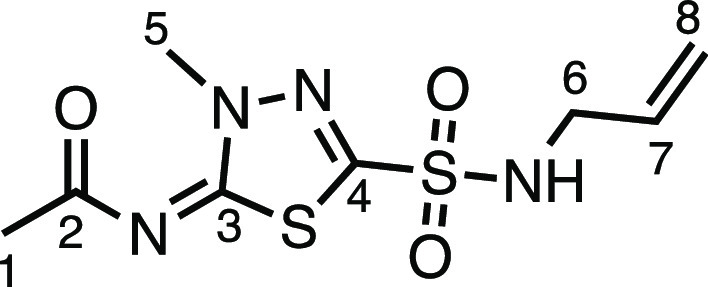



Alkyl bromide (1.0 equiv), K_2_CO_3_ (2.0 equiv), KI (0.10 equiv), and the corresponding sulfonamide (2.0 equiv) were combined in a round-bottom flask and the mixture subjected to a nitrogen atmosphere. MeCN (0.33 M) was then added and the resultant mixture was heated to 60 °C for 12 h and then cooled to room temperature. The mixture was then filtered and the filtrate concentrated *in vacuo*. The residue was then subjected to flash chromatography to yield the desired sulfonamide. The literature data for compounds **1h**,^26^**1i**,^27^ and **1ai**(22) matched the data obtained using this procedure.

#### (*E*)-*N*-(5-(*N*-Allylsulfamoyl)-3-methyl-1,3,4-thiadiazol-2(3*H*)-ylidene)acetamide (**1al**)

Allyl bromide (347 μL, 4.0 mmol), K_2_CO_3_ (1.107 g, 8.0 mmol), KI (67 mg, 0.4 mmol), MeCN (12 mL), and methazolamide (1.89 g, 8.0 mmol) were subjected to General Procedure B (chromatography eluent: 20–40% EtOAc in Pet ether) to give compound **1al** as a white solid (668 mg, 60%). ^1^H NMR (400 MHz, CDCl_3_) δ 5.93–5.73 (m, 2H, H_7_ + N–H), 5.28 (d, *J* = 17.1 Hz, 1H, H_8_), 5.20 (d, *J* = 10.2 Hz, 1H, H_8′_), 4.00 (s, 3H, H_5_), 3.85 (t, *J* = 5.8 Hz, 2H, H_6_), 2.36 (s, 3H, H_1_). ^13^C{^1^H} NMR (101 MHz, CDCl_3_) δ 181.5 (C_2_), 165.4 (C_3_), 156.2 (C_4_), 132.4 (C_7_), 118.7 (C_8_), 46.4 (C_6_), 38.6 (C_5_), 26.7 (C_1_). Mp: 143.6–146.1 °C. IR: ν_max_ 3081, 2881, 1588, 1489, 1457, 1385, 1353, 1312, 1147 cm^–1^. HRMS: *m*/*z* calculated for C_8_H_13_N_4_O_3_S_2_^+^, 277.0424 [M + H]^+^. Found *m*/*z* 277.0418, Δ = −2.0 ppm. Rf (30% EtOAc in Pet ether) = 0.16.
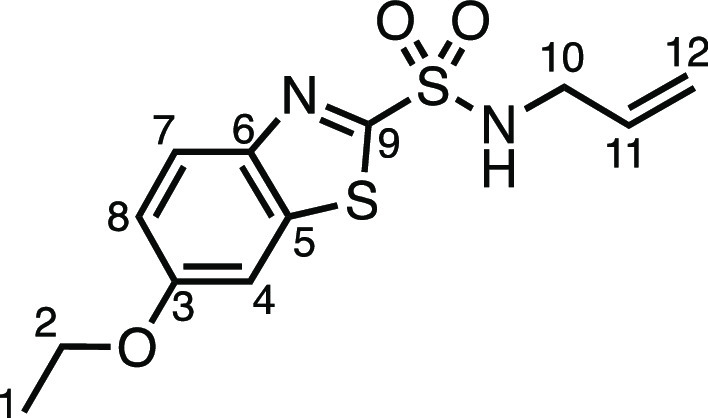


#### *N*-Allyl-6-ethoxybenzo[*d*]thiazole-2-sulfonamide (**1am**)

Allyl bromide (241 μL, 2.0 mmol), K_2_CO_3_ (276 mg, 2.0 mmol), KI (33.2 mg, 0.2 mmol), MeCN (8 mL), and ethoxzolamide (516.6 mg, 2.0 mmol) were subjected to General Procedure B (chromatography eluent: 20–40% EtOAc in Pet ether) to give compound **1am** as a white solid (178 mg, 30%). ^13^C{^1^H} NMR (400 MHz, CDCl_3_) δ 8.02 (d, *J* = 9.1 Hz, 1H, H_7_), 7.33 (d, *J* = 2.5 Hz, 1H, H_4_), 7.18 (dd, *J* = 9.1, 2.5 Hz, 1H, H_8_), 5.80 (ddt*, J* = 17.0, 10.3, 5.8, 1H, H_11_), 5.34 (t, *J* = 6.0 Hz, 1H, N–H), 5.24 (dq, *J* = 17.1, 1.5 Hz, 1H, H_12_), 5.12 (dq, *J* = 10.3, 1.2 Hz, 1H, H_12′_), 4.12 (q, *J* = 7.0 Hz, 2H, H_2_), 3.86 (tt, *J* = 6.1, 1.4 Hz, 2H, H_10_), 1.48 (t, *J* = 7.0 Hz, 3H, H_1_). ^13^C{^1^H} NMR (101 MHz, CDCl_3_) δ 162.7 (C_9_), 159.0 (C_3_), 146.8 (C_5_), 138.4 (C_6_), 132.7 (C_11_), 125.8 (C_7_), 118.4 (C_12_), 118.3 (C_8_), 104.2 (C_4_), 64.4 (C_2_), 46.6 (C_10_), 14.8 (C_1_). Mp: 115.0–116.1 °C. IR: ν_max_ 3111, 2978, 2935, 1599, 1486, 1471, 1432, 1395, 1330, 1254, 1224 cm^–1^. HRMS: *m*/*z* calculated for C_12_H_15_N_2_O_3_S_2_^+^, 299.0519 [M + H]^+^. Found *m*/*z* 299.0524, Δ = 1.7 ppm. Rf (3% EtOAc in Pet ether) = 0.49.

### General Procedure C: Preparation of Exocyclic Olefins **2m, 2n, 2o, 2p, 2q, 2r, 2t, 2u, 2v, 2w, 2x**, and **2z** (Wittig Conditions)

Following a typical Wittig reaction procedure, methyltriphenylphosphonium bromide (1.5 equiv) was suspended in dry tetrahydrofuran (THF) or Et_2_O (0.2 M) under N_2_ and then cooled to 0 °C. Fresh ^*t*^BuOK (1.5 equiv) was added in one portion and the mixture was stirred for 30 min at 0 °C. Ketone was then added dropwise if liquid or in small portions if solid. After 1 h, the reaction mixture was warmed to room temperature and stirred for a further 16 h. The crude product mixture was then filtered and the filtrate then poured into aqueous saturated NH_4_Cl solution. The two phases were separated and the aqueous phase was extracted with diethyl ether (×3). The combined organic phases were washed with brine, dried (MgSO_4_), filtered, and the solvent was removed *in vacuo*. The crude residue was then purified by silica flash column chromatography to yield the desired alkene precursor. The literature data for compounds **2m**,^28^**2n**,^29^**2o**,^30^**2p**,^31^**2q**,^32^**2r**,^33^**2t**,^34^**2u**,^35^**2x**,^34^ and **2z**(36) matched the data obtained using this procedure.
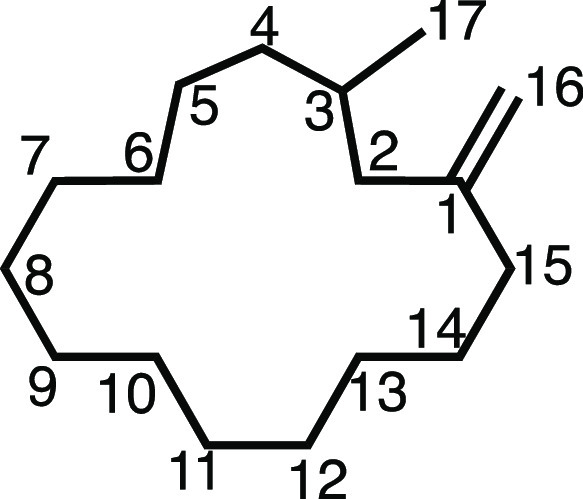


#### 1-Methyl-3-methylenecyclopentadecane (**2v**)

Methyltriphenylphosphonium bromide (1.607 g, 4.5 mmol), KO^t^Bu (505 mg, 4.5 mmol), dry THF (15 mL), and muscone (715.2 mg, 776 μL, 3.0 mmol) were subjected to General Procedure C (chromatography eluent: 0–3% Et_2_O in Pet ether) to give **2v** as a colorless oil (609.3 mg, 86%). ^1^H NMR (400 MHz, CDCl_3_) δ 4.74 (s, 1H, H_16_), 4.69 (s, 1H, H_16′_), 2.10 (dd, *J* = 13.5, 6.0 Hz, 1H, H_2_), 2.06–1.89 (m, 2H, H_15_), 1.76 (dd, *J* = 13.4, 7.9 Hz, 1H, H_2′_), 1.68–1.56 (m, 1H, H_3_), 1.53–1.04 (m, 22H, H_4–14_), 0.84 (d, *J* = 6.6 Hz, 3H, H_17_). ^13^C{^1^H} NMR (101 MHz, CDCl_3_) δ 149.4 (C_1_), 110.9 (C_16_), 44.3 (C_2_), 35.6 (C_4_), 35.2 (C_15_), 29.6 (C_3_), 27.7 (C_5–14_), 27.2 (C_5–14_), 27.0 (C_5–14_), 27.0 (C_5–14_), 26.9 (C_5–14_), 26.8 (2C, C_5–14_), 26.8 (C_5–14_), 26.6 (C_5–14_), 25.4 (C_5–14_), 20.4 (C_17_). IR: ν_max_ 2923, 2855, 1642, 1458, 1375 cm^–1^. HRMS: *m*/*z* calculated for C_17_H_32_Na^+^, 259.2396 [M + Na]^+^. Found *m*/*z* 259.2395, Δ = −0.5 ppm. Rf (5% Et_2_O in Pet ether) = 0.78.
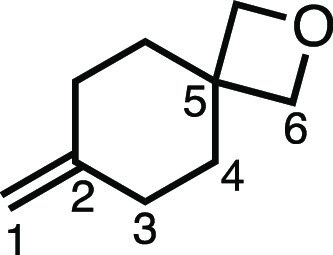


#### 7-Methylene-2-oxaspiro[3.5]nonane (**2w**)

Methyltriphenylphosphonium bromide (0.4432 g, 1.21 mmol), KO^t^Bu (136 mg, 1.21 mmol), dry Et_2_O (4 mL), and 2-oxaspiro[3.5]nonan-7-one (113.0 mg, 0.81 mmol) were subjected to General Procedure C to give **2w** as a colorless oil (105.0 mg, 94%). ^1^H NMR (400 MHz, CDCl_3_) δ 4.64 (s, 2H, H_1_), 4.42 (s, 4H, H_6_), 2.15–2.03 (m, 4H, H_3_), 1.90–1.77 (m, 4H, H_4_). ^13^C{^1^H} NMR (126 MHz, CDCl_3_) δ 147.5 (C_2_), 108.0 (C_1_), 82.0 (C_6_), 40.2 (C_5_), 36.6 (C_4_), 31.4 (C_3_). HRMS: *m*/*z* calculated for C_9_H_14_ONH_4_^+^, 156.1383 [M + NH_4_]^+^. Found *m*/*z* 156.1376, Δ = −4.5 ppm. Rf (10% EtOAc in Pet ether) = 0.33.

### General Procedure D: Preparation of Heterocyclic Exocyclic Olefins **2aa, 2ab, 2ac**, and **2ag**

To the *N*-Boc-protected olefin*, 4.0 N HCl in 1,4-dioxane was added dropwise (4 equiv) over a period of 10 min to keep the reaction temperature below 30 °C and then stirred at room temperature for 3 h. The solvent was then removed *in vacuo* to yield crude aminium chloride. The crude was then taken up in DCM (0.67 M). To this solution was added water (3:2 DCM:H_2_O solvent), K_2_CO_3_ (2.4 equiv), and the corresponding sulfonyl chloride (1.0 equiv) with vigorous stirring. After stirring for 16 h, the layers were separated and the organic layer extracted with DCM (×2). The combined organic layers were then washed with brine, dried (MgSO_4_), and the solvent then removed *in vacuo*. The crude residue was then purified by flash chromatography to yield the desired heterocyclic olefin. The literature data for compounds **2aa**(37) and **2ac**(37) matched the data obtained using this procedure.

*Where the *N*-Boc-protected olefin was not commercially available, it was instead prepared from the corresponding ketone using the Wittig reaction conditions of General Procedure C.
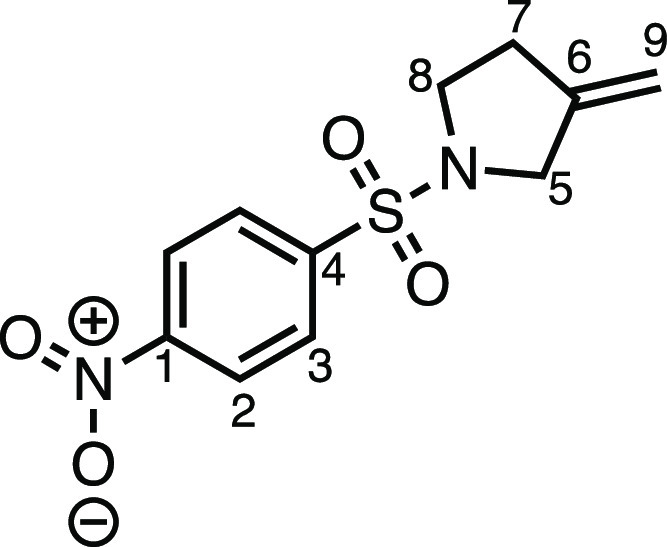


#### 3-Methylene-1-((4-nitrophenyl)sulfonyl)pyrrolidine (**2ab**)

*tert*-Butyl 3-methylenepyrrolidine-1-carboxylate (1.833 g, 10.0 mmol), 4.0 N HCl in 1,4-dioxane (20 mL), K_2_CO_3_ (3.33 g, 24.0 mmol), and nosyl chloride (2.22 g, 10.0 mmol) were subjected to General Procedure D (chromatography eluent 20% EtOAc in Pet ether) to give **2ab** as a white solid (1.655 g, 62%). ^1^H NMR (400 MHz, CDCl_3_) δ 8.39 (app d, *J* = 8.8 Hz, 2H, H_2_), 8.01 (app d, *J* = 8.8 Hz, 2H, H_3_), 5.01–4.96 (m, 1H, H_9_), 4.96–4.91 (m, 1H, H_9′_) 3.85 (s, 2H, H_5_), 3.37 (t, *J* = 7.1 Hz, 2H, H_8_), 2.53 (t, *J* = 7.1 Hz, 2H, H_7_). ^13^C{^1^H} NMR (101 MHz, CDCl_3_) δ 150.4 (C_1_), 143.1 (C_6_), 142.3 (C_4_), 128.9 (C_3_), 124.5 (C_2_), 108.4 (C_9_), 51.9 (C_5_), 48.3 (C_8_), 31.9 (C_7_). Mp: 109.0–111.6 °C. IR: ν_max_ 3110, 1535, 1346, 1314, 1161 cm^–1^. HRMS: *m*/*z* calculated for C_11_H_13_N_2_O_4_S^+^, 269.0590 [M + H]^+^. Found *m*/*z* 269.0579, Δ = −4.1 ppm.
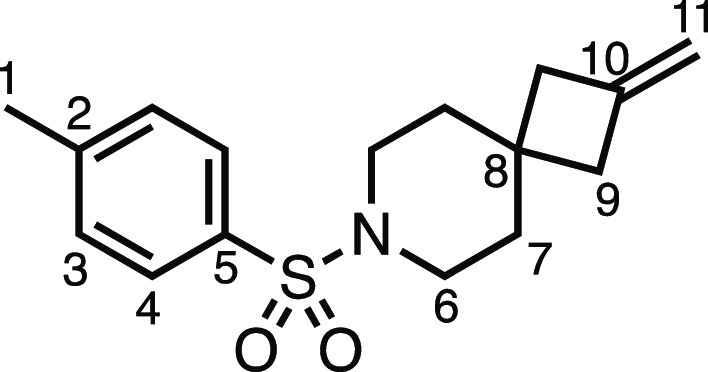


#### 2-Methylene-7-tosyl-7-azaspiro[3.5]nonane (**2ag**)

*tert*-Butyl 2-methylene-7-azaspiro[3.5]nonane-7-carboxylate (2.37 g, 10.0 mmol), 4.0 N HCl in 1,4-dioxane (20 mL), K_2_CO_3_ (3.32 g, 24.0 mmol), and tosyl chloride (1.90 g, 10.0 mmol) were subjected to General Procedure D (chromatography eluent 30% Et_2_O in Pet ether) to give **2ab** as a white solid (2.27 g, 78%). ^1^H NMR (400 MHz, CDCl_3_) δ 7.65 (app d, *J* = 8.0 Hz, 2H, H_4_), 7.34 (app d, *J* = 8.0 Hz, 2H, H_3_), 4.80 (s, 2H, H_11_), 3.04–2.85 (m, 4H, H_6_), 2.46 (s, 3H, H_1_), 2.33 (s, 4H, H_9_), 1.78–1.64 (m, 4H, H_7_). ^13^C{^1^H} NMR (101 MHz, CDCl_3_) δ 143.9 (C_10_), 143.5 (C_5_), 133.5 (C_2_), 129.7 (C_3_), 127.8 (C_4_), 108.0 (C_11_), 43.7 (C_6_), 41.8 (C_9_), 36.1 (C_7_), 33.0 (C_8_), 21.7 (C_1_). Mp: 129.6–132.9 °C. IR: ν_max_ 2920, 2846, 1462, 1348, 1328, 1158 cm^–1^. HRMS: *m*/*z* calculated for C_16_H_22_NO_2_S^+^, 292.1366 [M + H]^+^. Found *m*/*z* 292.1362, Δ = −1.3 ppm. Rf (30% Et_2_O in Pet ether) = 0.40.

### General Procedure E: Preparation of **β**-Halomethylated **γ**-Spirocyclic Pyrrolidines

**Conditions E1**: In a 6 mL microwave vial was placed *N*-allylsulfonamide (0.2 mmol, 1.0 equiv), 1,3-dichloro-5,5′-dimethylhydantoin (0.3 mmol, 1.5 equiv), and [Ir(dF(CF_3_)ppy)_2_(dtbpy)]PF_6_ (0.001 mmol, 0.5 mol %). The vial was then capped, degassed, and backfilled with N_2_ (×3), and then dry DCM (1 mL, 0.2 M) was added. The resulting solution was then stirred at room temperature for 10 min. The reaction mixture was checked by TLC (20% EtOAc in P.E.) and then dry DCM (3 mL, 0.05 M total) and the corresponding olefin (0.6 mmol, 3.0 equiv)* were added. The reaction mixture was then stirred under 470 nm LED irradiation with fan-cooling (see the SI for the setup) for 3 h. The crude reaction mixture was then concentrated *in vacuo* and then purified by silica flash column chromatography to yield the desired spirocyclic pyrrolidine product. **Conditions E2**: Same conditions, but using 4CzIPN (0.001 mol, 0.5 mol %) as the photosensitizer. **Conditions E3**: Same conditions, but using 1,3-dibromo-5,5′-dimethylhydantoin (0.3 mmol, 1.5 equiv) as the halogenating reagent. **Conditions E4**: Same conditions, but using 1,3-diiodo-5,5′-dimethylhydantoin (0.3 mmol, 1.5 equiv) as the halogenating reagent.

*If the olefin was a solid, it was predissolved in the aforementioned 3 mL of dry DCM added to the reaction mixture after the 10 min prestir, ensuring that the solution was flushed with N_2_.

### General Procedure F: Preparation of **β**-Methylated **γ**-Spirocyclic Pyrrolidines

Conditions F1: In a 6 mL microwave vial was placed *N*-allylsulfonamide (0.2 mmol, 1.0 equiv), diiodo-5,5′-dimethylhydantoin (0.3 mmol, 1.5 equiv), and [Ir(dF(CF_3_)ppy)_2_(dtbpy)]PF_6_ (0.001 mmol, 0.5 mol %). The vial was then capped, degassed, and backfilled with N_2_ (×3), and then dry DCM (1 mL, 0.2 M) was added. The resulting solution was then stirred at room temperature for 10 min. The reaction mixture was checked by TLC (20% EtOAc in P.E.), and then dry DCM (3 mL, 0.05 M total) and the corresponding olefin (0.6 mmol, 3.0 equiv)* were added. The reaction mixture was then stirred under 470 nm LED irradiation with fan-cooling (see the SI for the setup) for 3 h. Tris(trimethylsilyl)silane (247 μL, 0.8 mmol, 4.0 equiv) was then added by syringe to the reaction mixture and the resulting solution was immediately resubjected to 470 nm LED irradiation with fan-cooling for 14 h. The crude reaction mixture was then concentrated *in vacuo* and then purified by silica flash column chromatography to yield the desired spirocyclic pyrrolidine product. **Conditions F2**: Same conditions, but using 1,3-dibromo-5,5′-dimethylhydantoin (0.3 mmol, 1.5 equiv) as the halogenating reagent.

*If the olefin was a solid, it was predissolved in the aforementioned 3 mL of dry DCM added to the reaction mixture after the 10 min prestir, ensuring that the solution was flushed with N_2_.

### Scale-Up Procedure

Scale-up procedure was executed using a Vapourtec E-series fitted with a UV-150 photoreactor with 61 W 365 nm LED and a 10 mL heated jacket coil reactor (see the SI for the detailed setup). Independent solutions of *N*-allyltoluenesulfonamide **1a** (7.40 g, 35.0 mmol, 0.2 M), 1,3-dibromo-5,5′-dimethylhydantoin (20.01 g, 70 mmol, 0.2 M), and 4-methylene-1-tosylpiperidine **2ad** (26.39 g, 105 mmol, 0.6 M) in dry DCM under nitrogen were prepared (total volume of reagent solutions was 700 mL) and stirred throughout the experiment at room temperature. By use of the embedded peristaltic pumps on the Vapourtec E-series apparatus, the solutions of **1a** and 1,3-dibromo-5,5′-dimethylhydantoin were mixed (1.00 and 2.00 mL min^–1^ flow rates, respectively) at a Y-piece and then passed through a coil reactor (10 mL reactor volume, 28 °C, *t*_R_ = 3.33 min). The output of this reactor was then mixed at a Y-piece with an inlet stream of the **2ad** solution (1.00 mL min^–1^) and the output fed into the UV-150 photoreactor with 61 W 365 nm LED attachment (LED was operated at 100% power). The output was collected via an 8 bar BPR in a 1 L RBF. Upon completion of the reaction (3 h), the solvent of the product solution was removed *in vacuo* and the residue subjected to chromatography (15–25% EtOAc in Pet ether) to produce *bis*-azaheterospirocycle **3ad** as a white crystalline solid (13.20 g, 70%).

### Characterization of Products **3a-al, 4a–d, 5**, and **6**

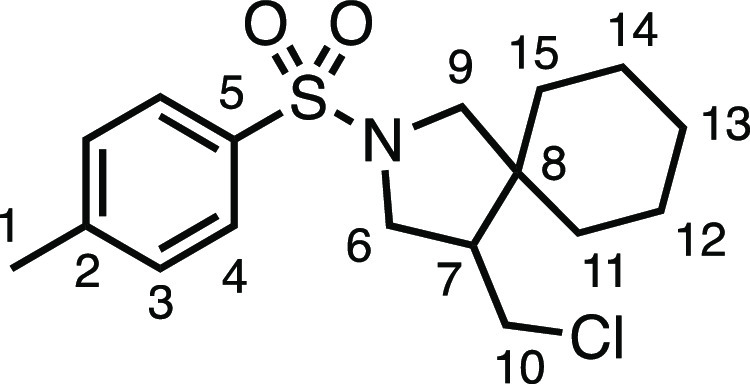



#### 4-(Chloromethyl)-2-tosyl-2-azaspiro[4.5]decane (**3a**)

The compound was prepared according to General Procedure E1. Flash column chromatography (5–30% Et_2_O in Pet ether) afforded the product as a colorless oil (52.0 mg, 76%). ^1^H NMR (400 MHz, CDCl_3_) δ 7.72 (app d, *J* = 8.1 Hz, 2H, H_4_), 7.32 (app d, *J* = 8.1 Hz, 2H, H_3_), 3.56 (dd, *J* = 10.2, 7.4 Hz, 1H, H_6_), 3.51 (dd, *J* = 10.9, 4.1 Hz, 1H, H_10_), 3.32 (d, *J* = 10.1 Hz, 1H, H_9_), 3.22 (dd, *J* = 10.2, 6.9 Hz, 1H, H_6′_), 3.09 (app t, *J* = 10.9 Hz, 1H, H_10′_), 3.04 (d, *J* = 10.1 Hz, 1H, H_9′_), 2.42 (s, 3H, H_1_), 2.06 (dtd, *J* = 10.9, 7.1, 4.2 Hz, 1H, H_7_), 1.60–1.43 (m, 3H, H_11_–H_15_), 1.37–1.00 (m, 7H, H_11_–H_15_). ^13^C{^1^H} NMR (101 MHz, CDCl_3_) δ 143.6 (C_5_), 133.8 (C_2_), 129.8 (C_3_), 127.5 (C_4_), 56.4 (C_9_), 50.5 (C_6_), 50.2 (C_7_), 44.9 (C_8_), 43.4 (C_10_), 35.7 (C_11–15_), 29.1 (C_11–15_), 25.8 (C_11–15_), 23.3 (C_11–15_), 22.8 (C_11–15_), 21.6 (C_1_). IR: ν_max_ 2928, 2854, 1340, 1158 cm^–1^. HRMS: *m*/*z* calculated for C_17_H_25_ClNO_2_S^+^, 342.1289 [M + H]^+^. Found *m*/*z* 342.1295, Δ = 1.6 ppm. Rf (30% Et_2_O in Pet ether) = 0.35.
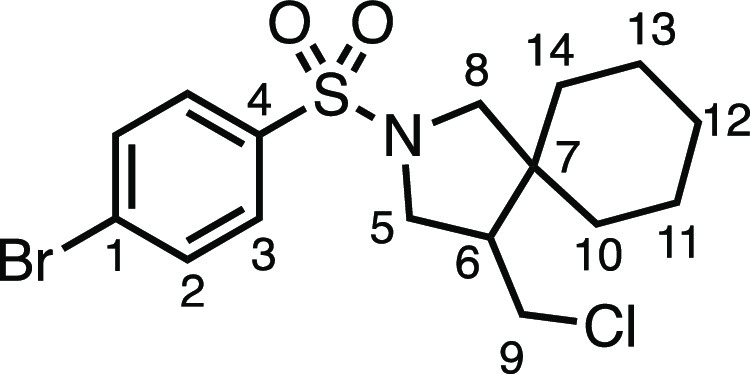


#### 2-((4-Bromophenyl)sulfonyl)-4-(chloromethyl)-2-azaspiro[4.5]decane (**3b**)

The compound was prepared according to General Procedure E1. Flash column chromatography (5–10% EtOAc in Pet ether) afforded the product as a white solid (22.7 mg, 28%). ^1^H NMR (400 MHz, CDCl_3_) δ 7.77–7.60 (m, 4H, H_2_ + H_3_), 3.58 (dd, *J* = 10.7, 7.8 Hz, 1H, H_5_), 3.54 (dd, *J* = 11.1, 3.8 Hz, 1H, H_9_), 3.34 (d, *J* = 10.1 Hz, 1H, H_8_), 3.25 (dd, *J* = 10.3, 6.9 Hz, 1H, H_5′_), 3.14 (t, *J* = 10.8 Hz, 1H, H_9′_), 3.05 (d, *J* = 10.1 Hz, 1H, H_8′_), 2.11 (dtd, *J* = 11.0, 7.1, 4.2 Hz, 1H, H_6_), 1.61–1.46 (m, 3H, H_10–14_), 1.40–1.32 (m, 1H, H_10–14_), 1.27–1.06 (m, 6H, H_10–14_). ^13^C{^1^H} NMR (101 MHz, CDCl_3_) δ 136.1 (C_4_), 132.5 (C_2_), 129.0 (C_3_), 127.9 (C_1_), 56.5 (C_8_), 50.6 (C_5_), 50.2 (C_6_), 45.0 (C_7_), 43.4 (C_9_), 35.7 (C_10–14_), 29.1 (C_10–14_), 25.8 (C_10–14_), 23.4 (C_10–14_), 22.8 (C_10–14_). Mp: 127.9–130.9 °C. IR: ν_max_ 2935, 2852, 1333, 1160, 1142 cm^–1^. HRMS: *m*/*z* calculated for C_16_H_22_BrClNO_2_S^+^, 406.0238 [M + H]^+^. Found *m*/*z* 406.0241, Δ = 0.7 ppm. Rf (10% EtOAc in Pet ether) = 0.12.
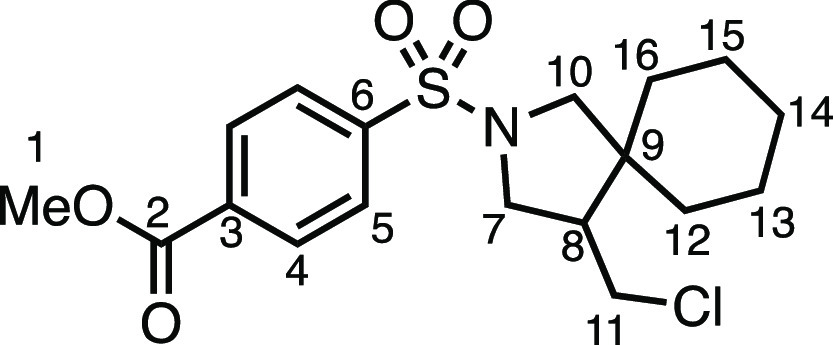


#### Methyl 4-((4-(Chloromethyl)-2-azaspiro[4.5]decan-2-yl)sulfonyl)benzoate (**3c**)

The compound was prepared according to General Procedure E1. Flash column chromatography (10% EtOAc in Pet ether) afforded the product as a white solid (57.2 mg, 74%). ^1^H NMR (400 MHz, CDCl_3_) δ 8.18 (app d, *J* = 8.4 Hz, 2H, H_4_), 7.90 (app d, *J* = 8.4 Hz, 2H, H_5_), 3.95 (s, 3H, H_1_), 3.59 (dd, *J* = 10.3, 7.4 Hz, 1H, H_7_), 3.51 (dd, *J* = 10.9, 4.0 Hz, 1H, H_11_), 3.34 (d, *J* = 10.1 Hz, 1H, H_10_), 3.25 (dd, *J* = 10.3, 6.9 Hz, 1H, H_7′_), 3.10 (app t, *J* = 10.8 Hz, 1H, H_11′_), 3.07 (d, *J* = 10.1 Hz, 1H, H_10′_), 2.08 (dtd, *J* = 10.9, 7.0, 4.1 Hz, 1H, H_8_), 1.60–0.99 (m, 10H, H_12–16_). ^13^C{^1^H} NMR (101 MHz, CDCl_3_) δ 165.8 (C_2_), 140.8 (C_3_), 134.0 (C_6_), 130.4 (C_4_), 127.4 (C_5_), 56.4 (C_10_), 52.8 (C_1_), 50.5 (C_7_), 50.1 (C_8_), 44.9 (C_9_), 43.3 (C_11_), 35.6 (C_12–16_), 29.0 (C_12–16_), 25.7 (C_12–16_), 23.3 (C_12–16_), 22.7 (C_12–16_). Mp: 94.0–96.0 °C. IR: ν_max_ 2932, 2849, 1724, 1346, 1277, 1163 cm^–1^. HRMS: *m*/*z* calculated for C_18_H_25_ClNO_4_S^+^, 386.1191 [M + H]^+^. Found *m*/*z* 386.1189, Δ = 0.5 ppm. Rf (30% EtOAc in Pet ether) = 0.35.
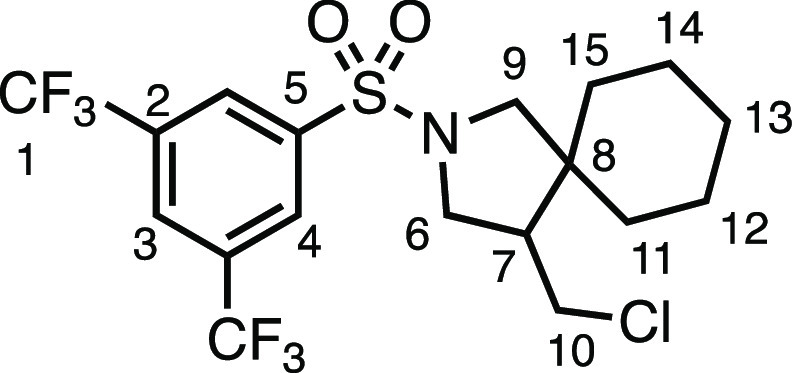


#### 2-((3,5-Bis(trifluoromethyl)phenyl)sulfonyl)-4-(chloromethyl)-2-azaspiro[4.5]decane (**3d**)

The compound was prepared according to General Procedure E1. Flash column chromatography (5% EtOAc in Pet ether) afforded the product as a white solid (61.5 mg, 66%). ^1^H NMR (400 MHz, CDCl_3_) δ 8.28 (s, 2H, H_4_), 8.09 (s, 1H, H_3_), 3.62 (dd, *J* = 10.3, 7.4 Hz, 1H, H_6_), 3.56 (dd, *J* = 11.0, 4.1 Hz, 1H, H_10_), 3.41 (d, *J* = 10.2 Hz, 1H, H_9_), 3.29 (dd, *J* = 10.3, 6.6 Hz, 1H, H_6′_), 3.24–3.11 (app t, 1H, *J* = 10.8 Hz, H_10′_), 3.24–3.11 (d, *J* = 10.2 Hz, 1H, H_9′_), 2.18 (dtd, *J* = 10.8, 7.0, 4.1 Hz, 1H, H_7_), 1.63–1.48 (m, 3H, H_11–15_), 1.43–1.34 (m, 1H, H_11–15_), 1.32–1.10 (m, 6H, H_11–15_). ^13^C{^1^H} NMR (101 MHz, CDCl_3_) δ 140.5 (C_5_), 133.1 (q, *J* = 34.5 Hz, C_2_), 127.5 (q, 3.9 Hz, C_4_), 126.3 (hept., *J* = 3.4 Hz, C_3_) 122.6 (q, *J* = 273.4 Hz, C_1_), 56.7 (C_9_), 50.5 (C_6_), 50.0 (C_7_), 45.1 (C_8_), 43.1 (C_10_), 35.6 (C_11–15_), 29.1 (C_11–15_), 25.8 (C_11–15_), 23.2 (C_11–15_), 22.7 (C_11–15_). Mp: 111.0–114.0 °C. IR: ν_max_ 3084, 2940, 2867, 1353, 1283, 1269, 1166, 1127 cm^–1^. HRMS: *m*/*z* calculated for C_18_H_21_ClF_6_NO_2_S^+^, 464.0880 [M + H]^+^. Found *m*/*z* 464.0881, Δ = 0.2 ppm. Rf (10% EtOAc in Pet ether) = 0.45.
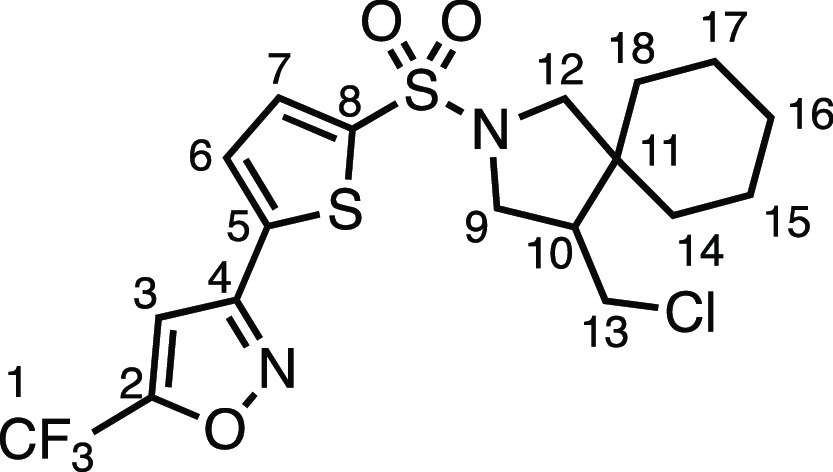


#### 3-(5-((4-(Chloromethyl)-2-azaspiro[4.5]decan-2-yl)sulfonyl)thiophen-2-yl)-5-(trifluoromethyl)isoxazole (**3e**)

The compound was prepared according to General Procedure E1. Flash column chromatography (5–10% EtOAc in Pet ether) afforded the product as a colorless oil (32.0 mg, 34%). ^1^H NMR (400 MHz, CDCl_3_) δ 7.61 (d, *J* = 3.9 Hz, 1H, H_7_), 7.52 (d, *J* = 3.9 Hz, 1H, H_6_), 6.99 (s, 1H, H_3_), 3.67 (dd, *J* = 10.5, 7.5 Hz, 1H, H_9_), 3.57 (dd, *J* = 10.9, 4.0 Hz, 1H, H_13_), 3.44 (d, *J* = 10.2 Hz, 1H, H_12_), 3.35 (dd, *J* = 10.5, 6.9 Hz, 1H, H_9′_), 3.20 (app t, *J* = 10.9 Hz, 1H, H_13′_), 3.18 (d, *J* = 10.2 Hz, 1H, H_12′_), 2.16 (dtd, *J* = 11.0, 7.1, 4.2 Hz, 1H, H_10_), 1.60–1.11 (m, 10H, H_14–18_). ^13^C{^1^H} NMR (101 MHz, CDCl_3_) δ 160.1 (q, *J* = 43.2 Hz, C_2_), 156.9 (C_4_), 140.0 (C_8_), 134.4 (C_5_), 132.1 (C_7_), 128.5 (C_6_), 117.7 (q, *J* = 270.9 Hz, C_1_), 103.7 (app d, *J* = 2.1 Hz, C_3_), 56.7 (C_12_), 50.8 (C_9_), 50.2 (C_10_), 45.2 (C_11_), 43.3 (C_13_), 35.7 (C_14–18_), 29.2 (C_14–18_), 25.8 (C_14–18_), 23.4 (C_14–18_), 22.8 (C_14–18_). IR: ν_max_ 2932, 1553, 1350, 1314, 1153 cm^–1^. HRMS: *m*/*z* calculated for C_18_H_21_ClF_3_N_2_O_3_S_2_^+^, 469.0629 [M + H]^+^. Found *m*/*z* 469.0628, Δ = −0.2 ppm. Rf (10% EtOAc in Pet ether) = 0.12.
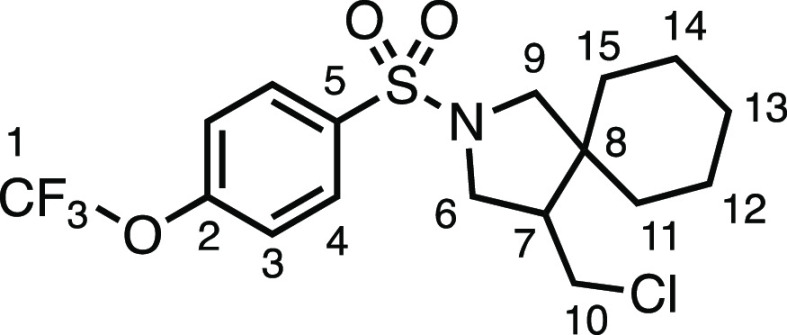


#### 4-(Chloromethyl)-2-((4-(trifluoromethoxy)phenyl)sulfonyl)-2-azaspiro[4.5]decane (**3f**)

The compound was prepared according to General Procedure E1. Flash column chromatography (5–10% EtOAc in Pet ether) afforded the product as a colorless oil (22.1 mg, 27%). ^1^H NMR (400 MHz, CDCl_3_) δ 7.90 (app d, *J* = 8.6 Hz, 2H, H_3_), 7.37 (app d, *J* = 8.6 Hz, 2H, H_4_), 3.60 (dd, *J* = 10.3, 7.4 Hz, 1H, H_6_), 3.54 (dd, *J* = 10.9, 4.0 Hz, 1H, H_10_), 3.35 (d, *J* = 10.1 Hz, 1H, H_9_), 3.26 (dd, *J* = 10.3, 6.9 Hz, 1H, H_6′_), 3.14 (t, *J* = 10.8 Hz, 1H, H_10′_), 3.08 (d, *J* = 10.1 Hz, 1H, H_9′_), 2.12 (dtd, *J* = 11.0, 7.1, 4.2 Hz, 1H, H_7_), 1.52 (m, 3H, H_11–15_), 1.39–1.31 (m, 1H, H_11–15_), 1.26–1.05 (m, 6H, H_11–15_). ^13^C{^1^H} NMR (101 MHz, CDCl_3_) δ 152.4 (q, *J* = 1.9 Hz, C_2_), 135.5 (C_5_), 129.5 (C_4_), 121.1 (q, *J* = 1.1 Hz, C_3_), 120.4 (q, *J* = 259.3 Hz, C_1_), 56.4 (C_9_), 50.6 (C_6_), 50.2 (C_7_), 45.0 (C_8_), 43.3 (C_10_), 35.6 (C_11–15_), 29.1 (C_11–15_), 25.8 (C_11–15_), 23.3 (C_11–15_), 22.8 (C_11–15_). IR: ν_max_ 2931, 2858, 1347, 1251, 1208, 1157 cm^–1^. HRMS: *m*/*z* calculated for C_17_H_22_ClF_3_NO_3_S^+^, 412.0955 [M + H]^+^. Found *m*/*z* 412.0958, Δ = 0.7 ppm. Rf (10% EtOAc in Pet ether) = 0.23.
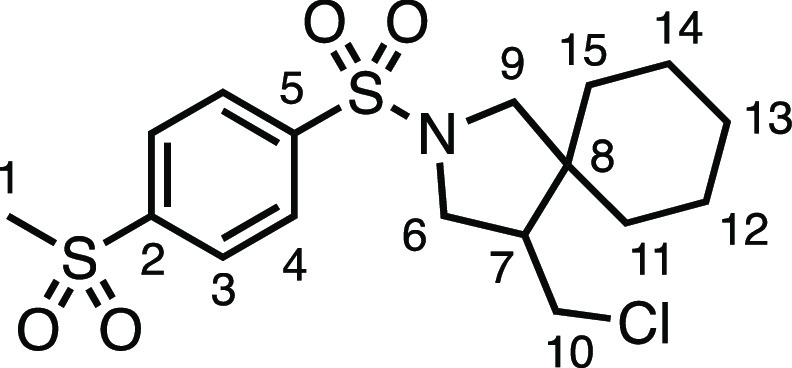


#### 4-(Chloromethyl)-2-((4-(methylsulfonyl)phenyl)sulfonyl)-2-azaspiro[4.5]decane (**3g**)

The compound was prepared according to General Procedure E1. Flash column chromatography (30–40% EtOAc in Pet ether) followed by recrystallization by vapor diffusion (DCM with Pet ether antisolvent) afforded the product as a white solid (76.2 mg, 83%). ^1^H NMR (400 MHz, CDCl_3_) δ 8.12 (app d, *J* = 8.3 Hz, 2H, H_4_), 8.03 (app d, *J* = 8.3 Hz, 2H, H_3_), 3.60 (dd, *J* = 10.2, 7.5 Hz, 1H, H_6_), 3.54 (dd, *J* = 10.9, 4.0 Hz, 1H, H_10_), 3.37 (d, *J* = 10.1 Hz, 1H, H_9_), 3.27 (dd, *J* = 10.2, 6.9 Hz, 1H, H_6′_), 3.14 (t, *J* = 10.9 Hz, 1H, H_10′_), 3.10 (s, 3H, H_1_), 3.08 (d, *J* = 10.1 Hz, 1H, H_9′_), 2.13 (dtd, *J* = 10.9, 7.5, 4.0 Hz, 1H, H_7_), 1.73–1.43 (m, 3H, H_11_–H_15_), 1.41–1.04 (m, 7H, H_11_–H_15_). ^13^C{^1^H} NMR (101 MHz, CDCl_3_) δ 144.4 (C_2_), 142.3 (C_5_), 128.5 (C_3_), 128.3 (C_4_), 56.5 (C_9_), 50.6 (C_6_), 50.0 (C_7_), 45.0 (C_8_), 44.4 (C_1_), 43.3 (C_10_), 35.6 (C_11–15_), 29.0 (C_11–15_), 25.7 (C_11–15_), 23.3 (C_11–15_), 22.8 (C_11–15_). Mp: 170.0–174.1 °C. IR: ν_max_ 2923, 2851, 1336, 1307, 1285, 1164, 1145 cm^–1^. HRMS: *m*/*z* calculated for C_17_H_25_ClNO_4_S_2_^+^, 406.0908 [M + H]^+^. Found *m*/*z* 406.0915, Δ = 1.8 ppm. Rf (40% EtOAc in Pet ether) = 0.28.
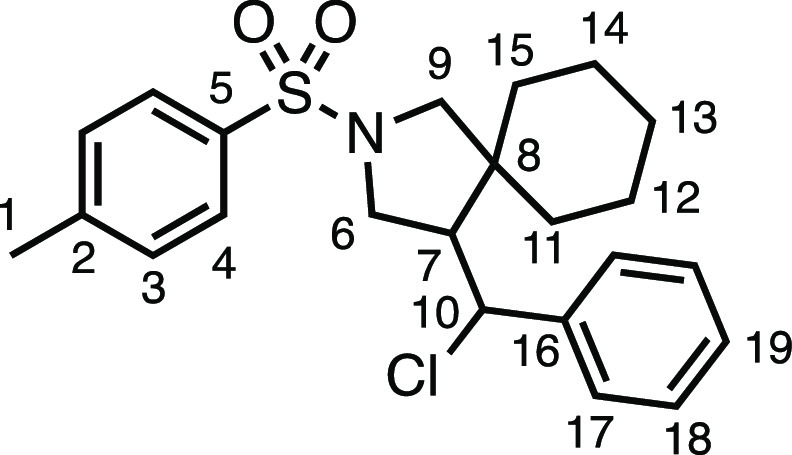


#### 4-(Chloro(phenyl)methyl)-2-tosyl-2-azaspiro[4.5]decane (**3h**)

The compound was prepared according to General Procedure E1. Flash column chromatography (5–10% EtOAc in Pet ether) afforded a single diastereomer of the product as a white solid (53.0 mg, 63%). ^1^H NMR (400 MHz, CDCl_3_) δ 7.76 (app d, *J* = 8.2 Hz, 2H, H_4_), 7.46–7.20 (m, 7H, H_3_ + H_17_ + H_18_ + H_19_), 4.82 (d, *J* = 9.4 Hz, 1H, H_10_), 3.79 (dd, *J* = 10.3, 8.1 Hz, 1H, H_6_), 3.61 (d, *J* = 10.1 Hz, 1H, H_9_), 3.34 (t, *J* = 10.0 Hz, 1H, H_6′_), 2.93 (d, *J* = 10.1 Hz, 1H, H_9′_), 2.55–2.35 (m, 4H, H_1_ + H_7_), 1.45 (s, 1H, H_11–15_), 1.30–0.68 (m, 8H, H_11–15_), 0.43 (td, *J* = 13.2, 3.8 Hz, 1H, H_11–15_). ^13^C{^1^H} NMR (126 MHz, CDCl_3_) δ 143.6 (C_5_), 140.6 (C_16_), 134.0 (C_2_), 129.8 (C_3_), 128.9 (C_19_), 128.8 (C_4_ or C_17_ or C_18_), 127.6 (C_4_ or C_17_ or C_18_), 127.6 (C_4_ or C_17_ or C_18_), 63.1 (C_10_), 56.9 (C_9_), 54.9 (C_7_), 51.1 (C_6_), 45.2 (C_8_), 35.8 (C_11–15_), 27.9 (C_11–15_), 25.7 (C_11–15_), 23.6 (C_11–15_), 22.5 (C_11–15_), 21.7 (C_1_). Mp: 139.4–144.8 °C. IR: ν_max_ 2943, 1342, 1168 cm^–1^. HRMS: *m*/*z* calculated for C_23_H_29_ClNO_2_S^+^, 418.1602 [M + H]^+^. Found *m*/*z* 418.1586, Δ = −3.8 ppm. Rf (20% EtOAc in Pet ether) = 0.39.
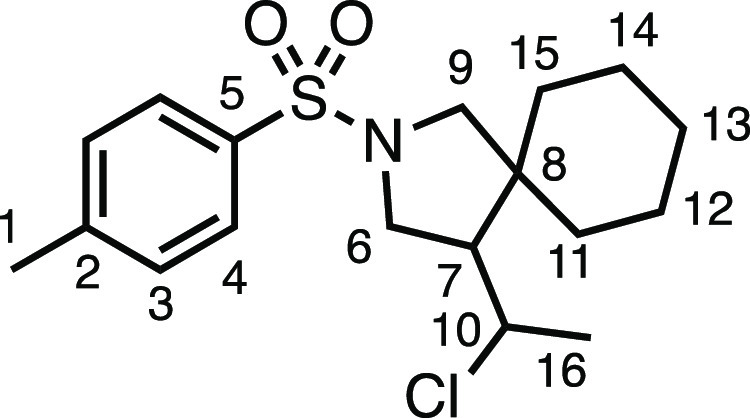


#### 4-(1-Chloroethyl)-2-tosyl-2-azaspiro[4.5]decane (**3i**)

The compound was prepared according to General Procedure E1. Flash column chromatography (5–10% EtOAc in Pet ether) afforded two separated diastereoisomers (3:2 d.r.) (total 26.8 mg, 38%). *Major diastereoisomer* (colorless oil, 16.0 mg): ^1^H NMR (400 MHz, CDCl_3_) δ 7.73 (app d, *J* = 8.1 Hz, 2H, H_4_), 7.31 (app d, *J* = 8.1 Hz, 2H, H_3_), 4.12–4.03 (m, 1H, H_10_), 3.61 (dd, *J* = 10.3, 8.4 Hz, 1H, H_6_), 3.36 (d, *J* = 9.7 Hz, 1H, H_9_), 3.28 (dd, *J* = 10.3, 6.9 Hz, 1H, H_6′_), 3.14 (d, *J* = 9.7 Hz, 1H, H_9′_), 2.42 (s, 3H, H_1_), 1.97–1.89 (m, 1H, H_7_), 1.62–1.48 (m, 3H, H_11–15_), 1.45 (d, *J* = 6.7 Hz, 3H, H_16_), 1.41–1.10 (m, 7H, H_11–15_). ^13^C{^1^H} NMR (101 MHz, CDCl_3_) δ 143.4 (C_5_), 134.0 (C_2_), 129.7 (C_3_), 127.7 (C_4_), 57.0 (C_10_), 56.4 (C_9_), 54.7 (C_7_), 48.9 (C_6_), 45.0 (C_8_), 36.9 (C_11–15_), 29.0 (C_11–15_), 25.9 (C_11–15_), 25.8 (C_16_), 23.4 (C_11–15_), 23.0 (C_11–15_), 21.7 (C_1_). HRMS: *m*/*z* calculated for C_18_H_27_ClNO_2_S^+^, 356.1445. [M + H]^+^. Found *m*/*z* 356.1447, Δ = 0.5 ppm. Rf (20% EtOAc in Pet ether) = 0.36. *Minor diastereoisomer* (colorless oil, 10.8 mg): ^1^H NMR (400 MHz, CDCl_3_) δ 7.72 (d, *J* = 8.1 Hz, 2H, H_4_), 7.33 (d, *J* = 8.1 Hz, 2H, H_3_), 4.01 (p, *J* = 6.6 Hz, 1H, H_10_), 3.47–3.35 (m, 2H, H_6_ + H_9_), 3.05 (t, *J* = 10.0 Hz, 2H, H_6′_ + H_9′_), 2.44 (s, 3H, H_1_), 2.08 (q, *J* = 8.1 Hz, 1H, H_7_), 1.84–1.74 (m, 1H, H_11–16_), 1.56–1.06 (m, 12H, H_11–16_). ^13^C{^1^H} NMR (101 MHz, CDCl_3_) δ 143.7 (C_5_), 133.7 (C_2_), 129.8 (C_3_), 127.6 (C_4_), 56.7 (C_9_), 56.0 (C_10_), 55.2 (C_7_), 49.3 (C_6_), 45.1 (C_8_), 36.8 (C_11–15_), 28.5 (C_11–15_), 25.8 (C_11–15_), 24.1 (C_16_), 23.8 (C_11–15_), 22.9 (C_11–15_), 21.7 (C_1_). HRMS: *m*/*z* calculated for C_18_H_27_ClNO_2_S^+^, 356.1445 [M + H]^+^. Found *m*/*z* 356.1447, Δ = 0.5 ppm. Rf (20% EtOAc in Pet ether) = 0.42.
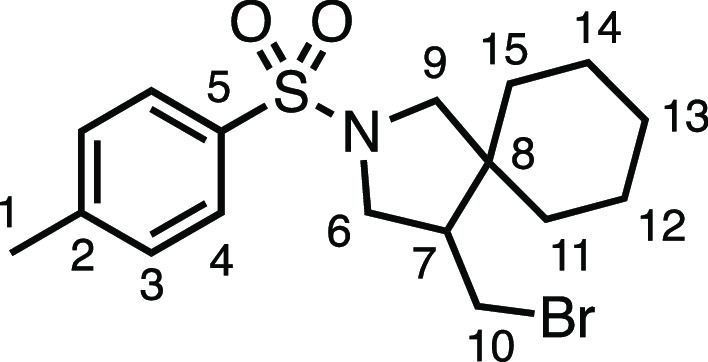


#### 4-(Bromomethyl)-2-tosyl-2-azaspiro[4.5]decane (**3j**)

The compound was prepared according to General Procedure E3. Flash column chromatography (5–10% EtOAc in Pet ether) afforded the product as a colorless oil (54.3 mg, 71%). ^1^H NMR (400 MHz, CDCl_3_) δ 7.72 (app d, *J* = 8.1 Hz, 2H, H_4_), 7.32 (app d, *J* = 8.1 Hz, 2H, H_3_), 3.61 (dd, *J* = 10.3, 7.4 Hz, 1H, H_6_), 3.38 (dd, *J* = 9.9, 3.8 Hz, 1H, H_10_), 3.37 (d, *J* = 10.1 Hz, 1H, H_9_), 3.19 (dd, *J* = 10.3, 7.3 Hz, 1H, H_6′_), 3.03 (d, *J* = 10.1 Hz, 1H, H_9′_), 2.94 (dd, *J* = 11.4, 10.1 Hz, 1H, H_10′_), 2.42 (s, 3H, H_1_), 2.12 (dtd, *J* = 11.2, 7.3, 3.7 Hz, 1H, H_7_), 1.60–1.43 (m, 3H, H_11–15_), 1.37–1.00 (m, 7H, H_11–15_). ^13^C{^1^H} NMR (101 MHz, CDCl_3_) δ 143.5 (C_5_), 133.7 (C_2_), 129.7 (C_3_), 127.4 (C_4_), 56.3 (C_9_), 51.5 (C_6_), 50.5 (C_7_), 45.4 (C_8_), 35.5 (C_11–15_), 31.5 (C_11–15_), 28.8 (C_11–15_), 25.7 (C_11–15_), 22.6 (C_11–15_), 21.6 (C_1_). IR: ν_max_ 2924, 2854, 1340, 1157 cm^–1^. HRMS: *m*/*z* calculated for C_17_H_25_BrNO_2_S^+^, 386.0784 [M + H]^+^. Found *m*/*z* 346.0789, Δ = 1.2 ppm. Rf (10% EtOAc in Pet ether) = 0.29.
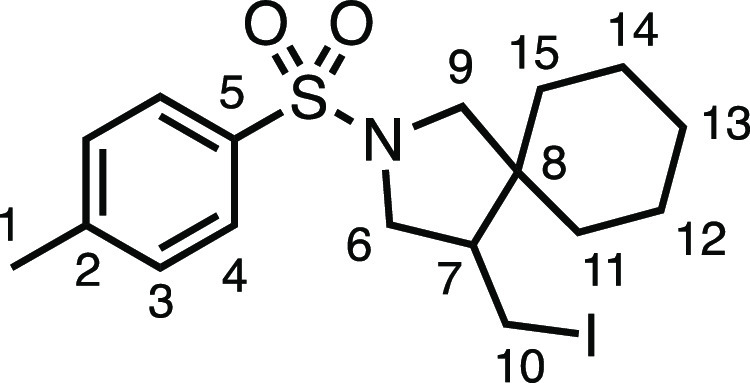


#### 4-(Iodomethyl)-2-tosyl-2-azaspiro[4.5]decane (**3k**)

The compound was prepared according to General Procedure E4. Flash column chromatography (5–10% EtOAc in Pet ether) afforded the product as an off-white crystalline solid (70.4 mg, 81%). ^1^H NMR (400 MHz, CDCl_3_) δ 7.73 (app d, *J* = 8.1 Hz, 2H, H_4_), 7.33 (app d, *J* = 8.1 Hz, 2H, H_3_), 3.67 (dd, *J* = 10.2, 7.4 Hz, 1H, H_6_), 3.45 (d, *J* = 10.1 Hz, 1H, H_9_), 3.18 (dd, *J* = 9.7, 3.4 Hz, 1H, H_10_), 3.10 (dd, *J* = 10.2, 7.9 Hz, 1H, H_6′_), 3.01 (d, *J* = 10.1 Hz, 1H, H_9′_), 2.70 (dd, *J* = 12.1, 9.7 Hz, 1H, H_10′_), 2.43 (s, 3H, H_1_), 2.11 (app dtd, *J* = 11.2, 7.6, 3.4 Hz, 1H, H_7_), 1.60–1.28 (m, 5H, H_11–15_), 1.23–1.04 (m, 5H, H_11–15_). ^13^C{^1^H} NMR (101 MHz, CDCl_3_) δ 143.6 (C_5_), 133.9 (C_2_), 129.8 (C_3_), 127.5 (C_4_), 56.6 (C_9_), 53.2 (C_6_), 51.4 (C_7_), 45.8 (C_8_), 35.5 (C_11–15_), 28.4 (C_11–15_), 25.9 (C_11–15_), 23.5 (C_11–15_), 22.6 (C_11–15_), 21.7 (C_1_), 3.4 (C_10_). Mp: 99.5–102.5 °C. IR: ν_max_ 2925, 2859, 1337, 1160 cm^–1^. HRMS: *m*/*z* calculated for C_17_H_25_INO_2_S^+^, 434.0646 [M + H]^+^. Found *m*/*z* 434.0656, Δ = 2.4 ppm. Rf (20% EtOAc in Pet ether) = 0.59. Spectroscopic data consistent with those previously reported.^38^
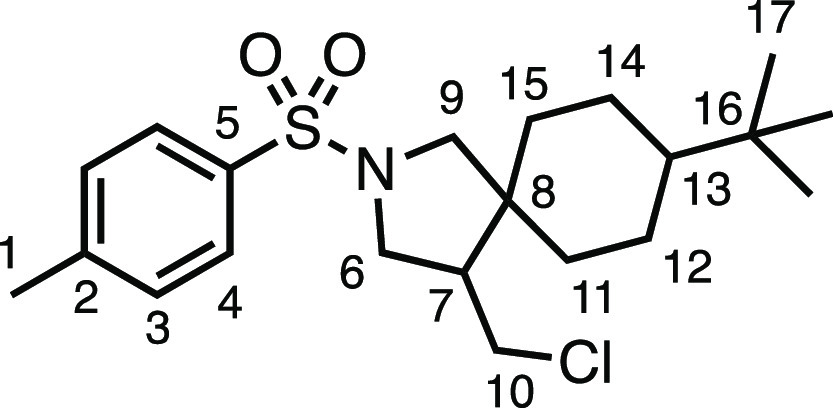


#### 8-(*tert*-Butyl)-4-(chloromethyl)-2-tosyl-2-azaspiro[4.5]decane (**3m**)

The compound was prepared according to General Procedure E1. Flash column chromatography (5–25% Et_2_O in Pet ether) afforded two separate diastereoisomers (4:3 d.r.) (total 57.0 mg, 72%). *Major diastereoisomer* (white solid, 29.0 mg): ^1^H NMR (400 MHz, CDCl_3_) δ 7.72 (app d, *J* = 8.0 Hz, 2H, H_4_), 7.32 (app d, *J* = 8.0 Hz, 2H, H_3_), 3.62 (d, *J* = 11.0 Hz, 1H, H_6_), 3.45 (m, 2H, H_6′_ + H_10_), 3.01 (d, *J* = 9.8 Hz, 1H, H_9_), 2.91 (d, *J* = 9.8 Hz, 1H, H_9′_), 2.73 (t, *J* = 11.2 Hz, 1H, H_10′_), 2.43 (s, 3H, H_1_), 2.27 (dt, *J* = 10.3, 5.2 Hz, 1H, H_7_), 1.68–1.43 (m, 4H, H_11–15_), 1.26–1.14 (m, 1H, H_11–15_), 1.06–0.86 (m, 4H, H_11–15_), 0.81 (s, 9H, H_17_). ^13^C{^1^H} NMR (101 MHz, CDCl_3_) δ 143.6 (C_5_), 133.7 (C_2_), 129.8 (C_3_), 127.6 (C_4_), 59.2 (C_9_), 50.0 (C_6_), 47.7 (C_13_), 44.8 (C_8_), 44.2 (C_10_), 44.0 (C_7_), 36.8 (C_11–15_), 32.5 (C_16_), 30.5 (C_11–15_), 27.6 (C_17_), 24.1 (C_11–15_), 23.4 (C_11–15_), 21.7 (C_1_). Mp: 148.8–150.8 °C. IR: ν_max_ 2944, 1335, 1155 cm^–1^. HRMS: *m*/*z* calculated for C_21_H_33_ClNO_2_S^+^, 398.1915 [M + H]^+^. Found *m*/*z* 398.1921, Δ = 1.4 ppm. Rf (30% Et_2_O in Pet ether) = 0.88. *Minor diastereoisomer* (white solid, 28.0 mg): ^1^H NMR (400 MHz, CDCl_3_) δ 7.73 (app d, *J* = 8.0 Hz, 2H, H_4_), 7.33 (app d, *J* = 8.0 Hz, 2H, H_3_), 3.59 (dd, *J* = 10.2, 7.7 Hz, 1H, H_6_), 3.51 (dd, *J* = 10.8, 4.1 Hz, 1H, H_10_), 3.34 (d, *J* = 10.0 Hz, 1H, H_9_), 3.17 (dd, *J* = 10.5, 5.8 Hz, 1H, H_6′_), 3.14 (app t, *J* = 8.4 Hz 1H, H_10′_), 3.03 (d, *J* = 10.0 Hz, 1H, H_9′_), 2.44 (s, 3H, H_1_), 2.02 (dtd, *J* = 11.6, 7.7, 4.1 Hz, 1H, H_7_), 1.62 (s, 1H, H_11_ or H_12_ or H_14_ or H_15_), 1.41–1.22 (m, 3H, H_11–15_), 1.09–0.85 (m, 5H, H_11–15_), 0.82 (s, 9H, H_17_). ^13^C{^1^H} NMR (101 MHz, CDCl_3_) δ 143.7 (C_5_), 133.8 (C_2_), 129.8 (C_3_), 127.6 (C_4_), 55.9 (C_9_), 51.0 (C_7_), 50.8 (C_6_), 47.9 (C_13_), 44.7 (C_8_), 43.4 (C_10_), 36.0 (C_11–15_), 32.5 (C_16_), 29.4 (C_11–15_), 27.6 (C_17_), 24.4 (C_11–15_), 23.6 (C_11–15_), 21.7 (C_1_). Mp: 128.0–131.0 °C. IR: ν_max_ 2944, 2859, 1345, 1155 cm^–1^. HRMS: *m*/*z* calculated for C_21_H_33_ClNO_2_S^+^, 398.1915 [M + H]^+^. Found *m*/*z* 398.1925, Δ = 2.4 ppm. Rf (30% Et_2_O in Pet ether) = 0.72. Spectroscopic data consistent with those in the literature.^11^
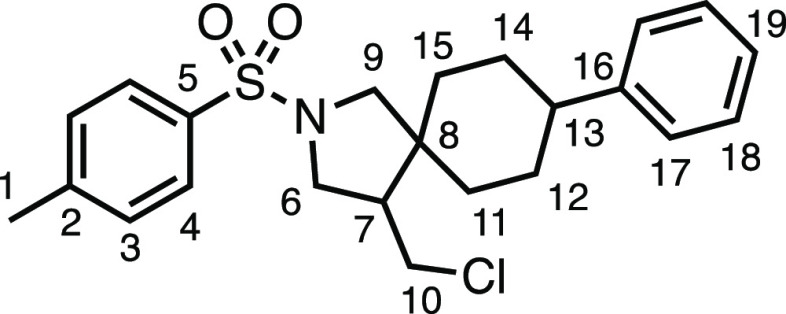


#### 4-(Chloromethyl)-8-phenyl-2-tosyl-2-azaspiro[4.5]decane (**3n**)

The compound was prepared according to General Procedure E1. Flash column chromatography (5–30% Et_2_O in Pet ether) afforded two separate diastereoisomers (3:2 d.r.) (total 60.0 mg, 72%). *Major diastereoisomer* (white solid, 35.0 mg): ^1^H NMR (400 MHz, CDCl_3_) δ 7.75 (app d, *J* = 8.1 Hz, 2H, H_4_), 7.35 (app d, *J* = 8.1 Hz, 2H, H_3_), 7.29 (t, *J* = 7.5 Hz, 2H, H_17_), 7.22–7.13 (m, 3H, H_18_ + H_19_), 3.65 (d, *J* = 11.0 Hz, 1H, H_6_), 3.55 (d, *J* = 10.9 Hz, 1H, H_6′_), 3.57–3.51 (m, 1H, H_10_), 3.12 (d, *J* = 9.8 Hz, 1H, H_9_), 3.01 (d, *J* = 9.8 Hz, 1H, H_9′_), 2.79 (t, *J* = 11.0 Hz, 1H, H_10′_), 2.55–2.37 (m, 5H, H_7_ + H_1_ + H_13_), 1.86–1.73 (m, 2H, H_11_ or H_12_ or H_14_ or H_15_), 1.69–1.19 (m, 6H, H_11_ or H_12_ or H_14_ or H_15_). ^13^C{^1^H} NMR (101 MHz, CDCl_3_) δ 146.0 (C_16_), 143.7 (C_5_), 133.7 (C_2_), 129.8 (C_3_), 128.6 (C_17_), 127.6 (C_4_), 126.8 (C_18_), 126.4 (C_19_), 59.1 (C_9_), 50.1 (C_6_), 44.6 (C_8_), 44.5 (C_7_), 44.0 (C_10_), 43.3 (C_13_), 36.3 (C_11_ or C_12_ or C_14_ or C_15_), 30.6 (C_11_ or C_12_ or C_14_ or C_15_), 30.1 (C_11_ or C_12_ or C_14_ or C_15_), 29.9 (C_11_ or C_12_ or C_14_ or C_15_), 21.7 (C_1_). Mp: 133.5–136.0 °C. IR: ν_max_ 2925, 2850, 1342, 1163 cm^–1^. HRMS: *m*/*z* calculated for C_23_H_29_ClNO_2_S^+^, 418.1602 [M + H]^+^. Found *m*/*z* 418.1608, Δ = 1.5 ppm. *Minor diastereoisomer* (white solid, 25.0 mg): ^1^H NMR (400 MHz, CDCl_3_) δ 7.76 (app d, *J* = 8.1 Hz, 2H, H_4_), 7.34 (app d, *J* = 8.1 Hz, 2H, H_3_), 7.30 (d, *J* = 7.5 Hz, 2H, H_17_), 7.21 (t, *J* = 7.5 Hz, 1H, H_19_), 7.17 (d, *J* = 7.5 Hz, 2H, H_18_), 3.64 (dd, *J* = 10.2, 7.7 Hz, 1H, H_6_), 3.58 (dd, *J* = 10.8, 4.2 Hz, 1H, H_10_), 3.50 (d, *J* = 10.1 Hz, 1H, H_9_), 3.26–3.15 (m, 3H, H_6′_ + H_9′_ + H_10′_), 2.43 (m, 4H, H_1_ + H_13_), 2.12 (dtd, *J* = 11.7, 7.8, 4.3 Hz, 1H, H_7_), 1.77 (d, *J* = 13.4 Hz, 2H, H_11_ or H_12_ or H_14_ or H_15_), 1.60–1.20 (m, 7H, H_11_ or H_12_ or H_14_ or H_15_). ^13^C{^1^H} NMR (101 MHz, CDCl_3_) δ 146.3 (C_16_), 143.7 (C_5_), 133.7 (C_2_), 129.9 (C_3_), 128.6 (C_17_), 127.6 (C_4_), 126.8 (C_18_), 126.4 (C_19_), 55.9 (C_9_), 51.0 (C_7_), 50.7 (C_6_), 44.4 (C_8_), 44.0 (C_13_), 43.2 (C_10_), 35.7 (C_11_ or C_12_ or C_14_ or C_15_), 31.0 (C_11_ or C_12_ or C_14_ or C_15_), 30.4 (C_11_ or C_12_ or C_14_ or C_15_), 29.2 (C_11_ or C_12_ or C_14_ or C_15_), 21.7 (C_1_). Mp: 146.0–149.0 °C. IR: ν_max_ 2929, 1340, 1163 cm^–1^. HRMS: *m*/*z* calculated for C_23_H_29_ClNO_2_S^+^, 418.1602 [M + H]^+^. Found *m*/*z* 418.1610, Δ = 1.9 ppm.
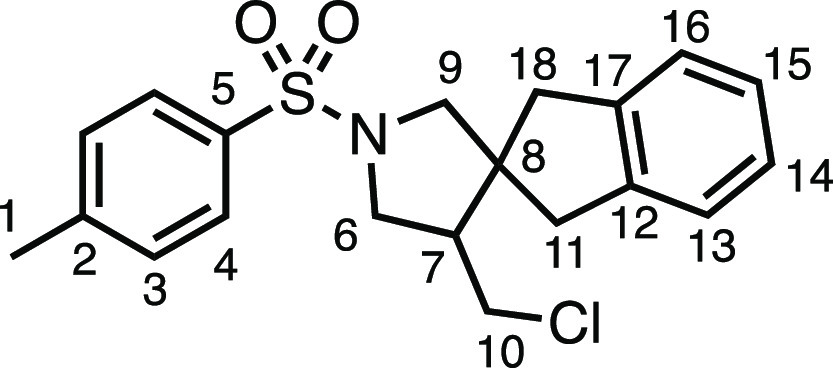


#### 4′-(Chloromethyl)-1′-tosyl-1,3-dihydrospiro[indene-2,3′-pyrrolidine] (**3o**)

The compound was prepared according to General Procedure E1. Flash column chromatography (10–20% EtOAc in Pet ether) afforded the product as a yellow oil (16.0 mg, 21%). ^1^H NMR (400 MHz, CDCl_3_) δ 7.73 (app d, *J* = 8.1 Hz, 2H, H_4_), 7.36 (app d, *J* = 8.1 Hz, 2H, H_3_), 7.17–7.05 (m, 4H, H_13–16_), 3.65 (dd, *J* = 10.5, 7.2 Hz, 1H, H_6_), 3.45–3.35 (m, 2H, H_6′_ + H_10_), 3.26 (d, *J* = 9.8 Hz, 1H, H_9_), 3.21 (d, *J* = 9.8 Hz, 1H, H_9′_), 3.08 (t, *J* = 10.9 Hz, 1H, H_10′_), 2.88 (d, *J* = 15.9 Hz, 1H, H_11_), 2.81 (d, *J* = 15.8 Hz, 2H, H_18_), 2.73 (d, *J* = 15.8 Hz, 1H, H_18′_), 2.56 (d, *J* = 15.9 Hz, 1H, H_11′_), 2.47 (s, 3H, H_1_), 2.36 (dtd, *J* = 10.8, 6.8, 4.3 Hz, 1H, H_7_). ^13^C{^1^H} NMR (101 MHz, CDCl_3_) δ 143.8 (C_5_), 141.0 (C_12_ or C_17_), 140.8 (C_12_ or C_17_), 133.9 (C_2_) 129.9 (C_3_), 127.6 (C_4_), 127.1 (C_13–16_), 127.1 (C_13–16_), 124.7 (C_13–16_), 124.6 (C_13–16_), 59.1 (C_9_), 53.0 (C_8_), 51.3 (C_6_), 49.4 (C_7_), 43.6 (C_10_), 43.0 (C_18_), 38.2 (C_11_), 21.7 (C_1_). IR: ν_max_ 1340, 1157 cm^–1^. HRMS: *m*/*z* calculated for C_20_H_23_ClNO_2_S^+^, 376.1133 [M + H]^+^. Found *m*/*z* 376.1137, Δ = 1.1 ppm. Rf (30% EtOAc in Pet ether) = 0.49.
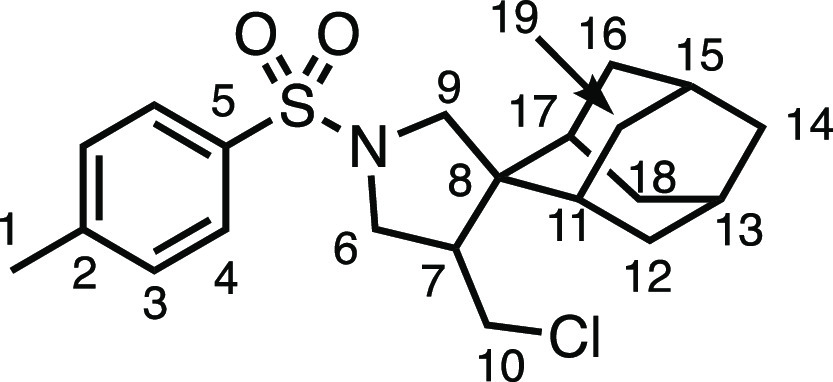


#### (1*R*,3*S*,5*r*,7*r*)-4′-(Chloromethyl)-1′-tosylspiro[adamantane-2,3′-pyrrolidine] (**3p**)

The compound was prepared according to General Procedure E1. Flash column chromatography (5–10% EtOAc in Pet ether) afforded the product as an off-white solid (45.5 mg, 58%). ^1^H NMR (400 MHz, CDCl_3_) δ 7.74 (app d, *J* = 8.0 Hz, 2H, H_4_), 7.32 (app d, *J* = 8.0 Hz, 2H, H_3_), 3.76 (d, *J* = 10.6 Hz, 1H, H_9_), 3.68 (d, *J* = 10.8 Hz, 1H, H_6_), 3.56–3.44 (m, 1H, H_10_), 3.30 (dd, *J* = 10.7, 5.5 Hz, 1H, H_6′_), 2.78 (d, *H* = 10.6 Hz, 1H, H_9′_), 2.75 (app t, *J* = 11.2 Hz,1H, H_10′_), 2.55 (dt, *J* = 11.7, 4.2 Hz, 1H, H_7_), 2.43 (s, 3H, H_1_), 1.93–1.42 (m, 14H, H_11–19_). ^13^C{^1^H} NMR (101 MHz, CDCl_3_) δ 143.6 (C_5_), 133.9 (C_2_), 129.7 (C_3_), 127.5 (C_4_), 54.2 (C_9_), 50.8 (C_8_), 49.0 (C_6_), 45.9 (C_7_), 43.7 (C_10_), 38.0 (C_11–19_), 34.5 (C_11–19_), 34.0 (C_11–19_), 33.6 (C_11–19_), 33.5 (C_11–19_), 32.0 (C_11–19_), 31.8 (C_11–19_), 27.1 (C_11–19_), 27.0 (C_11–19_), 21.7 (C_1_). Mp: 118.3–120.2 °C. IR: ν_max_ 2897, 1344, 1163 cm^–1^. HRMS: *m*/*z* calculated for C_21_H_29_ClNO_2_S^+^, 394.1602 [M + H]^+^. Found *m*/*z* 394.1608, Δ = 1.5 ppm. Rf (10% EtOAc in Pet ether) = 0.33.
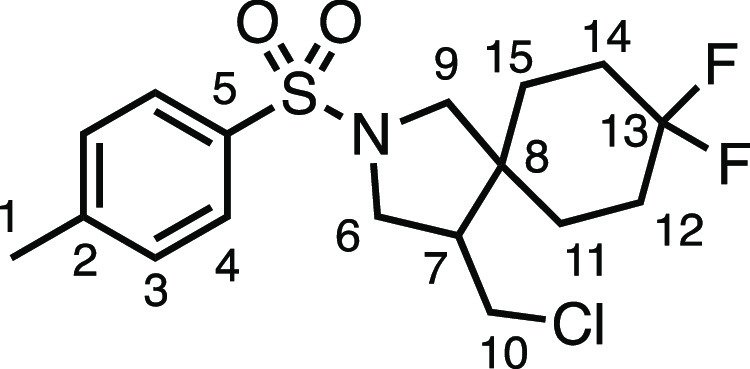


#### 4-(Chloromethyl)-8,8-difluoro-2-tosyl-2-azaspiro[4.5]decane (**3q**)

The compound was prepared according to General Procedure E1. Flash column chromatography (10% EtOAc in Pet ether) afforded the product as a white solid (48.9 mg, 65%). ^1^H NMR (400 MHz, CDCl_3_) δ 7.73 (app d, *J* = 8.0 Hz, 2H, H_4_), 7.35 (app d, *J* = 8.0 Hz, 2H, H_3_), 3.61 (dd, *J* = 10.4, 7.7 Hz, 1H, H_6_), 3.49 (dd, *J* = 10.9, 4.2 Hz, 1H, H_10_), 3.35 (d, *J* = 10.1 Hz, 1H, H_9_), 3.26 (dd, *J* = 10.4, 7.0 Hz, 1H, H_6′_), 3.15 (t, *J* = 10.6 Hz, 1H, H_10′_), 3.09 (d, *J* = 10.1 Hz, 1H, H_9_), 2.44 (s, 3H, H_1_), 2.22–2.12 (m, 1H, H_7_), 1.95 (dd, *J* = 15.2, 6.2 Hz, 2H, H_12_ + H_14_), 1.61–1.78 (m, 3H, H_12′_ + H_14′_ + H_11_ or H_15_), 1.48–1.30 (m, 3H, H_11_ + H_15_). ^13^C{^1^H} NMR (101 MHz, CDCl_3_) δ 144.0 (C_5_), 133.6 (C_2_), 130.0 (C_3_), 127.5 (C_4_), 122.4 (dd, *J* = 239.7, 240.1 Hz, C_13_), 55.4 (d, *J* = 1.7 Hz, C_9_), 50.5 (C_6_), 49.2 (d, *J* = 1.8 Hz, C_7_), 43.6 (d, *J* = 1.1 Hz, C_8_), 42.9 (C_10_), 31.6 (dd, *J* = 8.6, 1.2 Hz, C_11_ or C_15_), 31.18 (dd, *J* = 25.5, 23.7 Hz, C_12_ or C_14_), 30.67 (dd, *J* = 25.5, 24.0 Hz, C_12_ or C_14_), 25.2 (d, *J* = 9.9 Hz, C_11_ or C_15_), 21.7 (C_1_). ^19^F NMR (376 MHz, CDCl_3_) δ −94.1 (d, *J* = 237.5 Hz), −103.5 (d, *J* = 236.6 Hz). Mp: 101.0–103.0 °C. IR: ν_max_ 2963, 2941, 2920, 2861, 1338, 1163 cm^–1^. HRMS: *m*/*z* calculated for C_17_H_23_ClF_2_NO_2_S^+^, 378.1101 [M + H]^+^. Found *m*/*z* 378.1101, Δ = 0.1 ppm. Rf (20% EtOAc in Pet ether) = 0.31.
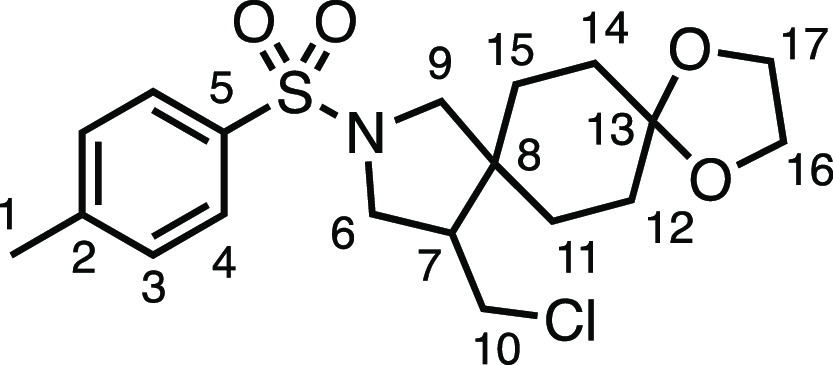


#### 12-(Chloromethyl)-10-tosyl-1,4-dioxa-10-azadispiro[4.2.4^8^.2^5^]tetradecane (**3r**)

The compound was prepared according to General Procedure E1. Flash column chromatography (10–20% EtOAc in Pet ether, then 5% MeCN in toluene) afforded the product as a white solid (54.2 mg, 68%). ^1^H NMR (400 MHz, CDCl_3_) δ 7.72 (app d, *J* = 8.1 Hz, 2H, H_4_), 7.33 (app d, *J* = 8.1 Hz, 2H, H_3_), 3.89 (m, 4H, H_16_ + H_17_), 3.60 (dd, *J* = 10.3, 7.4 Hz, 1H, H_6_), 3.52 (dd, *J* = 10.8, 4.0 Hz, 1H, H_10_), 3.35 (d, *J* = 10.2 Hz, 1H, H_9_), 3.24 (dd, *J* = 10.3, 6.9 Hz, 1H, H_6′_), 3.14 (d, *J* = 10.8 Hz, 1H, H_9′_), 3.10 (app t, *J* = 10.3 Hz, 1H, H_10′_), 2.43 (s, 3H, H_1_), 2.14 (dtd, *J* = 11.0, 7.1, 4.1 Hz, 1H, H_7_), 1.69–1.27 (m, 8H, H_11_ + H_12_ + H_14_ + H_15_). ^13^C{^1^H} NMR (126 MHz, CDCl_3_) δ 143.8 (C_5_), 133.7 (C_2_), 129.9 (C_3_), 127.5 (C_4_), 107.9 (C_13_), 64.5 (C_16_ or C_17_), 64.4 (C_16_ or C_17_), 55.8 (C_9_), 50.7 (C_6_), 49.5 (C_7_), 44.0 (C_8_), 43.3 (C_10_), 32.8 (C_11_ or C_12_ or C_14_ or C_15_), 32.1 (C_11_ or C_12_ or C_14_ or C_15_), 31.6 (C_11_ or C_12_ or C_14_ or C_15_), 26.2 (C_11_ or C_12_ or C_14_ or C_15_), 21.7 (C_1_). Mp: 110.7–113.0 °C. IR: ν_max_ 2926, 1338, 1161 cm^–1^. HRMS: *m*/*z* calculated for C_19_H_27_ClNO_4_S^+^, 400.1344 [M + H]^+^. Found *m*/*z* 400.1352, Δ = 2.0 ppm. Rf (20% EtOAc in Pet ether) = 0.08.
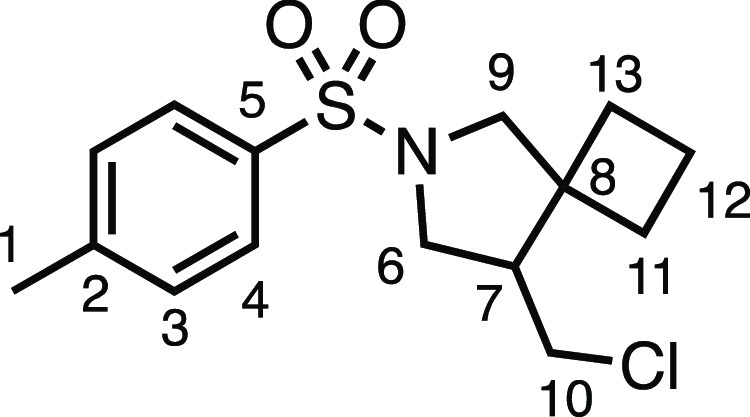


#### 8-(Chloromethyl)-6-tosyl-6-azaspiro[3.4]octane (**3s**)

The compound was prepared according to General Procedure E1. Flash column chromatography (5–30% Et_2_O in Pet ether) afforded the product as a colorless oil (48.0 mg, 77%). ^1^H NMR (400 MHz, CDCl_3_) δ 7.71 (app d, *J* = 8.0 Hz, 2H, H_4_), 7.32 (app d, *J* = 8.0 Hz, 2H, H_3_), 3.48 (dd, *J* = 10.9, 4.2 Hz, 1H, H_10_), 3.40 (dd, *J* = 10.5, 6.6 Hz, 1H, H_6_), 3.34 (d, *J* = 9.8 Hz, 1H, H_9_), 3.28 (dd, *J* = 10.4, 4.4 Hz, 1H, H_6′_), 3.20 (d, *J* = 9.8 Hz, 1H, H_9_), 2.93 (t, *J* = 10.7 Hz, 1H, H_10′_), 2.42 (s, 3H, H_1_), 2.18 (dq, *J* = 10.4, 4.4 Hz, 1H, H_7_), 2.05–1.94 (m, 1H, H_11_ or H_13_), 1.91–1.59 (m, 5H, H_11_ + H_12_ + H_13_). ^13^C{^1^H} NMR (101 MHz, CDCl_3_) δ 143.7 (C_5_), 133.7 (C_2_), 129.8 (C_3_), 127.6 (C_4_), 58.2 (C_9_), 50.1 (C_6_), 49.4 (C_7_), 47.5 (C_8_), 43.3 (C_10_), 32.6 (C_11_ or C_13_), 26.0 (C_11_ or C_13_), 21.6 (C_1_), 16.3 (C_12_). IR: ν_max_ 2929, 1341, 1155 cm^–1^. HRMS: *m*/*z* calculated for C_15_H_21_ClNO_2_S^+^, 314.0976 [M + H]^+^. Found *m*/*z* 314.0975, Δ = −0.3 ppm. Rf (30% Et_2_O in Pet ether) = 0.25.
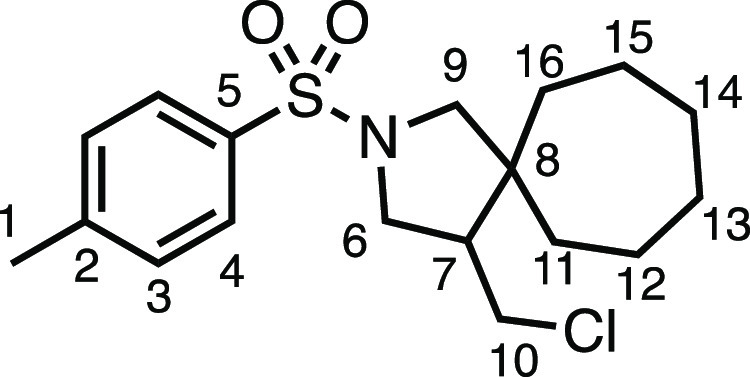


#### 4-(Chloromethyl)-2-tosyl-2-azaspiro[4.6]undecane (**3t**)

The compound was prepared according to General Procedure E1. Flash column chromatography (10% EtOAc in Pet ether) afforded the product as a colorless oil (40.7 mg, 57%). ^1^H NMR (400 MHz, CDCl_3_) δ 7.71 (app d, *J* = 8.1 Hz, 2H, H_4_), 7.32 (app d, *J* = 8.1 Hz, 2H, H_3_), 3.57 (app t, *J* = 10.1 Hz, 1H, H_6_), 3.55 (dd, *J* = 11.6, 5.1 Hz, 1H, H_10_), 3.25 (d, *J* = 9.7 Hz, 1H, H_9_), 3.20 (dd, *J* = 10.4, 7.6 Hz, 1H, H_6′_), 3.13 (t, *J* = 10.9 Hz, 1H, H_10′_), 2.97 (d, *J* = 9.7 Hz, 1H, H_9′_), 2.43 (s, 3H, H_1_), 2.08 (dtd, *J* = 11.5, 7.6, 4.2 Hz, 1H, H_7_), 1.59–1.17 (m, 12H, H_11–16_). ^13^C{^1^H} NMR (101 MHz, CDCl_3_) δ 143.6 (C_5_), 134.0 (C_2_), 130.0 (C_3_), 127.5 (C_4_), 59.3 (C_9_), 51.7 (C_7_), 51.1 (C_6_), 47.8 (C_8_), 43.6 (C_10_), 38.6 (C_11–16_), 31.8 (C_11–16_), 29.6 (C_11–16_), 29.5 (C_11–16_), 23.9 (C_11–16_), 23.6 (C_11–16_), 21.7 (C_1_). IR: ν_max_ 2922, 2854, 1341, 1158 cm^–1^. HRMS: *m*/*z* calculated for C_18_H_27_ClNO_2_S^+^, 356.1445 [M + H]^+^. Found *m*/*z* 356.1445, Δ = −0.1 ppm. Rf (20% EtOAc in Pet ether) = 0.43.
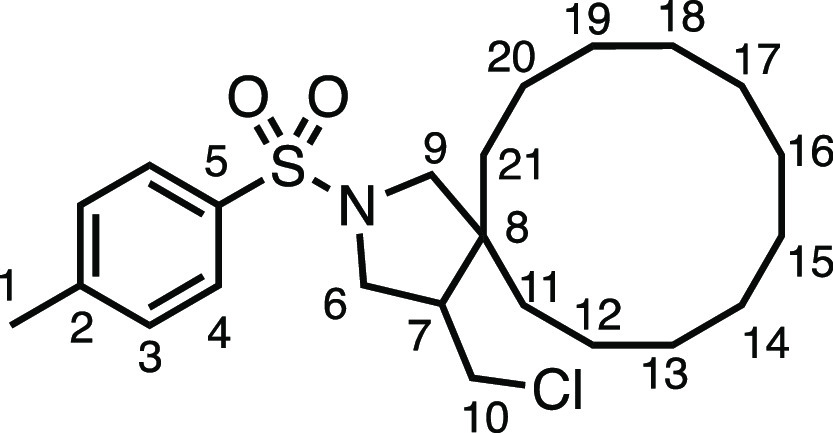


#### 4-(Chloromethyl)-2-tosyl-2-azaspiro[4.11]hexadecane (**3u**)

The compound was prepared according to General Procedure E1. Flash column chromatography (5–10% EtOAc in Pet ether) afforded inseparable diastereomers of the product as a colorless oil (44.9 mg, 53%). ^1^H NMR (400 MHz, CDCl_3_) δ 7.72 (app d, *J* = 8.1 Hz, 2H, H_4_), 7.32 (app d, *J* = 8.1 Hz, 2H, H_3_), 3.57 (dd, *J* = 10.7, 6.0 Hz, 1H, H_6_), 3.55 (app t, *J* = 10.1 Hz, 1H, H_10_), 3.36 (dd, *J* = 10.7, 4.8 Hz, 1H, H_6′_), 3.07–2.92 (m, 3H, H_9_ + H_9′_ + H_10′_), 2.43 (s, 3H, H_1_), 2.16 (ddt, *J* = 11.3, 8.3, 4.4 Hz, 1H, H_7_), 1.38–1.10 (m, 22H, H_11–21_). ^13^C{^1^H} NMR (101 MHz, CDCl_3_) δ 143.6 (C_2_), 133.7 (C_5_), 129.8 (C_3_), 127.6 (C_4_), 57.4 (C_9_), 51.2 (C_6_), 48.9 (C_7_), 47.5 (C_8_), 44.7 (C_10_), 32.3 (C_11–21_), 27.1 (C_11–21_), 26.8 (C_11–21_), 26.6 (C_11–21_), 26.1 (C_11–21_), 22.8 (C_11–21_), 22.7 (C_11–21_), 22.3 (C_11–21_), 22.2 (C_11–21_), 21.7 (C_1_), 19.6 (C_11–21_). IR: ν_max_ 2930, 2859, 1345, 1158 cm^–1^. HRMS: *m*/*z* calculated for C_23_H_37_ClNO_2_S^+^, 426.2228 [M + H]^+^. Found *m*/*z* 426.2239, Δ = 2.6 ppm. Rf (20% EtOAc in Pet ether) = 0.56.
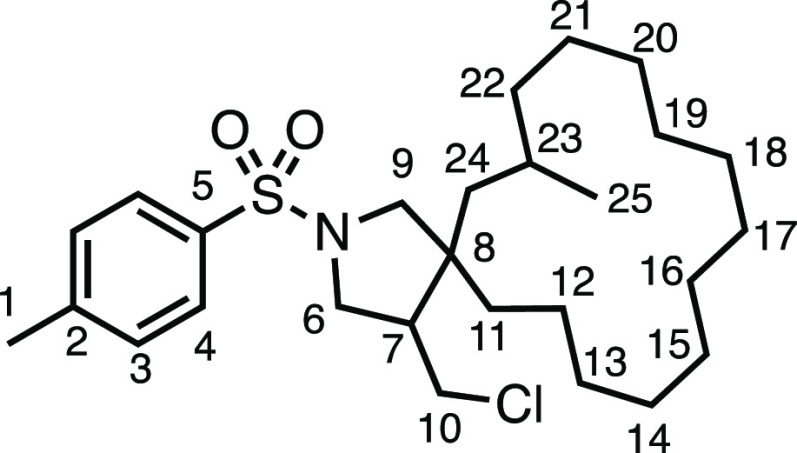


#### 4-(Chloromethyl)-7-methyl-2-tosyl-2-azaspiro[4.14]nonadecane (**3v**)

The compound was prepared according to General Procedure E1. Flash column chromatography (5–10% EtOAc in Pet ether) afforded an inseparable mixture of diastereomers as a colorless oil (52.2 mg, 54%). ^1^H NMR (400 MHz, CDCl_3_) δ 7.72 (app d, *J* = 7.7 Hz, 2H, H_4_), 7.33 (app d, *J* = 7.7 Hz, 2H, H_3_), 3.66–3.41 (m, 2H, H_6_ + H_10_), 3.33 (app dq, *J* = 11.0, 6.3, 5.8 Hz, 1H, H_6′_), 3.25–2.71 (m, 3H, H_9_ + H_9′_ + H_10′_), 2.43 (s, 3H, H_1_), 2.26–1.95 (m, 1H, H_7_), 1.45–0.91 (m, 27H, H_11–24_), 0.83 (app t, *J* = 6.9 Hz, 3H, H_25_). ^13^C{^1^H} NMR (101 MHz, CDCl_3_) δ 143.7, 143.6, 143.6, 134.0, 133.7, 133.6, 129.8, 129.8, 127.7, 127.6, 127.6, 59.1, 57.8, 56.9, 51.4, 51.2, 50.5, 50.2, 48.6, 48.2, 47.8, 47.5, 44.7, 44.4, 44.3, 43.8, 39.7, 37.9, 37.4, 37.3, 37.0, 36.0, 31.9, 28.5, 28.2, 28.0, 28.0, 27.8, 27.7, 27.6, 27.2, 27.1, 27.00, 27.0, 26.9, 26.9, 26.8, 26.8, 26.8, 26.3, 26.1, 25.9, 25.9, 25.7, 25.6, 25.6, 25.6, 25.5, 25.5, 25.1, 24.8, 24.7, 23.2, 23.0, 22.8, 22.6, 22.5, 22.1, 21.7, 21.5, 21.2. IR: ν_max_ 2925, 2855, 1345, 1160 cm^–1^. HRMS: *m*/*z* calculated for C_27_H_45_ClNO_2_S^+^, 482.2866 [M + H]^+^. Found *m*/*z* 482.2860, Δ = 1.2 ppm. Rf (20% EtOAc in Pet ether) = 0.56.
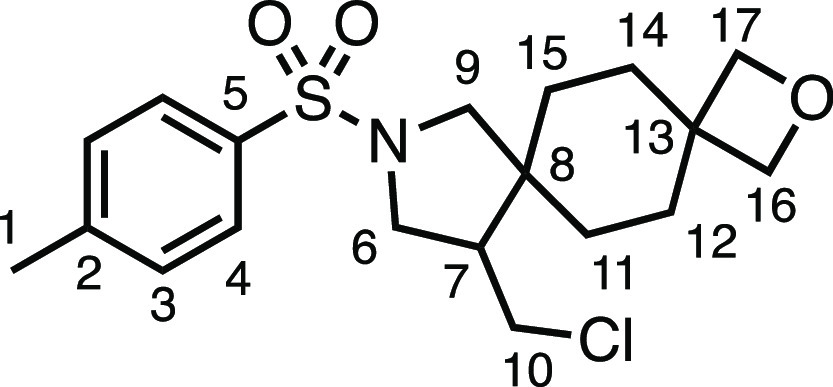


#### 11-(Chloromethyl)-9-tosyl-2-oxa-9-azadispiro[3.2.4^7^.2^4^]tridecane (**3w**)

The compound was prepared according to General Procedure E1. Flash column chromatography (20–40% EtOAc in Pet ether) afforded the product as a colorless oil (41.9 mg, 55%). ^1^H NMR (500 MHz, CDCl_3_) δ 7.71 (app d, *J* = 8.1 Hz, 2H, H_4_), 7.33 (app d, *J* = 8.1 Hz, 2H, H_3_), 4.38–4.21 (m, 4H, H_16_ + H_17_), 3.55 (dd, *J* = 10.4, 7.5 Hz, 1H, H_6_), 3.44 (dd, *J* = 10.9, 4.4 Hz, 1H, H_10_), 3.26 (d, *J* = 10.2 Hz, 1H, H_9_), 3.23 (dd, *J* = 10.5, 6.8 Hz, 1H, H_6′_), 3.0899 (app t, *J* = 10.7 Hz, 1H, H_10′_), 3.04 (d, *J* = 10.2 Hz, 1H, H_9′_), 2.44 (s, 3H, H_1_), 2.10–2.03 (m, 1H, H_7_), 1.91 (t, *J* = 11.9 Hz, 2H, H_12_ or H_14_), 1.51–1.39 (m, 2H, H_11–15_), 1.33 (td, *J* = 12.8, 12.2, 3.6 Hz, 1H, H_11–15_), 1.24–1.05 (m, 3H, H_11–15_). ^13^C{^1^H} NMR (126 MHz, CDCl_3_) δ 143.8 (C_2_), 133.6 (C_5_), 129.8 (C_3_), 127.5 (C_4_), 82.0 (C_16_ or C_17_), 81.4 (C_16_ or C_17_), 55.6 (C_9_), 50.4 (C_6_), 49.5 (C_7_), 43.9 (C_8_), 43.1 (C_10_), 39.7 (C_13_), 32.0 (C_11_ or C_12_ or C_14_ or C_15_), 32.0 (C_11_ or C_12_ or C_14_ or C_15_), 31.6 (C_11_ or C_12_ or C_14_ or C_15_), 25.5 (C_11_ or C_12_ or C_14_ or C_15_), 21.7 (C_1_). IR: ν_max_ 2922, 2858, 1339, 1157 cm^–1^. HRMS: *m*/*z* calculated for C_19_H_27_ClNO_3_S^+^, 384.1395 [M + H]^+^. Found *m*/*z* 384.1399, Δ = 1.1 ppm. Rf (40% EtOAc in Pet ether) = 0.13.
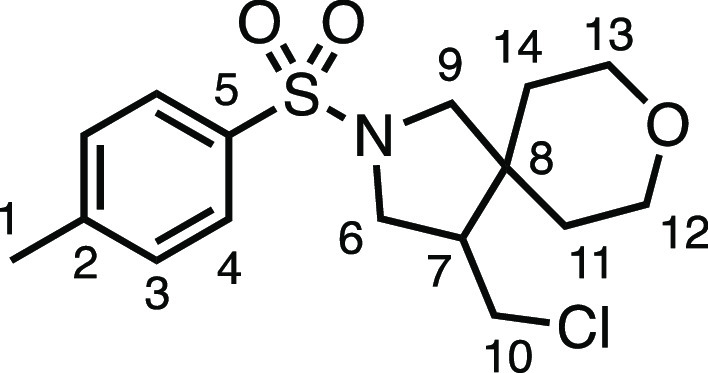


#### 4-(Chloromethyl)-2-tosyl-8-oxa-2-azaspiro[4.5]decane (**3x**)

The compound was prepared according to General Procedure E1. Flash column chromatography (20–40% EtOAc in Pet ether) afforded the product as a colorless oil (31.5 mg, 46%). ^1^H NMR (400 MHz, CDCl_3_) δ 7.72 (app d, *J* = 8.1 Hz, 2H, H_4_), 7.33 (app d, *J* = 8.1 Hz, 2H, H_3_), 3.79–3.68 (m, 2H, H_12_ + H_13_), 3.57 (dd, *J* = 10.5, 7.4 Hz, 1H, H_6_), 3.52 (dd, *J* = 10.9, 4.2 Hz, 1H, H_10_), 3.43–3.32 (m, 3H, H_9_ + H_12′_ + H_13′_), 3.26 (dd, *J* = 10.5, 6.6 Hz, 1H, H_6′_), 3.19 (d, *J* = 10.2 Hz, 1H, H_9′_), 3.12 (t, *J* = 10.7 Hz, 1H, H_10′_), 2.43 (s, 3H, H_1_), 2.18–2.04 (m, 1H, H_7_), 1.66 (ddd, *J* = 13.5, 11.5, 4.6 Hz, 1H, H_11–14_), 1.48 (ddd, *J* = 13.1, 11.6, 4.5 Hz, 1H, H_11–14_), 1.19 (dq, *J* = 13.5, 2.4 Hz, 1H, H_11–14_), 1.15–1.07 (m, 1H, H_11–14_). ^13^C{^1^H} NMR (101 MHz, CDCl_3_) δ 143.9, (C_2_), 133.6 (C_5_), 129.9 (C_3_), 127.5 (C_4_), 65.0 (C_12_ or C_13_), 64.6 (C_12_ or C_13_), 55.4 (C_9_), 50.2 (C_6_), 50.1 (C_7_), 42.9 (C_8_), 42.7 (C_10)_, 35.3 (C_11_ or C_14_), 29.6 (C_11_ or C_14_), 21.7 (C_1_). IR: ν_max_ 2923, 2851, 1339, 1156 cm^–1^. HRMS: *m*/*z* calculated for C_16_H_23_ClNO_3_S^+^, 344.1082 [M + H]^+^. Found *m*/*z* 344.1076, Δ = −1.7 ppm. Rf (40% EtOAc in Pet ether) = 0.22.
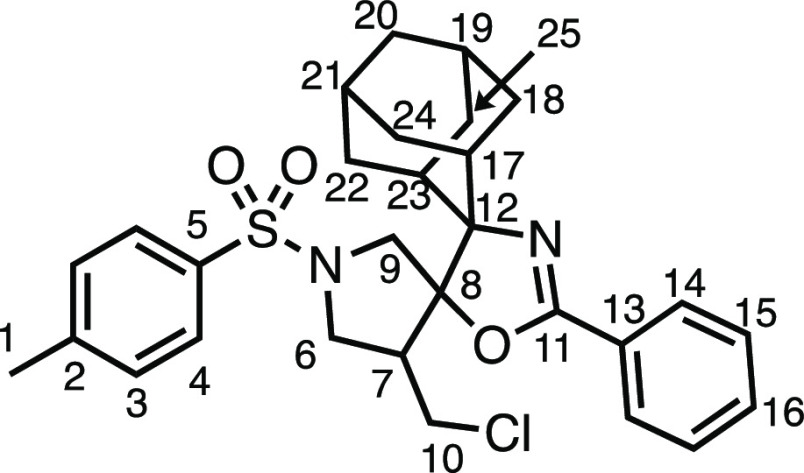


#### (5*r*,7*r*)-4″-(Chloromethyl)-2′-phenyl-1″-tosyldispiro[adamantane-2,4′-oxazole-5′,3″-pyrrolidine] (**3y**)

The compound was prepared according to General Procedure E1. Flash column chromatography (10–30% Et_2_O in Pet ether) afforded the product as an off-white solid (36.8 mg, 39%). ^1^H NMR (400 MHz, CDCl_3_) δ 7.78 (d, *J* = 7.7 Hz, 2H, H_4_), 7.52 (d, *J* = 7.7 Hz, 2H, H_14_), 7.46 (t, *J* = 7.4 Hz, 1H, H_16_), 7.32 (app q, *J* = 7.6 Hz, 4H, H_3_ + H_15_), 4.13 (d, *J* = 11.8 Hz, 1H, H_9_), 3.98 (dd, *J* = 9.5, 8.0 Hz, 1H, H_6_), 3.77 (d, *J* = 11.8 Hz, 1H, H_9′_), 3.74 (dd, *J* = 11.1, 3.4 Hz, 1H, H_10_), 3.20 (t, *J* = 10.0 Hz, 1H, H_6_), 3.13 (t, *J* = 11.1 Hz, 1H, H_10_), 2.81 (tdd, *J* = 11.0, 8.0, 3.3, 1H, H_7_), 2.68 (d, *J* = 12.2 Hz, 1H, H_17_ or H_23_), 2.55 (d, *J* = 12.6 Hz, 1H, H_17_ or H_23_), 2.46 (s, 3H, H_1_), 1.96–1.46 (m, 12H, H_18–22_ + H_24–25_). ^13^C{^1^H} NMR (126 MHz, CDCl_3_) δ 158.2 (C_11_), 143.7 (C_5_), 134.1 (C_2_), 131.7 (C_16_), 129.9 (C_3_), 128.4 (C_15_), 128.2 (C_14_), 127.9 (C_4_), 127.1 (C_13_), 95.4 (C_8_), 75.4 (C_12_), 56.5 (C_9_), 51.4 (C_6_), 47.4 (C_7_), 42.6 (C_10_), 38.4 (C_18–22_ or C_24–25_), 36.2 (C_18–22_ or C_24–25_), 34.7 (C_17_ or C_23_), 34.6 (C_18–25_), 34.6 (C_18–25_), 33.7 (C_18–22_ or C_24–25_), 33.6 (C_18–22_ or C_24–25_), 30.5 (C_18–22_ or C_24–25_), 27.6 (C_18–22_ or C_24–25_), 26.7 (C_18–22_ or C_24–25_), 21.8 (C_1_). Mp: 86.7–90.0 °C. IR: ν_max_ 2908, 1653, 1335, 1162 cm^–1^. HRMS: *m*/*z* calculated for C_29_H_34_ClN_2_O_3_S^+^, 525.1973 [M + H]^+^. Found *m*/*z* 525.1978, Δ = 1.0 ppm.
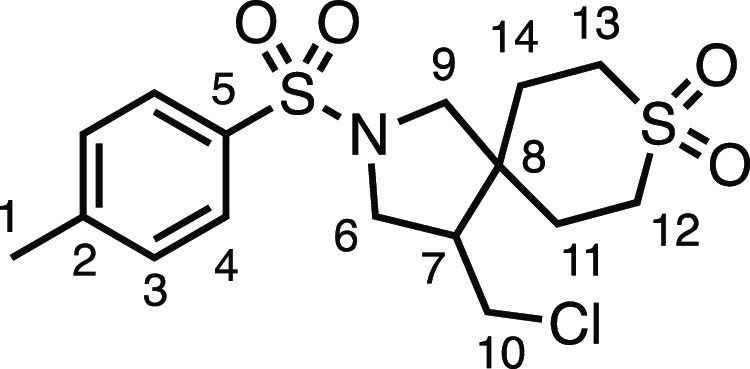


#### 4-(Chloromethyl)-2-tosyl-8-thia-2-azaspiro[4.5]decane 8,8-Dioxide (**3z**)

The compound was prepared according to General Procedure E1. Flash column chromatography (20–30% EtOAc in Pet ether) followed by vapor diffusion recrystallization from DCM (Pet ether antisolvent) afforded the product as off-white crystals (43.6 mg, 56%). ^1^H NMR (400 MHz, CDCl_3_) δ 7.73 (app d, *J* = 8.1 Hz, 2H, H_4_), 7.36 (app d, *J* = 8.1 Hz, 2H, H_3_), 3.64 (dd, *J* = 10.6, 8.0 Hz, 1H, H_6_), 3.52 (dd, *J* = 11.2, 4.7 Hz, 1H, H_10_), 3.49 (d, *J* = 10.1 Hz, 1H, H_9_), 3.30–3.17 (m, 2H, H_6′_ + H_10′_), 3.12 (d, *J* = 10.3 Hz, 1H, H_9′_), 3.00–2.85 (m, 4H, H_12_ + H_13_), 2.45 (s, 3H, H_1_), 2.26 (dtt, *J* = 12.7, 8.2, 4.4 Hz, 2H, H_7_ + H_11_ or H_14_), 1.99 (td, *J* = 12.8, 10.8, 5.1 Hz, 1H, H_11_ or H_14_), 1.81–1.67 (m, 2H, H_11_ or H_14_). ^13^C{^1^H} NMR (101 MHz, CDCl_3_) δ 144.4 (C_5_), 133.4 (C_2_), 130.1 (C_3_), 127.5 (C_4_), 54.8 (C_9_), 50.4 (C_6_), 49.2 (C_7_), 48.7 (C_12_ or C_13_), 47.9 (C_12_ or C_13_), 43.0 (C_8_), 42.2 (C_10_), 33.0 (C_11_ or C_14_), 26.6 (C_11_ or C_14_), 21.7 (C_1_). Mp: 176.0–178.0 °C. IR: ν_max_ 2916, 2854, 1340, 1161 cm^–1^. HRMS: *m*/*z* calculated for C_16_H_23_ClNO_4_S_2_^+^, 392.0751 [M + H]^+^. Found *m*/*z* 392.0748, Δ = −0.8 ppm.
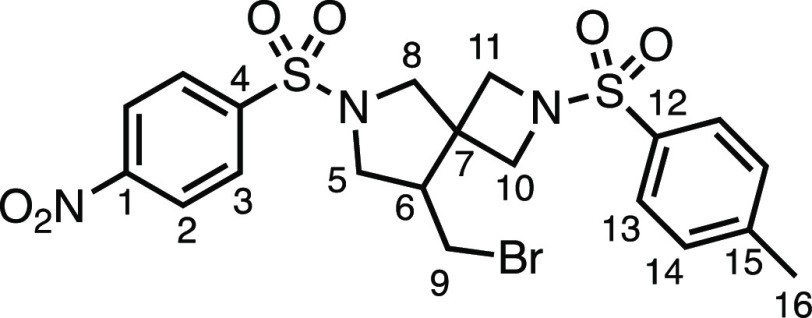


#### 8-(Bromomethyl)-6-((4-nitrophenyl)sulfonyl)-2-tosyl-2,6-diazaspiro[3.4]octane (**3aa**)

The compound was prepared according to General Procedure E3. Flash column chromatography (DCM) followed by recrystallization by vapor diffusion from DCM (Pet ether as the antisolvent) afforded the product as a white crystalline solid (57.8 mg, 53%). ^1^H NMR (400 MHz, CDCl_3_) δ 8.39 (app d, *J* = 8.8 Hz, 2H, H_2_), 7.97 (app d, *J* = 8.8 Hz, 2H, H_3_), 7.69 (app d, *J* = 8.2 Hz, 2H, H_13_), 7.40 (app d, *J* = 8.1 Hz, 2H, H_14_), 3.74 (d, *J* = 8.8 Hz, 1H, H_8_), 3.60 (d, *J* = 8.3 Hz, 1H, H_10_), 3.60 (d, *J* = 8.4 Hz, 1H, H_10′_), 3.53 (dd, *J* = 10.7, 7.4 Hz, 1H, H_5_), 3.45 (d, *J* = 8.8 Hz, 1H, H_8′_), 3.34 (s, 2H, H_11_), 3.18 (dd, *J* = 10.6, 8.1 Hz, 1H, H_9_), 3.17 (t, *J* = 10.5 Hz, 1H, H_5′_), 2.87 (t, *J* = 10.1 Hz, 1H, H_9′_), 2.48 (s, 3H, H_16_), 2.37 (qt, *J* = 6.9, 4.2 Hz, 1H, H_6_). ^13^C{^1^H} NMR (101 MHz, CDCl_3_) δ 150.5 (C_4_), 145.1 (C_12_), 142.3 (C_1_), 131.0 (C_15_), 130.2 (C_14_), 128.7 (C_3_), 128.5 (C_13_), 124.7 (C_2_), 59.1 (C_10_ or C_11_), 57.0 (C_10_ or C_11_), 54.3 (C_8_), 51.3 (C_5_), 46.7 (C_6_), 42.4 (C_7_), 29.5 (C_9_), 21.8 (C_16_). Mp: 189.8–192.4 °C. IR: ν_max_ 2887, 1527, 1346, 1333, 1164, 1150 cm^–1^. HRMS: *m*/*z* calculated for C_20_H_23_BrN_3_O_6_S_2_^+^, 544.0206 [M + H]^+^. Found *m*/*z* 544.0215, Δ = 1.7 ppm. Rf (DCM) = 0.10.
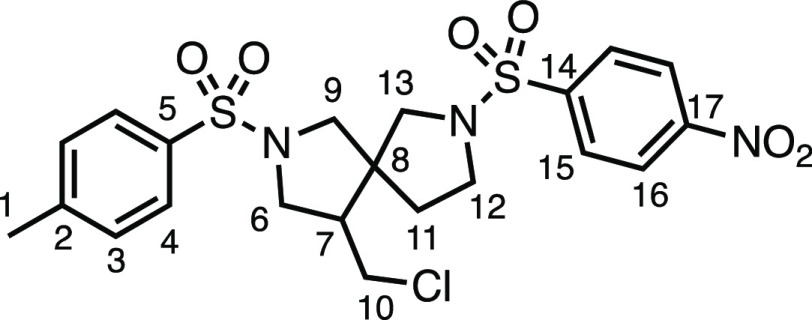


#### 4-(Chloromethyl)-7-((4-nitrophenyl)sulfonyl)-2-tosyl-2,7-diazaspiro[4.4]nonane (**3ab**)

The compound was prepared according to General Procedure E1. Flash column chromatography (30% EtOAc in Pet ether) afforded the product as an inseparable 1:1 mixture of diastereomers (yellow oil, 50.2 mg, 49%). ^1^H NMR (400 MHz, CDCl_3_) δ 8.38 (app d, *J* = 8.6 Hz, 2H, H_16_), 7.97 (app d, *J* = 8.6 Hz, 2H, H_15_), 7.72–7.57 (m, 2H, H_4_), 7.34 (app d, *J* = 7.9 Hz, 2H, H_3_), 3.55–3.32 (m, 3H, H_6–13_), 3.30–2.84 (m, 7H, H_6–13_), 2.44 (s, 3H, H_1_), 2.25 (tt, *J* = 8.8, 4.4 Hz, 1H, H_7_), 1.87–1.58 (m, 2H, H_11_). ^13^C{^1^H} NMR (101 MHz, CDCl_3_) δ 150.4, 150.4, 144.4, 144.3, 142.4, 142.2, 133.0, 132.9, 130.1, 130.1, 128.7, 128.6, 127.6, 124.7, 124.7, 57.0 (C_12_), 56.5 (C_13_), 56.3 (C_13″_), 51.9 (C_12″_), 51.5 (C_8_), 51.2 (C_8″_), 50.9 (C_6_), 50.8 (C_6″_), 47.6 (C_7_), 46.9 (C_6_), 46.1 (C_6″_), 45.9 (C_7″_), 43.1 (C_10_), 42.8 (C_10″_), 35.0 (C_11_), 29.8 (C_11″_), 21.7 (C_1_ + C_1″_). IR: ν_max_ 2922, 1528, 1346, 1159 cm^–1^. HRMS: *m*/*z* calculated for C_21_H_25_ClN_3_O_6_S_2_^+^, 514.0868 [M + H]^+^. Found *m*/*z* 514.0842, Δ = −5.0 ppm. Rf (30% EtOAc in Pet ether) = 0.29.
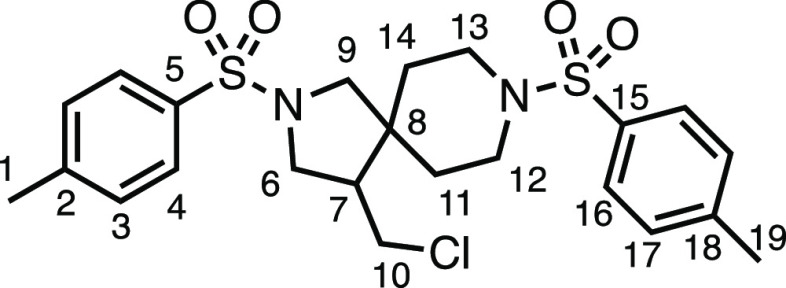


#### 4-(Chloromethyl)-2,8-ditosyl-2,8-diazaspiro[4.5]decane (**3ac**)

The compound was prepared according to General Procedure E1. Flash column chromatography (20–30% EtOAc in Pet ether) afforded the product as a white solid (75.5 mg, 76%). ^1^H NMR (400 MHz, CDCl_3_) δ 7.64 (app d, *J* = 8.1 Hz, 2H, H_4_ or H_16_), 7.60 (app d, *J* = 8.2 Hz, 2H, H_4_ or H_16_), 7.35 (app d, *J* = 8.1 Hz, 2H, H_3_ or H_17_), 7.30 (app d, *J* = 8.1 Hz, 2H, H_3_ or H_17_), 3.53 (app dt, *J* = 10.4, 5.3 Hz, 3H, H_6_ + H_12_ + H_13_), 3.44 (dd, *J* = 10.8, 4.2 Hz, 1H, H_10_), 3.18 (dd, *J* = 10.6, 7.1 Hz, 1H, H_6′_), 3.14 (d, *J* = 10.0 Hz, 1H, H_9_), 3.08 (t, *J* = 10.8 Hz, 1H, H_10′_), 2.89 (d, *J* = 10.1 Hz, 1H, H_9′_), 2.47 (s, 3H, H_1_ or H_19_), 2.43 (s, 3H, H_1_ or H_19_), 2.25 (qd, *J* = 12.1, 2.3 Hz, 2H, H_12′_ + H_13′_), 2.05 (dtd, *J* = 11.3, 7.3, 4.5 Hz, 1H, H_7_), 1.72 (td, *J* = 12.8, 4.3 Hz, 1H, H_11_ or H_14_), 1.52 (td, *J* = 12.7, 4.2 Hz, 1H, H_11_ or H_14_), 1.39–1.26 (m, 2H, H_11′_ + H_14′_). ^13^C{^1^H} NMR (101 MHz, CDCl_3_) δ 144.2 (C_5_ or C_15_), 144.1 (C_5_ or C_15_), 133.3 (C_2_ or C_18_), 132.8 (C_2_ or C_18_), 130.0 (C_3_ or C_17_), 129.9 (C_3_ or C_17_), 127.7 (C_4_ or C_16_), 127.5 (C_4_ or C_16_), 54.9 (C_9_), 50.2 (C_6_), 49.6 (C_7_), 43.6 (C_12_ or C_13_), 43.1 (C_12_ or C_13_), 42.7 (C_8_), 42.6 (C_10_), 34.1 (C_11_ or C_14_), 28.2 (C_11_ or C_14_), 21.7 (C_1_ + C_19_). Mp: 102.8–104.0 °C. IR: ν_max_ 2843, 1341, 1328, 1157; cm^–1^. HRMS: *m*/*z* calculated for C_23_H_30_ClN_2_O_4_S_2_^+^, 497.1330 [M + H]^+^. Found *m*/*z* 497.1335, Δ = 0.9 ppm. Rf (30% EtOAc in Pet ether) = 0.23.
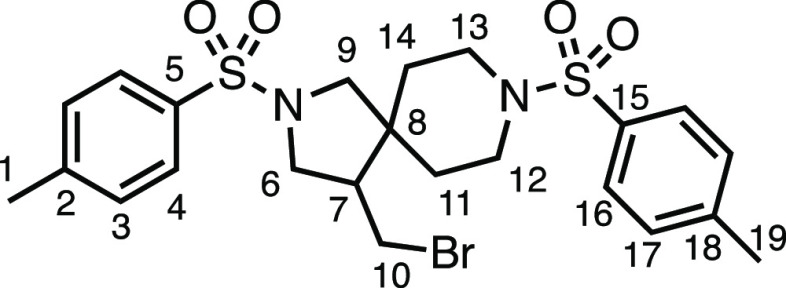


#### 4-(Bromomethyl)-2,8-ditosyl-2,8-diazaspiro[4.5]decane (**3ad**)

The compound was prepared according to General Procedure E3. Flash column chromatography (20% EtOAc in Pet ether) afforded the product as a white solid (84.6 mg, 78%). ^1^H NMR (400 MHz, CDCl_3_) δ 7.64 (app d, *J* = 8.3 Hz, 2H, H_4_ or H_16_), 7.59 (app d, *J* = 8.3 Hz, 2H, H_4_ or H_16_), 7.35 (app d, *J* = 8.0 Hz, 2H, H_3_ or H_17_), 7.30 (app d, *J* = 8.0 Hz, 2H, H_3_ or H_17_), 3.57 (dd, *J* = 10.5, 7.7 Hz, 3H, H_6_ + H_12_ + H_13_), 3.29 (dd, *J* = 10.1, 3.9 Hz, 1H, H_10_), 3.20 (d, *J* = 10.2 Hz, 1H, H_9_), 3.16 (dd, *J* = 10.6, 7.5 Hz, 1H, H_6′_), 2.90 (app t, *J* = 10.6 Hz, 1H, H_10′_), 2.87 (d, *J* = 10.1 Hz, 2H, H_9′_), 2.47 (s, 3H, H_1_ or H_19_), 2.43 (s, 3H, H_1_ or H_19_), 2.30–2.16 (m, 2H, H_12′_ + H_13′_), 2.11 (dtd, *J* = 11.3, 7.6, 3.9 Hz, 1H, H_7_), 1.73 (td, *J* = 12.9, 12.4, 4.5 Hz, 1H, H_11_ or H_14_), 1.51 (td, *J* = 12.7, 4.3 Hz, 1H, H_11_ or H_14_), 1.36–1.26 (m, 2H, H_11′_ + H_14′_). ^13^C{^1^H} NMR (101 MHz, CDCl_3_) δ 144.2 (C_5_ or C_15_), 144.1 (C_5_ or C_15_), 133.4 (C_2_ or C_18_), 132.8 (C_2_ or C_18_), 130.1 (C_3_ or C_17_), 129.9 (C_3_ or C_17_), 127.7 (C_4_ or C_16_), 127.5 (C_4_ or C_16_), 54.9 (C_9_), 51.3 (C_6_), 50.0 (C_7_), 43.6 (C_12_ or C_13_), 43.2 (C_8_), 43.0 (C_12_ or C_13_), 34.0 (C_11_ or C_14_), 30.2 (C_10_), 28.0 (C_11_ or C_14_), 21.7 (C_1_ or C_19_), 21.7 (C_1_ or C_19_). Mp: Decomposed above 160 °C. IR: ν_max_ 2923, 2845, 1340, 1328, 1157; cm^–1^. HRMS: *m*/*z* calculated for C_23_H_30_BrN_2_O_4_S_2_^+^, 541.0825 [M + H]^+^. Found *m*/*z* 541.0830, Δ = 0.9 ppm. Rf (30% EtOAc in Pet ether) = 0.24.
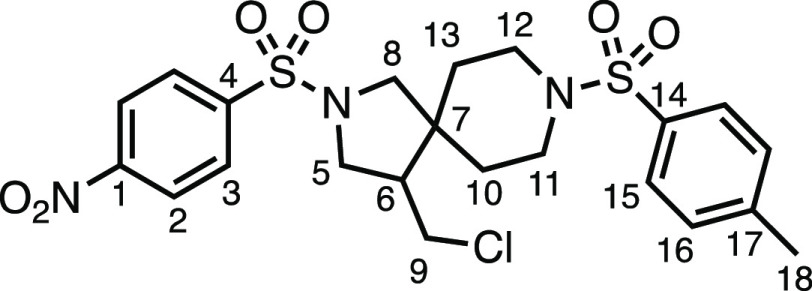


#### 4-(Chloromethyl)-2-((4-nitrophenyl)sulfonyl)-8-tosyl-2,8-diazaspiro[4.5]decane (**3ae**)

The compound was prepared according to General Procedure E1. Flash column chromatography (DCM) afforded the product as a white solid (55.7 mg, 53%). ^1^H NMR (400 MHz, CDCl_3_) δ 8.37 (app d, *J* = 8.8 Hz, 2H, H_2_), 7.96 (app d, *J* = 8.8 Hz, 2H, H_3_), 7.62 (app d, *J* = 8.2 Hz, 2H, H_15_), 7.36 (app d, *J* = 8.1 Hz, 2H, H_16_), 3.64–3.54 (m, 3H, H_5_ + H_11_ + H_12_), 3.47 (dd, *J* = 11.2, 4.1 Hz, 1H, H_9_), 3.27 (dd, *J* = 10.6, 6.8 Hz, 1H, H_5′_), 3.22 (d, *J* = 9.9 Hz, 1H, H_8_), 3.18–3.09 (m, 1H, H_9′_), 2.97 (d, *J* = 9.9 Hz, 1H, H_8′_), 2.48 (s, 3H, H_1_), 2.39–2.26 (m, 2H, H_11′_ + H_12′_), 2.13 (dtd, *J* = 11.3, 7.5, 4.4 Hz, 1H, H_6_), 1.79 (td, *J* = 13.4, 4.3 Hz, 1H, H_10_ or H_13_), 1.67–1.55 (m, 1H, H_10_ or H_13_), 1.47 (d, *J* = 13.6 Hz, 2H, H_10′_ + H_13′_). ^13^C{^1^H} NMR (101 MHz, CDCl_3_) δ 150.3 (C_1_), 144.1 (C_17_), 142.5 (C_4_), 132.8 (C_14_), 130.0 (C_16_), 128.5 (C_3_), 127.6 (C_15_), 124.5 (C_2_), 55.0 (C_8_), 50.1 (C_5_), 49.3 (C_6_), 43.4 (C_11_ or C_12_), 43.0 (C_11_ or C_12_), 42.9 (C_7_), 42.5 (C_9_), 34.1 (C_10_ or C_13_), 28.2 (C_10_ or C_13_), 21.6 (C_18_). Mp: Decomposed above 200 °C. IR: ν_max_ 2853, 1529, 1347, 1327, 1161 cm^–1^. HRMS: *m*/*z* calculated for C_22_H_27_ClN_3_O_6_S_2_^+^, 528.1024 [M + H]^+^. Found *m*/*z* 528.1020, Δ = −0.7 ppm. Rf (DCM) = 0.13.
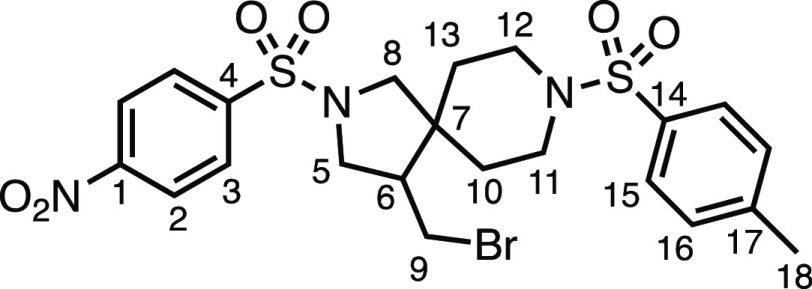


#### 4-(Bromomethyl)-2-((4-nitrophenyl)sulfonyl)-8-tosyl-2,8-diazaspiro[4.5]decane (**3af**)

The compound was prepared according to General Procedure E2. Flash column chromatography (DCM) followed by recrystallization by vapor diffusion from DCM (Pet ether as the antisolvent) afforded the product as a white crystalline solid (68.9 mg, 60%). ^1^H NMR (400 MHz, CDCl_3_) δ 8.38 (app d, *J* = 8.8 Hz, 2H, H_2_), 7.97 (app d, *J* = 8.8 Hz, 2H, H_3_), 7.62 (app d, *J* = 8.1 Hz, 2H, H_18_), 7.36 (app d, *J* = 8.1 Hz, 2H, H_19_), 3.63 (dd, *J* = 10.5, 7.8 Hz, 3H, H_5_ + H_11_ + H_12_), 3.37–3.20 (m, 3H, H_5′_ + H_8_ + H_9_), 2.95 (app t, *J* = 10.5 Hz, 1H, H_9′_), 2.92 (d, *J* = 9.9 Hz, 1H, H_8′_), 2.48 (s, 3H, H_21_), 2.38–2.23 (m, 2H, H_11′_ + H_12′_), 2.18 (dtd, *J* = 11.1, 7.5, 3.8 Hz, 1H, H_6_), 1.80 (td, *J* = 13.4, 4.3 Hz, 1H, H_10_ or H_13_), 1.65–1.56 (m, 1H, H_10_ or H_13_), 1.43 (d, *J* = 13.5 Hz, 2H, H_10′_ + H_13′_). ^13^C{^1^H} NMR (101 MHz, CDCl_3_) δ 150.4 (C_1_), 144.3 (C_17_), 142.7 (C_4_), 133.0 (C_14_), 130.1 (C_16_), 128.6 (C_3_), 127.7 (C_15_), 124.7 (C_2_), 55.1 (C_8_), 51.3 (C_5_), 49.9 (C_6_), 43.6 (C_11_ or C_12_), 43.5 (C_7_), 43.5 (C_11_ or C_12_), 43.0 (C_11_ or C_12_), 34.1 (C_10_ or C_13_), 30.0 (C_9_), 28.0 (C_10_ or C_13_), 21.8 (C_18_). Mp: 206.8–209.9 °C. IR: ν_max_ 2853, 1529, 1347, 1327, 1161 cm^–1^. HRMS: *m*/*z* calculated for C_22_H_27_BrN_3_O_6_S_2_^+^, 572.0519 [M + H]^+^. Found *m*/*z* 572.0517, Δ = −0.4 ppm. Rf (DCM) = 0.16.
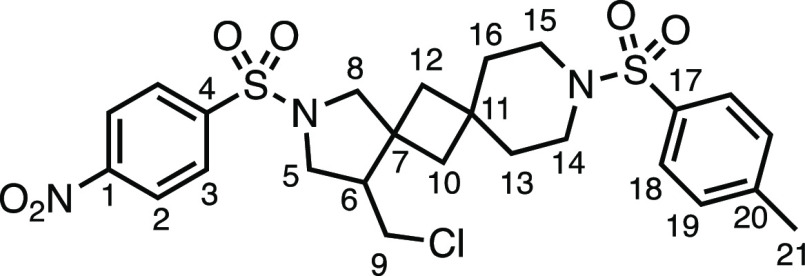


#### 4-(Chloromethyl)-2-((4-nitrophenyl)sulfonyl)-10-tosyl-2,10-diazadispiro[4.1.5^7^.1^5^]tridecane (**3ag**)

The compound was prepared according to General Procedure E1. Flash column chromatography (20–30% EtOAc in Pet ether) afforded the product as a white solid (56.8 mg, 50%). ^1^H NMR (400 MHz, CDCl_3_) δ 8.36 (app d, *J* = 8.7 Hz, 2H, H_2_), 7.98 (app d, *J* = 8.7 Hz, 2H, H_3_), 7.58 (app d, *J* = 8.0 Hz, 2H, H_18_), 7.30 (app d, *J* = 8.0 Hz, 2H, H_19_), 3.40 (dt, *J* = 10.7, 5.5 Hz, 2H, H_5_ + H_9_), 3.30 (td, *J* = 7.8, 3.6 Hz, 2H, H_5_ + H_8_), 3.18 (d, *J* = 9.7 Hz, 1H, H_8′_), 2.94–2.75 (m, 5H, H_9′_ + H_14_ + H_15_), 2.42 (s, 3H, H_21_), 2.17 (app dq, *J* = 10.2, 5.4, 4.5 Hz, 1H, H_6_), 1.78 (d, *J* = 12.9 Hz, 1H, H_10_ or H_12_), 1.70–1.49 (m, 7H, H_10_ + H_12_ + H_13_ + H_16_). ^13^C{^1^H} NMR (101 MHz, CDCl_3_) δ 150.3 (C_4_), 143.8 (C_17_), 142.8 (C_1_), 133.0 (C_20_), 129.8 (C_19_), 128.5 (C_3_), 127.7 (C_18_), 124.5 (C_2_), 59.7 (C_8_), 50.3 (C_6_), 49.7 (C_5_), 43.1 (C_10_ or C_12_), 42.8 (C_9_ or C_14_ or C_15_), 42.8 (C_9_ or C_14_ or C_15_), 42.8 (C_9_ or C_14_ or C_15_), 40.8 (C_7_), 38.0 (C_13_ or C_16_), 37.2 (C_13_ or C_16_), 35.4 (C_10_ or C_12_), 31.0 (C_11_), 21.6 (C_21_). Mp: 188.0–189.0 °C. IR: ν_max_ 2921, 1528, 1347, 1328, 1161 cm^–1^. HRMS: *m*/*z* calculated for C_25_H_31_ClN_3_O_6_S_2_^+^, 568.1338 [M + H]^+^. Found *m*/*z* 568.1336, Δ = −0.3 ppm. Rf (40% EtOAc in Pet ether) = 0.56.
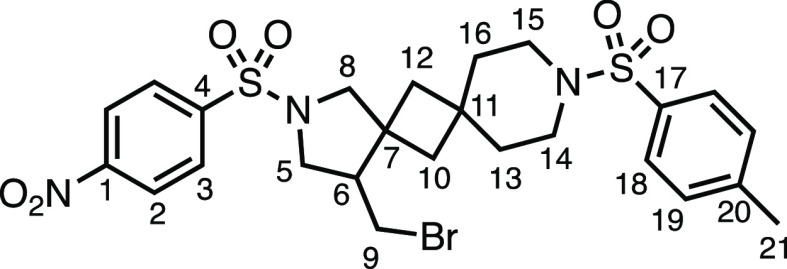


#### 4-(Bromomethyl)-2-((4-nitrophenyl)sulfonyl)-10-tosyl-2,10-diazadispiro[4.1.5^7^.1^5^]tridecane (**3ah**)

The compound was prepared according to General Procedure E3. Flash column chromatography (20–30% EtOAc in Pet ether) afforded the product as a white solid (77.4 mg, 63%). ^1^H NMR (400 MHz, CDCl_3_) δ 8.35 (app d, *J* = 8.8 Hz, 2H, H_2_), 7.98 (app d, *J* = 8.8 Hz, 2H, H_3_), 7.57 (app d, *J* = 8.1 Hz, 2H, H_18_), 7.30 (app d, *J* = 8.1 Hz, 2H, H_19_), 3.42–3.36 (m, 1H, H_5_), 3.34–3.23 (m, 3H H_5′_ + H_8_ + H_9_), 3.20 (d, *J* = 9.8 Hz, 1H, H_8′_), 2.92–2.75 (m, 4H, H_14_ + H_15_), 2.63 (t, *J* = 10.6 Hz, 1H, H_9′_), 2.42 (s, 3H, H_21_), 2.22 (app tt, *J* = 6.4, 3.9 Hz, 1H, H_6_), 1.76 (d, *J* = 12.9 Hz, 1H, H_10_ or H_12_), 1.66–1.47 (m, 7H, H_10_ + H_12_ + H_13_ + H_16_). ^13^C{^1^H} NMR (101 MHz, CDCl_3_) δ 150.2 (C_4_), 143.8 (C_17_), 142.7 (C_1_), 133.0 (C_20_), 129.8 (C_19_), 128.5 (C_3_), 127.6 (C_18_), 124.5 (C_2_), 59.5 (C_8_), 50.6 (C_5_), 50.5 (C_6_), 42.8 (C_10_ or C_12_ or C_14_ or C_15_), 42.8 (C_10_ or C_12_ or C_14_ or C_15_), 42.8 (C_10_ or C_12_ or C_14_ or C_15_), 41.3 (C_7_), 37.9 (C_13_ or C_16_), 37.2 (C_13_ or C_16_), 35.3 (C_10_ or C_12_), 30.9 (C_11_), 30.7 (C_9_), 21.6 (C_21_). Mp: Decomposed above 200 °C. IR: ν_max_ 2921, 2841, 1535, 1350, 1330, 1161 cm^–1^. HRMS: *m*/*z* calculated for C_25_H_31_BrN_3_O_6_S_2_^+^, 612.0832 [M + H]^+^. Found *m*/*z* 612.0840, Δ = 1.3 ppm. Rf (30% EtOAc in Pet ether) = 0.25.
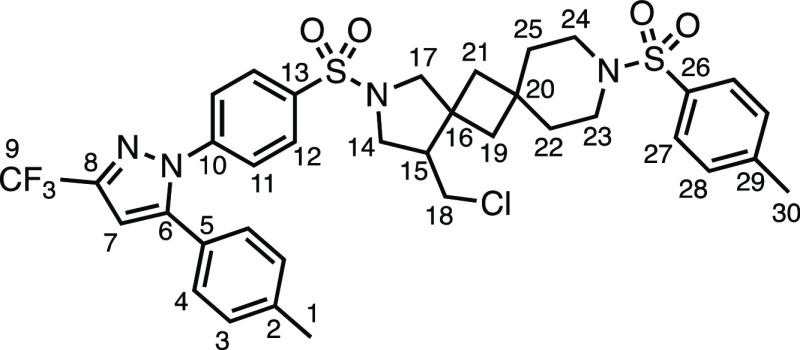


#### 4-(Chloromethyl)-2-((4-(5-(*p*-tolyl)-3-(trifluoromethyl)-1*H*-pyrazol-1-yl)phenyl)sulfonyl)-10-tosyl-2,10-diazadispiro[4.1.5^7^.1^5^]tridecane (**3ai**)

The compound was prepared according to General Procedure E1. Flash column chromatography (15–30% EtOAc in Pet ether, then 5% MeCN in toluene) afforded the product as an off-white crystalline solid (74.2 mg, 50%). ^1^H NMR (400 MHz, CDCl_3_) δ 7.79 (app d, *J* = 8.7 Hz, 2H, H_27_), 7.61 (app d, *J* = 8.2 Hz, 2H, H_12_), 7.49 (app d, *J* = 8.7 Hz, 2H, H_28_), 7.31 (app d, *J* = 8.0 Hz, 2H, H_11_), 7.16 (app d, *J* = 8.1 Hz, 2H, H_3_), 7.08 (app d, *J* = 8.1 Hz, 2H, H_4_), 6.74 (s, 1H, H_7_), 3.43 (dd, *J* = 11.1, 4.1 Hz, 1H, H_18_), 3.39 (dd, *J* = 10.7, 6.6 Hz, 1H, H_14_), 3.29 (d, *J* = 9.5 Hz, 1H, H_17_), 3.28 (dd, *J* = 10.6, 4.2 Hz, 2H, H_14′_), 3.12 (d, *J* = 9.6 Hz, 1H, H_17′_), 2.98–2.77 (m, 5H, H_18′_ + H_23_ + H_24_), 2.43 (s, 3H, H_30_), 2.37 (s, 3H, H_1_), 2.14 (ddt, *J* = 10.4, 6.6, 4.0 Hz, 1H, H_15_), 1.80 (d, *J* = 12.9 Hz, 1H, H_19_), 1.66–1.51 (m, 7H, H_19′_ + H_21_ + H_22_ + H_25_). ^13^C{^1^H} NMR (101 MHz, CDCl_3_) δ 145.4 (C_6_), 144.3 (d, *J* = 38.6 Hz, C_8_), 143.8 (C_26_), 142.9 (C_13_), 140.0 (C_2_), 136.3 (C_10_), 133.1 (C_29_), 129.9 (C_3_), 129.8 (C_28_), 128.9 (C_4_), 128.5 (C_12_), 127.8 (C_27_), 125.8 (C_5_), 125.7 (C_11_), 121.2 (q, *J* = 269.2 Hz, C_9_), 106.5 (C_7_), 59.8 (C_17_), 50.5 (C_15_), 49.8 (C_14_), 43.3 (C_21_), 43.0 (C_14_ or C_23_ or C_24_), 42.9 (C_18_ or C_23_ or C_24_), 42.9 (C_14_ or C_23_ or C_24_), 40.8 (C_16_), 38.2 (C_22_ or C_25_), 37.3 (C_22_ or C_25_), 35.6 (C_19_), 31.1 (C_20_), 21.7 (C_30_), 21.5 (C_1_). ^19^F NMR (376 MHz, CDCl_3_) δ −62.4 (CF_3_). Mp: 199.0–201.5 °C. IR: ν_max_ 2923, 1346, 1235, 1161 cm^–1^. HRMS: *m*/*z* calculated for C_36_H_38_ClF_3_N_4_ O_4_S_2_^+^, 747.2048 [M + H]^+^. Found *m*/*z* 747.2049, Δ = 0.2 ppm. Rf (30% EtOAc in Pet ether) = 0.40.
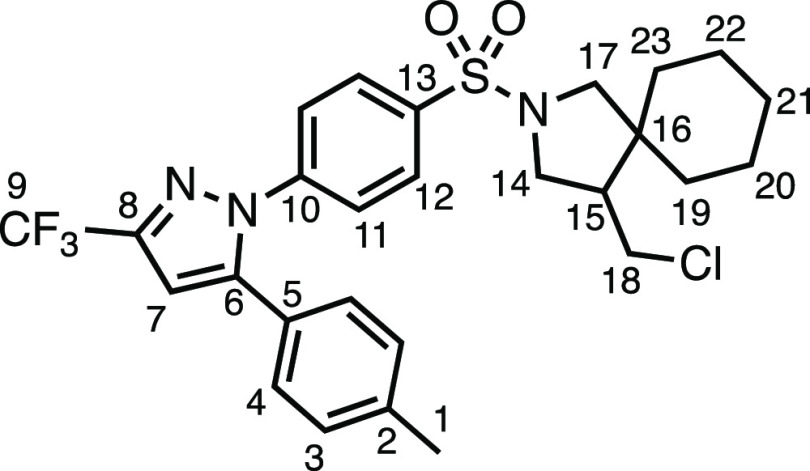


#### 4-(Chloromethyl)-2-((4-(5-(*p*-tolyl)-3-(trifluoromethyl)-1*H*-pyrazol-1-yl)phenyl)sulfonyl)-2-azaspiro[4.5]decane (**3aj**)

The compound was prepared according to General Procedure E1. Flash column chromatography (10–30% Et_2_O in Pet ether) afforded the product as a white solid (89.0 mg, 81%). ^1^H NMR (400 MHz, CDCl_3_) δ 7.83 (app d, *J* = 8.5 Hz, 2H, H_12_), 7.50 (app d, *J* = 8.5 Hz, 2H, H_11_), 7.16 (app d, *J* = 8.0 Hz, 2H, H_3_), 7.09 (app d, *J* = 8.0 Hz, 2H, H_4_), 6.74 (s, 1H, H_7_), 3.56 (td, *J* = 10.5, 5.9 Hz, 2H, H_14_ + H_18_), 3.56 (td, *J* = 10.5, 5.9 Hz, 2H, H_14_ + H_18_), 3.33 (d, *J* = 10.0 Hz, 1H, H_17_), 3.24 (dd, *J* = 10.2, 7.0 Hz, 1H, H_14′_), 3.14 (t, *J* = 10.8 Hz, 1H, H_18′_), 3.05 (d, *J* = 10.0 Hz, 1H, H_17′_), 2.37 (s, 3H, H_1_), 2.15–2.04 (m, 1H, H_15_), 1.61–1.08 (m, 10H, H_19–20_). ^13^C{^1^H} NMR (101 MHz, CDCl_3_) δ 145.4 (C_6_), 144.2 (q, *J* = 38.5 Hz, C_8_), 142.7 (C_13_), 139.9 (C_5_), 136.5 (C_10_), 129.9 (C_3_), 128.8 (C_4_), 128.4 (C_12_), 125.8 (C_2_), 125.7 (C_11_), 121.2 (q, *J* = 269.2 Hz, C_9_), 106.4 (d, *J* = 1.8 Hz, C_7_), 56.5 (C_17_), 50.6 (C_14_), 50.1 (C_15_), 45.0 (C_16_), 43.3 (C_18_), 35.7 (C_19–23_), 29.2 (C_19–23_), 25.8 (C_19–23_), 23.3 (C_19–23_), 22.8 (C_19–23_), 21.4 (C_1_). Mp: 82.0–86.0 °C. IR: ν_max_ 2925, 1471, 1347, 1235, 1159, 1129 cm^–1^. HRMS: *m*/*z* calculated for C_27_H_30_ClF_3_N_3_O_2_S^+^, 552.1694 [M + H]^+^. Found *m*/*z* 552.1696, Δ = 0.4 ppm. Rf (30% Et_2_O in Pet ether) = 0.34.
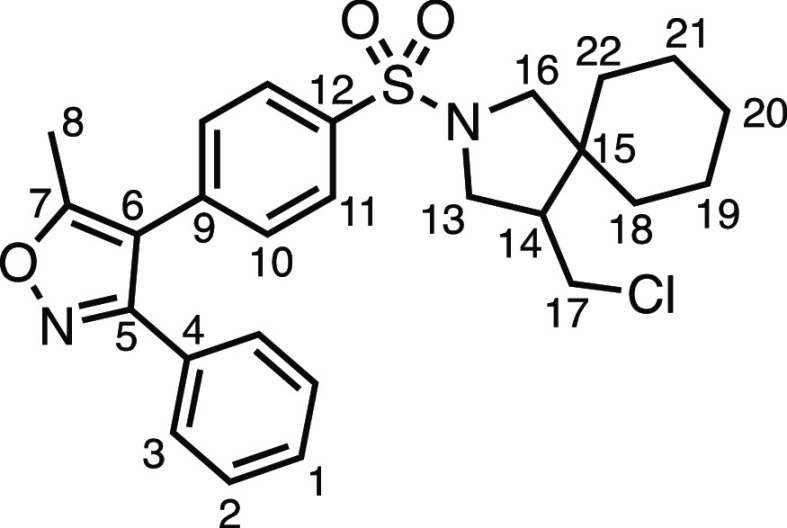


#### 4-(4-((4-(Chloromethyl)-2-azaspiro[4.5]decan-2-yl)sulfonyl)phenyl)-5-methyl-3-phenylisoxazole (**3ak**)

The compound was prepared according to General Procedure E1. Flash column chromatography (10–30% Et_2_O in Pet ether) afforded the product as a colorless oil (70.0 mg, 72%). ^1^H NMR (400 MHz, CDCl_3_) δ 7.84 (app d, *J* = 8.2 Hz, 2H, H_11_), 7.42–7.27 (m, 7H, H_1_ + H_2_ + H_3_ + H_10_), 3.61 (dd, *J* = 10.3, 7.3 Hz, 1H, H_13_), 3.54 (dd, *J* = 10.9, 3.9 Hz, 1H, H_17_), 3.32 (d, *J* = 9.9 Hz, 1H, H_16_), 3.28 (dd, *J* = 10.4, 6.6 Hz, 1H, H_13′_), 3.14 (d, *J* = 10.2 Hz, 1H, H_16′_), 3.09 (app t, *J* = 10.8 Hz, 1H, H_17′_), 2.47 (s, 3H, H_8_), 2.17–2.06 (m, 1H, H_14_), 1.57–1.12 (m, 10H, H_18–22_). ^13^C{^1^H} NMR (101 MHz, CDCl_3_) δ 167.3 (C_7_), 161.1 (C_5_), 136.0 (C_9_), 135.4 (C_12_), 130.4 (C_10_), 129.8 (C_1_), 128.8 (C_2_ or C_3_), 128.6 (C_4_), 128.5 (C_2_ or C_3_), 127.8 (C_11_), 114.6 (C_6_), 56.3 (C_16_), 50.4 (C_13_), 49.9 (C_14_), 45.0 (C_15_), 43.3 (C_17_), 35.6 (C_18–22_), 29.2 (C_18–22_), 25.8 (C_18–22_), 23.2 (C_18–22_), 22.8 (C_18–22_), 11.8 (C_8_). IR: ν_max_ 2928, 2855, 1343, 1159 cm^–1^. HRMS: *m*/*z* calculated for C_26_H_30_ClN_2_ O_3_S^+^, 485.1660 [M + H]^+^. Found *m*/*z* 485.1667, Δ = 1.3 ppm. Rf (30% EtOAc in Pet ether) = 0.69.
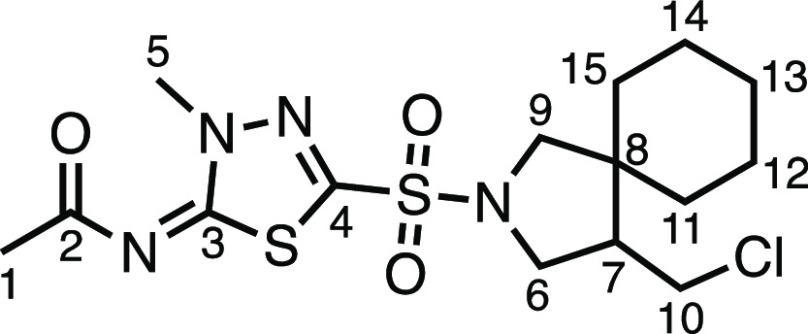


#### *N*-(5-((4-(Chloromethyl)-2-azaspiro[4.5]decan-2-yl)sulfonyl)-3-methyl-1,3,4-thiadiazol-2(3*H*)-ylidene)acetamide (**3al**)

The compound was prepared according to General Procedure E1. Flash column chromatography (10–30% EtOAc in Pet ether, then 3–5% MeCN in toluene) afforded the product as a white solid (36.7 mg, 45%). ^1^H NMR (400 MHz, CDCl_3_) δ 4.00 (s, 3H, H_5_), 3.80 (dd, *J* = 10.6, 7.4 Hz, 1H, H_6_), 3.61 (dd, *J* = 11.0, 4.1 Hz, 1H, H_10_), 3.57 (d, *J* = 10.2 Hz, 1H, H_9_), 3.48 (dd, *J* = 10.6, 6.8 Hz, 1H, H_6′_), 3.30 (t, *J* = 10.7 Hz, 1H, H_10′_), 3.29 (d, *J* = 10.1 Hz, 1H, H_9′_), 2.36 (s, 3H, H_1_), 2.25 (dtd, *J* = 10.9, 7.1, 4.1 Hz, 1H, H_7_), 1.65–1.16 (m, 10H, H_11–15_). ^13^C{^1^H} NMR (101 MHz, CDCl_3_) δ 181.1 (C_2_), 164.9 (C_3_), 153.9 (C_4_), 56.8 (C_9_), 50.9 (C_6_), 50.1 (C_7_), 45.3 (C_8_), 43.1 (C_10_), 38.6 (C_5_), 35.6 (C_11–15_), 29.1 (C_11–15_), 26.7 (C_1_), 25.8 (C_11–15_), 23.3 (C_11–15_), 22.7 (C_11–15_). Mp: 157.0–158.4 °C. IR: ν_max_ 2929, 2848, 1618, 1486, 1366, 1301, 1242, 1163 cm^–1^. HRMS: *m*/*z* calculated for C_15_H_24_ClN_4_ O_3_S_2_^+^, 407.0979 [M + H]^+^. Found *m*/*z* 407.0976, Δ = 0.8 ppm. Rf (30% EtOAc in Pet ether) = 0.18.
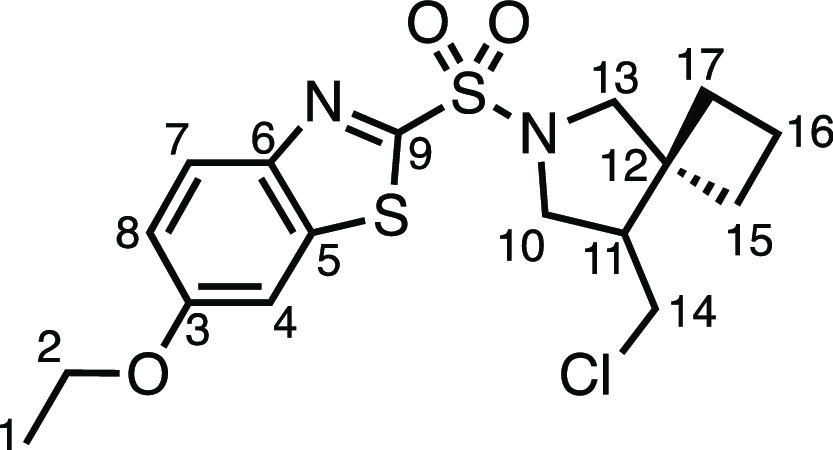


#### 2-((8-(Chloromethyl)-6-azaspiro[3.4]octan-6-yl)sulfonyl)-6-ethoxybenzo[*d*]thiazole (**3am**)

The compound was prepared according to General Procedure E1. Flash column chromatography (7–10% EtOAc) afforded the product as an off-white solid (29.5 mg, 37%). ^1^H NMR (500 MHz, CDCl_3_) δ 8.03 (d, *J* = 9.1 Hz, 1H, H_7_), 7.34 (d, *J* = 2.5 Hz, 1H, H_4_), 7.17 (dd, *J* = 9.1, 2.5 Hz, 1H, H_8_), 4.11 (q, *J* = 7.0 Hz, 2H, H_2_), 3.71 (dd, *J* = 10.6, 6.5 Hz, 1H, H_10_), 3.68 (d, *J* = 10.0 Hz, 1H, H_13_), 3.61 (dd, *J* = 10.6, 4.4 Hz, 1H, H_10′_), 3.56 (d, *J* = 10.3 Hz, 1H, H_13′_), 3.56 (dd, *J* = 10.7, 3.9 Hz, 1H, H_14_), 3.19 (t, *J* = 10.8 Hz, 1H, H_14′_), 2.37–2.28 (m, 1H, H_11_), 2.05 (td, *J* = 10.6, 9.9, 6.3 Hz, 1H, H_15_), 1.94–1.71 (m, 5H, H_15′-17_), 1.47 (t, *J* = 7.0 Hz, 3H, H_1_). ^13^C{^1^H} NMR (126 MHz, CDCl_3_) δ 160.5 (C_9_), 159.0 (C_3_), 147.0 (C_5_), 138.1 (C_6_), 125.9 (C_7_), 118.3 (C_8_), 104.1 (C_4_), 64.4 (C_2_), 58.7 (C_13_), 50.7 (C_10_), 49.6 (C_11_), 47.7 (C_12_), 43.1 (C_14_), 32.6 (C_17_), 26.0 (C_15_), 16.3 (C_16_), 14.8 (C_1_). Mp: 100.0–102.1 °C. IR: ν_max_ 2980, 2927, 2874, 1599, 1485, 1470, 1365, 1255, 1226, 1156 cm^–1^. HRMS: *m*/*z* calculated for C_17_H_22_ClN_2_O_3_S_2_^+^, 407.0755 [M + H]^+^. Found *m*/*z* 401.0761, Δ = 1.4 ppm. Rf (20% EtOAc in Pet ether) = 0.28.
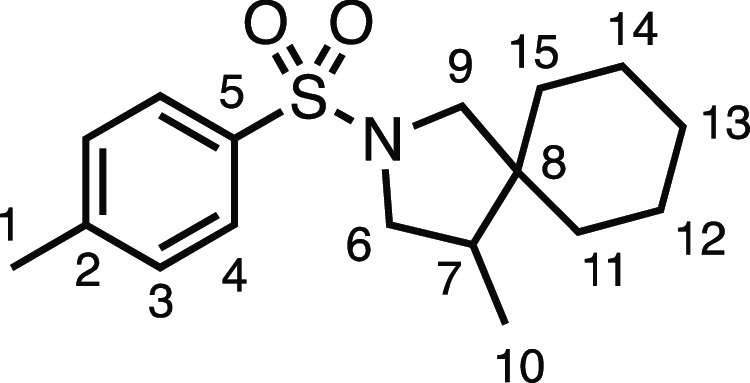


#### 4-Methyl-2-tosyl-2-azaspiro[4.5]decane (**4a**)

The compound was prepared according to General Procedure F1. Flash column chromatography (5% EtOAc in Pet ether) afforded the product as a white solid (46.5 mg, 76%). ^1^H NMR (400 MHz, CDCl_3_) δ 7.71 (app d, *J* = 8.1 Hz, 2H, H_4_), 7.30 (app d, *J* = 8.1 Hz, 2H, H_3_), 3.44 (dd, *J* = 9.5, 7.4 Hz, 1H, H_6_), 3.38 (d, *J* = 10.1 Hz, 1H, H_9_), 2.94 (d, *J* = 10.1 Hz, 1H, H_9′_), 2.85 (dd, *J* = 9.5, 8.5 Hz, 1H, H_6′_), 2.41 (s, 3H, H_1_), 1.73 (h, *J* = 7.2 Hz, 1H, H_7_), 1.58–1.39 (m, 3H, H_11–15_), 1.28–0.85 (m, 7H, H_11–15_), 0.76 (d, *J* = 7.0 Hz, 3H, H_10_). ^13^C{^1^H} NMR (101 MHz, CDCl_3_) δ 143.3 (C_5_), 134.3 (C_2_), 129.6 (C_3_), 127.5 (C_4_), 56.2 (C_9_), 53.3 (C_6_), 44.2 (C_8_), 42.2 (C_7_), 35.1 (C_11–15_), 28.0 (C_11–15_), 26.1 (C_11–15_), 23.7 (C_11–15_), 22.8 (C_11–15_), 21.6 (C_1_), 12.0 (C_10_). Mp: 92.2–95.4 °C. IR: ν_max_ 2925, 2857, 1335, 1153 cm^–1^. HRMS: *m*/*z* calculated for C_17_H_26_NO_2_S^+^, 308.1679 [M + H]^+^. Found *m*/*z* 308.1674, Δ = −1.6 ppm. Rf (10% EtOAc in Pet ether) = 0.35.
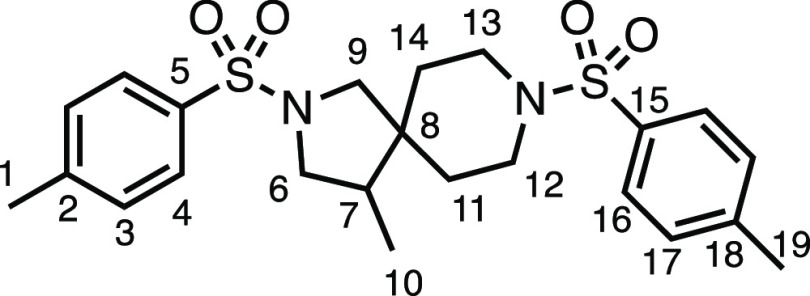


#### 4-Methyl-2,8-ditosyl-2,8-diazaspiro[4.5]decane (**4b**)

The compound was prepared according to General Procedure F1. Flash column chromatography (10–30% EtOAc in Pet ether) afforded the product as a white solid (59.0 mg, 64%). ^1^H NMR (400 MHz, CDCl_3_) δ 7.62 (app d, *J* = 8.6 Hz, 2H, H_4_ or H_16_), 7.60 (app d, *J* = 8.6 Hz, 2H, H_4_ or H_16_), 7.35 (app d, *J* = 8.1 Hz, 2H, H_3_ or H_17_), 7.28 (app d, *J* = 8.0 Hz, 2H, H_3_ or H_17_), 3.52 (app t, *J* = 11.8 Hz, 2H, H_12_ + H_13_), 3.40 (dd, *J* = 9.9, 7.6 Hz, 1H, H_6_), 3.17 (d, *J* = 10.1 Hz, 1H, H_9_), 2.83 (dd, *J* = 9.8, 8.5 Hz, 1H, H_6′_), 2.78 (d, *J* = 10.2 Hz, 1H, H_9′_), 2.47 (s, 3H, H_1_ or H_19_), 2.42 (s, 3H, H_1_ or H_19_), 2.27–2.12 (m, 2H, H_12′_ + H_13′_), 1.72 (dt, *J* = 14.8, 7.3 Hz, 1H, H_7_), 1.63 (td, *J* = 13.1, 4.5 Hz, 1H, H_11_ or H_14_), 1.47 (td, *J* = 12.7, 4.1 Hz, 1H, H_11_ or H_14_), 1.22–1.14 (m, 1H, H_11′_ or H_14′_), 1.10–1.01 (m, 1H, H_11′_ or H_14′_), 0.74 (d, *J* = 6.9 Hz, 3H, H_10_). ^13^C{^1^H} NMR (101 MHz, CDCl_3_) δ 144.0 (C_2_ or C_18_), 143.7 (C_2_ or C_18_), 133.8 (C_5_ or C_15_), 132.9 (C_5_ or C_15_), 130.0 (C_3_ or C_17_), 129.8 (C_3_ or C_17_), 127.7 (C_4_ or C_16_), 127.4 (C_4_ or C_16_), 54.7 (C_9_), 52.9 (C_6_), 43.9 (C_12_ or C_13_), 43.1 (C_12_ or C_13_), 42.1 (C_8_), 41.7 (C_7_), 33.4 (C_11_ or C_14_), 27.2 (C_11_ or C_14_), 21.7 (C_1_ or C_19_), 21.7 (C_1_ or C_19_), 11.9 (C_10_). Mp: 61.1–64.2 °C. IR: ν_max_ 2923, 1339, 1155 cm^–1^. HRMS: *m*/*z* calculated for C_23_H_31_N_2_O_4_S_2_^+^, 463.1720 [M + H]^+^. Found *m*/*z* 463.1727, Δ = 1.5 ppm. Rf (30% EtOAc in Pet ether) = 0.22.
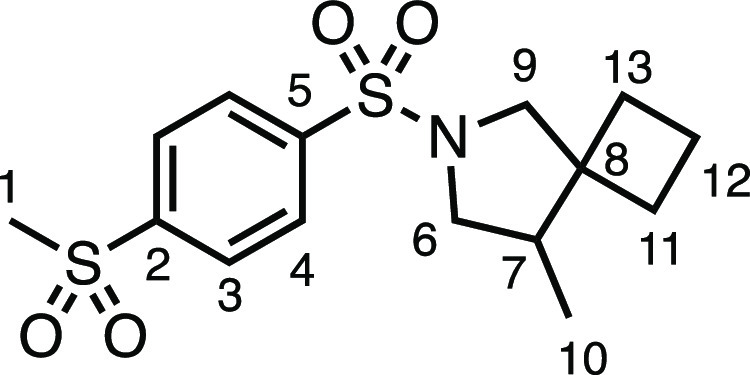


#### 8-Methyl-6-((4-(methylsulfonyl)phenyl)sulfonyl)-6-azaspiro[3.4]octane (**4c**)

The compound was prepared according to General Procedure F1. Flash column chromatography (20–40% EtOAc in Pet ether) afforded the product as an off-white crystalline solid (46.4 mg, 68%). ^1^H NMR (400 MHz, CDCl_3_) δ 8.09 (app dd, *J* = 6.7, 1.7 Hz, 2H, H_3_), 8.01 (app dd, *J* = 6.7, 1.7 Hz, 2H, H_4_), 3.37 (dd, *J* = 9.6, 6.5 Hz, 1H, H_6_), 3.36 (d, *J* = 9.7 Hz, 1H, H_9_), 3.24 (d, *J* = 9.7 Hz, 1H, H_9′_), 3.09 (s, 3H, H_1_), 2.89 (dd, *J* = 9.7, 6.2 Hz, 1H, H_6′_), 1.90 (app ddt, *J* = 13.5, 9.6, 6.5 Hz, 2H, H_7_ + H_11_), 1.81–1.64 (m, 4H, H_12_ + H_13_), 1.56–1.46 (m, 1H, H_11′_), 0.81 (d, *J* = 6.9 Hz, 3H, H_10_). ^13^C{^1^H} NMR (101 MHz, CDCl_3_) δ 144.1 (C_2_), 142.8 (C_5_), 128.3 (C_3_ or C_4_), 128.3 (C_3_ or C_4_), 58.4 (C_9_), 53.3 (C_6_), 48.0 (C_8_), 44.4 (C_1_), 40.8 (C_7_), 30.1 (C_12_), 25.7 (C_11_), 15.7 (C_13_), 12.8 (C_10_). Mp: 148.0–151.0 °C. IR: ν_max_ 3096, 2925, 1335, 1308, 1283, 1157 cm^–1^. HRMS: *m*/*z* calculated for C_15_H_22_NO_4_S_2_^+^, 344.0984 [M + H]^+^. Found *m*/*z* 344.0980, Δ = −1.3 ppm. Rf (40% EtOAc in Pet ether) = 0.28.
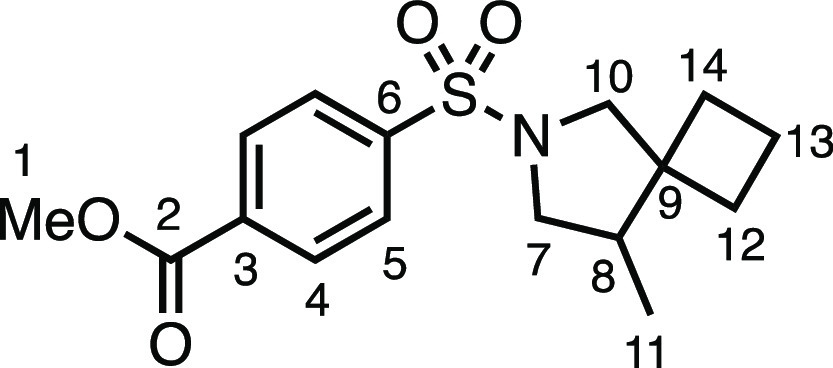


#### Methyl 4-((8-Methyl-6-azaspiro[3.4]octan-6-yl)sulfonyl)benzoate (**4d**)

The compound was prepared according to General Procedure F1. Flash column chromatography (5–10% EtOAc in Pet ether, then 0–2% MeCN in toluene) afforded the product as a white solid (58.1 mg, 90%). ^1^H NMR (400 MHz, CDCl_3_) δ 8.17 (app d, *J* = 8.5 Hz, 2H, H_4_), 7.89 (app d, *J* = 8.5 Hz, 2H, H_5_), 3.94 (s, 3H, H_1_), 3.36 (dd, *J* = 9.7, 6.6 Hz, 1H, H_7_), 3.34 (d, *J* = 9.8 Hz, 1H, H_10_), 3.24 (d, *J* = 9.8 Hz, 1H, H_10′_), 2.89 (dd, *J* = 9.7, 6.0 Hz, 1H, H_7′_), 1.87 (dt, *J* = 12.5, 6.5 Hz, 2H, H_8_ + H_12_), 1.81–1.58 (m, 4H, H_13_ + H_14_), 1.54–1.42 (m, 1H, H_12′_), 0.78 (d, *J* = 6.9 Hz, 3H, H_11_). ^13^C{^1^H} NMR (101 MHz, CDCl_3_) δ 165.8 (C_2_), 141.3 (C_3_), 133.8 (C_6_), 130.3 (C_4_), 127.4 (C_5_), 58.3 (C_10_), 53.3 (C_7_), 52.7 (C_1_), 48.0 (C_9_), 40.9 (C_8_), 30.3 (C_14_), 25.7 (C_12_), 15.7 (C_13_), 12.8 (C_11_). Mp: 83.0–84.3 °C. IR: ν_max_ 2954, 2870, 1726, 1336, 1275, 1154 cm^–1^. HRMS: *m*/*z* calculated for C_16_H_22_NO_4_S^+^, 324.1264 [M + H]^+^. Found *m*/*z* 324.1264, Δ = 0.1 ppm. Rf (20% EtOAc in Pet ether) = 0.29.

### Derivatization Procedures to **5** and **6**

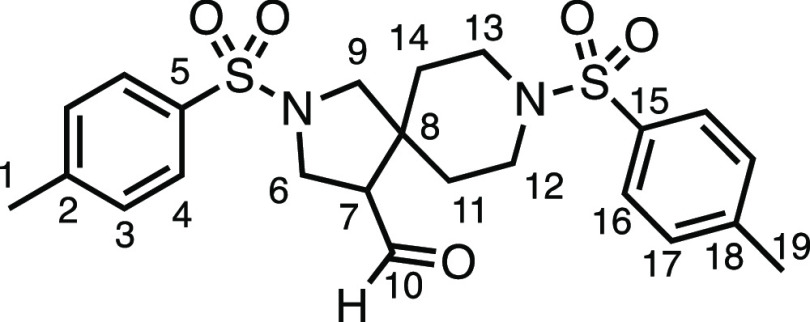



#### 2,8-Ditosyl-2,8-diazaspiro[4.5]decane-4-carbaldehyde (**5**)

The compound was prepared following an adapted literature procedure.^39^ A 20 mL microwave vial was charged with spirocycle **3ad** (199.1 mg, 0.368 mmol, 1.0 equiv) and AgBF_4_ (214.7 mg, 1.162 mmol, 3.0 equiv), placed under nitrogen, and then dry DMSO (7 mL) was added. The mixture was then heated to 100 °C for 16 h before being allowed to cool to room temperature. Et_3_N (61 μL, 0.44 mmol, 1.2 equiv) was then added and the solution stirred at room temperature for 1 h. The mixture was then poured into Et_2_O (100 mL) and washed with H_2_O (100 mL). The aqueous layer was then extracted with Et_2_O (2 × 50 mL) and the organic layers combined. The solvent was removed *in vacuo* and the crude product subjected to flash column chromatography (20–30% EtOAc in Pet ether), which afforded the product as a white crystalline solid (70.7 mg, 40%). ^1^H NMR (400 MHz, CDCl_3_) δ 9.52 (d, *J* = 2.1 Hz, 1H, H_10_), 7.66 (app d, *J* = 8.2 Hz, 4H, H_4_ or H_16_), 7.61 (app d, *J* = 8.2 Hz, 4H, H_4_ or H_16_), 7.36 (app d, *J* = 8.0 Hz, 4H, H_3_ or H_17_), 7.32 (app d, *J* = 8.0 Hz, 4H, H_3_ or H_17_), 3.61–3.34 (m, 4H, H_6_ + H_6′_ + H_12_ + H_13_), 3.09 (d, *J* = 10.1 Hz, 1H, H_9_), 3.04 (d, *J* = 10.1 Hz, 1H, H_9′_), 2.63 (ddd, *J* = 7.9, 6.2, 2.0 Hz, 1H, H_7_), 2.51–2.30 (m, 8H, H_1_ + H_12′_ + H_13′_ + H_19_), 1.88 (ddd, *J* = 13.6, 11.3, 4.3 Hz, 1H, H_11_), 1.71–1.56 (m, 2H, H_11′_ + H_14_), 1.40 (d, *J* = 13.5 Hz, 1H, H_14′_). ^13^C{^1^H} NMR (101 MHz, CDCl_3_) δ 199.3 (C_10_), 144.3 (C_5_ or C_15_), 144.3 (C_5_ or C_15_), 133.2 (C_2_ or C_18_), 132.9 (C_2_ or C_18_), 130.1 (C_3_ or C_17_), 130.0 (C_3_ or C_17_), 127.7 (C_4_ or C_16_), 127.6 (C_4_ or C_16_), 57.8 (C_7_), 55.0 (C_9_), 45.5 (C_6_), 44.1 (C_8_), 43.5 (C_12_ or C_13_), 43.2 (C_12_ or C_13_), 34.5 (C_11_), 29.8 (C_14_), 21.7 (C_1_ + C_19_). Mp: 70.0–73.1 °C. IR: ν_max_ 2923, 2847, 1720, 1597, 1340, 1327, 1157; cm^–1^. HRMS: *m*/*z* calculated for C_23_H_29_N_2_O_5_S_2_^+^, 477.1512 [M + H]^+^. Found *m*/*z* 477.1520, Δ = 1.7 ppm. Rf (30% EtOAc in Pet ether) = 0.16.
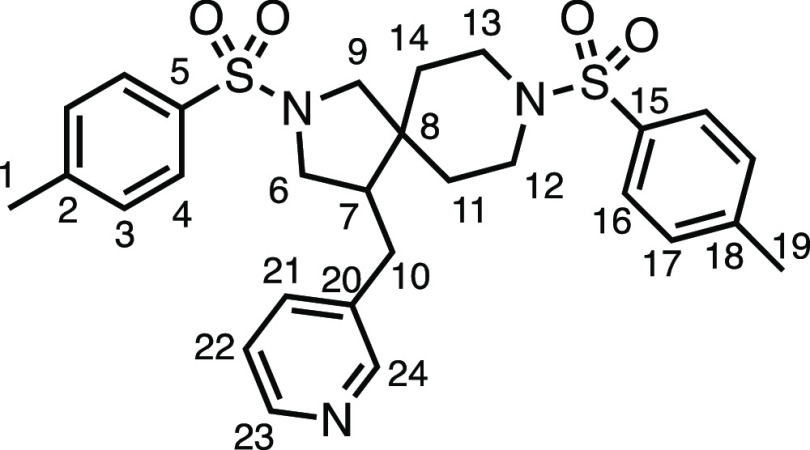


#### 4-(Pyridin-3-ylmethyl)-2,8-ditosyl-2,8-diazaspiro[4.5]decane (**6**)

The compound was prepared following the general procedure as disclosed by MacMillan.^12^ To an 8 mL microwave vial equipped with a stir bar was added photocatalyst (Ir[dF(CF_3_)ppy]_2_(dtbbpy))PF_6_ (5.6 mg, 0.01 equiv), 4-(bromomethyl)-2,8-ditosyl-2,8-diazaspiro[4.5]decane (**3ad**) (406.1 mg, 0.75 mmol, 1.5 equiv), 3-bromopyridine (48 μL, 0.50 mmol, 1.0 equiv), tris(trimethylsilyl)silane (154 μL, 0.50 mmol, 1.0 equiv), and anhydrous sodium carbonate (106 mg, 1.0 mmol, 2.0 equiv). The vial was sealed and placed under nitrogen before 4 mL of 1,2-dimethoxyethane (DME) was added. To a separate vial were added NiCl_2_·glyme (1 mg, 5 μmol, 0.01 equiv) and 4,4_′_-di-*tert*-butyl-2,2_′_-bipyridine (1.3 mg, 5 μmol, 0.01 equiv). The catalyst vial was sealed, purged with nitrogen, and then to it was added 2 mL of DME. The precatalyst solution was sonicated for 5 min and 1 mL (0.5 mol % catalyst, 2.5 μmol, 0.005 equiv) was syringed into the reaction vessel. The solution was degassed by sparging with nitrogen while stirring for 10 min before sealing with Parafilm. The reaction was stirred and irradiated with a 470 nm LED with fan-cooling (see the SI for setup) for 20 h. The reaction was then concentrated *in vacuo* and purified by chromatography (50–60% EtOAc in Pet ether) to give *tris*-azaheterocycle **6** (86.4 mg, 32%). ^1^H NMR (400 MHz, CDCl_3_) δ 8.51–8.43 (m, 1H, H_23_), 8.29–8.23 (m, 1H, H_24_), 7.63 (app dd, *J* = 6.6, 1.6 Hz, 2H, H_4_ or H_16_), 7.58 (app dd, *J* = 6.6, 1.7 Hz, 2H, H_4_ or H_16_), 7.37 (d, *J* = 8.0 Hz, 2H, H_3_ or H_17_), 7.34 (dt, *J* = 7.8, 1.9 Hz, 1H, H_21_), 7.29 (d, *J* = 7.9 Hz, 2H, H_3_ or H_17_), 7.21 (dd, *J* = 7.7, 4.8 Hz, 1H, H_22_), 3.65 (d, *J* = 11.7 Hz, 2H, H_12_ + H_13_), 3.31 (d, *J* = 10.2 Hz, 1H, H_9_), 3.17 (dd, *J* = 10.1, 7.6 Hz, 1H, H_6_), 2.92 (dd, *J* = 10.1, 8.6 Hz, 1H, H_6′_), 2.83 (d, *J* = 10.2 Hz, 1H, H_9′_), 2.70 (dd, *J* = 13.7, 3.4 Hz, 1H, H_10_), 2.49 (s, 3H, H_1_ or H_19_), 2.44 (s, 3H, H_1_ or H_19_), 2.27 (td, *J* = 12.1, 2.4 Hz, 1H, H_12′_), 2.19 (dtd, *J* = 14.8, 7.5, 7.0, 4.1 Hz, 2H, H_13′_ + H_10′_), 1.89 (dtd, *J* = 11.6, 8.2, 3.5 Hz, 1H, H_7_), 1.81 (td, *J* = 13.0, 4.5 Hz, 1H, H_14_), 1.65 (td, *J* = 12.6, 4.1 Hz, 1H, H_11_), 1.35–1.24 (m, 2H, H_11′_ + H_14′_). ^13^C{^1^H} NMR (126 MHz, CDCl_3_) δ 149.8 (C_24_), 148.2 (C_23_), 144.2 (C_5_ or C_15_), 144.0 (C_5_ or C_15_), 136.2 (C_21_), 134.8 (C_20_), 133.7 (d, *J* = 0.6 Hz, C_2_ or C_18_), 132.8 (d, *J* = 0.7 Hz, C_2_ or C_18_), 130.1 (C_3_ or C_17_), 129.9 (C_3_ or C_17_), 127.7 (C_4_ or C_16_), 127.4 (C_4_ or C_16_), 123.8 (C_22_), 54.9 (C_9_), 50.6 (C_6_), 49.3 (C_7_), 43.9 (C_13_), 43.1 (C_12_), 42.6 (C_8_), 33.7 (C_14_), 31.0 (C_10_), 27.8 (C_11_), 21.8 (C_1_ or C_19_), 21.7 (C_1_ or C_19_). Mp: 174.6–177.7 °C. IR: ν_max_ 2844, 1338, 1326, 1157 cm^–1^. HRMS: *m*/*z* calculated for C_28_H_34_N_3_O_4_S_2_^+^, 539.1985 [M + H]^+^. Found *m*/*z* 539.1997, Δ = 2.2 ppm. Rf (50% EtOAc in Pet ether) = 0.09.

## Supporting Information Available

Details of experimental setup, NMR spectra, and X-ray crystallographic data (PDF)
