# Supplementary material for: Multicomponent Direct Assembly of N-Heterospirocycles Facilitated by Visible-Light-Driven Photocatalysis
Source: J Org Chem. 2022 Sep 14;87(19):13204–23. doi: 10.1021/acs.joc.2c01684 (PMC9552240; doi:10.1021/acs.joc.2c01684)
Supplement: Supplementary file 1 — jo2c01684_si_001.pdf [file jo2c01684_si_001.pdf]

Supporting Information

Multicomponent Direct Assembly of *N*-Heterospirocycles Facilitated by Visible-Light-Driven Photocatalysis

Oliver M. Griffiths[a] and Steven V. Ley [a]*

[a] *Yusuf Hamied Department of Chemistry, University of Cambridge, Cambridge CB2 1EW, United Kingdom*

E-mail: svl1000@cam.ac.uk

Table of contents

1. Reactor set-up S1
2. Scale-up experimental set-up and optimization S2
3. 1H, 13C{1H}, COSY, HSQC, eHSQC and HMBC NMR Spectra S3
4. Thermal ellipsoid plot/ORTEP diagram for 3aa S131
5. X-ray Crystallographic information S132

1. Reactor set-up

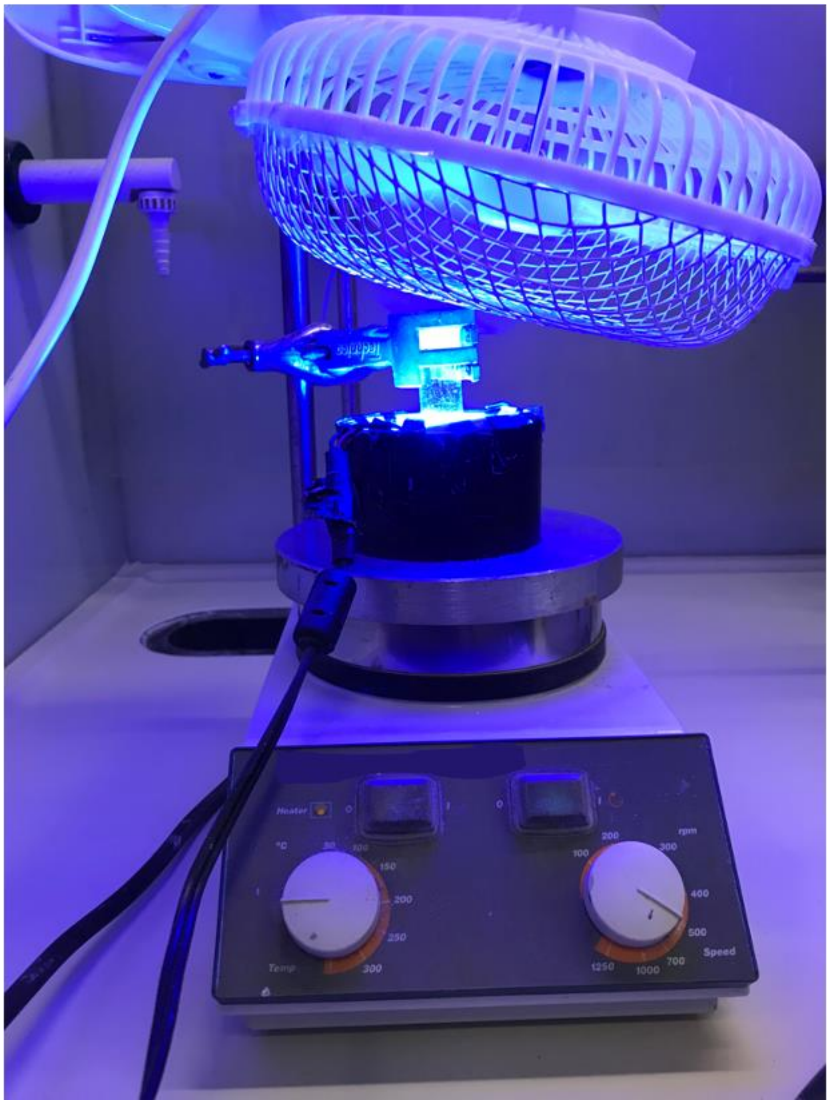

Figure S1. Reactor constitutes 470 nm LED strips (~10 W total power) with fan cooling from above.

2. Scale-up experimental set-up and optimization

Scale-up experiments were carried out using the setup shown in **Figure S2**, which was comprised of a Vapourtec E-series fitted with a UV-150 photoreactor with 61 W 365 nm LED and a 10 mL heated jacket coil reactor (kept at room temperature), stirrer hotplates and reagent/collection flasks.

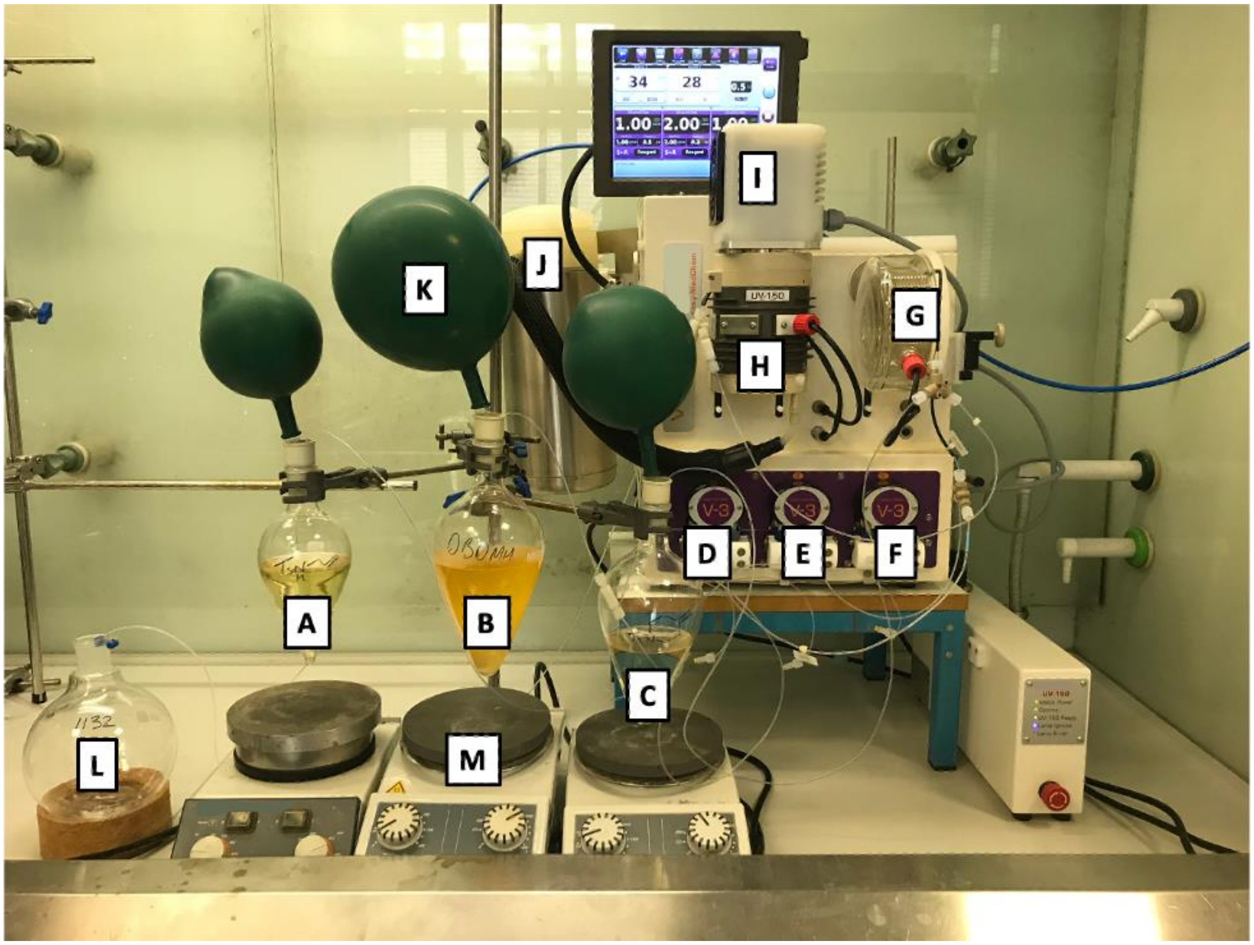

Figure S2. Flow system apparatus for reaction scale-up. **A**: *N*-allyltoluenesulfonamide in DCM (0.2 M); **B**: 1,3-dibromo-5,5'-dimethylhydantoin in DCM (0.2 M); **C**: 4-methylene-1-tosylpiperidine in DCM (0.6 M); **D**: peristaltic pump for solution **A**; **E**: peristaltic pump for solution **B**; **F**: peristaltic pump for solution **C**; **G**: 10 mL coil reactor; **H**: 10 mL coil in UV-150 photoreactor; **I**: 365 nm LED; **J**: cooling unit; **K**: nitrogen balloons; **L**: reaction output collection (1 L round-bottomed flask); **M**: stirrer hotplates.

3. ^{1}H, ^{13}C{^{1}H}, COSY, HSQC, eHSQC and HMBC NMR Spectra

Methyl 4-(*N*-(but-3-en-1-yl)sulfamoyl)benzoate (1c)

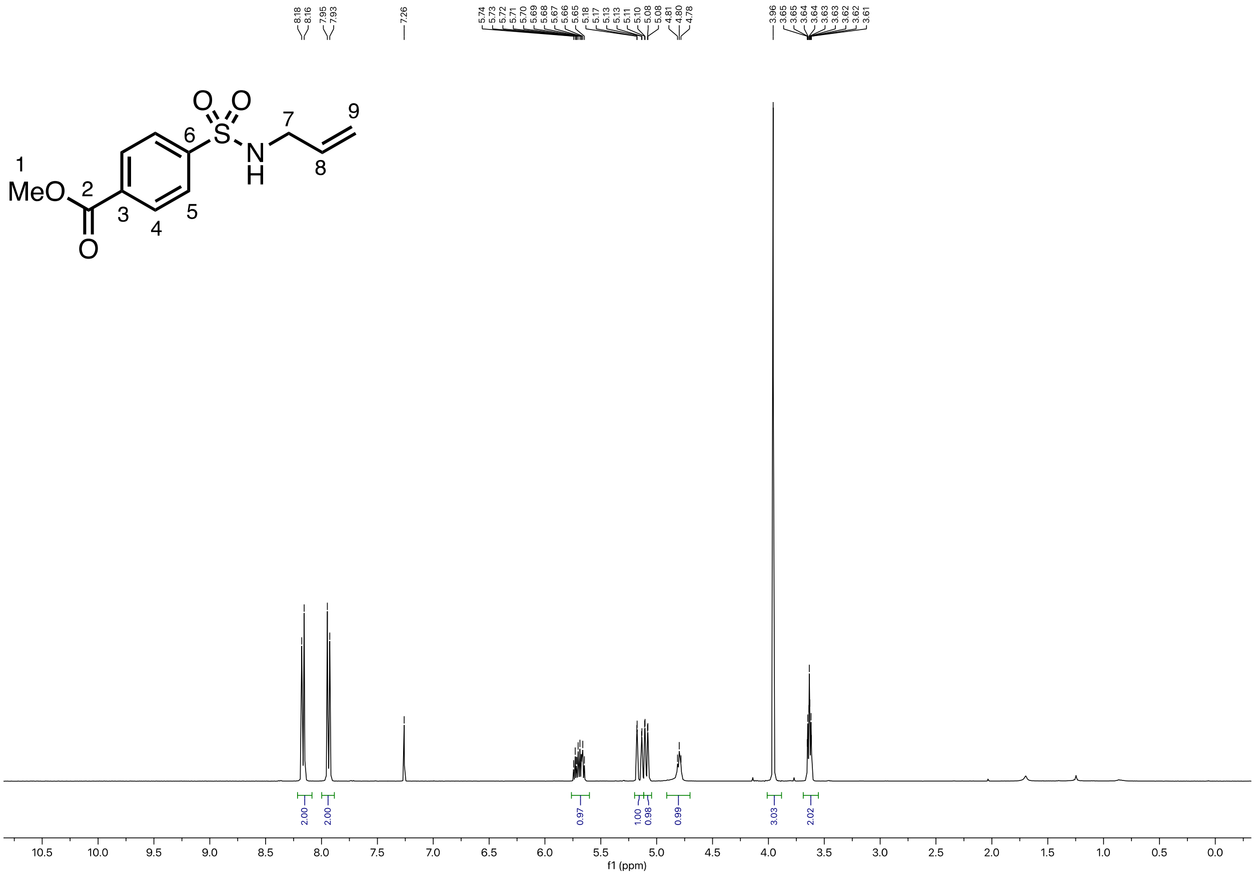

^{1}H NMR of *Methyl 4-(N-(but-3-en-1-yl)sulfamoyl)benzoate* (400 MHz, $CDCl_3$).

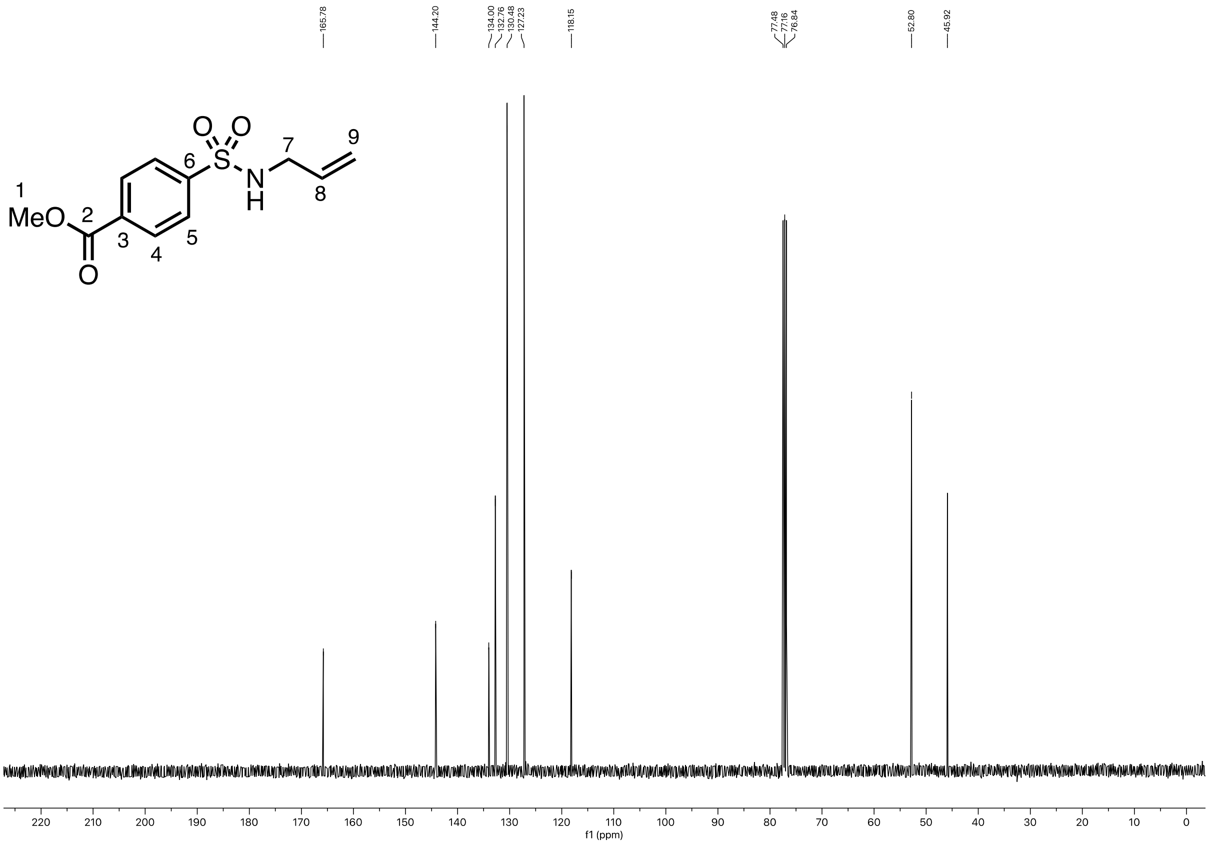

$^{13}C\{^{1}H\}$ NMR of *Methyl 4-(N-(but-3-en-1-yl)sulfamoyl)benzoate* (101 MHz, $CDCl_3$).

N-allyl-4-(methylsulfonyl)benzenesulfonamide (1g)

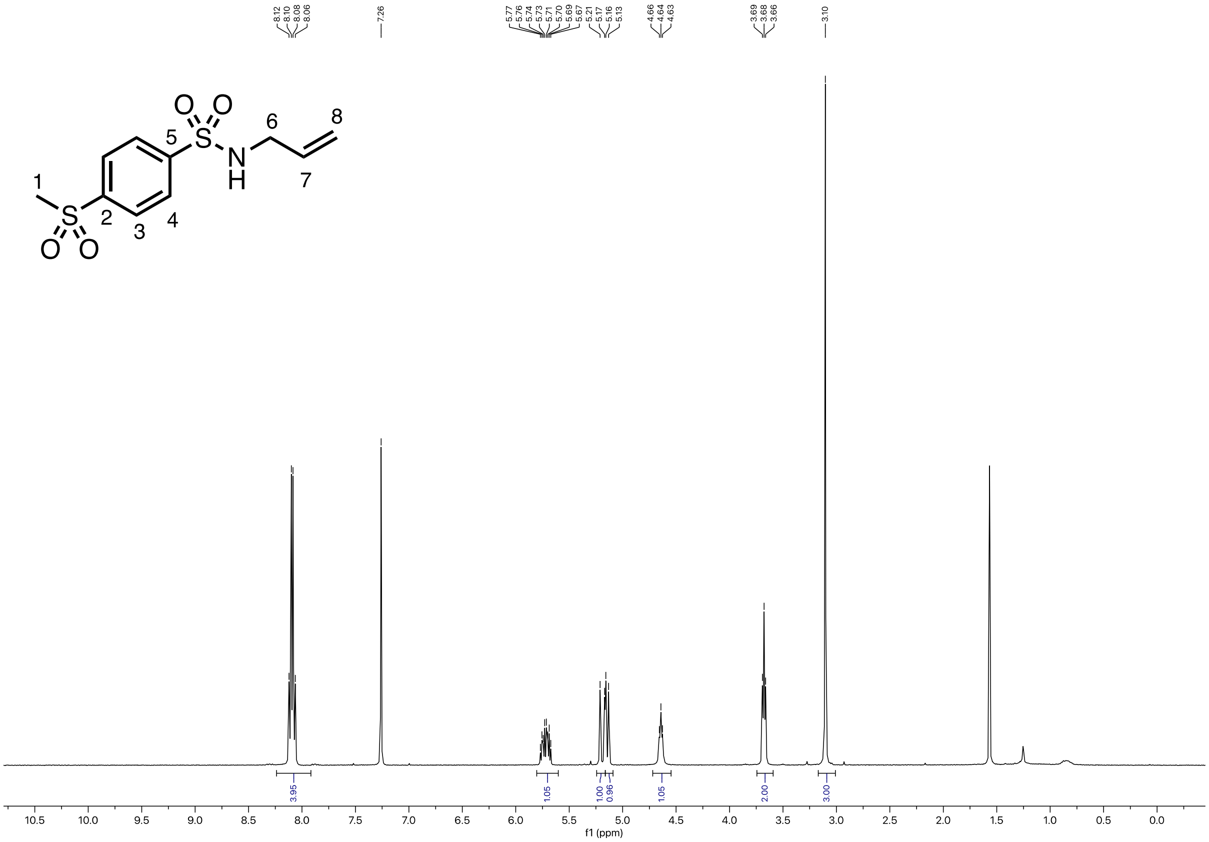

^{1}H NMR of *N-allyl-4-(methylsulfonyl)benzenesulfonamide* (400 MHz, $CDCl_3$).

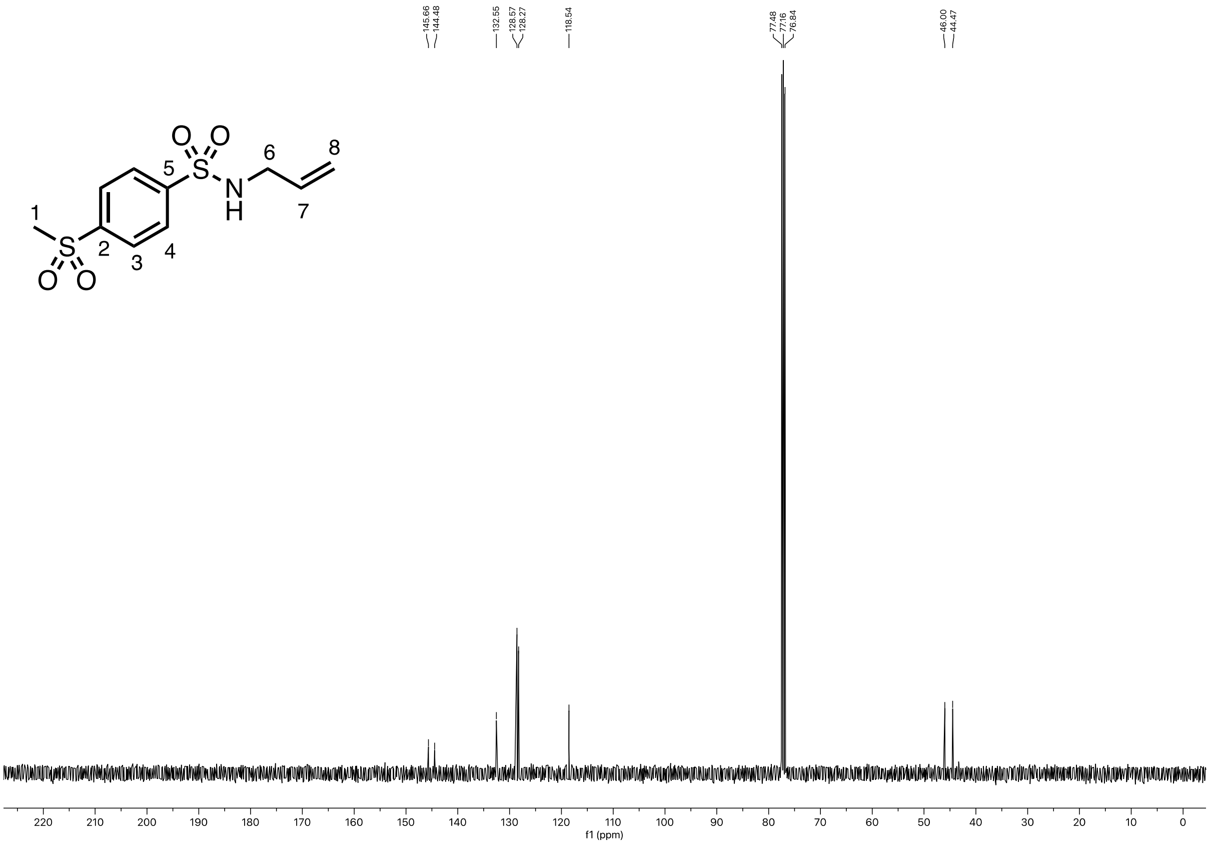

$^{13}C\{^1H\}$ NMR of *N-allyl-4-(methylsulfonyl)benzenesulfonamide* (101 MHz, $CDCl_3$).

N-allyl-4-(5-methyl-3-phenylisoxazol-4-yl)benzenesulfonamide (1ak)

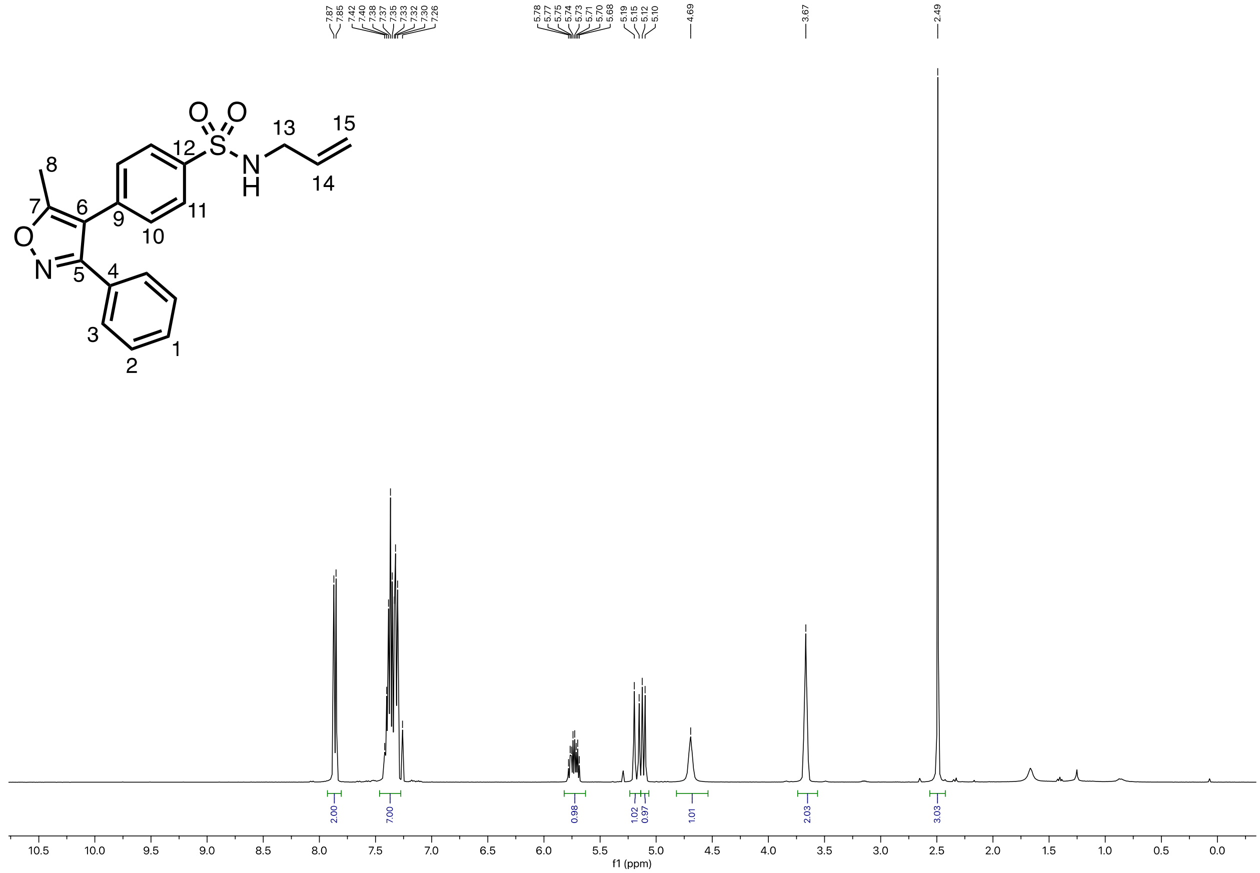

^1H NMR of *N-allyl-4-(5-methyl-3-phenylisoxazol-4-yl)benzenesulfonamide* (400 MHz, $CDCl_3$).

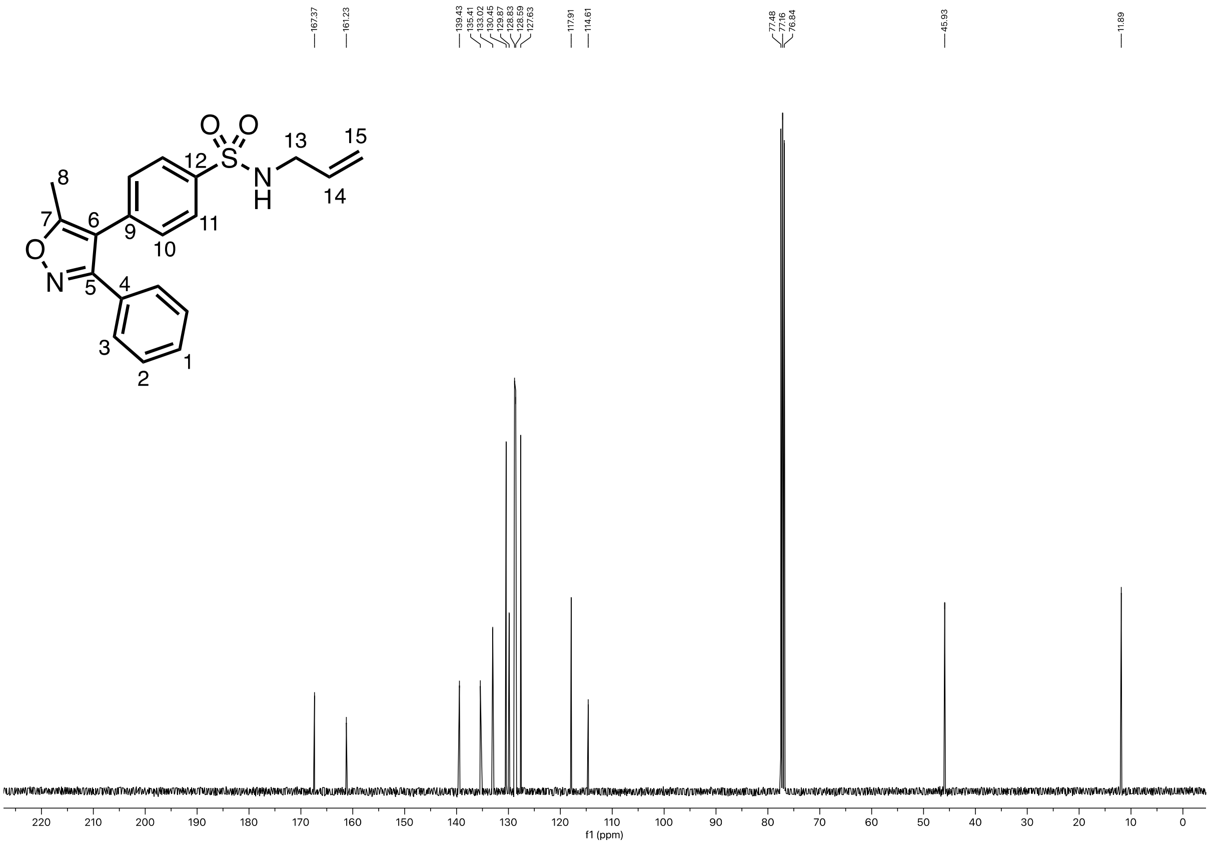

$^{13}C\{^{1}H\}$ NMR of *N-allyl-4-(5-methyl-3-phenylisoxazol-4-yl)benzenesulfonamide* (101 MHz, $CDCl_3$).

(E)-*N*-(5-(*N*-allylsulfamoyl)-3-methyl-1,3,4-thiadiazol-2(3H)-ylidene)acetamide (1al)

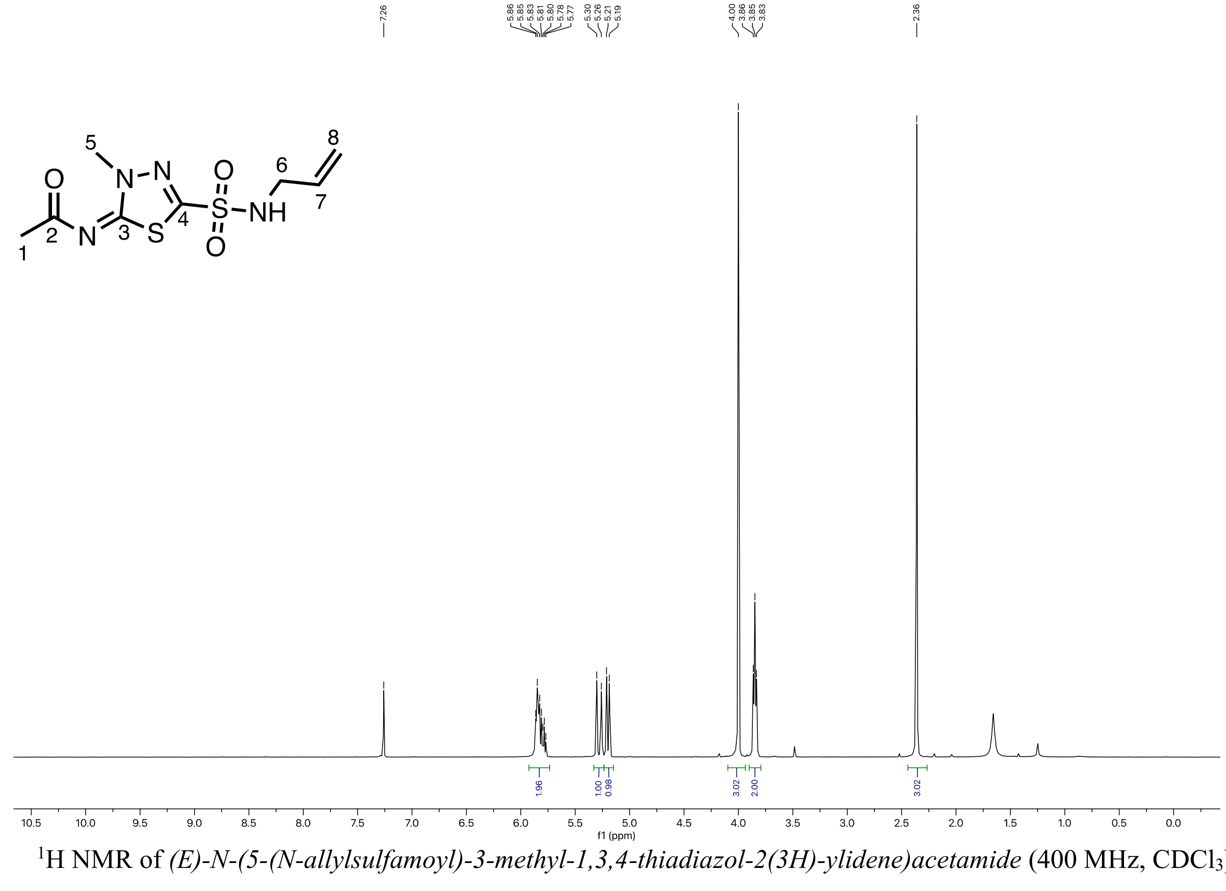

^{1}H NMR of *(E)-N-(5-(N-allylsulfamoyl)-3-methyl-1,3,4-thiadiazol-2(3H)-ylidene)acetamide* (400 MHz, $CDCl_3$).

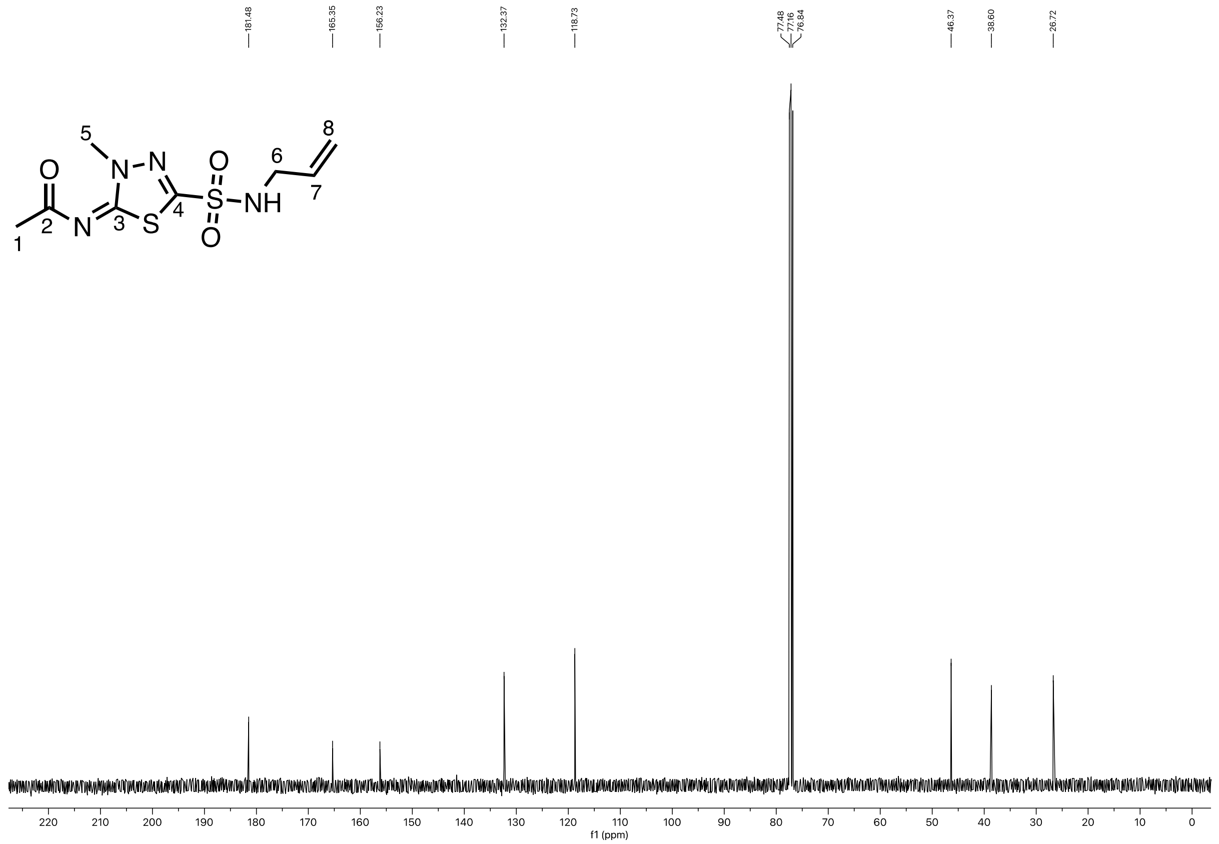

$^{13}C\{^{1}H\}$ NMR of *(E)-N-(5-(N-allylsulfamoyl)-3-methyl-1,3,4-thiadiazol-2(3H)-ylidene)acetamide* (101 MHz, $CDCl_3$).

(E)-*N*-allyl-6-ethoxybenzo[d]thiazole-2-sulfonamide (1am)

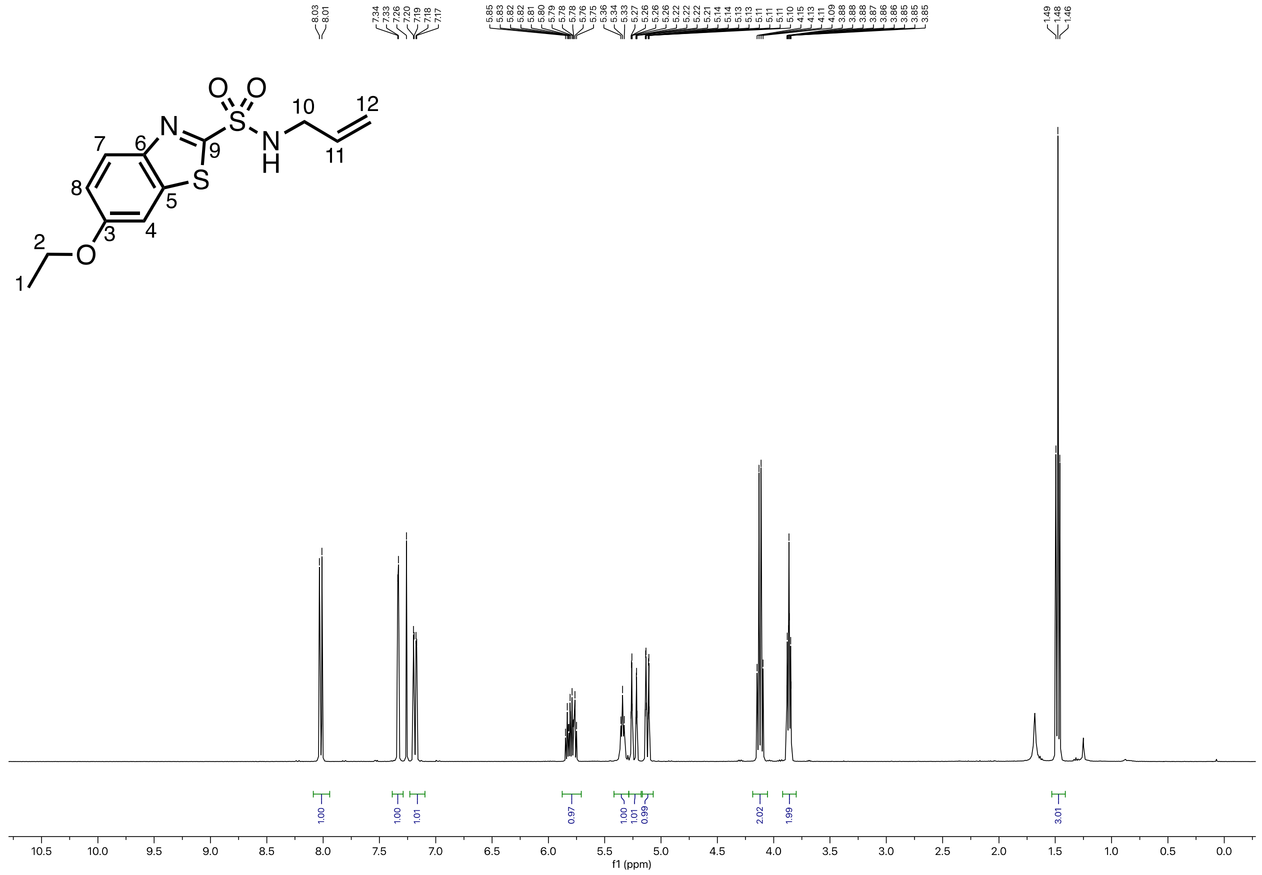

^{1}H NMR of *N-allyl-6-ethoxybenzo[d]thiazole-2-sulfonamide (1am)* (400 MHz, $CDCl_3$).

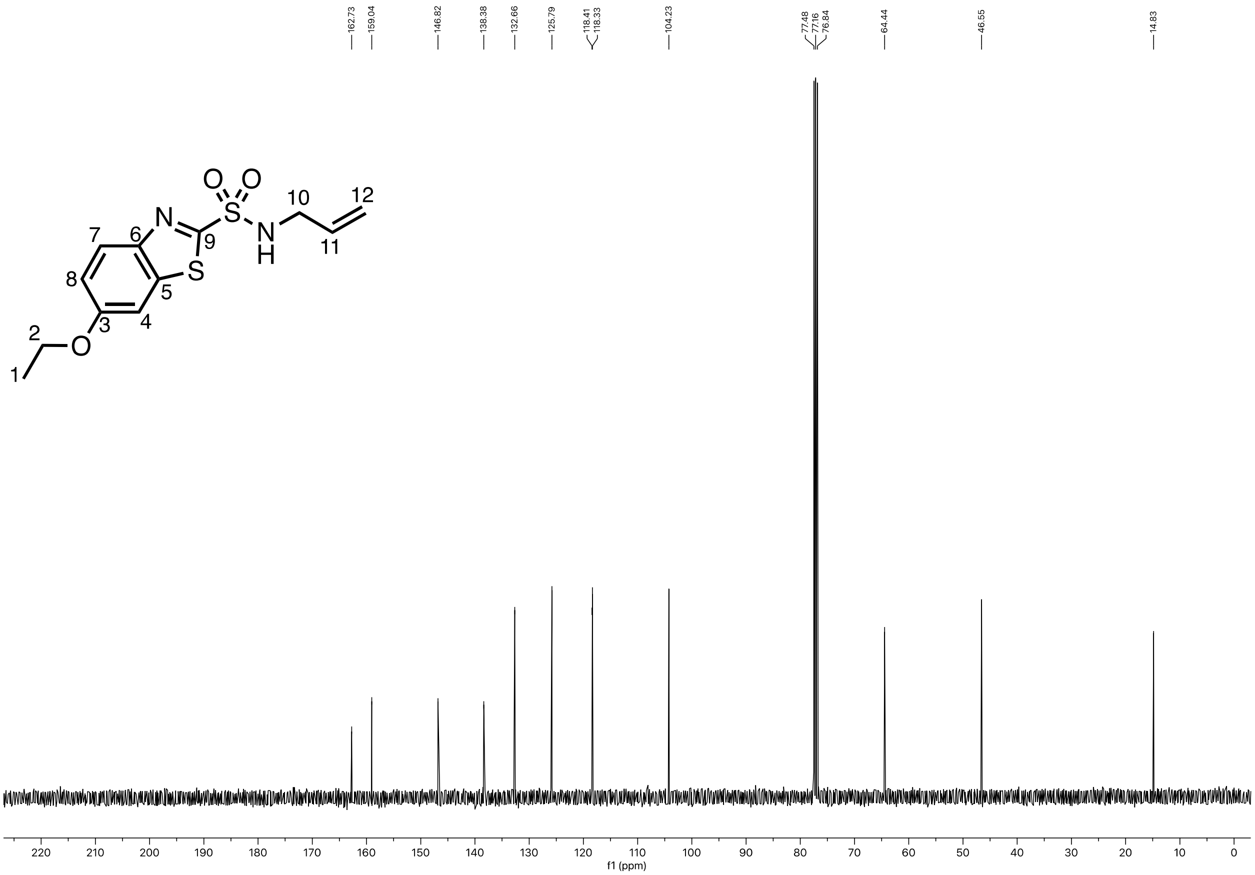

$^{13}C\{^{1}H\}$ NMR of *N-allyl-6-ethoxybenzo[d]thiazole-2-sulfonamide (1am)* (101 MHz, $CDCl_3$).

1-methyl-3-methylenecyclopentadecane (2v)

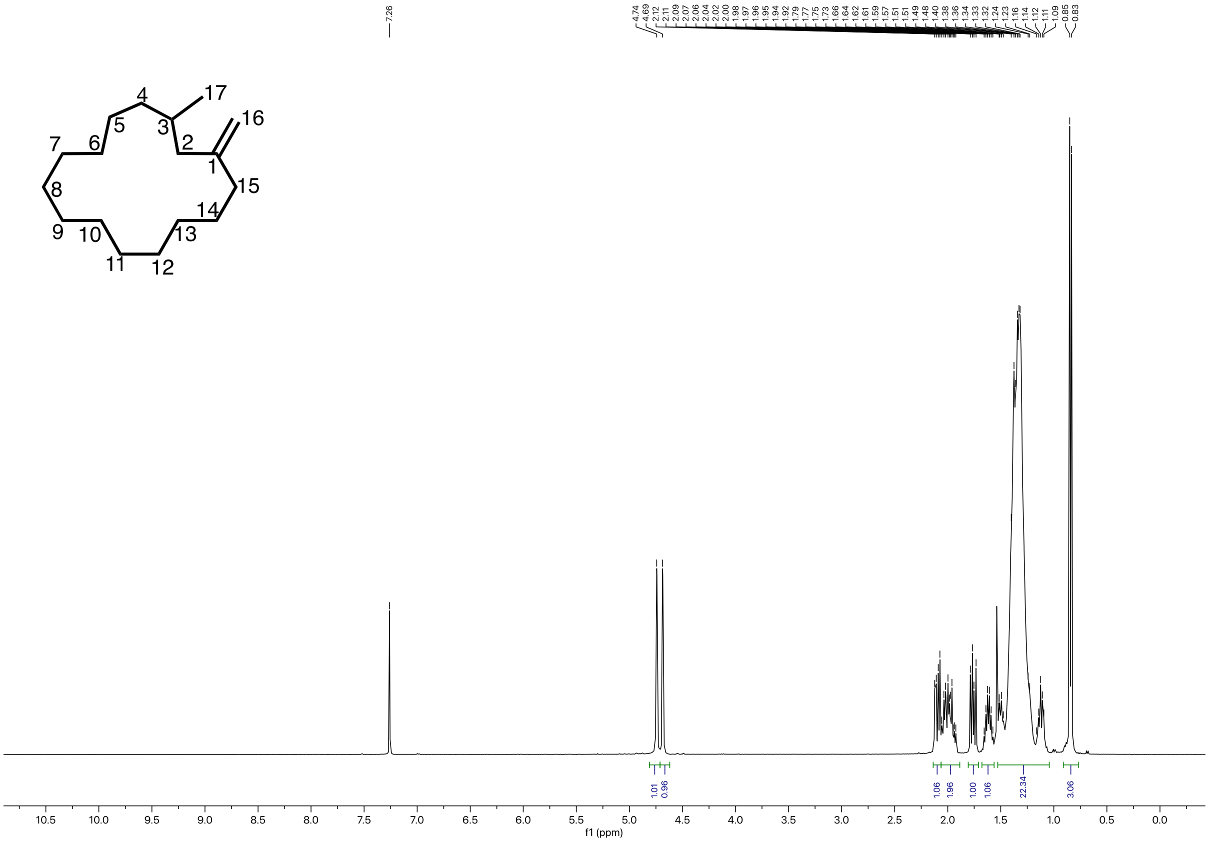

^{1}H NMR of *1-methyl-3-methylenecyclopentadecane* (400 MHz, $CDCl_3$).

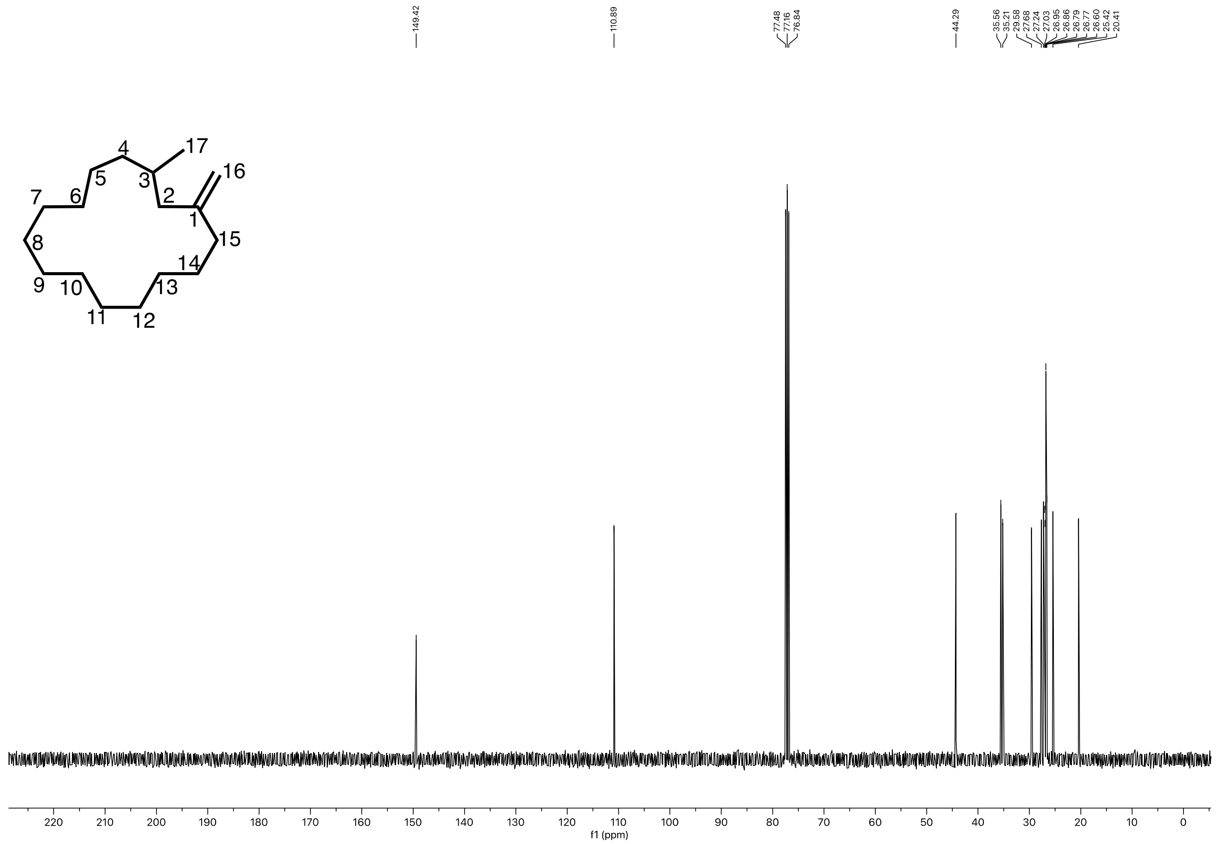

$^{13}C\{^1H\}$ NMR of *1-methyl-3-methylenecyclopentadecane* (101 MHz, $CDCl_3$).

7-methylene-2-oxaspiro[3.5]nonane (2w)

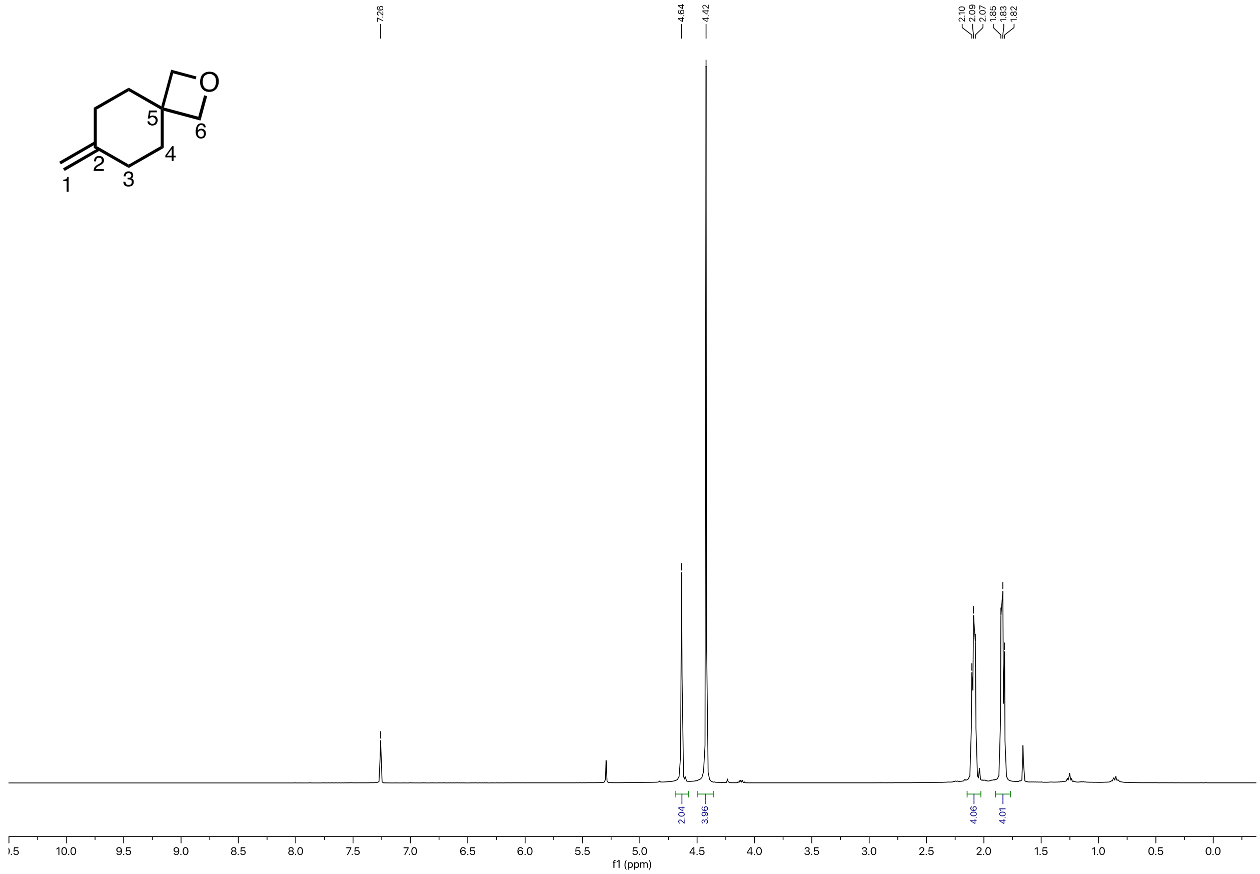

^1H NMR of *7-methylene-2-oxaspiro[3.5]nonane* (400 MHz, $CDCl_3$).

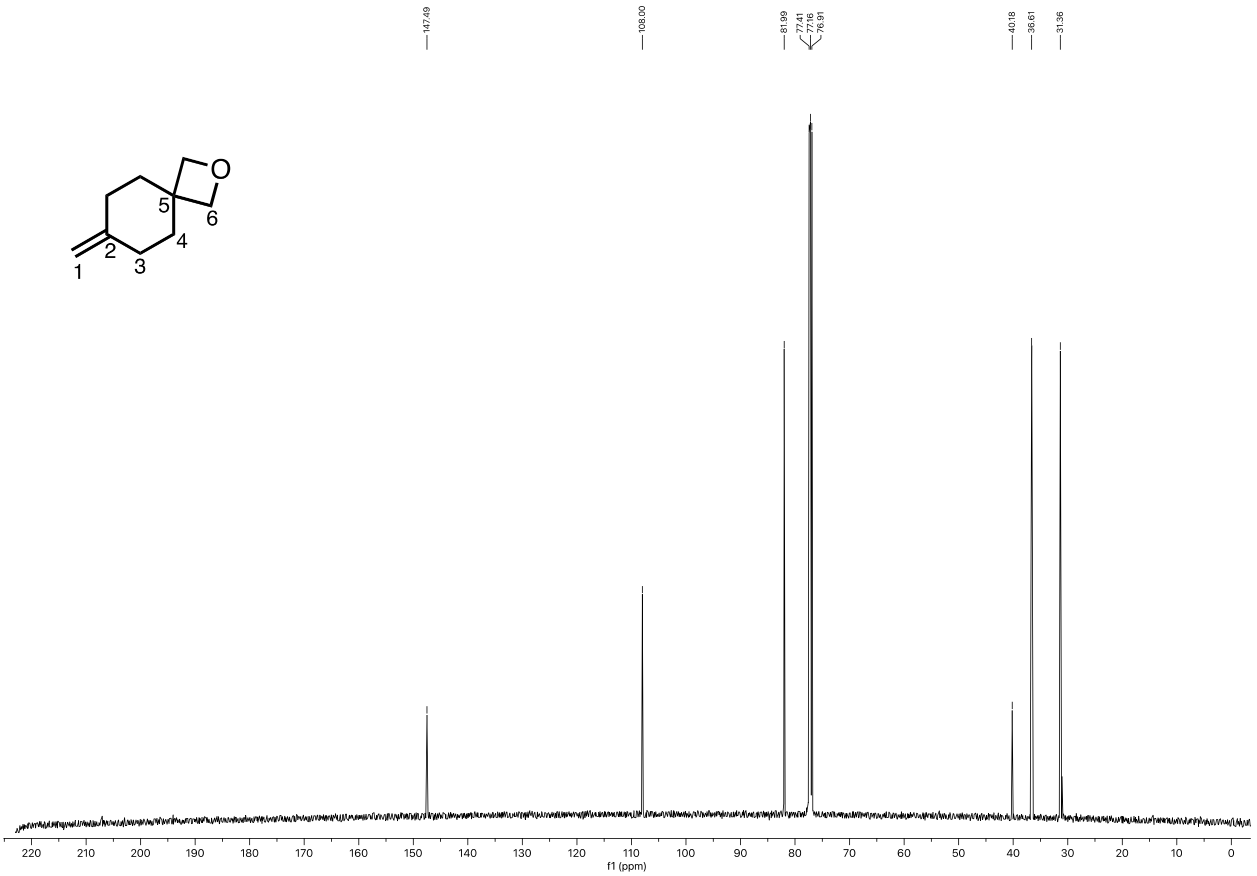

$^{13}C\{^{1}H\}$ NMR of *7-methylene-2-oxaspiro[3.5]nonane* (126 MHz, $CDCl_3$).

3-methylene-1-((4-nitrophenyl)sulfonyl)pyrrolidine (2ab)

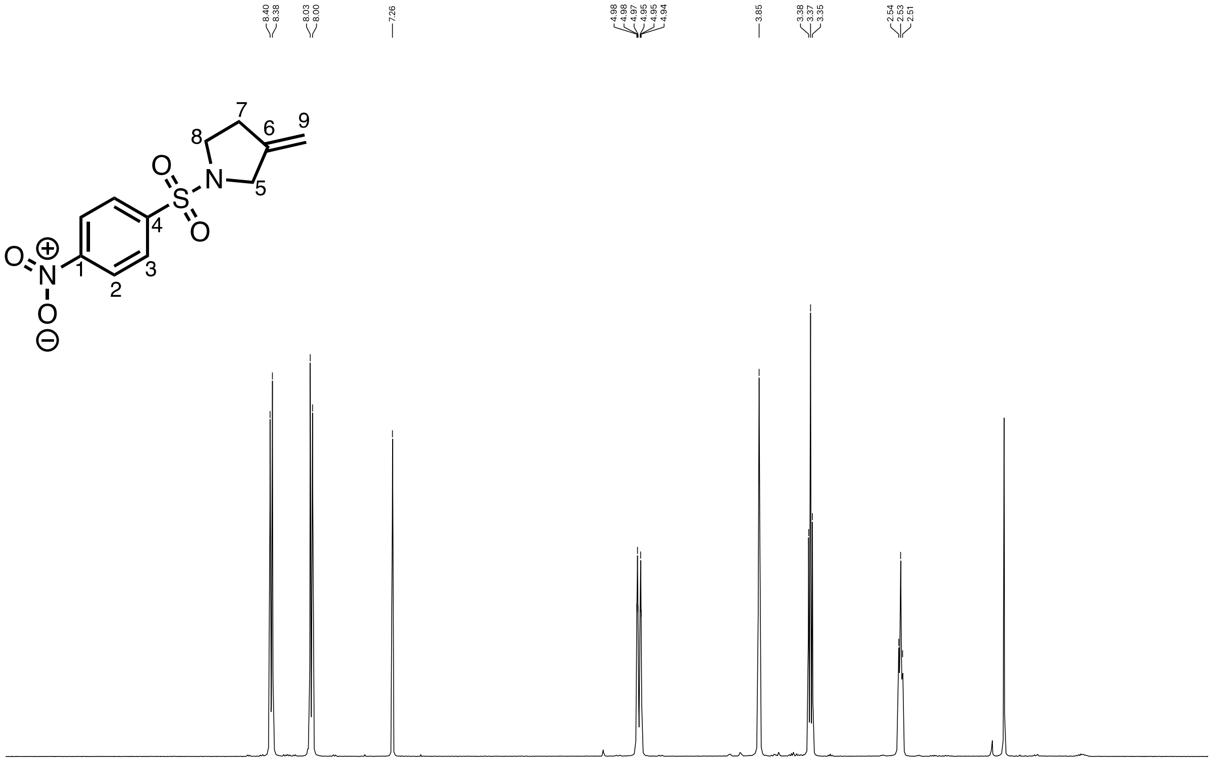

^{1}H NMR of *3-methylene-1-((4-nitrophenyl)sulfonyl)pyrrolidine* (400 MHz, $CDCl_3$).

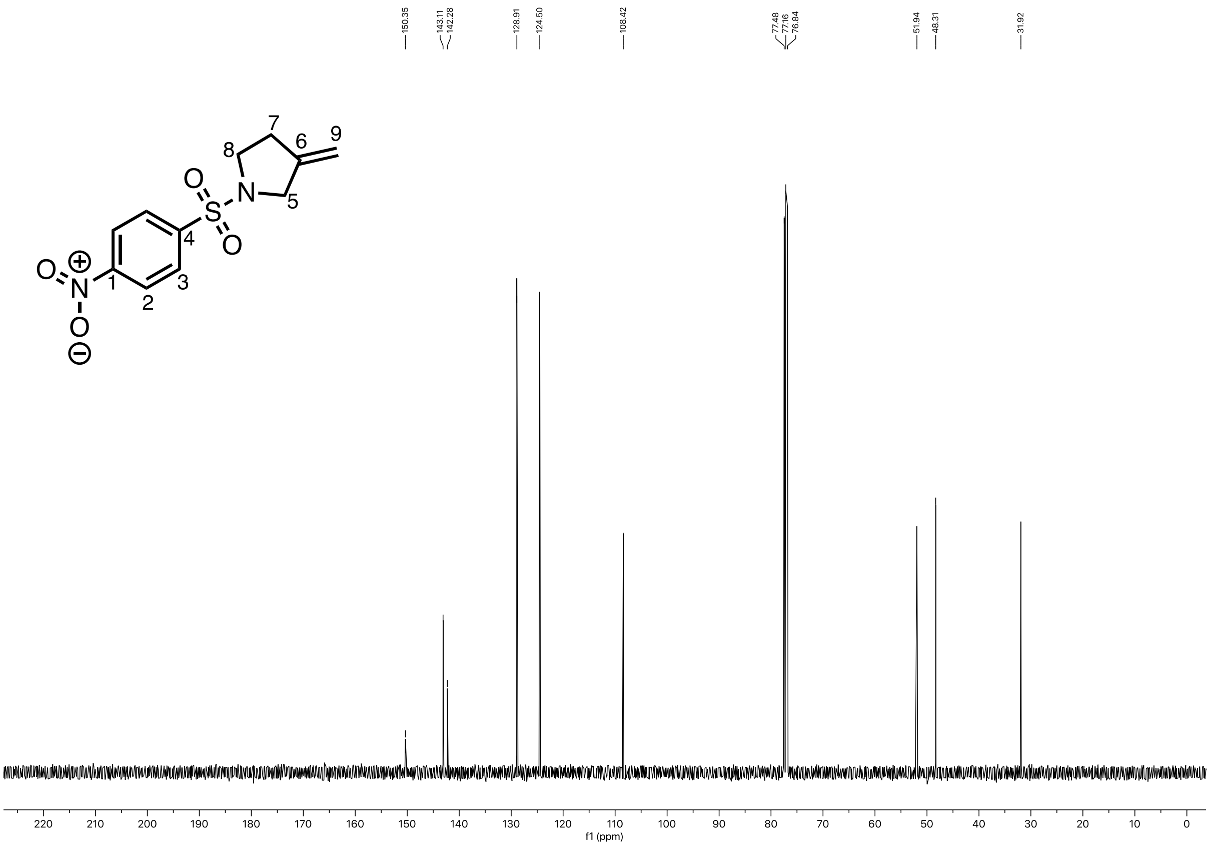

$^{13}C\{^{1}H\}$ NMR of *3-methylene-1-((4-nitrophenyl)sulfonyl)pyrrolidine* (101 MHz, $CDCl_3$).

2-methylene-7-tosyl-7-azaspiro[3.5]nonane (2ag)

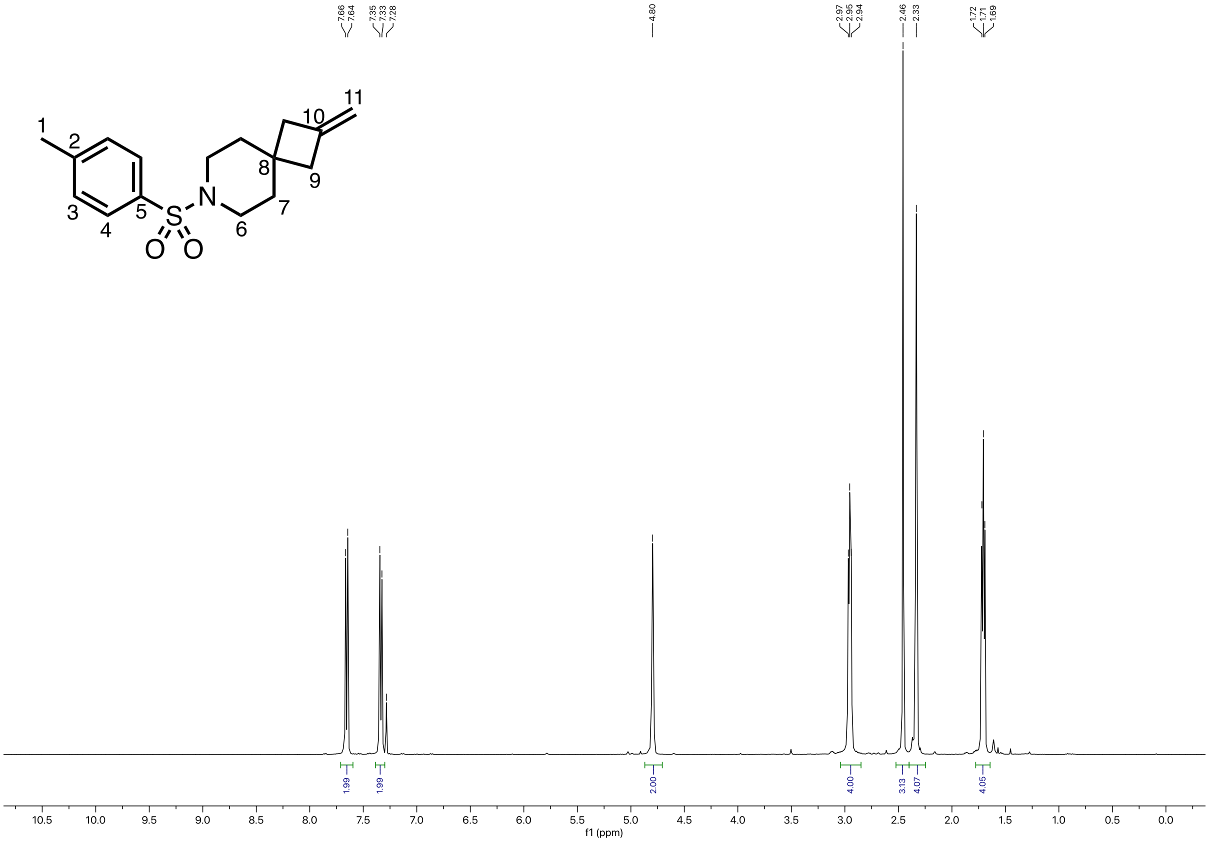

^{1}H NMR of *2-methylene-7-tosyl-7-azaspiro[3.5]nonane* (400 MHz, $CDCl_3$).

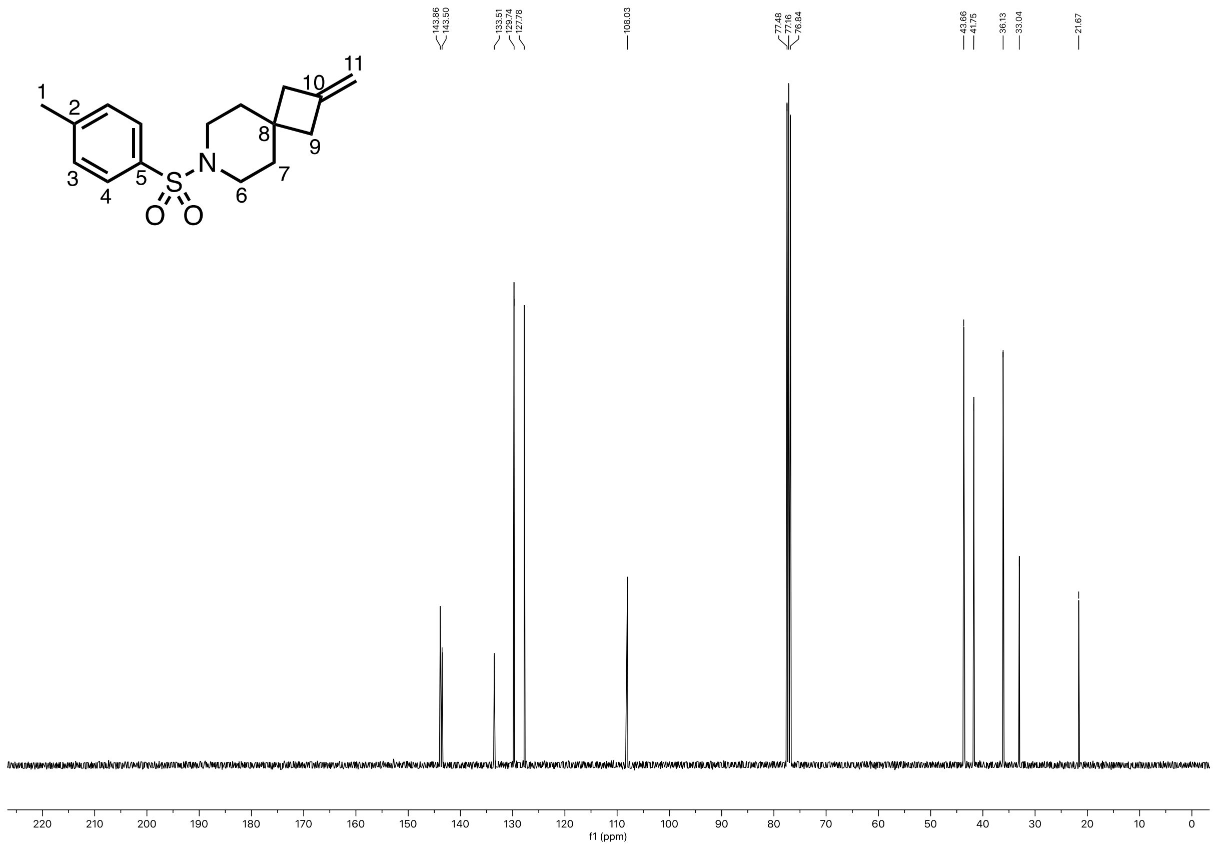

$^{13}C\{^1H\}$ NMR of *2-methylene-7-tosyl-7-azaspiro[3.5]nonane* (101 MHz, $CDCl_3$).

4-(chloromethyl)-2-tosyl-2-azaspiro[4.5]decane (3a)

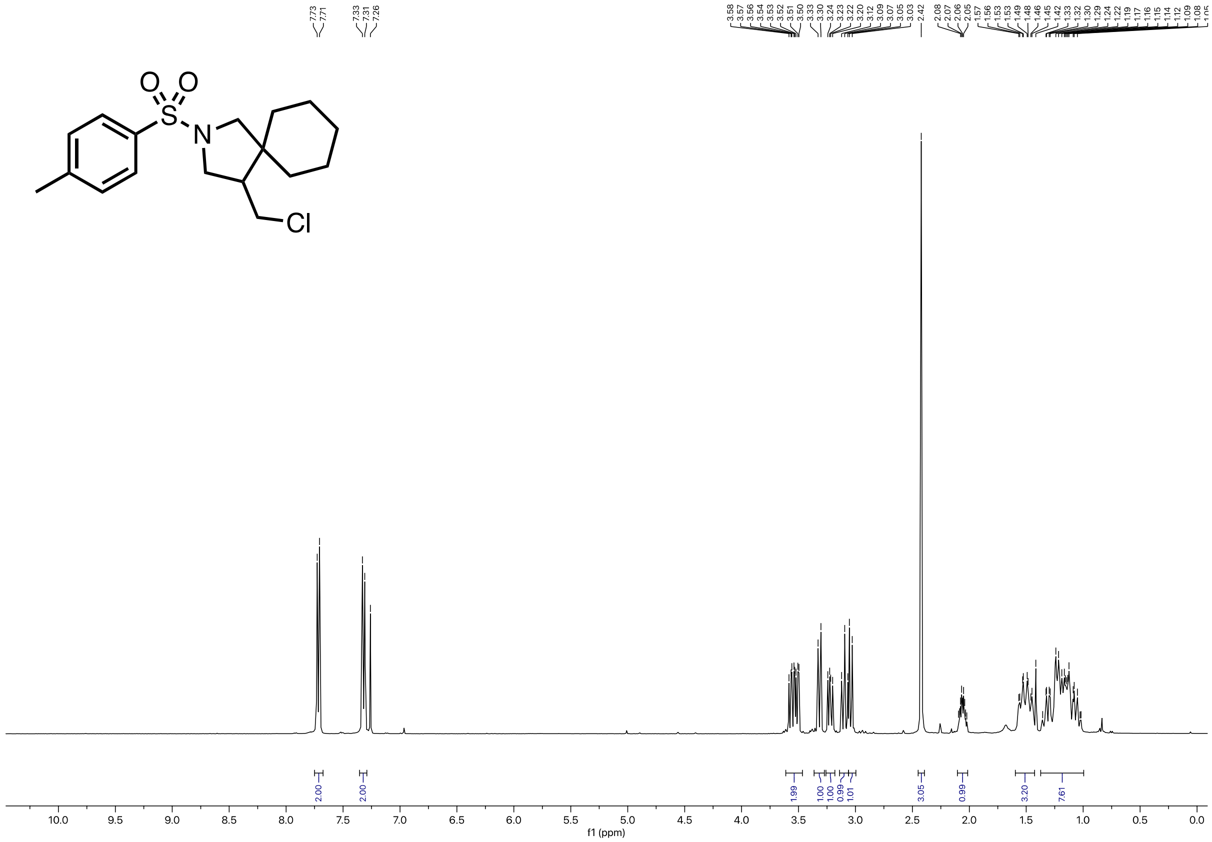

^1H NMR of *4-(chloromethyl)-2-tosyl-2-azaspiro[4.5]decane* (400 MHz, $CDCl_3$).

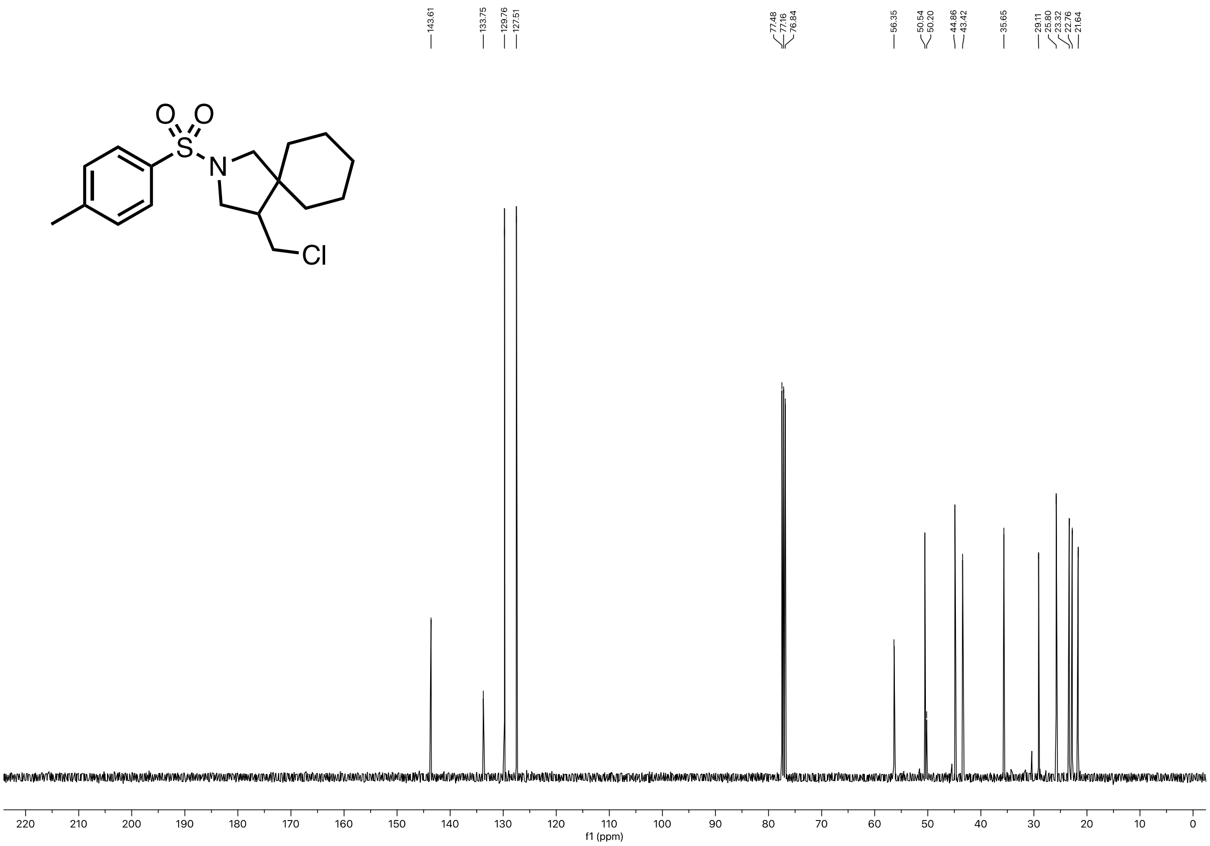

$^{13}C\{^1H\}$ NMR of *4-(chloromethyl)-2-tosyl-2-azaspiro[4.5]decane* (101 MHz, $CDCl_3$).

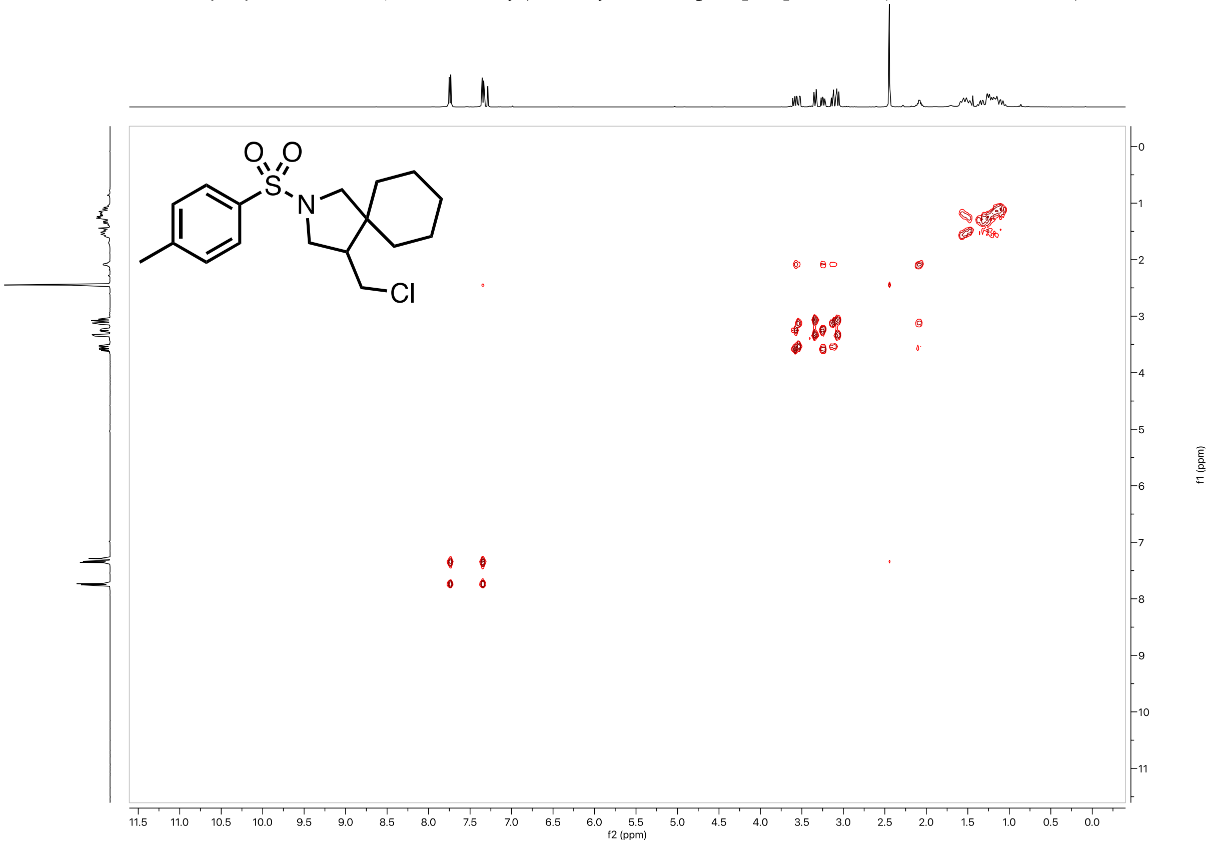

COSY of *4-(chloromethyl)-2-tosyl-2-azaspiro[4.5]decane* ($CDCl_3$).

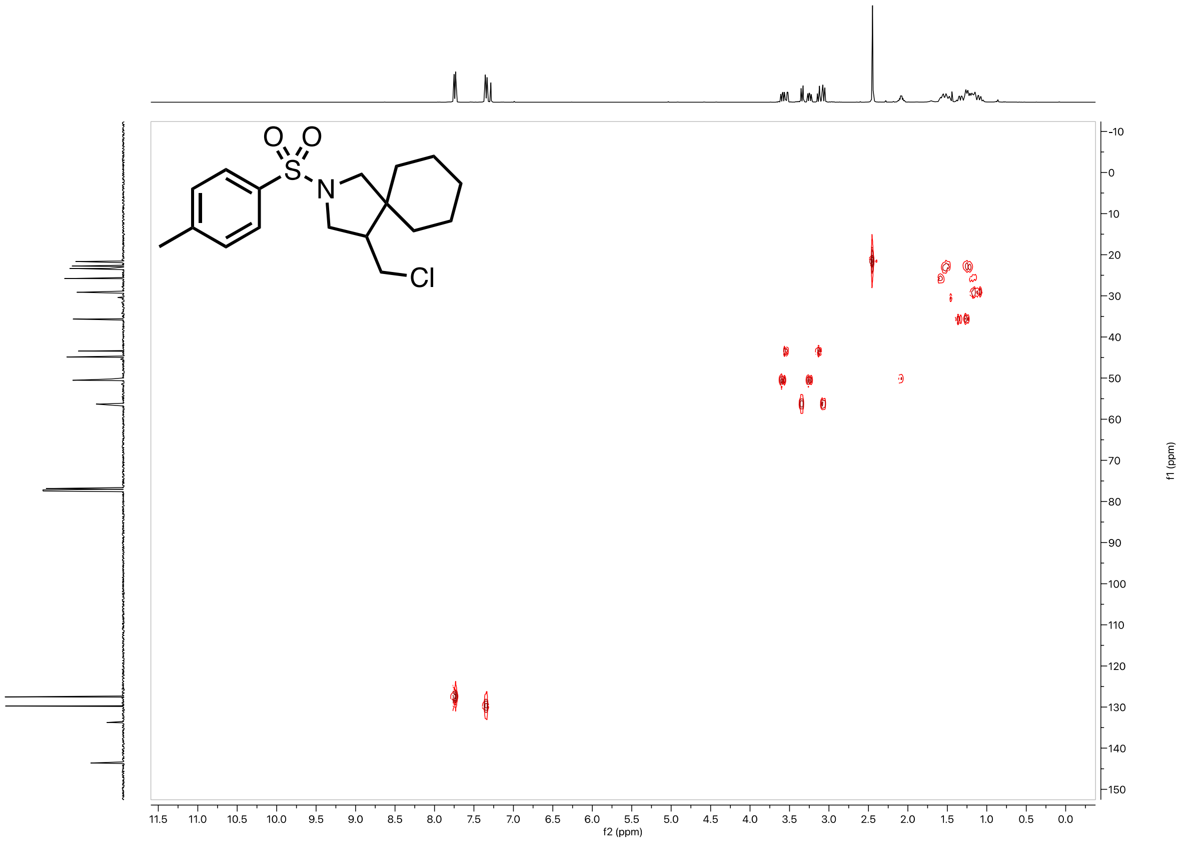

HSQC of *4-(chloromethyl)-2-tosyl-2-azaspiro[4.5]decane* ($CDCl_3$).

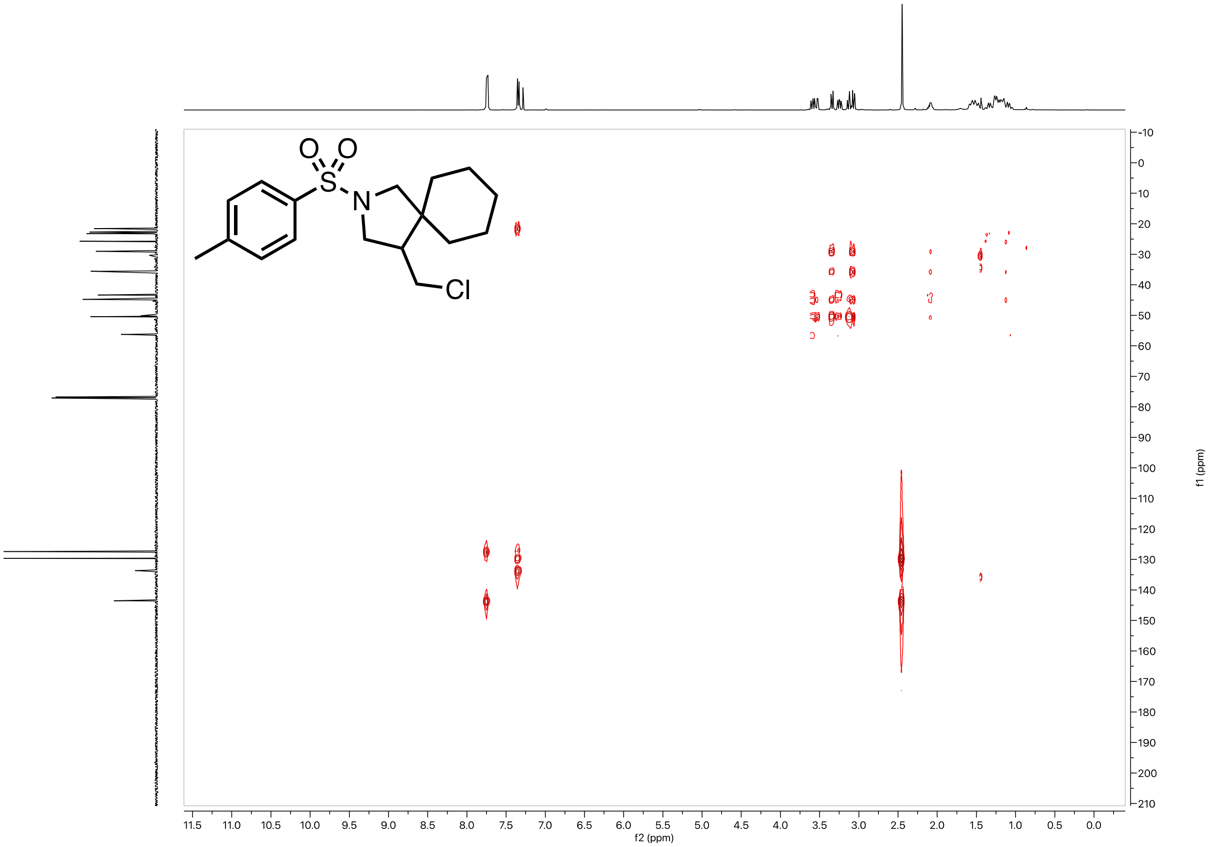

HMBC of *4-(chloromethyl)-2-tosyl-2-azaspiro[4.5]decane* ($CDCl_3$).

2-((4-bromophenyl)sulfonyl)-4-(chloromethyl)-2-azaspiro[4.5]decane (3b)

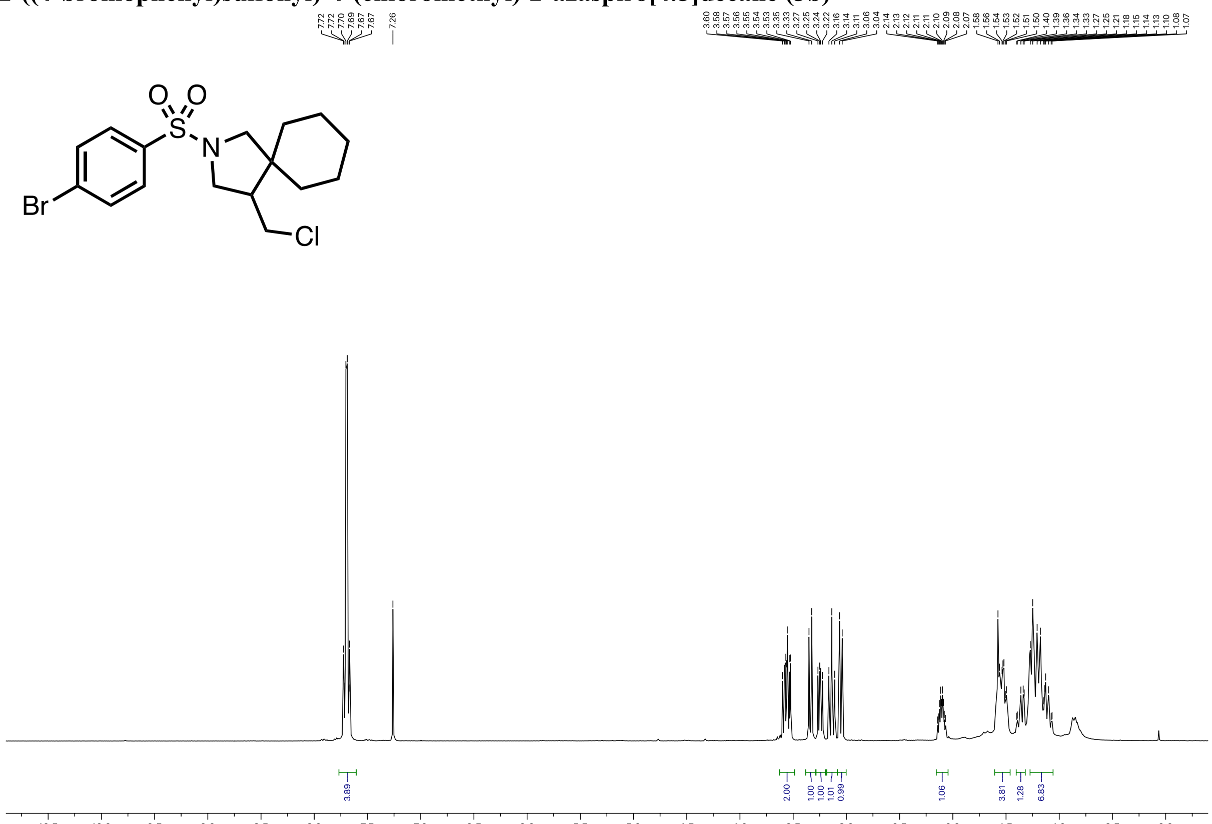

^{1}H NMR of *2-((4-bromophenyl)sulfonyl)-4-(chloromethyl)-2-azaspiro[4.5]decane* (400 MHz, $CDCl_3$).

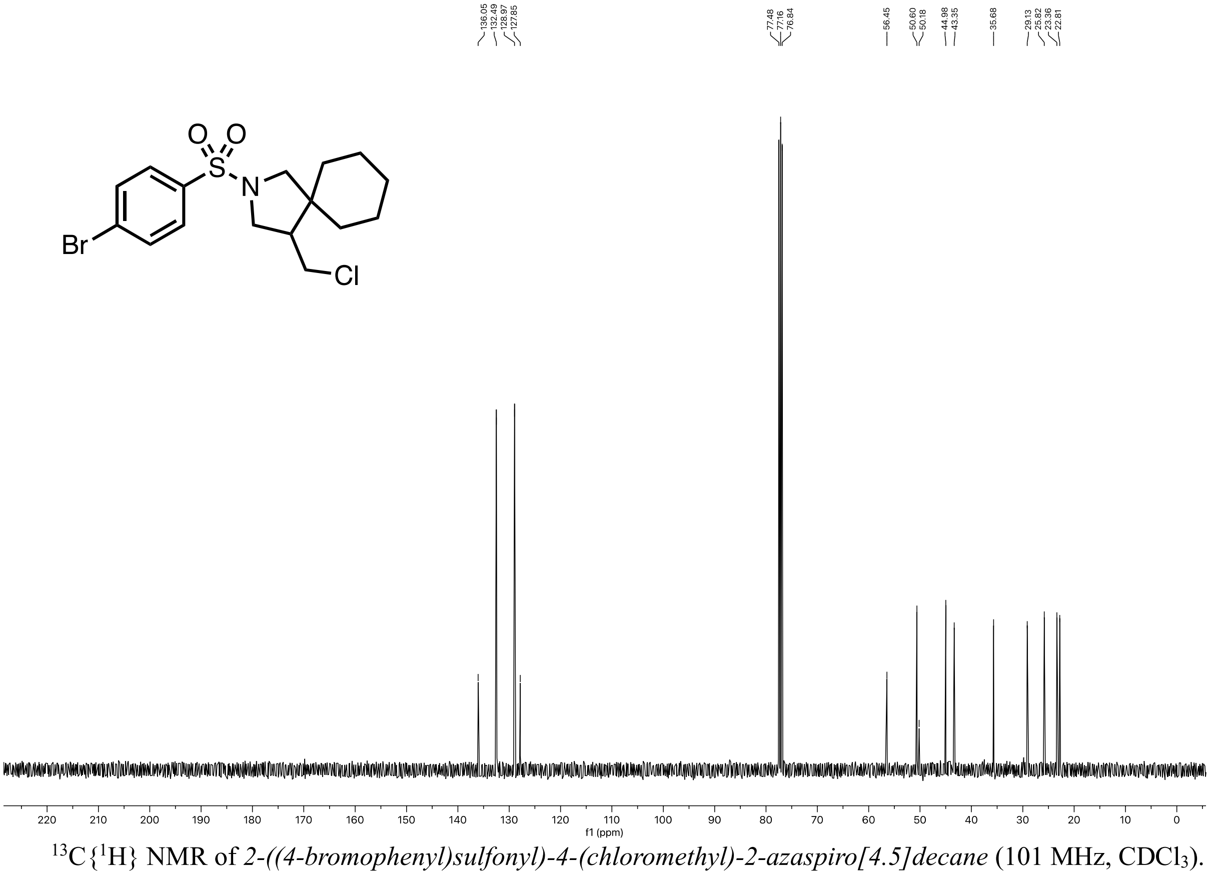

^{13}C{^{1}H} NMR of *2-((4-bromophenyl)sulfonyl)-4-(chloromethyl)-2-azaspiro[4.5]decane* (101 MHz, $CDCl_3$).

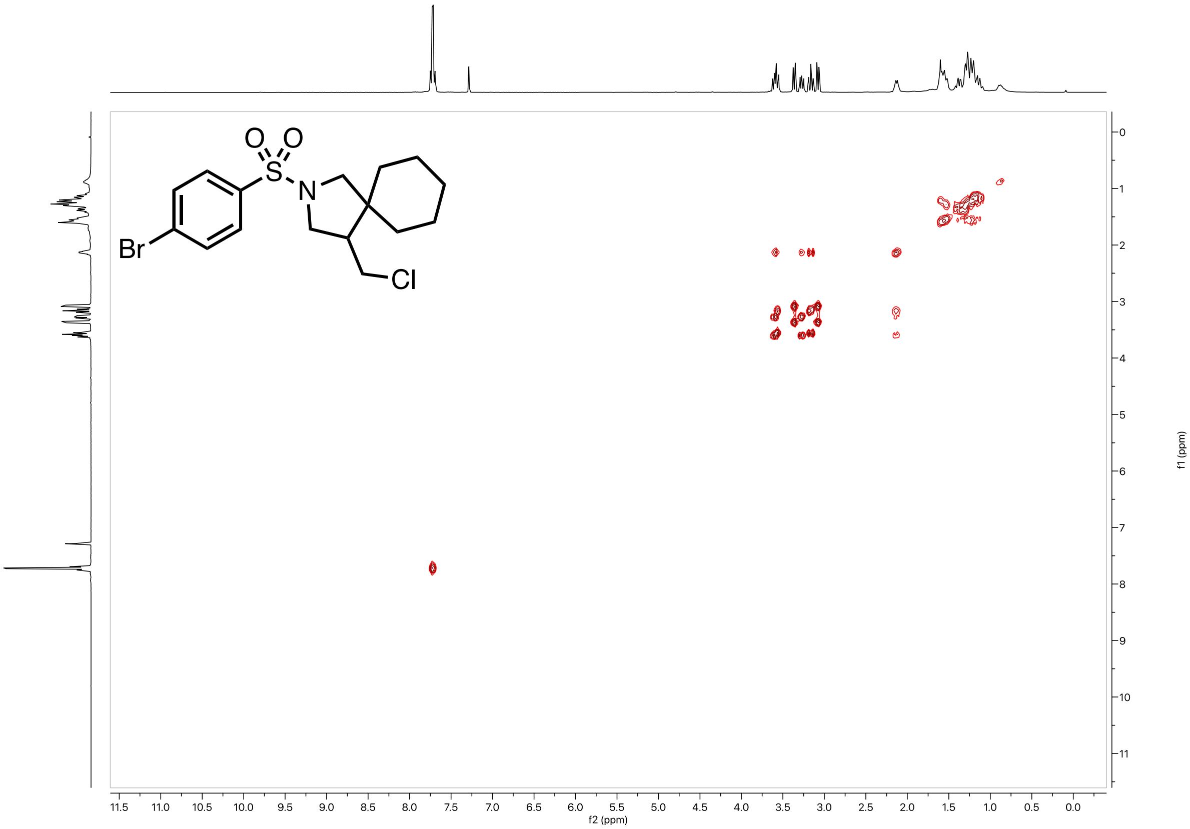

COSY of *2-((4-bromophenyl)sulfonyl)-4-(chloromethyl)-2-azaspiro[4.5]decane* ($CDCl_3$).

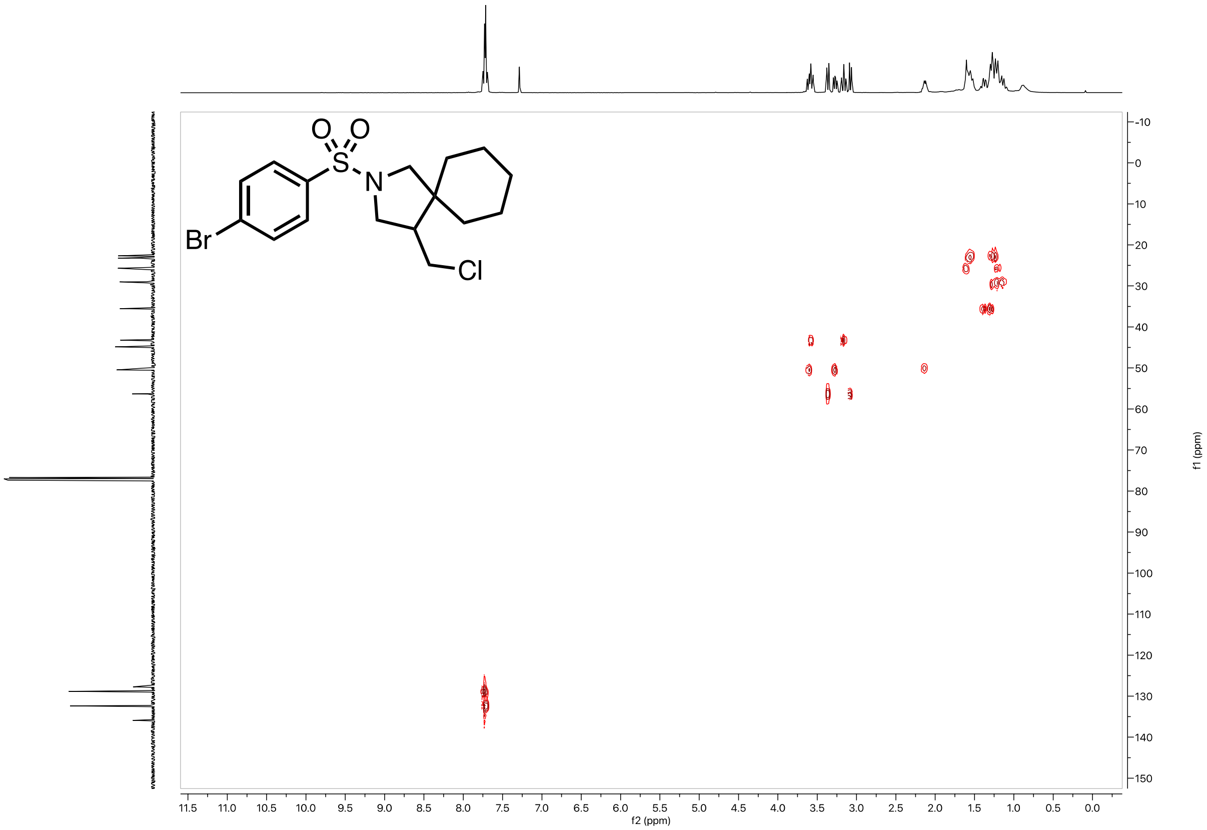

HSQC of *2-((4-bromophenyl)sulfonyl)-4-(chloromethyl)-2-azaspiro[4.5]decane* ($CDCl_3$).

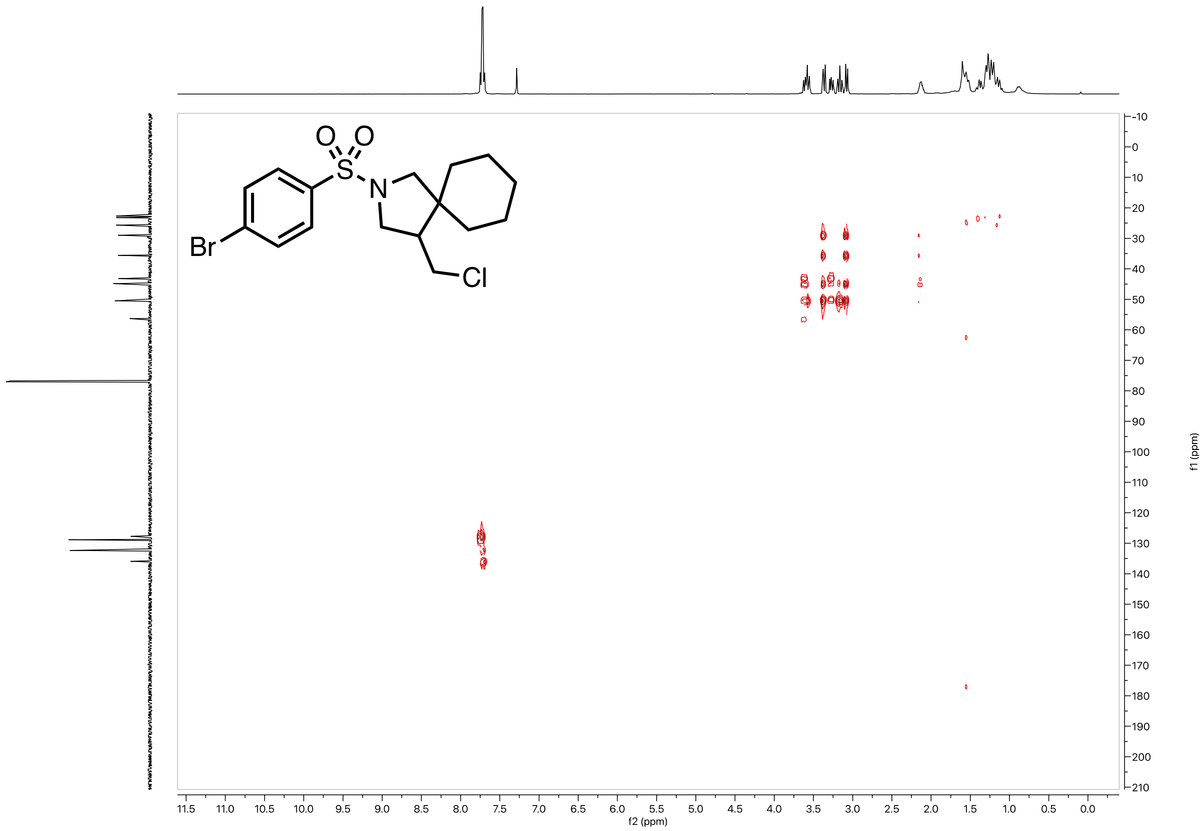

HMBC of *2-((4-bromophenyl)sulfonyl)-4-(chloromethyl)-2-azaspiro[4.5]decane* ($CDCl_3$).

Methyl 4-((4-(chloromethyl)-2-azaspiro[4.5]decan-2-yl)sulfonyl)benzoate (3c)

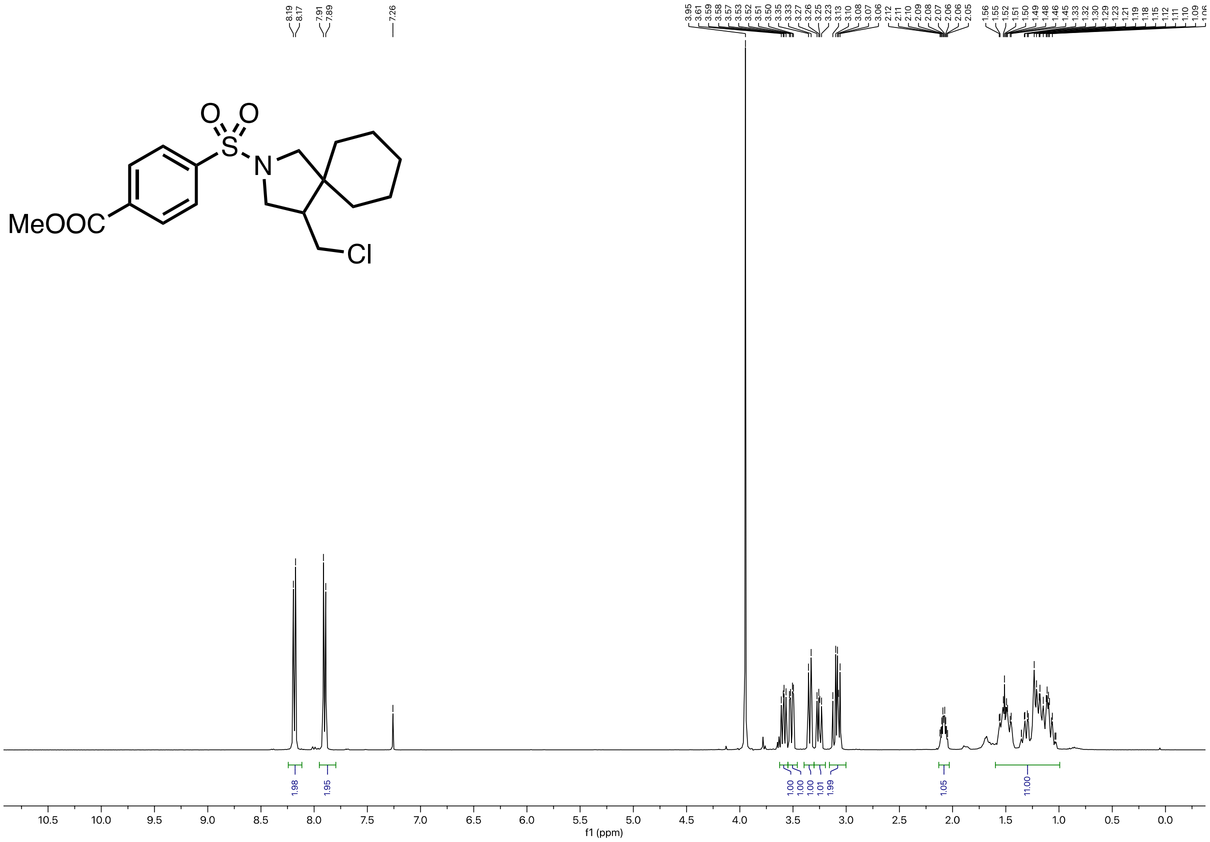

^{1}H NMR of *Methyl 4-((4-(chloromethyl)-2-azaspiro[4.5]decan-2-yl)sulfonyl)benzoate* (400 MHz, $CDCl_3$).

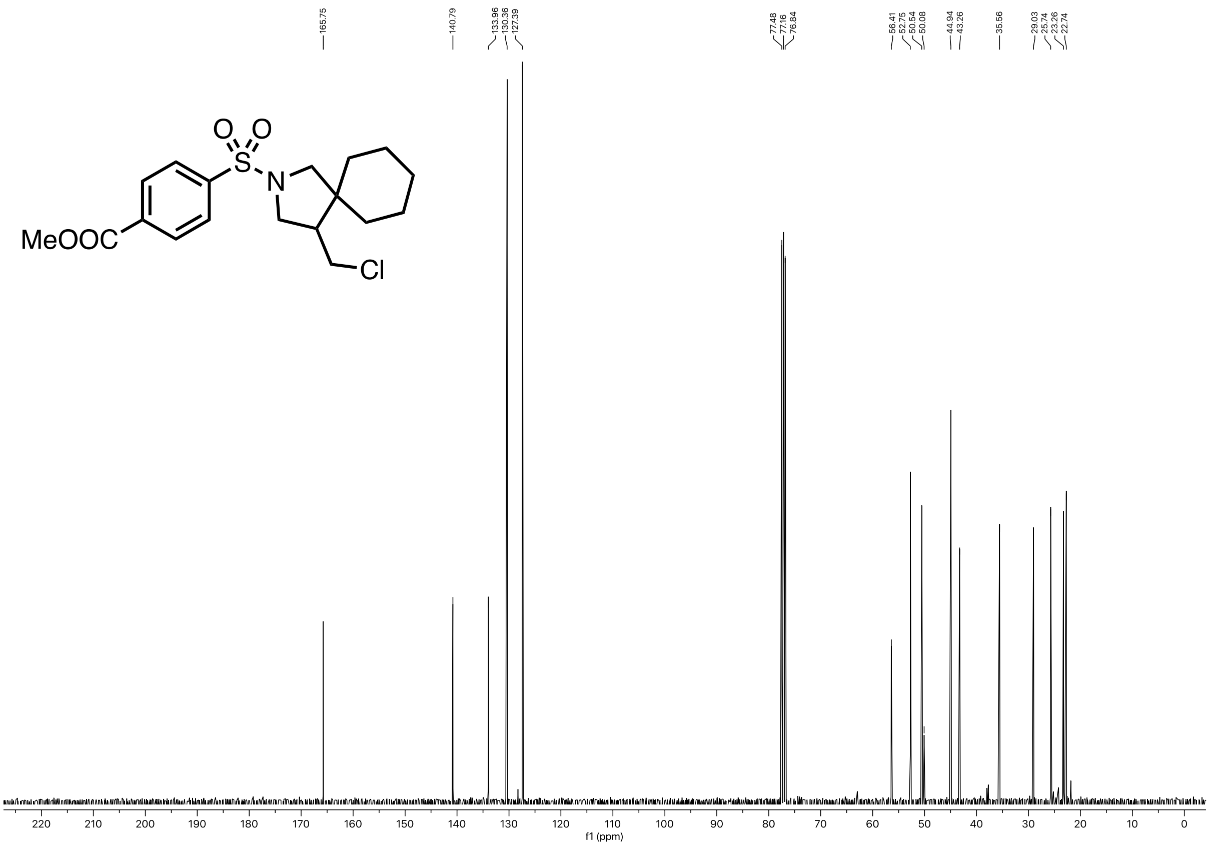

$^{13}C\{^1H\}$ NMR of *Methyl 4-((4-(chloromethyl)-2-azaspiro[4.5]decan-2-yl)sulfonyl)benzoate* (101 MHz, $CDCl_3$).

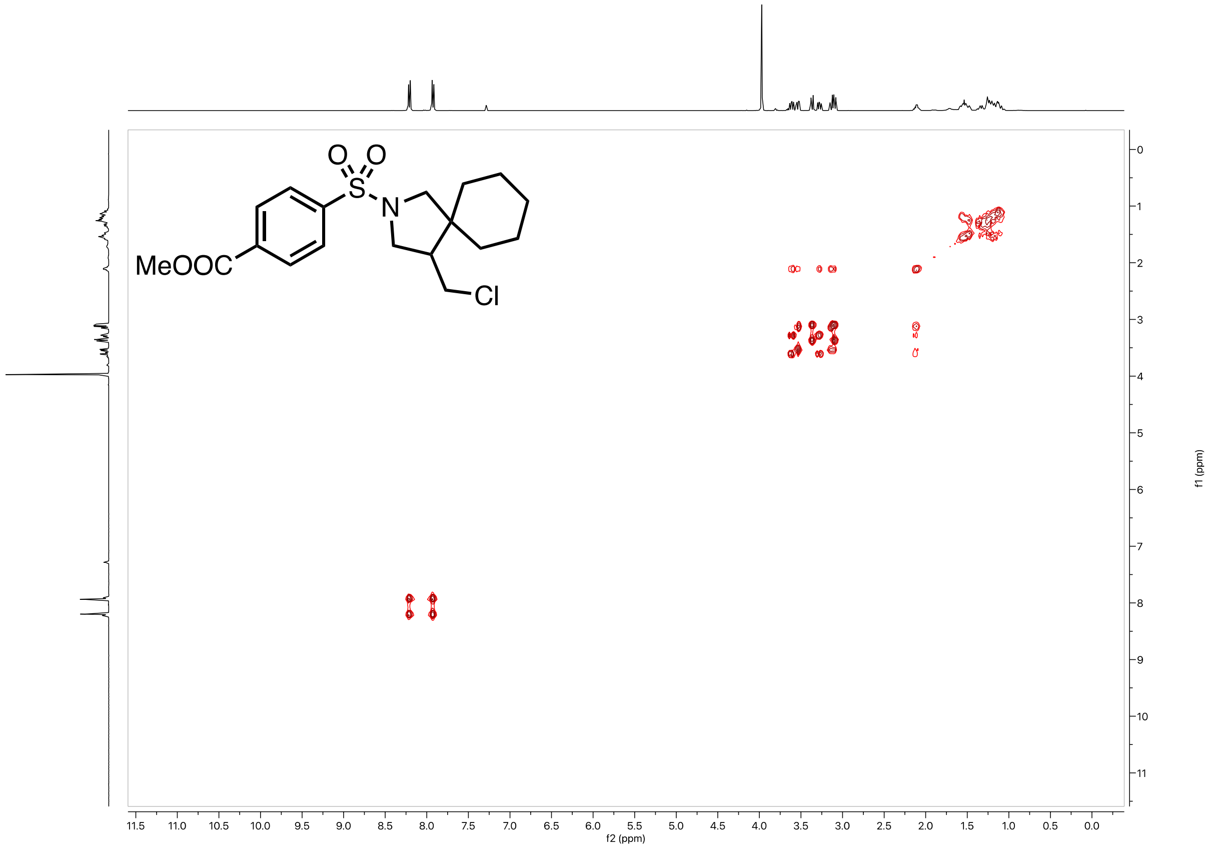

COSY of *Methyl 4-((4-(chloromethyl)-2-azaspiro[4.5]decan-2-yl)sulfonyl)benzoate* ($CDCl_3$).

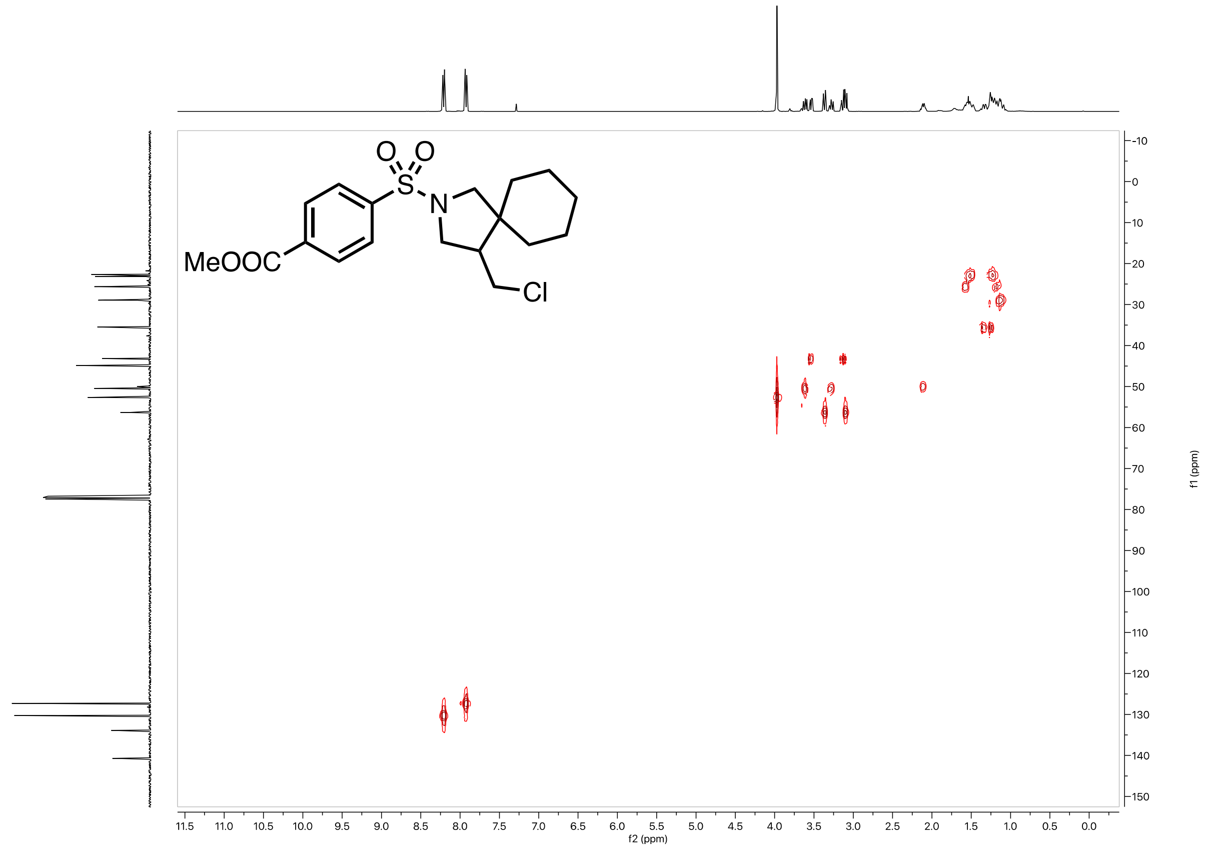

HSQC of *Methyl 4-((4-(chloromethyl)-2-azaspiro[4.5]decan-2-yl)sulfonyl)benzoate* ($CDCl_3$).

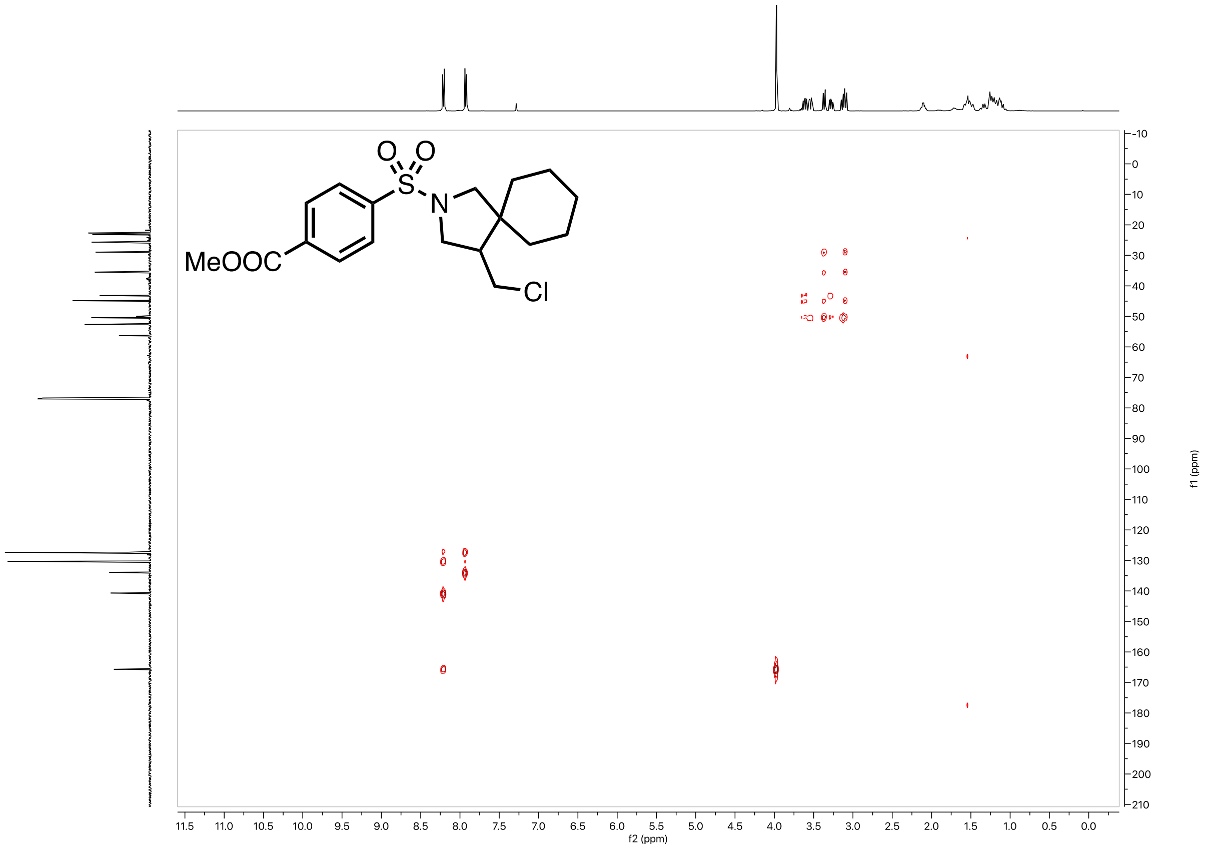

HMBC of *Methyl 4-((4-(chloromethyl)-2-azaspiro[4.5]decan-2-yl)sulfonyl)benzoate* ($CDCl_3$).

2-((3,5-bis(trifluoromethyl)phenyl)sulfonyl)-4-(chloromethyl)-2-azaspiro[4.5]decane (3d)

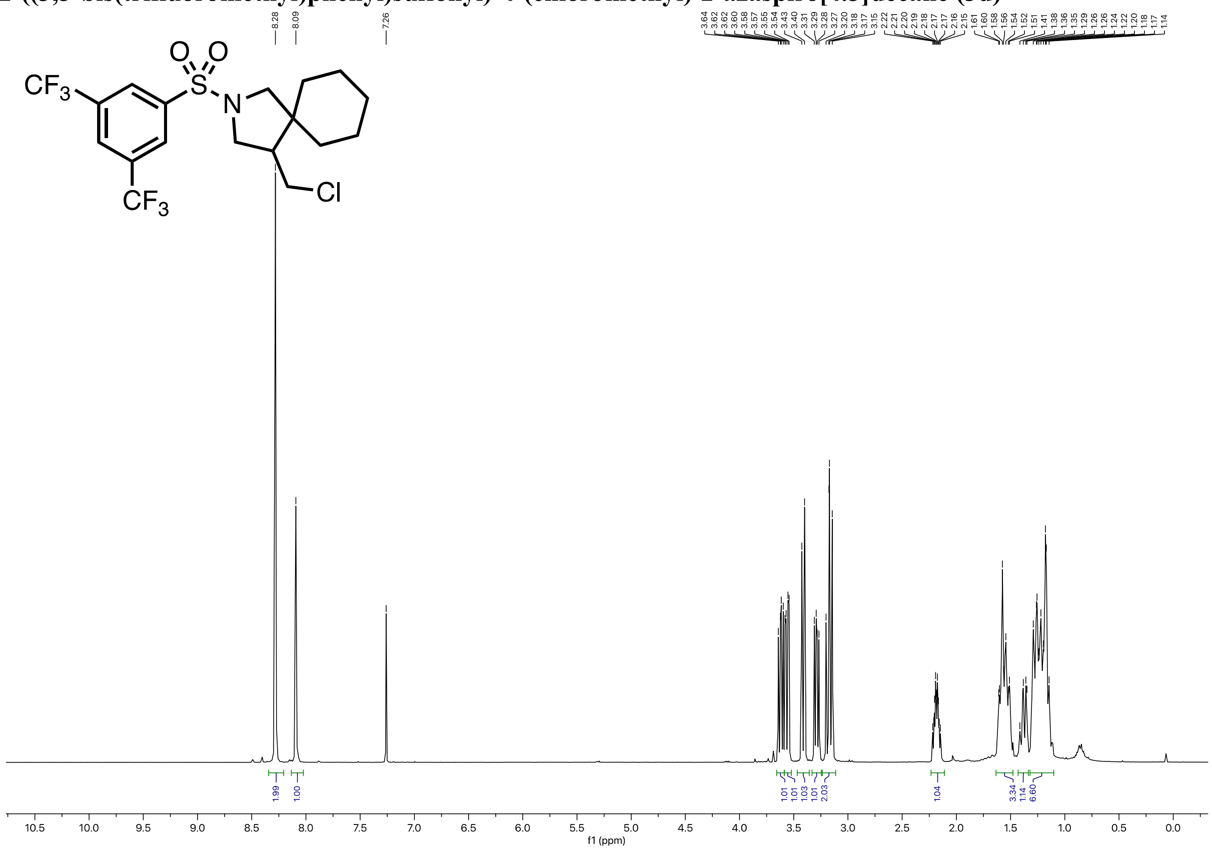

^{1}H NMR of *2-((3,5-bis(trifluoromethyl)phenyl)sulfonyl)-4-(chloromethyl)-2-azaspiro[4.5]decane* (400 MHz, $CDCl_3$).

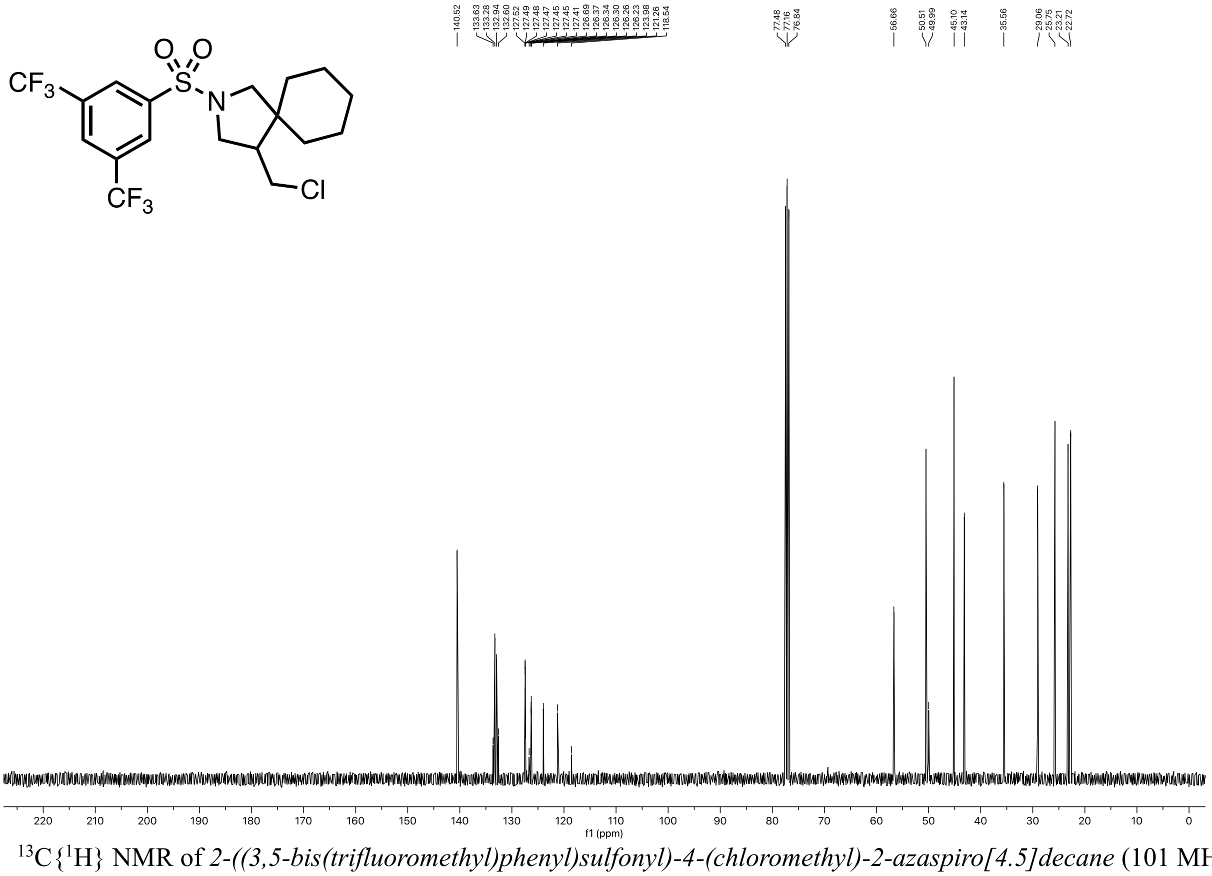

$^{13}C\{^{1}H\}$ NMR of *2-((3,5-bis(trifluoromethyl)phenyl)sulfonyl)-4-(chloromethyl)-2-azaspiro[4.5]decane* (101 MHz, $CDCl_3$).

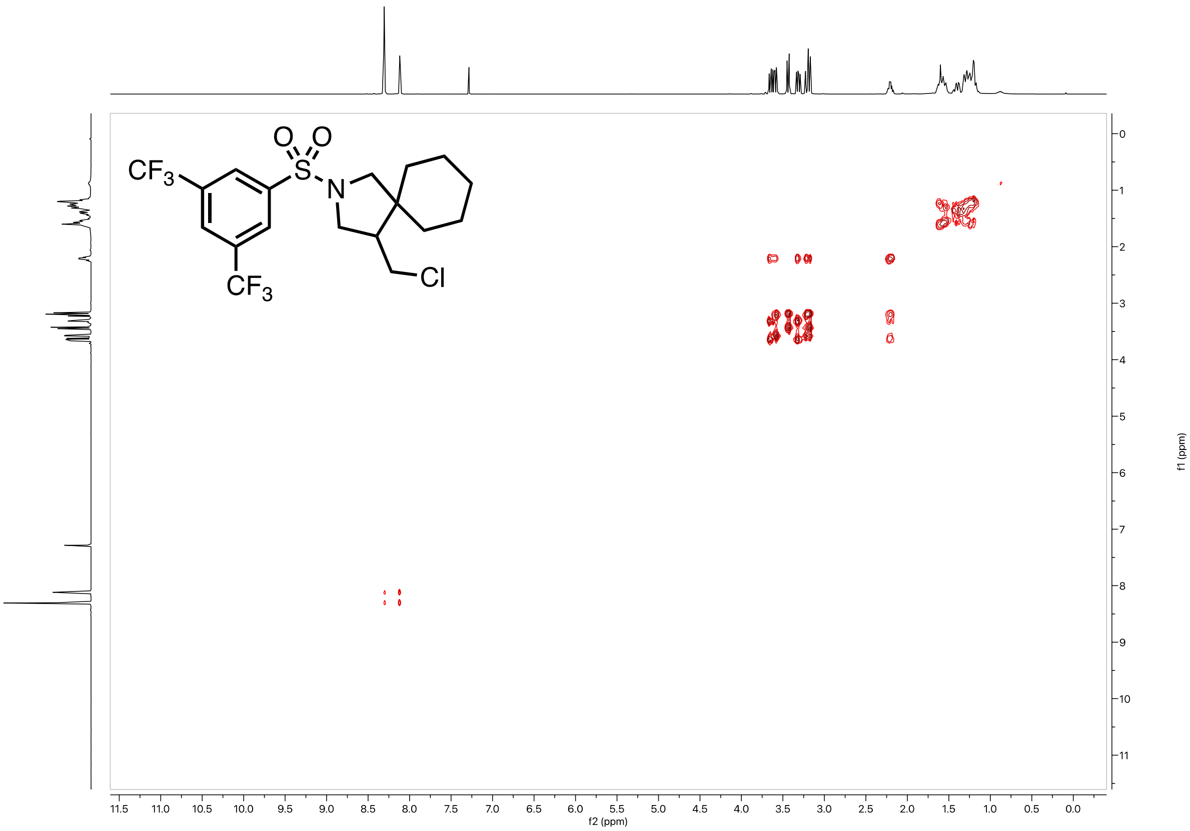

COSY of *2-((3,5-bis(trifluoromethyl)phenyl)sulfonyl)-4-(chloromethyl)-2-azaspiro[4.5]decane* ($CDCl_3$).

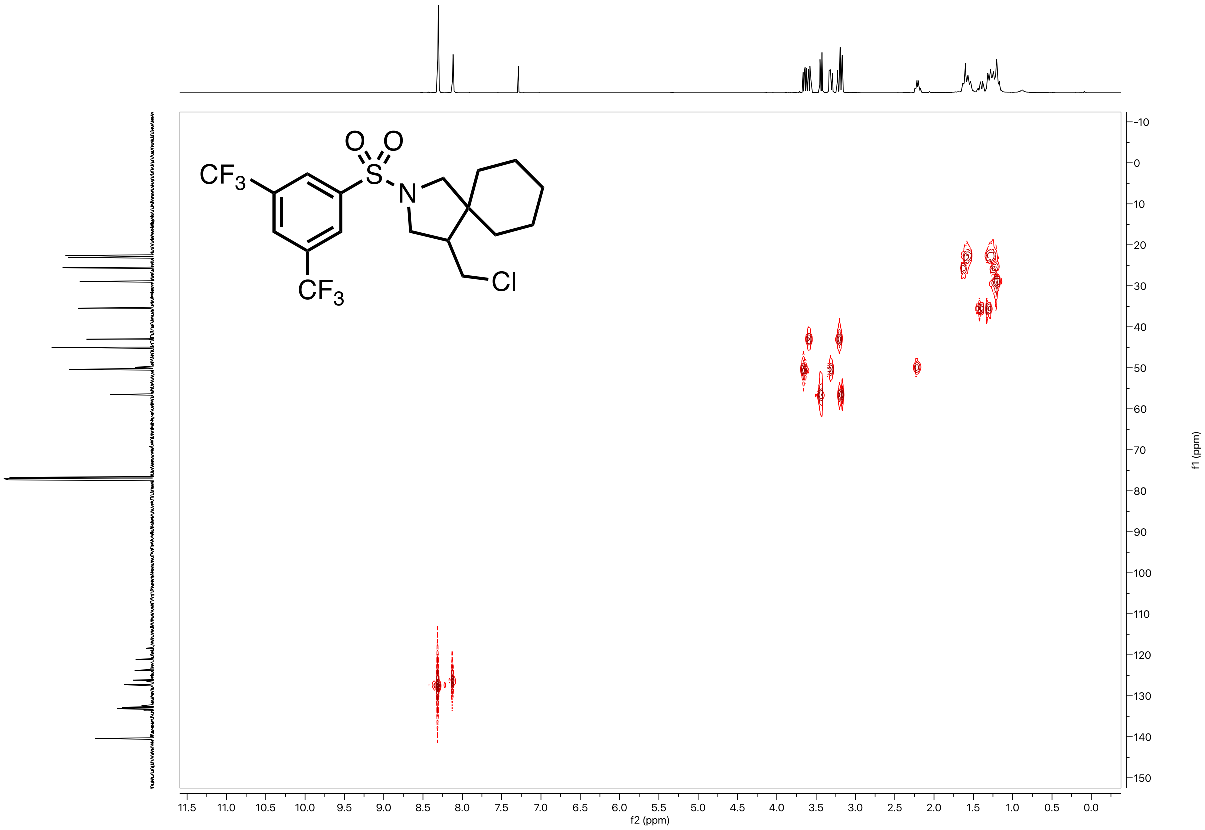

HSQC of *2-((3,5-bis(trifluoromethyl)phenyl)sulfonyl)-4-(chloromethyl)-2-azaspiro[4.5]decane* ($CDCl_3$).

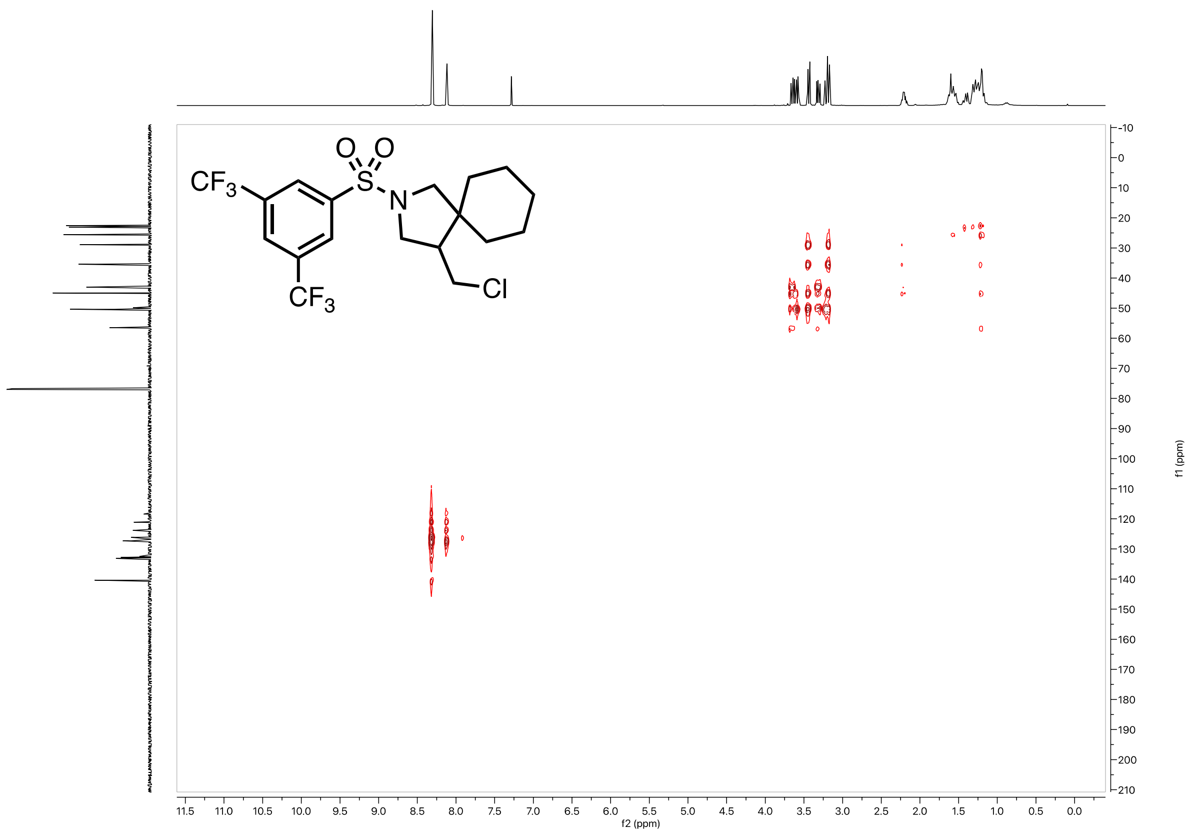

HMBC of *2-((3,5-bis(trifluoromethyl)phenyl)sulfonyl)-4-(chloromethyl)-2-azaspiro[4.5]decane* ($CDCl_3$).

3-(5-((4-(chloromethyl)-2-azaspiro[4.5]decan-2-yl)sulfonyl)thiophen-2-yl)-5-(trifluoromethyl)isoxazole (3e)

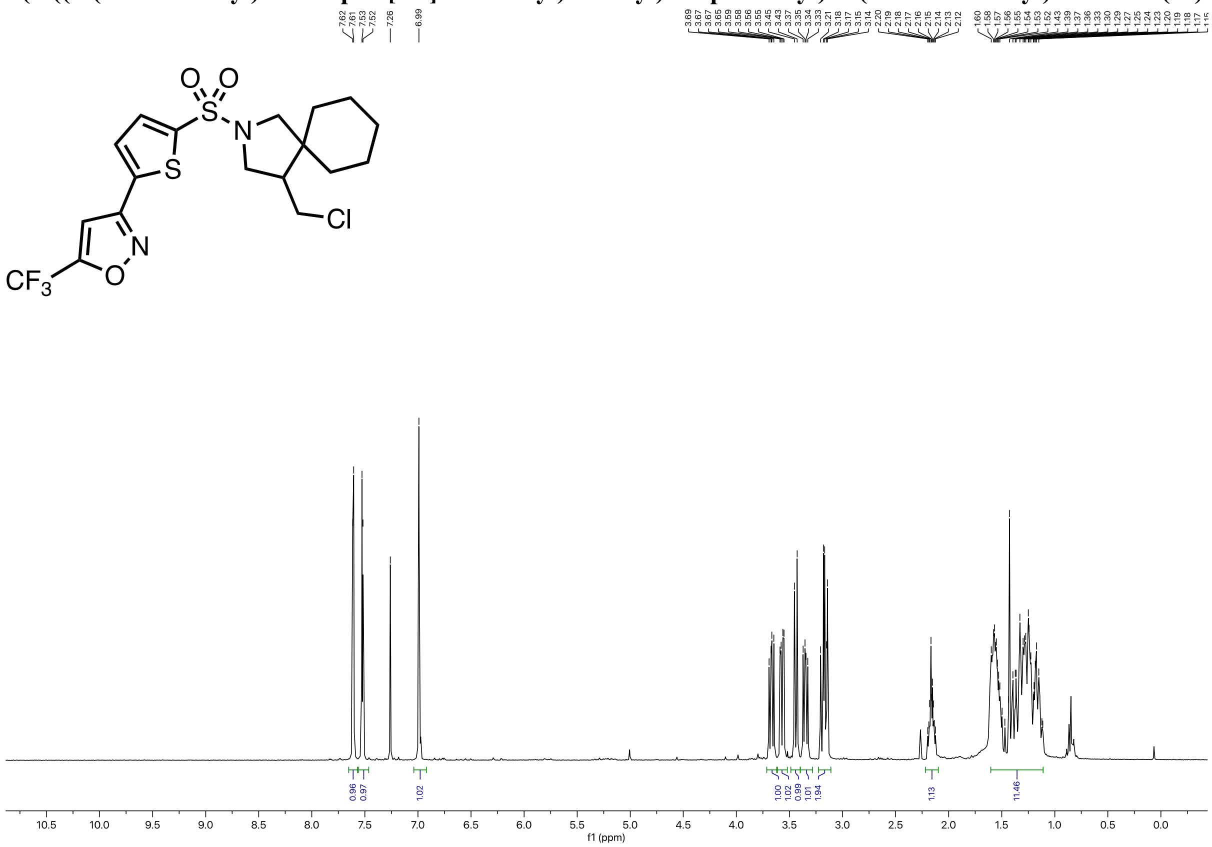

^{1}H NMR of *3-(5-((4-(chloromethyl)-2-azaspiro[4.5]decan-2-yl)sulfonyl)thiophen-2-yl)-5-(trifluoromethyl)isoxazole* (400 MHz, $CDCl_3$).

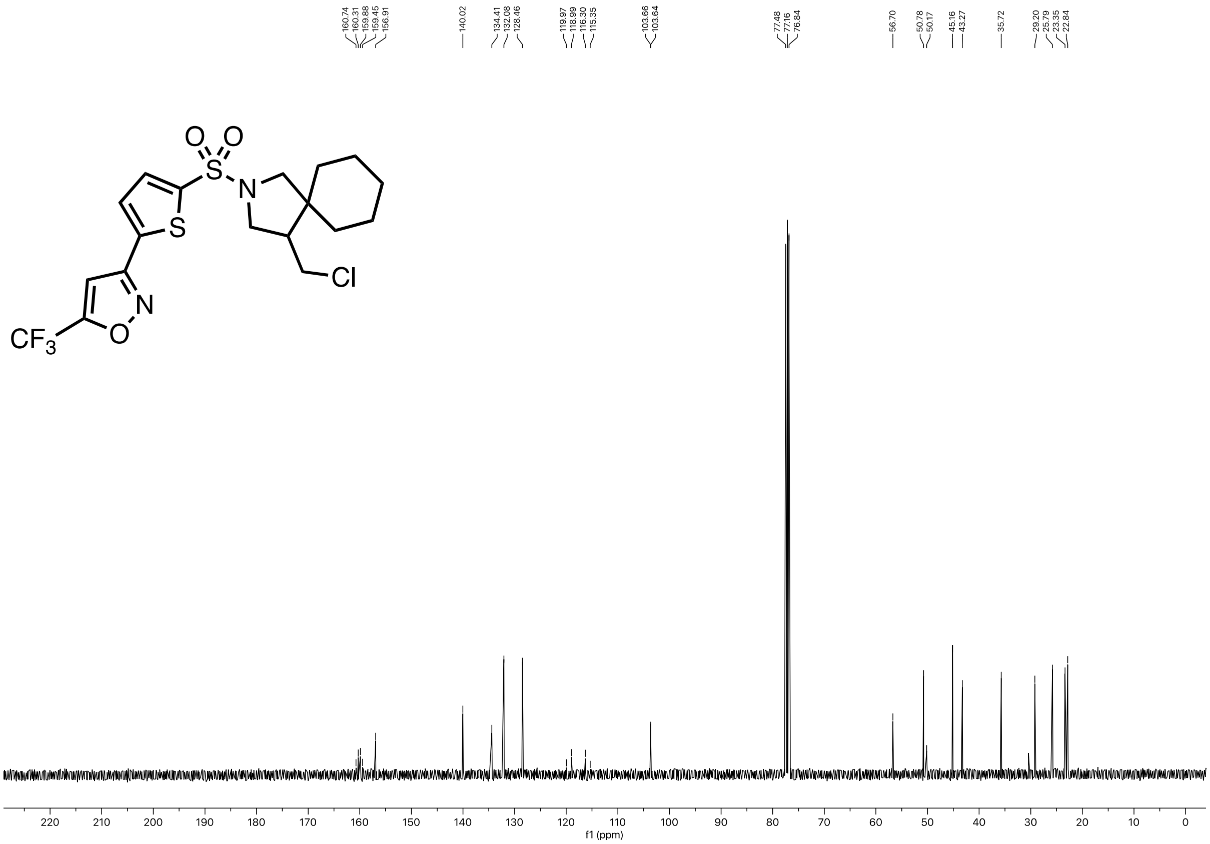

$^{13}C\{^{1}H\}$ NMR of *3-(5-((4-(chloromethyl)-2-azaspiro[4.5]decan-2-yl)sulfonyl)thiophen-2-yl)-5-(trifluoromethyl)isoxazole* (101 MHz, $CDCl_3$).

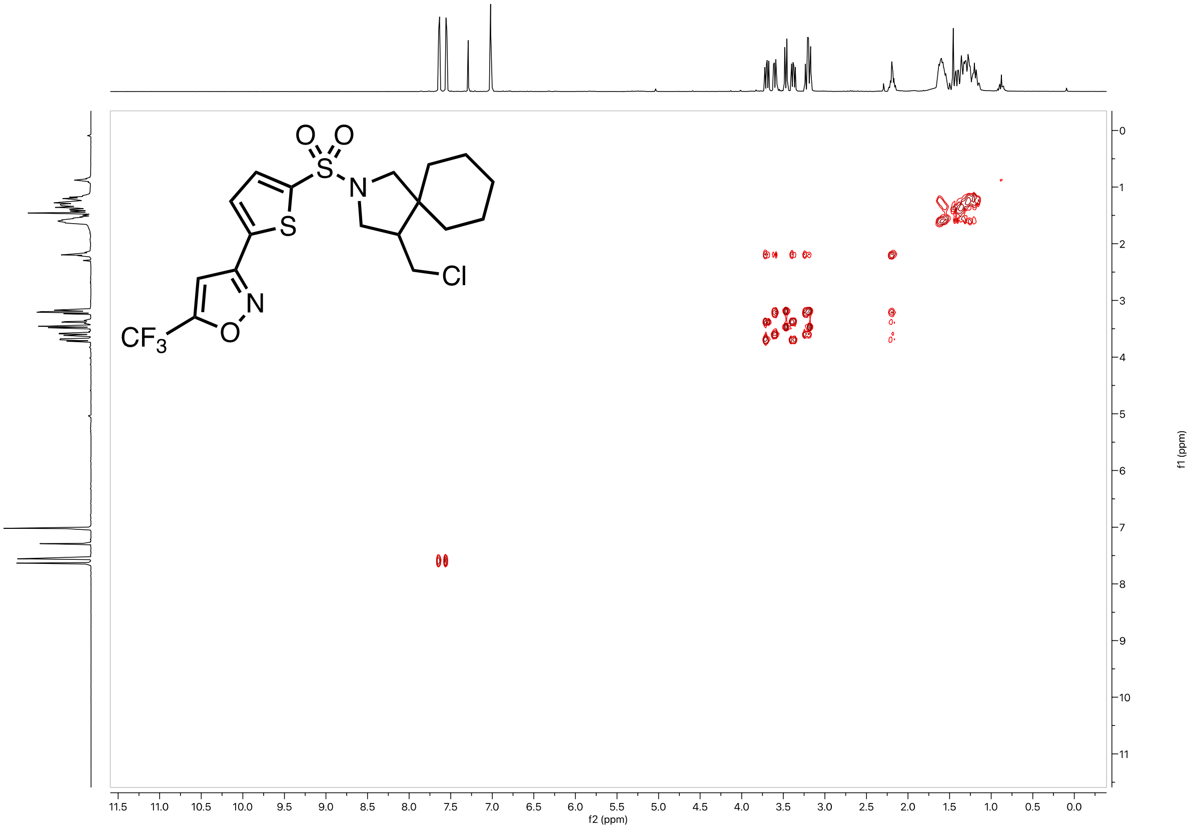

COSY of *3-(5-((4-(chloromethyl)-2-azaspiro[4.5]decan-2-yl)sulfonyl)thiophen-2-yl)-5-(trifluoromethyl)isoxazole* ($CDCl_3$).

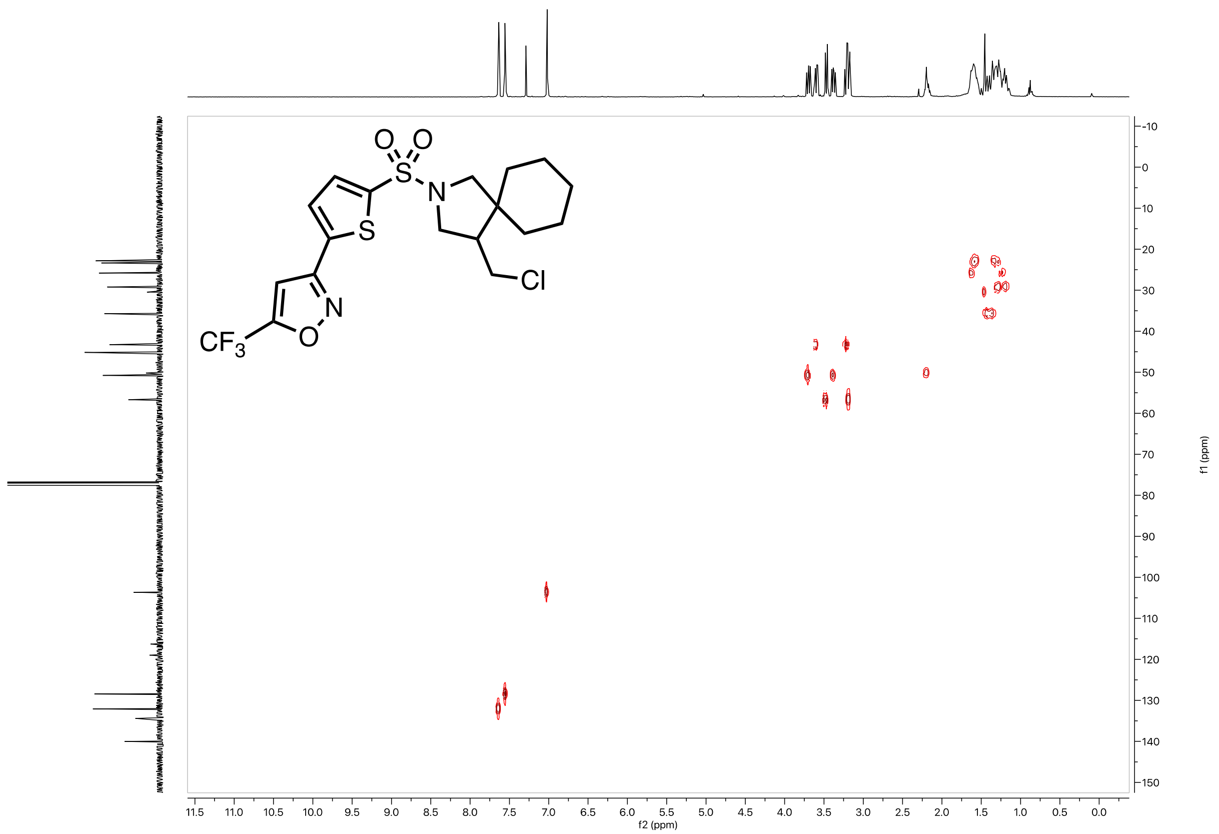

HSQC of *3-(5-((4-(chloromethyl)-2-azaspiro[4.5]decan-2-yl)sulfonyl)thiophen-2-yl)-5-(trifluoromethyl)isoxazole* ($CDCl_3$).

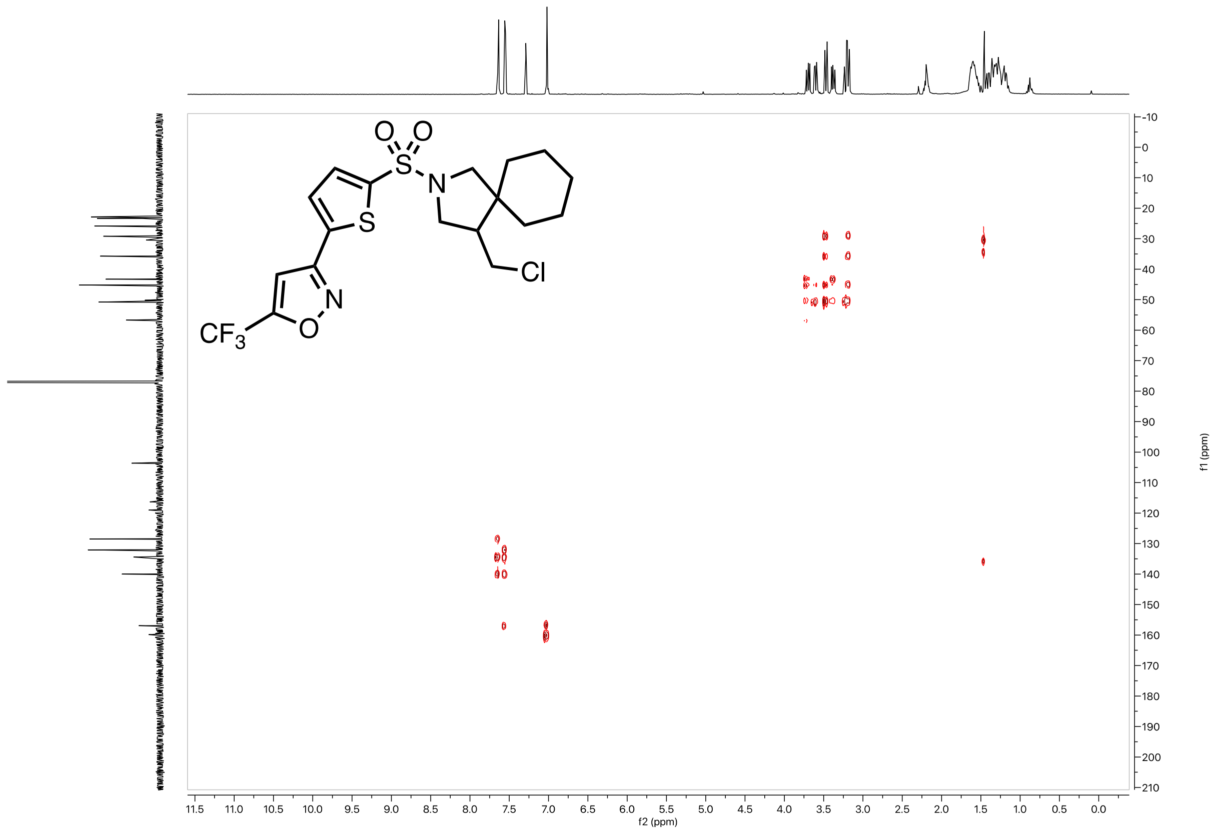

HMBC of *3-(5-((4-(chloromethyl)-2-azaspiro[4.5]decan-2-yl)sulfonyl)thiophen-2-yl)-5-(trifluoromethyl)isoxazole* ($CDCl_3$).

4-(chloromethyl)-2-((4-(trifluoromethoxy)phenyl)sulfonyl)-2-azaspiro[4.5]decane (3f)

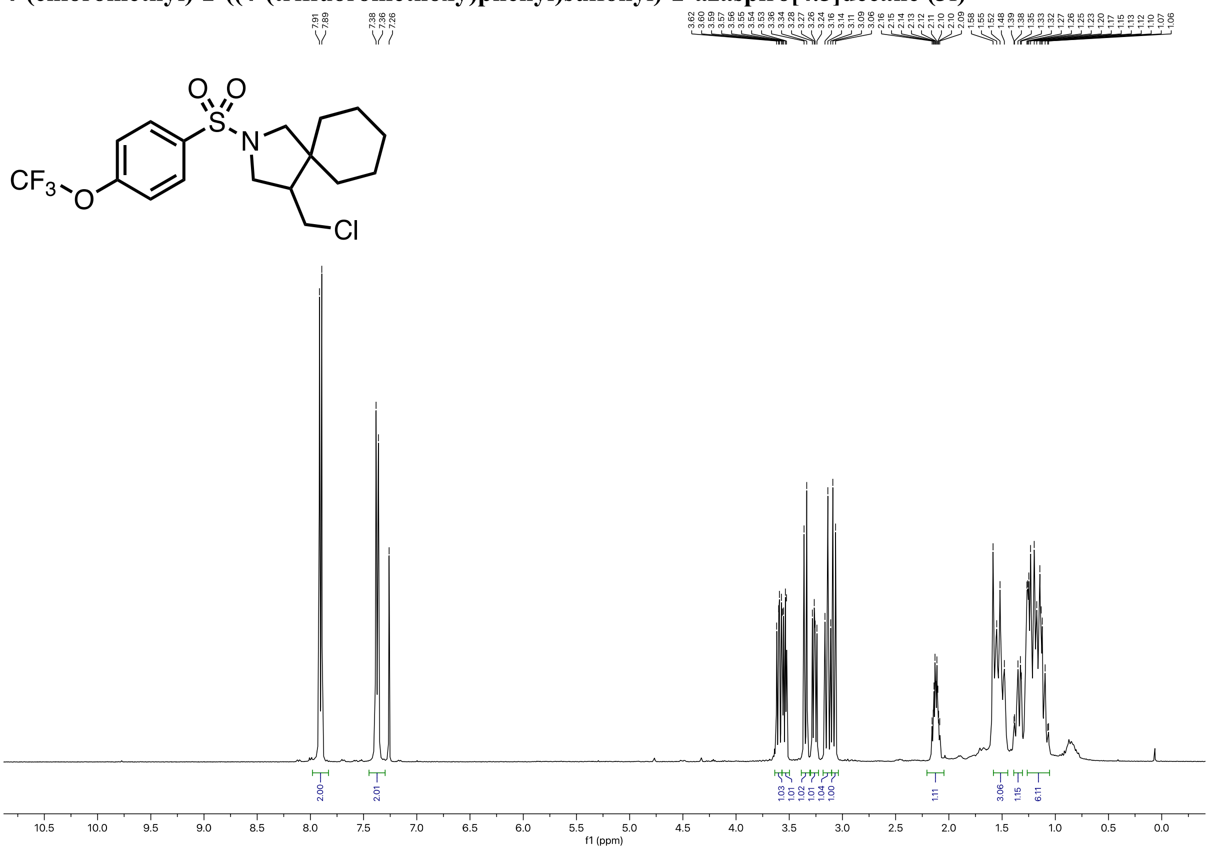

^{1}H NMR of *4-(chloromethyl)-2-((4-(trifluoromethoxy)phenyl)sulfonyl)-2-azaspiro[4.5]decane* (400 MHz, $CDCl_3$).

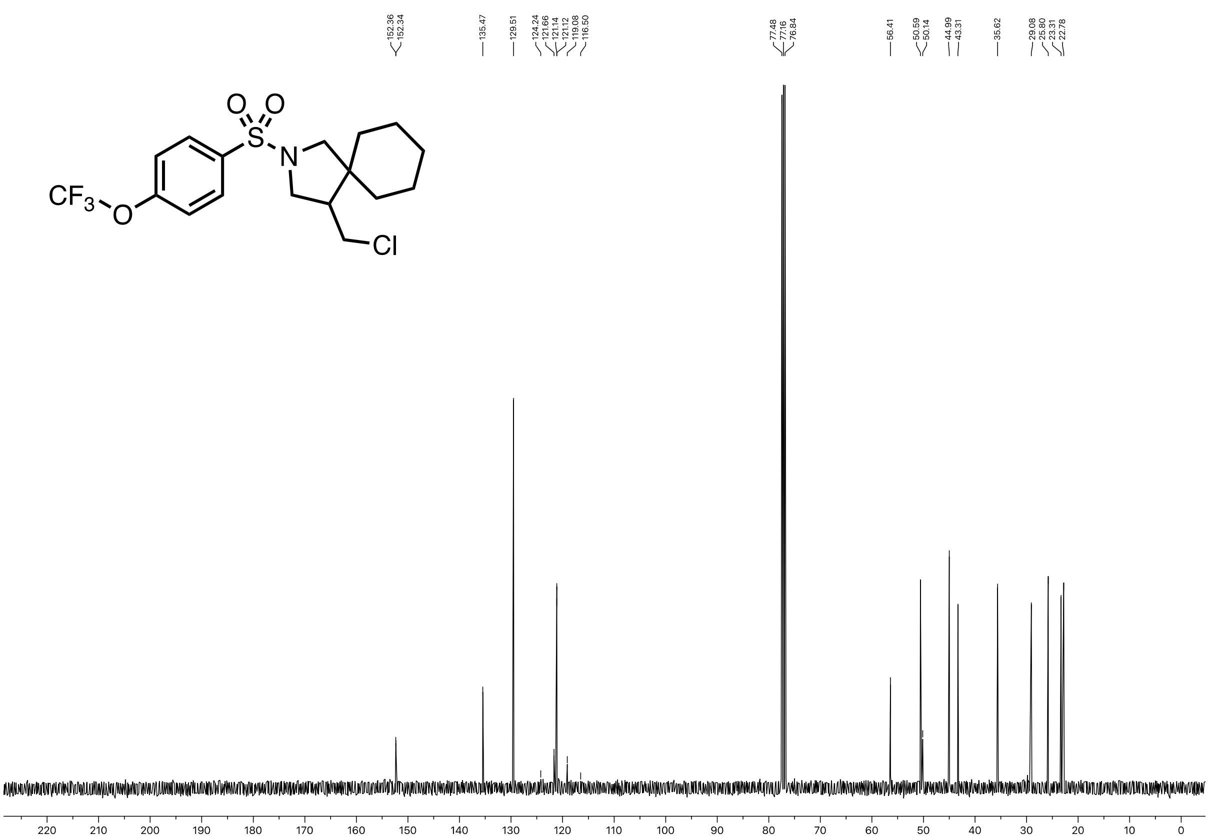

^{13}C{^{1}H} NMR of *4-(chloromethyl)-2-((4-(trifluoromethoxy)phenyl)sulfonyl)-2-azaspiro[4.5]decane* (101 MHz, $CDCl_3$).

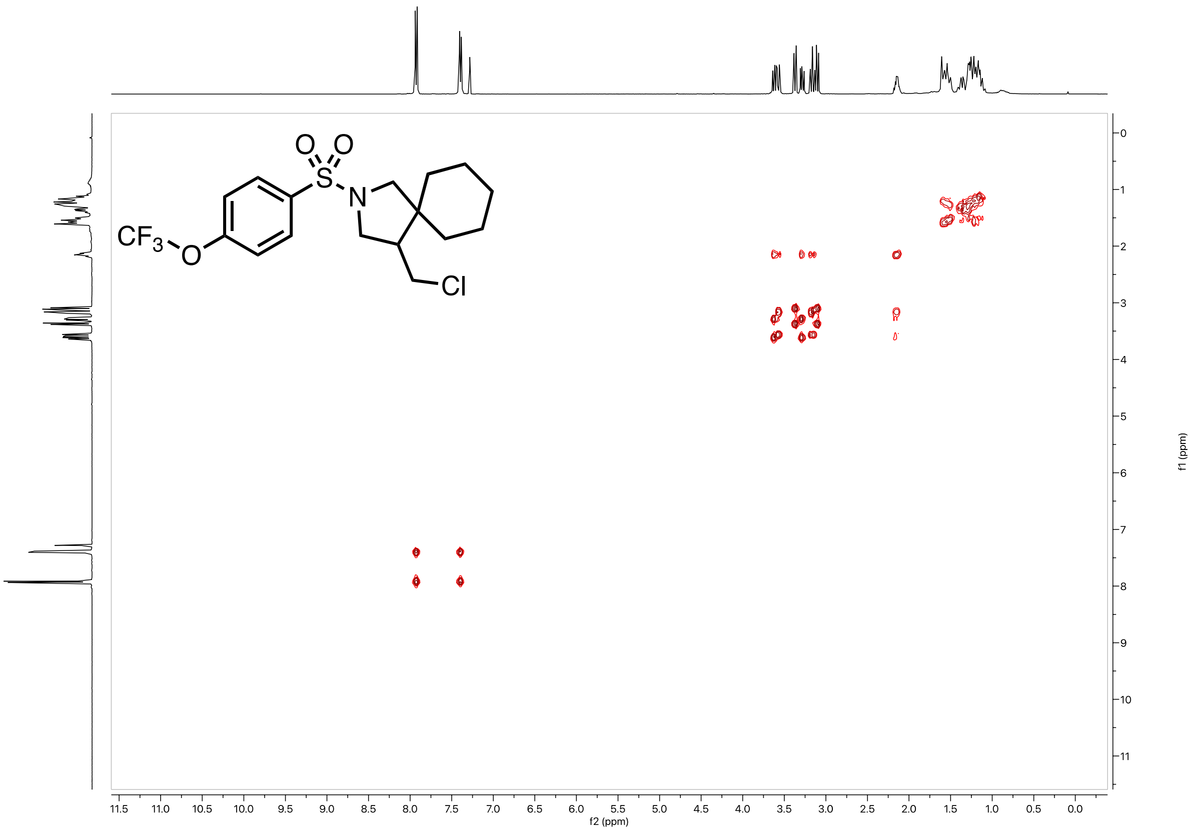

COSY of *4-(chloromethyl)-2-((4-(trifluoromethoxy)phenyl)sulfonyl)-2-azaspiro[4.5]decane* ($CDCl_3$).

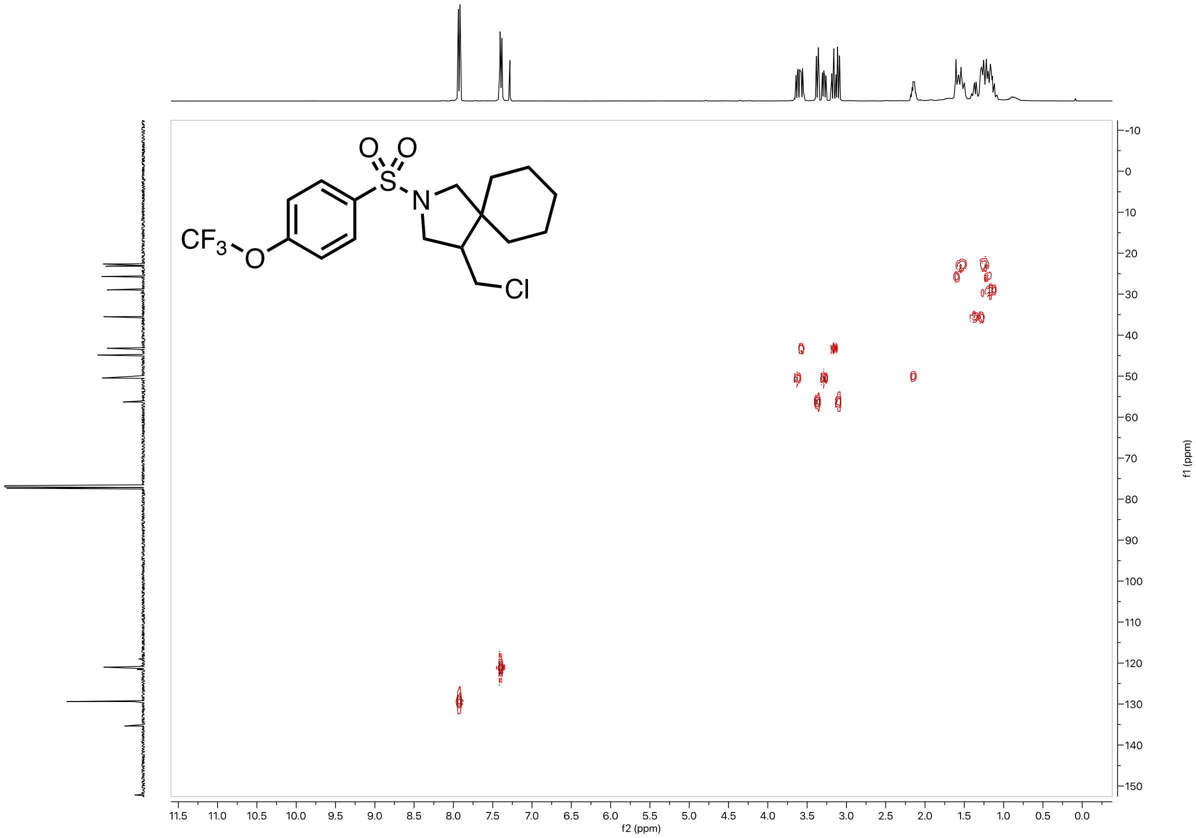

HSQC of *4-(chloromethyl)-2-((4-(trifluoromethoxy)phenyl)sulfonyl)-2-azaspiro[4.5]decane* ($CDCl_3$).

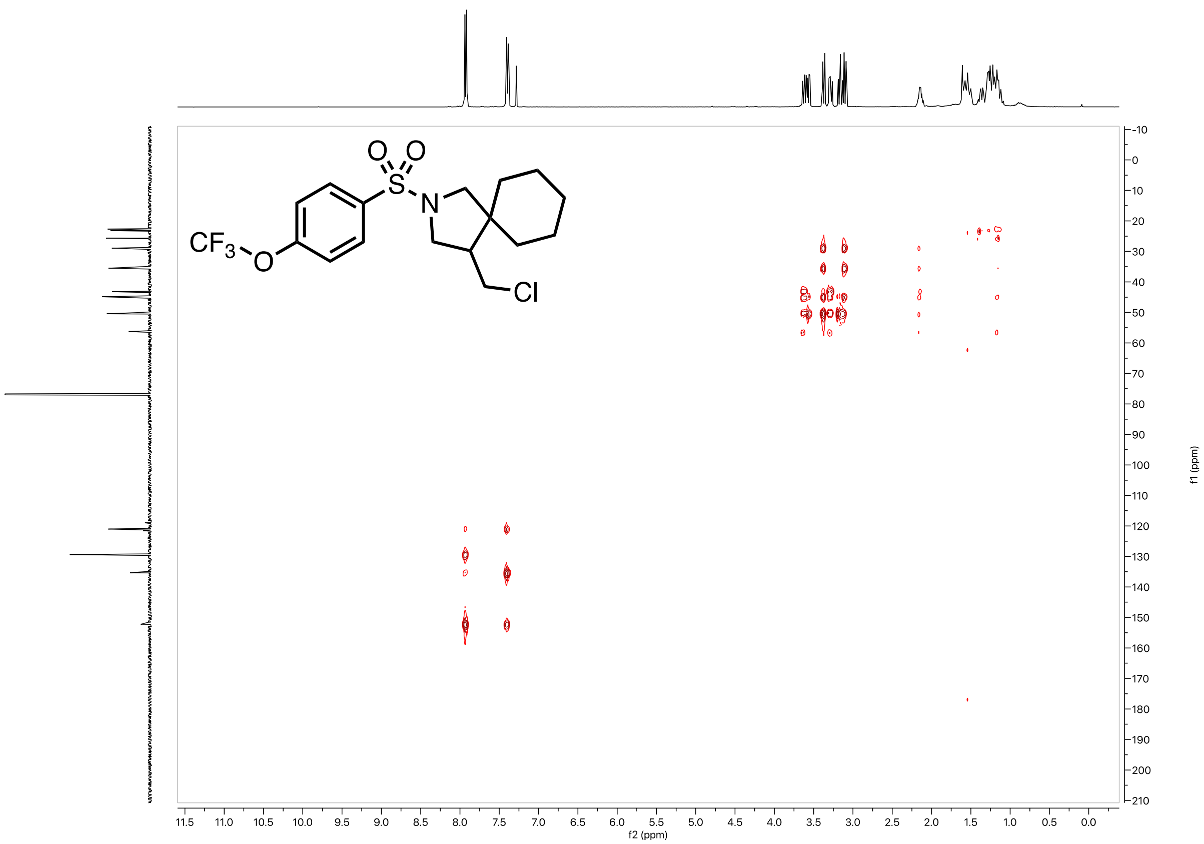

HMBC of *4-(chloromethyl)-2-((4-(trifluoromethoxy)phenyl)sulfonyl)-2-azaspiro[4.5]decane* ($CDCl_3$).

4-(chloromethyl)-2-((4-(methylsulfonyl)phenyl)sulfonyl)-2-azaspiro[4.5]decane (3g)

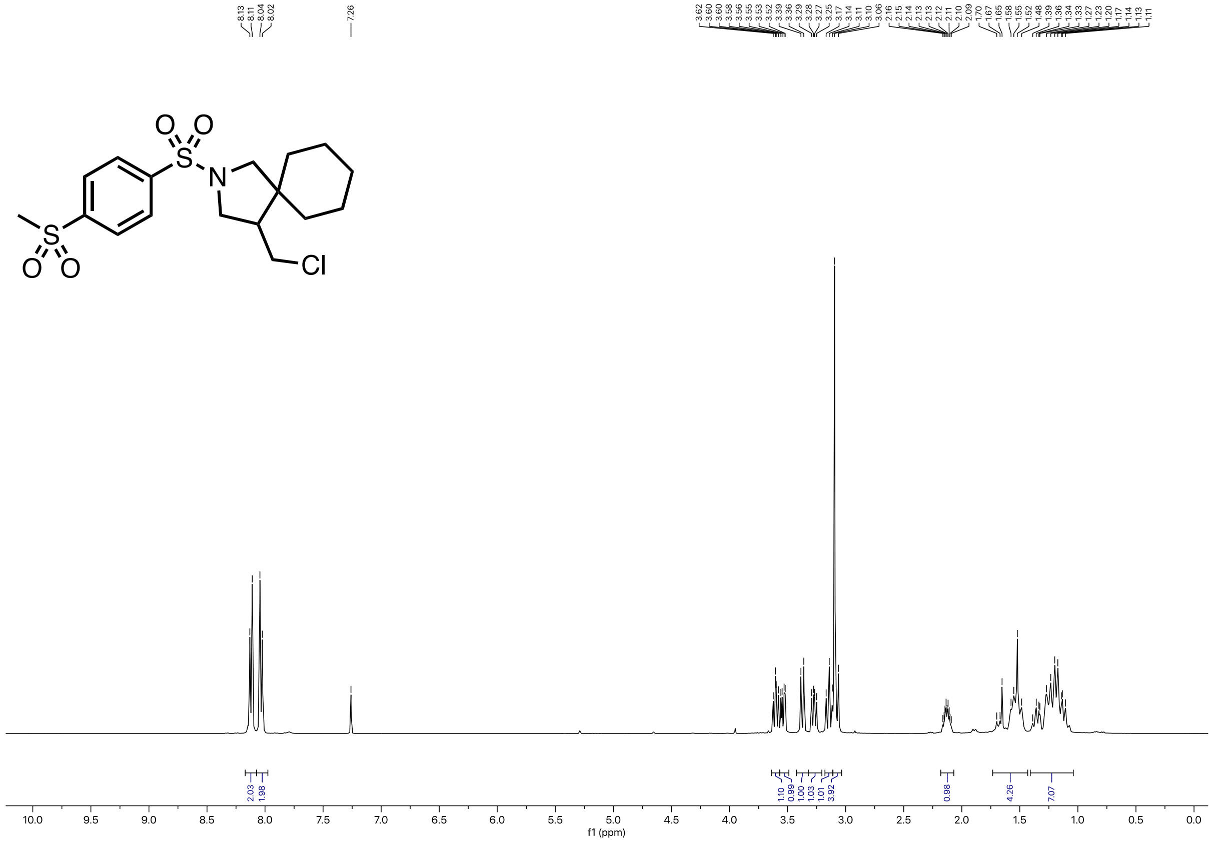

^{1}H NMR of *4-(chloromethyl)-2-((4-(methylsulfonyl)phenyl)sulfonyl)-2-azaspiro[4.5]decane* (400 MHz, $CDCl_3$).

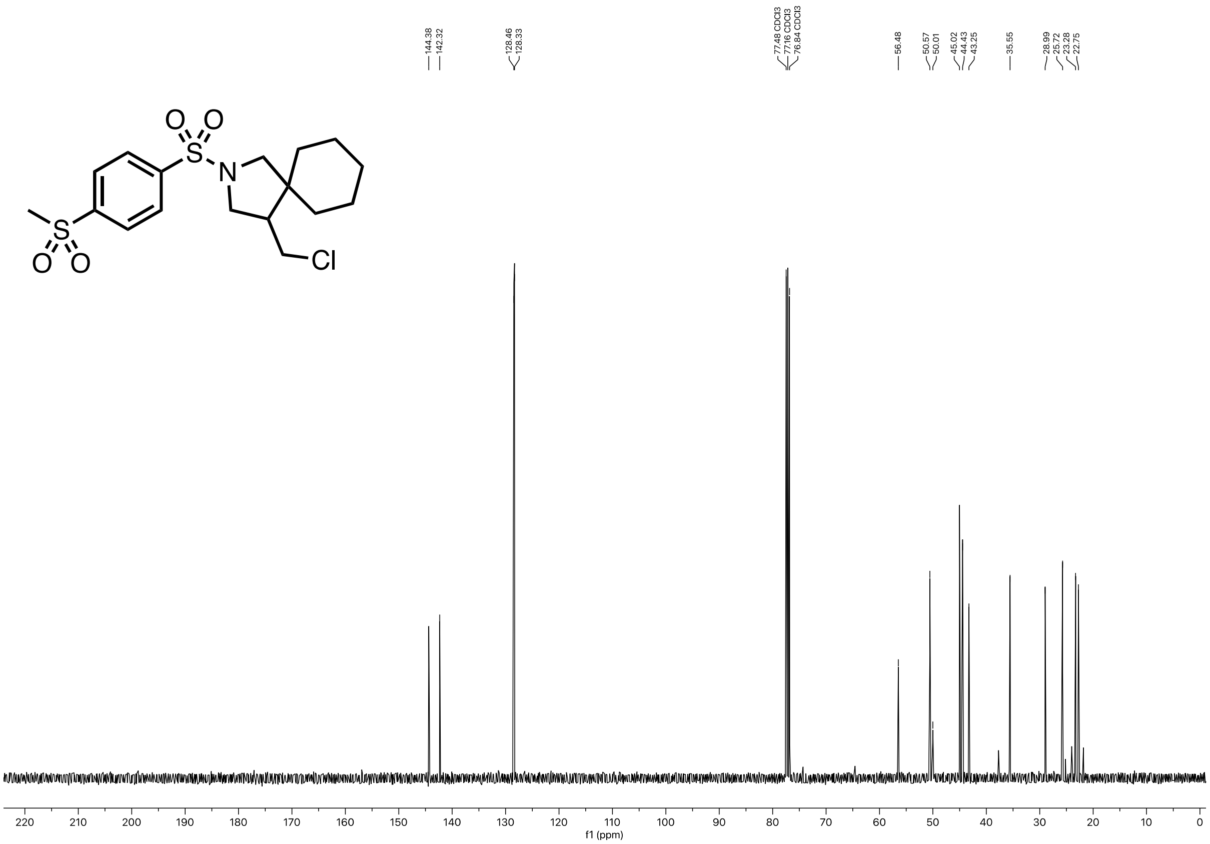

$^{13}C\{^{1}H\}$ NMR of *4-(chloromethyl)-2-((4-(methylsulfonyl)phenyl)sulfonyl)-2-azaspiro[4.5]decane* (101 MHz, $CDCl_3$).

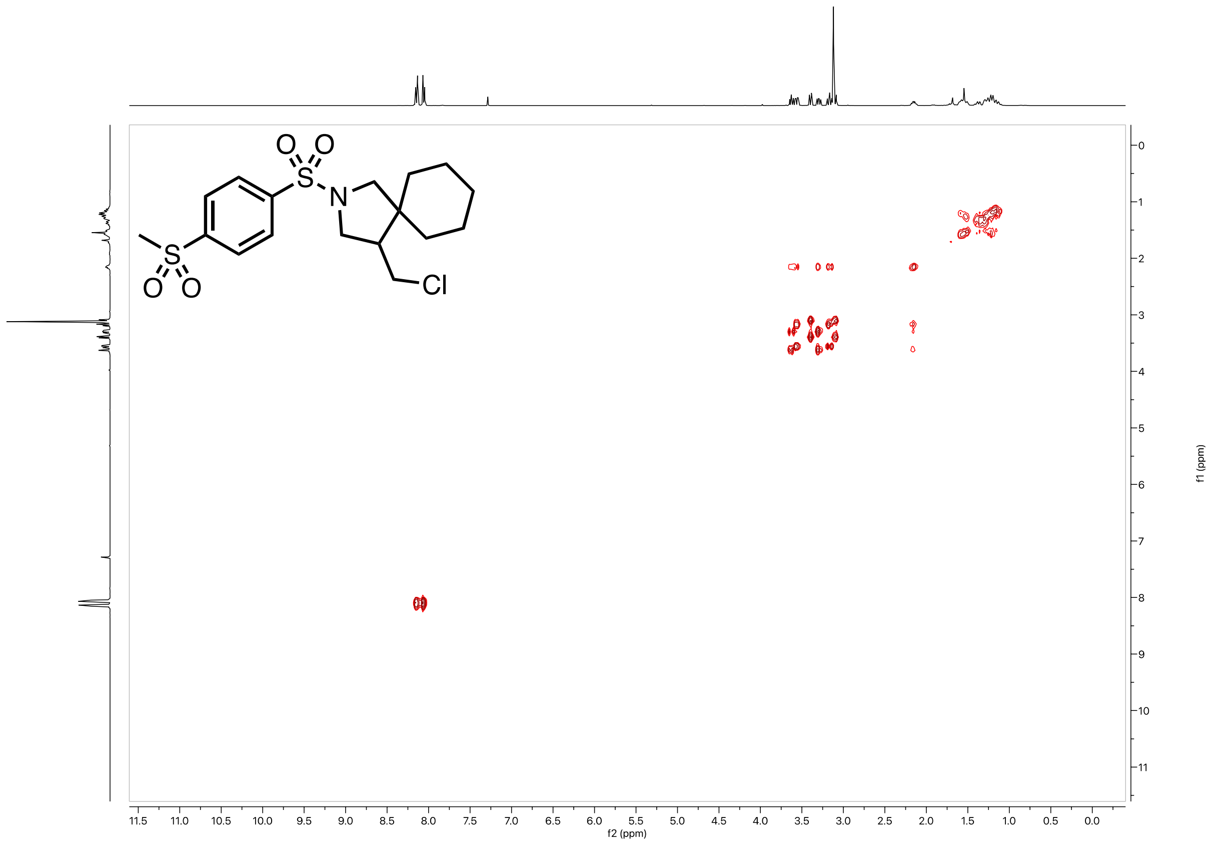

COSY of *4-(chloromethyl)-2-((4-(methylsulfonyl)phenyl)sulfonyl)-2-azaspiro[4.5]decane* ($CDCl_3$).

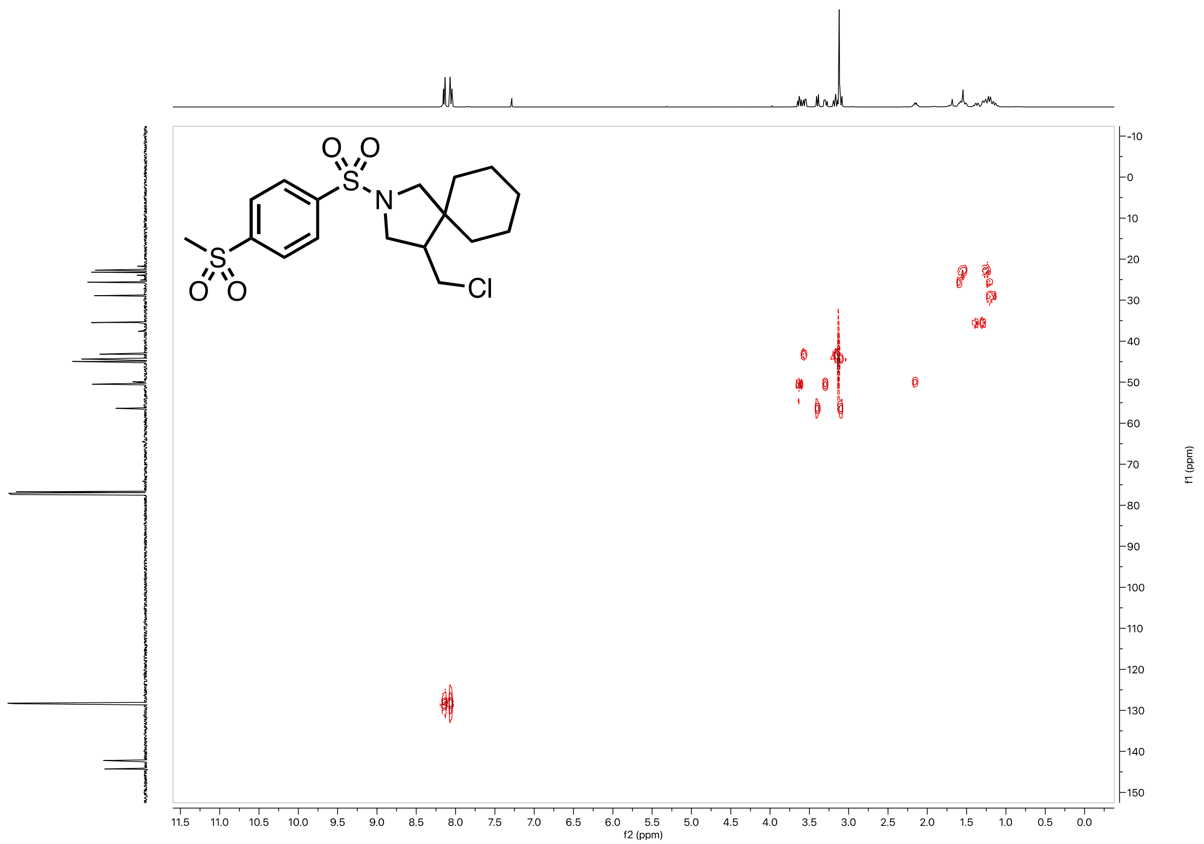

HSQC of *4-(chloromethyl)-2-((4-(methylsulfonyl)phenyl)sulfonyl)-2-azaspiro[4.5]decane* ($CDCl_3$).

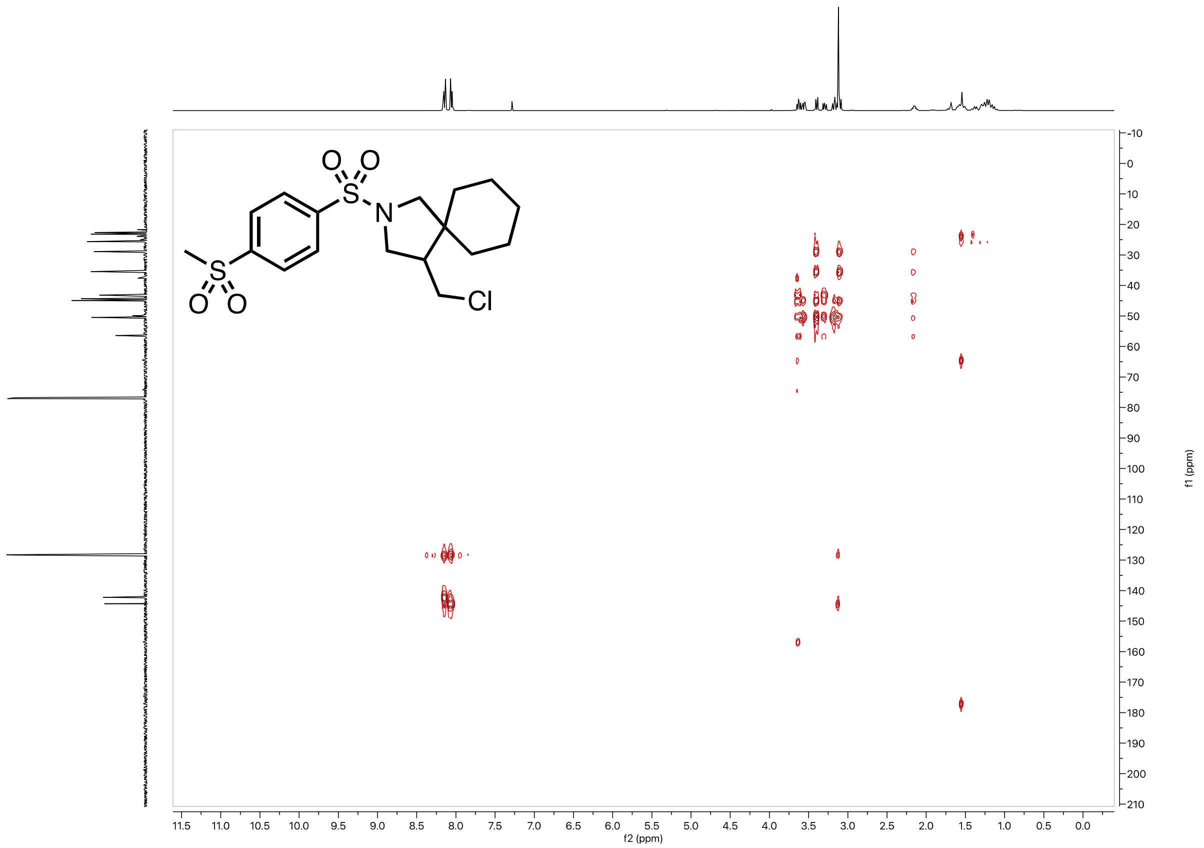

HMBC of *4-(chloromethyl)-2-((4-(methylsulfonyl)phenyl)sulfonyl)-2-azaspiro[4.5]decane* ($CDCl_3$).

4-(chloro(phenyl)methyl)-2-tosyl-2-azaspiro[4.5]decane (3h)

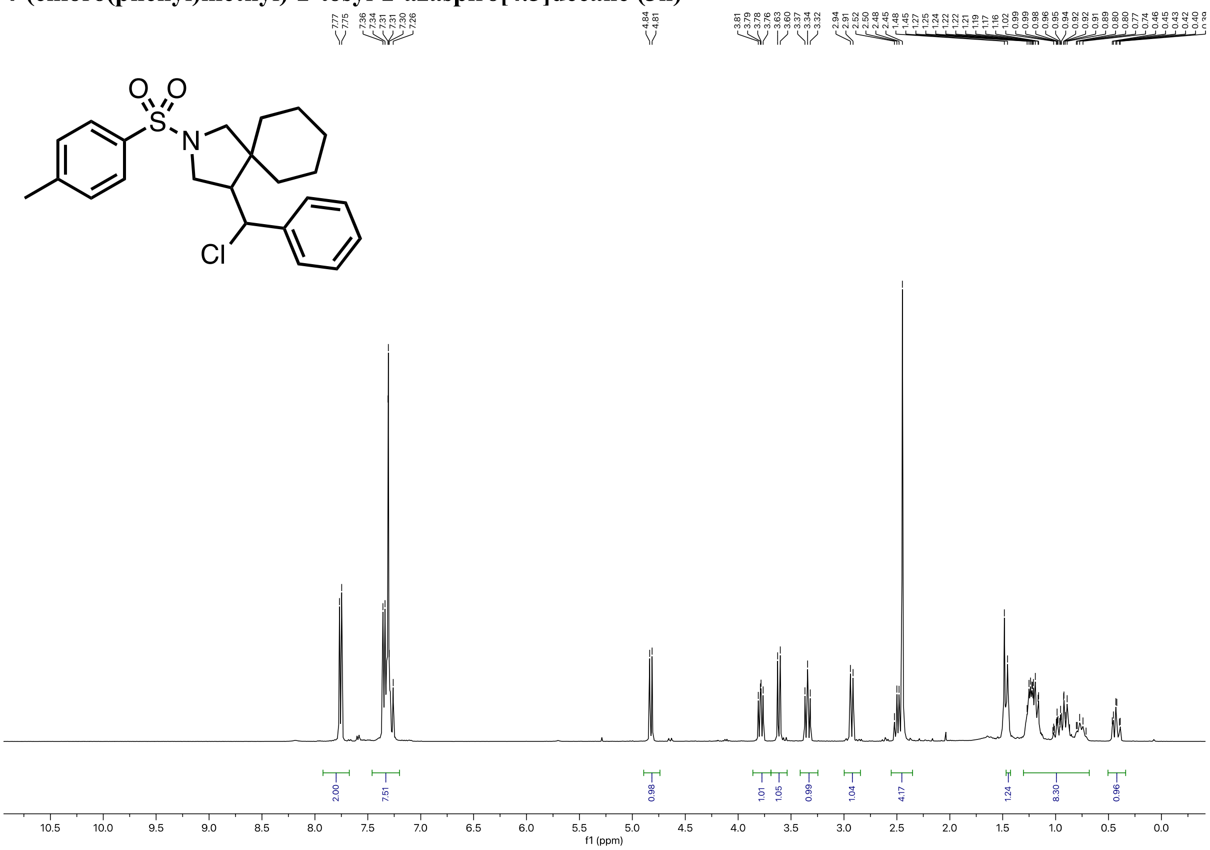

^{1}H NMR of *4-(chloro(phenyl)methyl)-2-tosyl-2-azaspiro[4.5]decane* (400 MHz, $CDCl_3$).

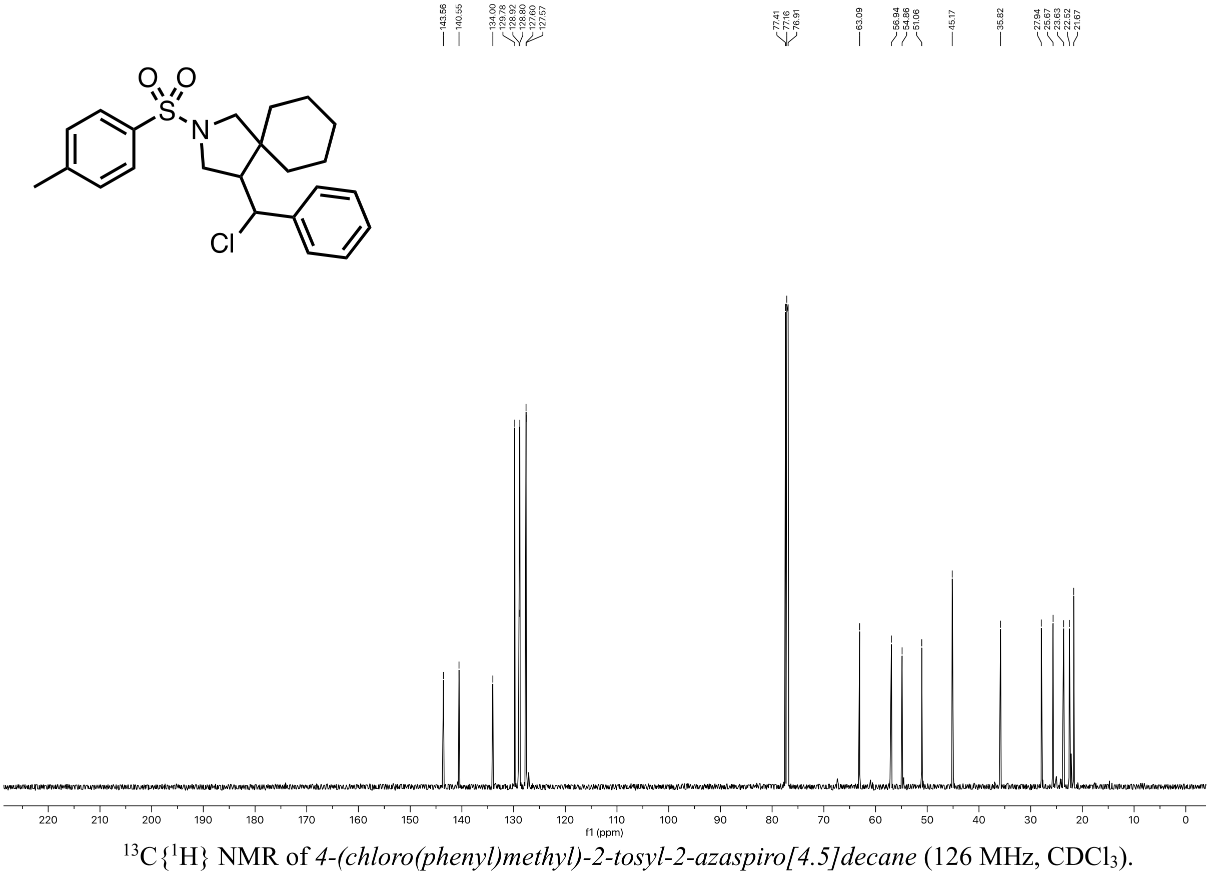

$^{13}C\{^1H\}$ NMR of *4-(chloro(phenyl)methyl)-2-tosyl-2-azaspiro[4.5]decane* (126 MHz, $CDCl_3$).

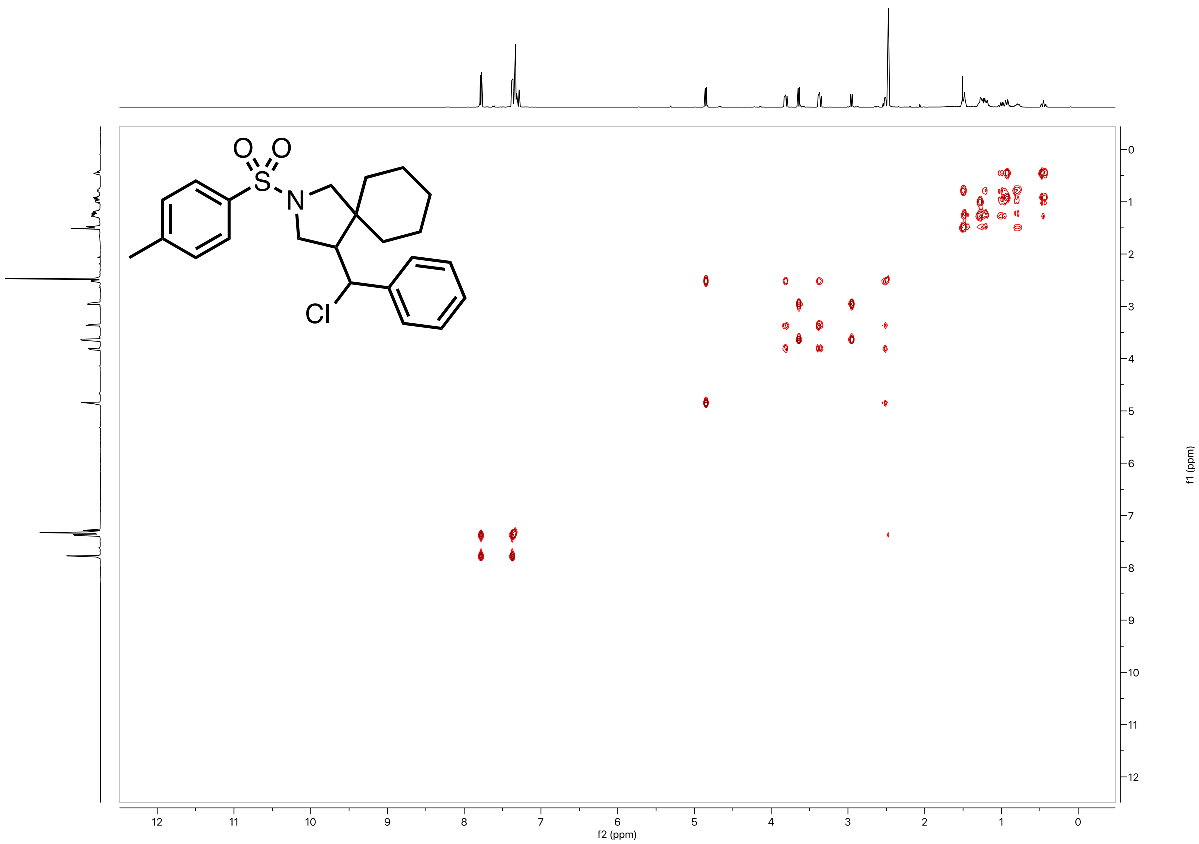

COSY of *4-(chloro(phenyl)methyl)-2-tosyl-2-azaspiro[4.5]decane* ($CDCl_3$).

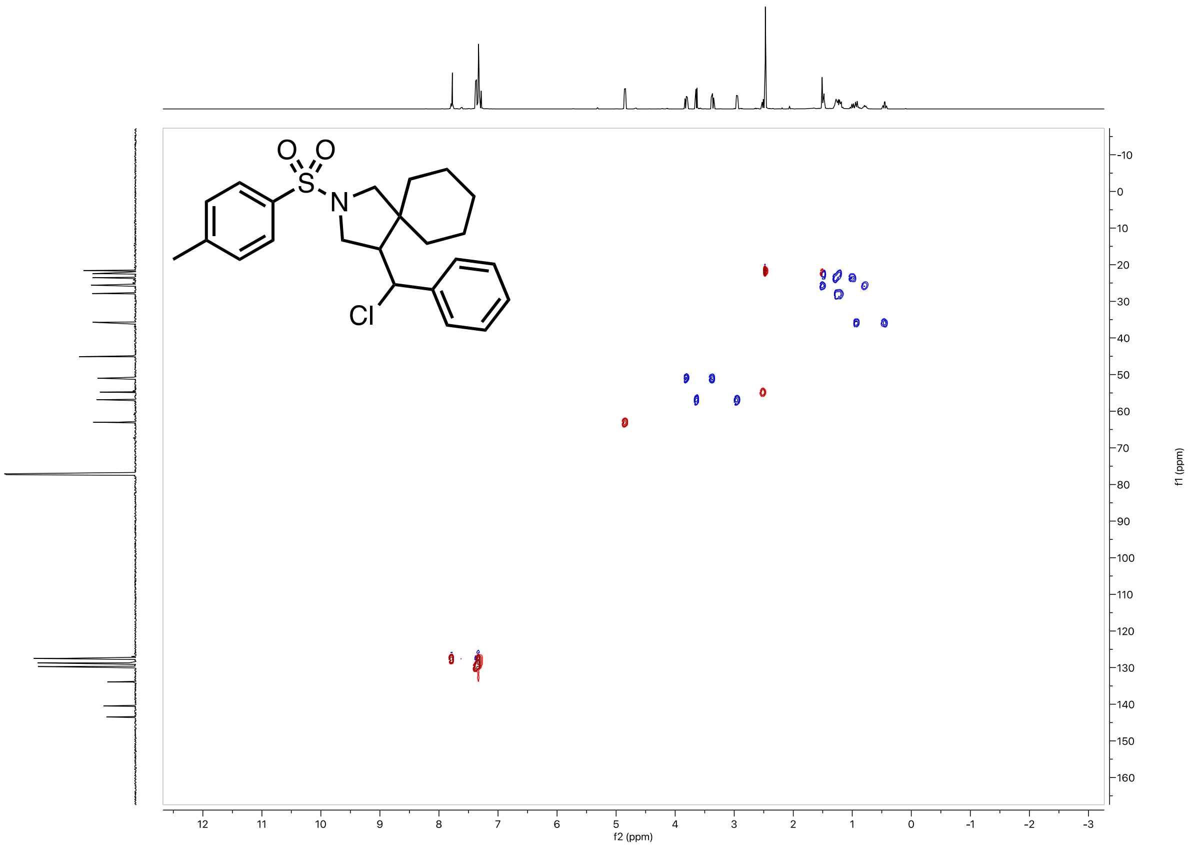

eHSQC of *4-(chloro(phenyl)methyl)-2-tosyl-2-azaspiro[4.5]decane* ($CDCl_3$).

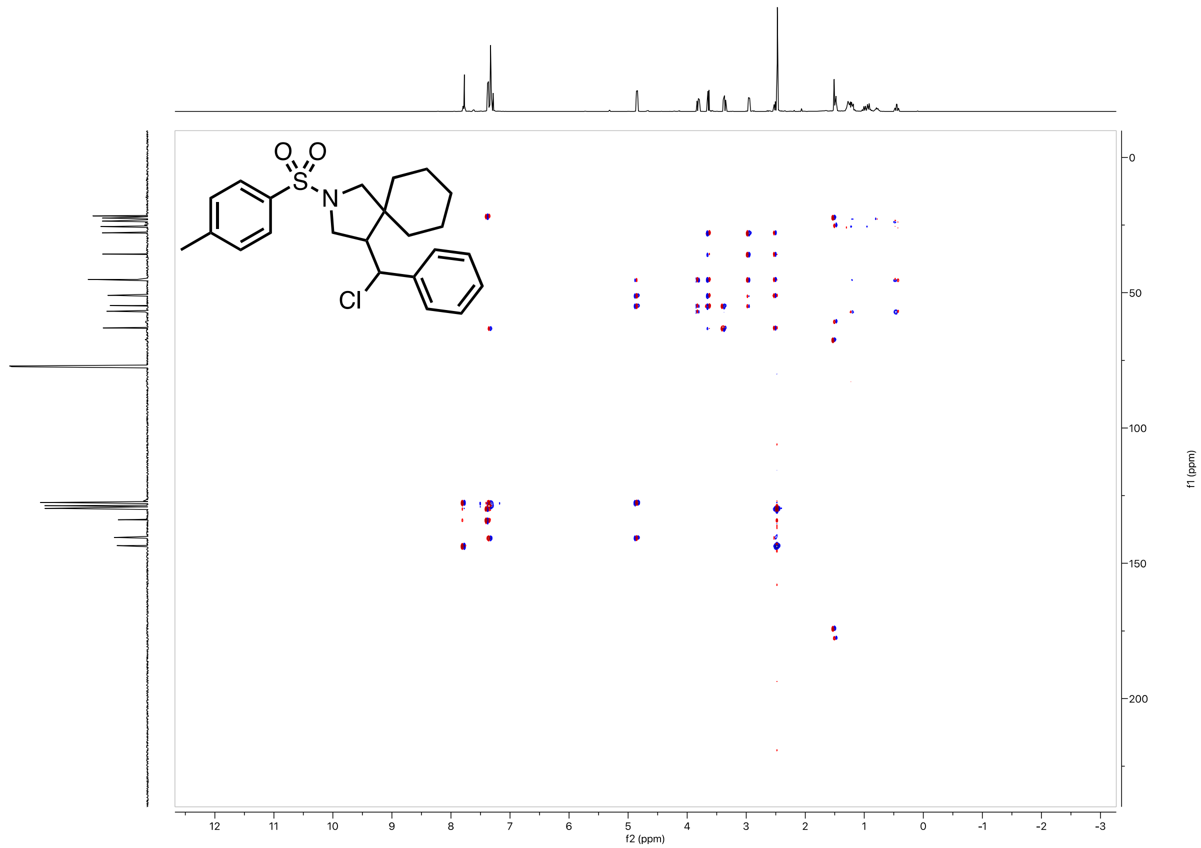

HMBC of *4-(chloro(phenyl)methyl)-2-tosyl-2-azaspiro[4.5]decane* ($CDCl_3$).

4-(1-chloroethyl)-2-tosyl-2-azaspiro[4.5]decane (3i)

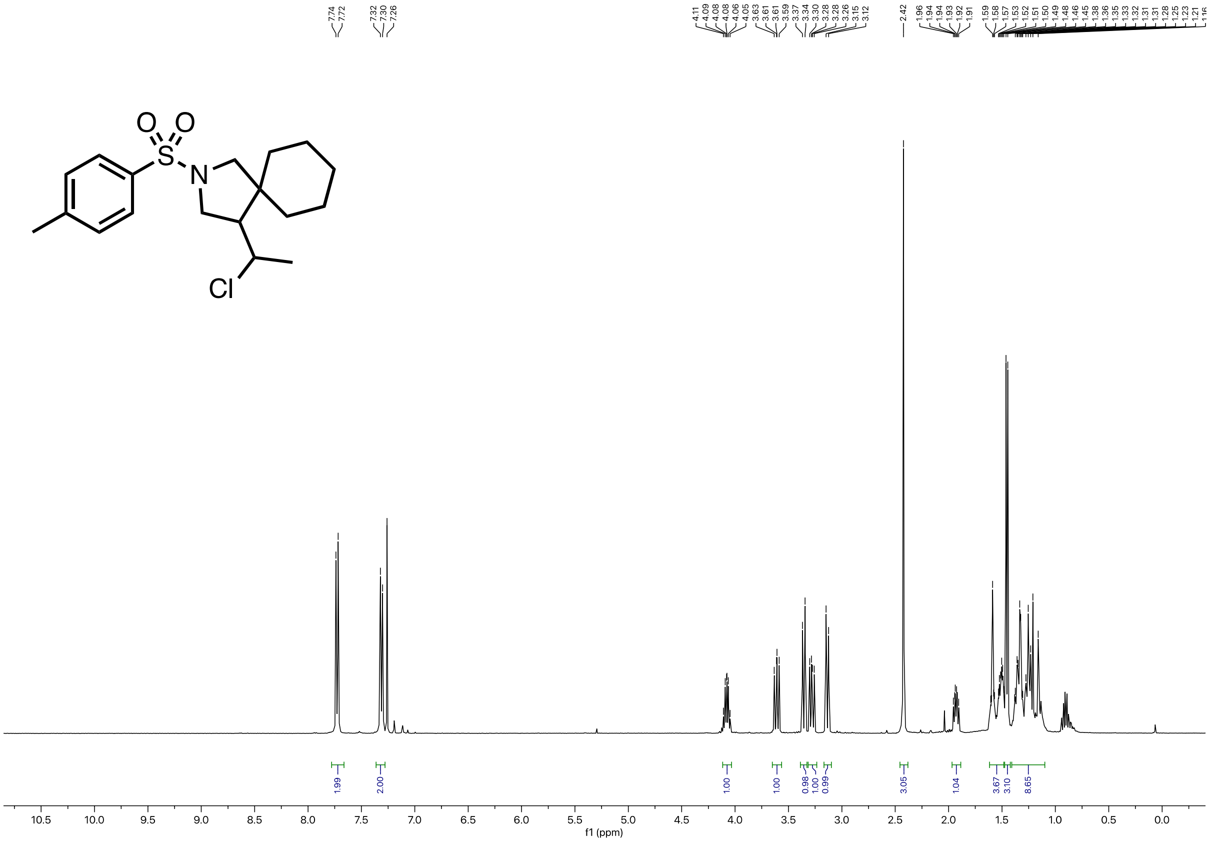

^{1}H NMR of *4-(1-chloroethyl)-2-tosyl-2-azaspiro[4.5]decane (Major Diastereomer)* (400 MHz, $CDCl_3$).

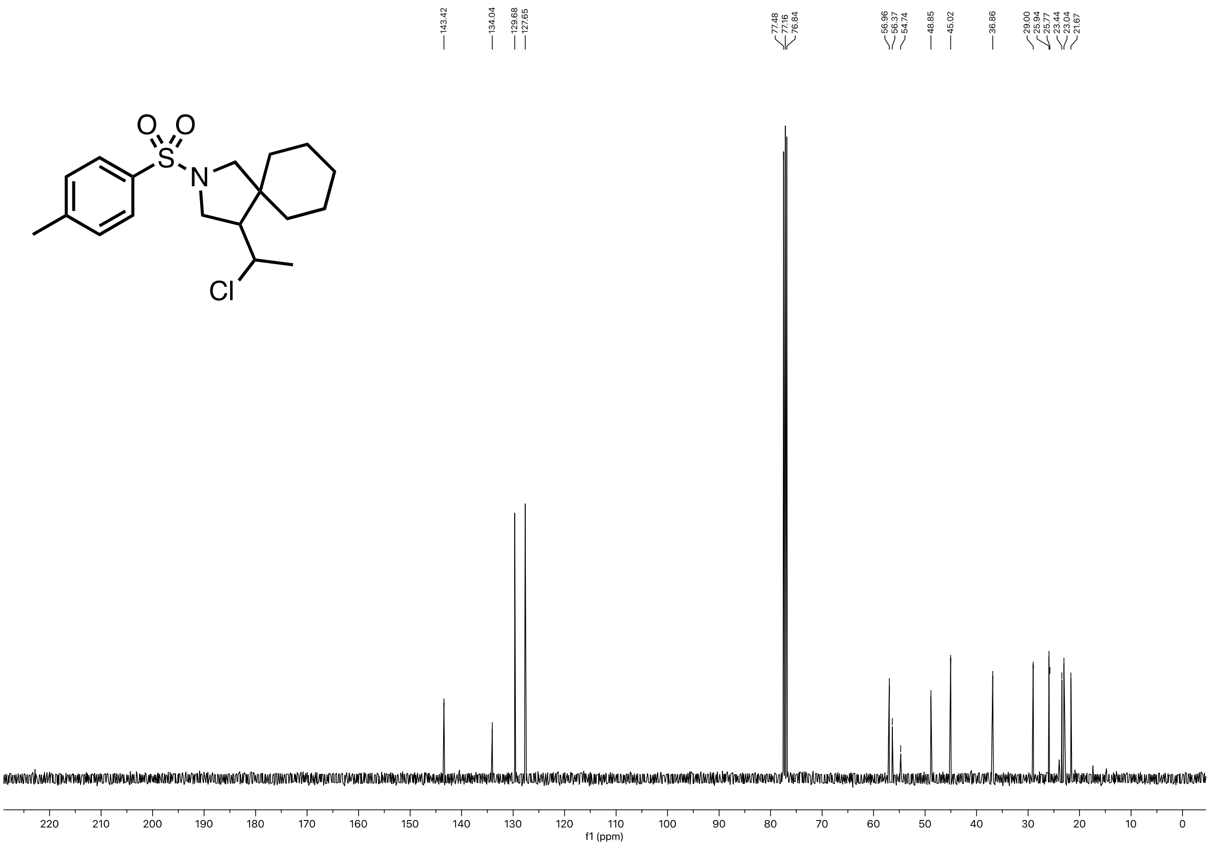

$^{13}C\{^{1}H\}$ NMR of *4-(1-chloroethyl)-2-tosyl-2-azaspiro[4.5]decane (Major Diastereomer)* (101 MHz, $CDCl_3$).

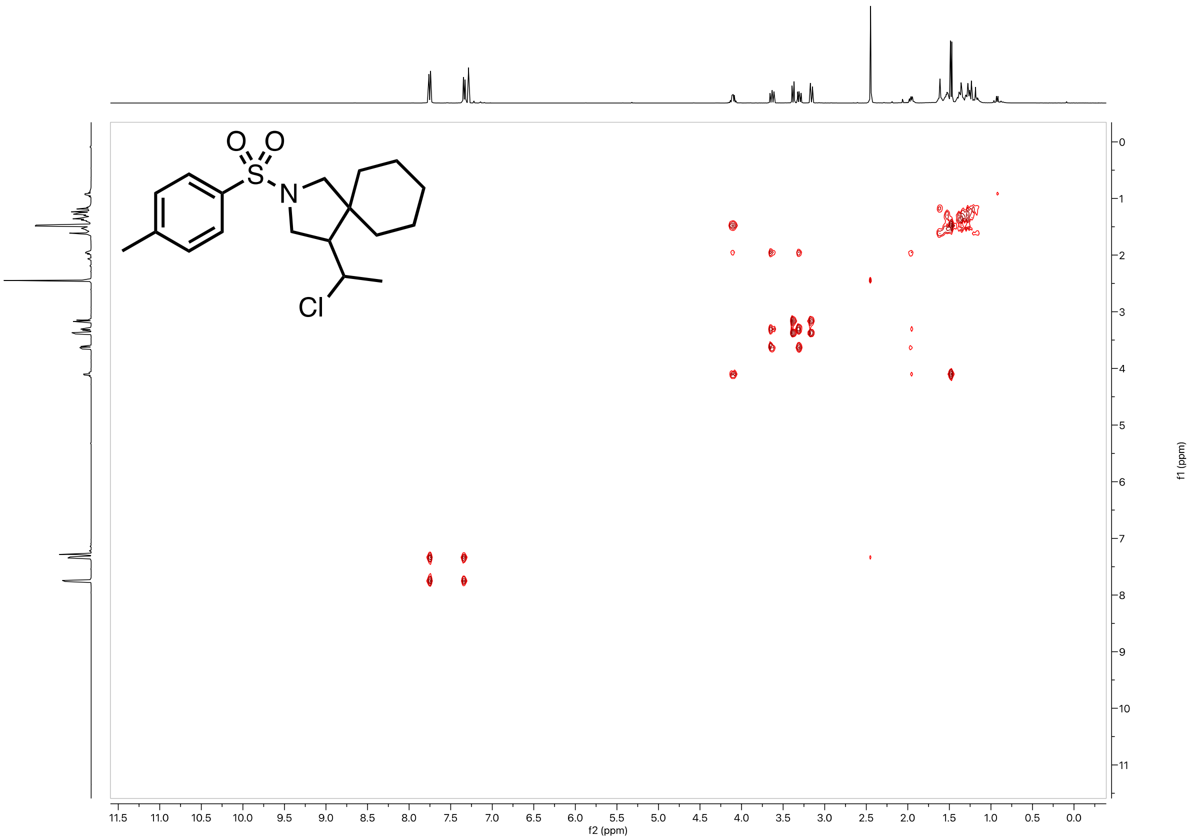

COSY of *4-(1-chloroethyl)-2-tosyl-2-azaspiro[4.5]decane (Major Diastereomer)* ($CDCl_3$).

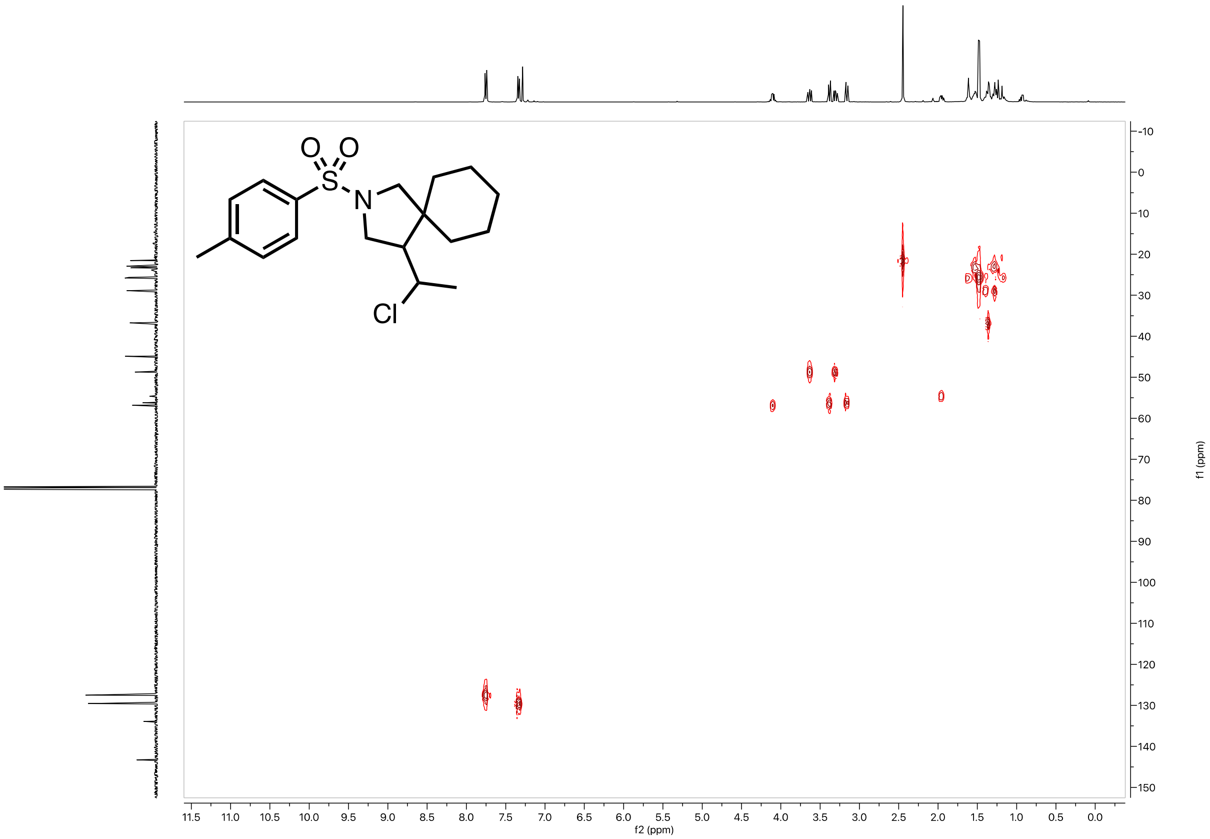

HSQC of *4-(1-chloroethyl)-2-tosyl-2-azaspiro[4.5]decane (Major Diastereomer)* ($CDCl_3$).

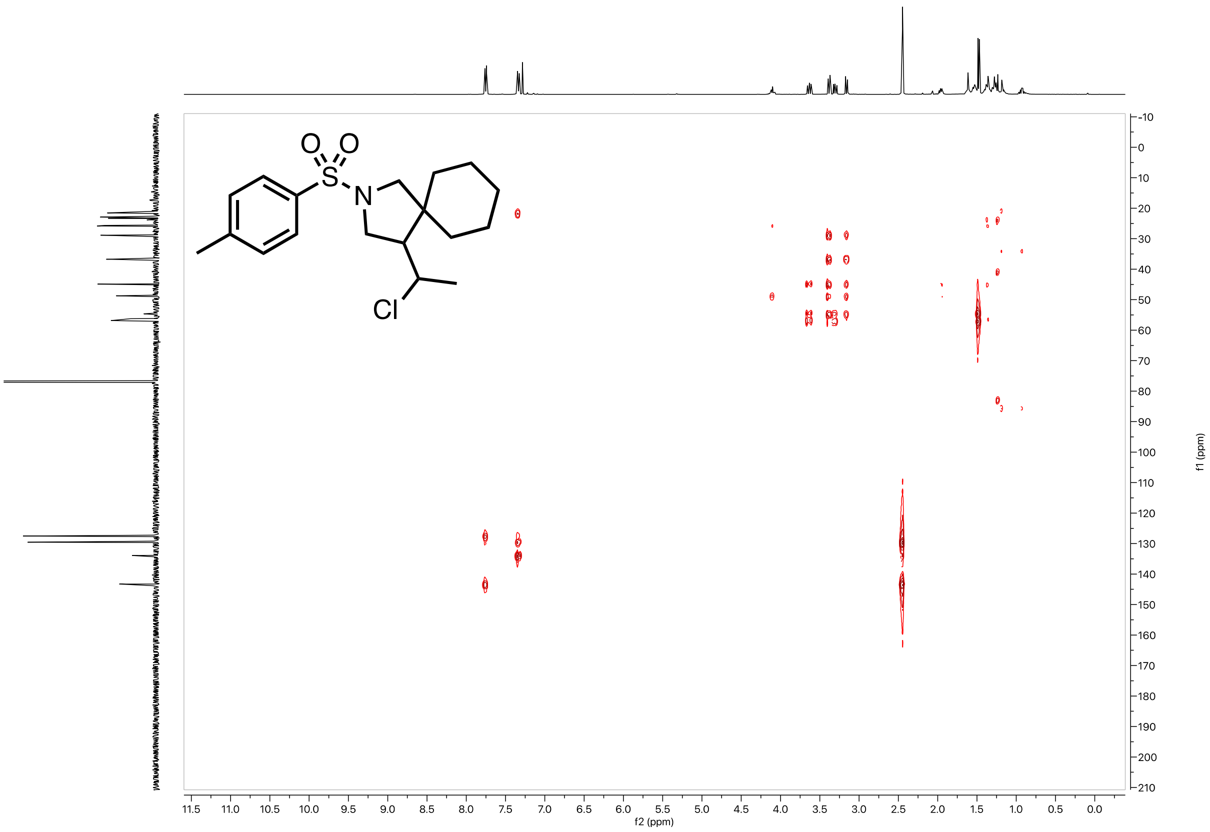

HMBC of *4-(1-chloroethyl)-2-tosyl-2-azaspiro[4.5]decane (Major Diastereomer)* ($CDCl_3$).

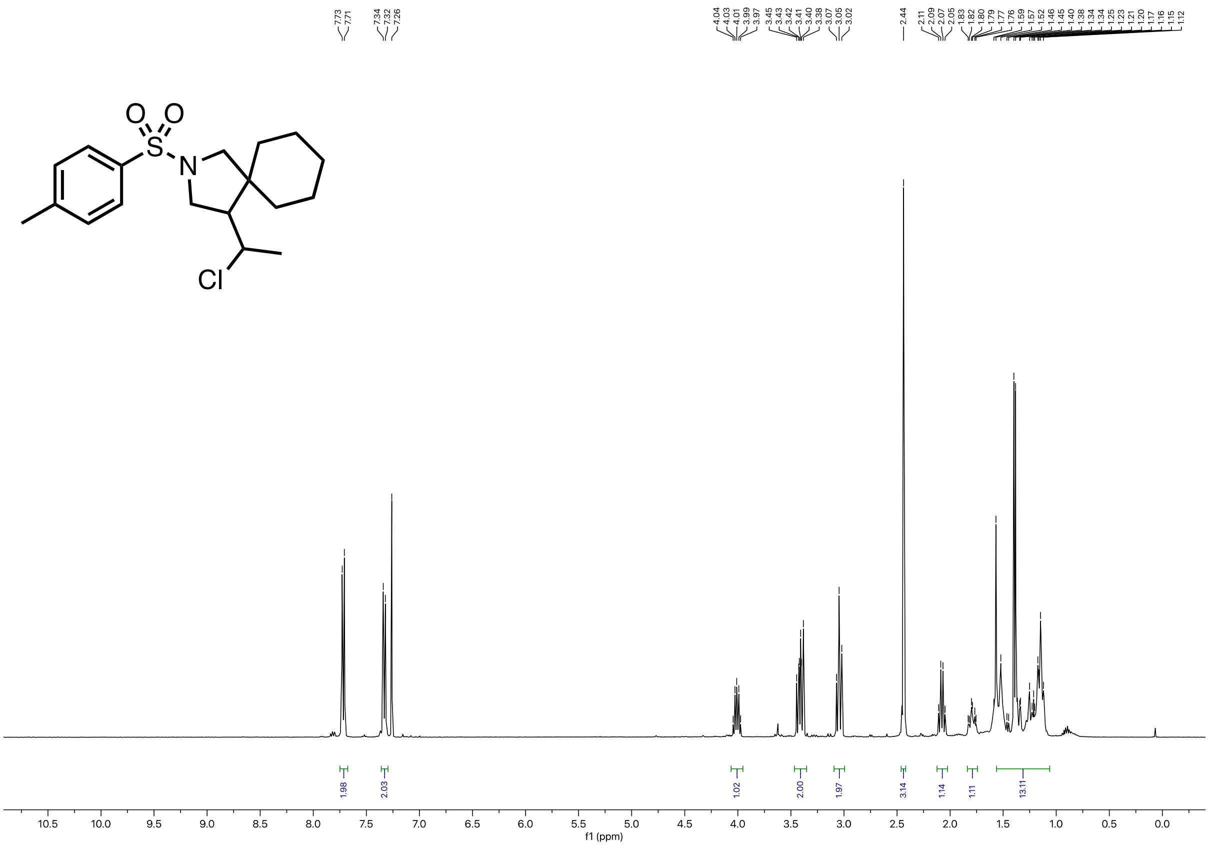

^{1}H NMR of *4-(1-chloroethyl)-2-tosyl-2-azaspiro[4.5]decane (Minor Diastereomer)* (400 MHz, $CDCl_3$).

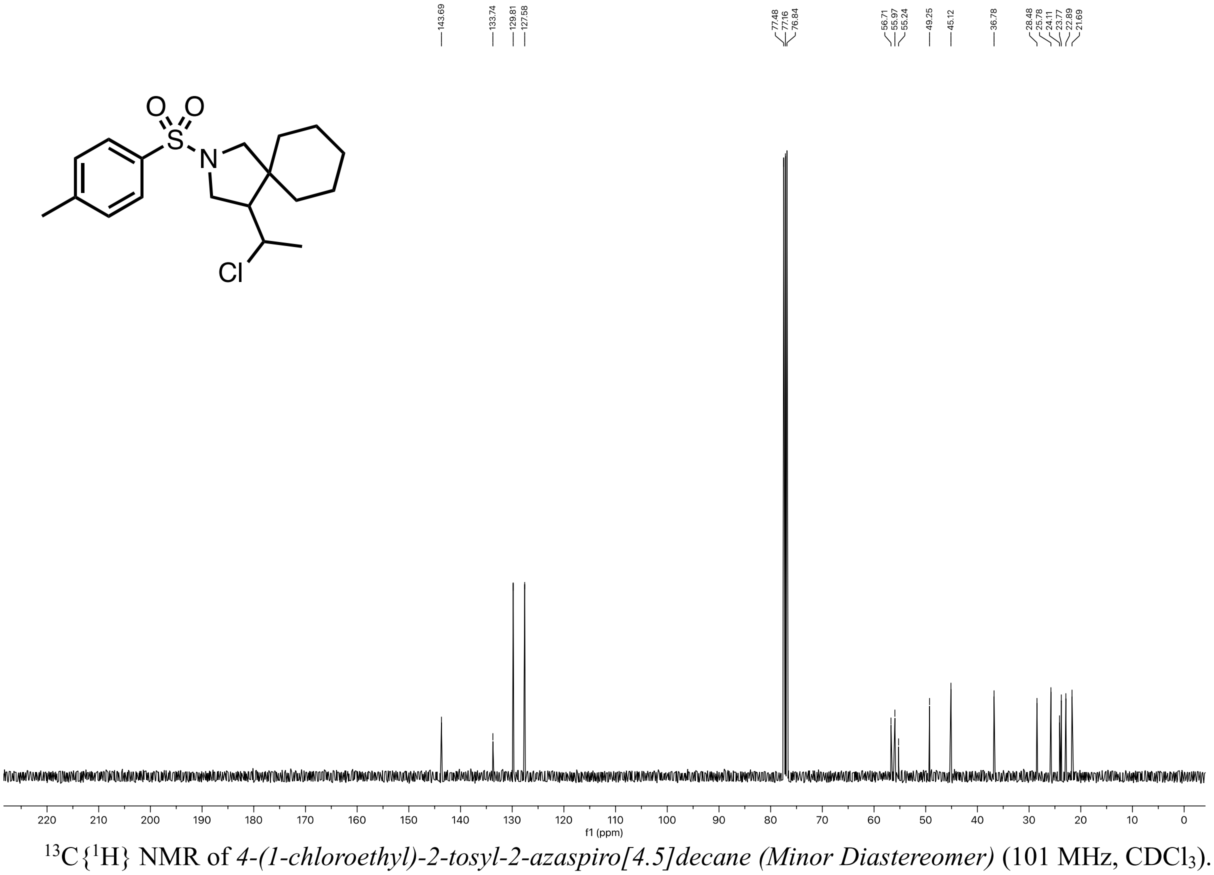

$^{13}C\{^1H\}$ NMR of *4-(1-chloroethyl)-2-tosyl-2-azaspiro[4.5]decane (Minor Diastereomer)* (101 MHz, $CDCl_3$).

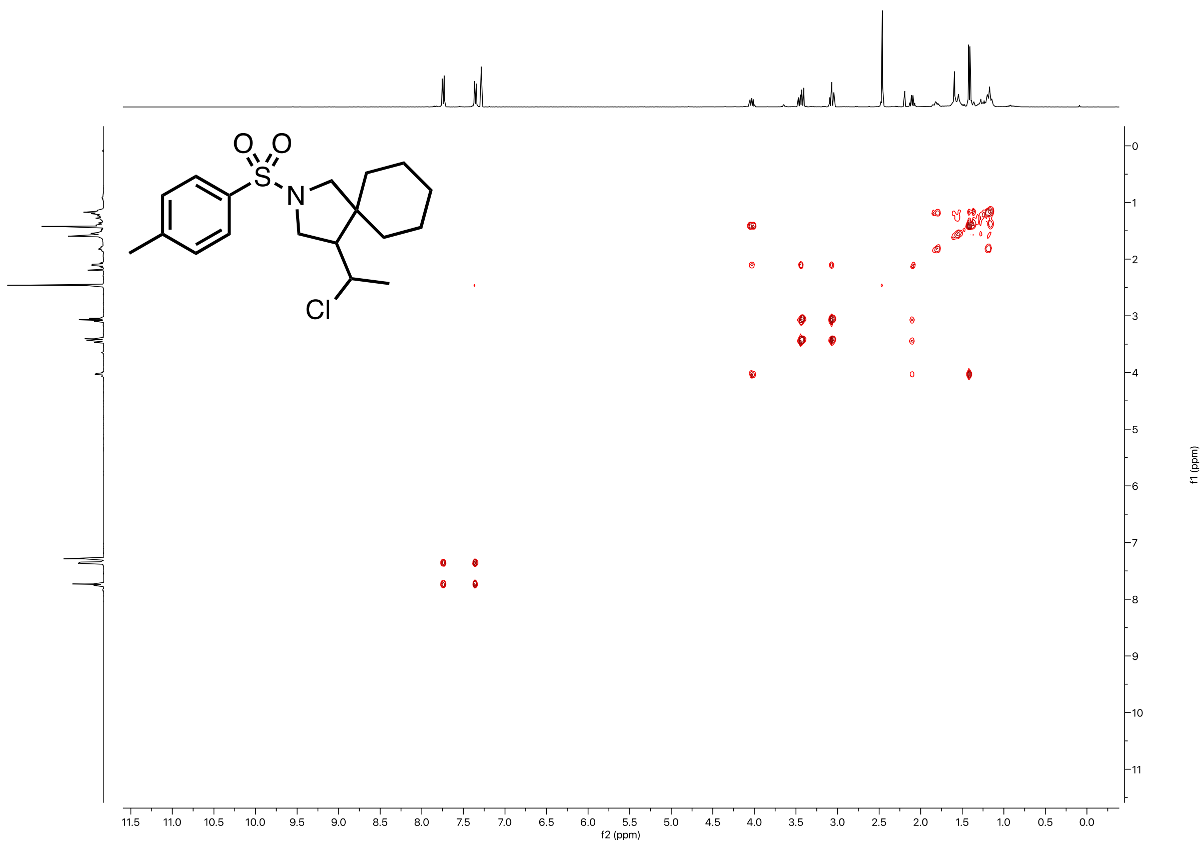

COSY of *4-(1-chloroethyl)-2-tosyl-2-azaspiro[4.5]decane (Minor Diastereomer)* ($CDCl_3$).

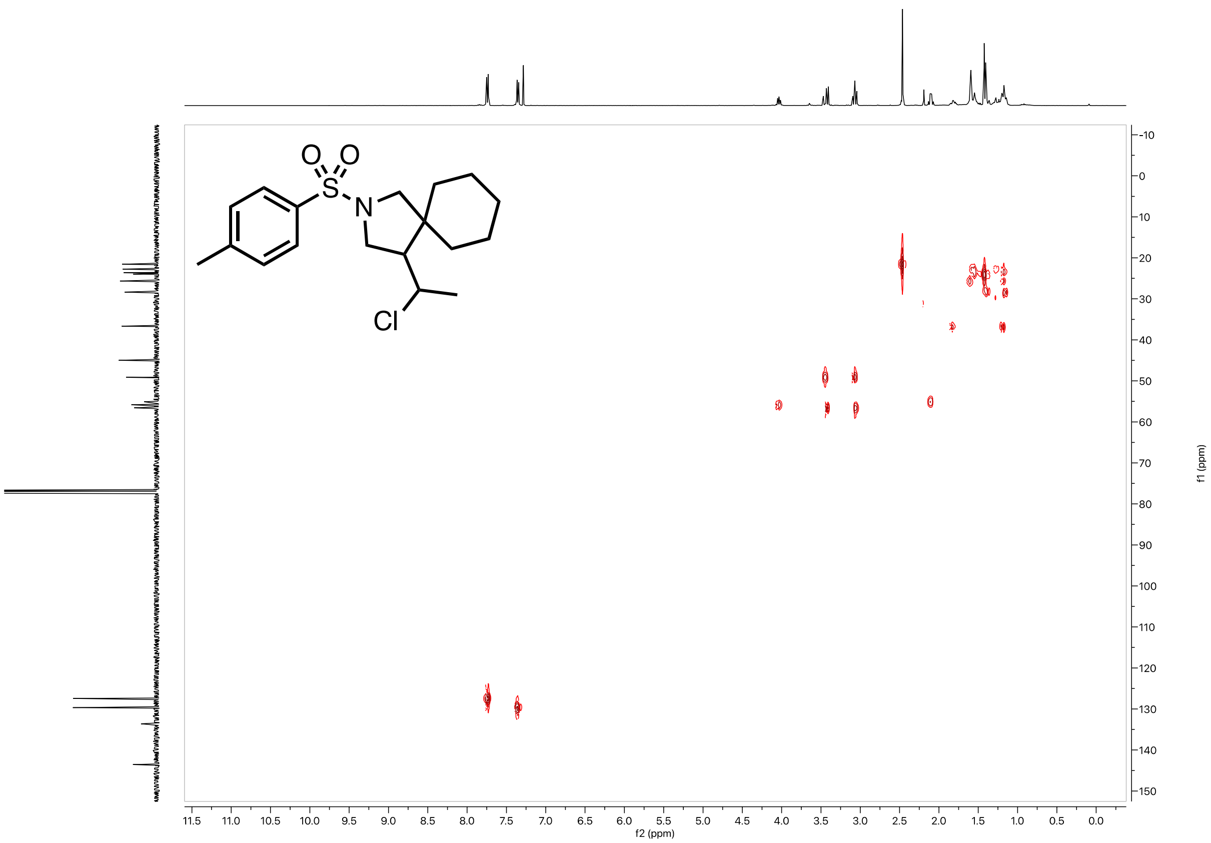

HSQC of *4-(1-chloroethyl)-2-tosyl-2-azaspiro[4.5]decane (Minor Diastereomer)* ($CDCl_3$).

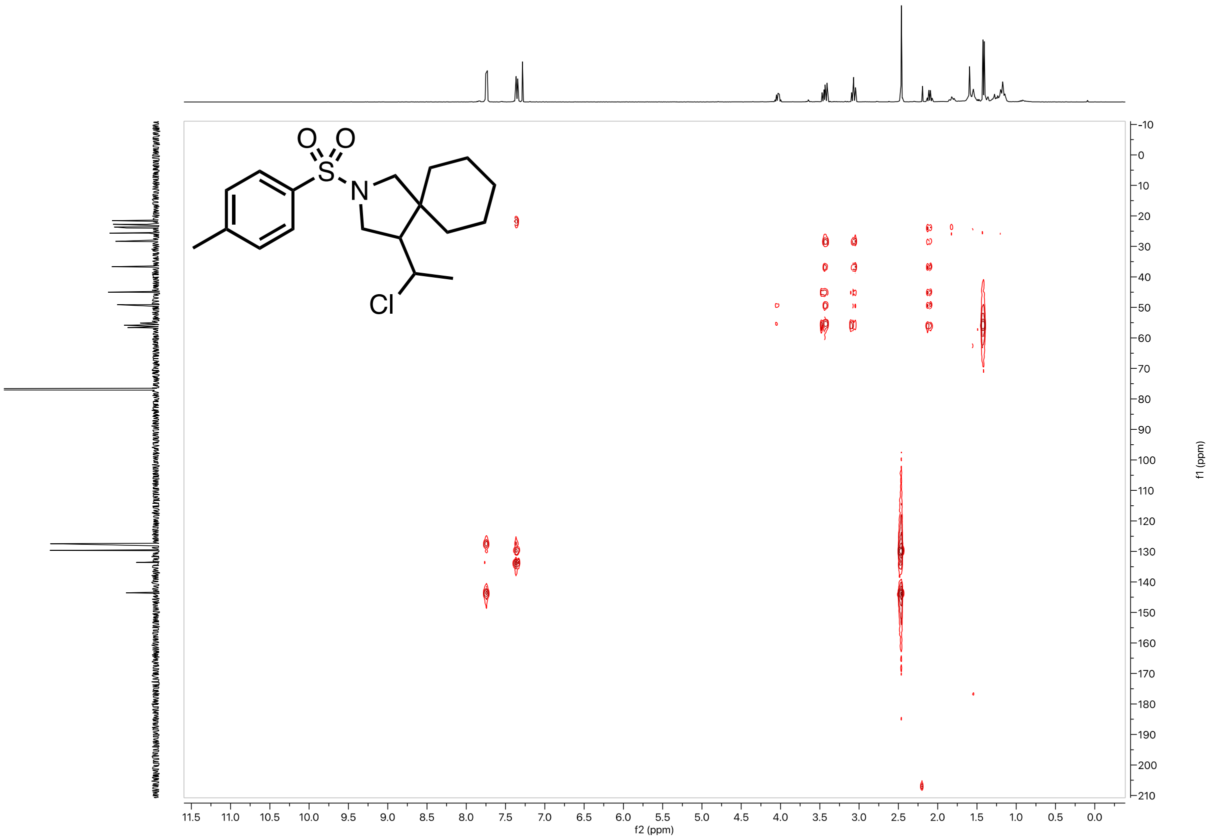

HMBC of *4-(1-chloroethyl)-2-tosyl-2-azaspiro[4.5]decane (Minor Diastereomer)* ($CDCl_3$).

4-(bromomethyl)-2-tosyl-2-azaspiro[4.5]decane (3j)

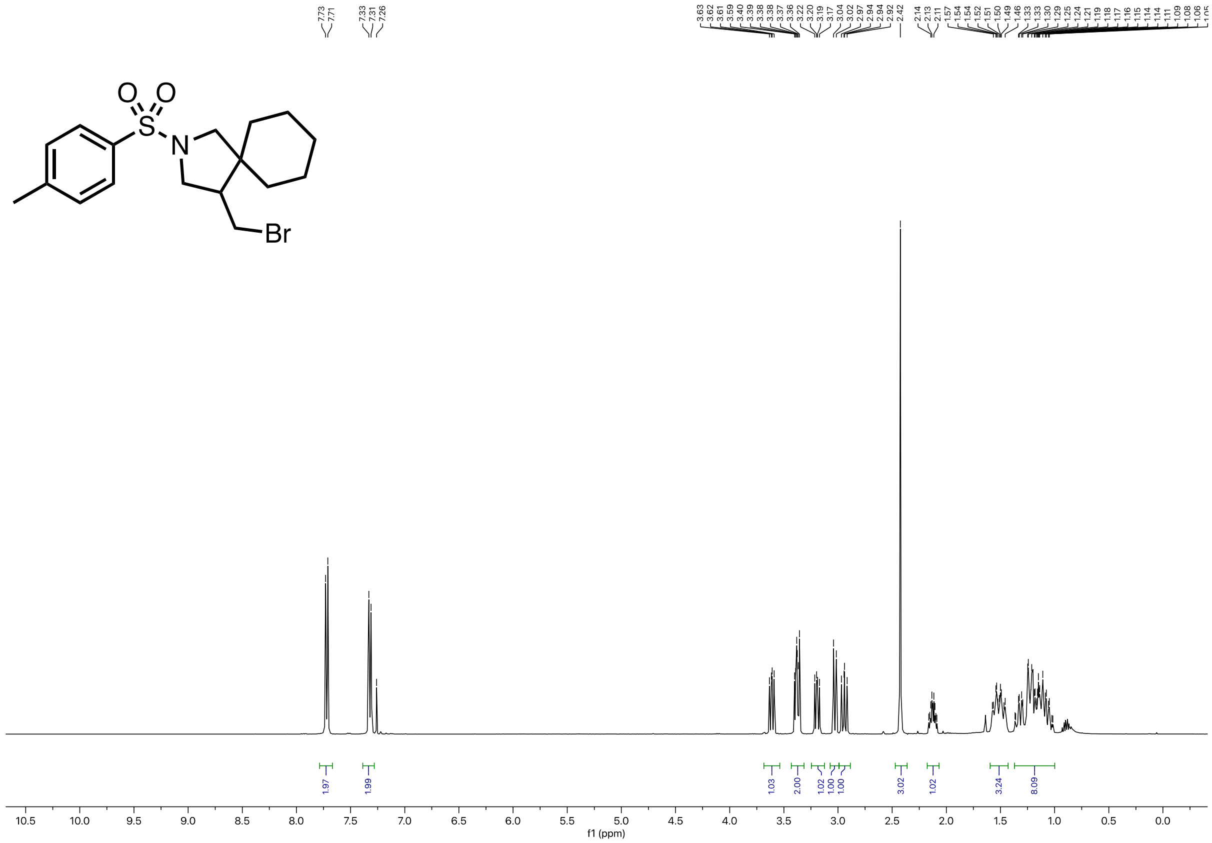

^{1}H NMR of *4-(bromomethyl)-2-tosyl-2-azaspiro[4.5]decane* (400 MHz, $CDCl_3$).

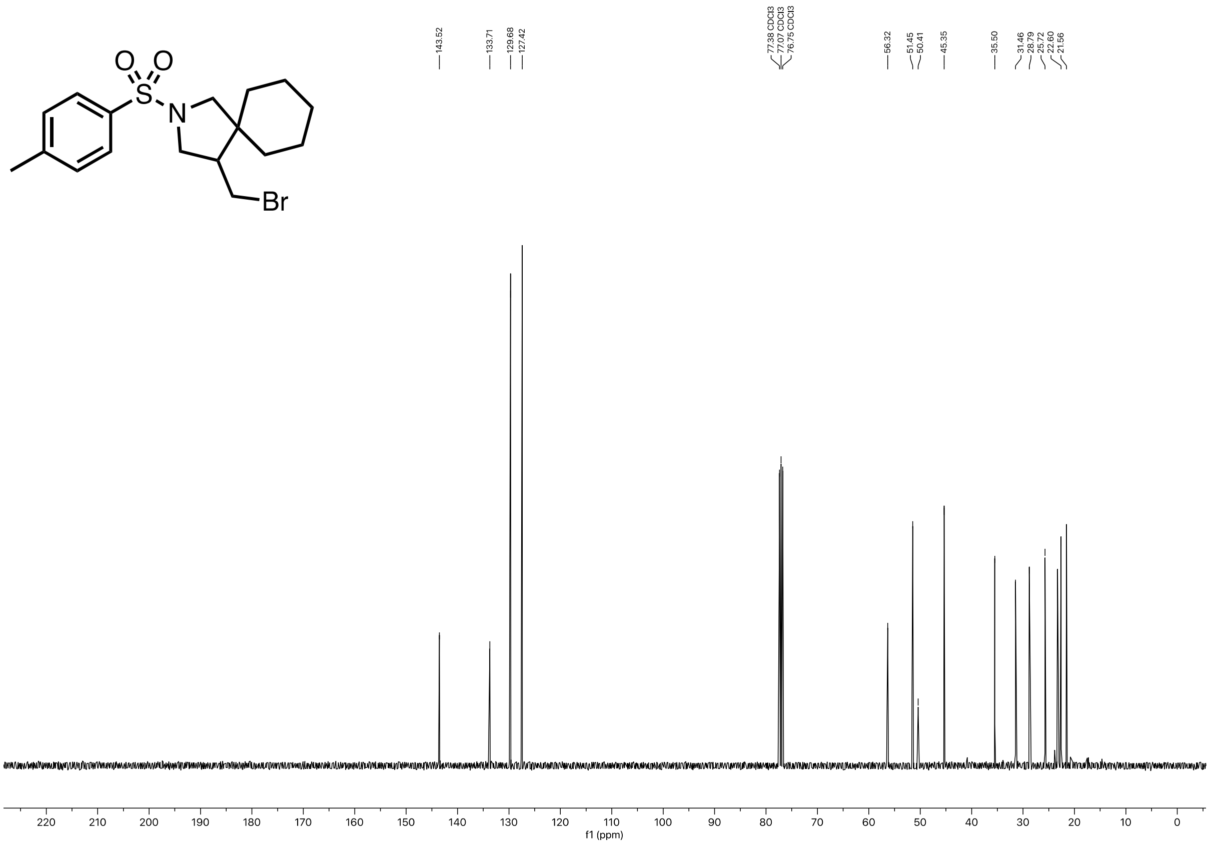

$^{13}C\{^{1}H\}$ NMR of *4-(bromomethyl)-2-tosyl-2-azaspiro[4.5]decane* (101 MHz, $CDCl_3$).

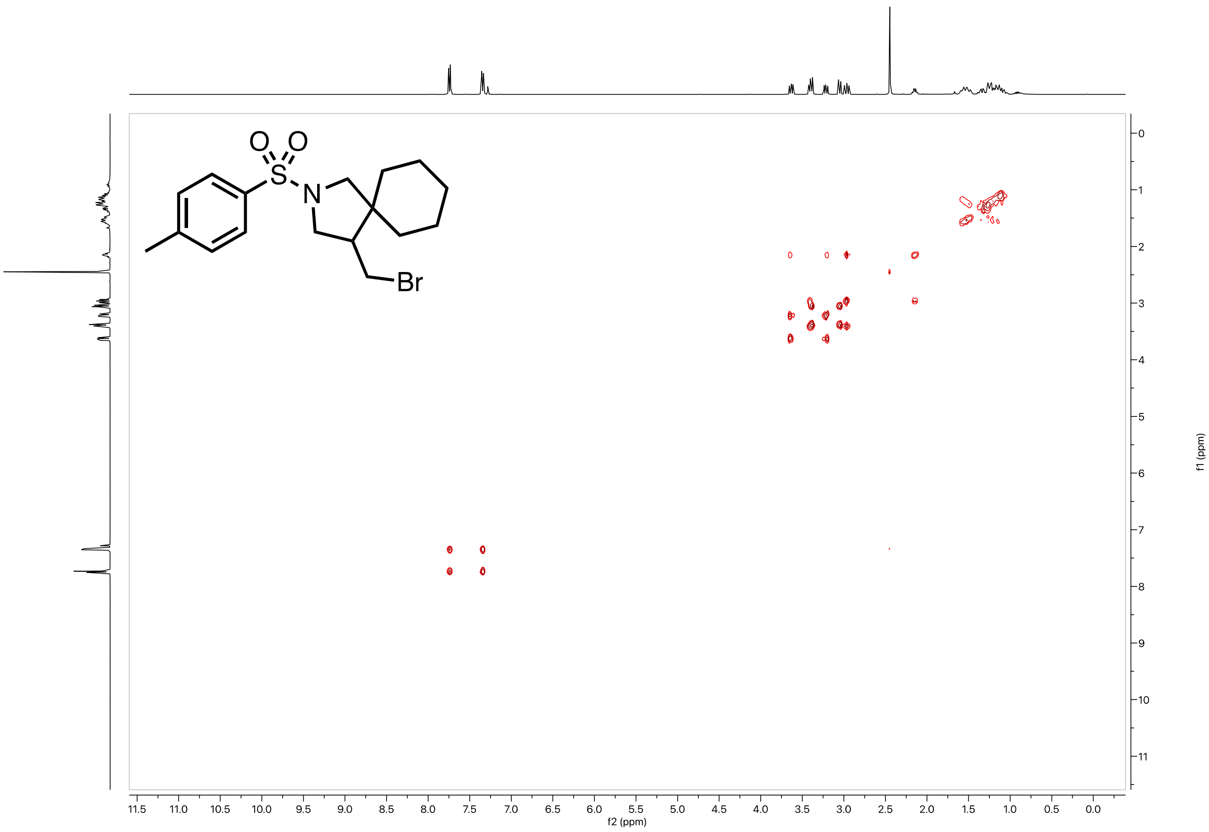

COSY of *4-(bromomethyl)-2-tosyl-2-azaspiro[4.5]decane* ($CDCl_3$).

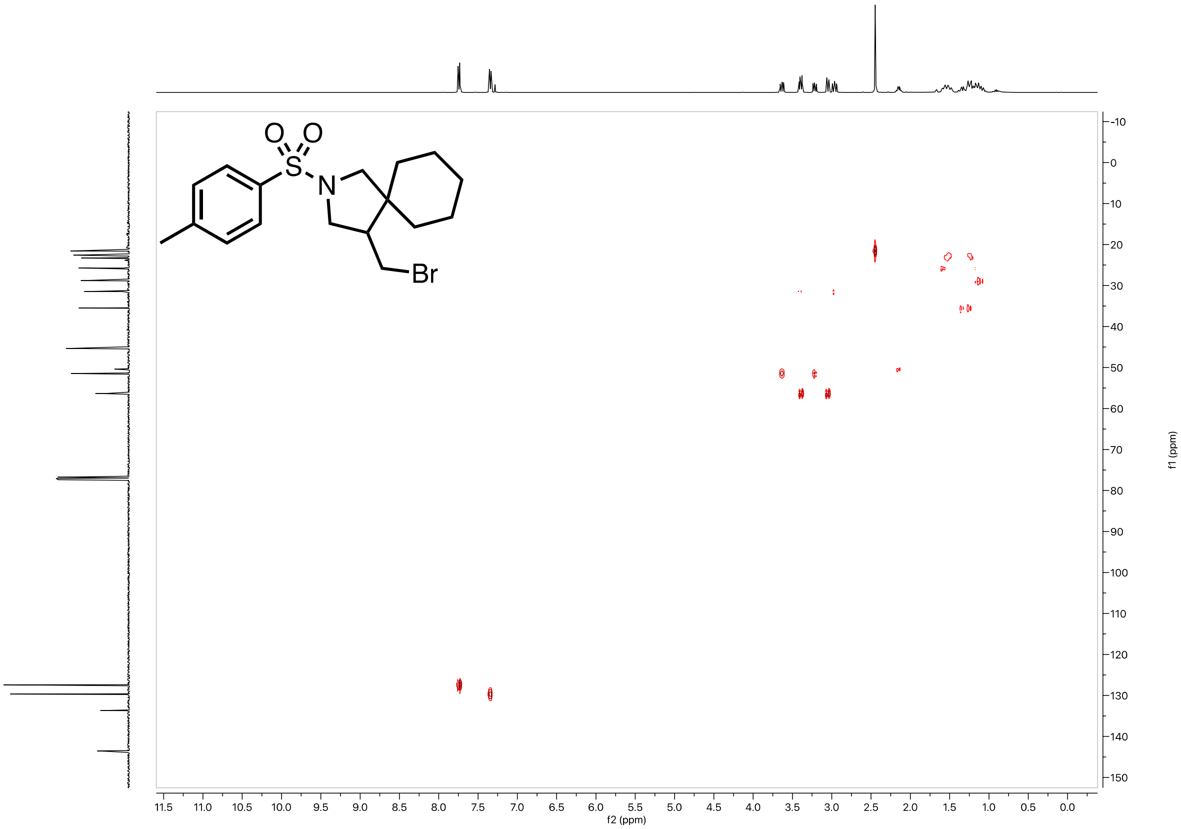

HSQC of *4-(bromomethyl)-2-tosyl-2-azaspiro[4.5]decane* ($CDCl_3$).

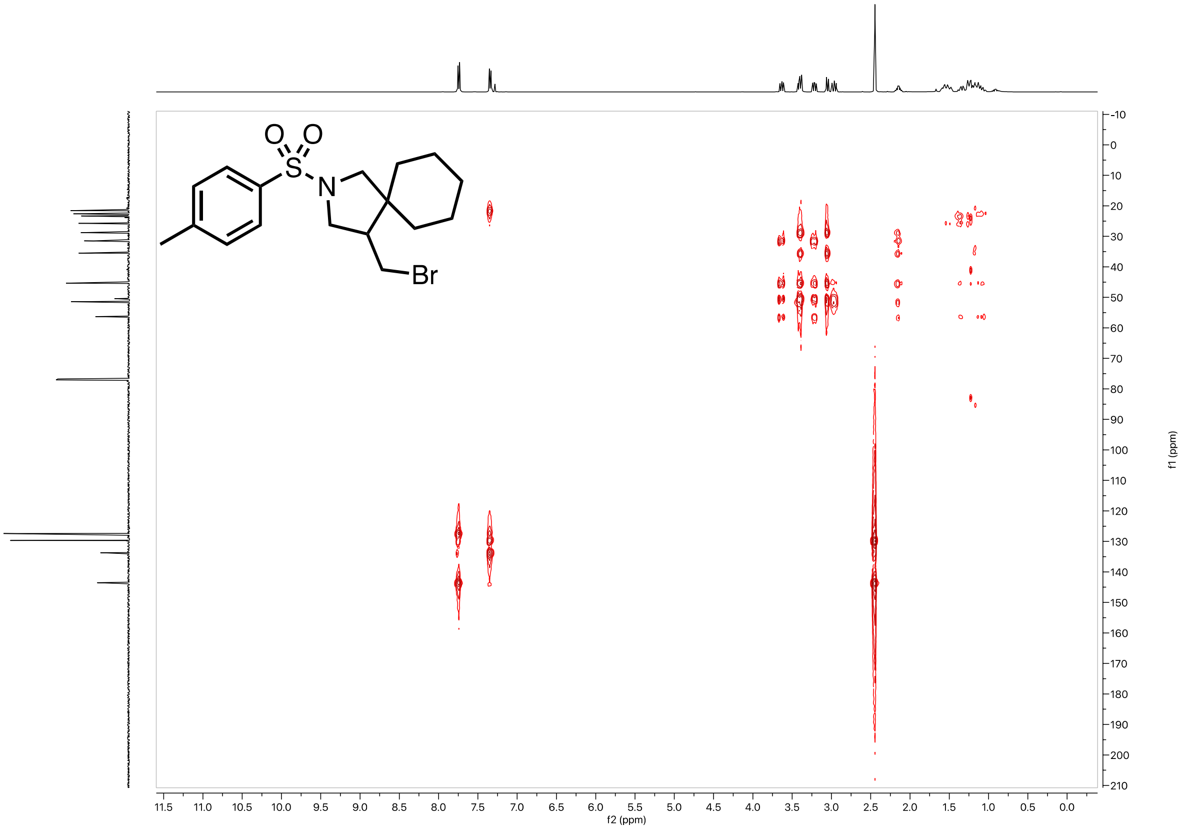

HMBC of *4-(bromomethyl)-2-tosyl-2-azaspiro[4.5]decane* ($CDCl_3$).

4-(iodomethyl)-2-tosyl-2-azaspiro[4.5]decane (3k)

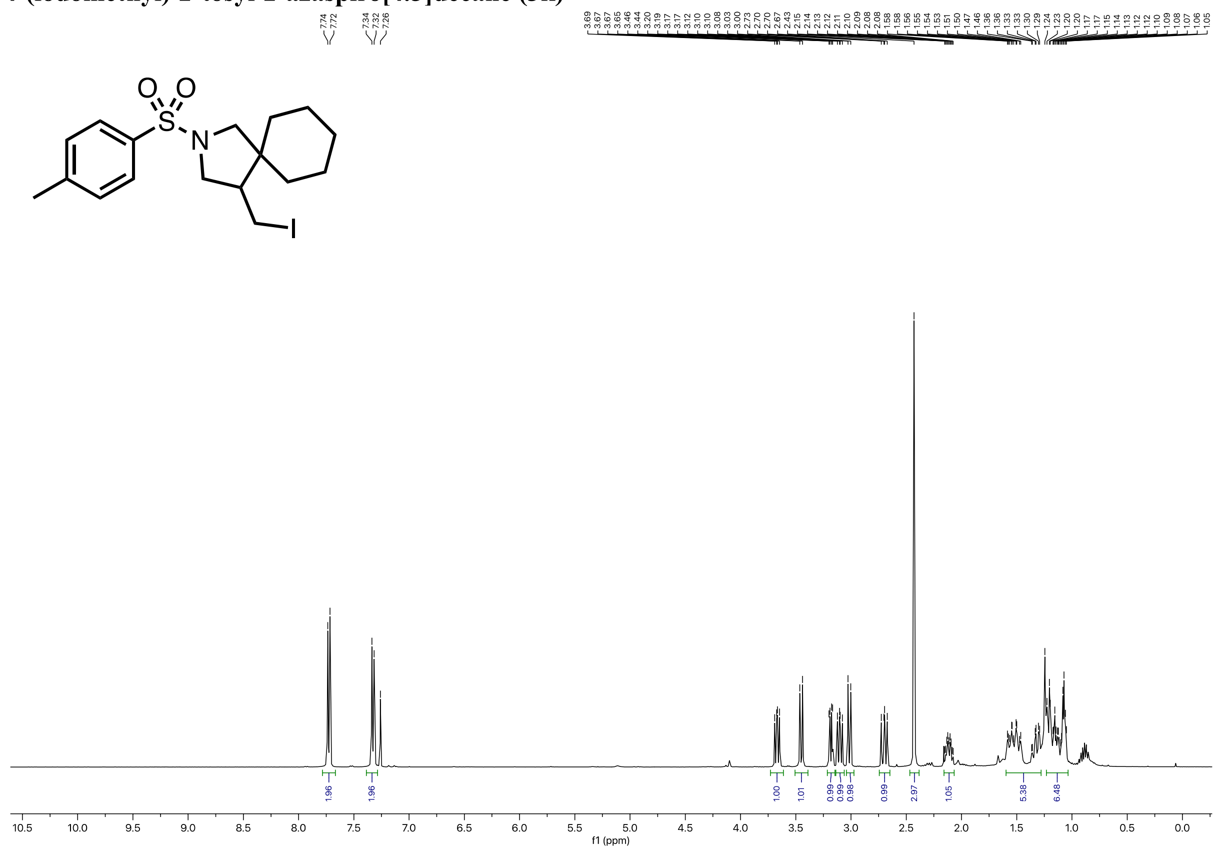

^{1}H NMR of *4-(iodomethyl)-2-tosyl-2-azaspiro[4.5]decane* (400 MHz, $CDCl_3$).

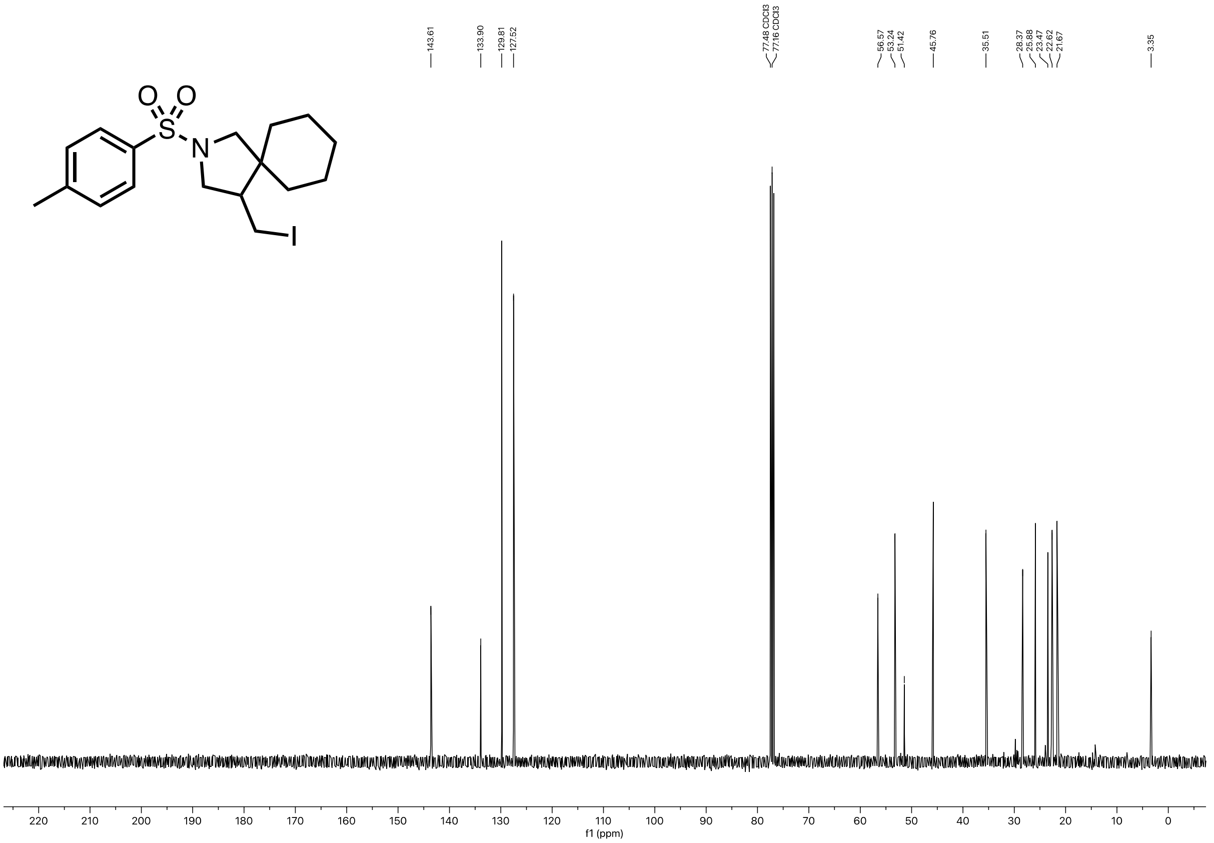

$^{13}C\{^{1}H\}$ NMR of *4-(iodomethyl)-2-tosyl-2-azaspiro[4.5]decane* (101 MHz, $CDCl_3$).

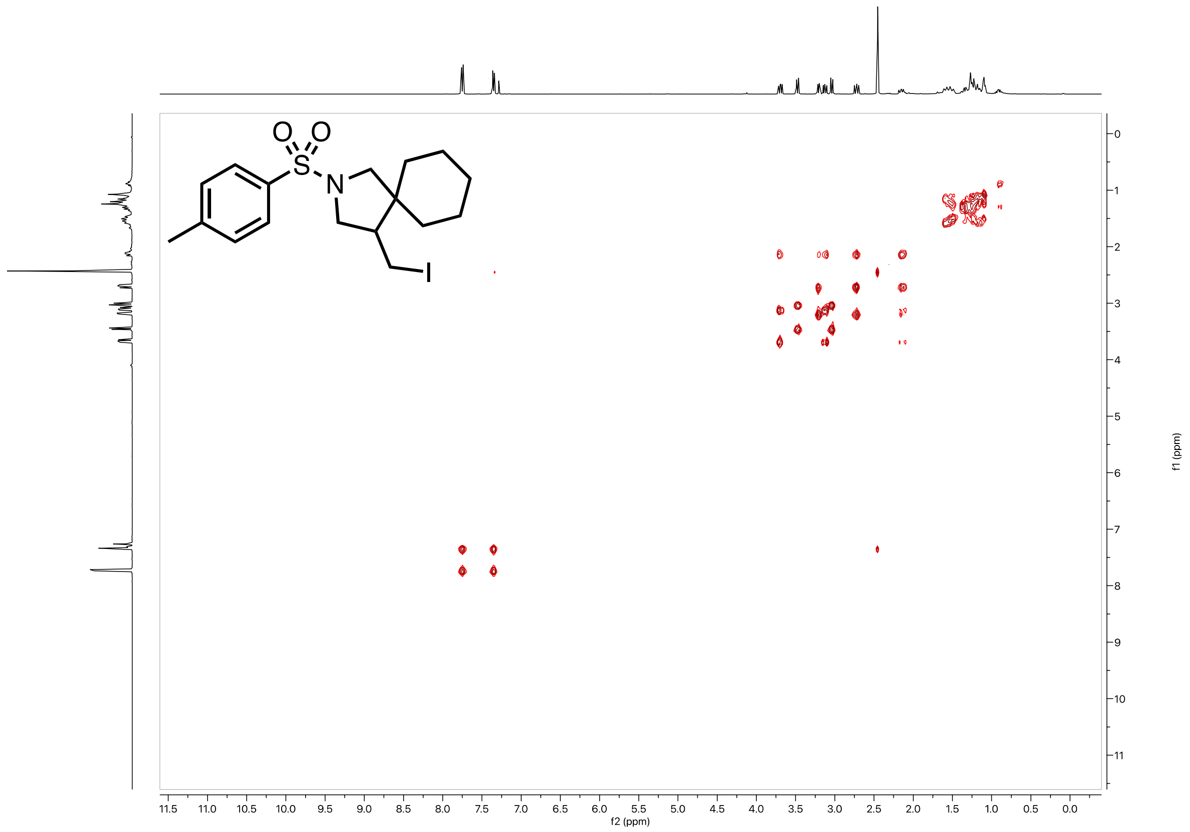

COSY of *4-(iodomethyl)-2-tosyl-2-azaspiro[4.5]decane* ($CDCl_3$).

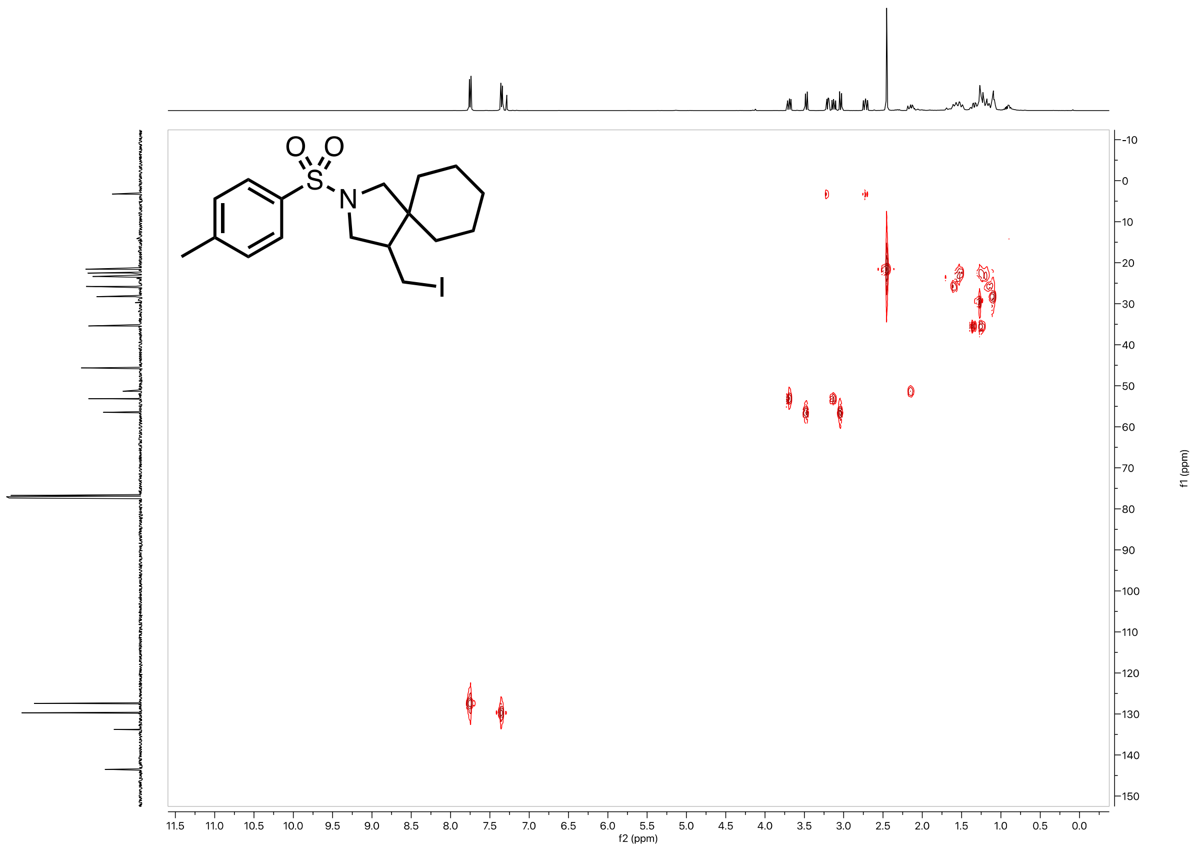

HSQC of *4-(iodomethyl)-2-tosyl-2-azaspiro[4.5]decane* ($CDCl_3$).

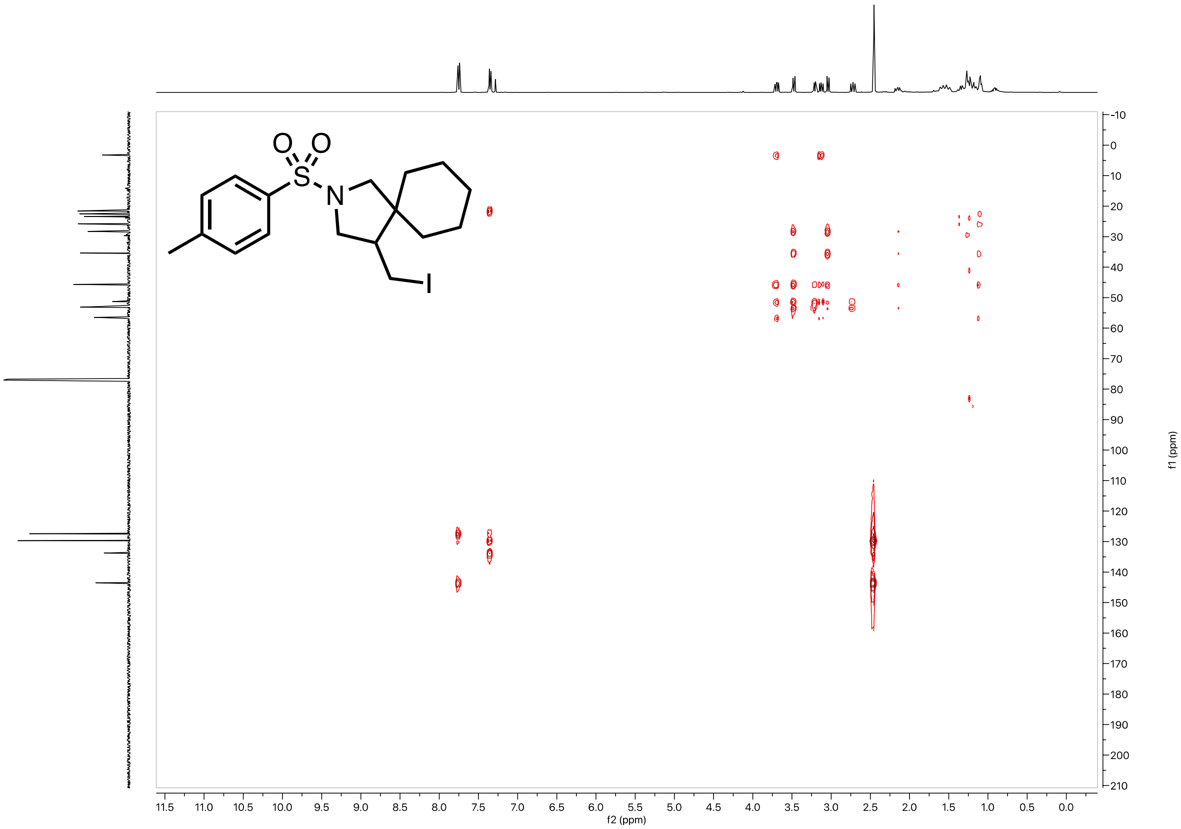

HMBC of *4-(iodomethyl)-2-tosyl-2-azaspiro[4.5]decane* ($CDCl_3$).

8-(tert-butyl)-4-(chloromethyl)-2-tosyl-2-azaspiro[4.5]decane (3m)

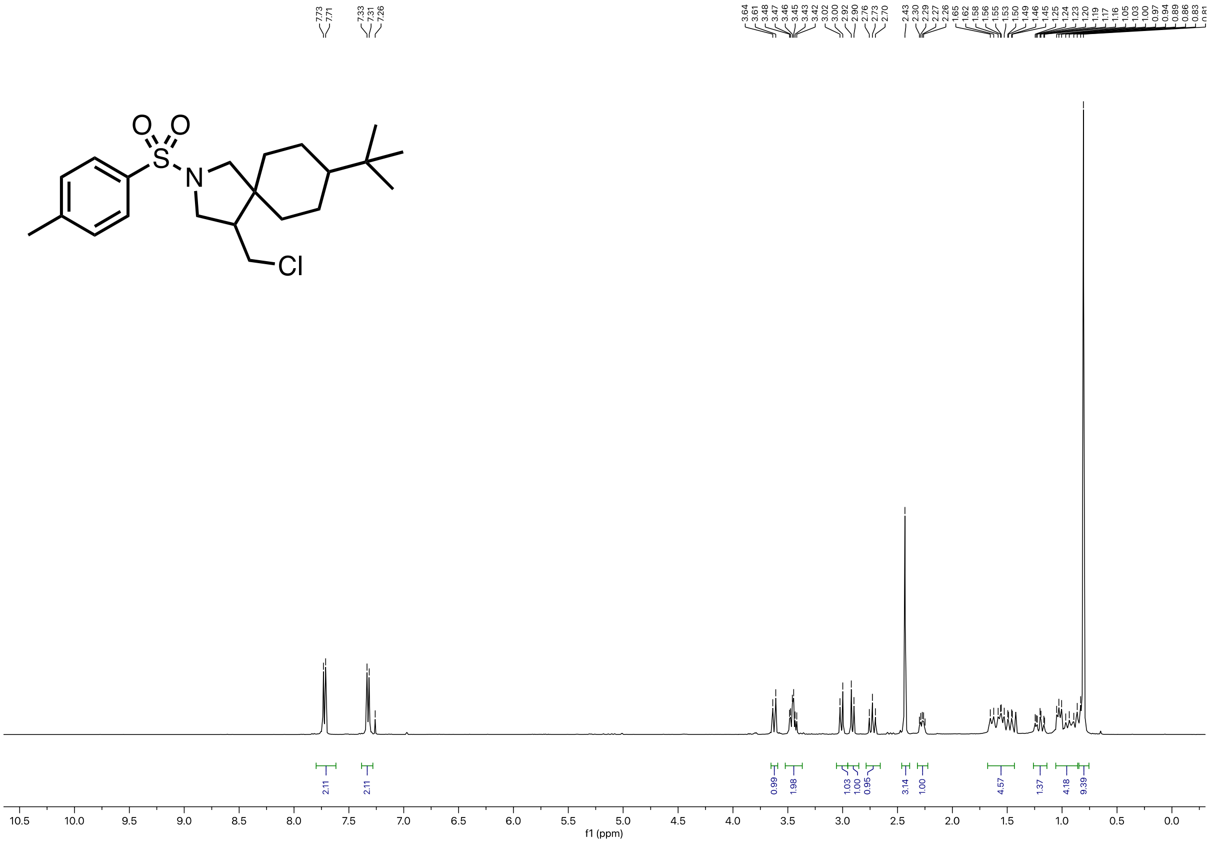

^{1}H NMR of *8-(tert-butyl)-4-(chloromethyl)-2-tosyl-2-azaspiro[4.5]decane (Major Diastereomer)* (400 MHz, $CDCl_3$).

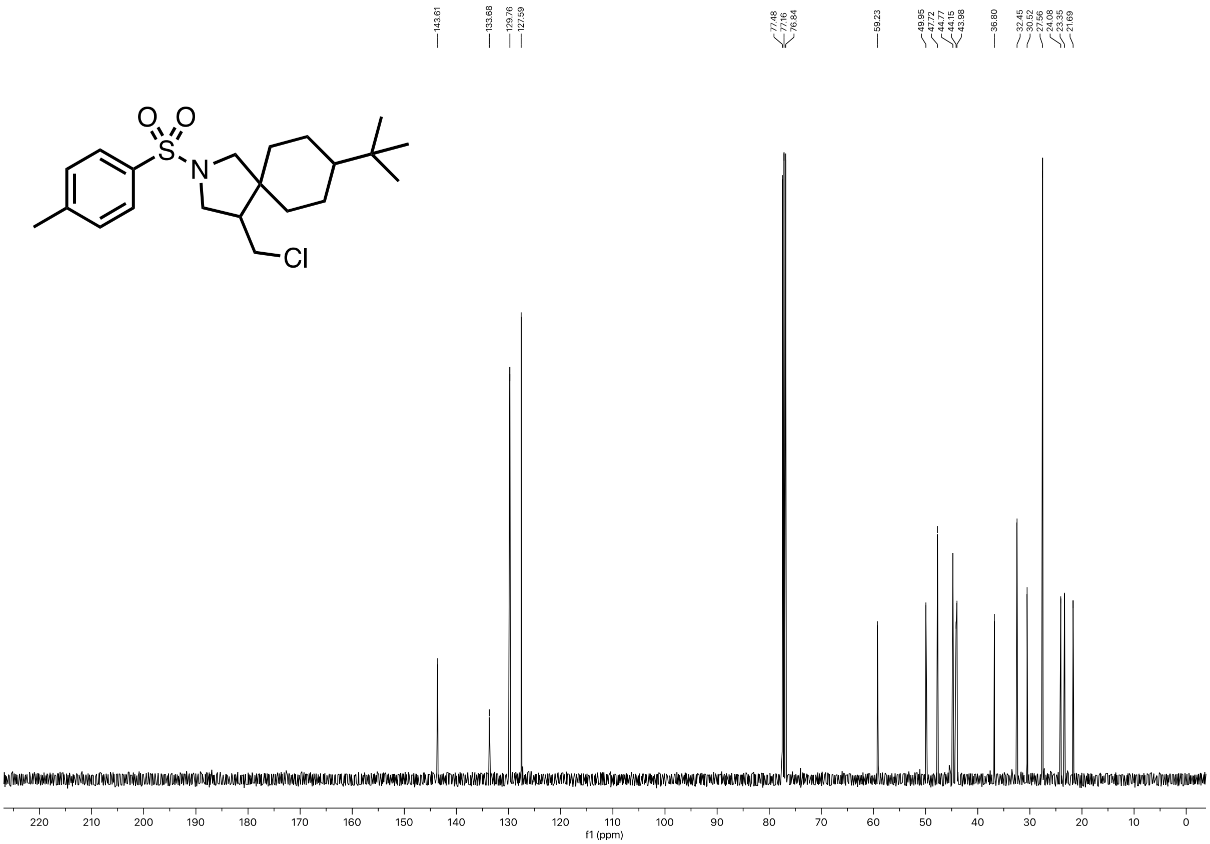

$^{13}C\{^{1}H\}$ NMR of *8-(tert-butyl)-4-(chloromethyl)-2-tosyl-2-azaspiro[4.5]decane (Major Diastereomer)* (101 MHz, $CDCl_3$).

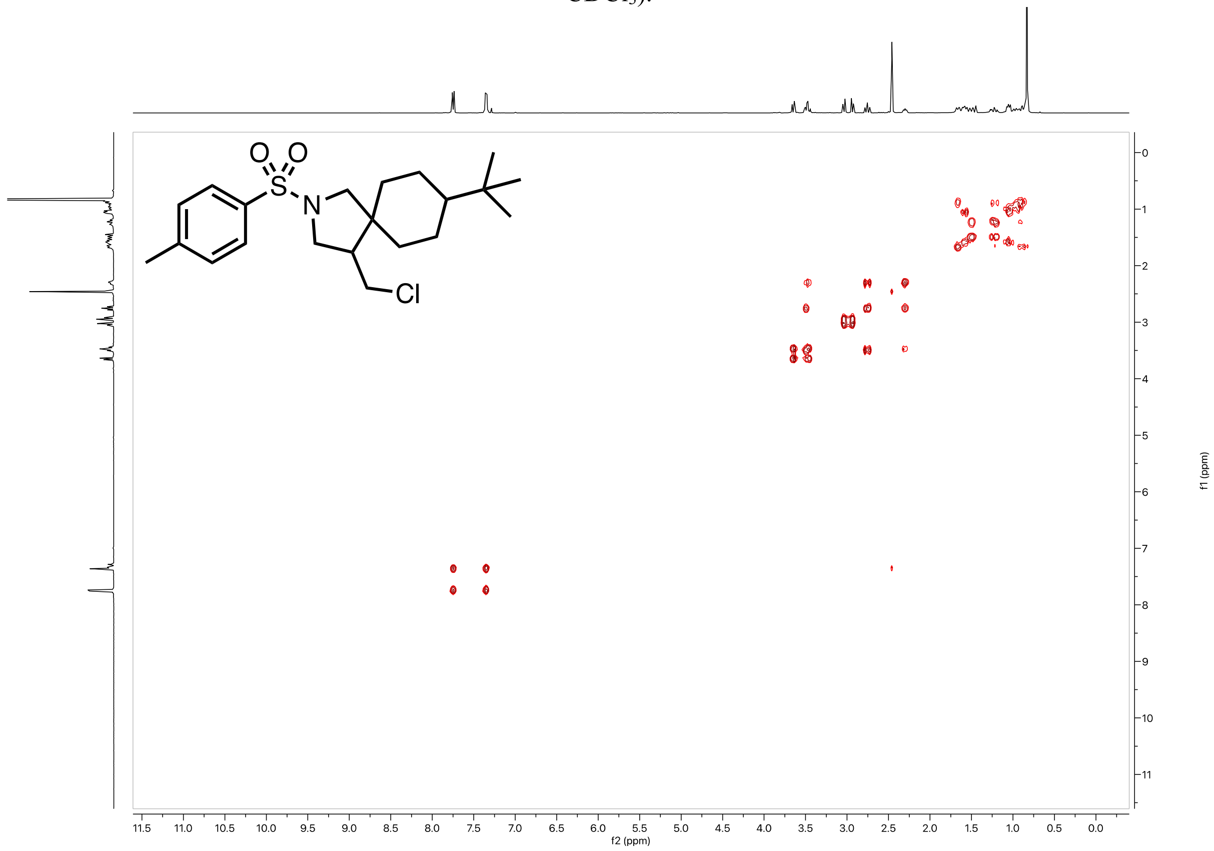

COSY of *8-(tert-butyl)-4-(chloromethyl)-2-tosyl-2-azaspiro[4.5]decane (Major Diastereomer)* ($CDCl_3$).

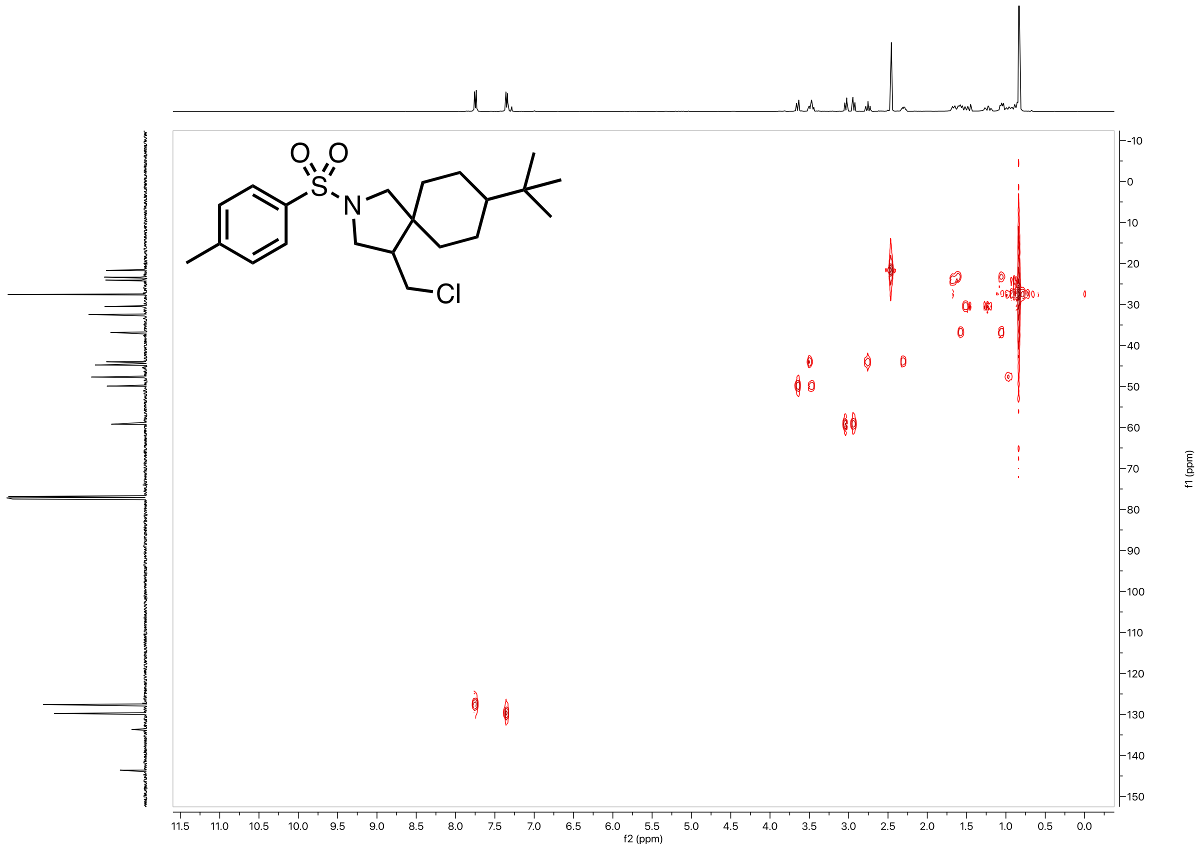

HSQC of *8-(tert-butyl)-4-(chloromethyl)-2-tosyl-2-azaspiro[4.5]decane (Major Diastereomer)* ($CDCl_3$).

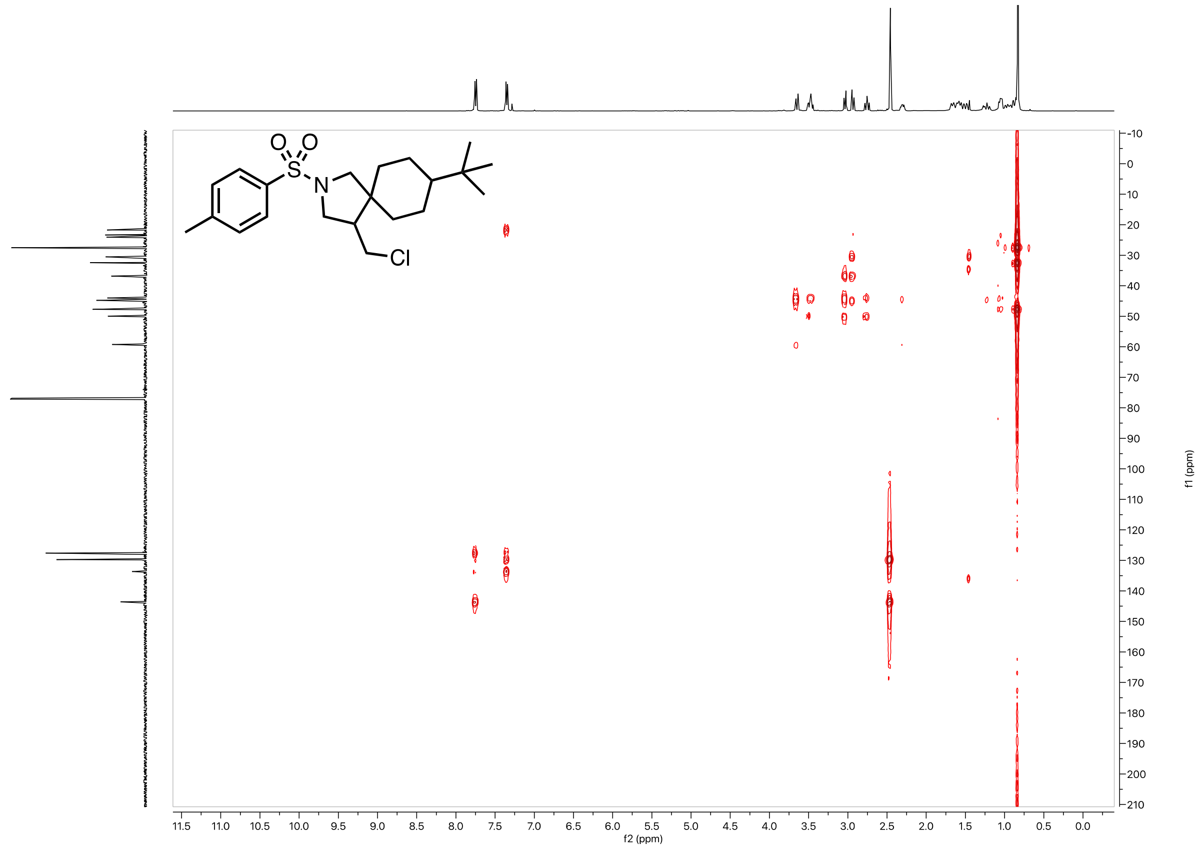

HMBC of *8-(tert-butyl)-4-(chloromethyl)-2-tosyl-2-azaspiro[4.5]decane (Major Diastereomer)* ($CDCl_3$).

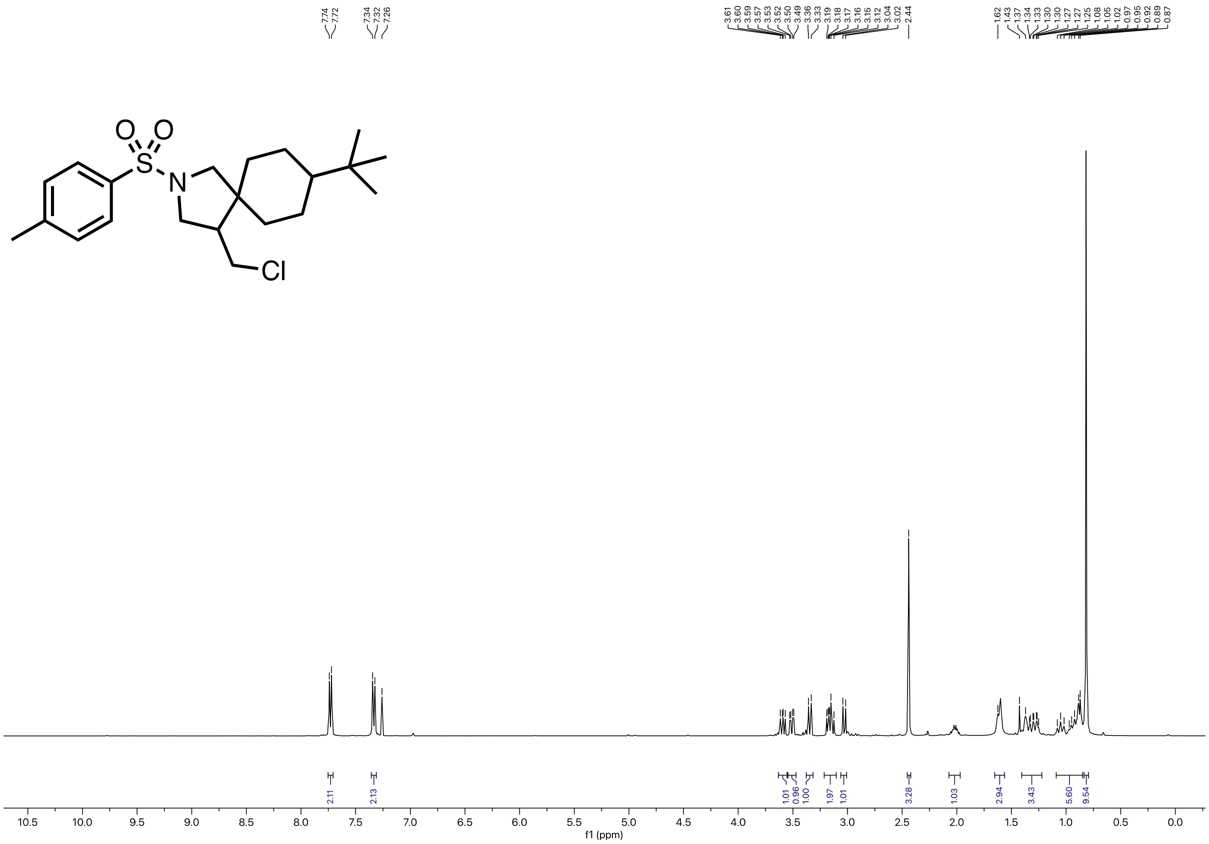

^{1}H NMR of *8-(tert-butyl)-4-(chloromethyl)-2-tosyl-2-azaspiro[4.5]decane (Minor Diastereomer)* (400 MHz, $CDCl_3$).

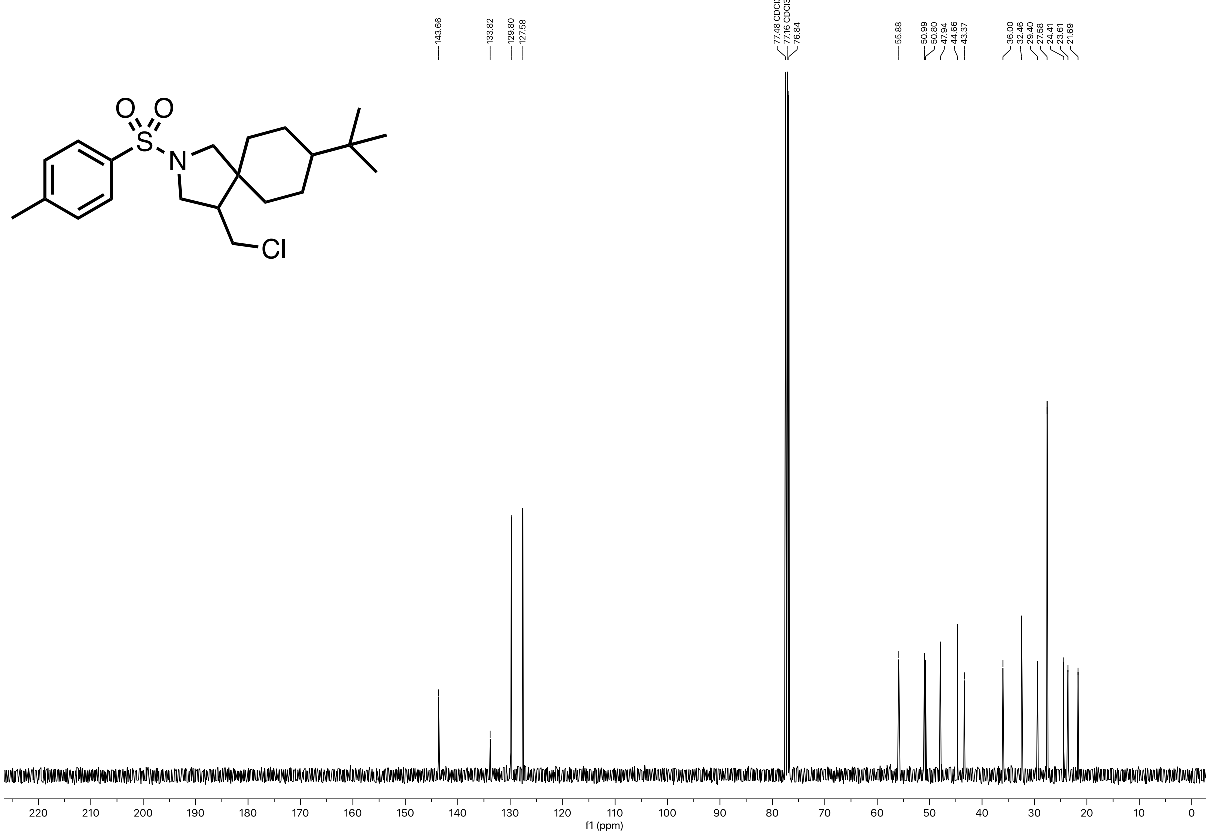

^{13}C{^{1}H} NMR of *8-(tert-butyl)-4-(chloromethyl)-2-tosyl-2-azaspiro[4.5]decane (Minor Diastereomer)* (101 MHz, $CDCl_3$).

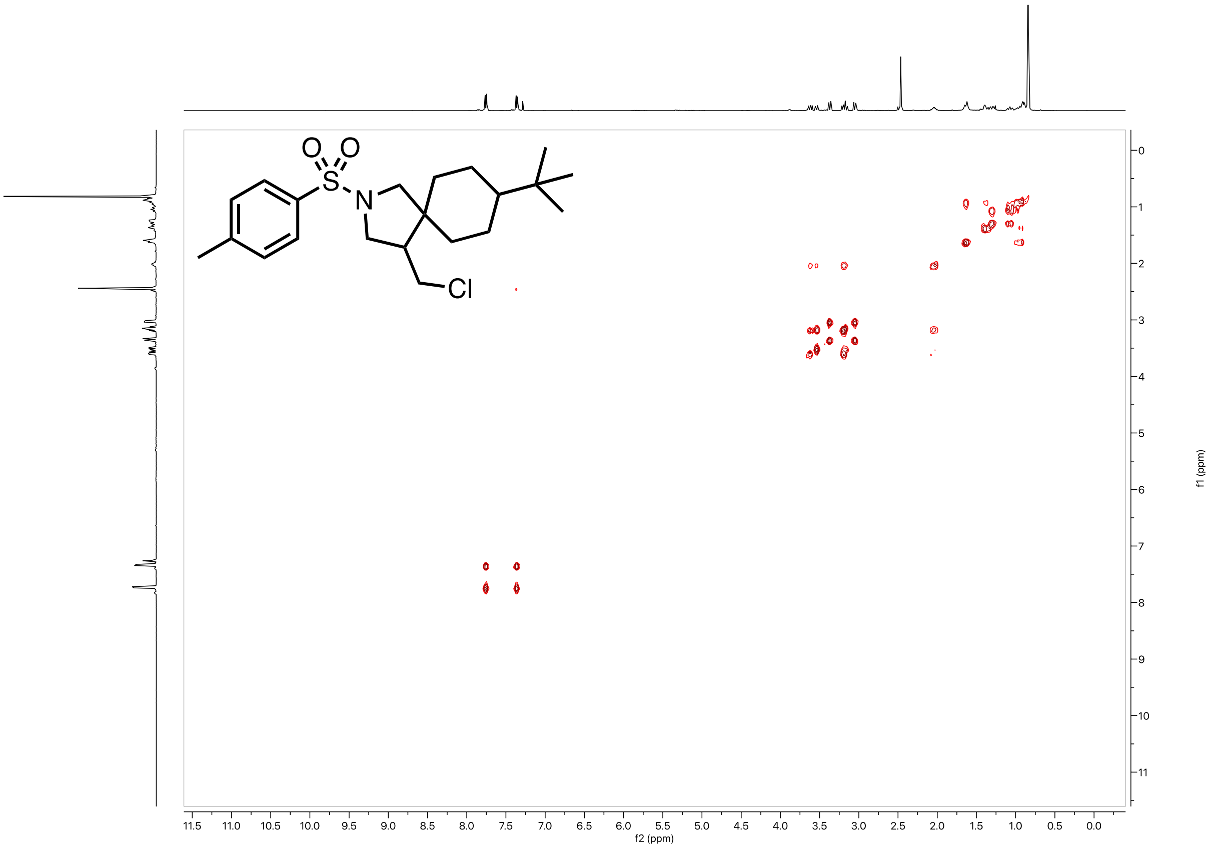

COSY of *8-(tert-butyl)-4-(chloromethyl)-2-tosyl-2-azaspiro[4.5]decane (Minor Diastereomer)* ($CDCl_3$).

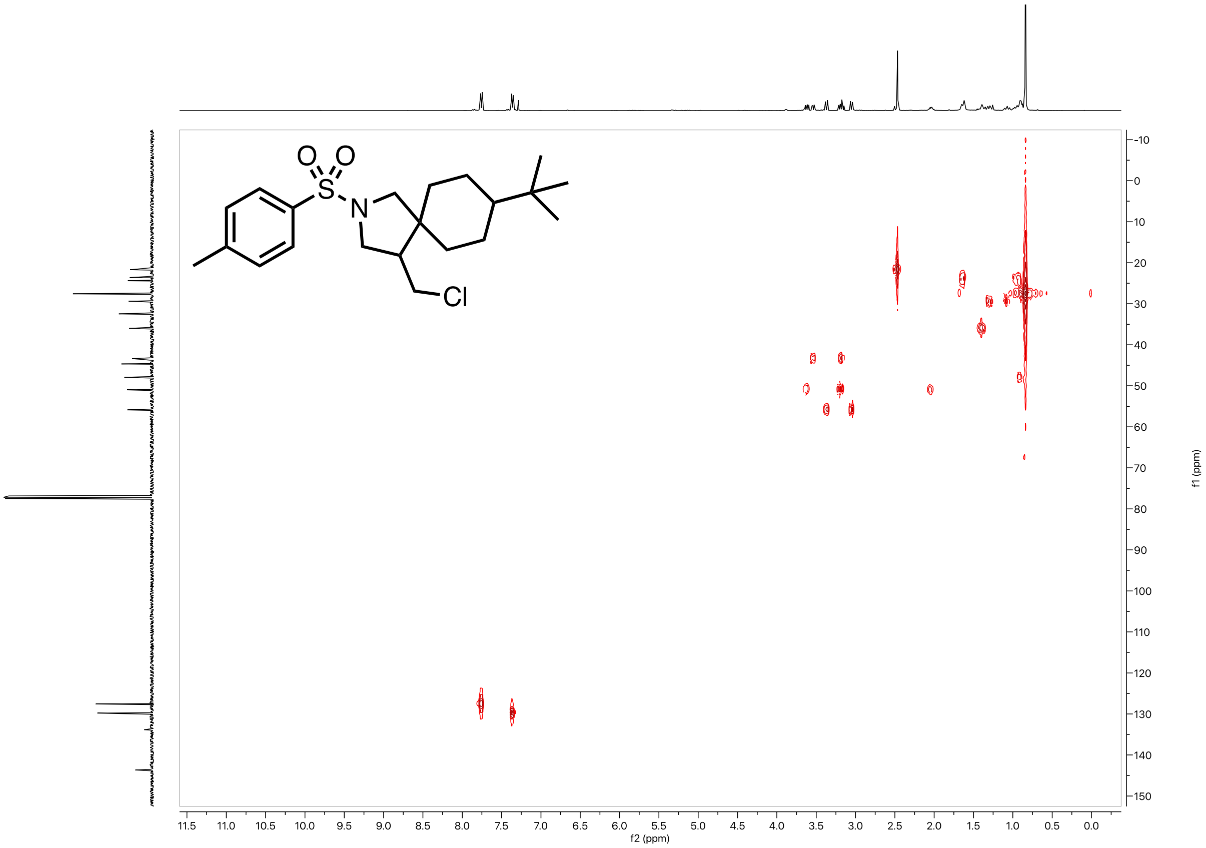

HSQC of *8-(tert-butyl)-4-(chloromethyl)-2-tosyl-2-azaspiro[4.5]decane (Minor Diastereomer)* ($CDCl_3$).

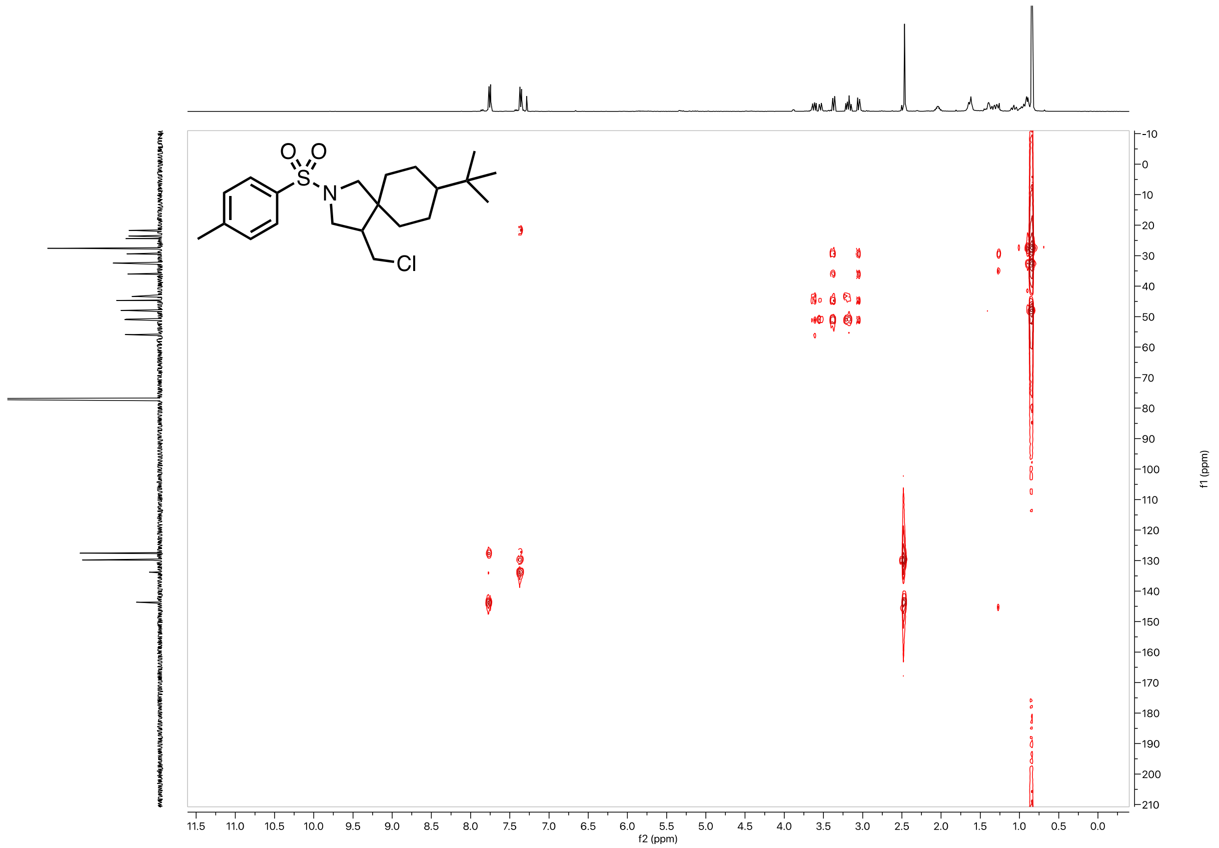

HMBC of *8-(tert-butyl)-4-(chloromethyl)-2-tosyl-2-azaspiro[4.5]decane (Minor Diastereomer)* ($CDCl_3$).

4-(chloromethyl)-8-phenyl-2-tosyl-2-azaspiro[4.5]decane (3n)

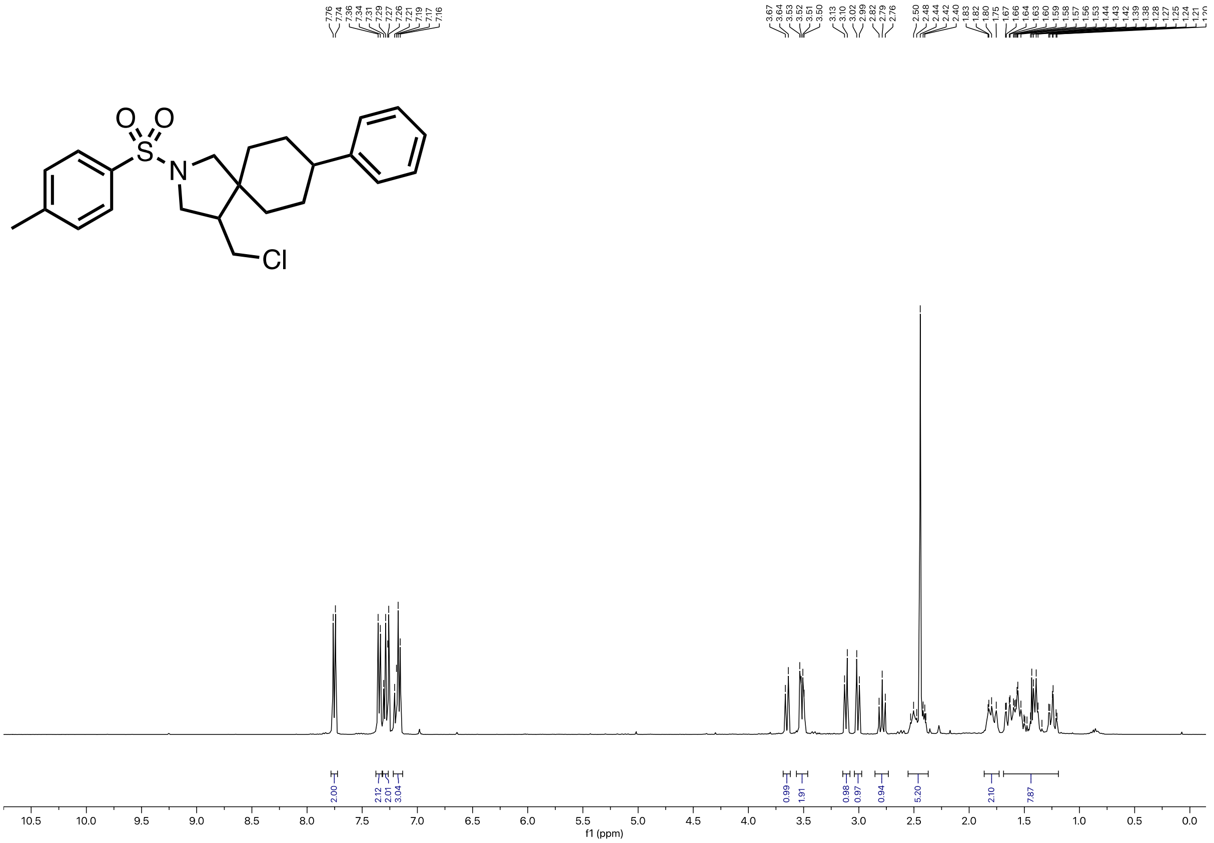

^{1}H NMR of *4-(chloromethyl)-8-phenyl-2-tosyl-2-azaspiro[4.5]decane (Major Diastereomer)* (400 MHz, $CDCl_3$).

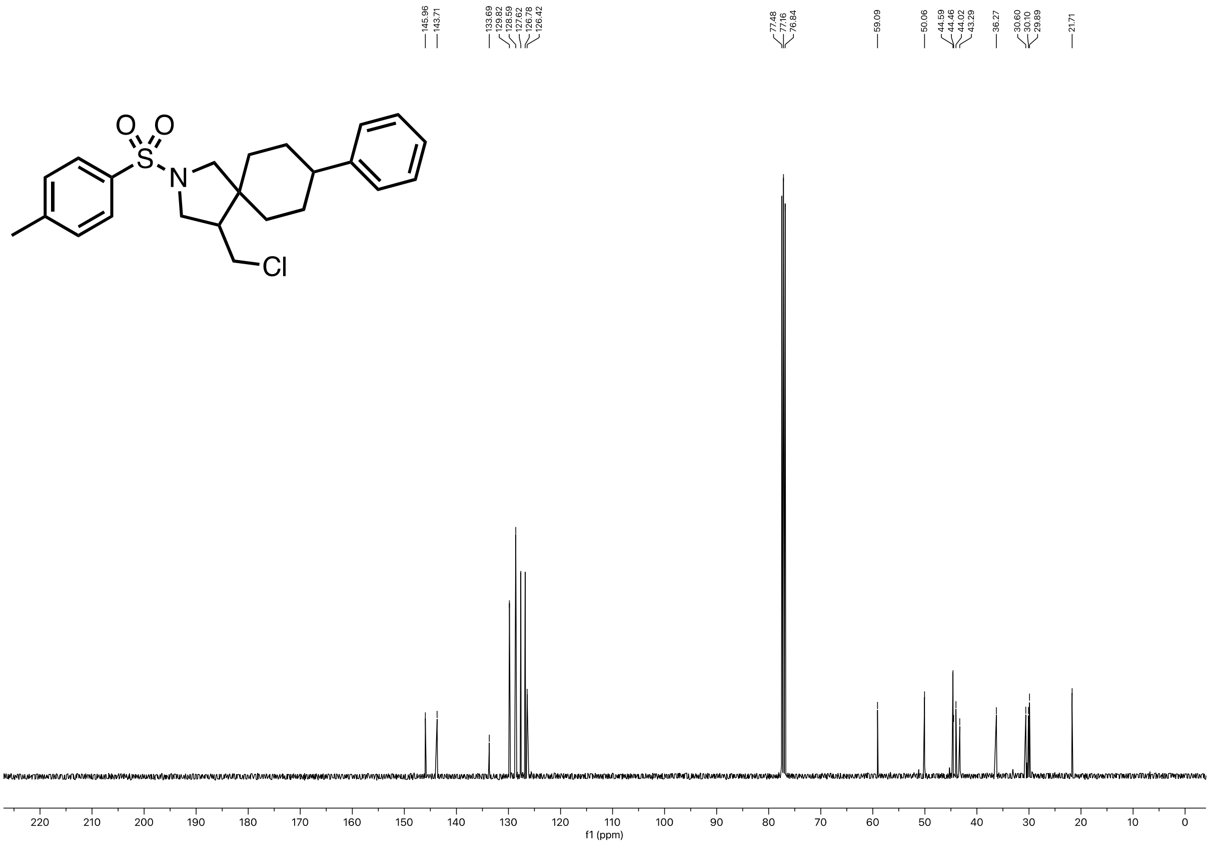

$^{13}C\{^{1}H\}$ NMR of *4-(chloromethyl)-8-phenyl-2-tosyl-2-azaspiro[4.5]decane (Major Diastereomer)* (101 MHz, $CDCl_3$).

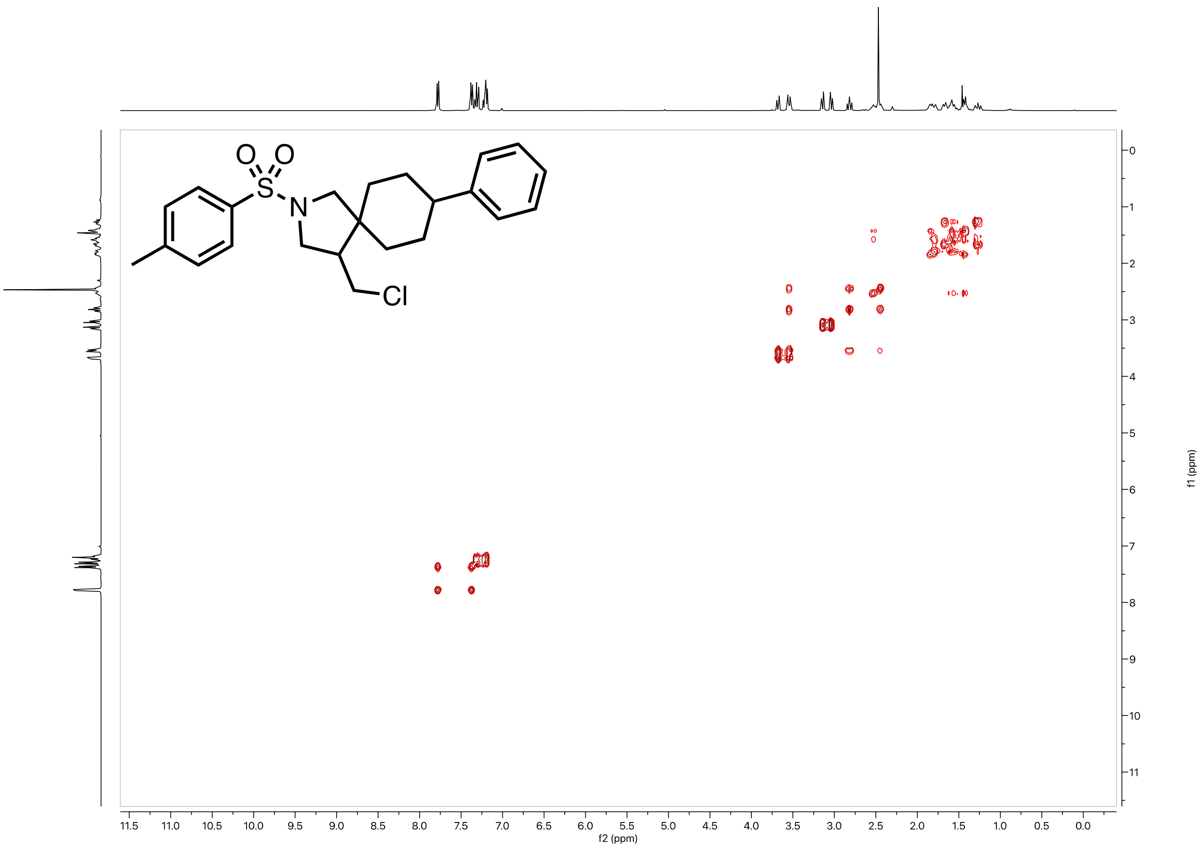

COSY of *4-(chloromethyl)-8-phenyl-2-tosyl-2-azaspiro[4.5]decane (Major Diastereomer)* ($CDCl_3$).

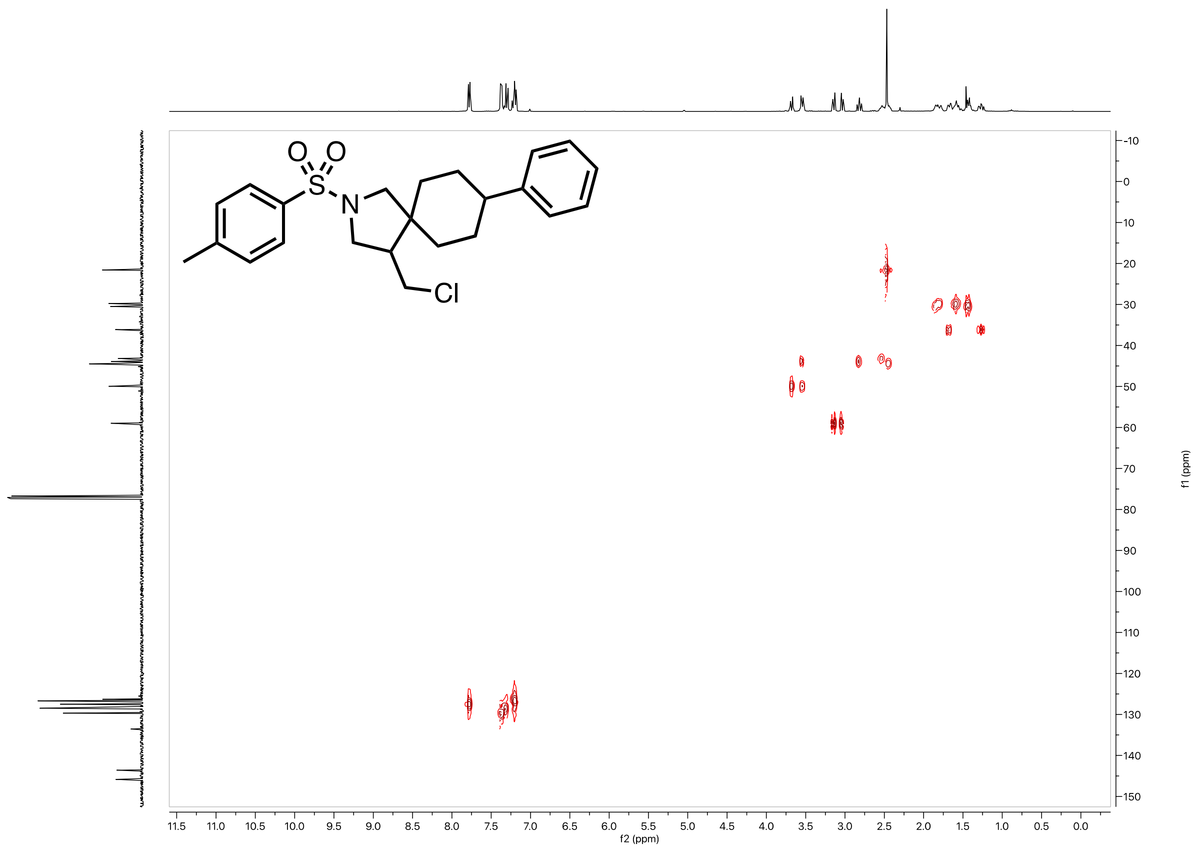

HSQC of *4-(chloromethyl)-8-phenyl-2-tosyl-2-azaspiro[4.5]decane (Major Diastereomer)* ($CDCl_3$).

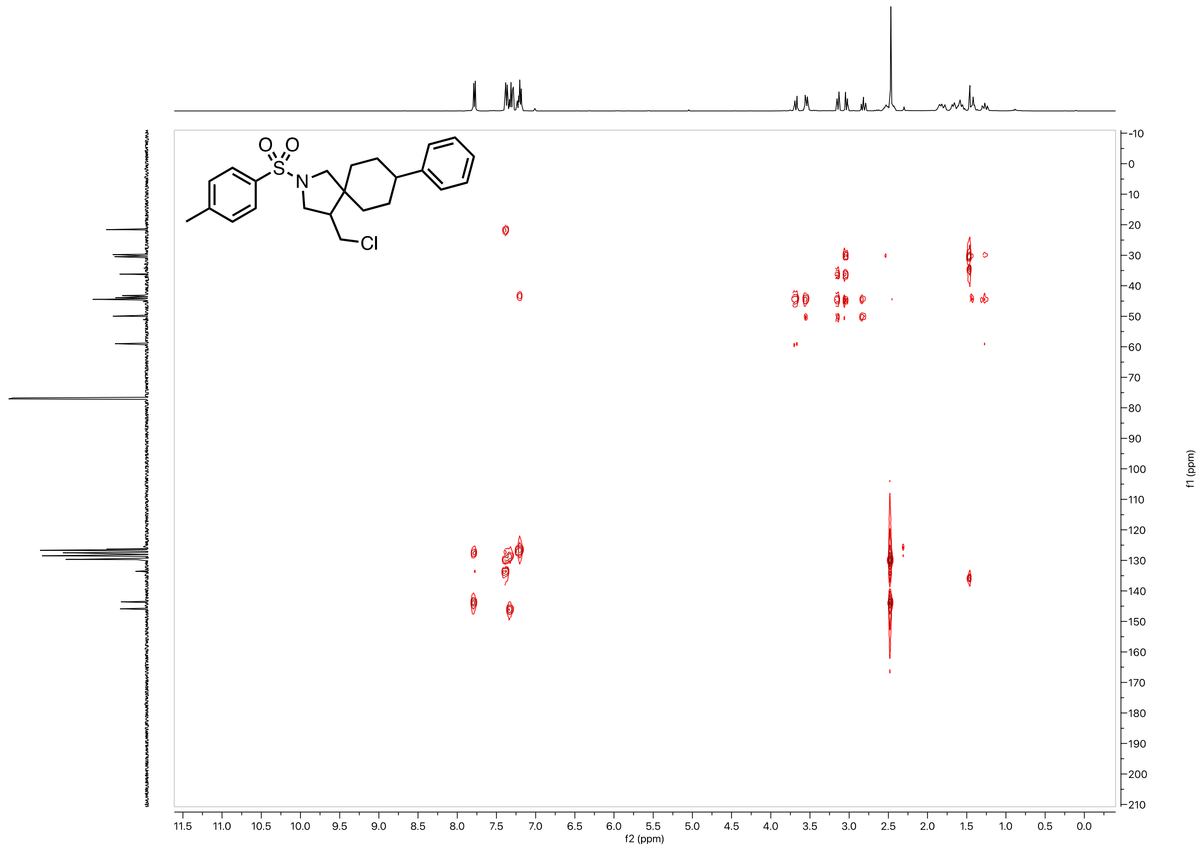

HMBC of *4-(chloromethyl)-8-phenyl-2-tosyl-2-azaspiro[4.5]decane (Major Diastereomer)* ($CDCl_3$).

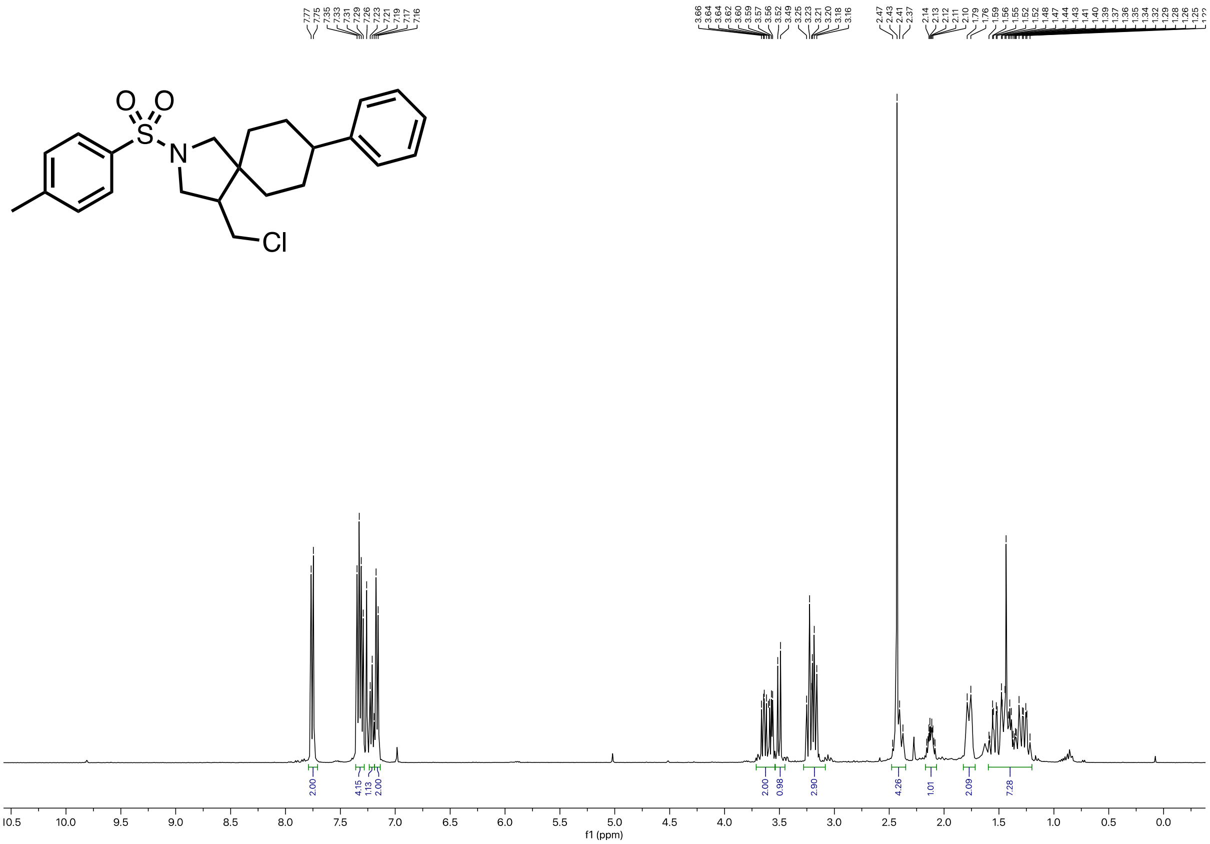

^{1}H NMR of *4-(chloromethyl)-8-phenyl-2-tosyl-2-azaspiro[4.5]decane (Minor Diastereomer)* (400 MHz, $CDCl_3$).

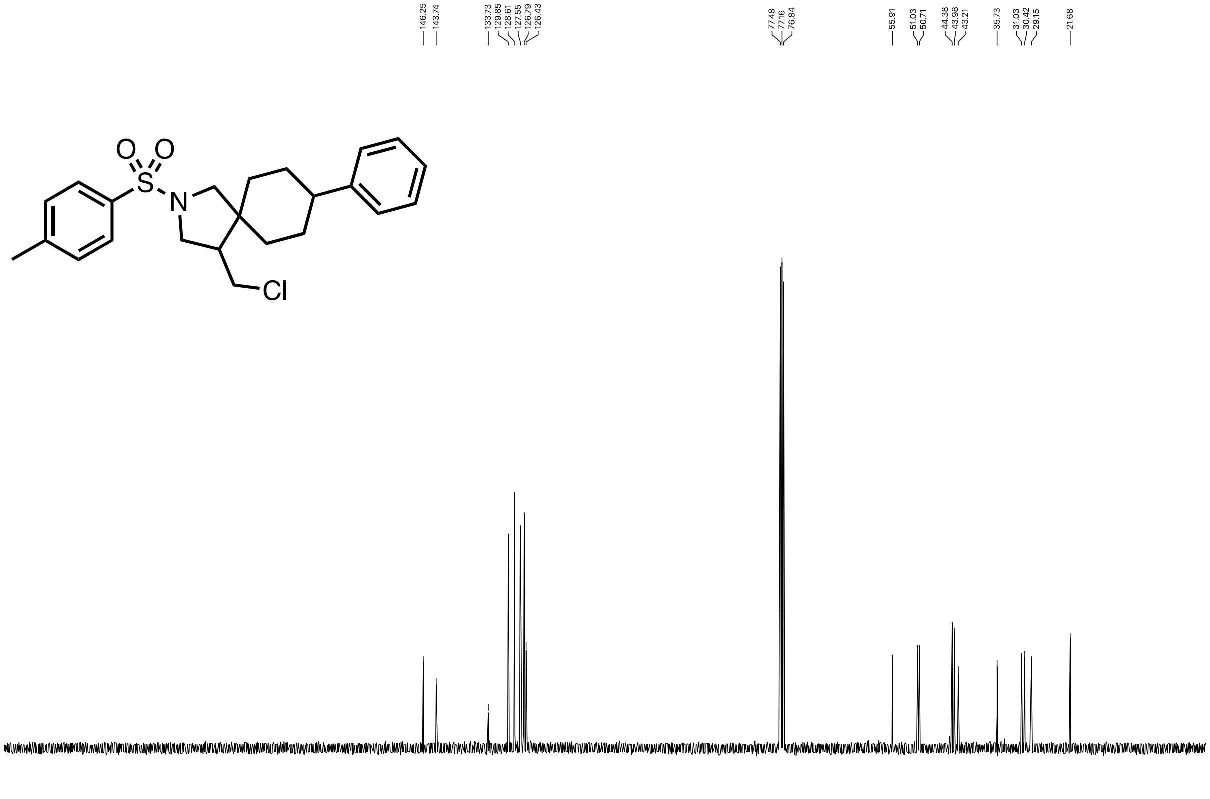

$^{13}C\{^{1}H\}$ NMR of *4-(chloromethyl)-8-phenyl-2-tosyl-2-azaspiro[4.5]decane (Minor Diastereomer)* (101 MHz, $CDCl_3$).

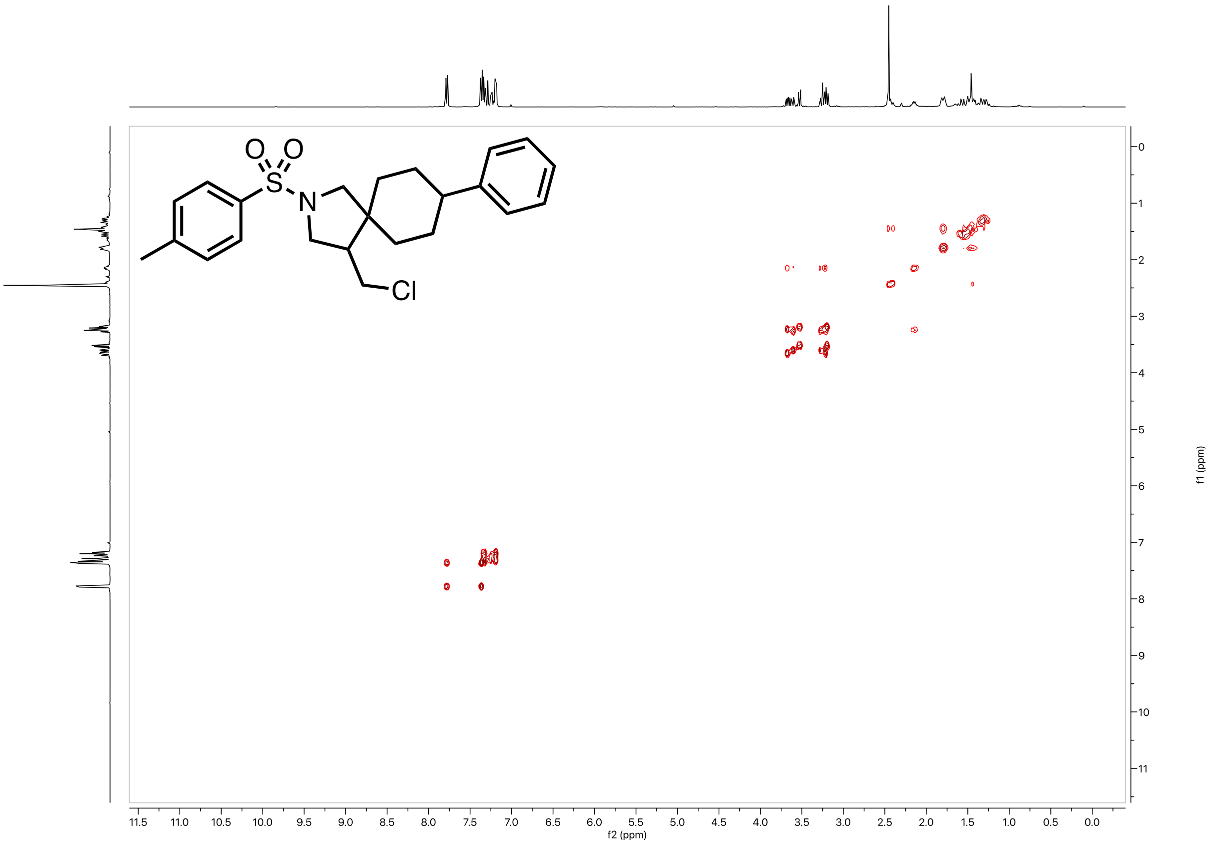

COSY of *4-(chloromethyl)-8-phenyl-2-tosyl-2-azaspiro[4.5]decane (Minor Diastereomer)* ($CDCl_3$).

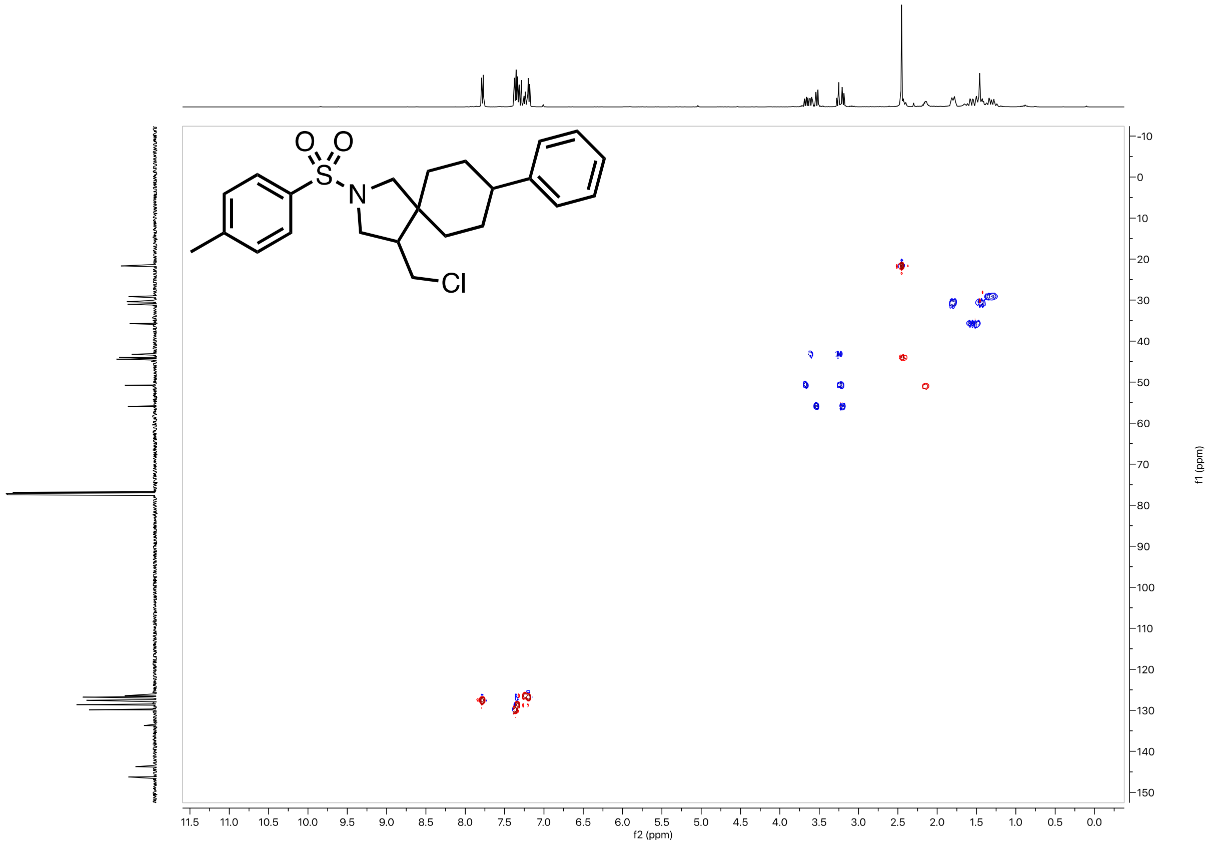

eHSQC of *4-(chloromethyl)-8-phenyl-2-tosyl-2-azaspiro[4.5]decane (Minor Diastereomer)* ($CDCl_3$).

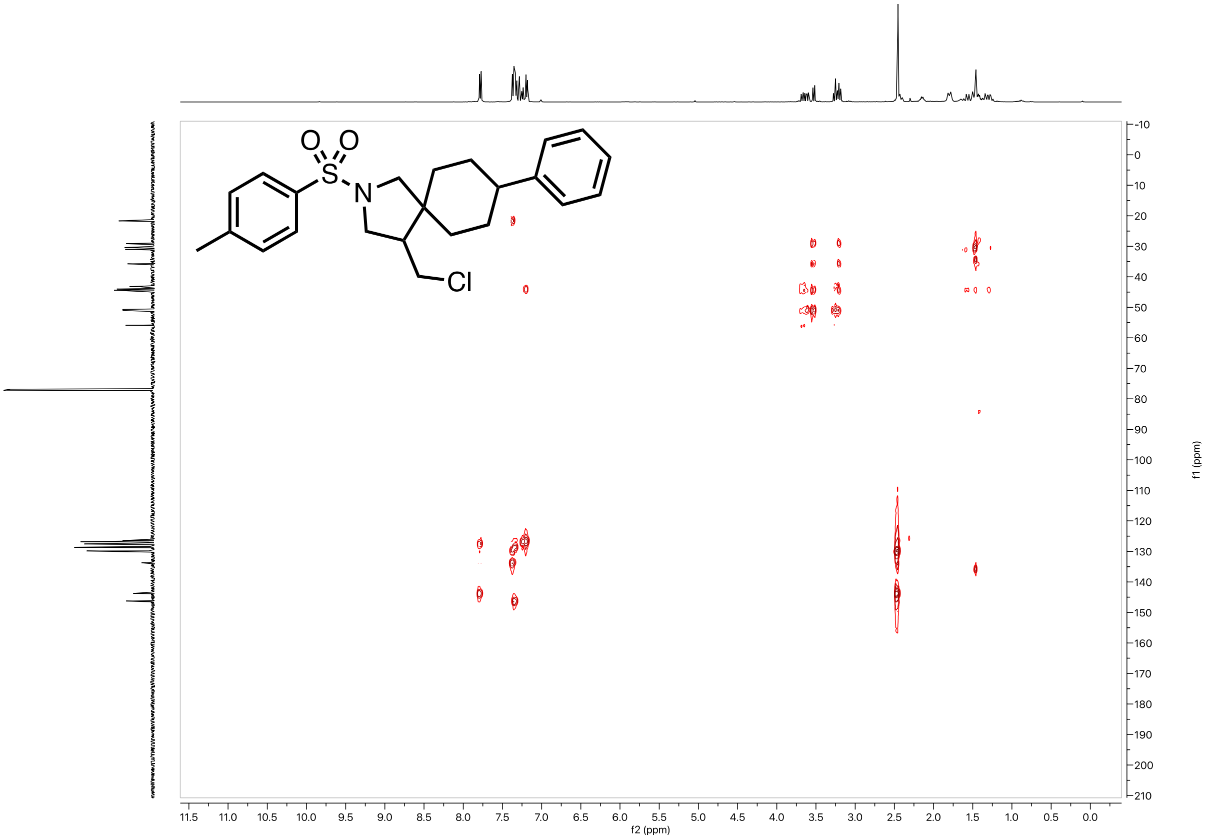

HMBC of *4-(chloromethyl)-8-phenyl-2-tosyl-2-azaspiro[4.5]decane (Minor Diastereomer)* ($CDCl_3$).

4'-(chloromethyl)-1'-tosyl-1,3-dihydrospiro[indene-2,3'-pyrrolidine] (3o)

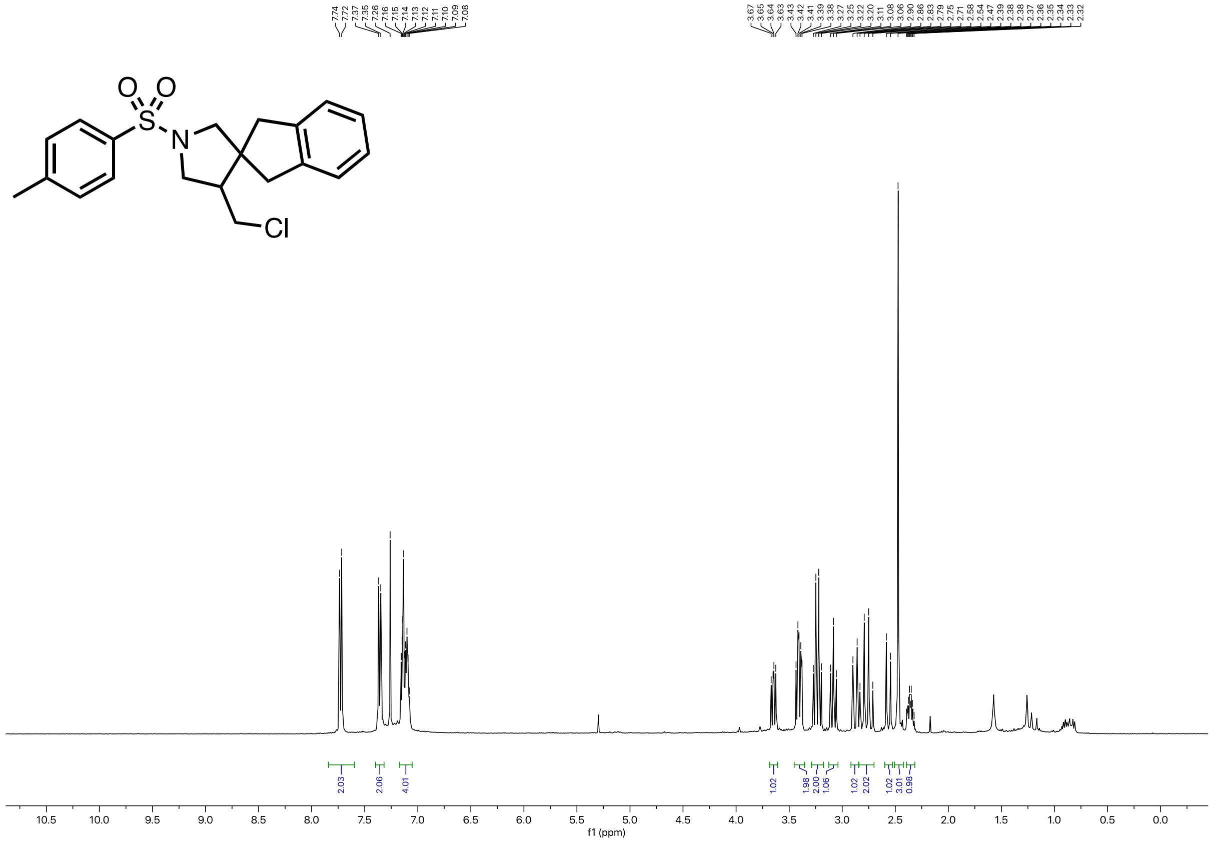

^{1}H NMR of *4'-(chloromethyl)-1'-tosyl-1,3-dihydrospiro[indene-2,3'-pyrrolidine]* (400 MHz, $CDCl_3$).

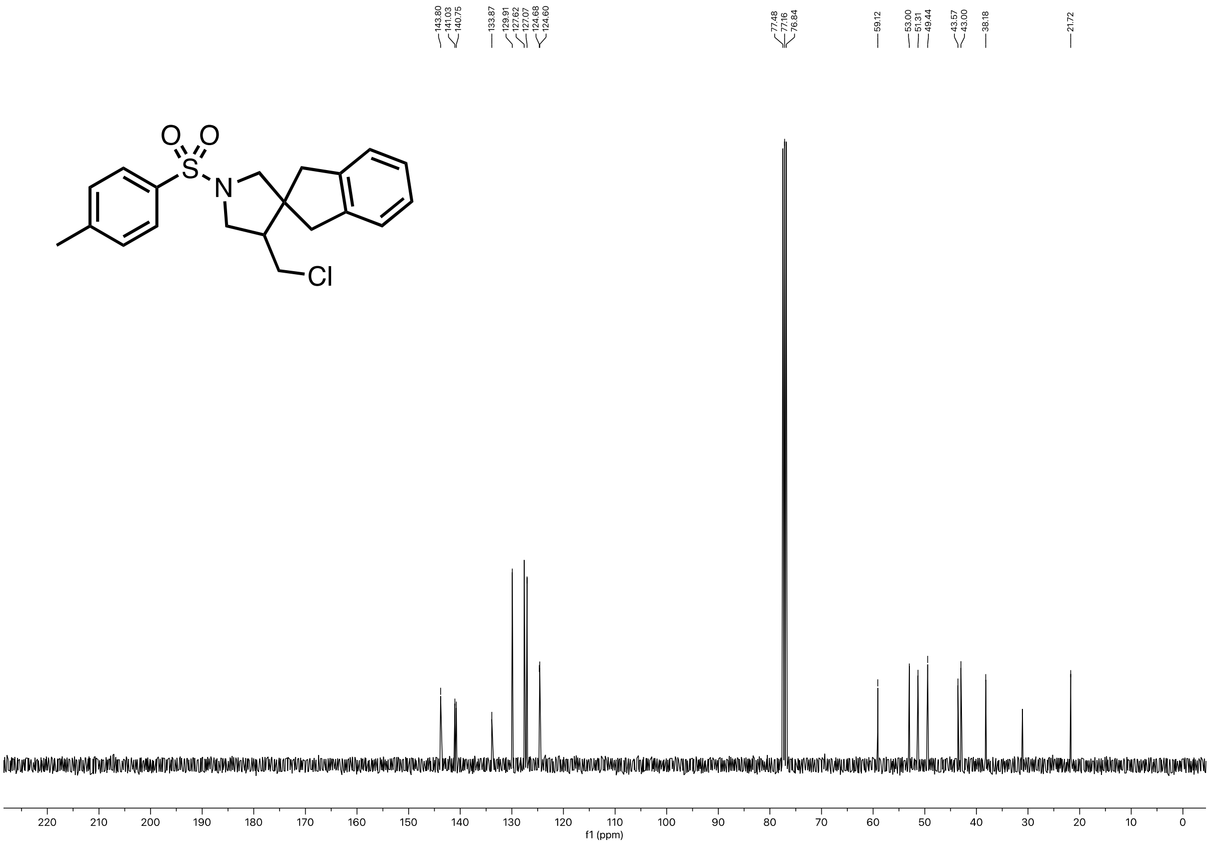

$^{13}C\{^1H\}$ NMR of *4'-(chloromethyl)-1'-tosyl-1,3-dihydrospiro[indene-2,3'-pyrrolidine]* (101 MHz, $CDCl_3$).

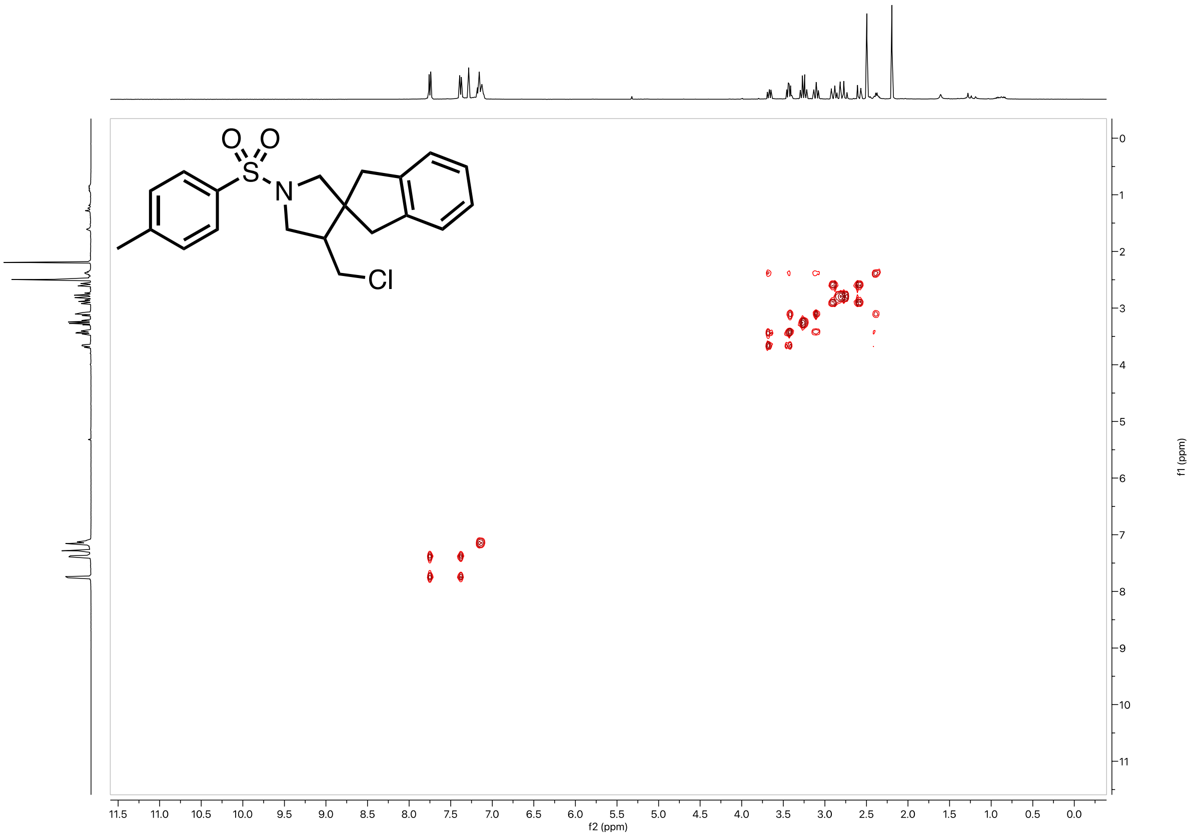

COSY of *4'-(chloromethyl)-1'-tosyl-1,3-dihydrospiro[indene-2,3'-pyrrolidine]* ($CDCl_3$).

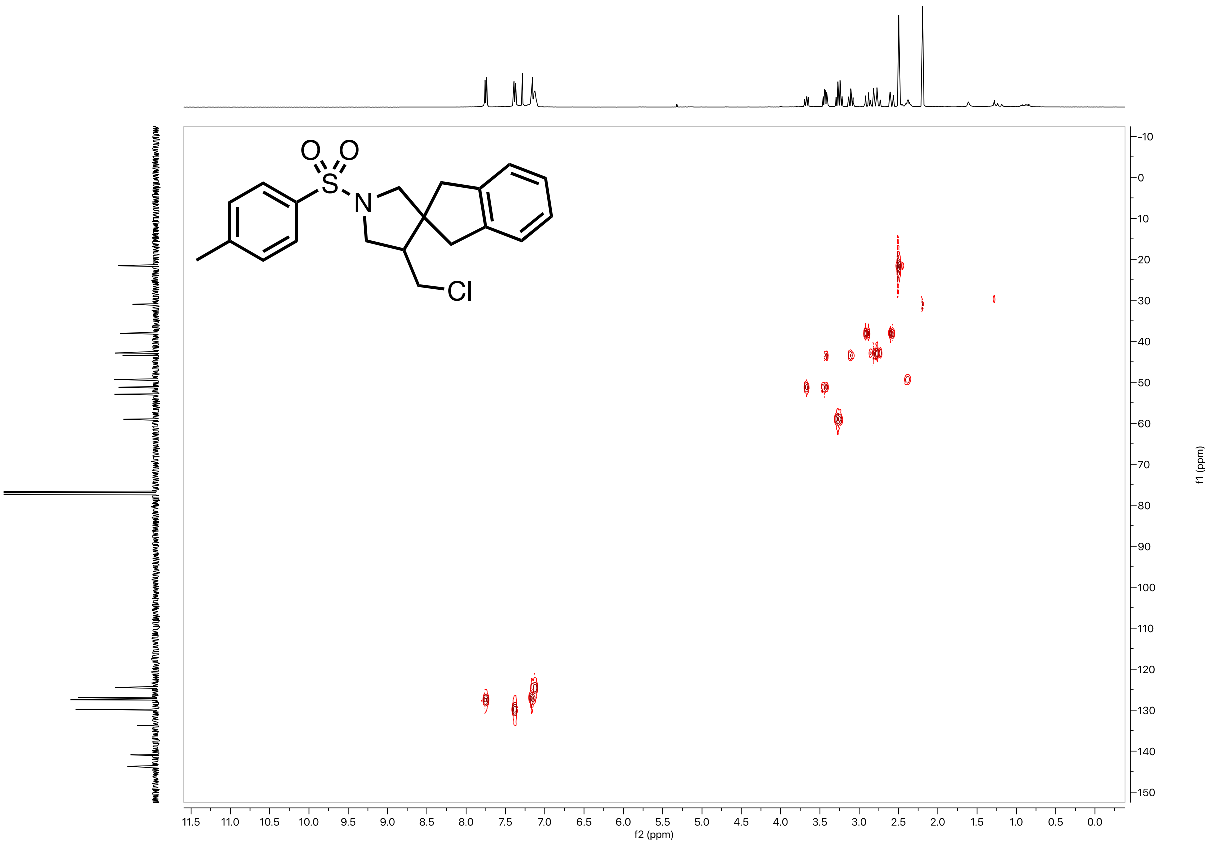

HSQC of *4'-(chloromethyl)-1'-tosyl-1,3-dihydrospiro[indene-2,3'-pyrrolidine]* ($CDCl_3$).

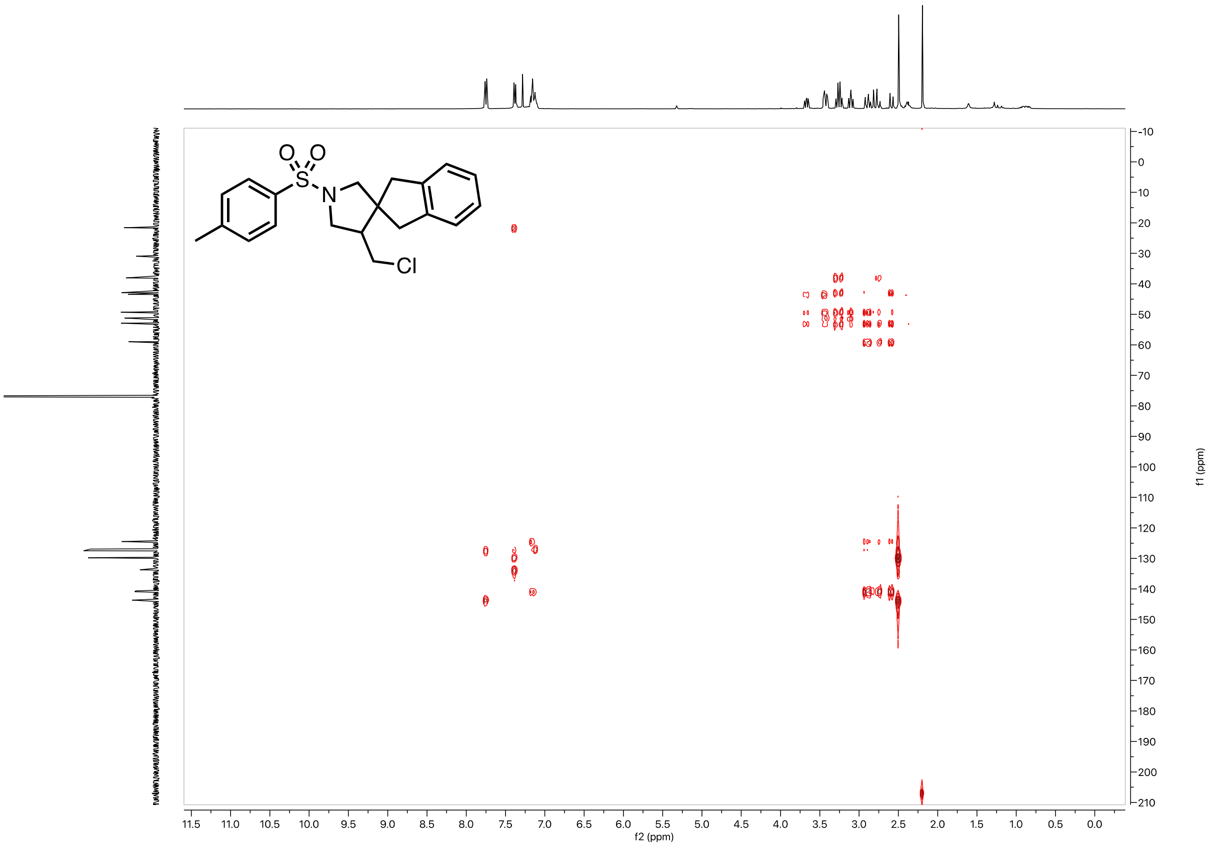

HMBC of *4'-(chloromethyl)-1'-tosyl-1,3-dihydrospiro[indene-2,3'-pyrrolidine]* ($CDCl_3$).

(1R,3S,5r,7r)-4'-(chloromethyl)-1'-tosylspiro[adamantane-2,3'-pyrrolidine] (3p)

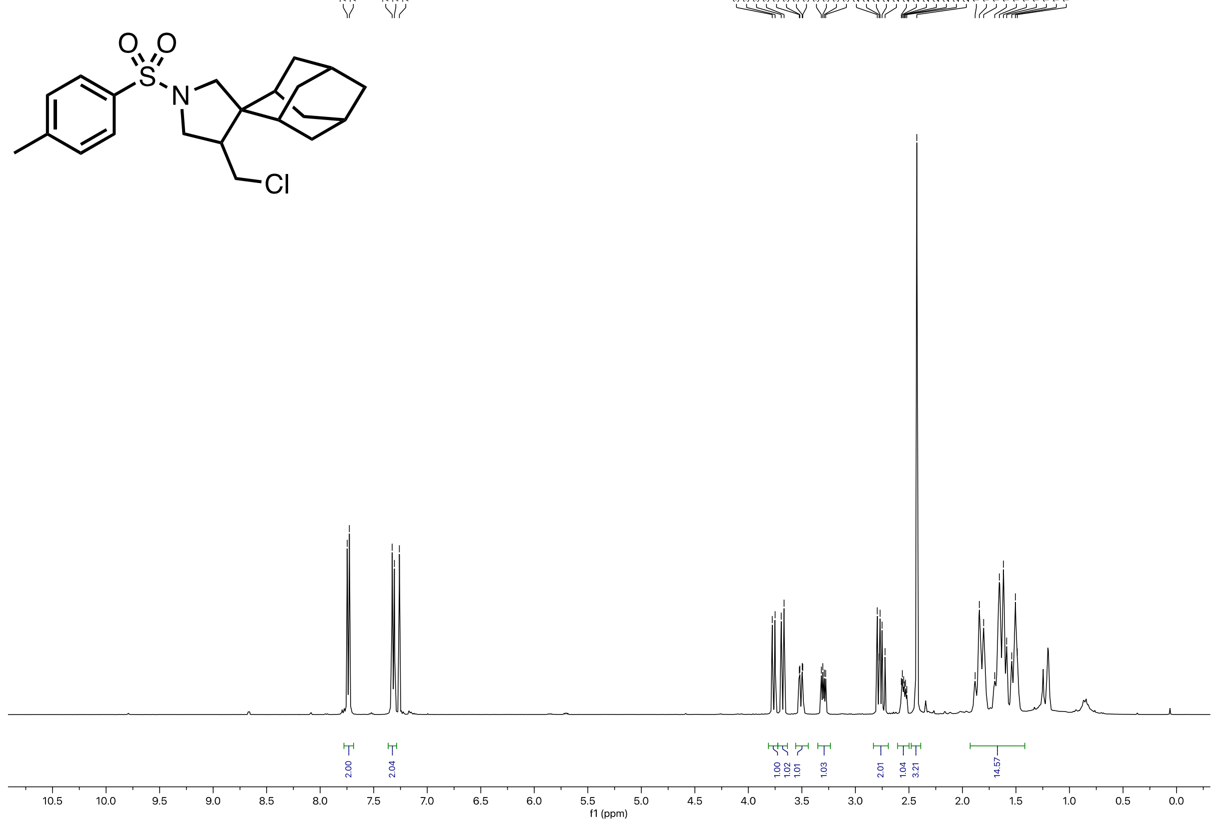

^{1}H NMR of *(1R,3S,5r,7r)-4'-(chloromethyl)-1'-tosylspiro[adamantane-2,3'-pyrrolidine]* (400 MHz, $CDCl_3$).

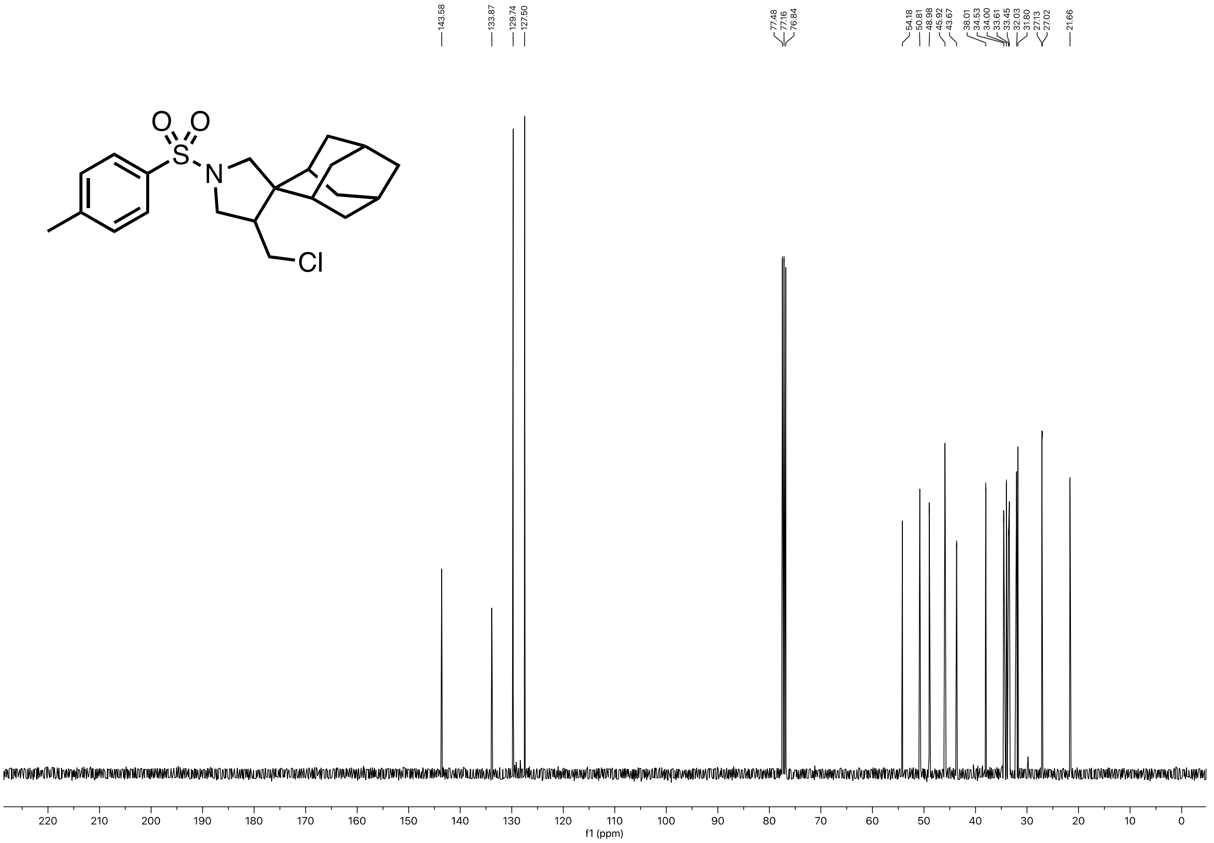

^{13}C{^{1}H} NMR of *(1R,3S,5r,7r)-4'-(chloromethyl)-1'-tosylspiro[adamantane-2,3'-pyrrolidine]* (101 MHz, $CDCl_3$).

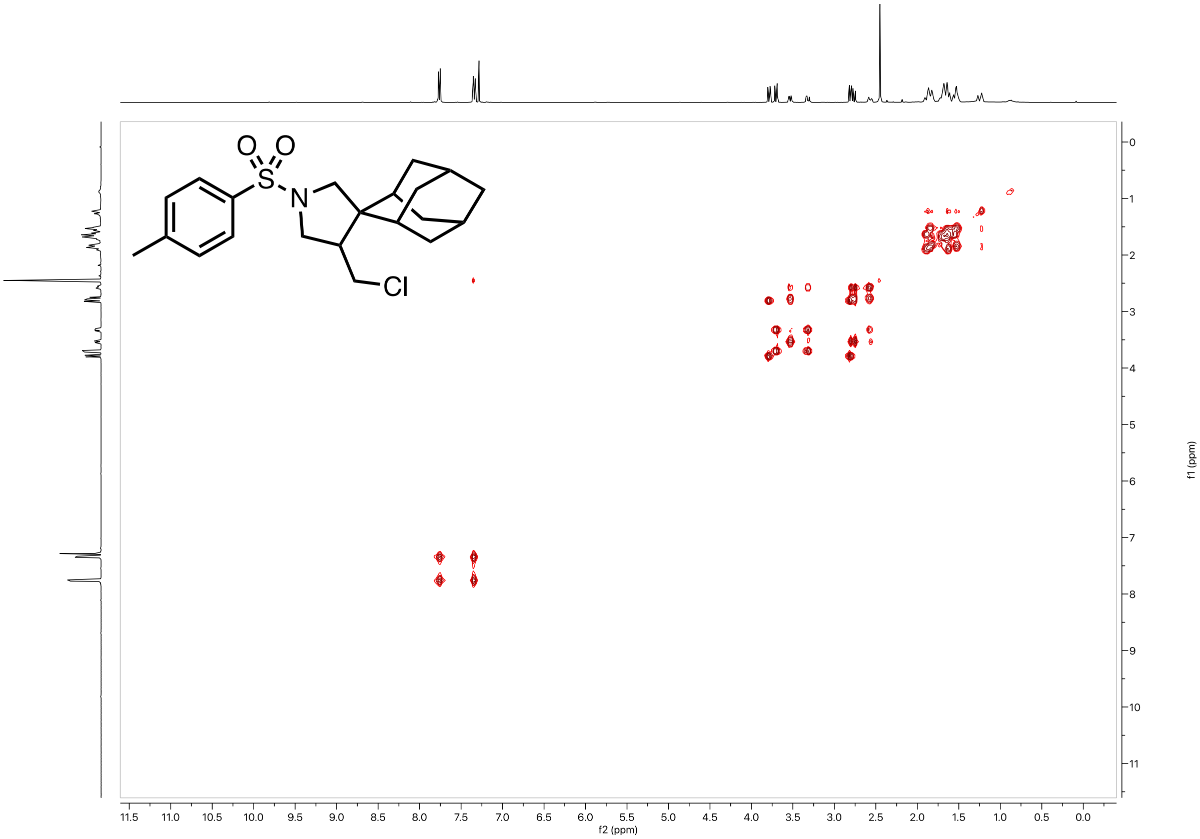

COSY of *(1R,3S,5r,7r)-4'-(chloromethyl)-1'-tosylspiro[adamantane-2,3'-pyrrolidine]* ($CDCl_3$).

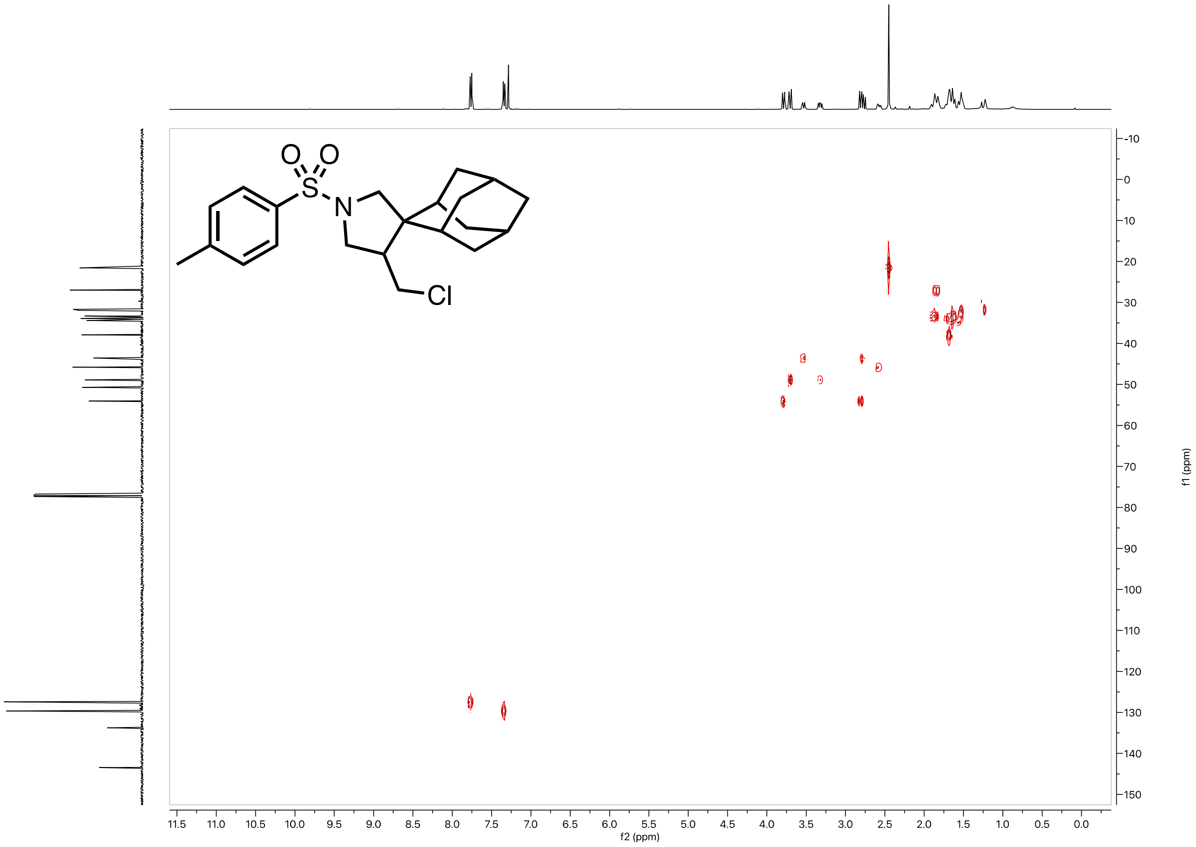

HSQC of *(1R,3S,5r,7r)-4'-(chloromethyl)-1'-tosylspiro[adamantane-2,3'-pyrrolidine]* ($CDCl_3$).

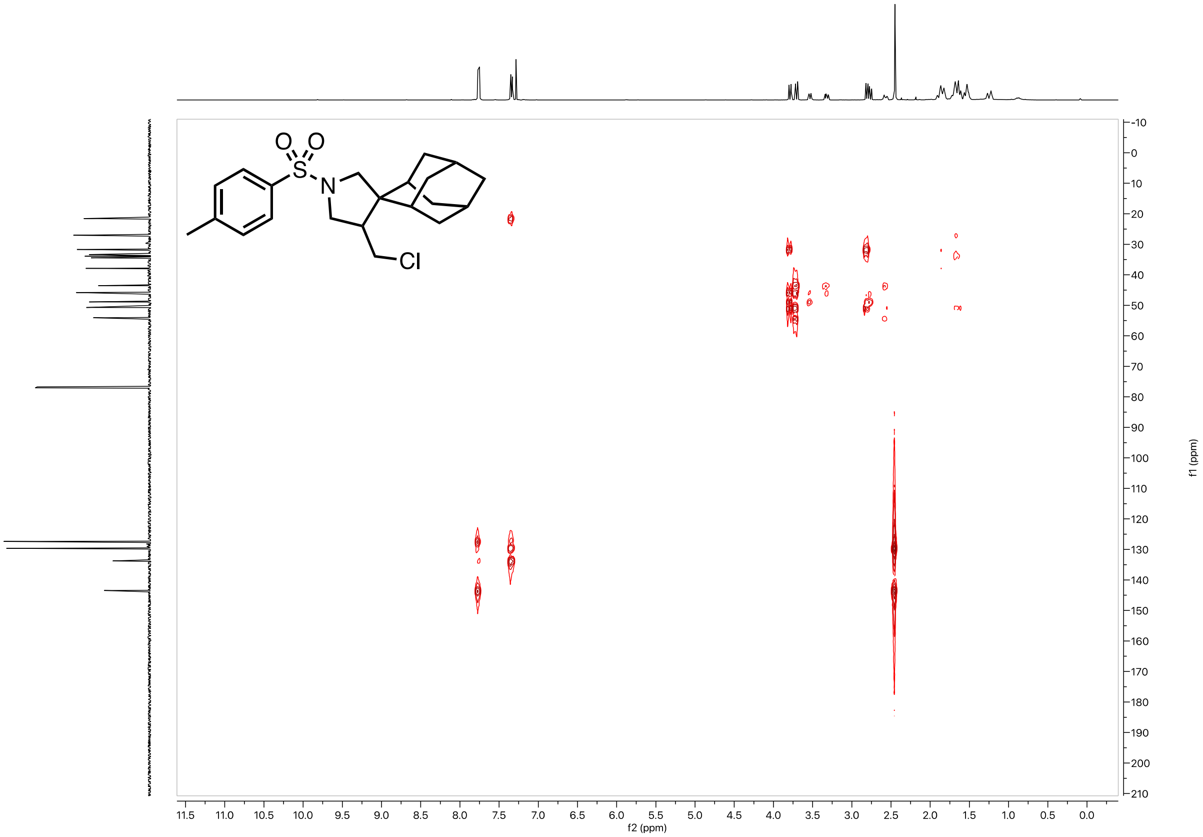

HMBC of *(1R,3S,5r,7r)-4'-(chloromethyl)-1'-tosylspiro[adamantane-2,3'-pyrrolidine]* ($CDCl_3$).

4-(chloromethyl)-8,8-difluoro-2-tosyl-2-azaspiro[4.5]decane (3q)

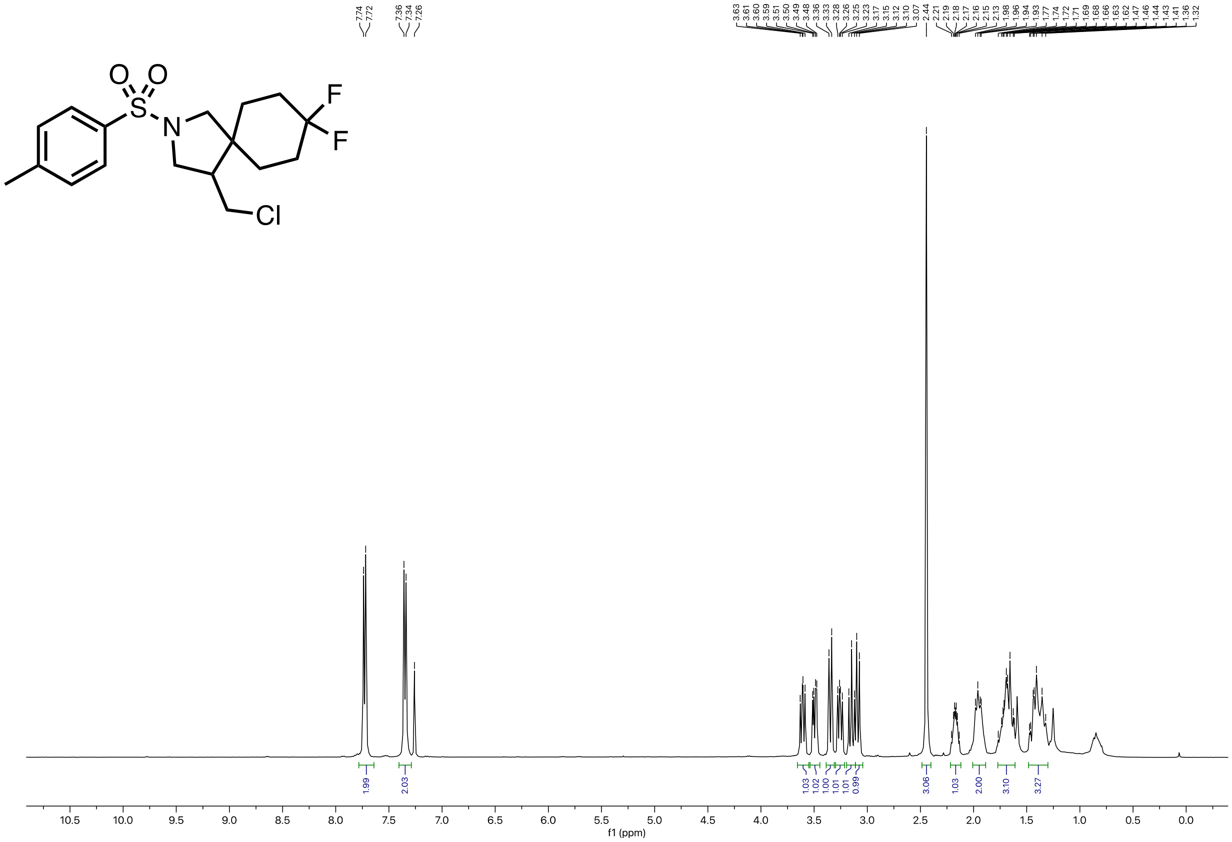

^{1}H NMR of *4-(chloromethyl)-8,8-difluoro-2-tosyl-2-azaspiro[4.5]decane* (400 MHz, $CDCl_3$).

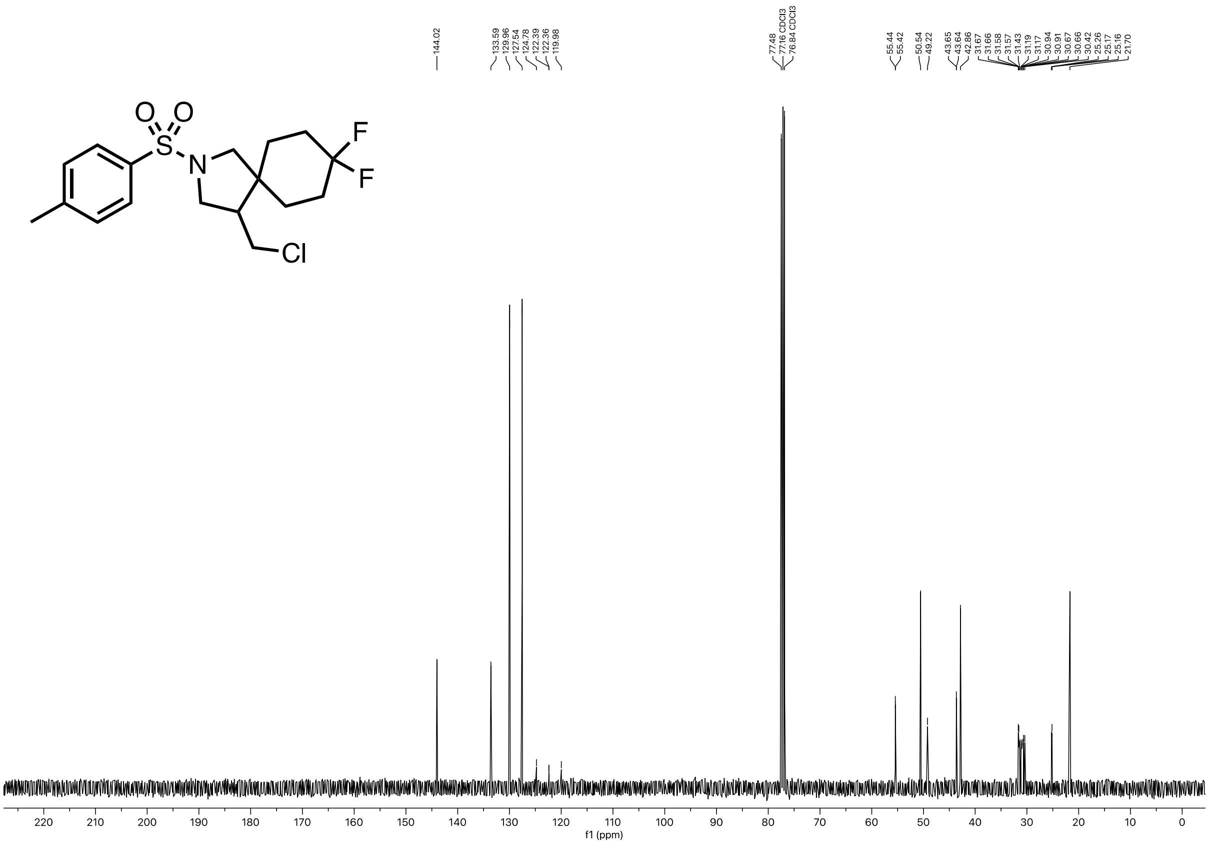

$^{13}C\{^{1}H\}$ NMR of *4-(chloromethyl)-8,8-difluoro-2-tosyl-2-azaspiro[4.5]decane* (101 MHz, $CDCl_3$).

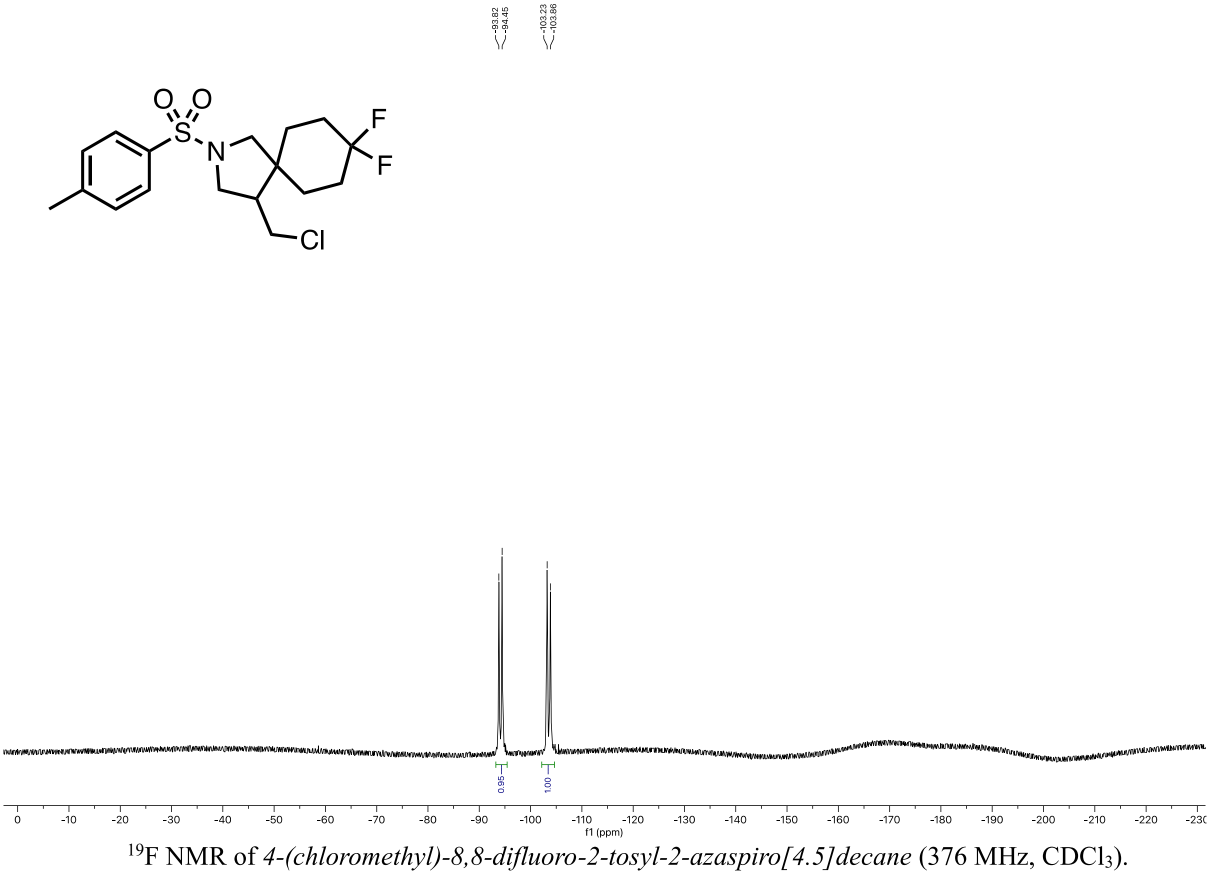

^{19}F NMR of *4-(chloromethyl)-8,8-difluoro-2-tosyl-2-azaspiro[4.5]decane* (376 MHz, $CDCl_3$).

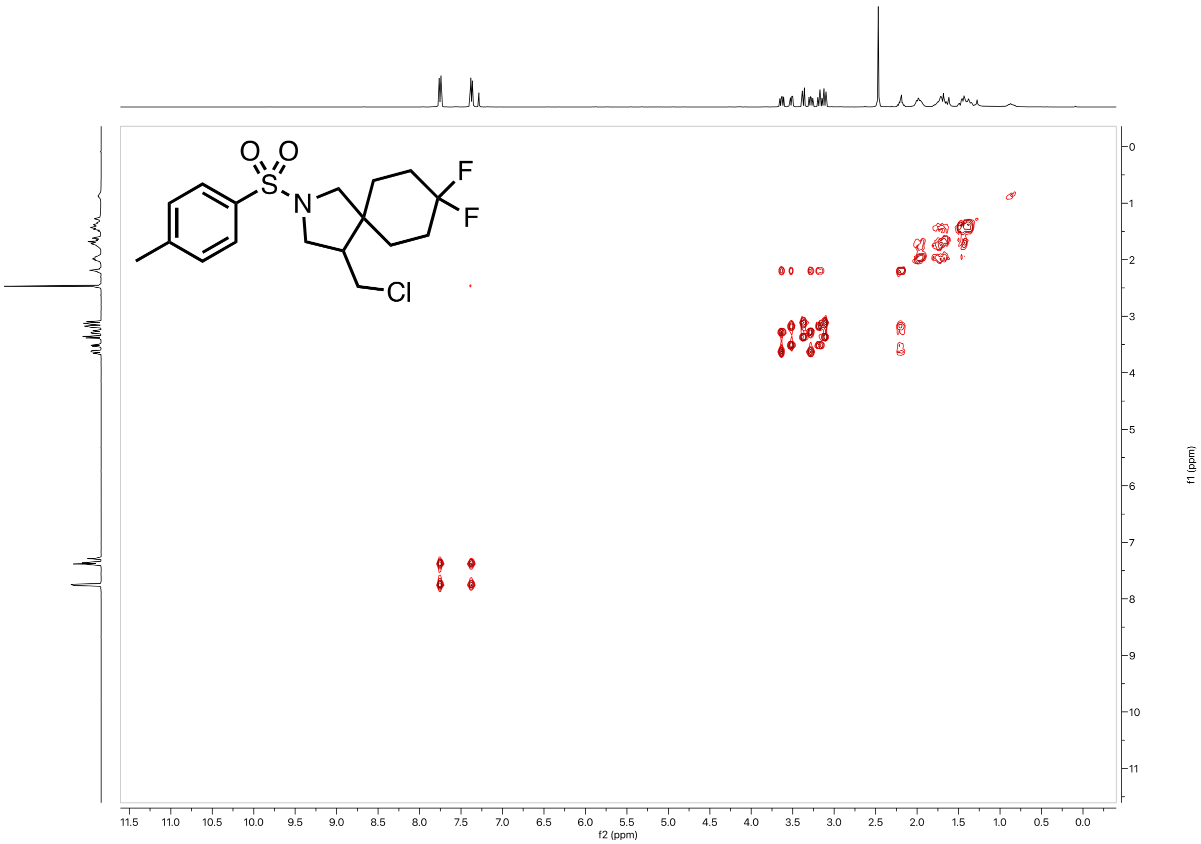

COSY of *4-(chloromethyl)-8,8-difluoro-2-tosyl-2-azaspiro[4.5]decane* ($CDCl_3$).

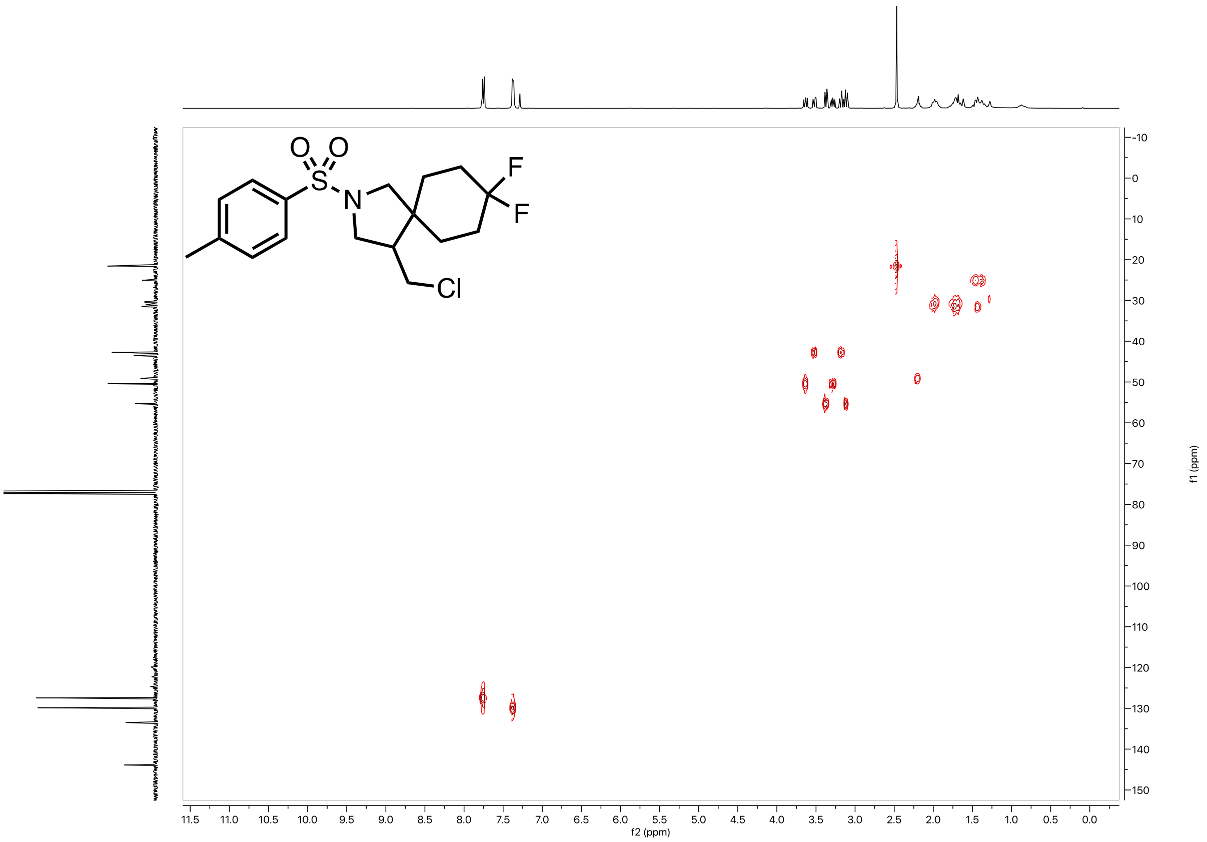

HSQC of *4-(chloromethyl)-8,8-difluoro-2-tosyl-2-azaspiro[4.5]decane* ($CDCl_3$).

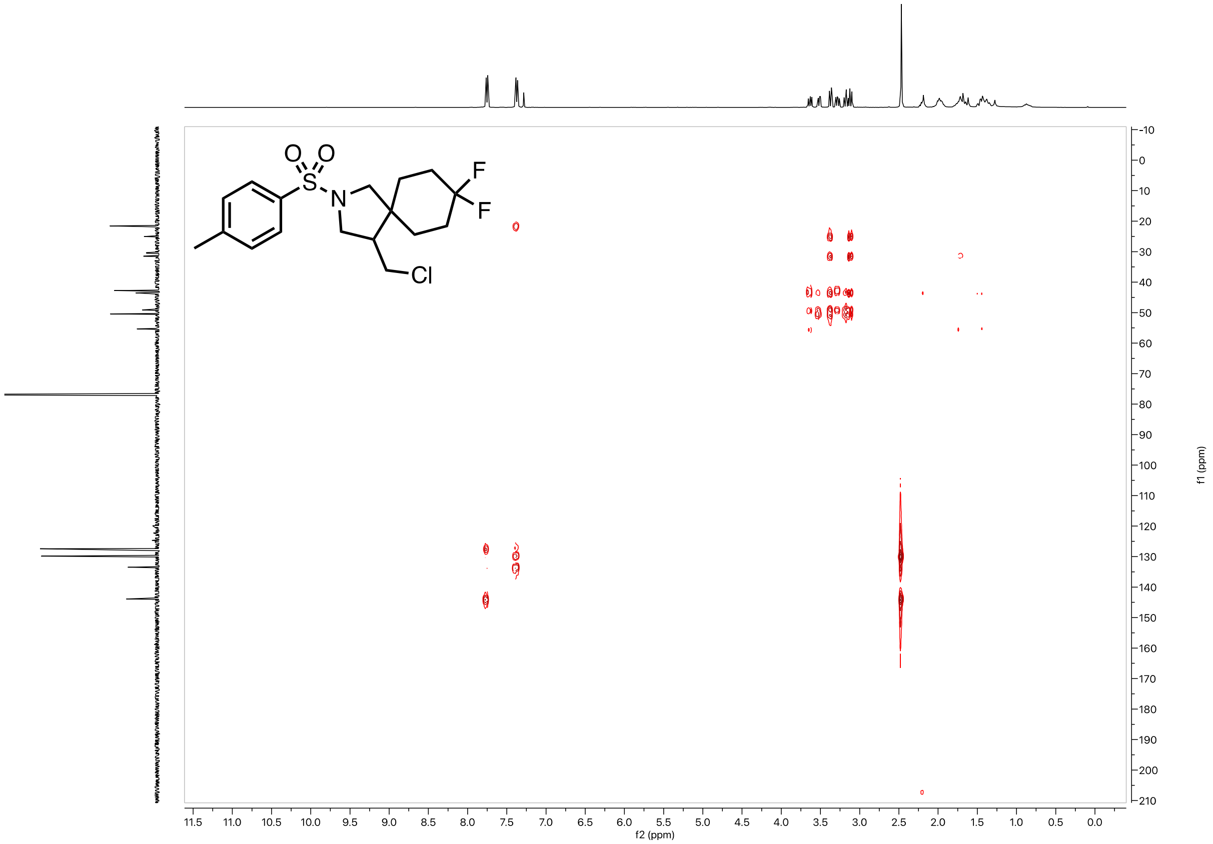

HMBC of *4-(chloromethyl)-8,8-difluoro-2-tosyl-2-azaspiro[4.5]decane* ($CDCl_3$).

12-(chloromethyl)-10-tosyl-1,4-dioxa-10-azadispiro[4.2.4^{8}.2^{5}]tetradecane (3r)

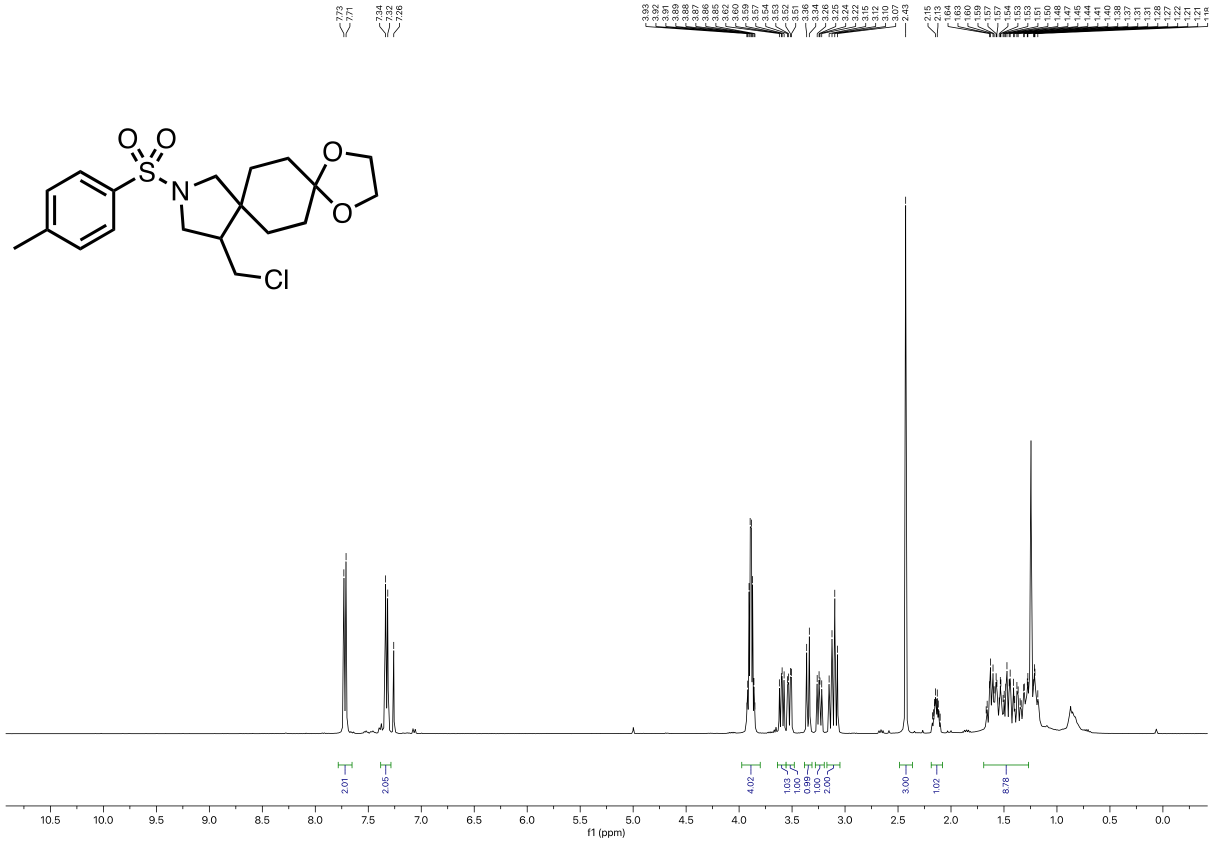

^{1}H NMR of *12-(chloromethyl)-10-tosyl-1,4-dioxa-10-azadispiro[4.2.4^{8}.2^{5}]tetradecane* (400 MHz, $CDCl_3$).

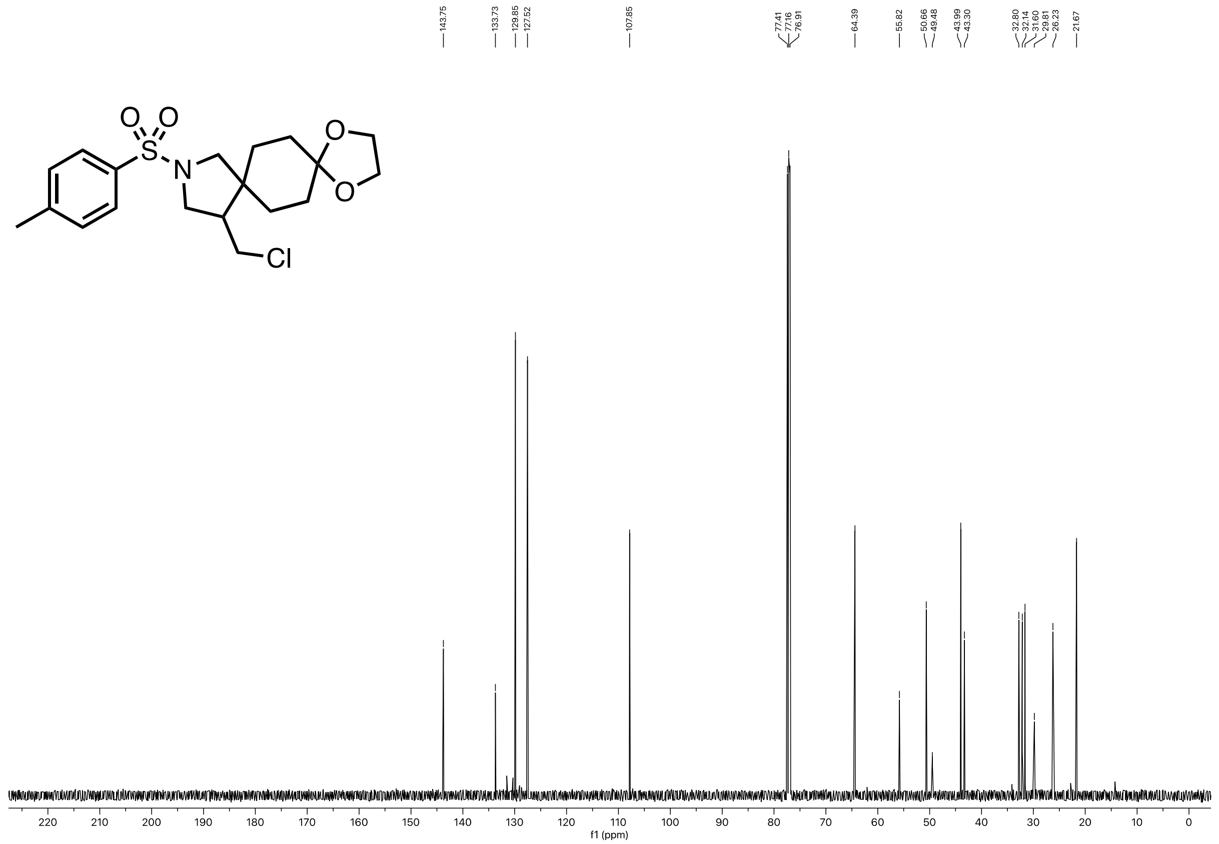

$^{13}C\{^1H\}$ NMR of *12-(chloromethyl)-10-tosyl-1,4-dioxa-10-azadispiro[4.2.4^8.2^5]tetradecane* (126 MHz, $CDCl_3$).

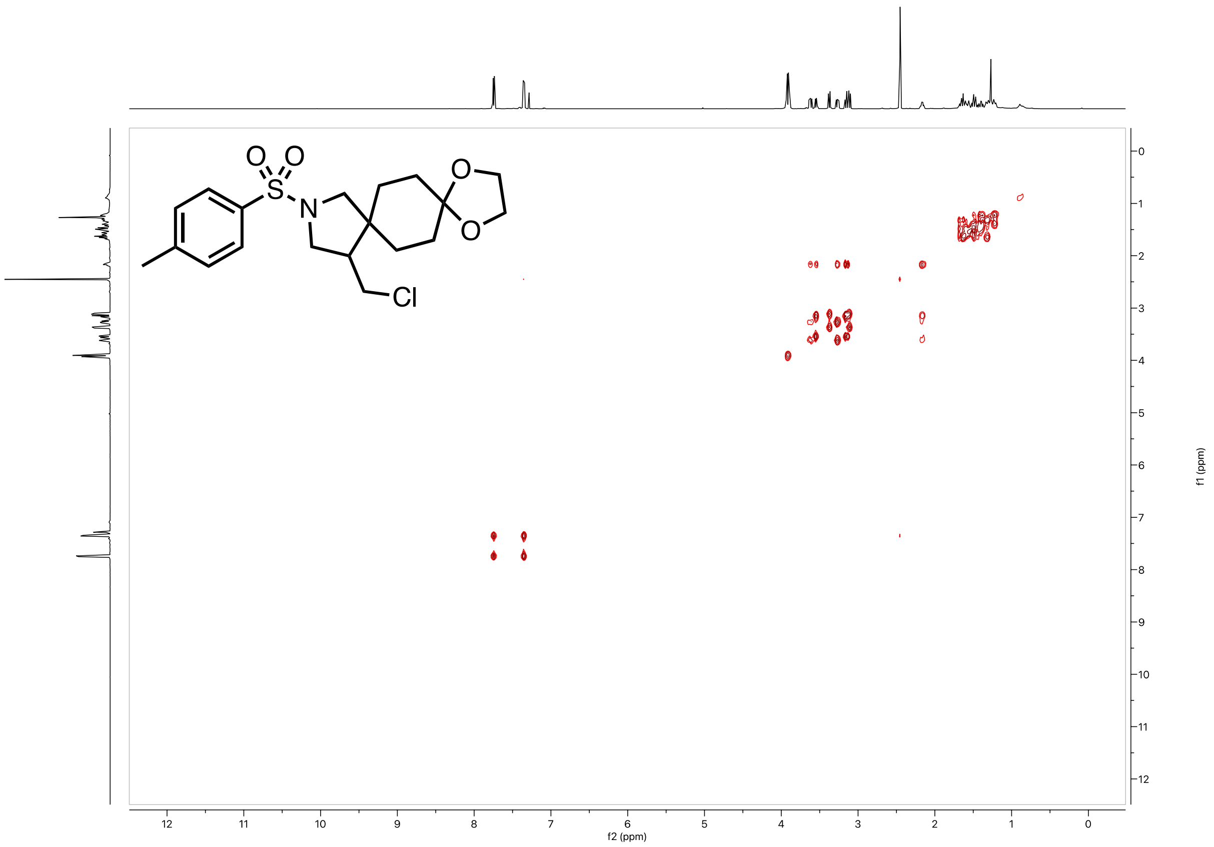

COSY of *12-(chloromethyl)-10-tosyl-1,4-dioxa-10-azadispiro[4.2.4^8.2^5]tetradecane* ($CDCl_3$).

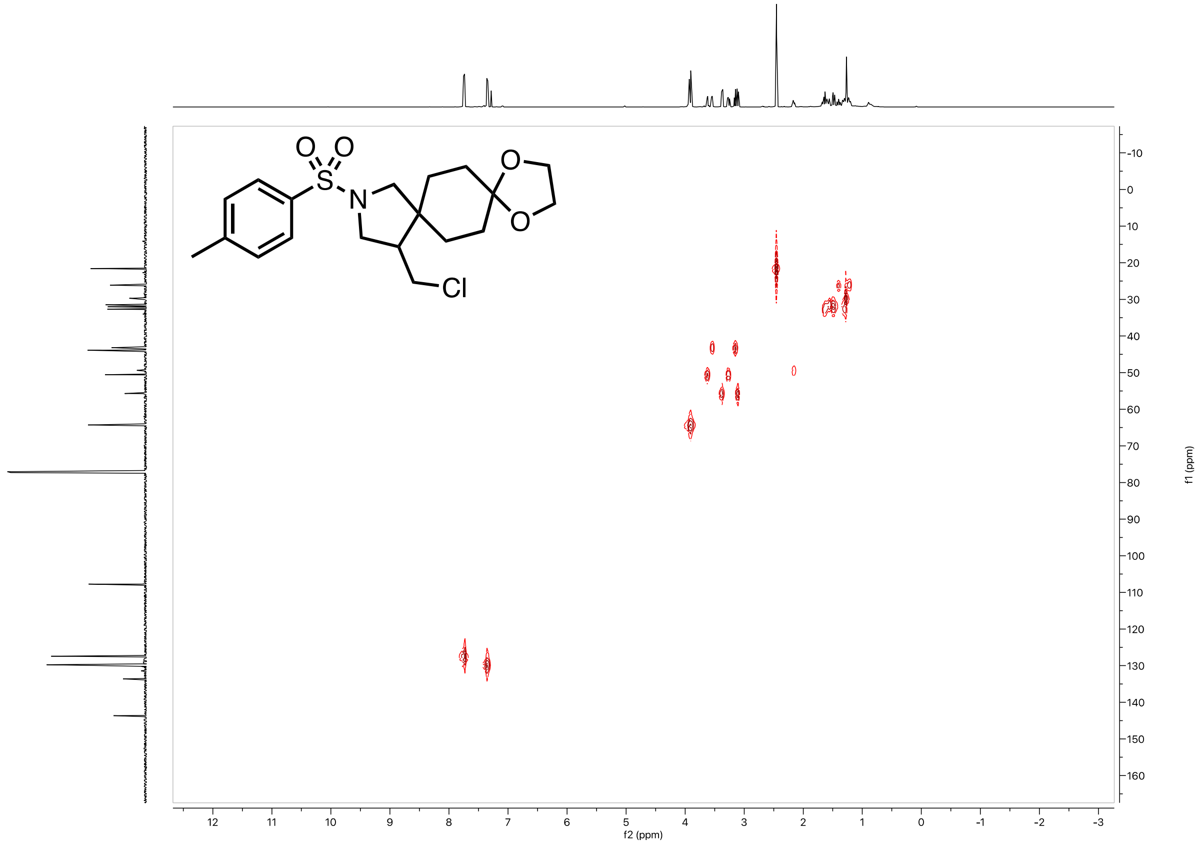

HSQC of *12-(chloromethyl)-10-tosyl-1,4-dioxa-10-azadispiro[4.2.4^{8}.2^{5}]tetradecane* ($CDCl_3$).

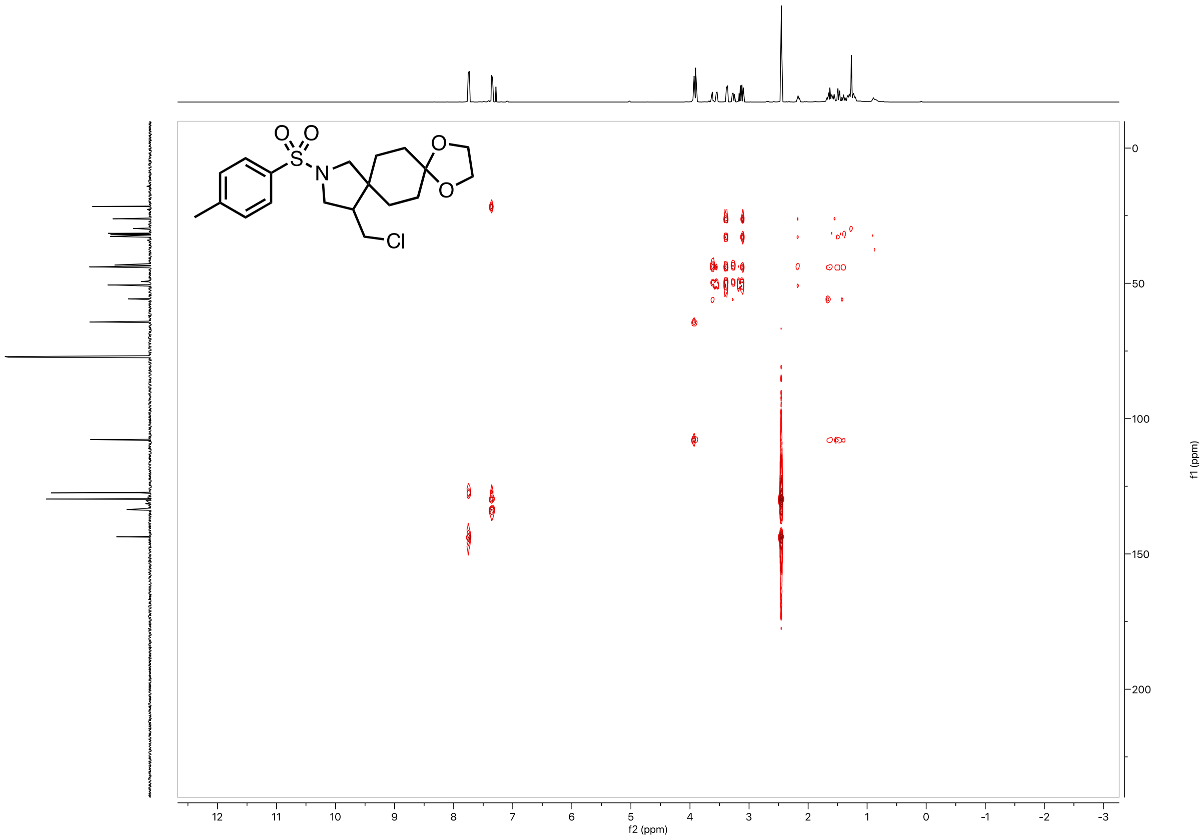

HMBC of *12-(chloromethyl)-10-tosyl-1,4-dioxa-10-azadispiro[4.2.4^{8}.2^{5}]tetradecane* ($CDCl_3$).

8-(chloromethyl)-6-tosyl-6-azaspiro[3.4]octane (3s)

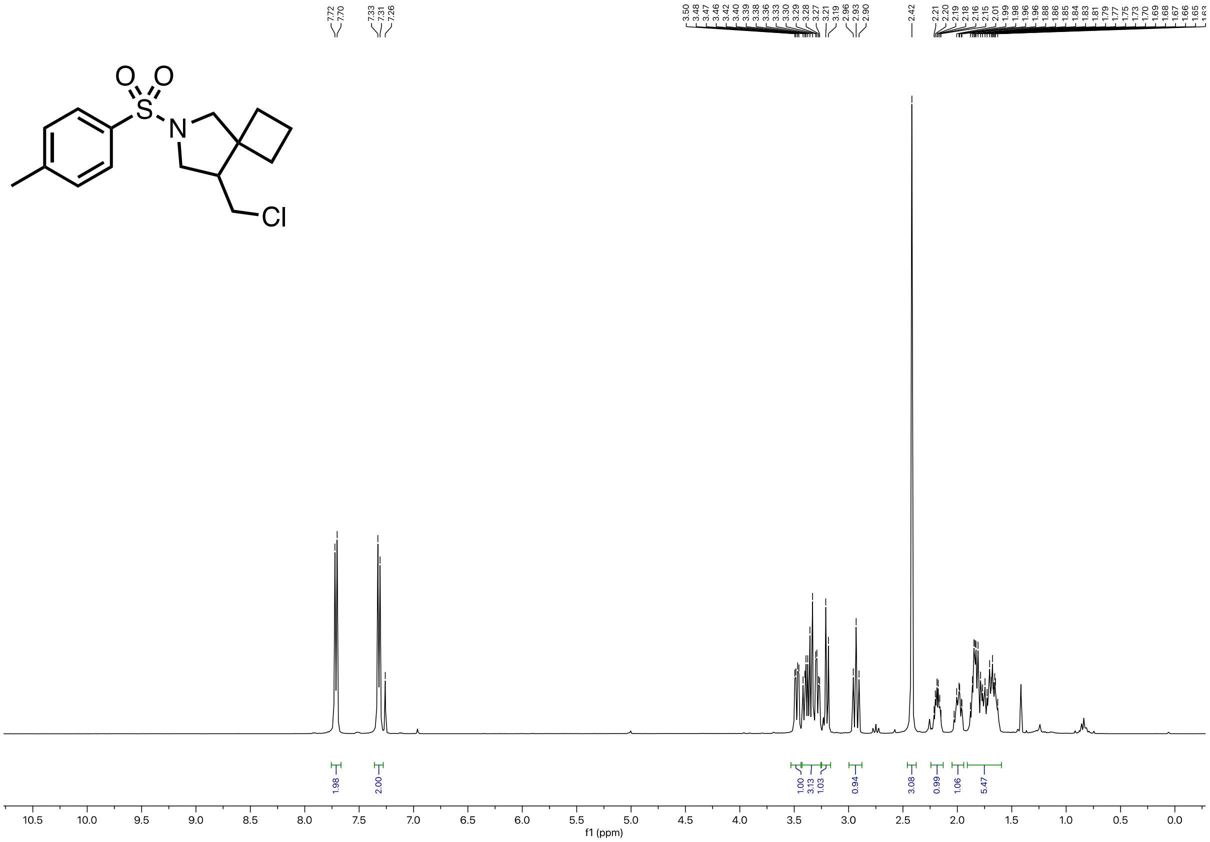

^{1}H NMR of *8-(chloromethyl)-6-tosyl-6-azaspiro[3.4]octane* (400 MHz, $CDCl_3$).

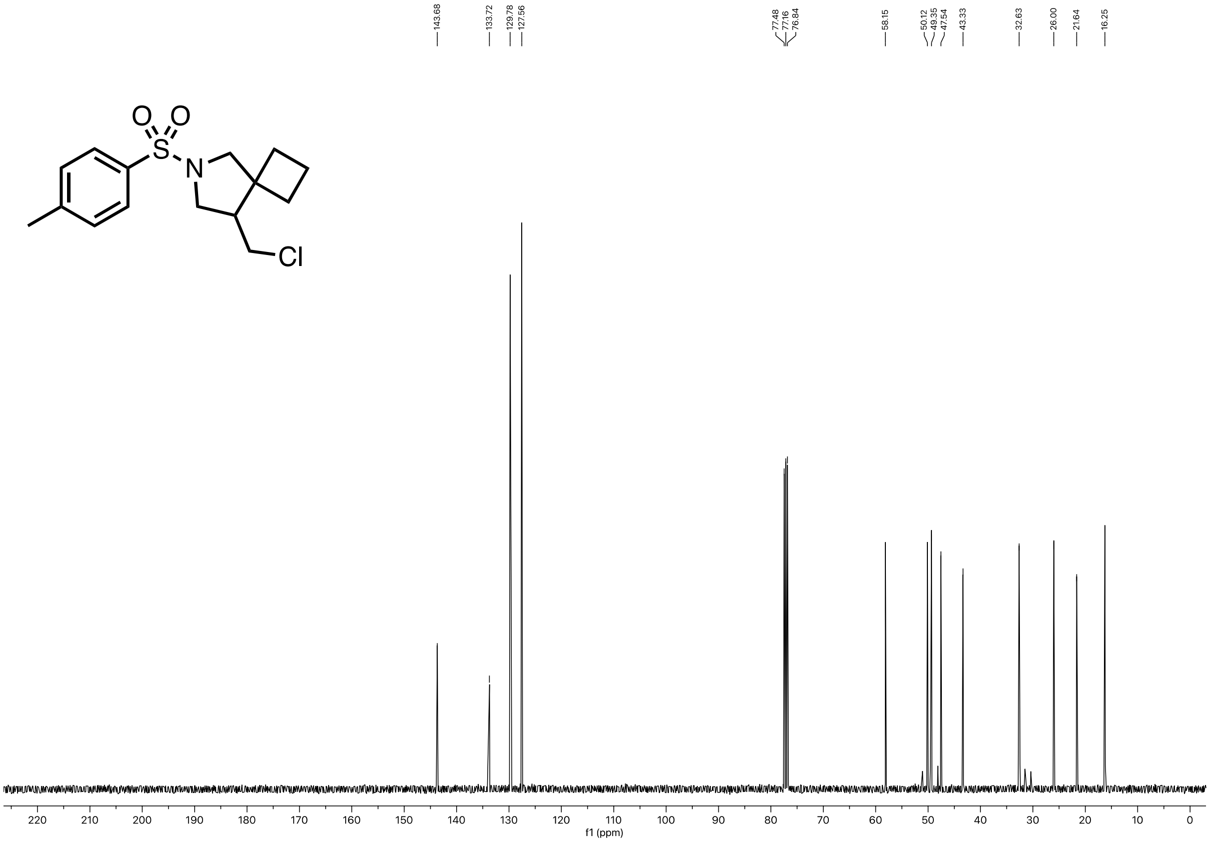

^{13}C{^{1}H} NMR of *8-(chloromethyl)-6-tosyl-6-azaspiro[3.4]octane* (101 MHz, $CDCl_3$).

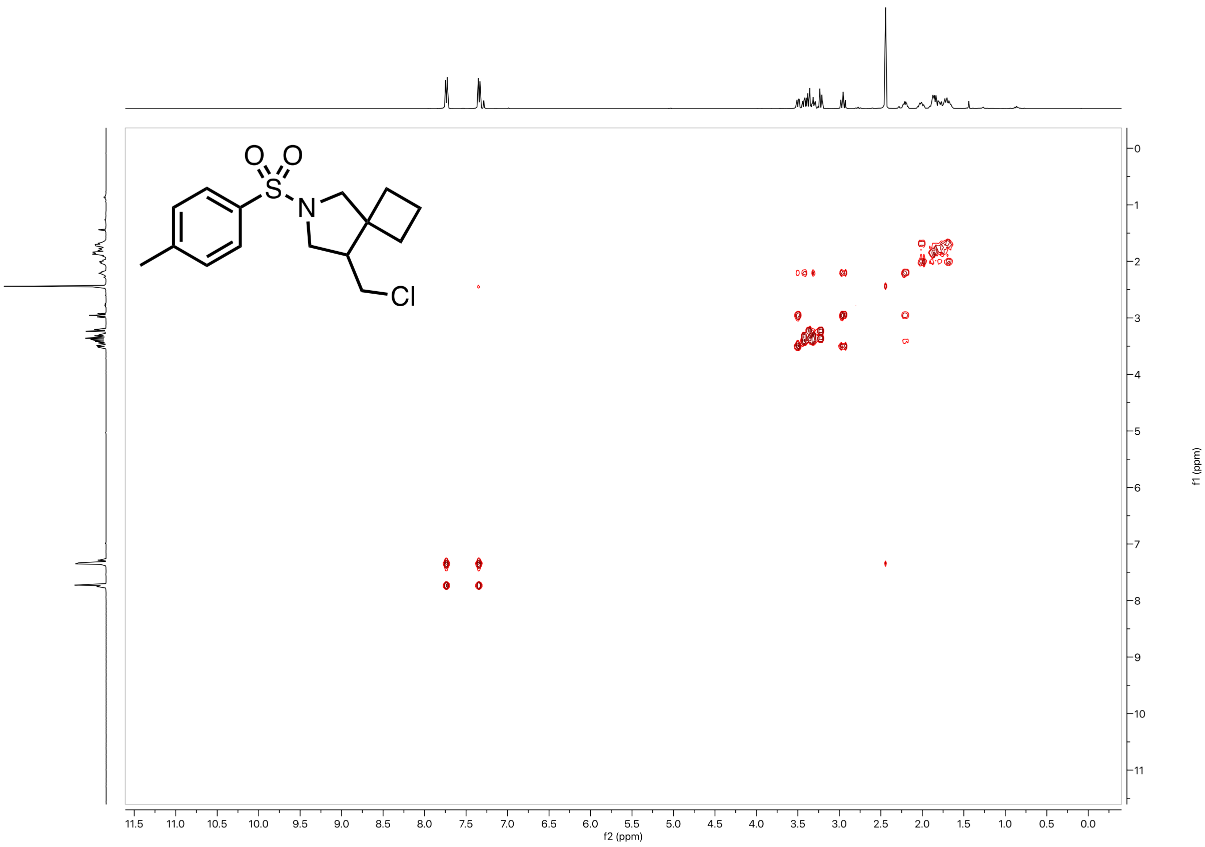

COSY of *8-(chloromethyl)-6-tosyl-6-azaspiro[3.4]octane* ($CDCl_3$).

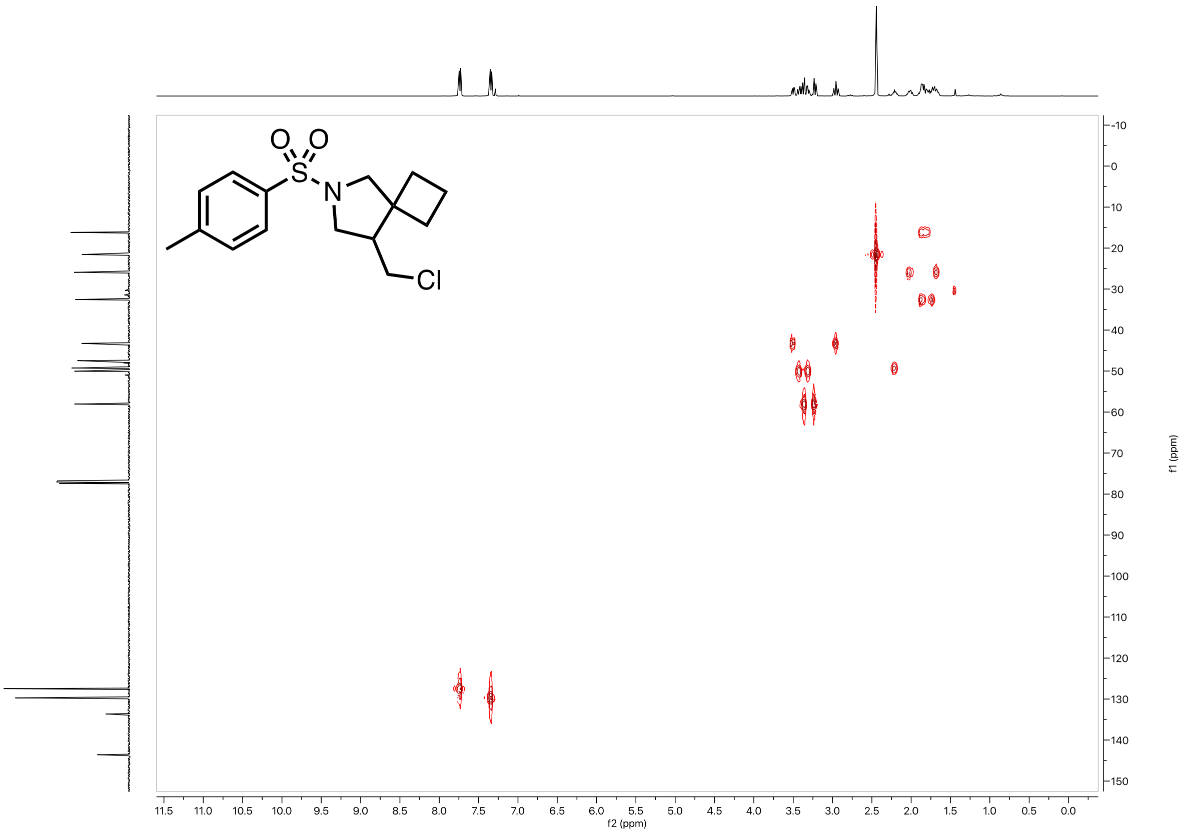

HSQC of *8-(chloromethyl)-6-tosyl-6-azaspiro[3.4]octane* ($CDCl_3$).

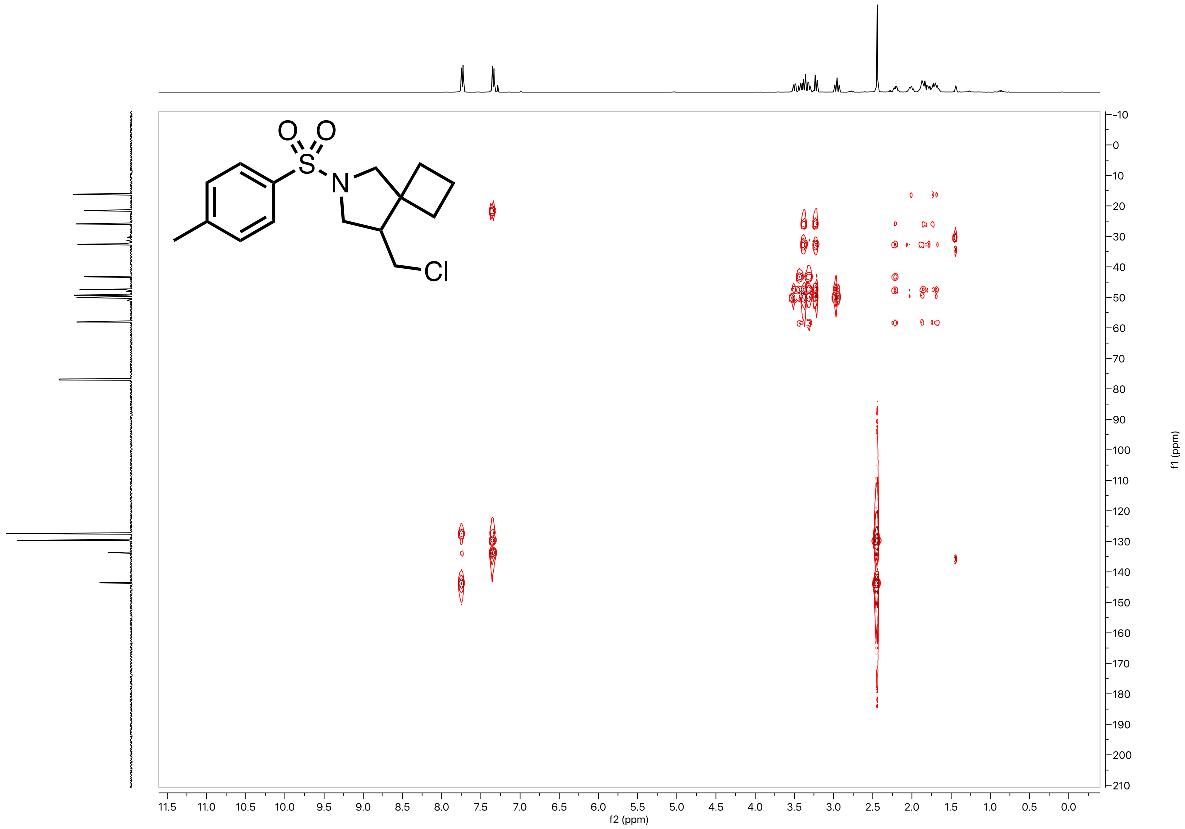

HMBC of *8-(chloromethyl)-6-tosyl-6-azaspiro[3.4]octane* ($CDCl_3$).

4-(chloromethyl)-2-tosyl-2-azaspiro[4.6]undecane (3t)

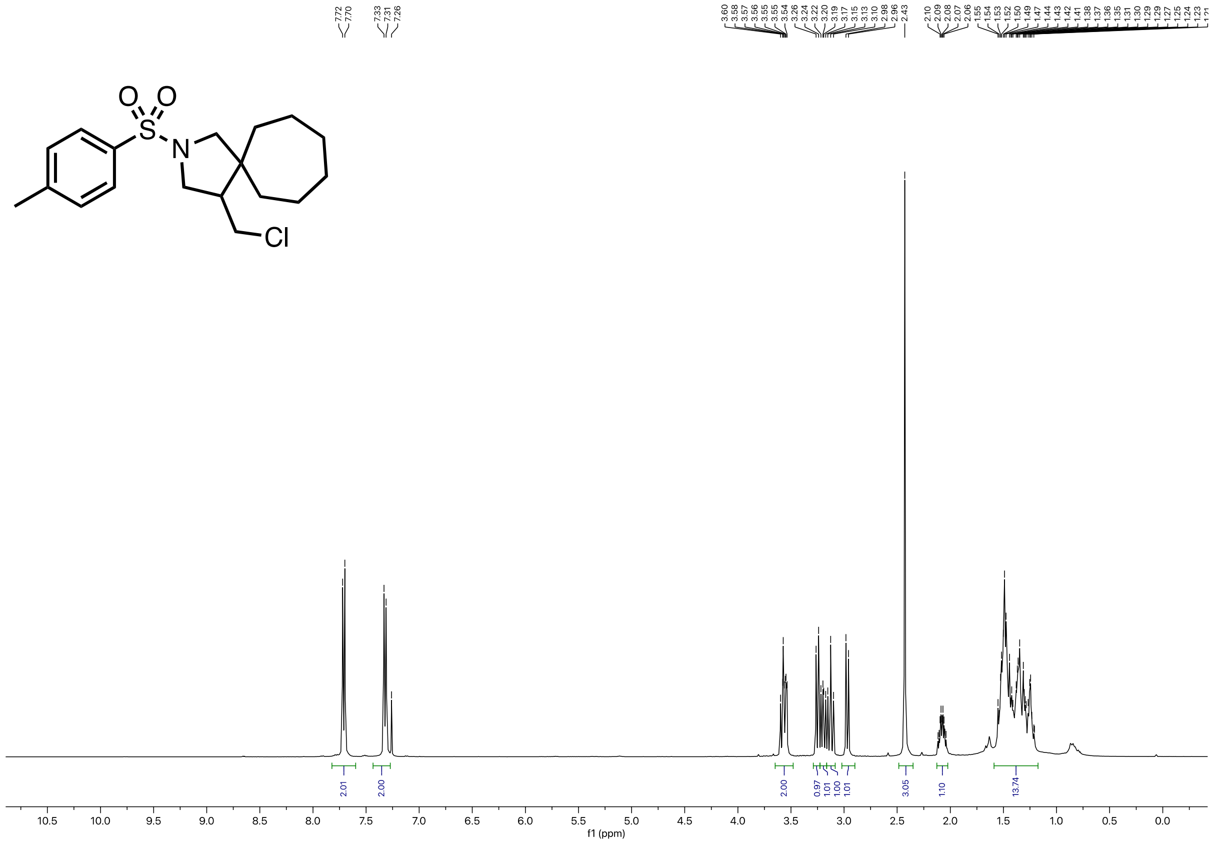

^{1}H NMR of *4-(chloromethyl)-2-tosyl-2-azaspiro[4.6]undecane* (400 MHz, $CDCl_3$).

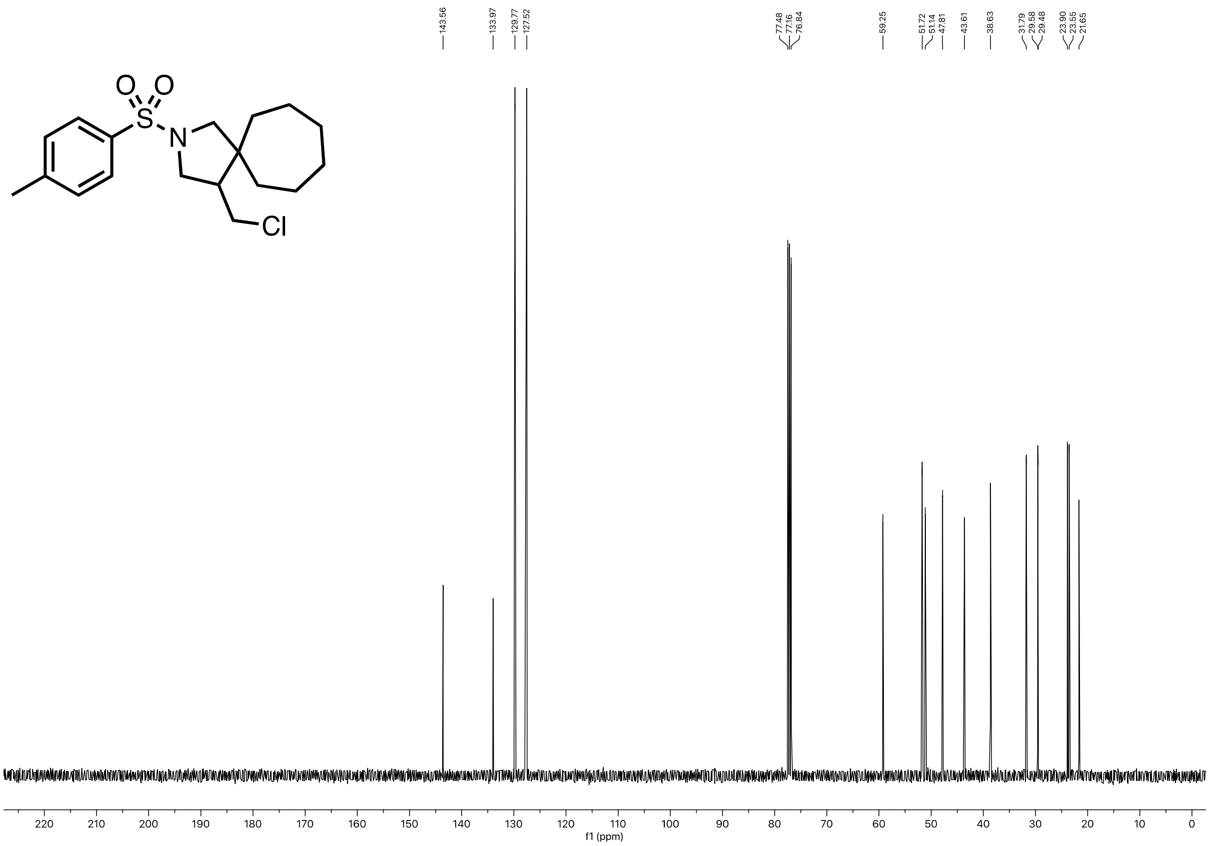

$^{13}C\{^{1}H\}$ NMR of *4-(chloromethyl)-2-tosyl-2-azaspiro[4.6]undecane* (101 MHz, $CDCl_3$).

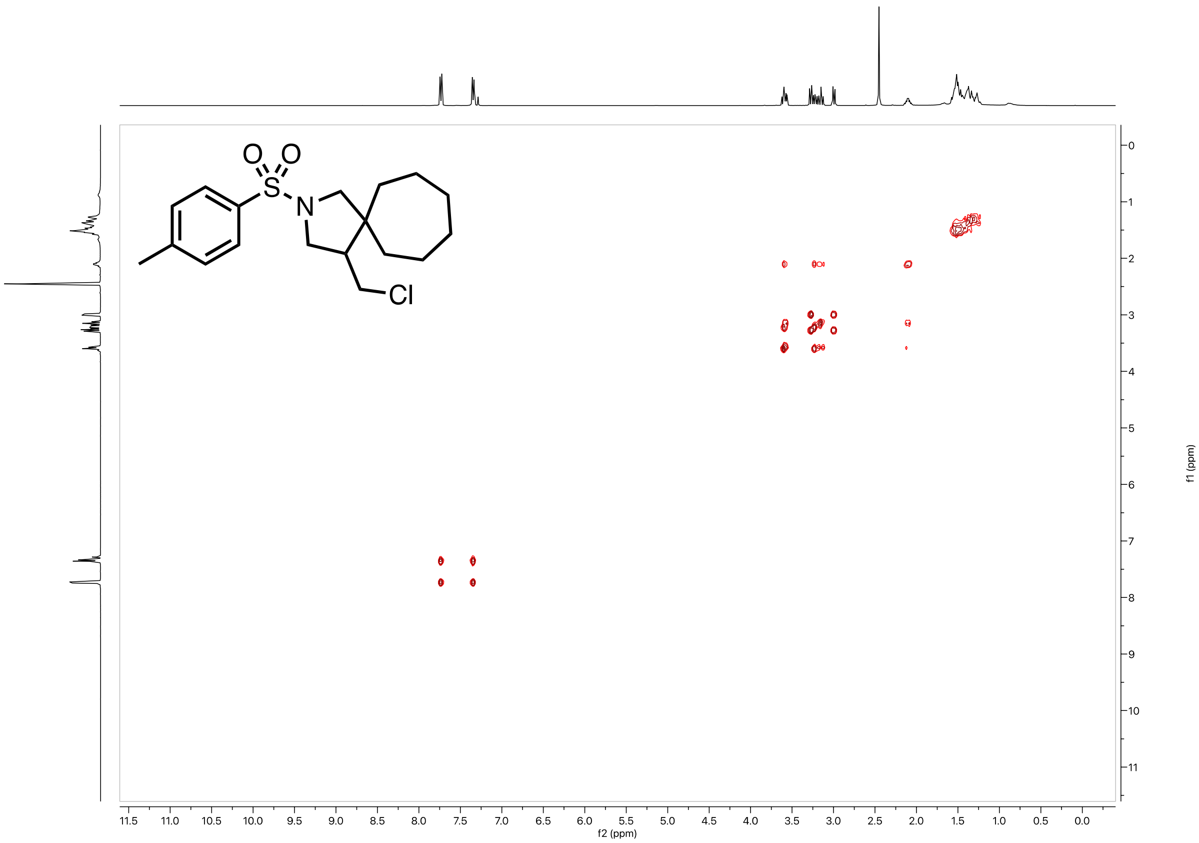

COSY of *4-(chloromethyl)-2-tosyl-2-azaspiro[4.6]undecane* ($CDCl_3$).

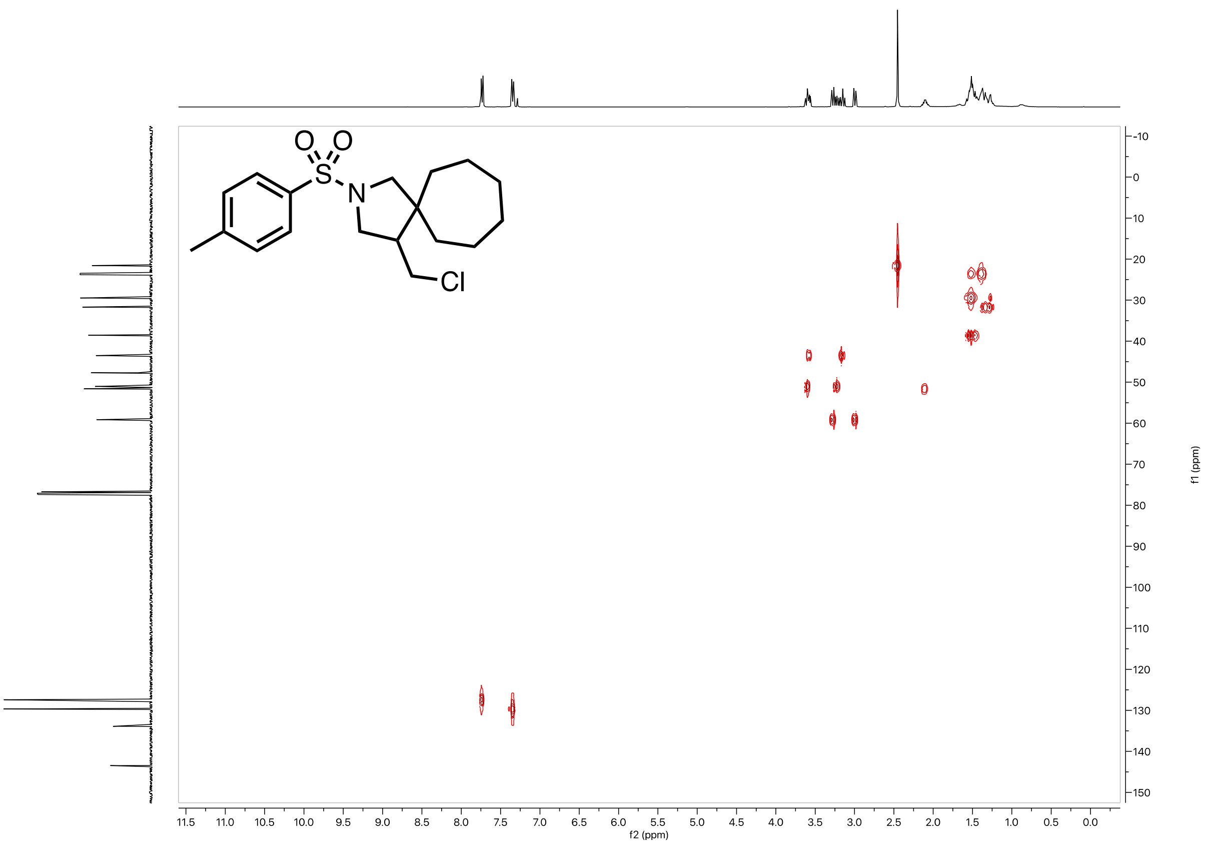

HSQC of *4-(chloromethyl)-2-tosyl-2-azaspiro[4.6]undecane* ($CDCl_3$).

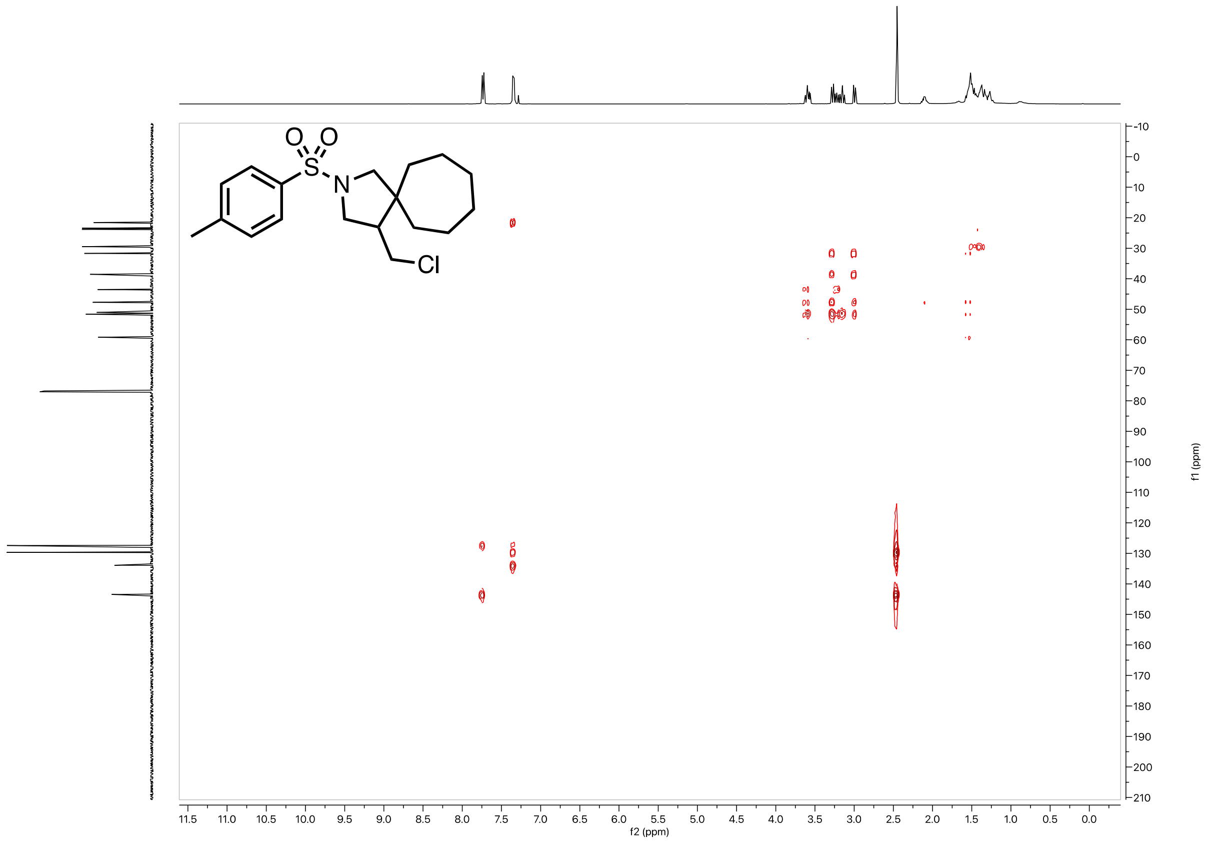

HMBC of *4-(chloromethyl)-2-tosyl-2-azaspiro[4.6]undecane* ($CDCl_3$).

4-(chloromethyl)-2-tosyl-2-azaspiro[4.11]hexadecane (3u)

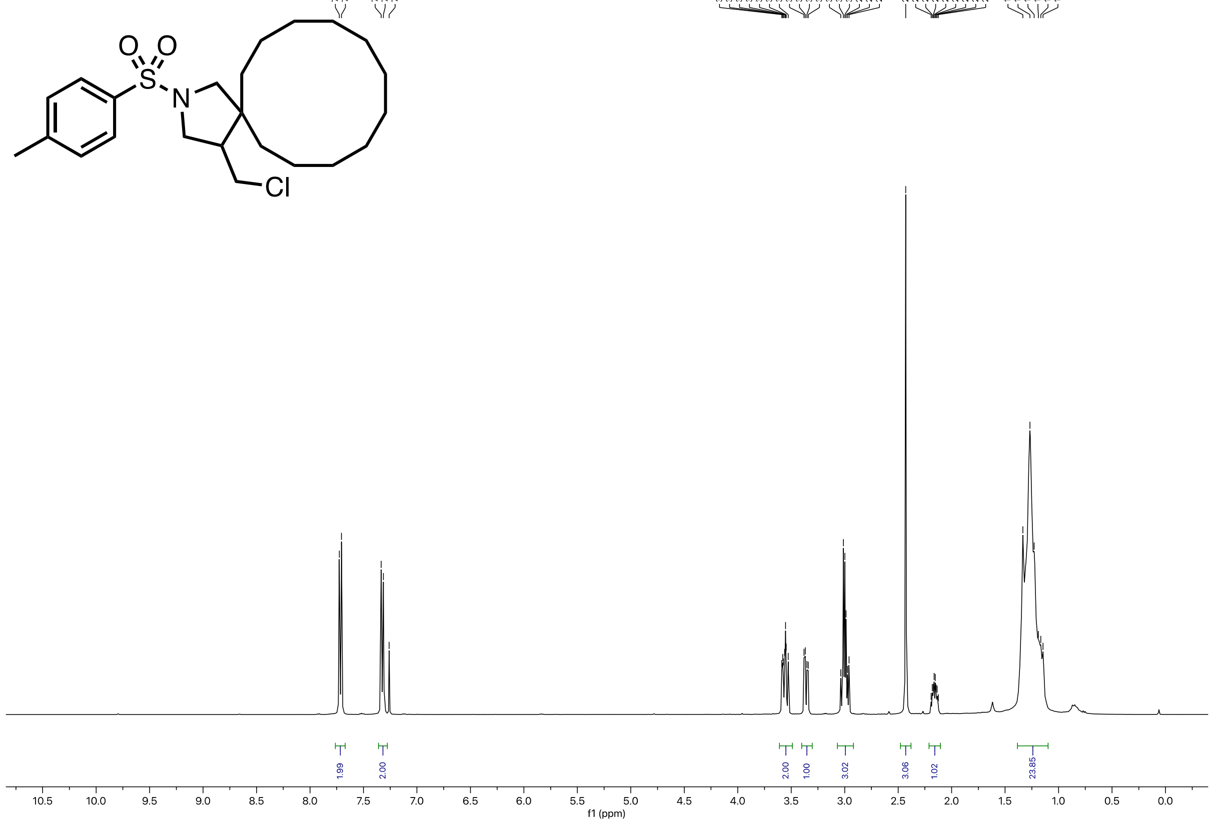

^{1}H NMR of *4-(chloromethyl)-2-tosyl-2-azaspiro[4.11]hexadecane* (400 MHz, $CDCl_3$).

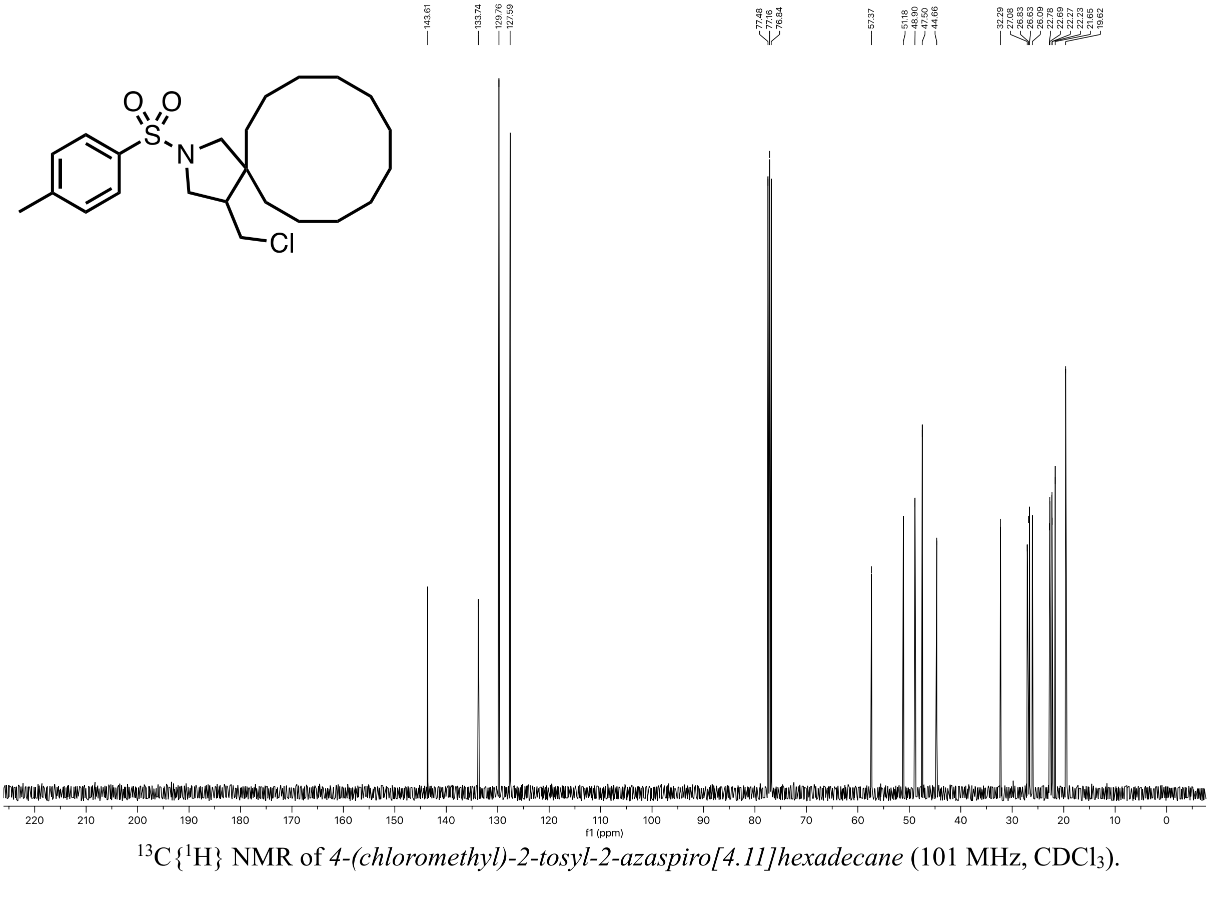

$^{13}C\{^{1}H\}$ NMR of *4-(chloromethyl)-2-tosyl-2-azaspiro[4.11]hexadecane* (101 MHz, $CDCl_3$).

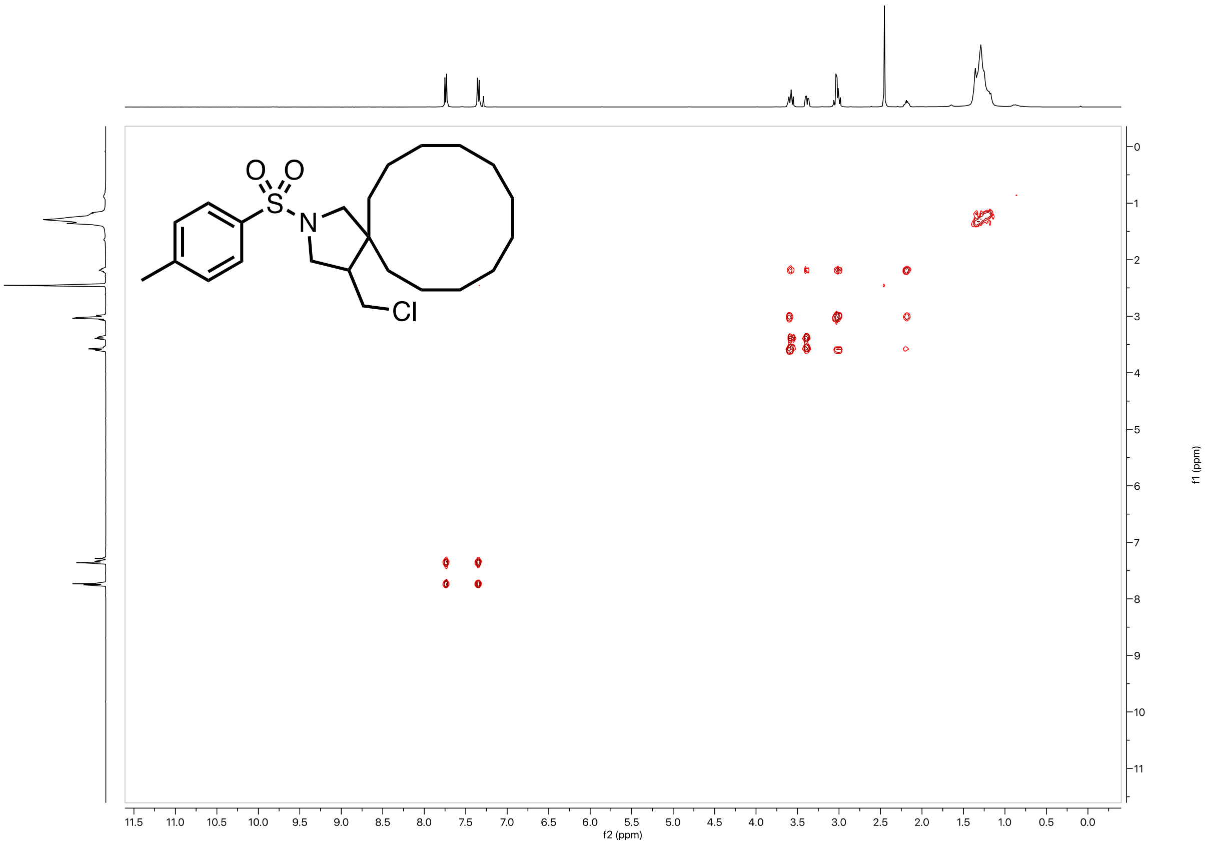

COSY of *4-(chloromethyl)-2-tosyl-2-azaspiro[4.11]hexadecane* ($CDCl_3$).

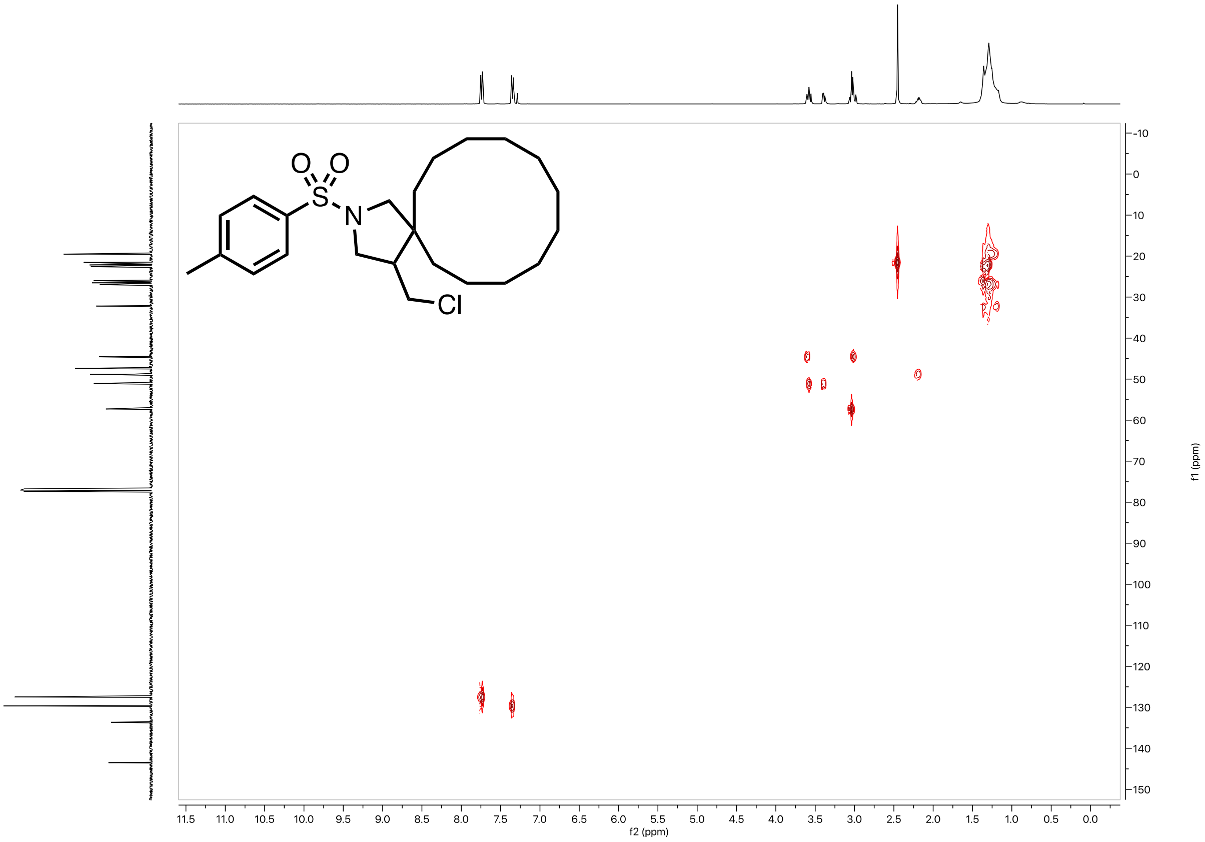

HSQC of *4-(chloromethyl)-2-tosyl-2-azaspiro[4.11]hexadecane* ($CDCl_3$).

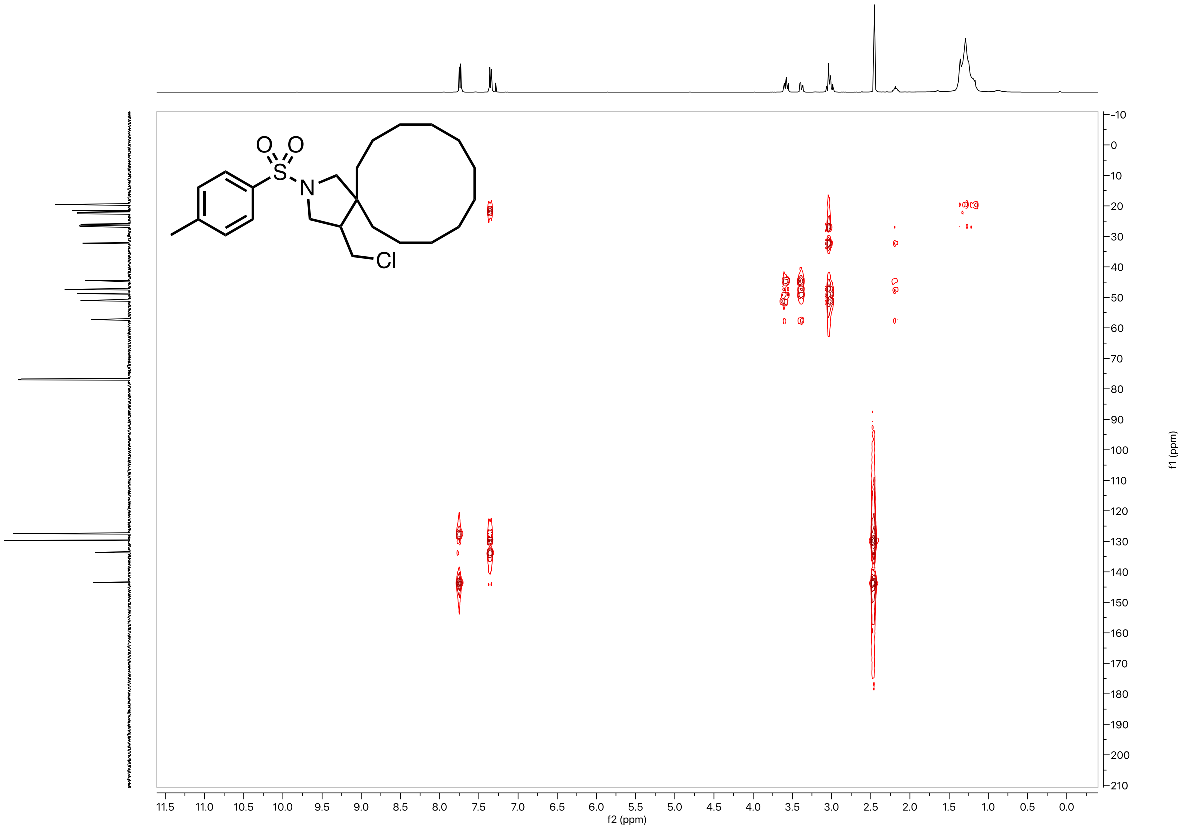

HMBC of *4-(chloromethyl)-2-tosyl-2-azaspiro[4.11]hexadecane* ($CDCl_3$).

4-(chloromethyl)-7-methyl-2-tosyl-2-azaspiro[4.14]nonadecane (3v)

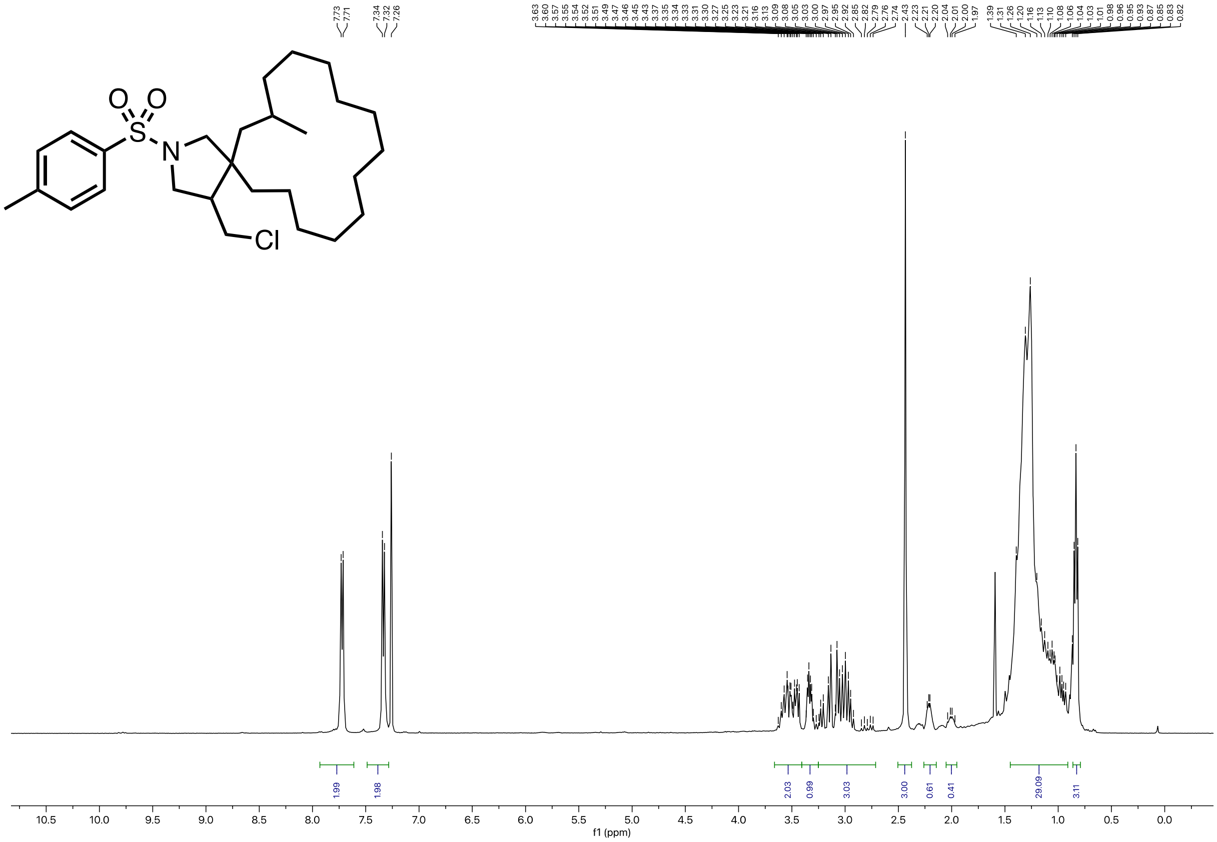

^{1}H NMR of *4-(chloromethyl)-7-methyl-2-tosyl-2-azaspiro[4.14]nonadecane* (400 MHz, $CDCl_3$).

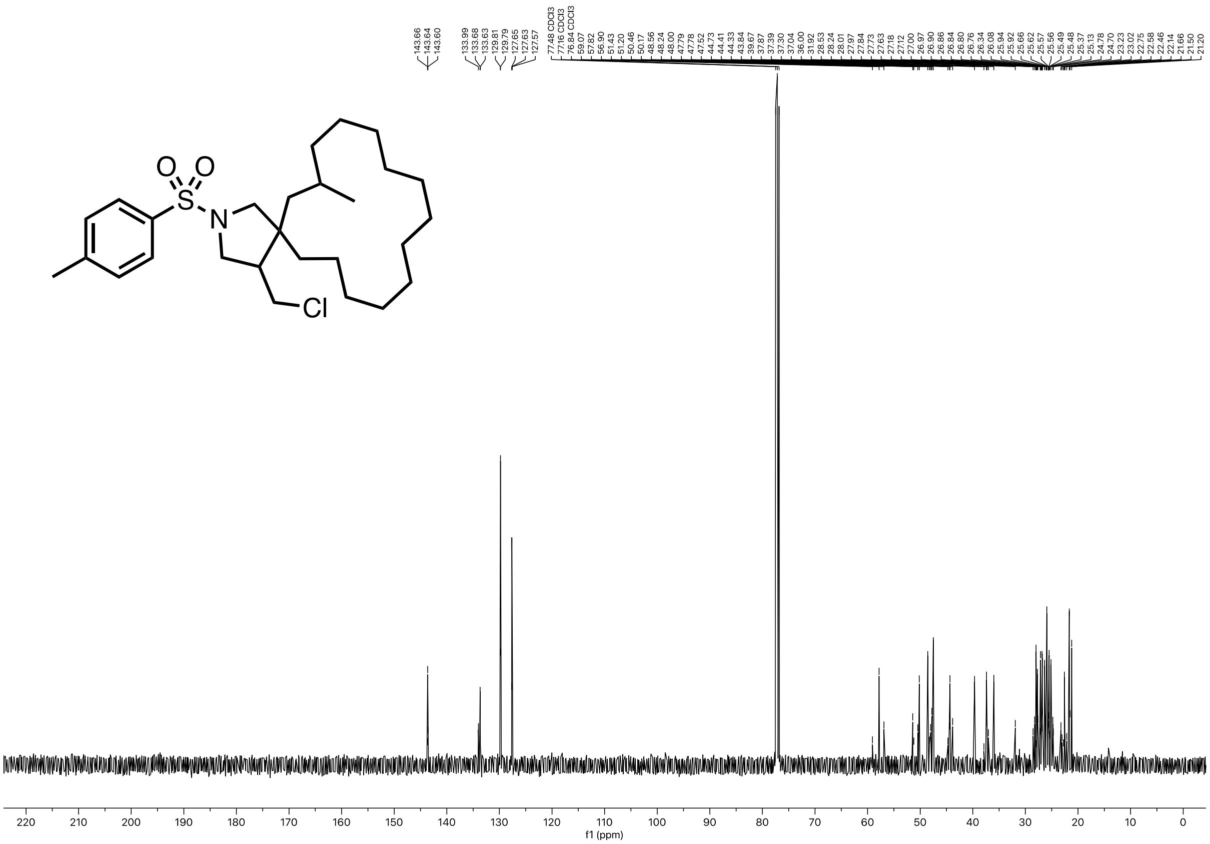

$^{13}C\{^{1}H\}$ NMR of *4-(chloromethyl)-7-methyl-2-tosyl-2-azaspiro[4.14]nonadecane* (101 MHz, $CDCl_3$).

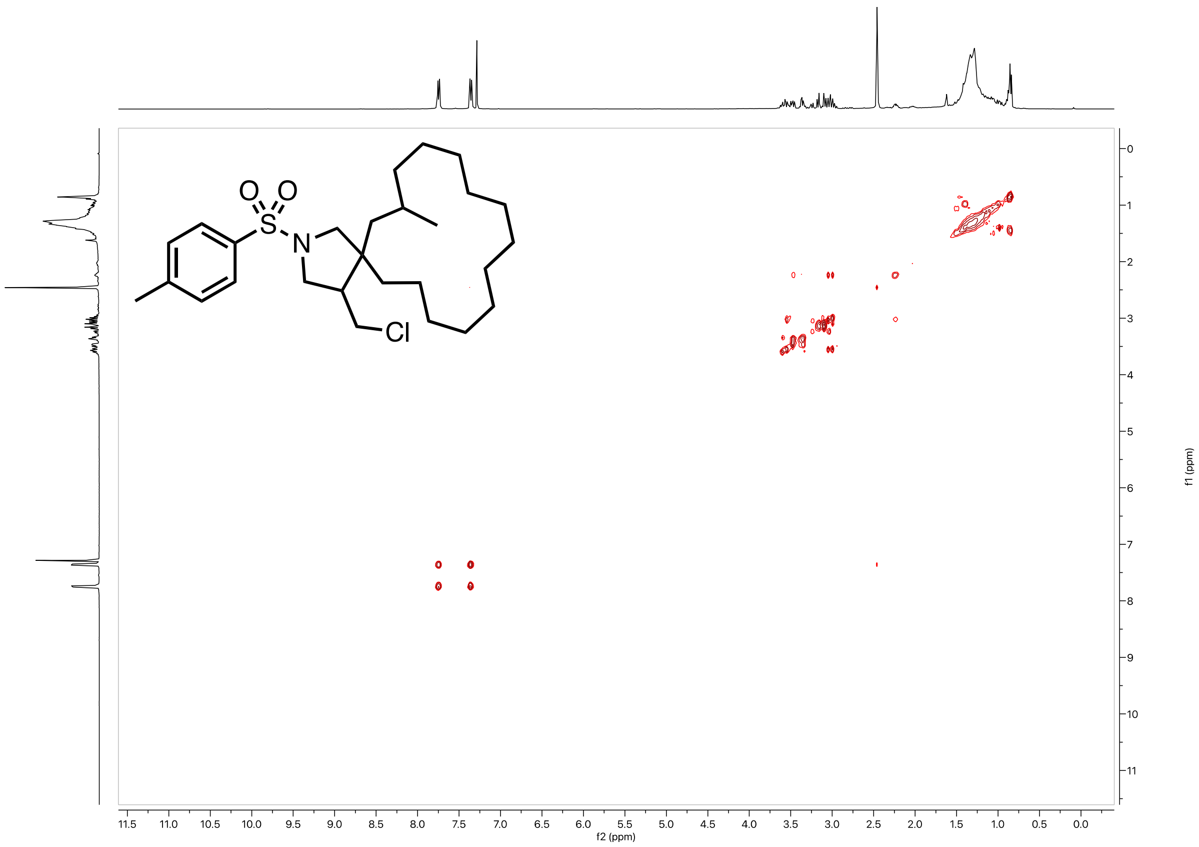

COSY of *4-(chloromethyl)-7-methyl-2-tosyl-2-azaspiro[4.14]nonadecane* ($CDCl_3$).

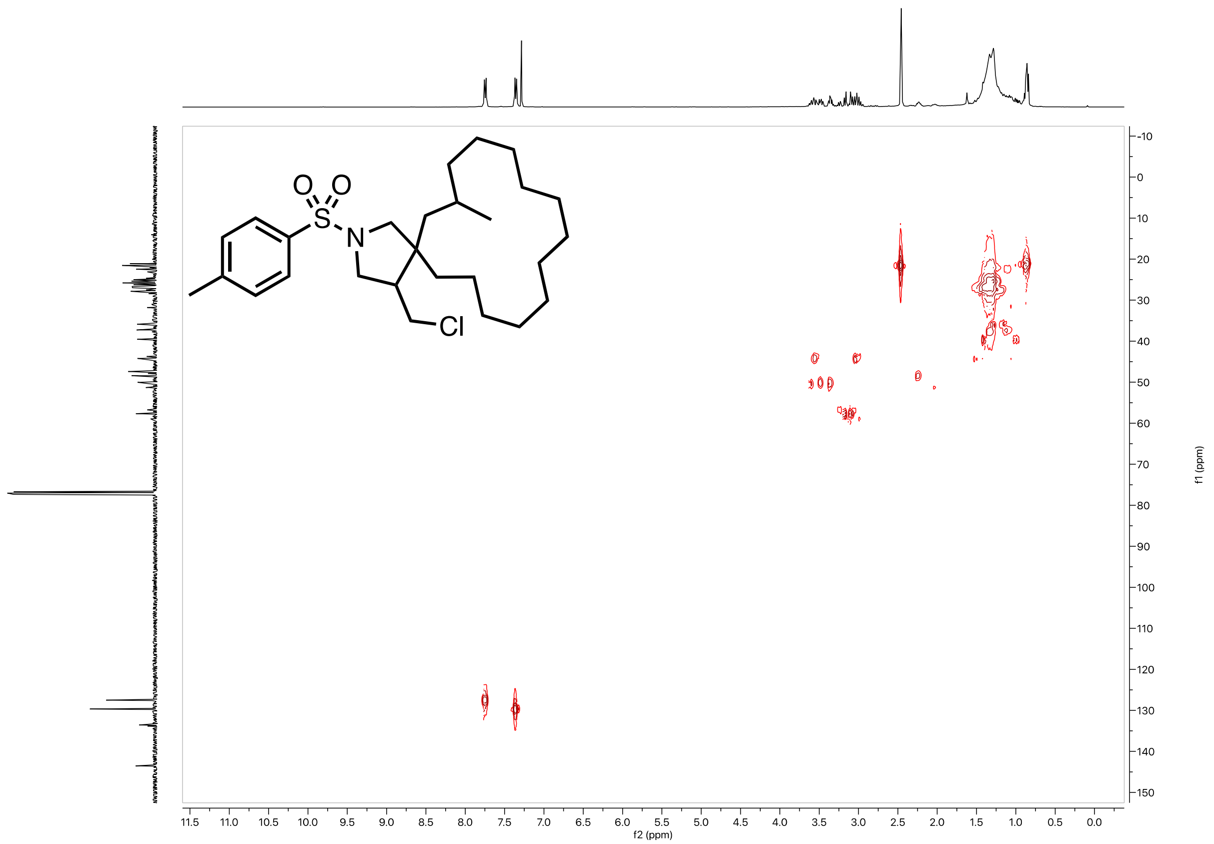

HSQC of *4-(chloromethyl)-7-methyl-2-tosyl-2-azaspiro[4.14]nonadecane* ($CDCl_3$).

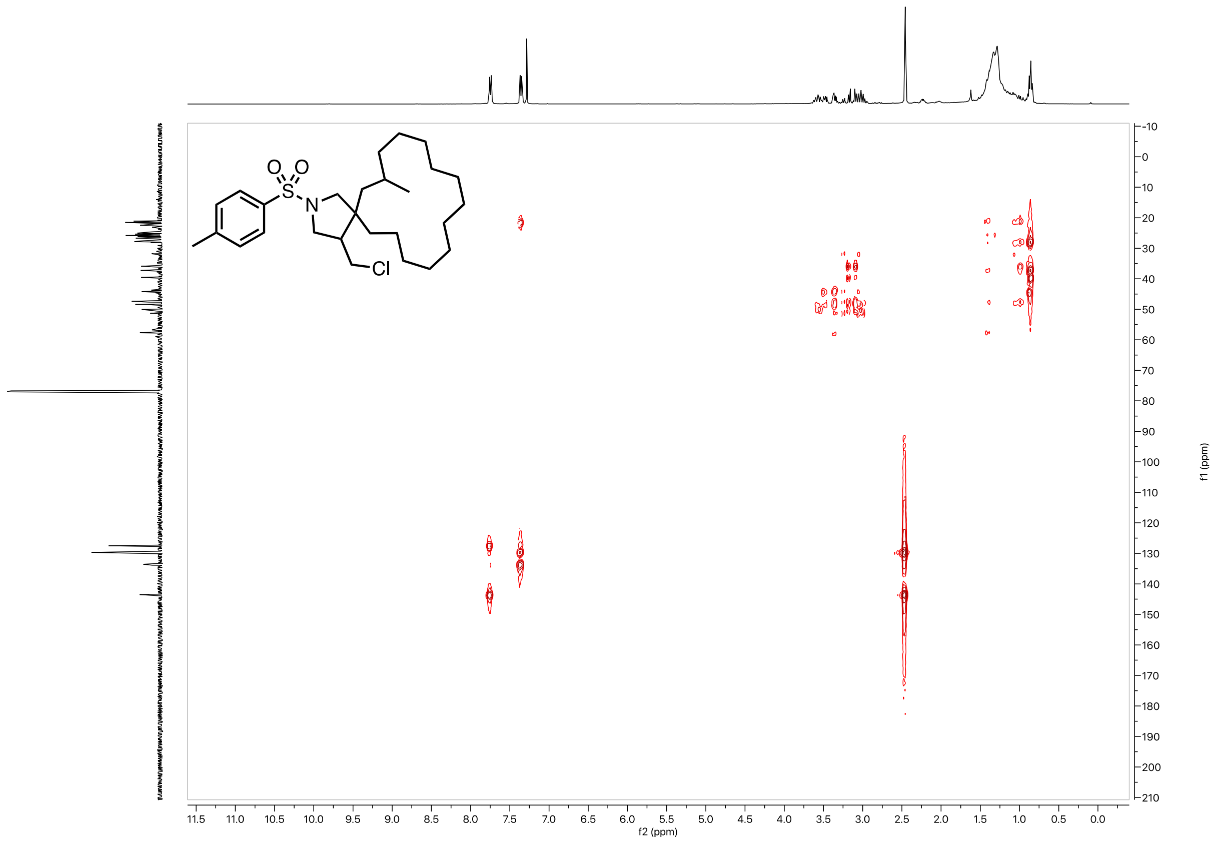

HMBC of *4-(chloromethyl)-7-methyl-2-tosyl-2-azaspiro[4.14]nonadecane* ($CDCl_3$).

11-(chloromethyl)-9-tosyl-2-oxa-9-azadispiro[3.2.4^7.2^4]tridecane (3w)

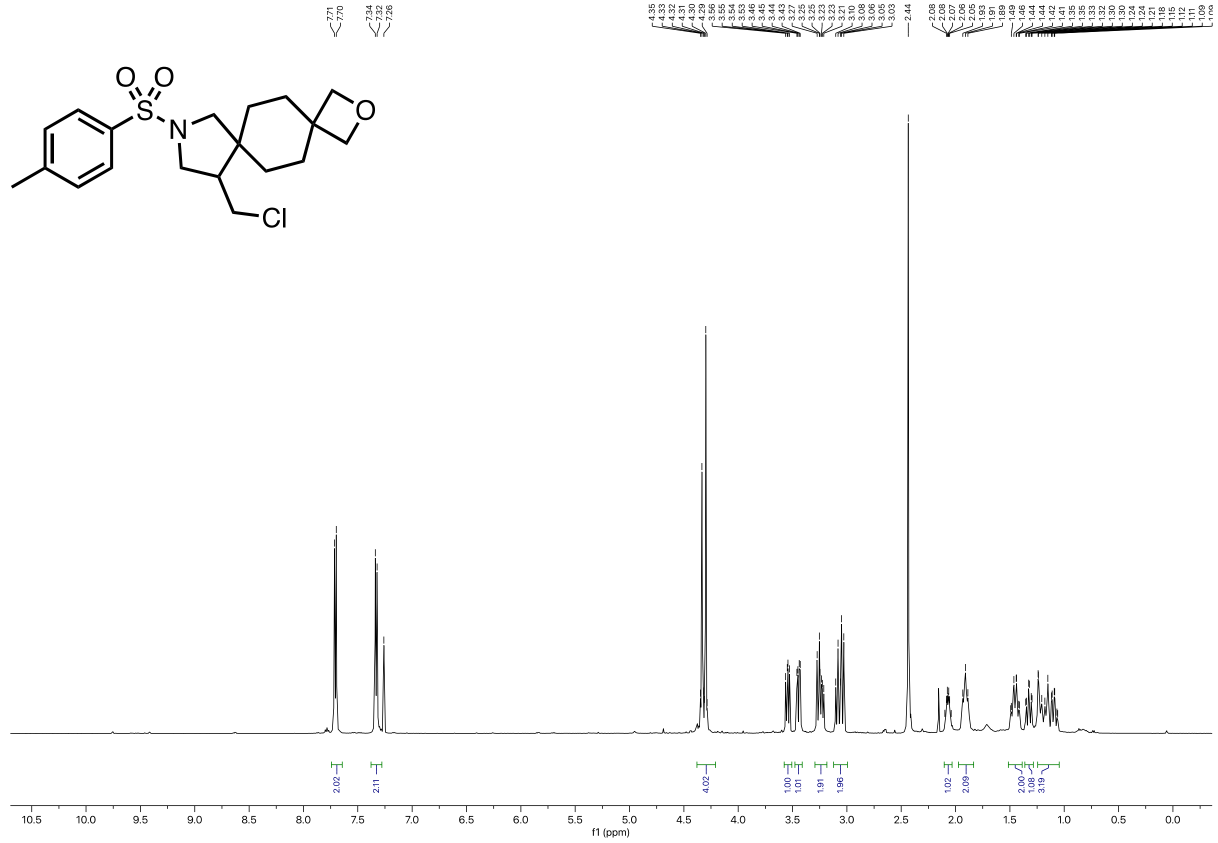

^{1}H NMR of *11-(chloromethyl)-9-tosyl-2-oxa-9-azadispiro[3.2.4^7.2^4]tridecane* (500 MHz, $CDCl_3$).

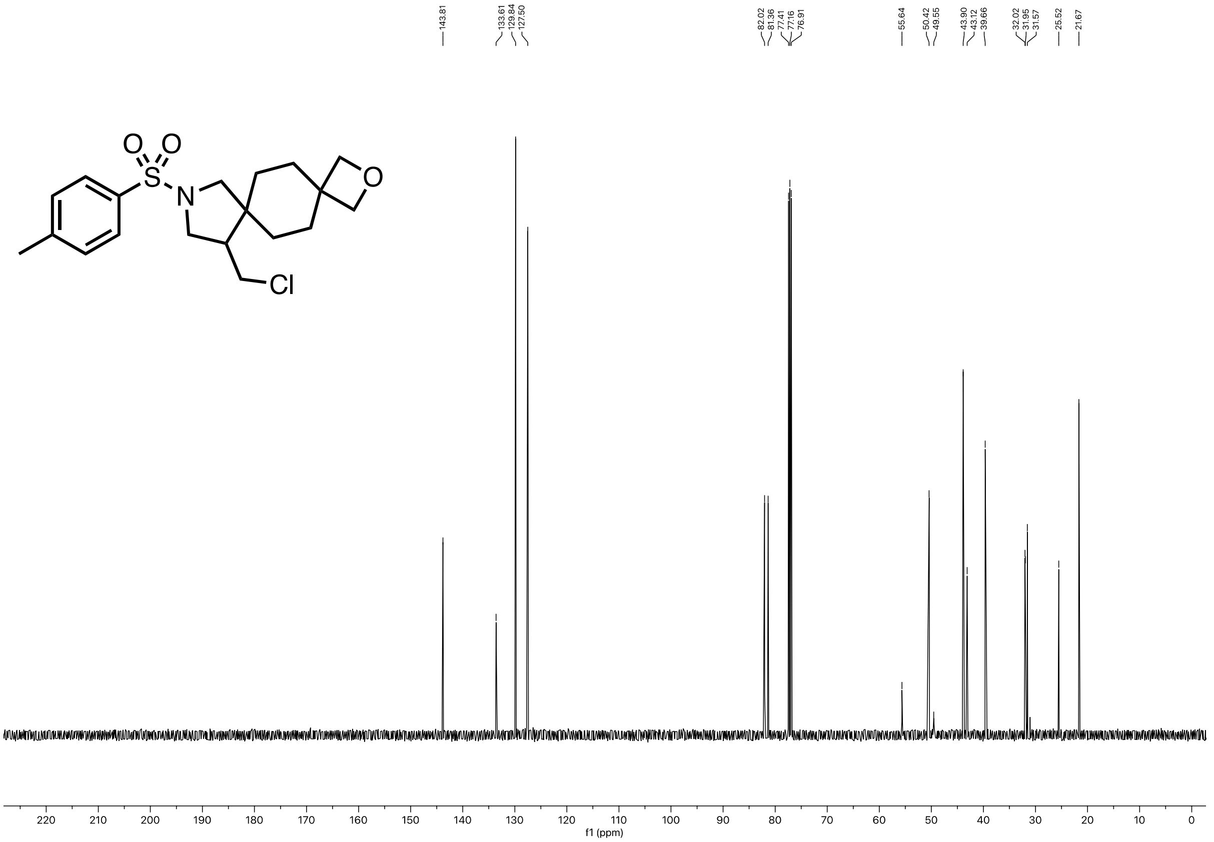

$^{13}C\{^1H\}$ NMR of *11-(chloromethyl)-9-tosyl-2-oxa-9-azadispiro[3.2.4^7.2^4]tridecane* (126 MHz, $CDCl_3$).

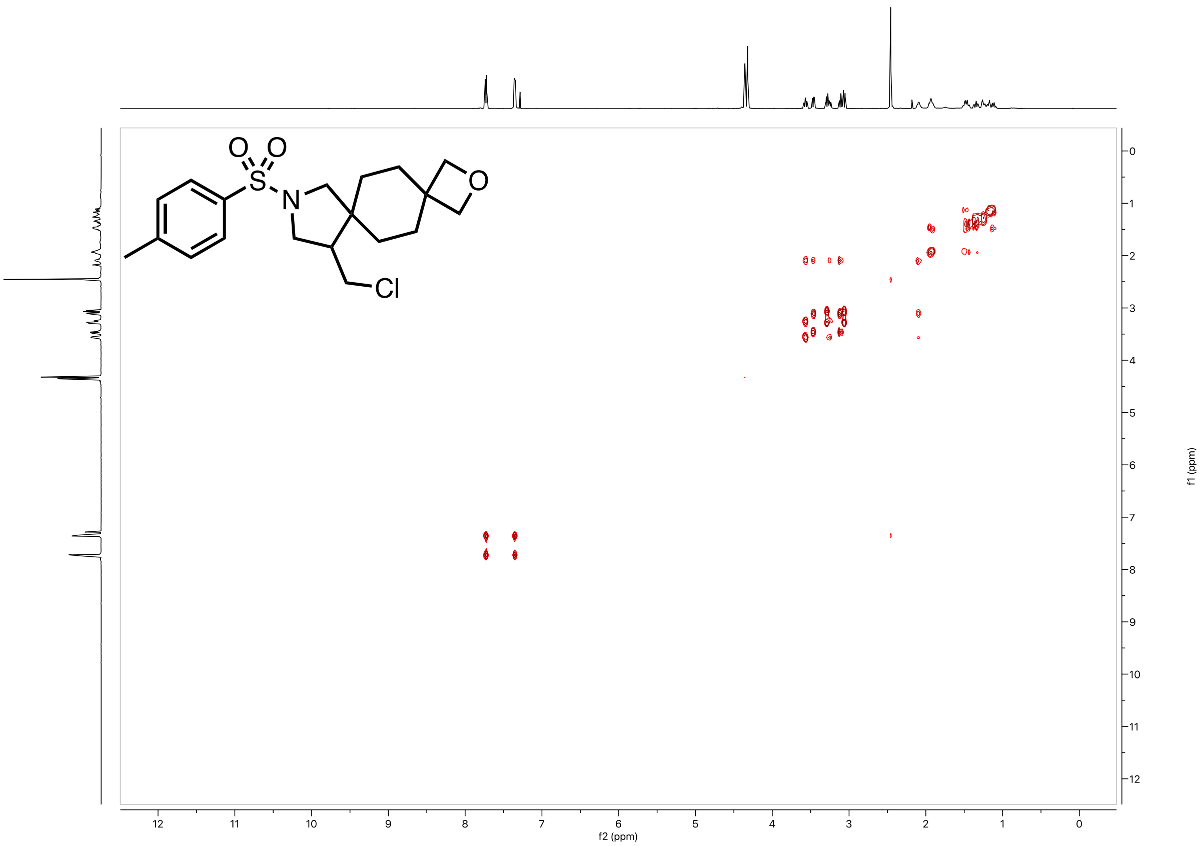

COSY of *11-(chloromethyl)-9-tosyl-2-oxa-9-azadispiro[3.2.4^7.2^4]tridecane* ($CDCl_3$).

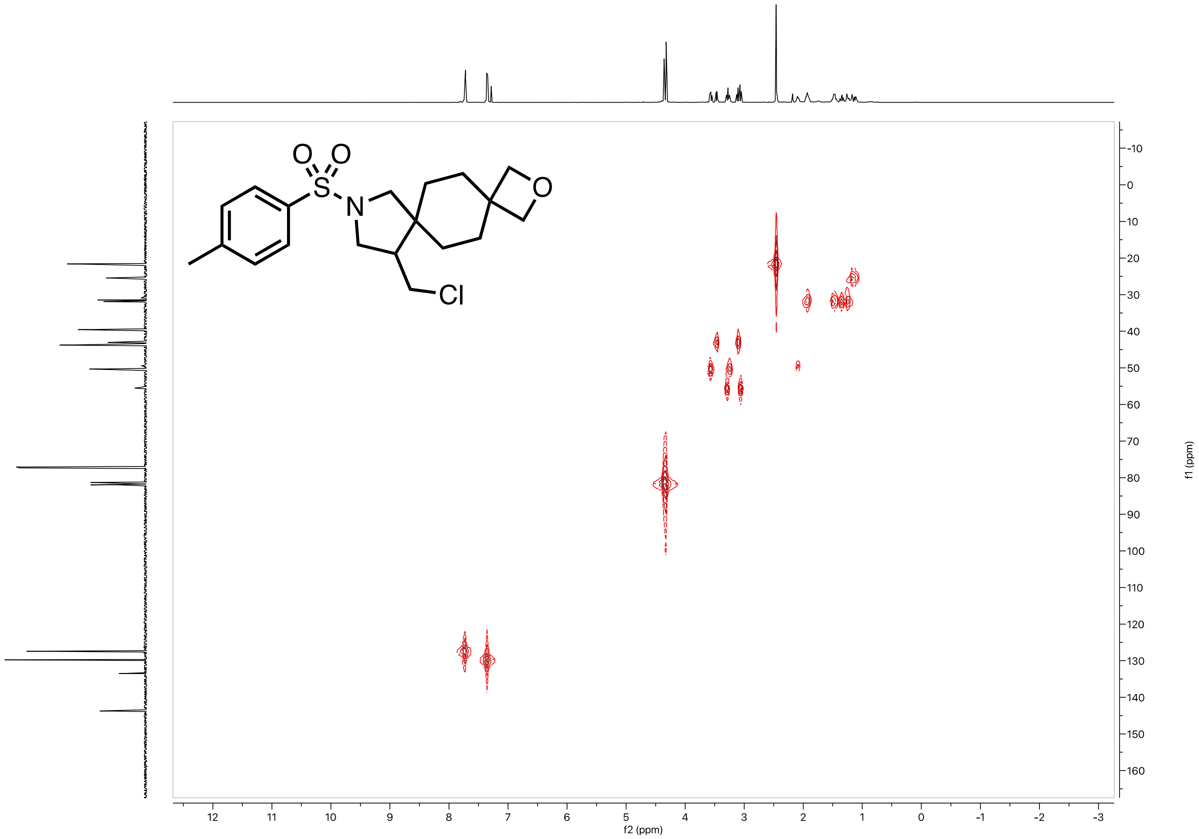

HSQC of *11-(chloromethyl)-9-tosyl-2-oxa-9-azadispiro[3.2.4^7.2^4]tridecane* ($CDCl_3$).

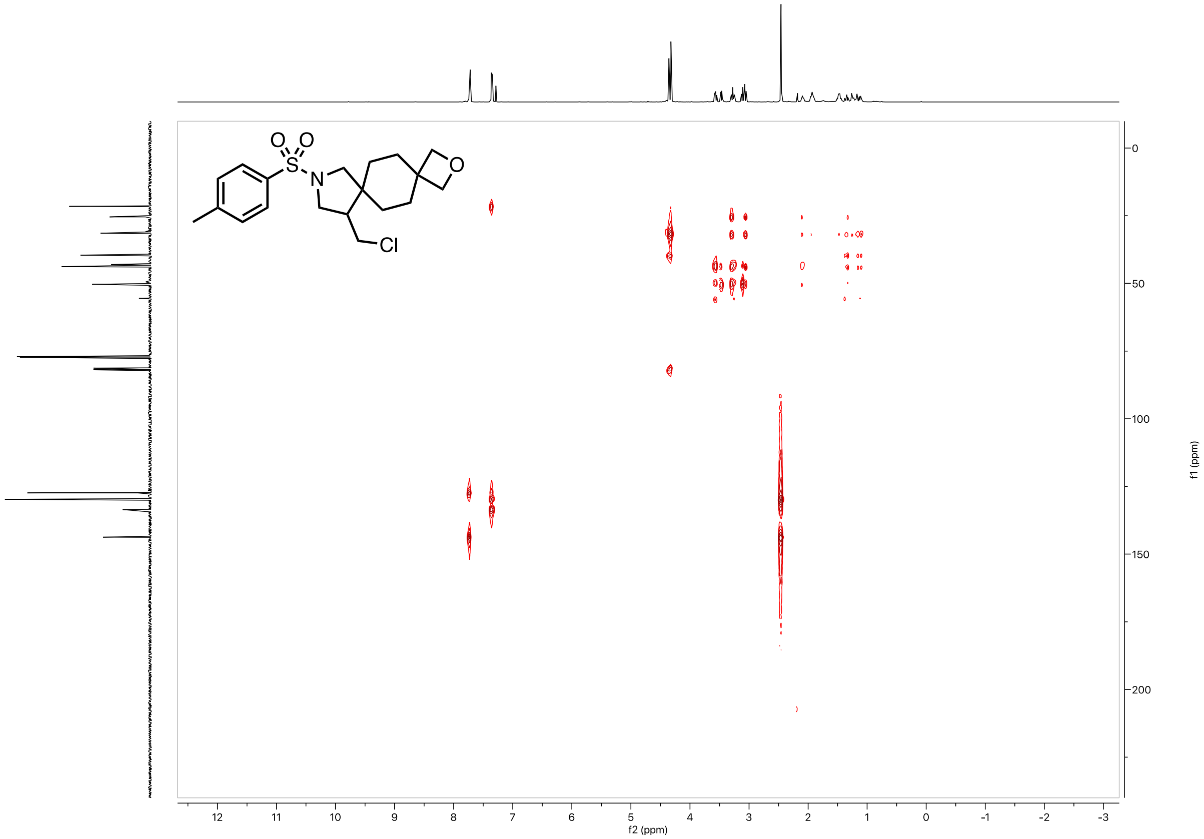

HMBC of *11-(chloromethyl)-9-tosyl-2-oxa-9-azadispiro[3.2.4^{7}.2^{4}]tridecane* ($CDCl_3$).

4-(chloromethyl)-2-tosyl-8-oxa-2-azaspiro[4.5]decane (3x)

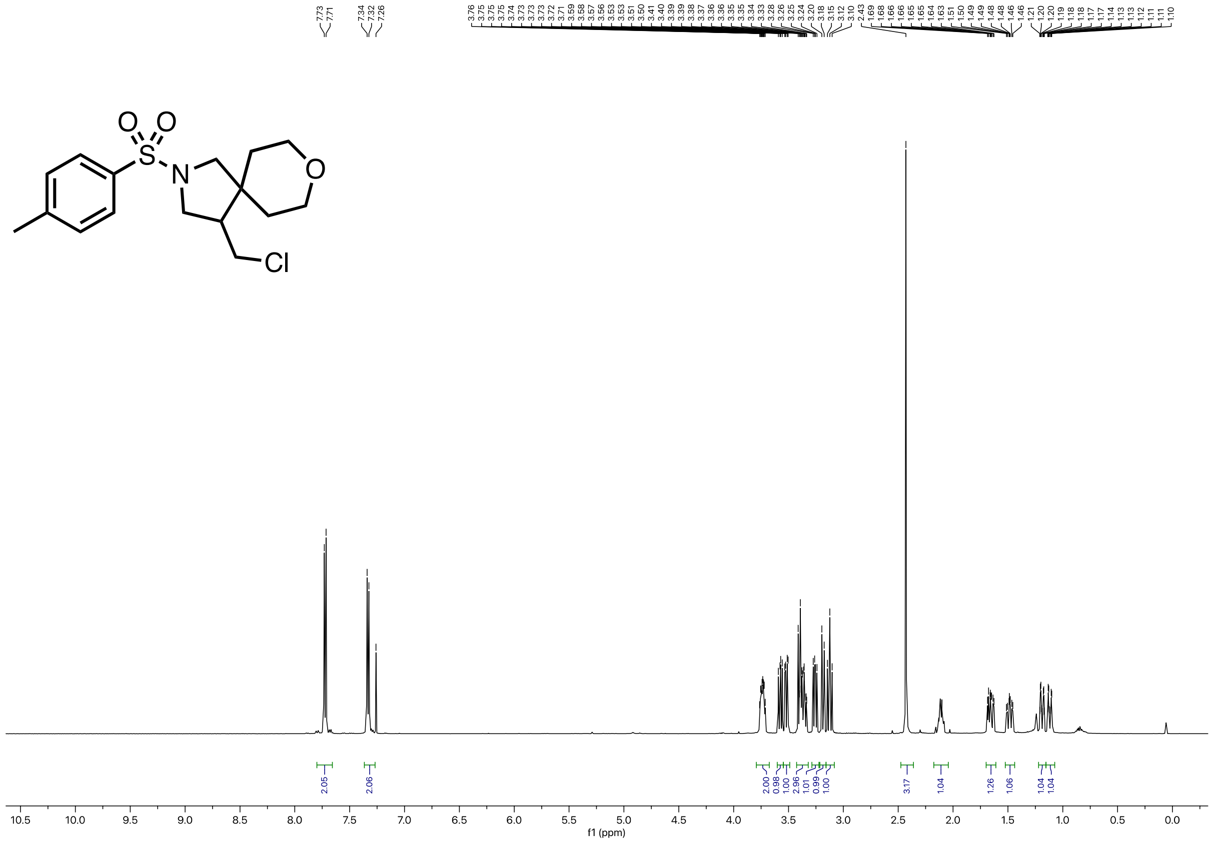

^{1}H NMR of *4-(chloromethyl)-2-tosyl-8-oxa-2-azaspiro[4.5]decane* (400 MHz, $CDCl_3$).

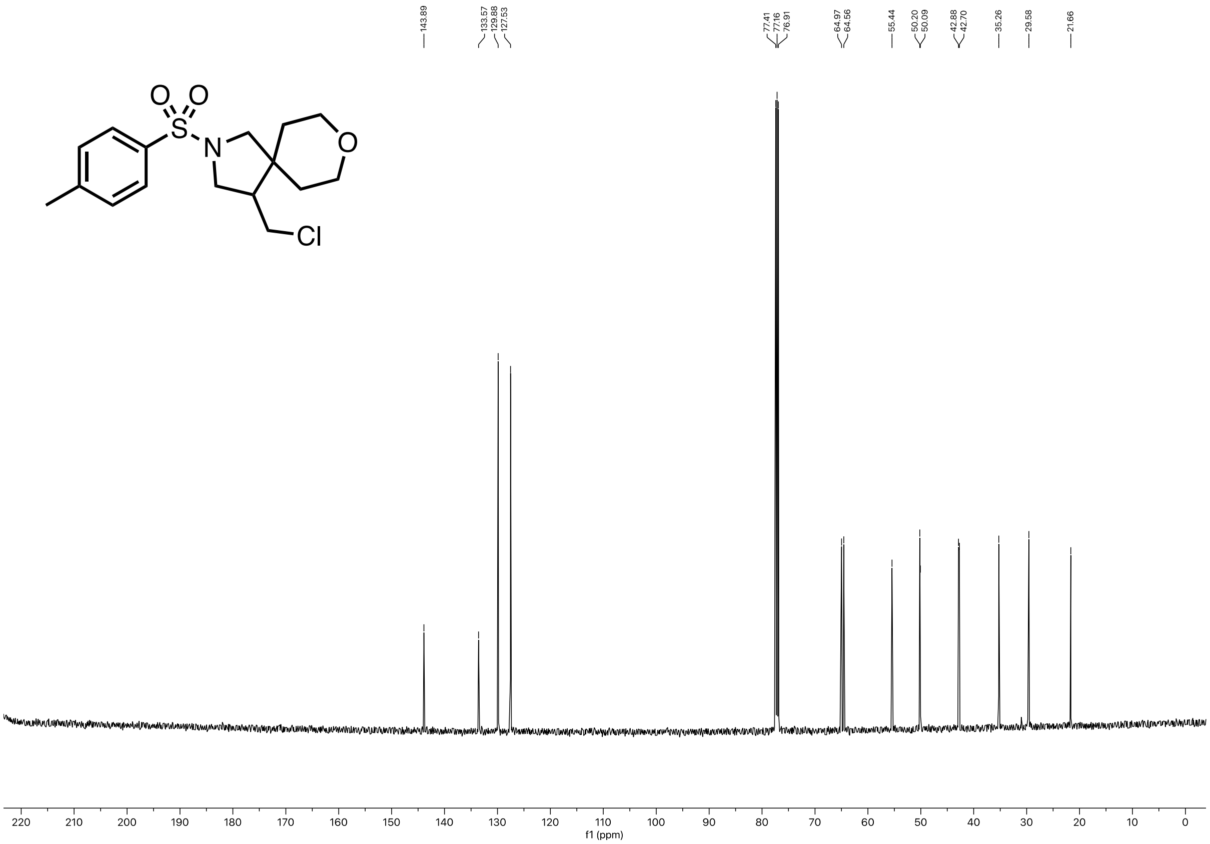

$^{13}C\{^1H\}$ NMR of *4-(chloromethyl)-2-tosyl-8-oxa-2-azaspiro[4.5]decane* (101 MHz, $CDCl_3$).

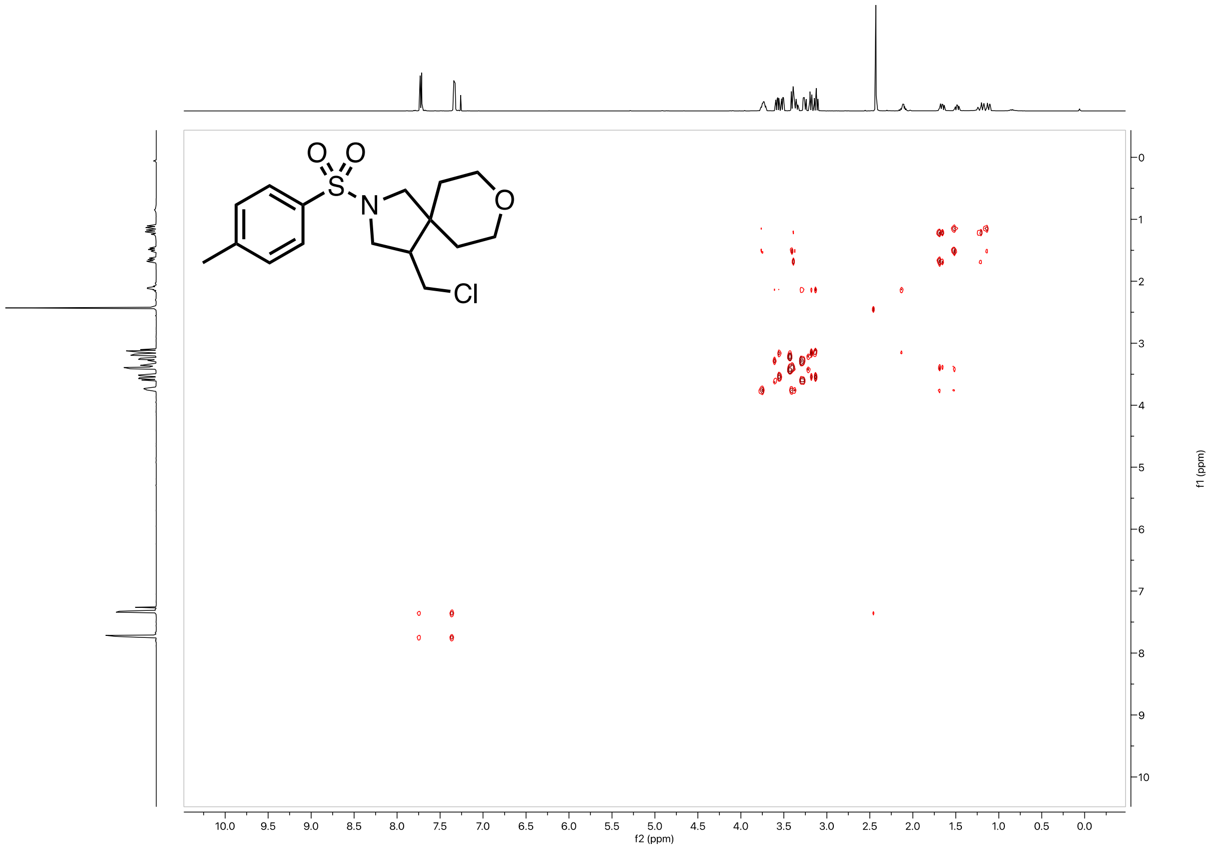

COSY of *4-(chloromethyl)-2-tosyl-8-oxa-2-azaspiro[4.5]decane* ($CDCl_3$).

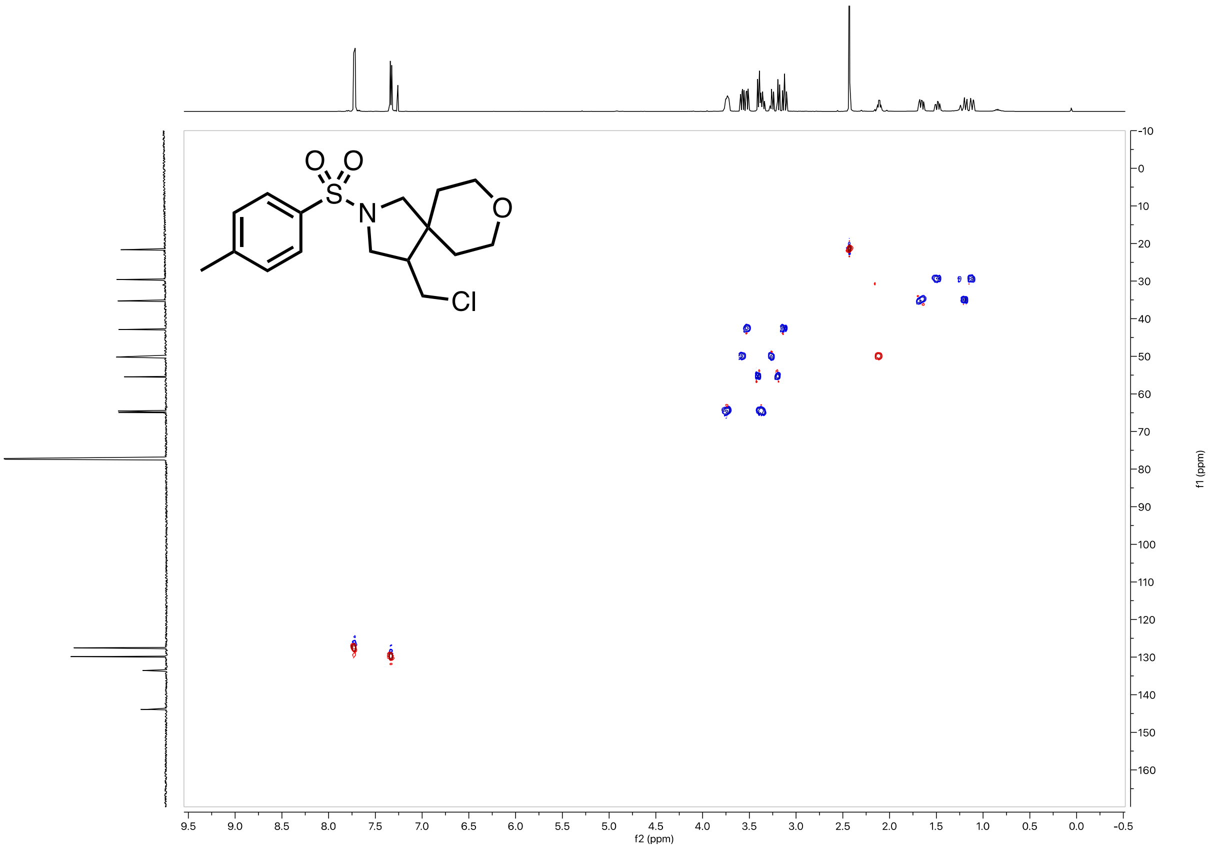

eHSQC of *4-(chloromethyl)-2-tosyl-8-oxa-2-azaspiro[4.5]decane* ($CDCl_3$).

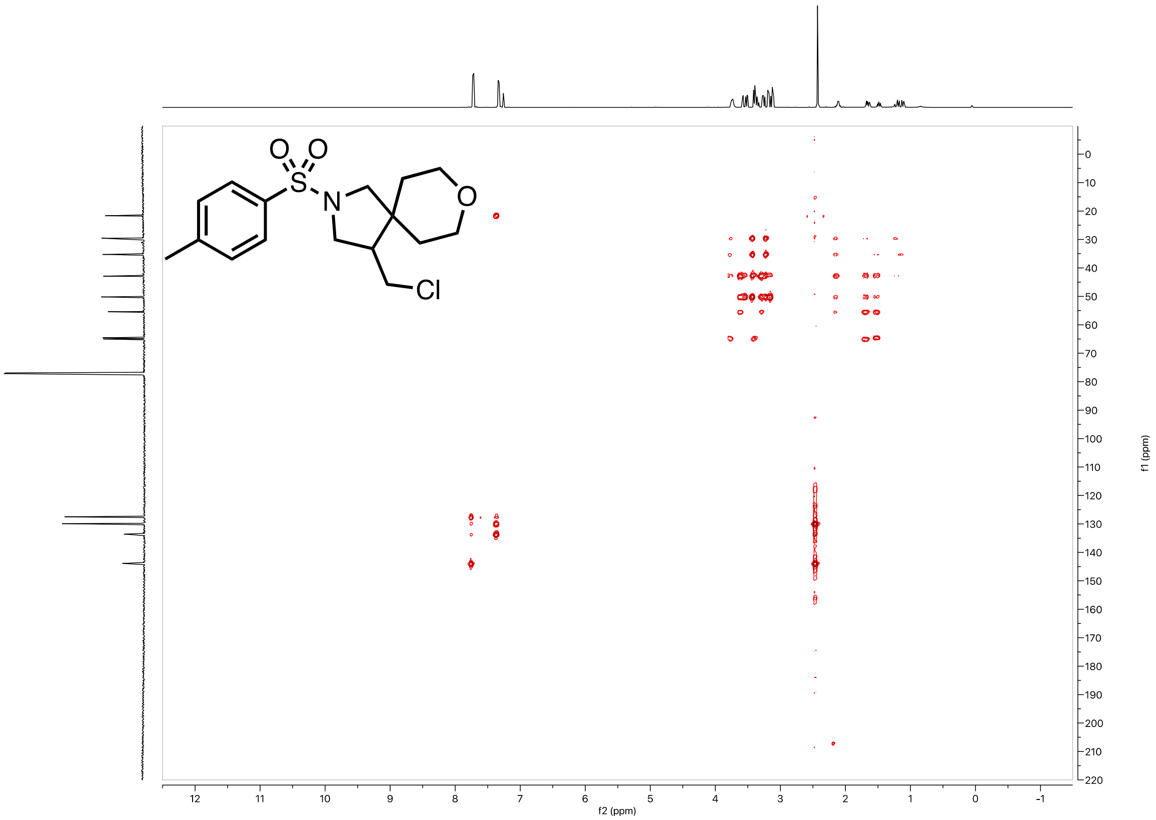

HMBC of *4-(chloromethyl)-2-tosyl-8-oxa-2-azaspiro[4.5]decane* ($CDCl_3$).

(5r,7r)-4''-(chloromethyl)-2'-phenyl-1''-tosyldispiro[adamantane-2,4'-oxazole-5',3''-pyrrolidine] (3y)

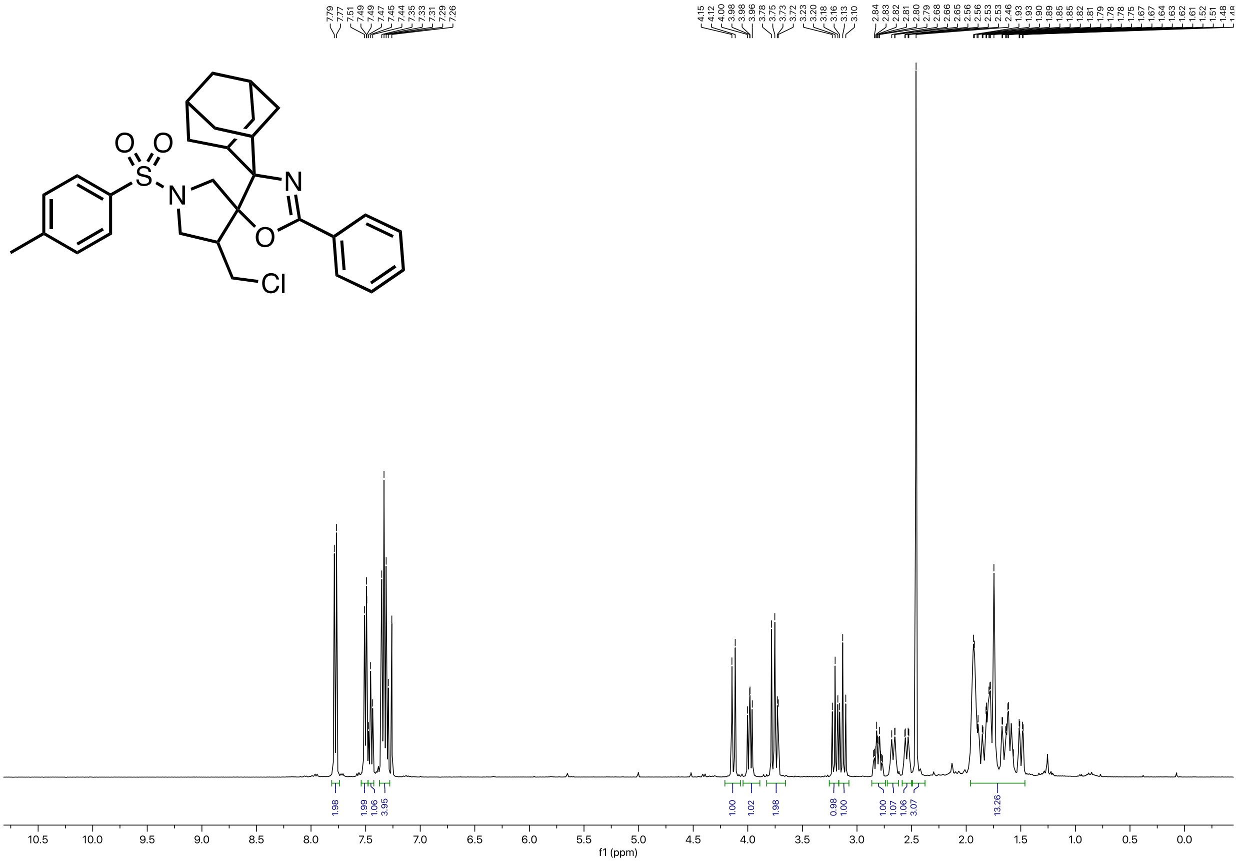

^1H NMR of *(5r,7r)-4''-(chloromethyl)-2'-phenyl-1''-tosyldispiro[adamantane-2,4'-oxazole-5',3''-pyrrolidine]* (400 MHz, $CDCl_3$).

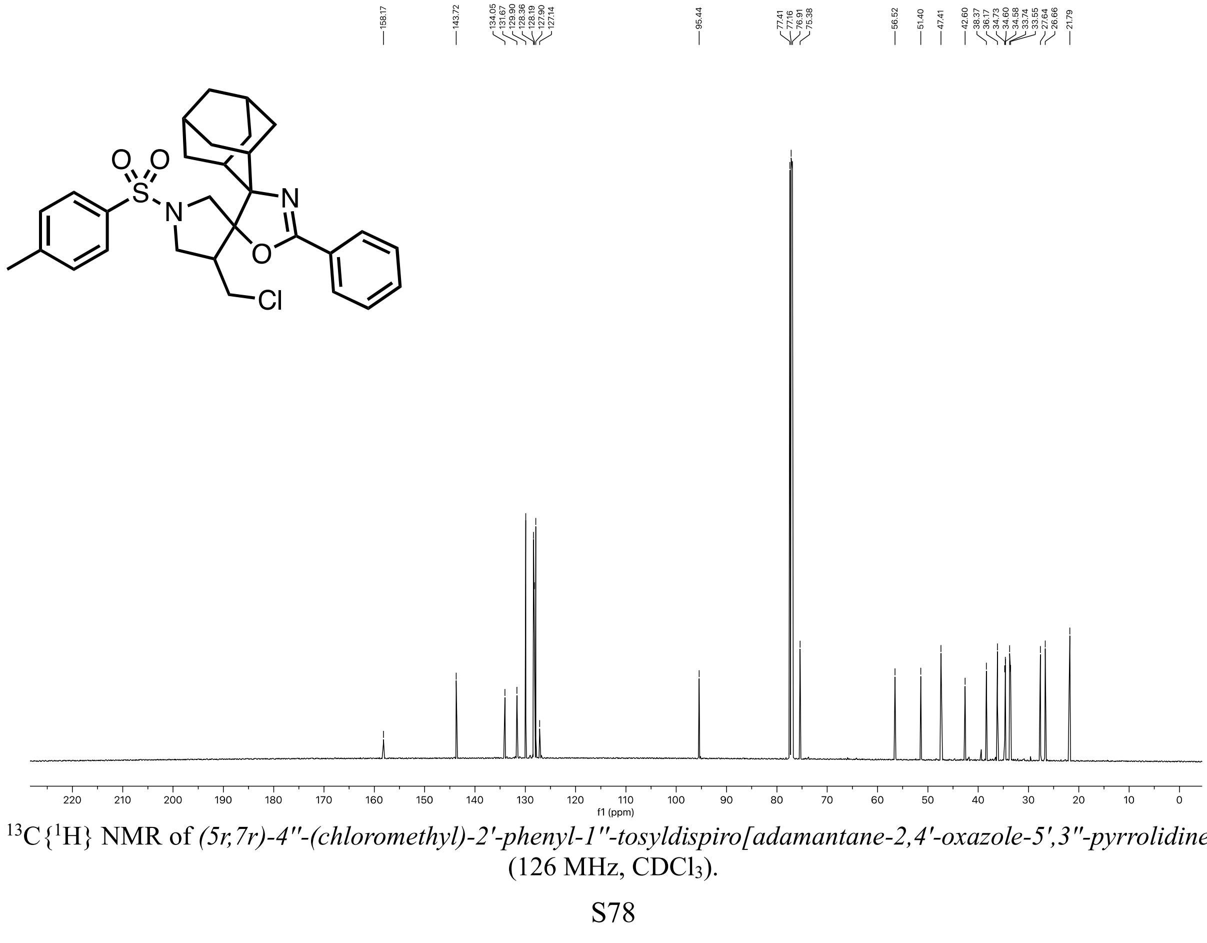

$^{13}C\{^1H\}$ NMR of *(5r,7r)-4''-(chloromethyl)-2'-phenyl-1''-tosyldispiro[adamantane-2,4'-oxazole-5',3''-pyrrolidine]* (126 MHz, $CDCl_3$).

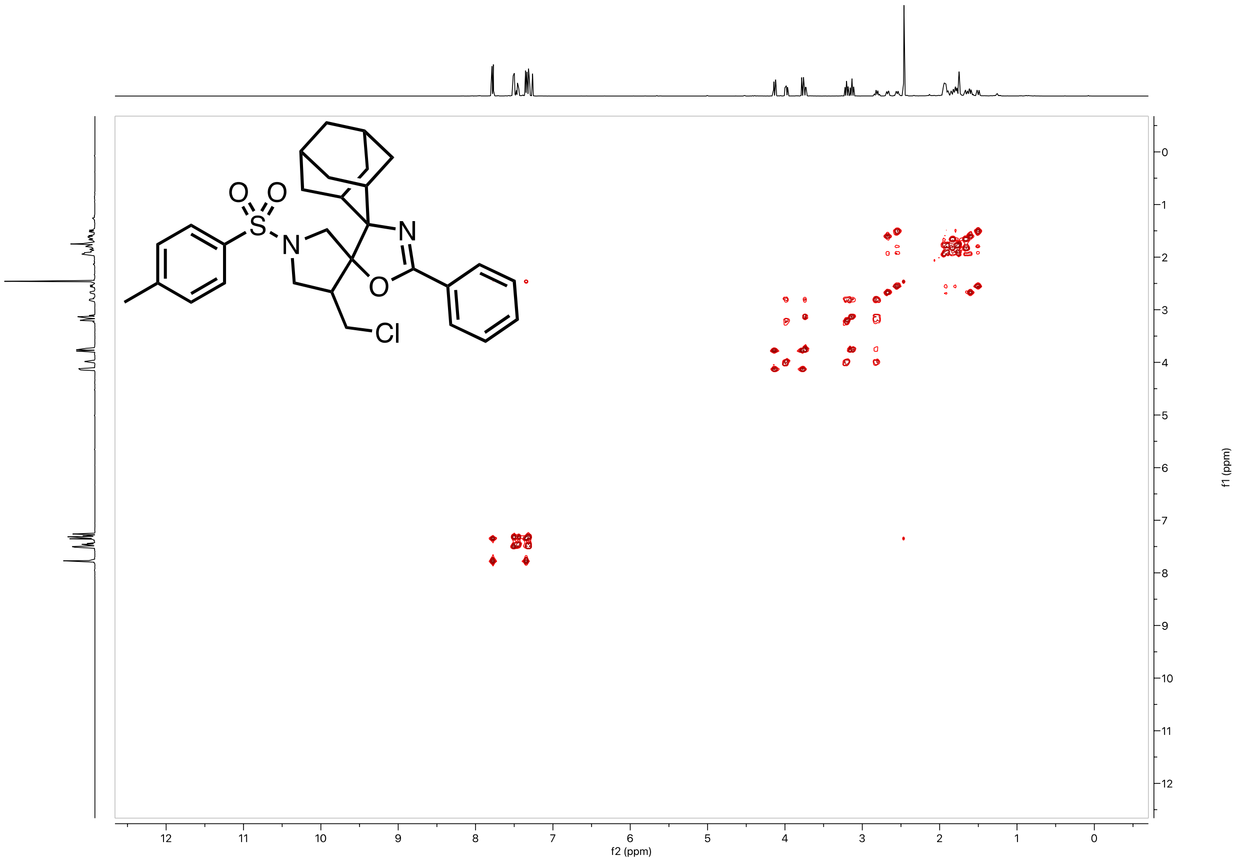

COSY of *(5r,7r)-4''-(chloromethyl)-2'-phenyl-1''-tosyldispiro[adamantane-2,4'-oxazole-5',3''-pyrrolidine* ($CDCl_3$).

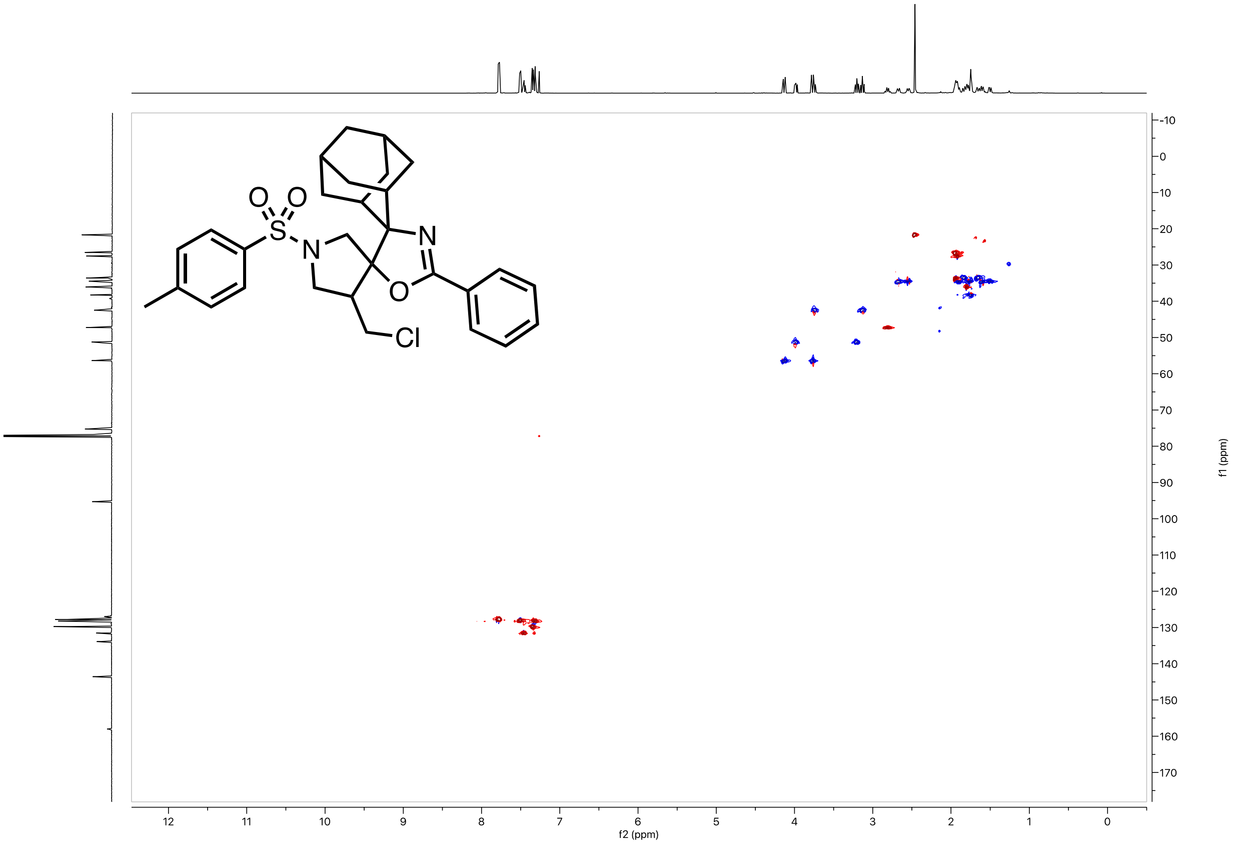

eHSQC of *(5r,7r)-4''-(chloromethyl)-2'-phenyl-1''-tosyldispiro[adamantane-2,4'-oxazole-5',3''-pyrrolidine]* ($CDCl_3$).

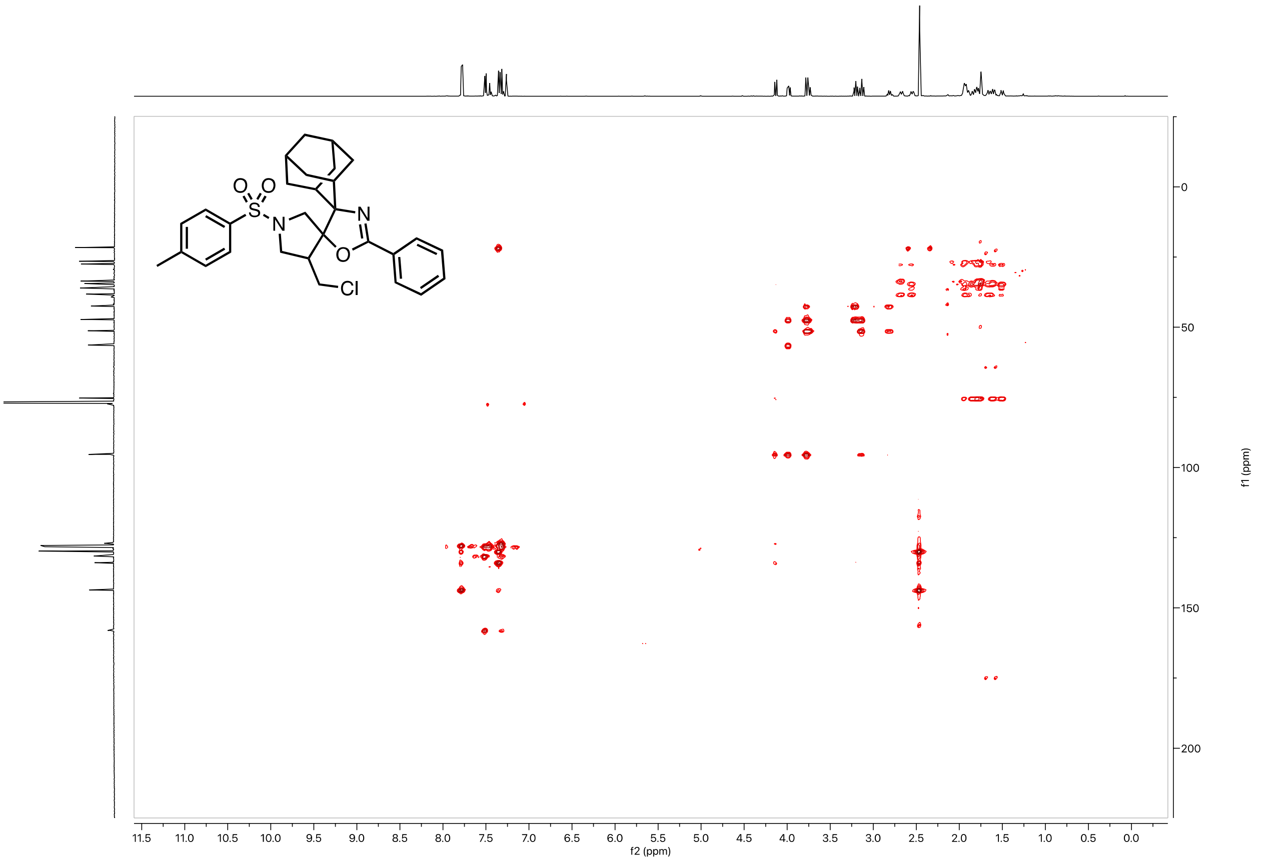

HMBC of *(5r,7r)-4''-(chloromethyl)-2'-phenyl-1''-tosyldispiro[adamantane-2,4'-oxazole-5',3''-pyrrolidine]* ($CDCl_3$).

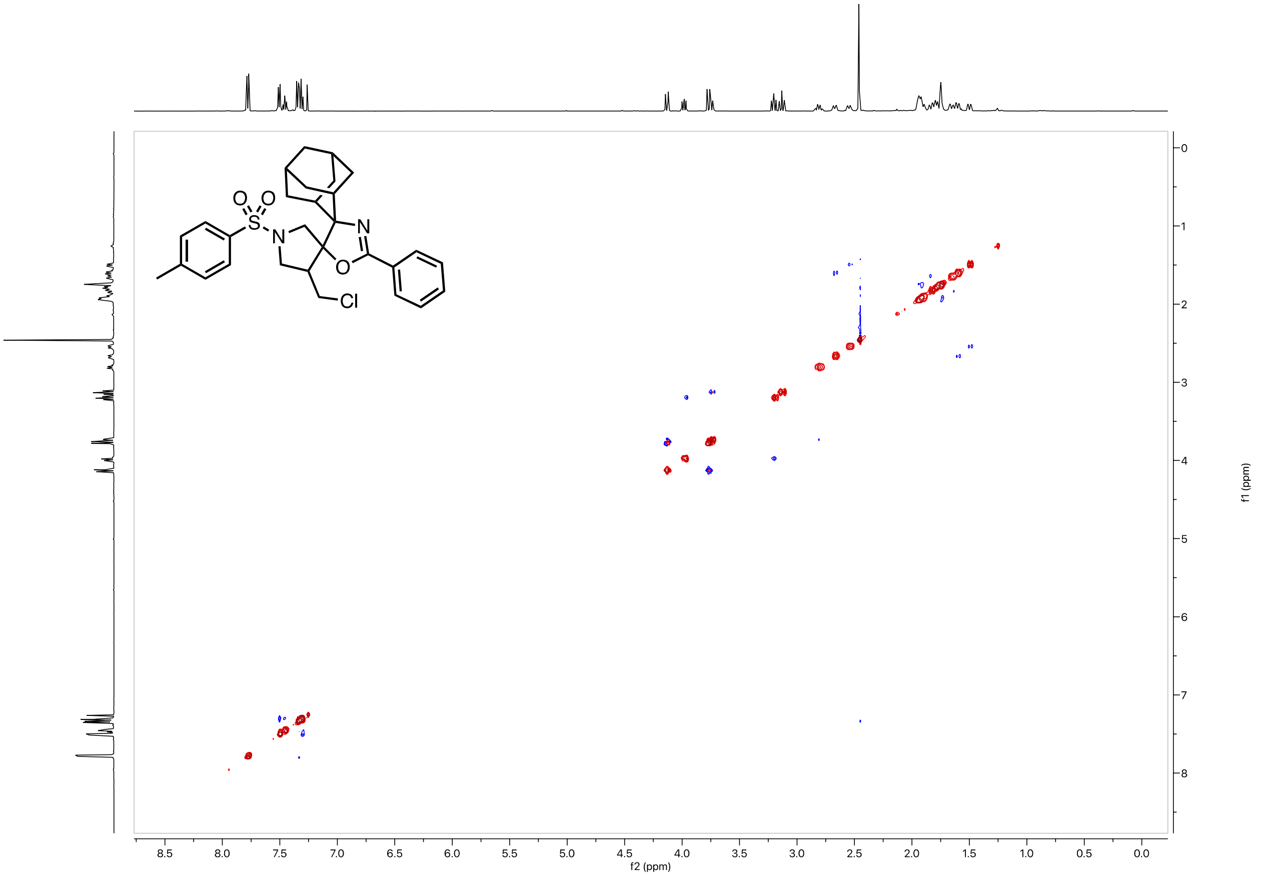

NOESY of *(5r,7r)-4''-(chloromethyl)-2'-phenyl-1''-tosyldispiro[adamantane-2,4'-oxazole-5',3''-pyrrolidine]* ($CDCl_3$).

4-(chloromethyl)-2-tosyl-8-thia-2-azaspiro[4.5]decane 8,8-dioxide (3z)

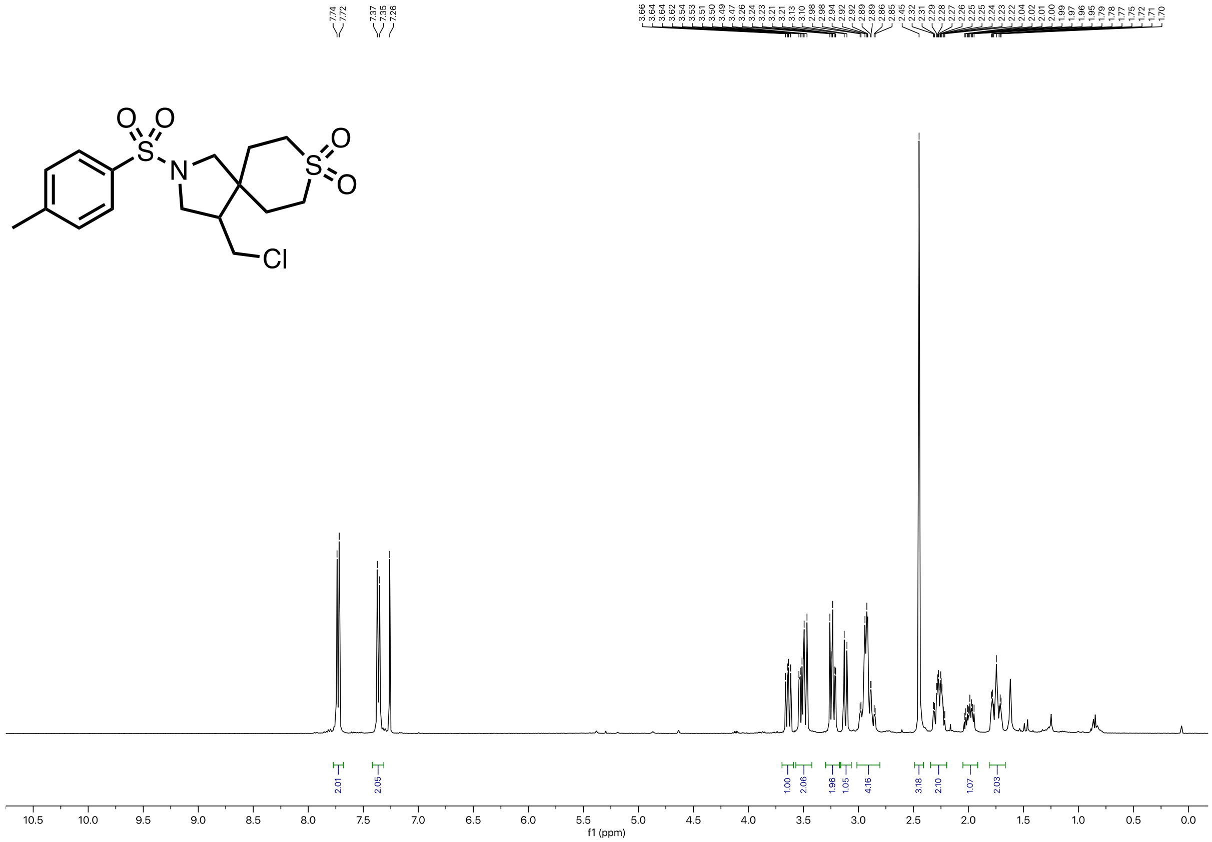

^{1}H NMR of *4-(chloromethyl)-2-tosyl-8-thia-2-azaspiro[4.5]decane 8,8-dioxide* (400 MHz, $CDCl_3$).

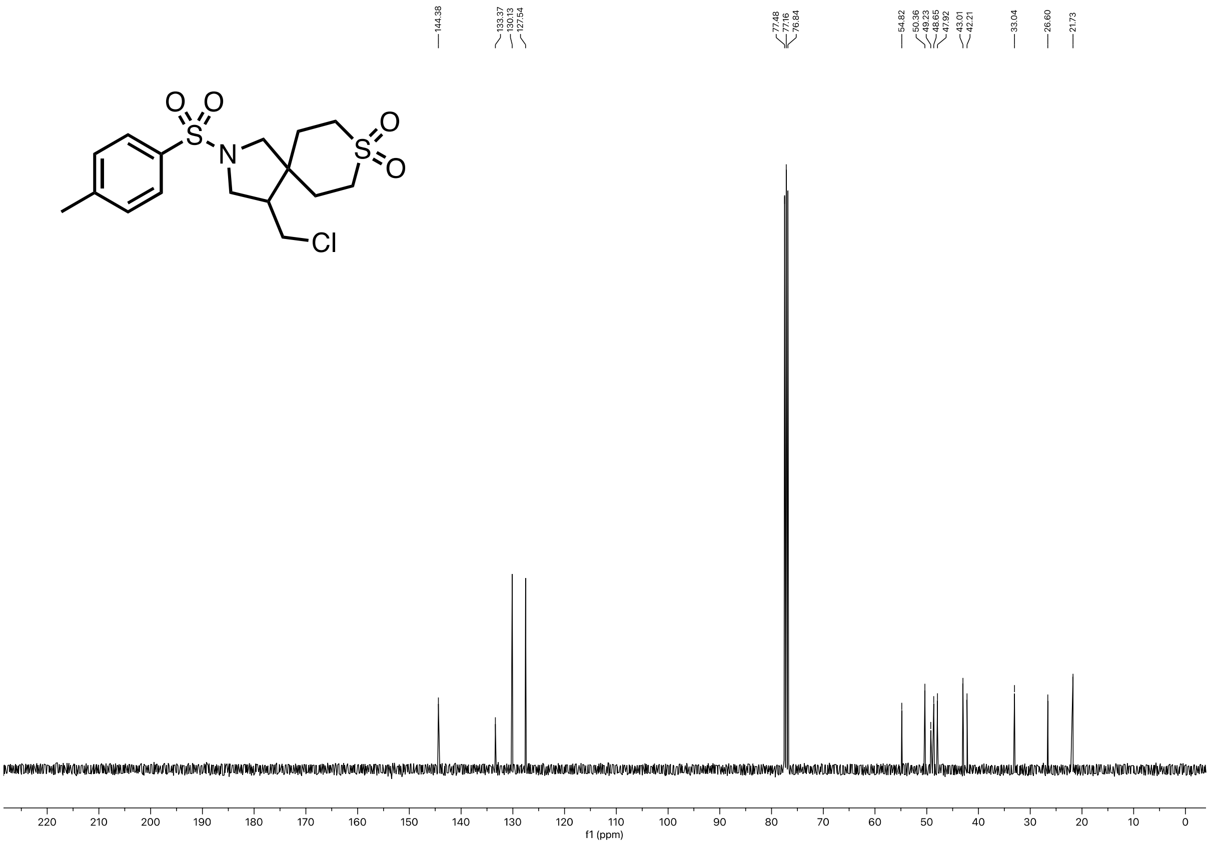

^{13}C{^{1}H} NMR of *4-(chloromethyl)-2-tosyl-8-thia-2-azaspiro[4.5]decane 8,8-dioxide* (101 MHz, $CDCl_3$).

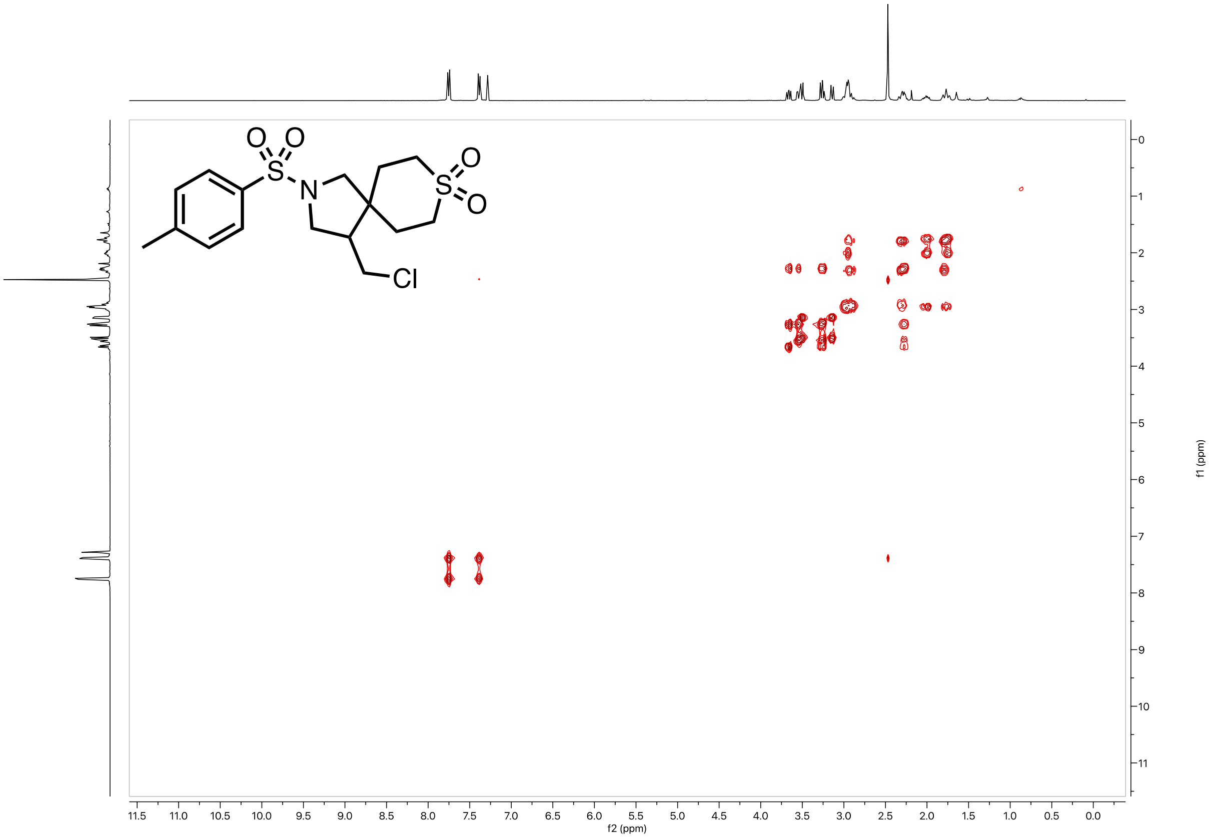

COSY of *4-(chloromethyl)-2-tosyl-8-thia-2-azaspiro[4.5]decane 8,8-dioxide* ($CDCl_3$).

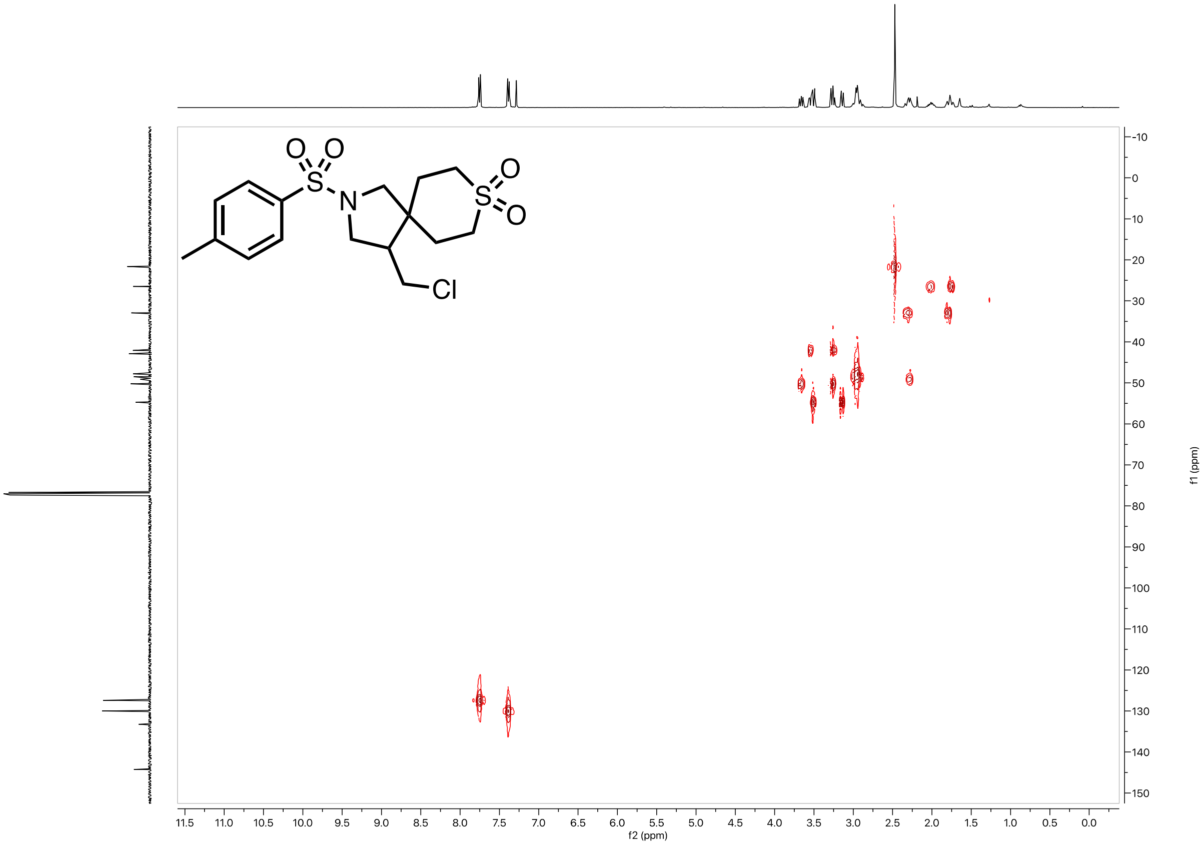

HSQC of *4-(chloromethyl)-2-tosyl-8-thia-2-azaspiro[4.5]decane 8,8-dioxide* ($CDCl_3$).

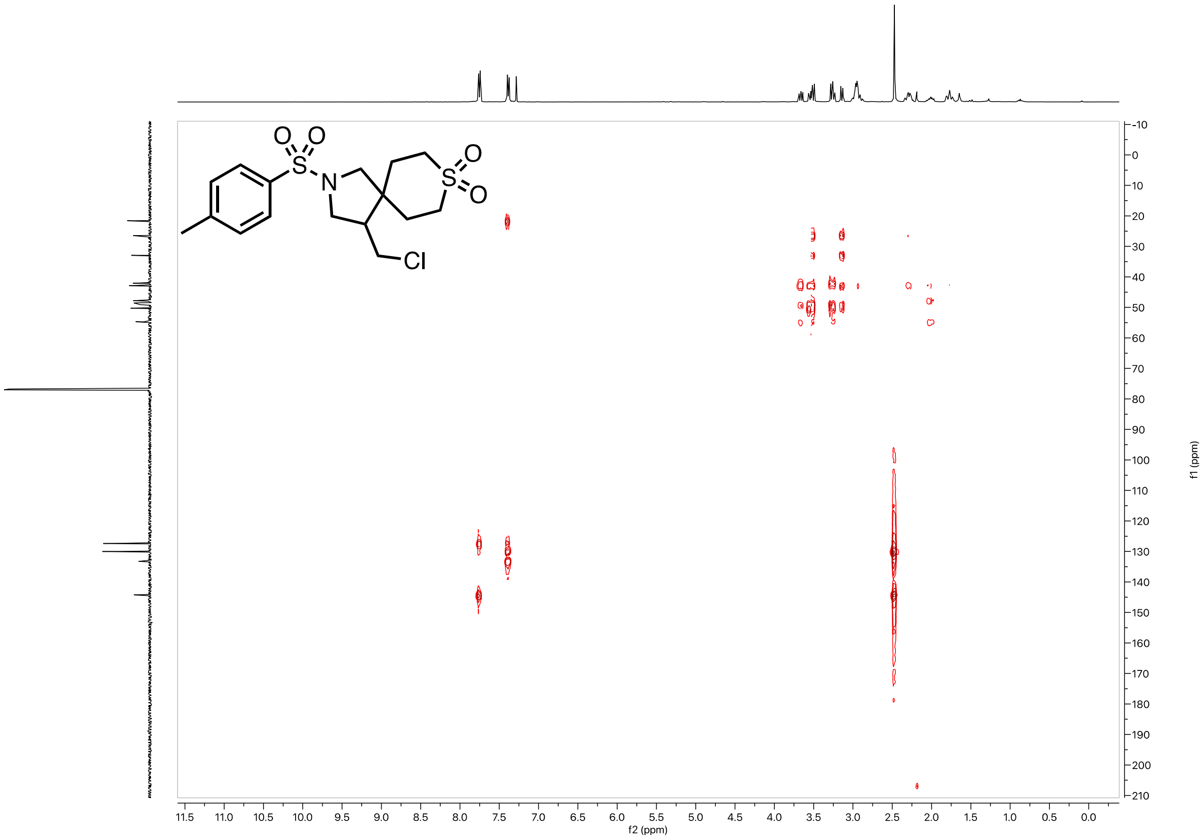

HMBC of *4-(chloromethyl)-2-tosyl-8-thia-2-azaspiro[4.5]decane 8,8-dioxide* ($CDCl_3$).

8-(bromomethyl)-6-((4-nitrophenyl)sulfonyl)-2-tosyl-2,6-diazaspiro[3.4]octane (3aa)

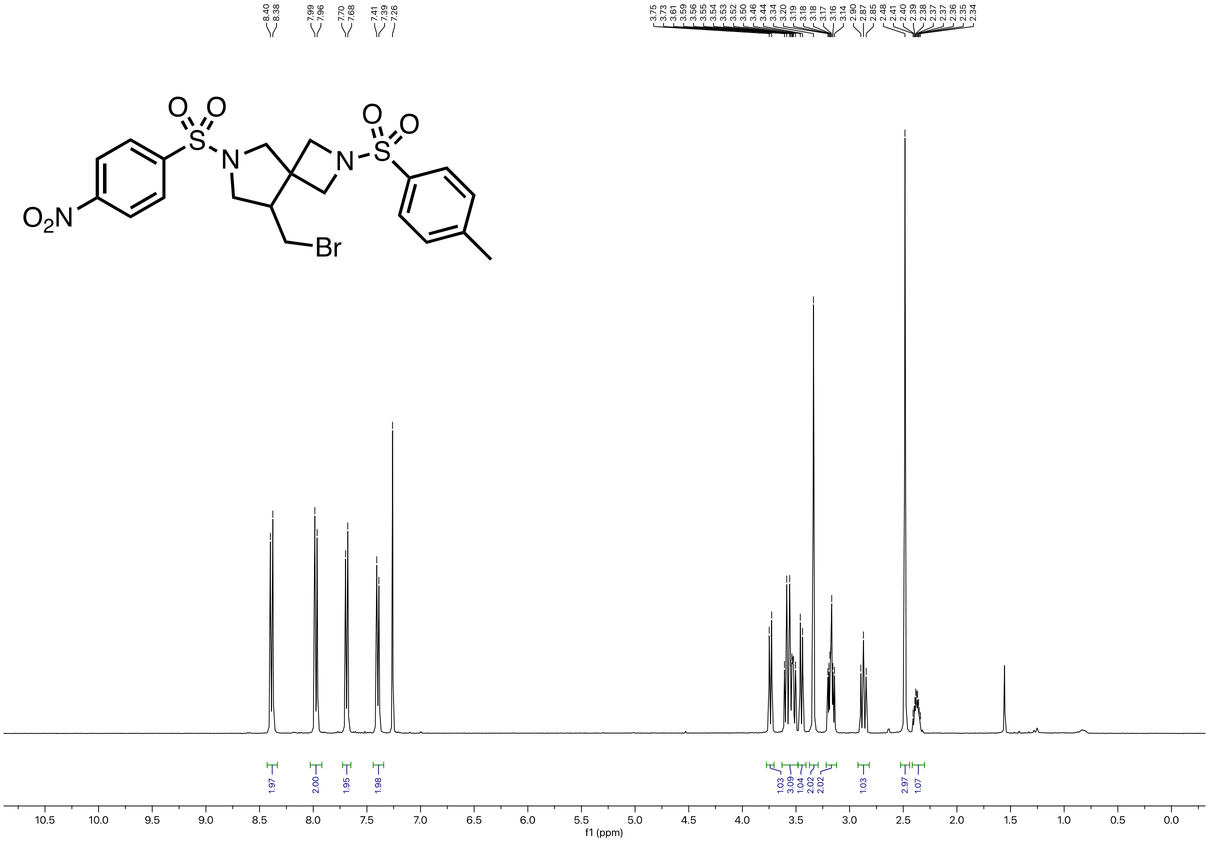

^{1}H NMR of *8-(bromomethyl)-6-((4-nitrophenyl)sulfonyl)-2-tosyl-2,6-diazaspiro[3.4]octane* (400 MHz, $CDCl_3$).

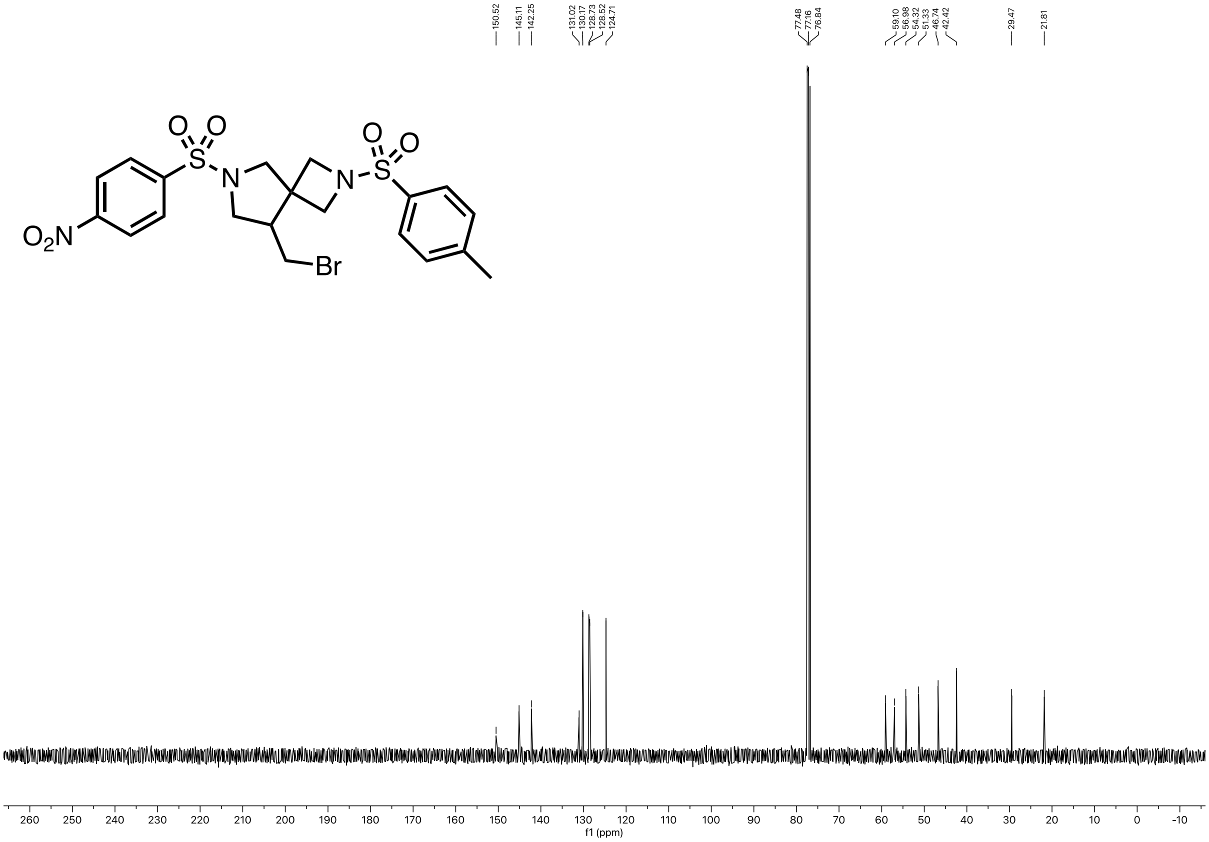

$^{13}C\{^{1}H\}$ NMR of *8-(bromomethyl)-6-((4-nitrophenyl)sulfonyl)-2-tosyl-2,6-diazaspiro[3.4]octane* (101 MHz, $CDCl_3$).

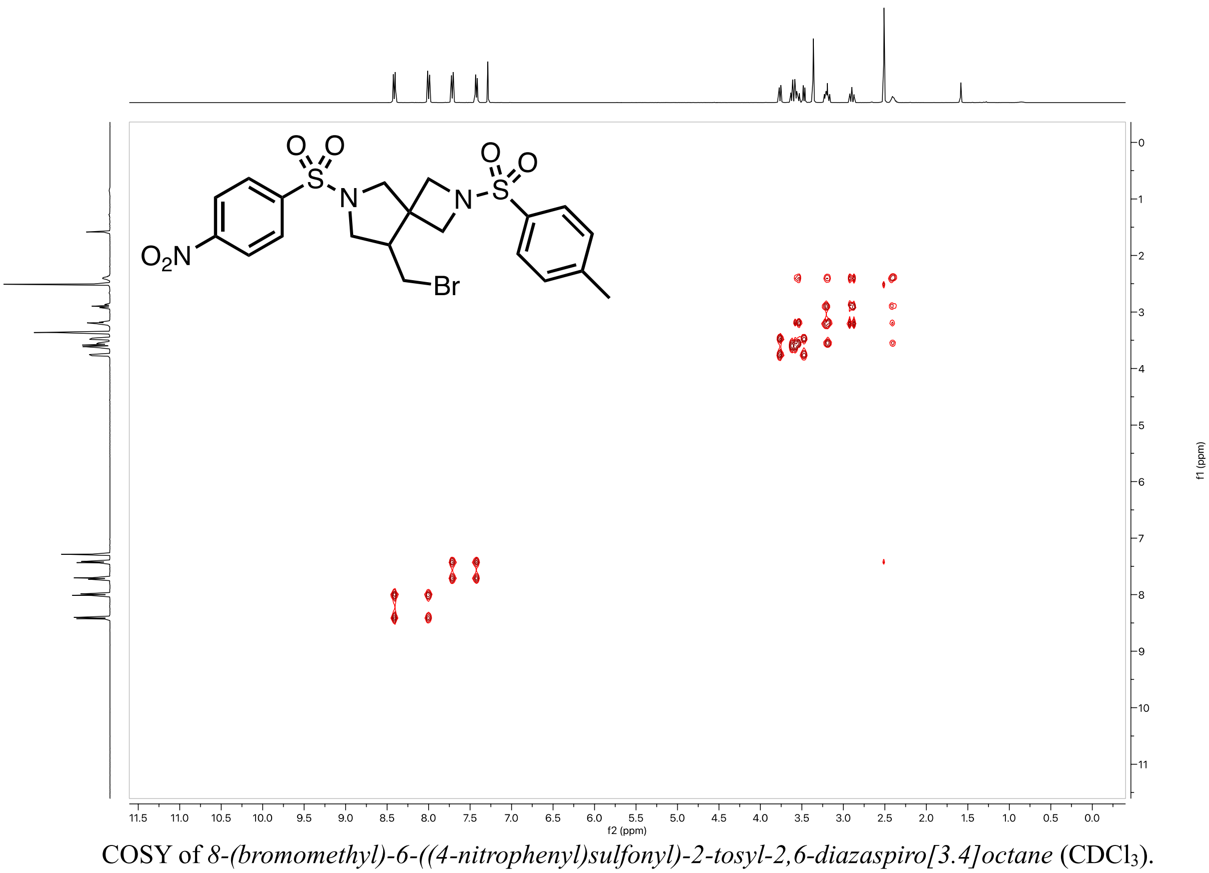

COSY of *8-(bromomethyl)-6-((4-nitrophenyl)sulfonyl)-2-tosyl-2,6-diazaspiro[3.4]octane* ($CDCl_3$).

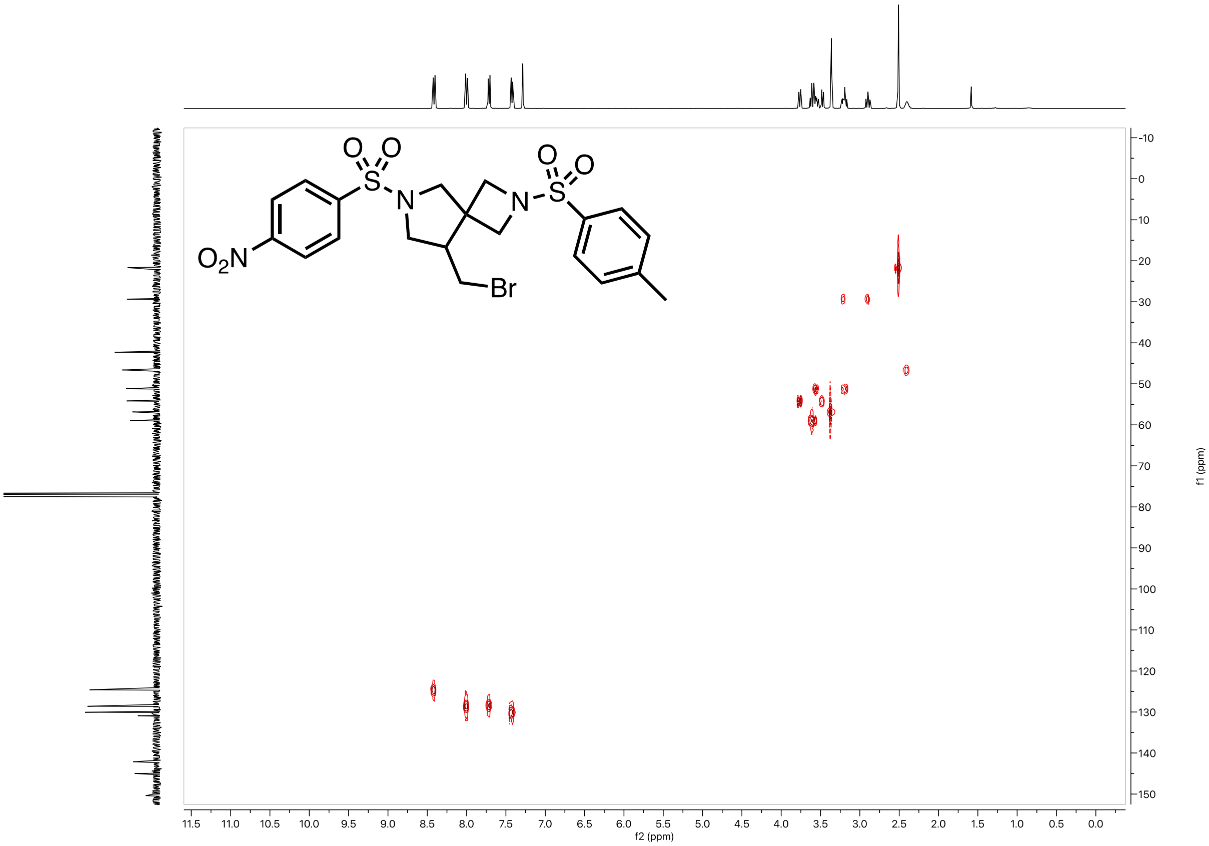

HSQC of *8-(bromomethyl)-6-((4-nitrophenyl)sulfonyl)-2-tosyl-2,6-diazaspiro[3.4]octane* ($CDCl_3$).

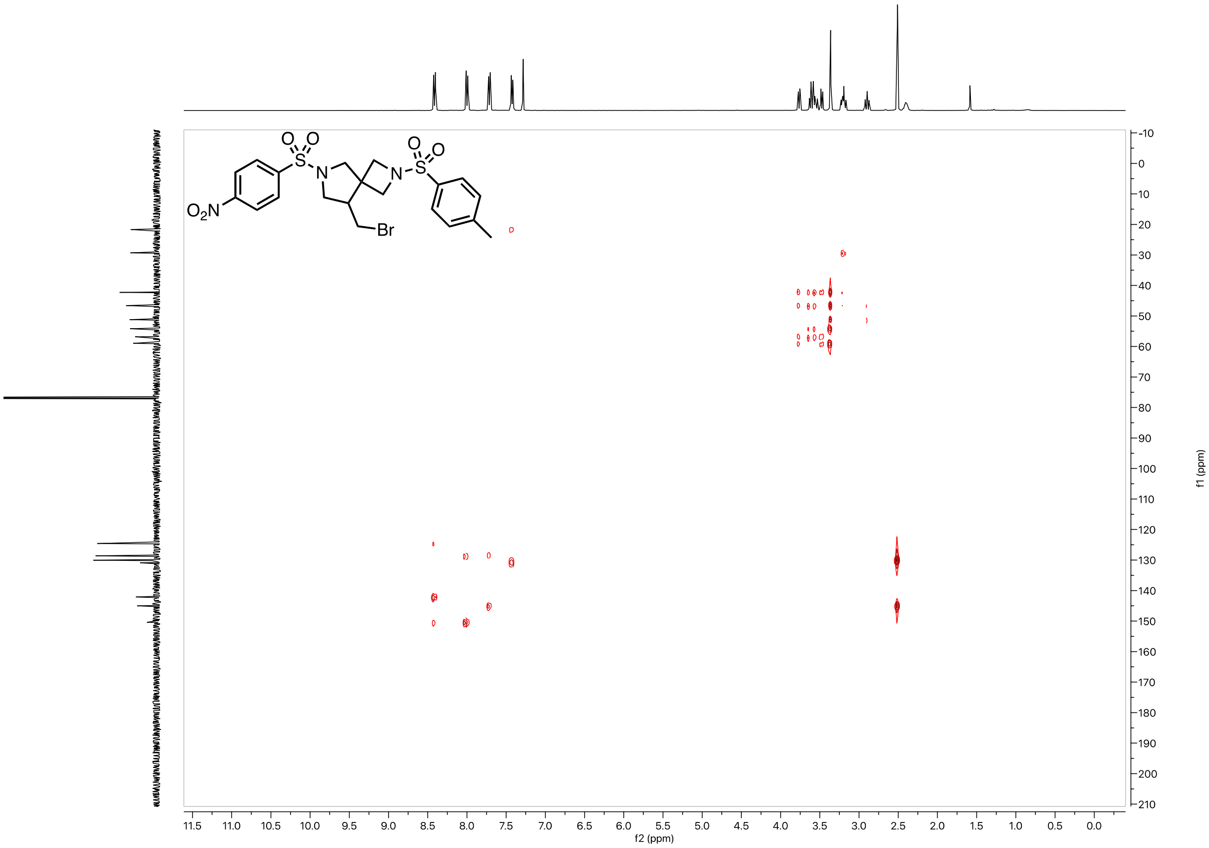

HMBC of *8-(bromomethyl)-6-((4-nitrophenyl)sulfonyl)-2-tosyl-2,6-diazaspiro[3.4]octane* ($CDCl_3$).

4-(chloromethyl)-7-((4-nitrophenyl)sulfonyl)-2-tosyl-2,7-diazaspiro[4.4]nonane (3ab)

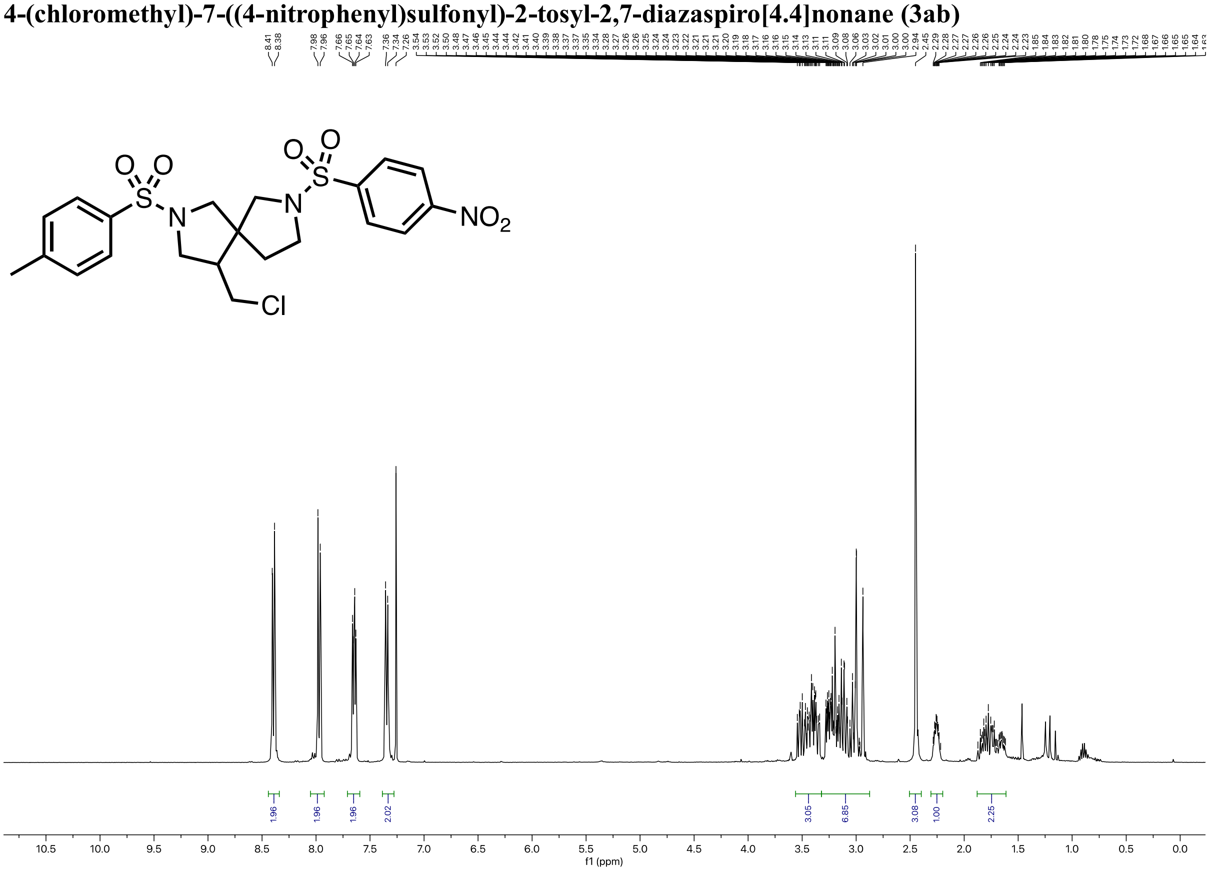

^{1}H NMR of *4-(chloromethyl)-7-((4-nitrophenyl)sulfonyl)-2-tosyl-2,7-diazaspiro[4.4]nonane* (400 MHz, $CDCl_3$).

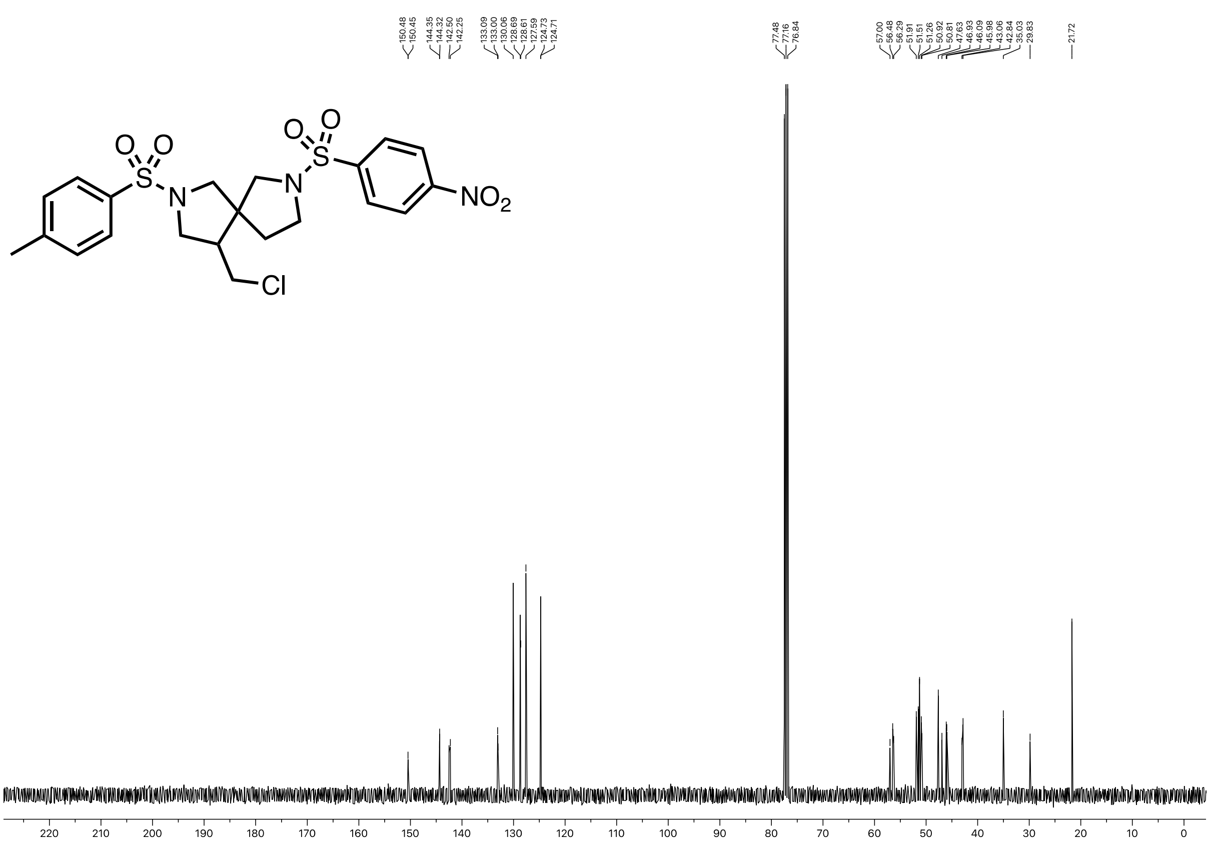

$^{13}C\{^{1}H\}$ NMR of *4-(chloromethyl)-7-((4-nitrophenyl)sulfonyl)-2-tosyl-2,7-diazaspiro[4.4]nonane* (101 MHz, $CDCl_3$).

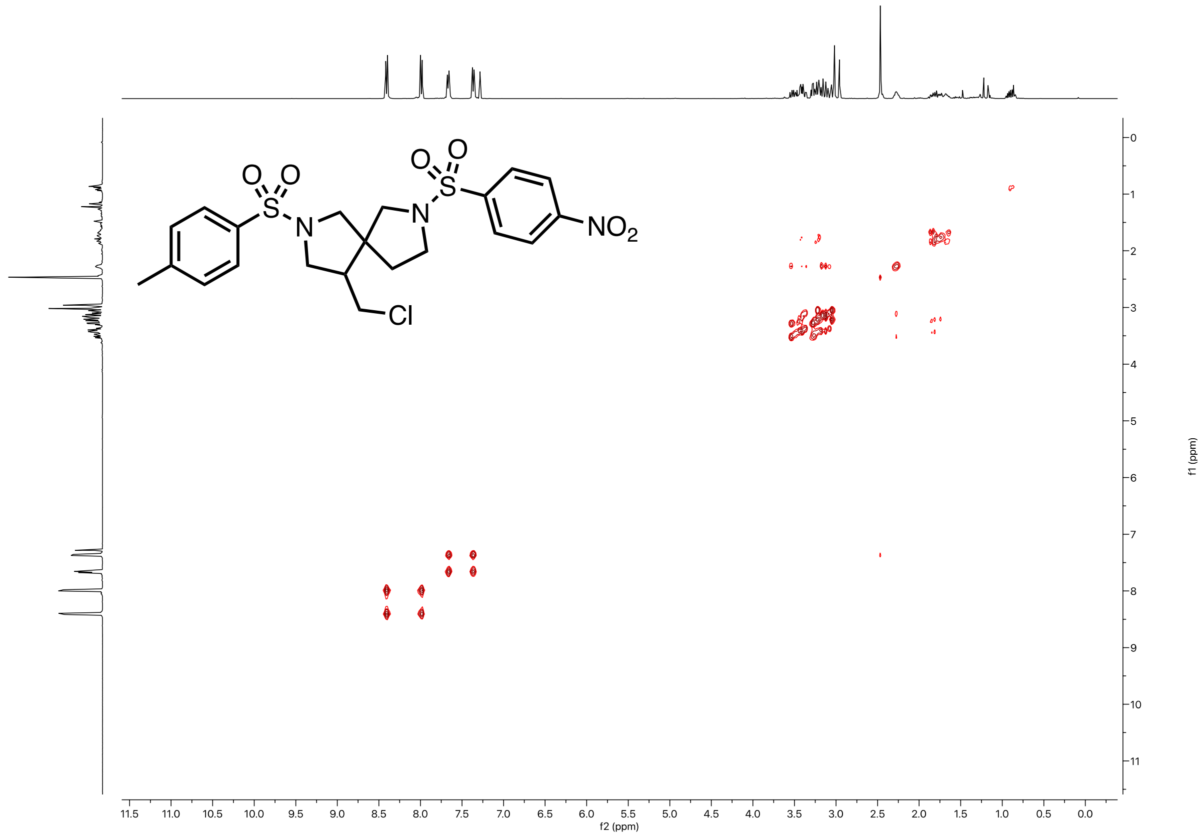

COSY of *4-(chloromethyl)-7-((4-nitrophenyl)sulfonyl)-2-tosyl-2,7-diazaspiro[4.4]nonane* ($CDCl_3$).

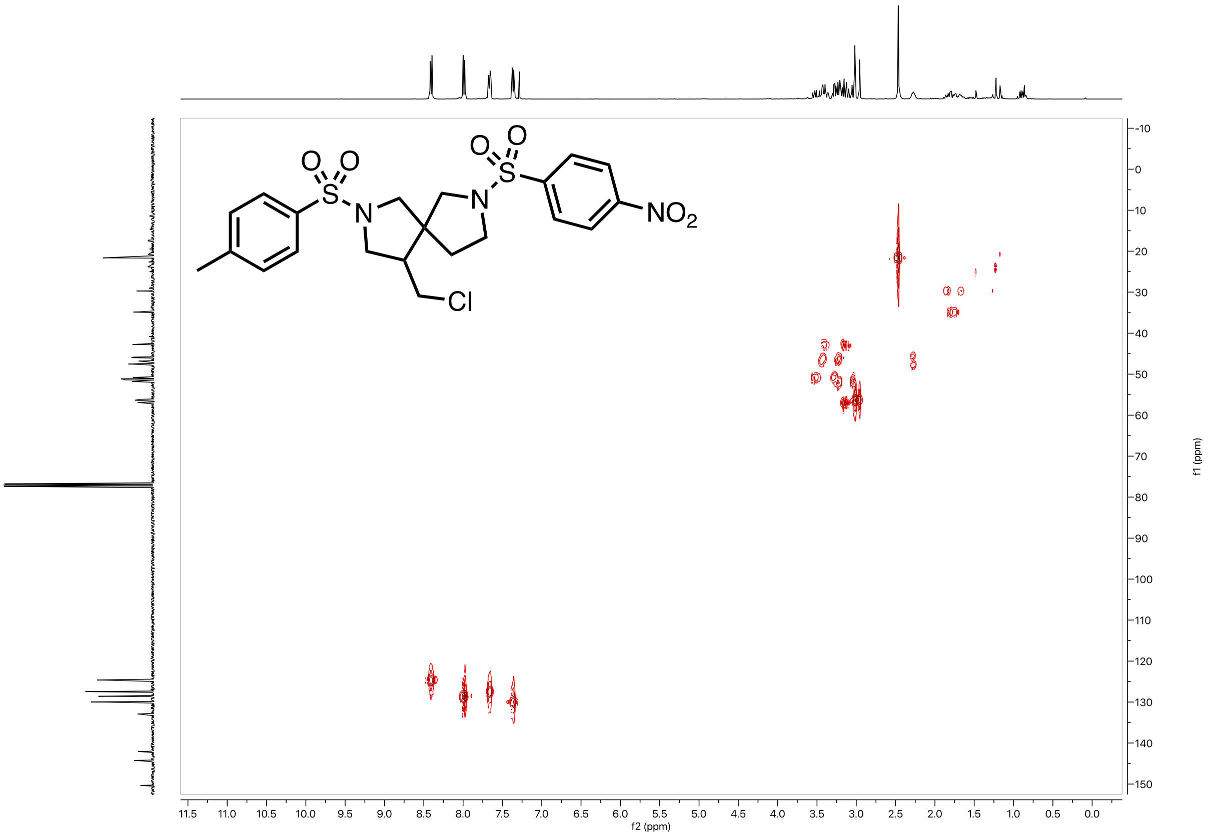

HSQC of *4-(chloromethyl)-7-((4-nitrophenyl)sulfonyl)-2-tosyl-2,7-diazaspiro[4.4]nonane* ($CDCl_3$).

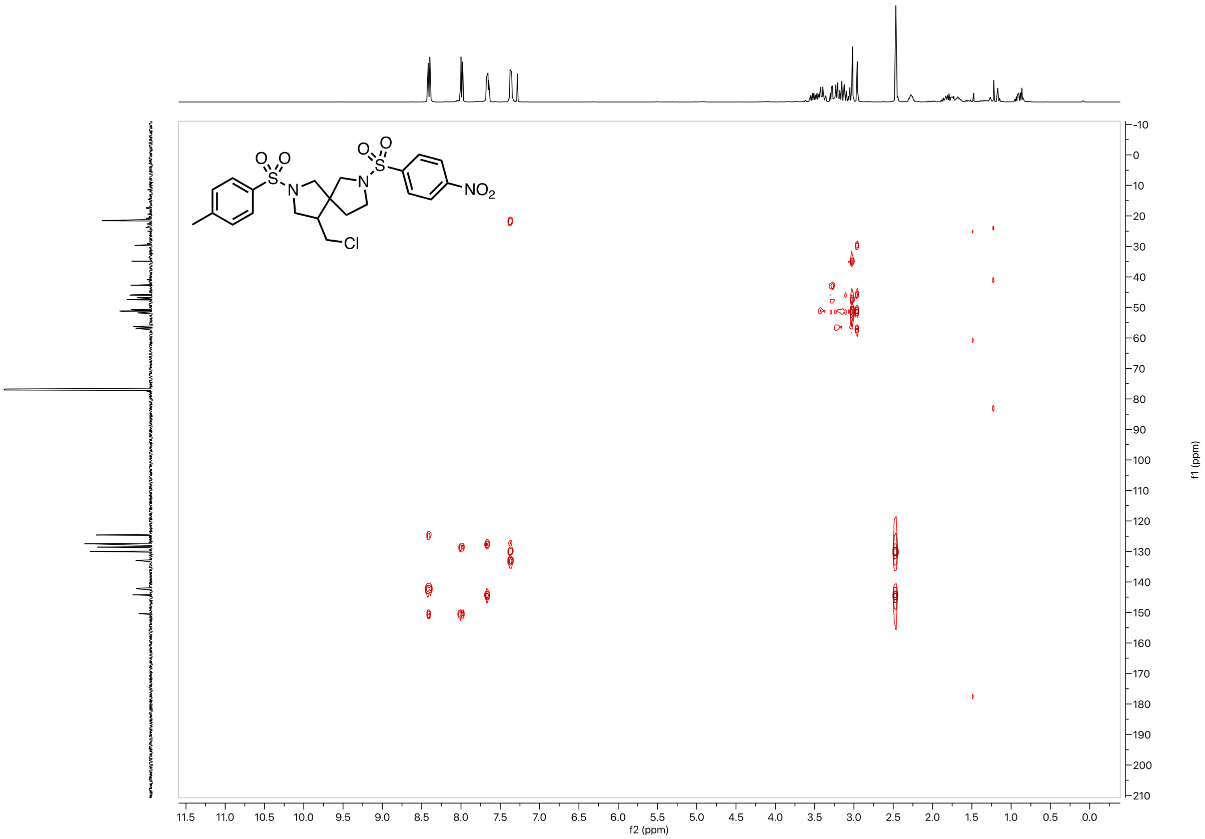

HMBC of *4-(chloromethyl)-7-((4-nitrophenyl)sulfonyl)-2-tosyl-2,7-diazaspiro[4.4]nonane* ($CDCl_3$).

4-(chloromethyl)-2,8-ditosyl-2,8-diazaspiro[4.5]decane (3ac)

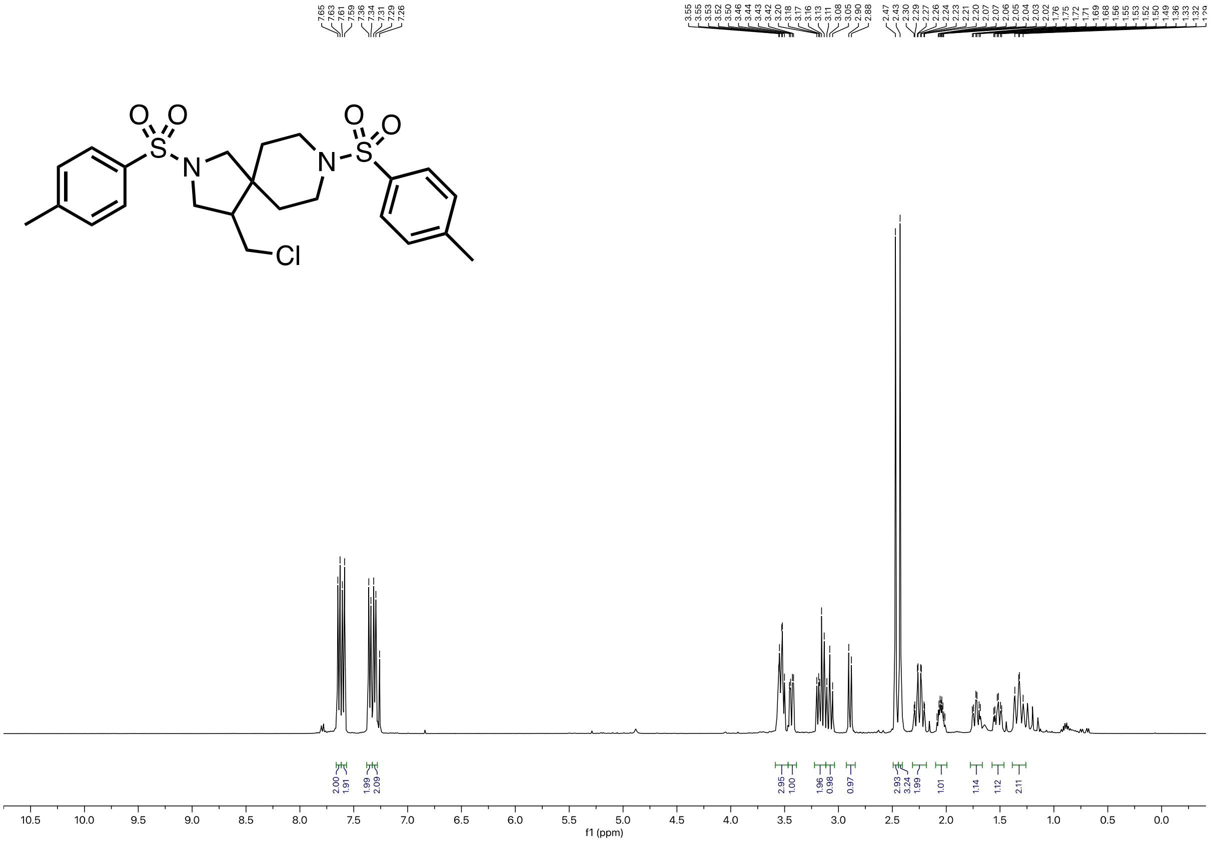

^{1}H NMR of *4-(chloromethyl)-2,8-ditosyl-2,8-diazaspiro[4.5]decane* (400 MHz, $CDCl_3$).

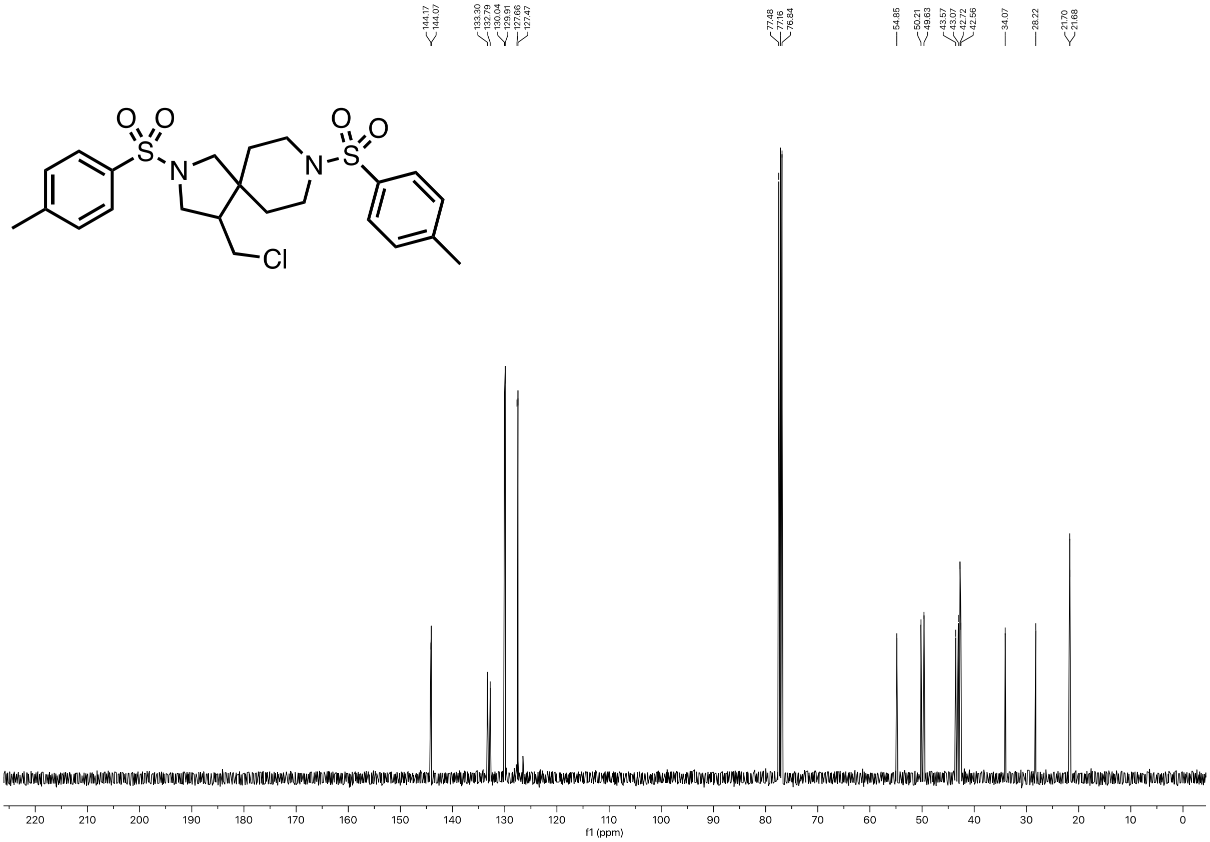

$^{13}C\{^{1}H\}$ NMR of *4-(chloromethyl)-2,8-ditosyl-2,8-diazaspiro[4.5]decane* (101 MHz, $CDCl_3$).

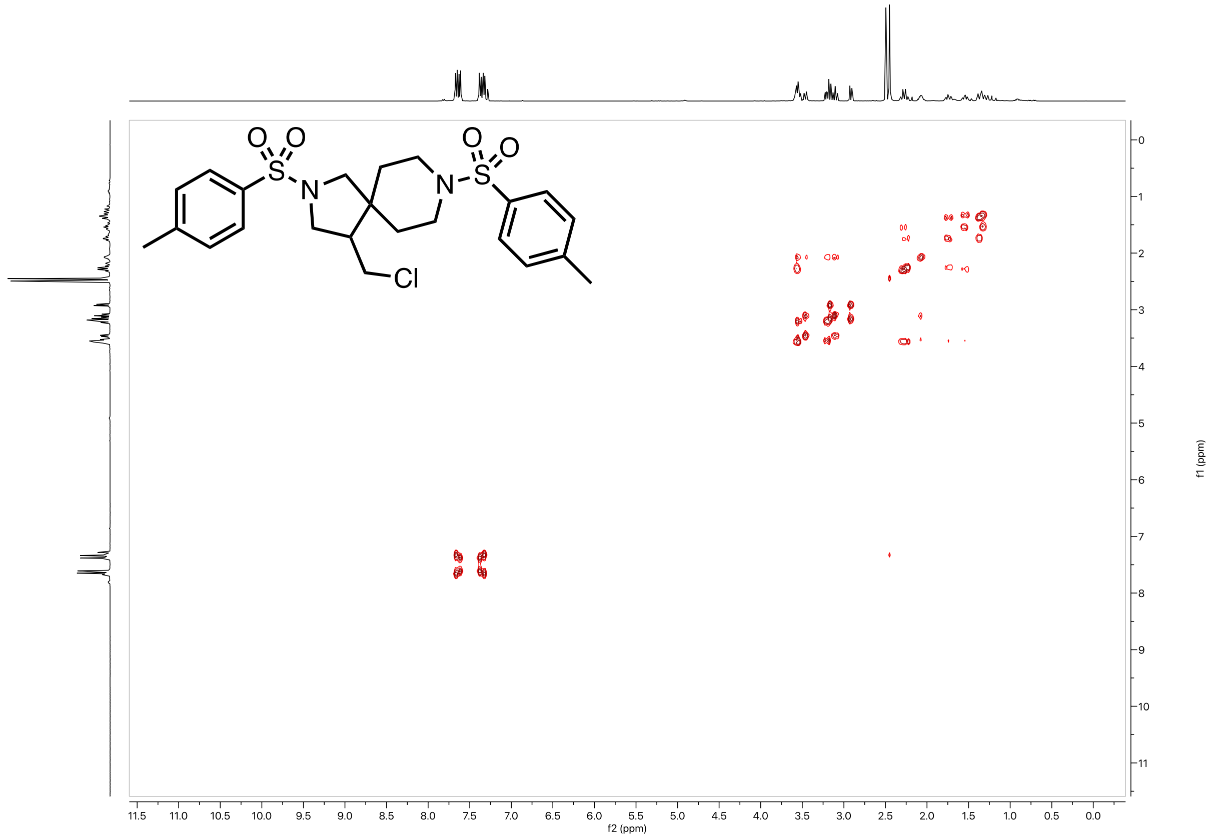

COSY of *4-(chloromethyl)-2,8-ditosyl-2,8-diazaspiro[4.5]decane* ($CDCl_3$).

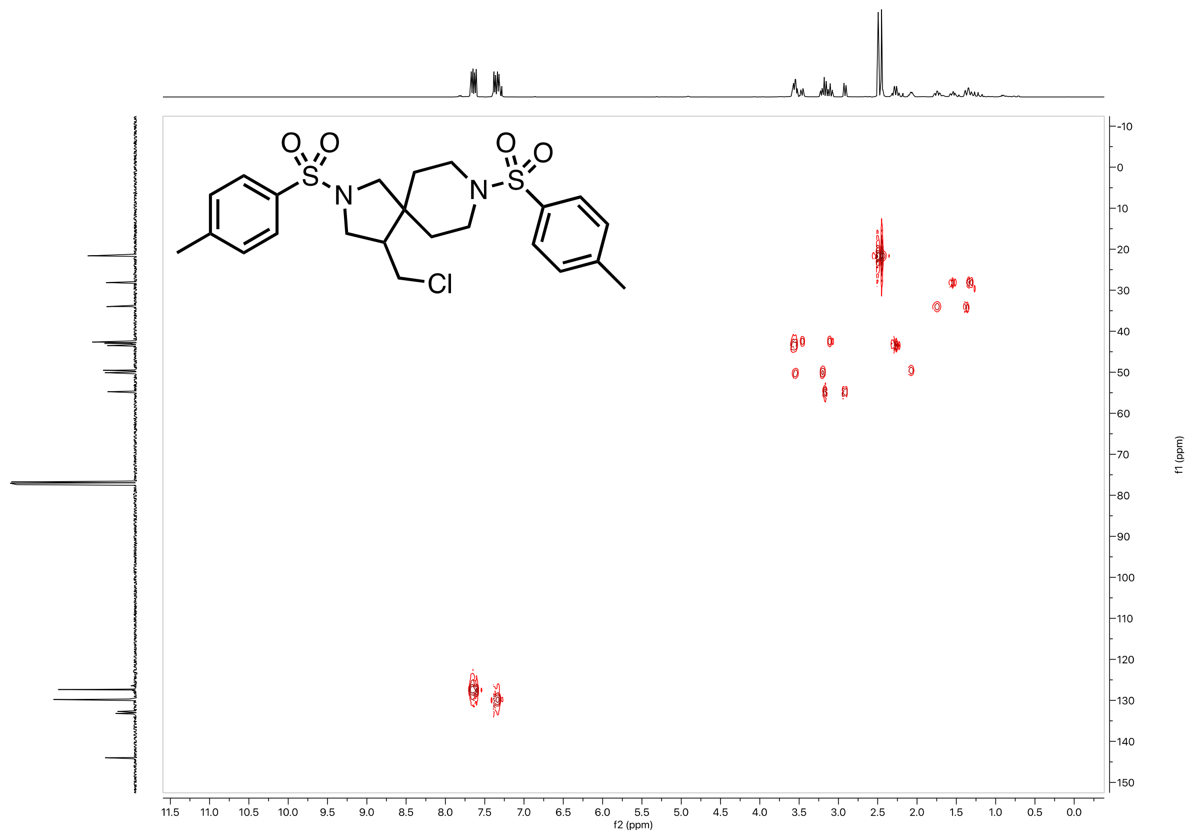

HSQC of *4-(chloromethyl)-2,8-ditosyl-2,8-diazaspiro[4.5]decane* ($CDCl_3$).

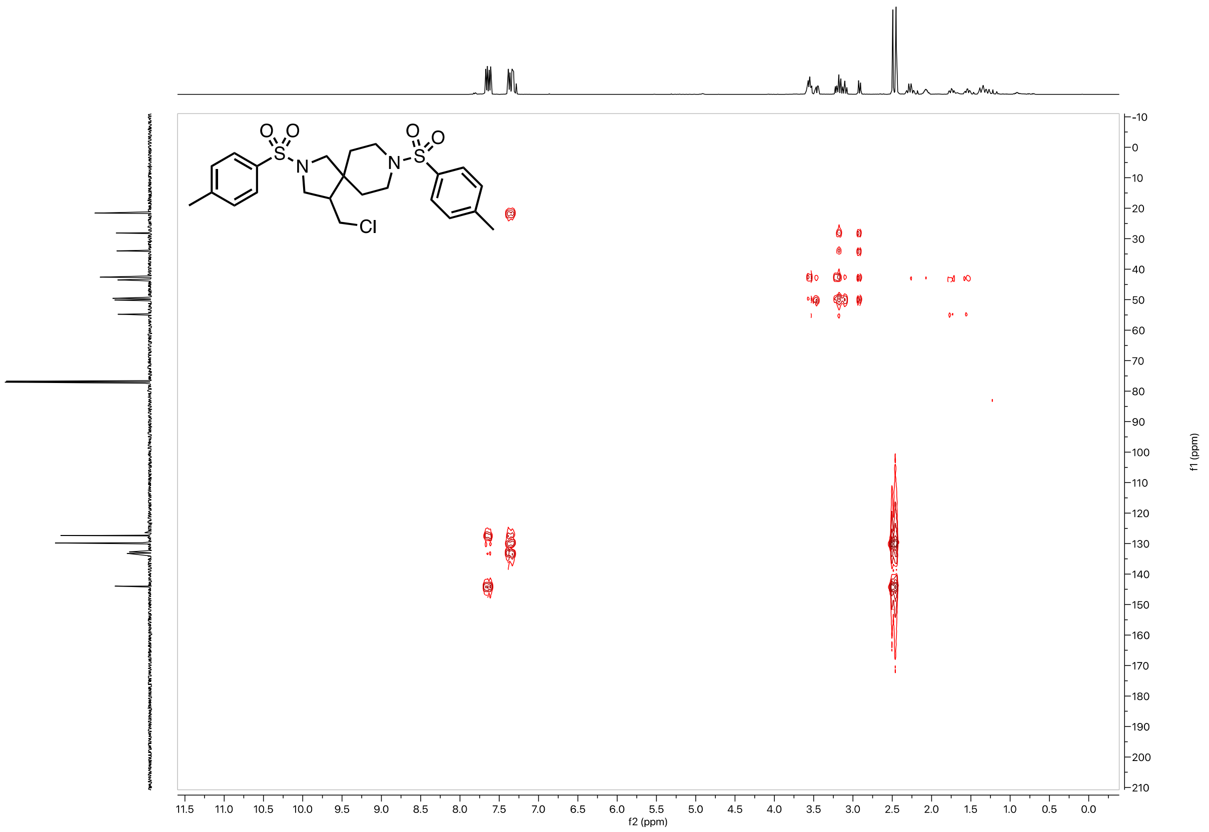

HMBC of *4-(chloromethyl)-2,8-ditosyl-2,8-diazaspiro[4.5]decane* ($CDCl_3$).

4-(bromomethyl)-2,8-ditosyl-2,8-diazaspiro[4.5]decane (3ad)

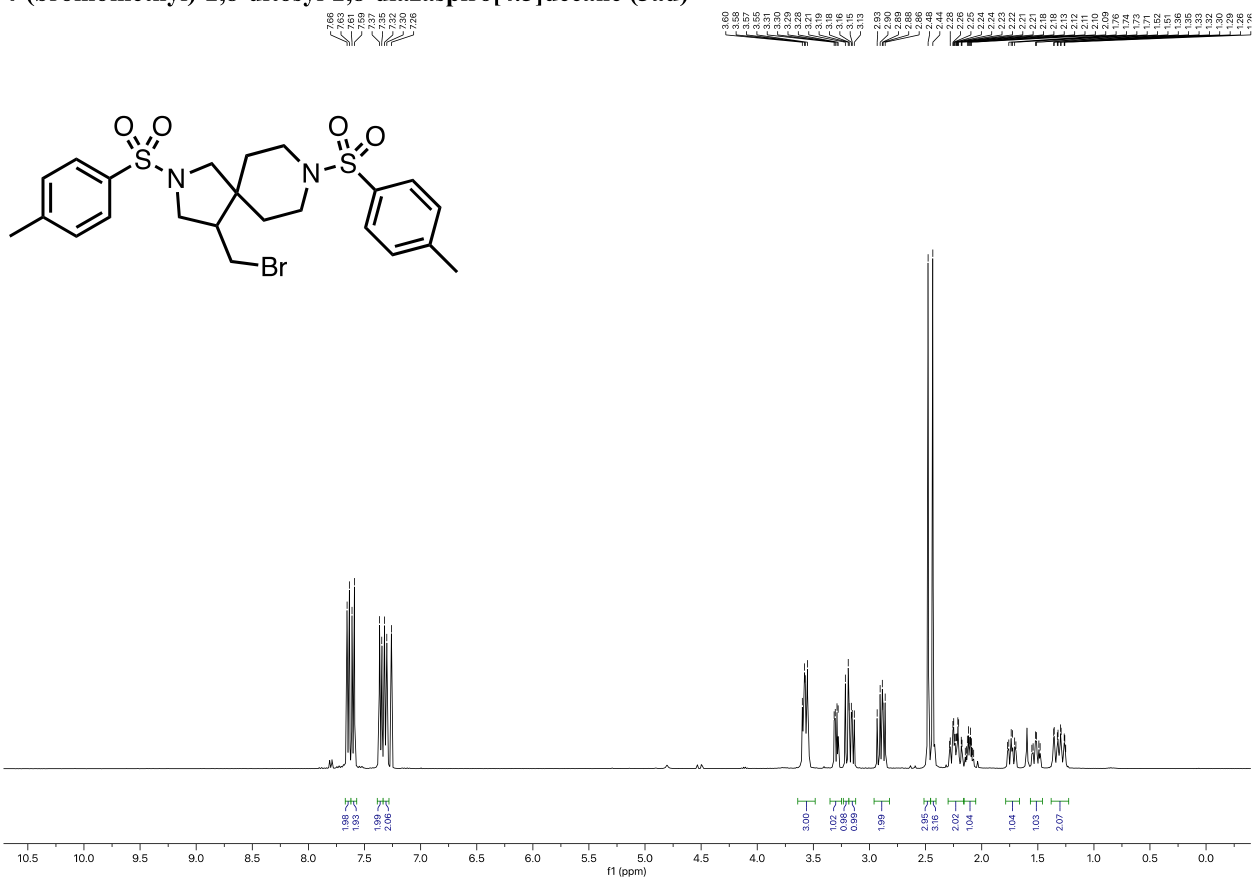

^{1}H NMR of *4-(bromomethyl)-2,8-ditosyl-2,8-diazaspiro[4.5]decane* (400 MHz, $CDCl_3$).

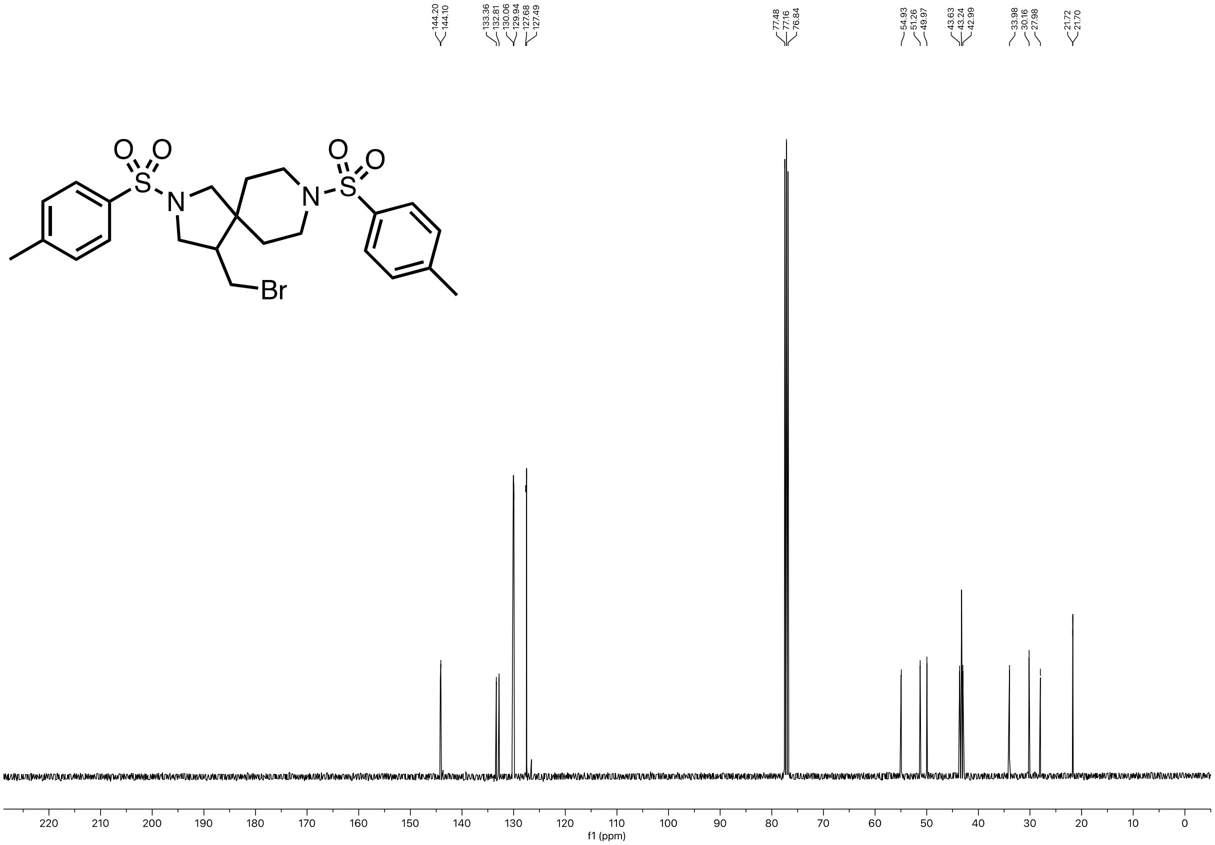

^{13}C{^{1}H} NMR of *4-(bromomethyl)-2,8-ditosyl-2,8-diazaspiro[4.5]decane* (101 MHz, $CDCl_3$).

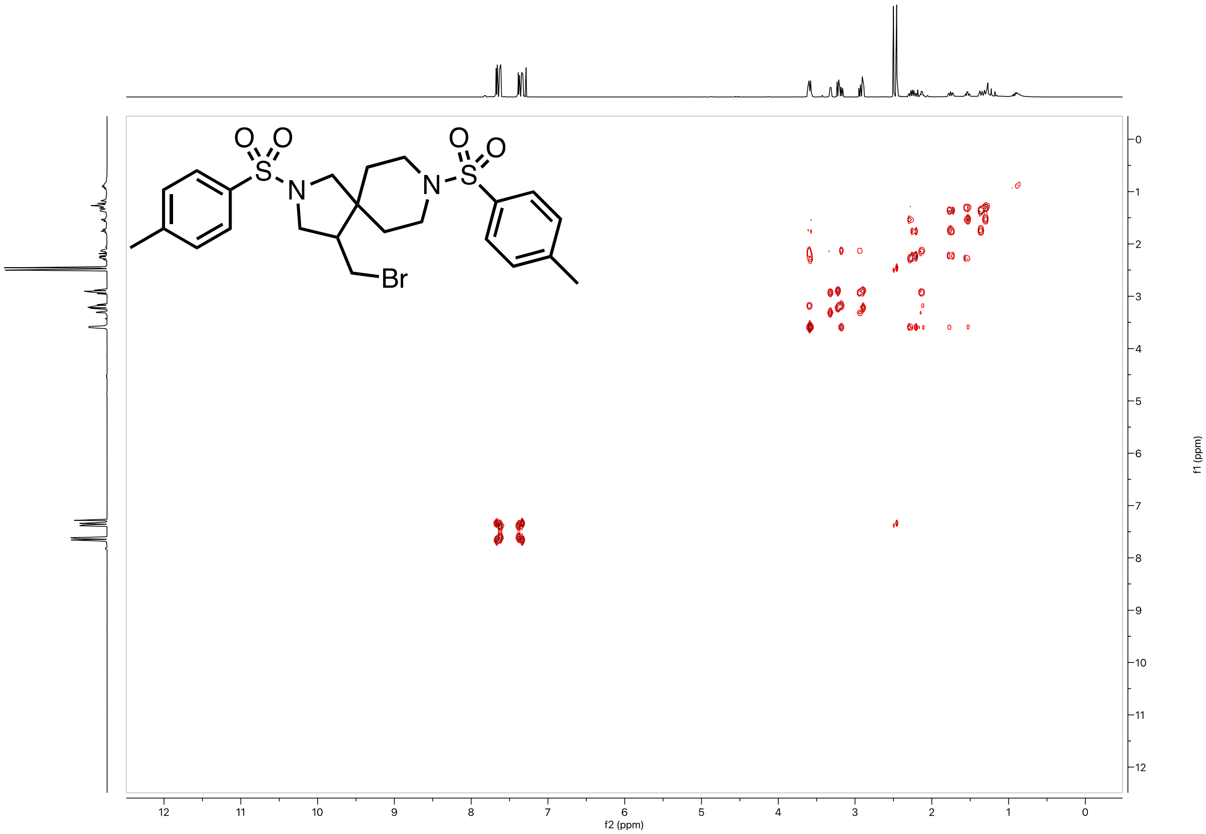

COSY of *4-(bromomethyl)-2,8-ditosyl-2,8-diazaspiro[4.5]decane* ($CDCl_3$).

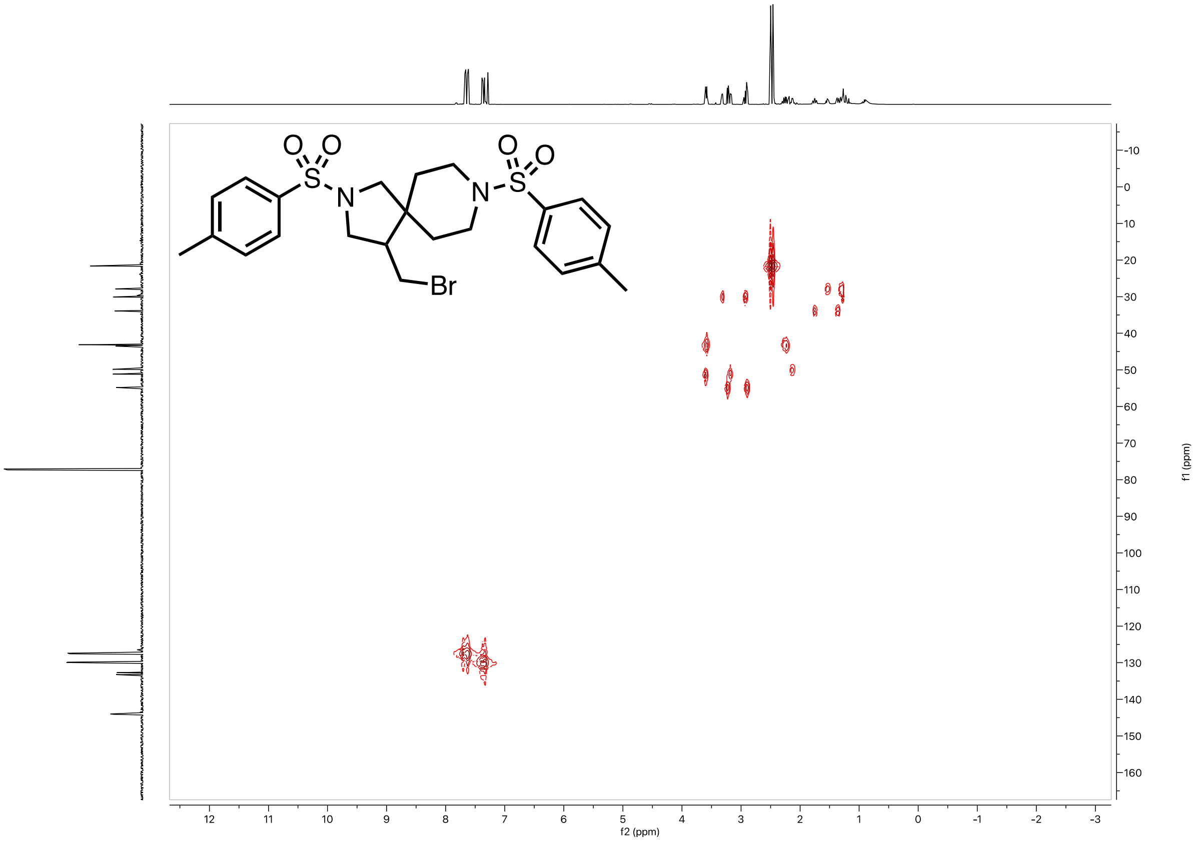

HSQC of *4-(bromomethyl)-2,8-ditosyl-2,8-diazaspiro[4.5]decane* ($CDCl_3$).

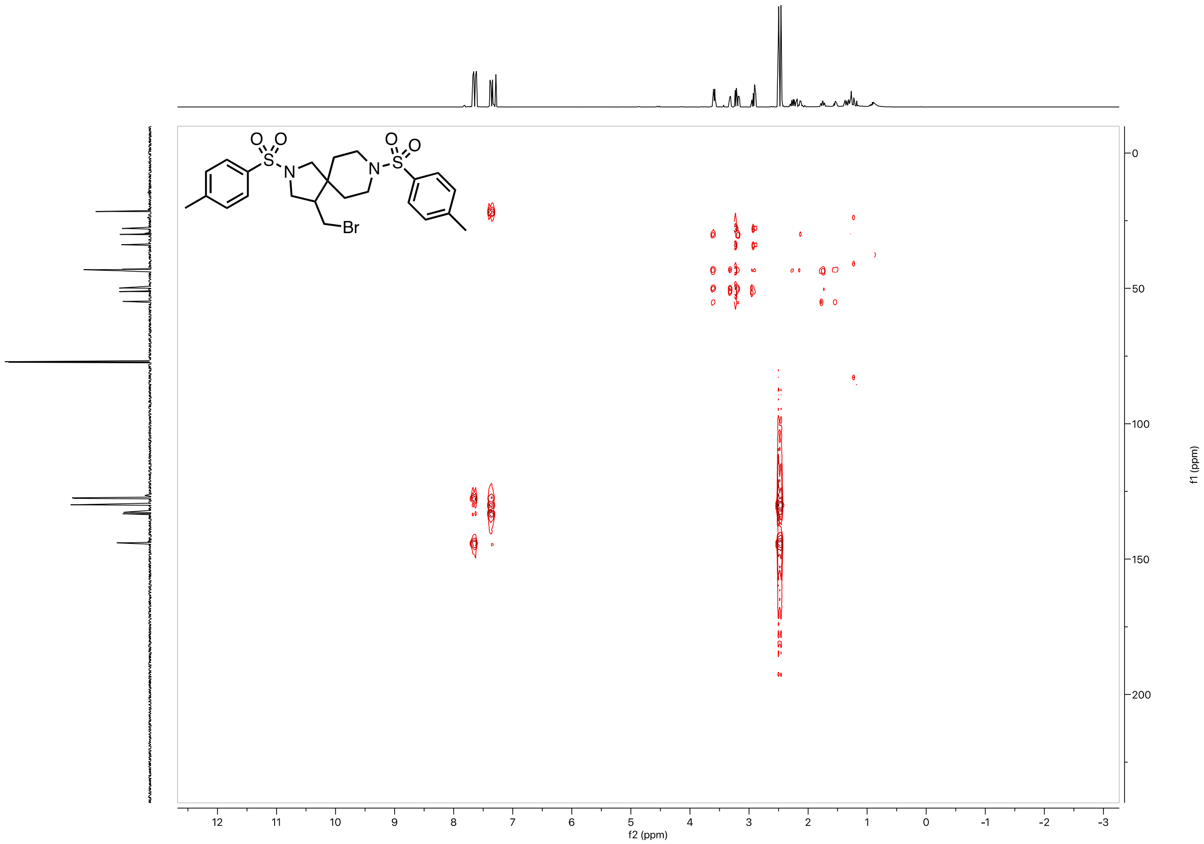

HMBC of *4-(bromomethyl)-2,8-ditosyl-2,8-diazaspiro[4.5]decane* ($CDCl_3$).

4-(chloromethyl)-2-((4-nitrophenyl)sulfonyl)-8-tosyl-2,8-diazaspiro[4.5]decane (3ae)

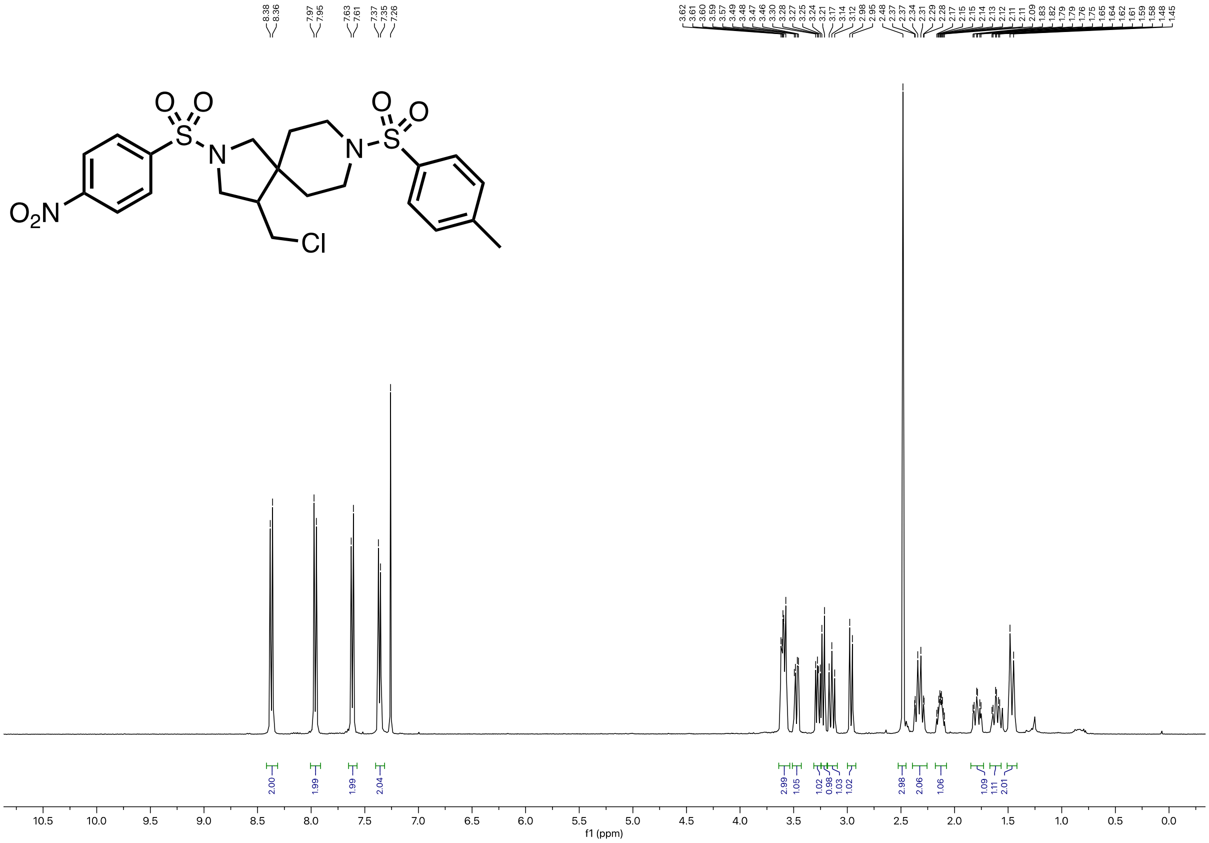

^{1}H NMR of *4-(chloromethyl)-2-((4-nitrophenyl)sulfonyl)-8-tosyl-2,8-diazaspiro[4.5]decane* (400 MHz, $CDCl_3$).

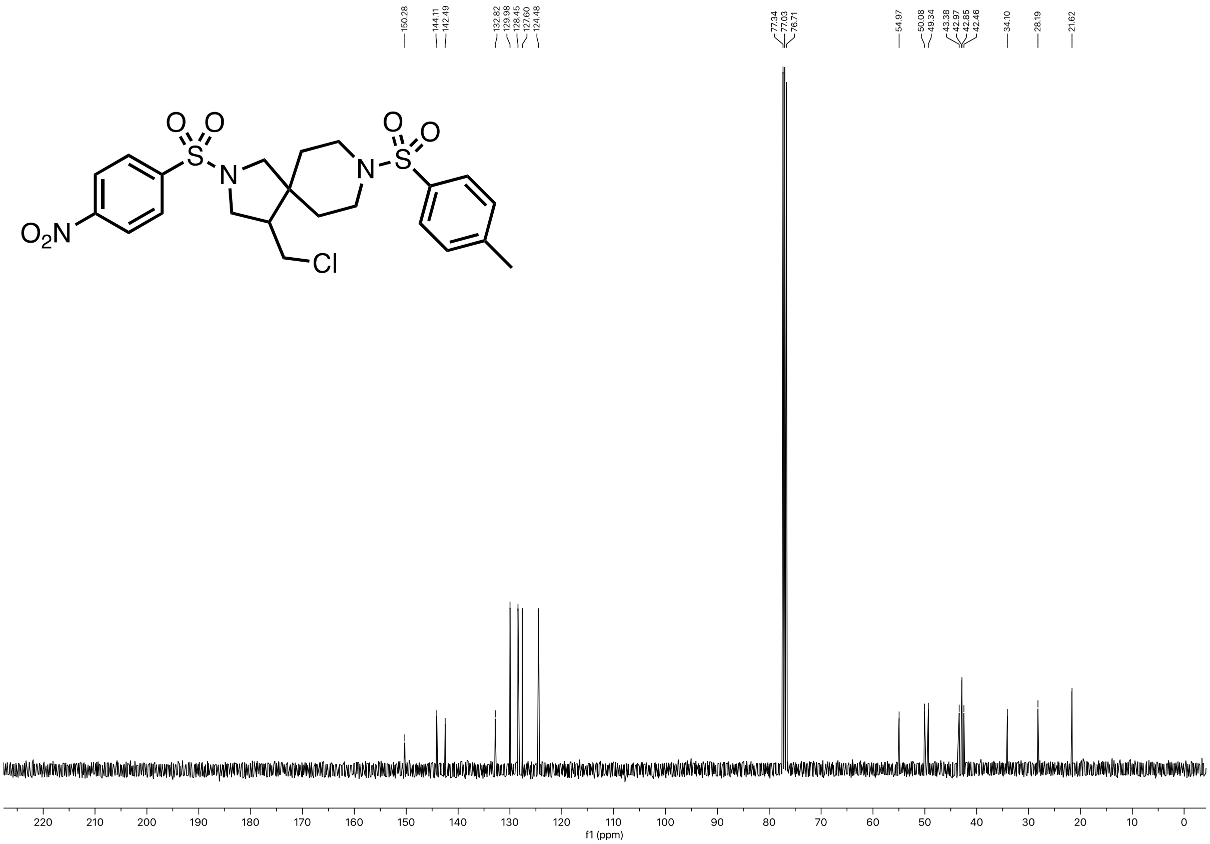

$^{13}C\{^{1}H\}$ NMR of *4-(chloromethyl)-2-((4-nitrophenyl)sulfonyl)-8-tosyl-2,8-diazaspiro[4.5]decane* (101 MHz, $CDCl_3$).

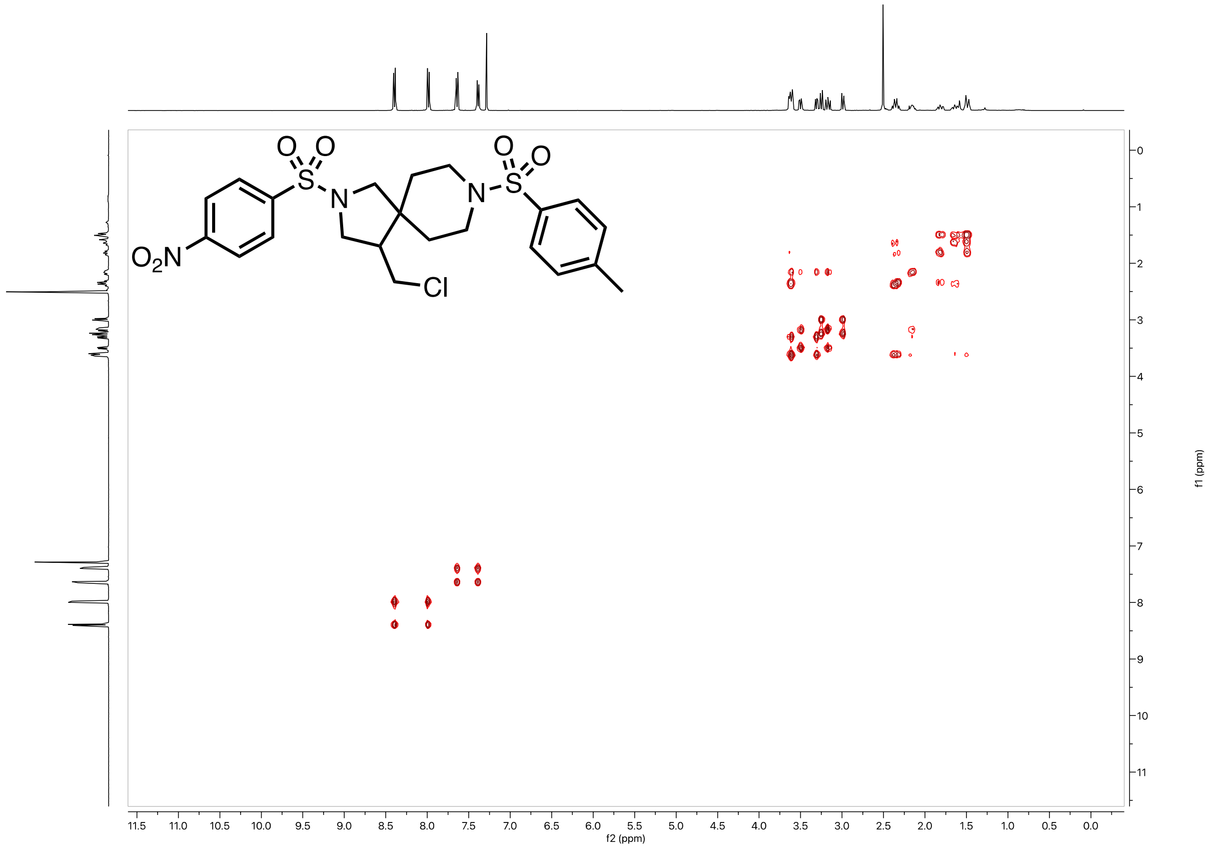

COSY of *4-(chloromethyl)-2-((4-nitrophenyl)sulfonyl)-8-tosyl-2,8-diazaspiro[4.5]decane* ($CDCl_3$).

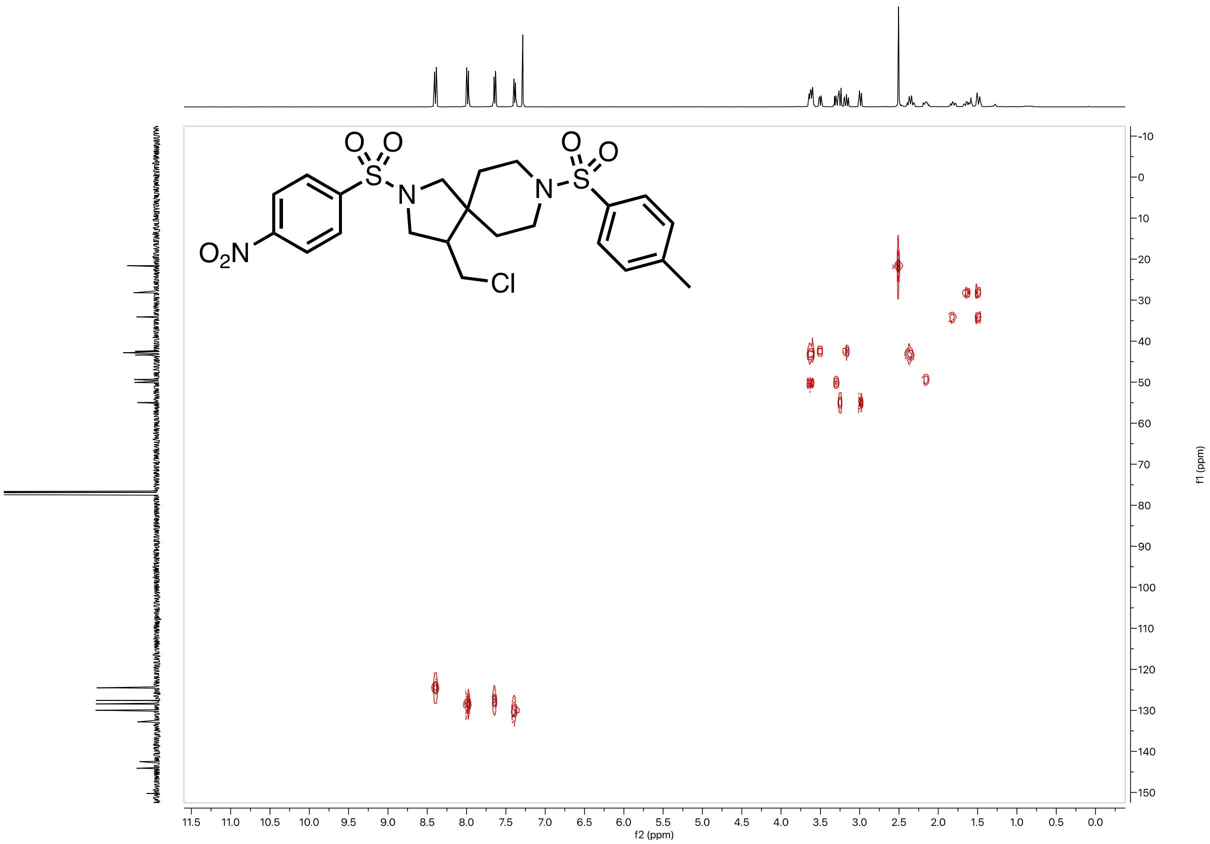

HSQC of *4-(chloromethyl)-2-((4-nitrophenyl)sulfonyl)-8-tosyl-2,8-diazaspiro[4.5]decane* ($CDCl_3$).

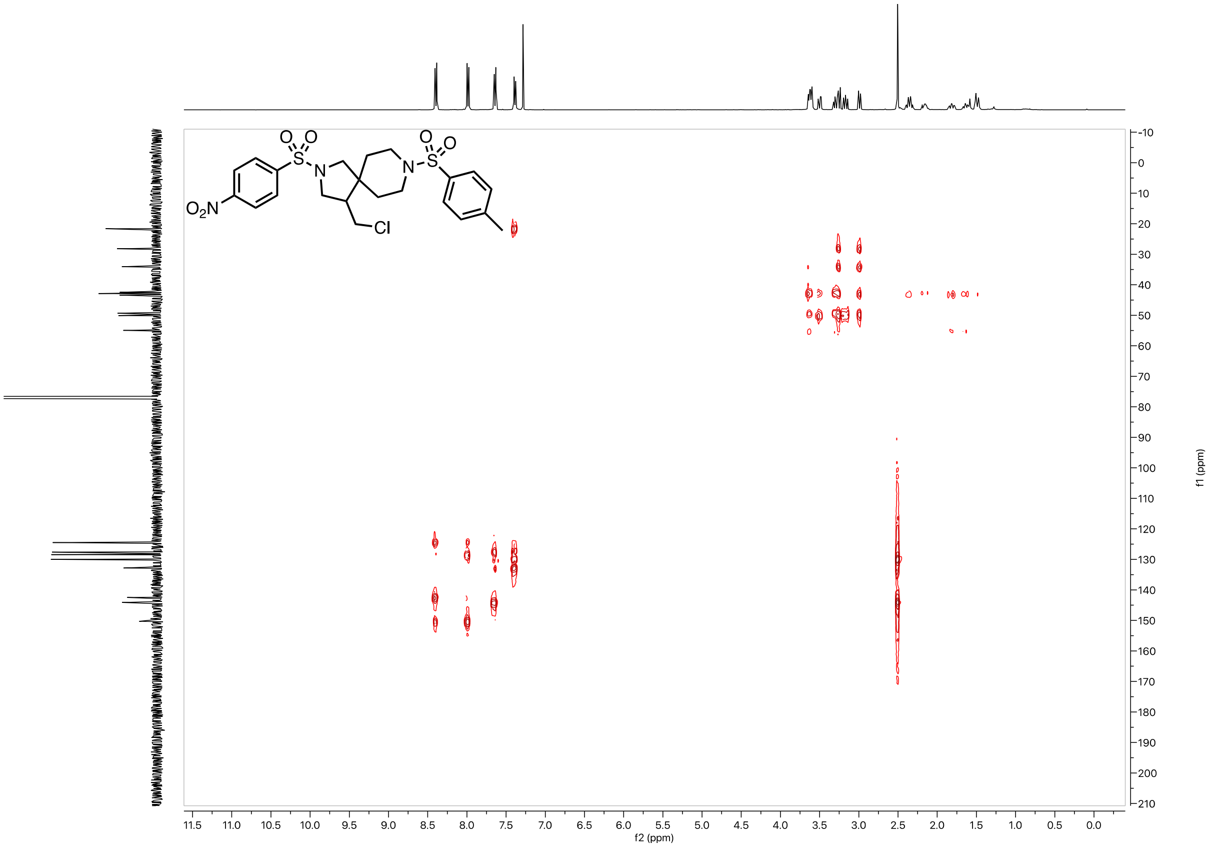

HMBC of *4-(chloromethyl)-2-((4-nitrophenyl)sulfonyl)-8-tosyl-2,8-diazaspiro[4.5]decane* ($CDCl_3$).

4-(bromomethyl)-2-((4-nitrophenyl)sulfonyl)-8-tosyl-2,8-diazaspiro[4.5]decane (3af)

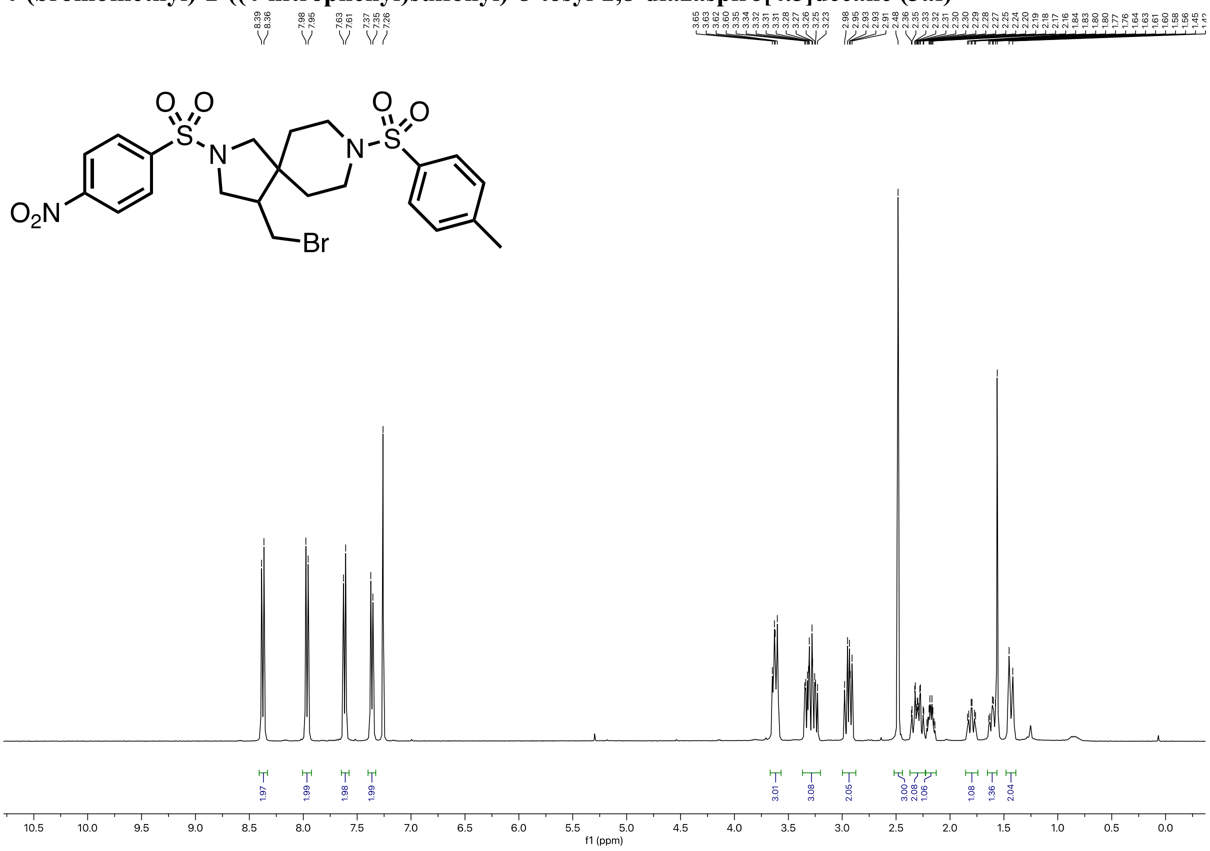

^{1}H NMR of *4-(bromomethyl)-2-((4-nitrophenyl)sulfonyl)-8-tosyl-2,8-diazaspiro[4.5]decane* (400 MHz, $CDCl_3$).

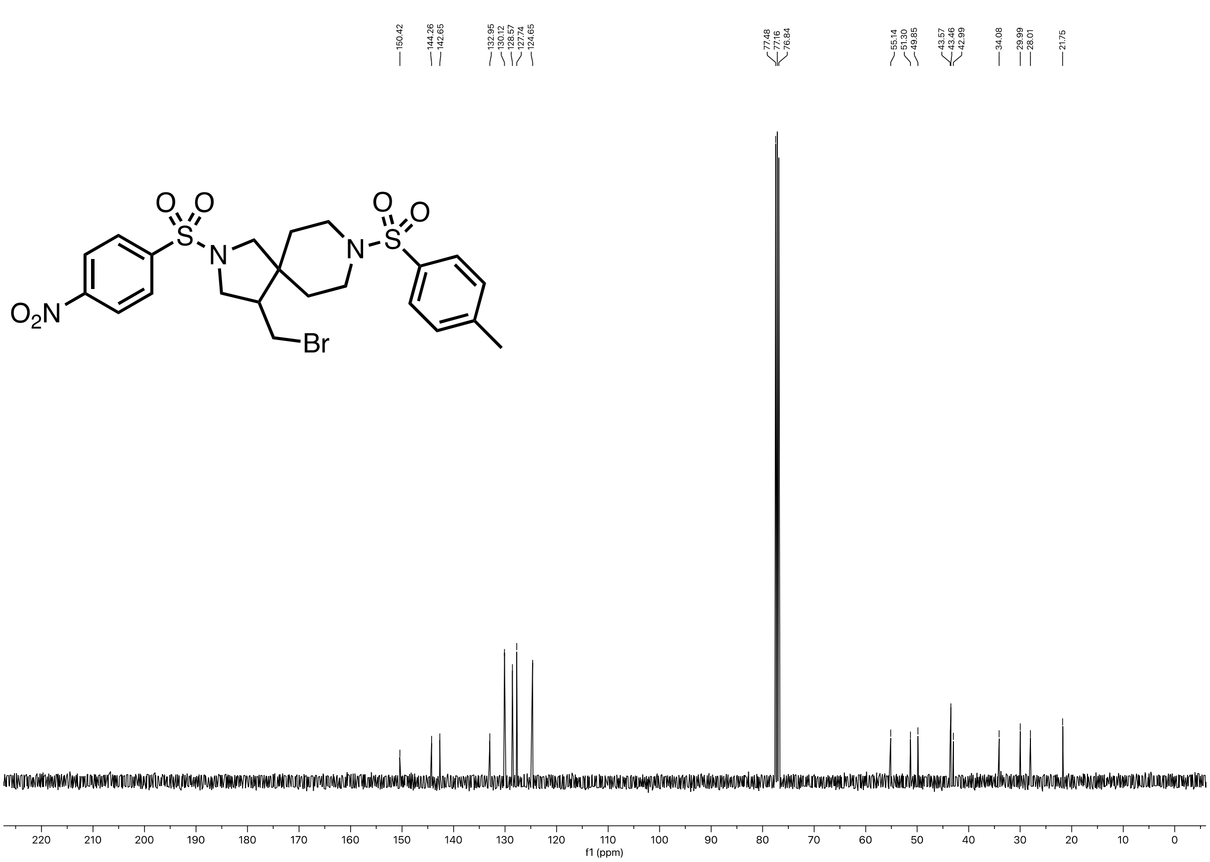

^{13}C{^{1}H} NMR of *4-(bromomethyl)-2-((4-nitrophenyl)sulfonyl)-8-tosyl-2,8-diazaspiro[4.5]decane* (101 MHz, $CDCl_3$).

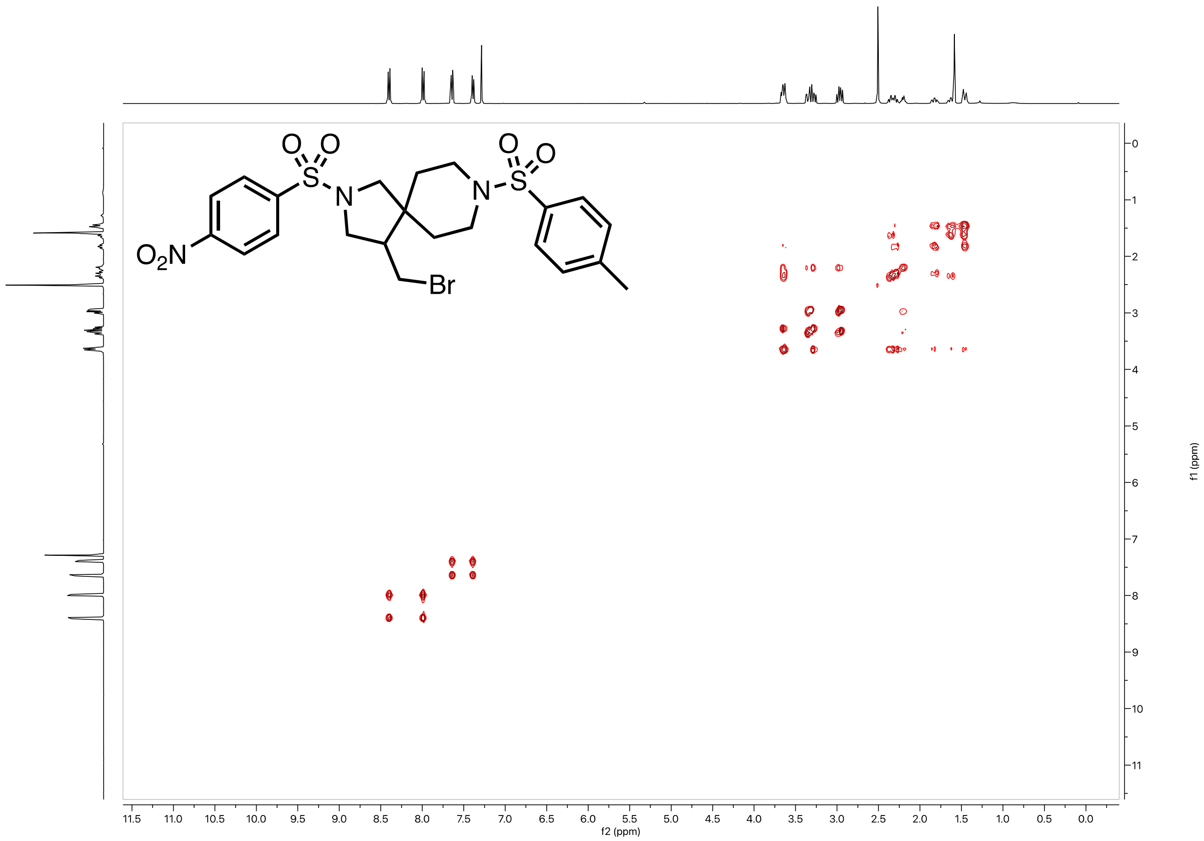

COSY of *4-(bromomethyl)-2-((4-nitrophenyl)sulfonyl)-8-tosyl-2,8-diazaspiro[4.5]decane* ($CDCl_3$).

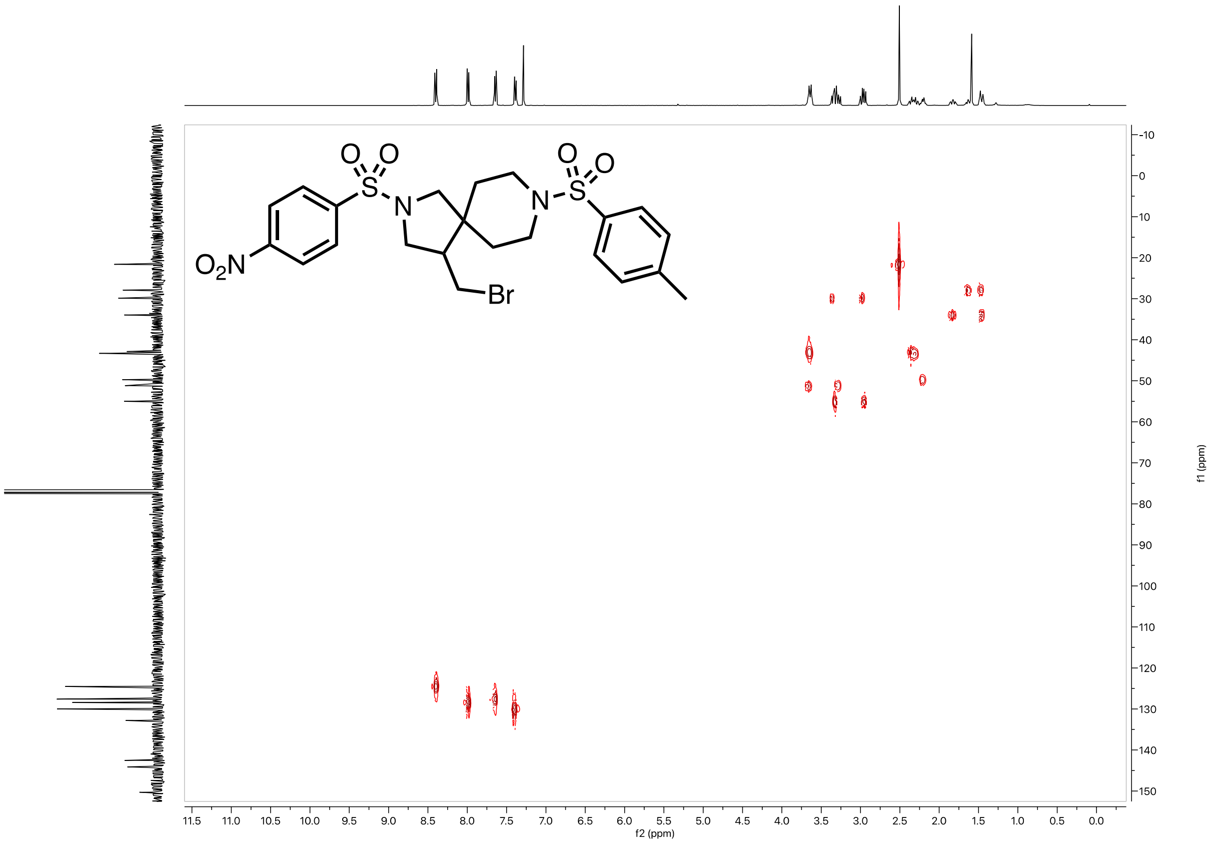

HSQC of *4-(bromomethyl)-2-((4-nitrophenyl)sulfonyl)-8-tosyl-2,8-diazaspiro[4.5]decane* ($CDCl_3$).

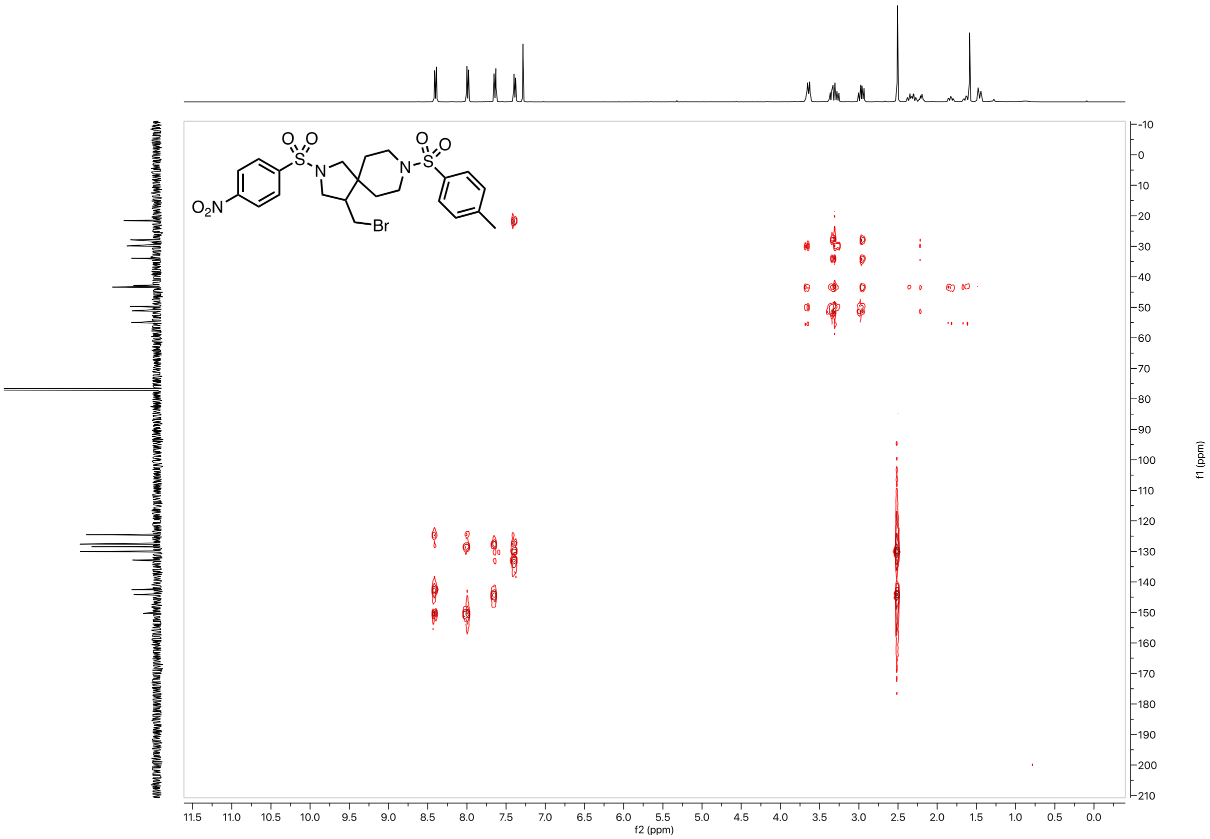

HMBC of *4-(bromomethyl)-2-((4-nitrophenyl)sulfonyl)-8-tosyl-2,8-diazaspiro[4.5]decane* ($CDCl_3$).

4-(chloromethyl)-2-((4-nitrophenyl)sulfonyl)-10-tosyl-2,10-diazadispiro[4.1.5^{7}.1^{5}]tridecane (3ag)

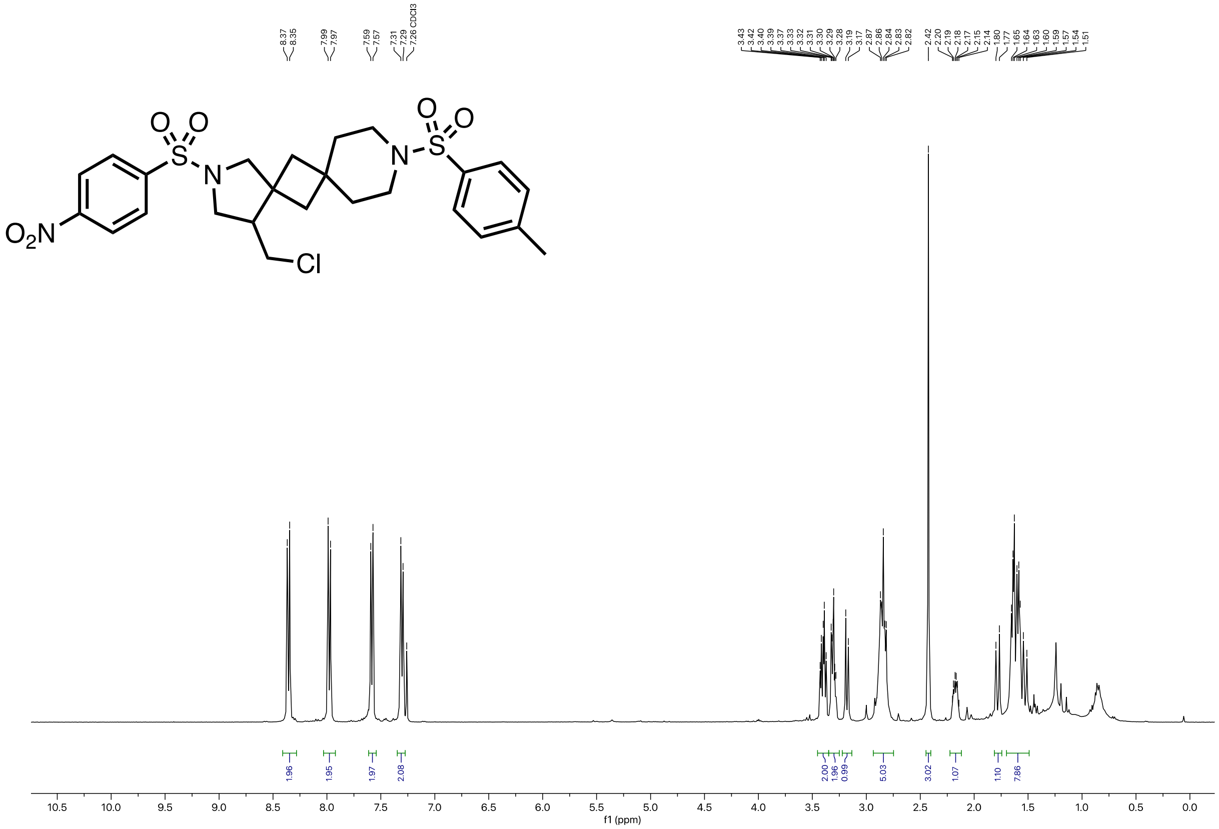

^{1}H NMR of *4-(chloromethyl)-2-((4-nitrophenyl)sulfonyl)-10-tosyl-2,10-diazadispiro[4.1.5^{7}.1^{5}]tridecane* (400 MHz, $CDCl_3$).

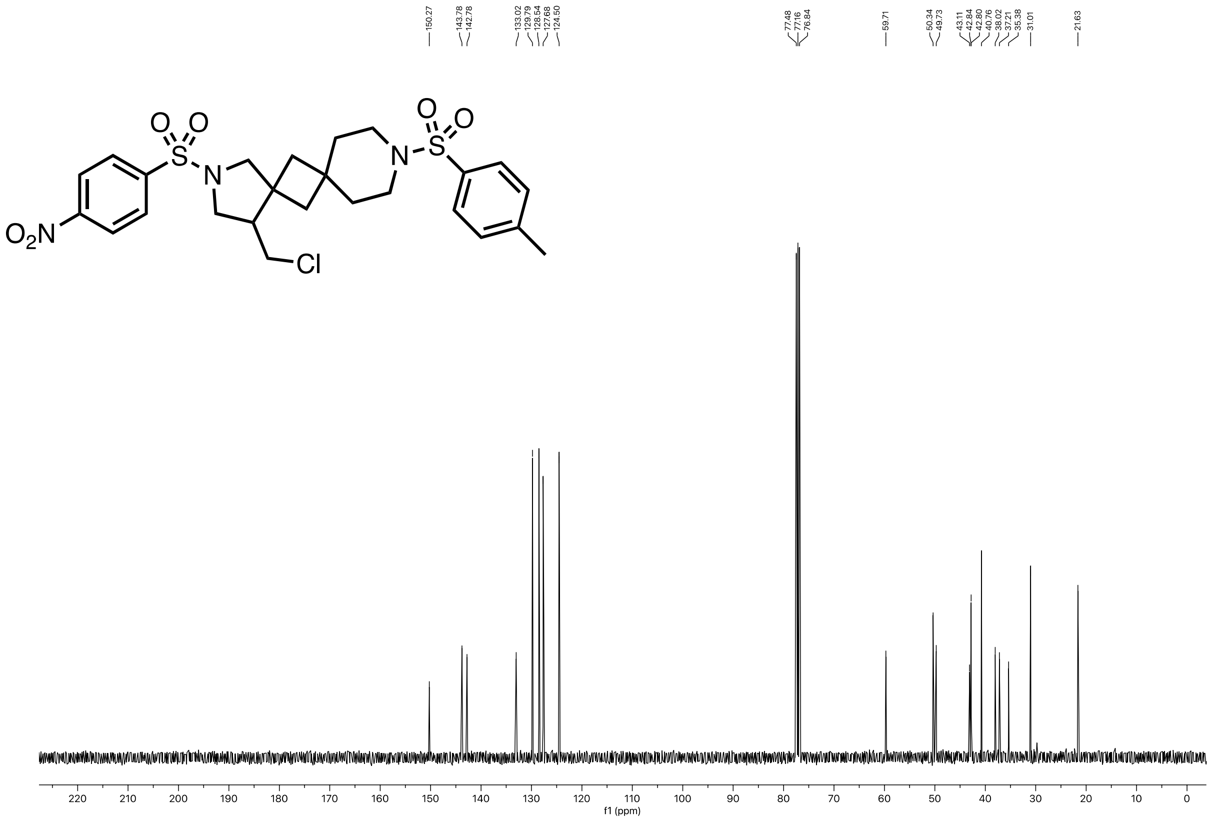

$^{13}C\{^{1}H\}$ NMR of *4-(chloromethyl)-2-((4-nitrophenyl)sulfonyl)-10-tosyl-2,10-diazadispiro[4.1.5^{7}.1^{5}]tridecane* (101 MHz, $CDCl_3$).

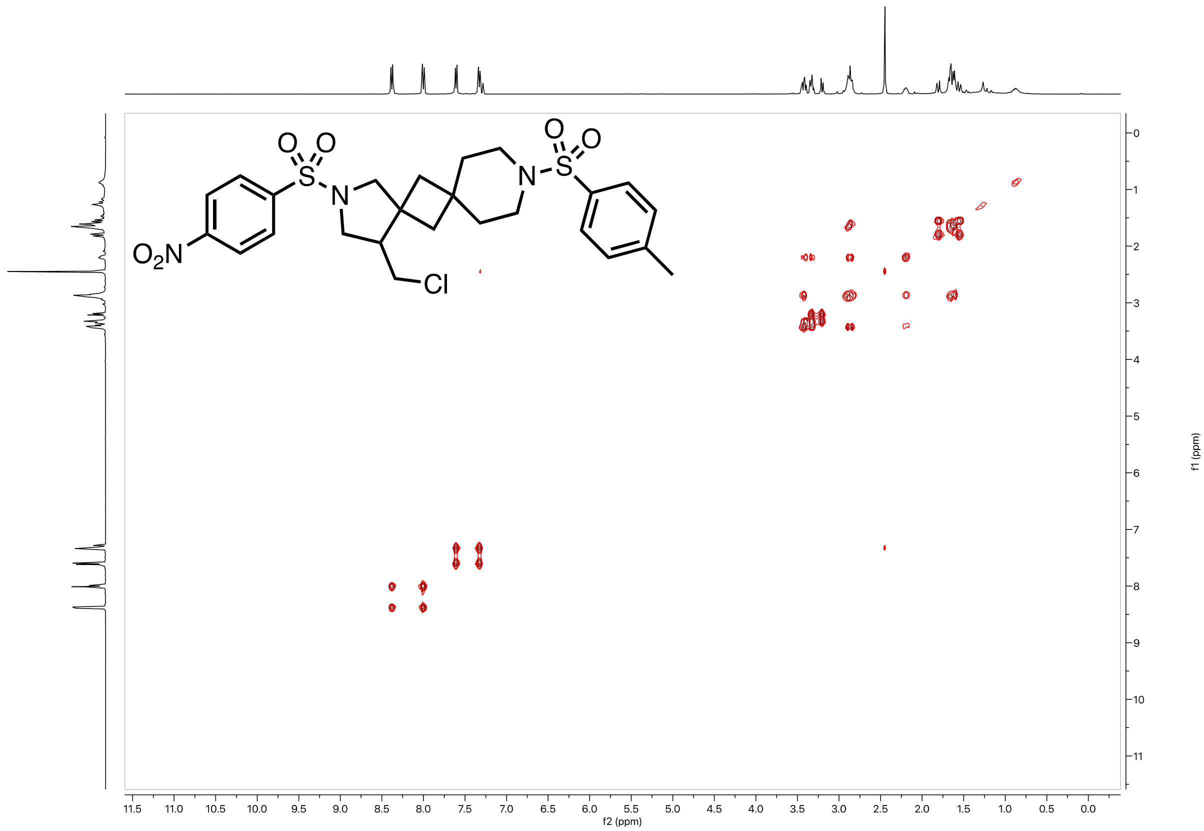

COSY of *4-(chloromethyl)-2-((4-nitrophenyl)sulfonyl)-10-tosyl-2,10-diazadispiro[4.1.5^{7}.1^{5}]tridecane* ($CDCl_3$).

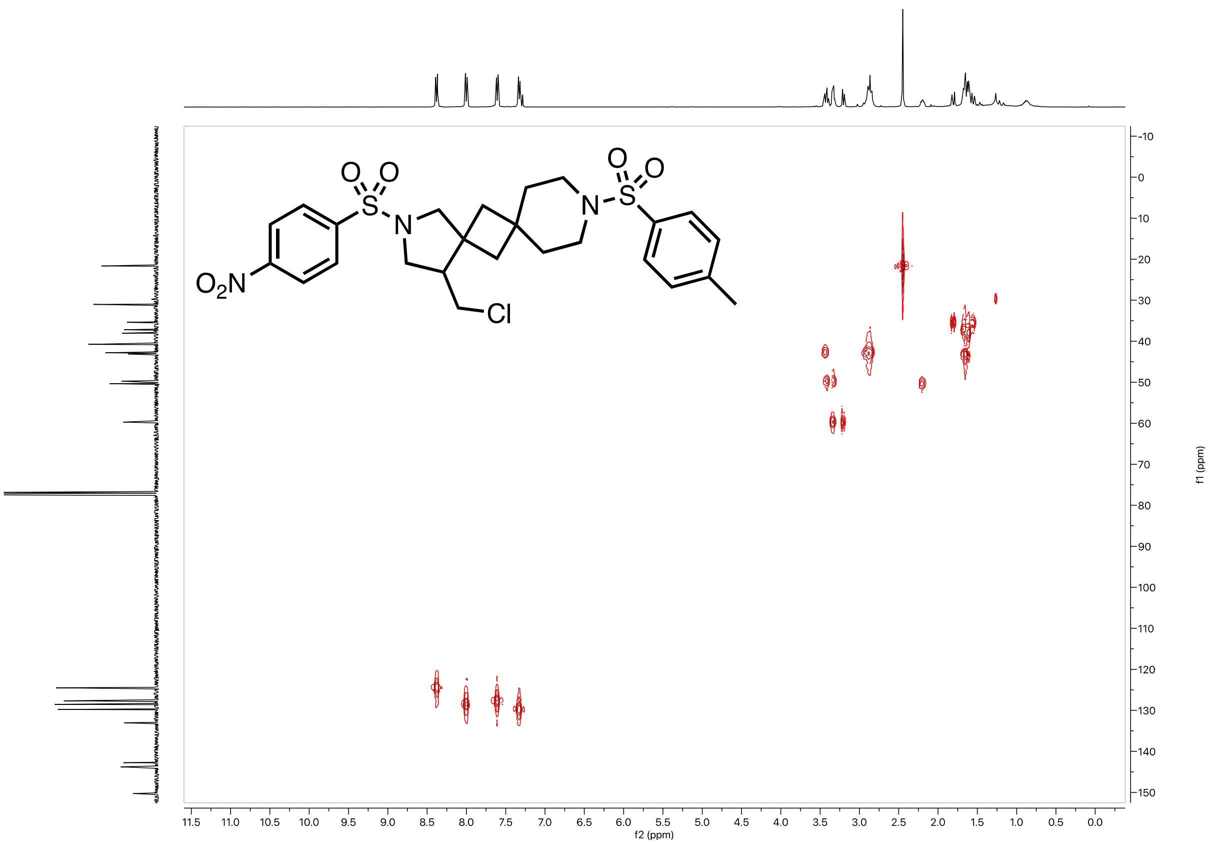

HSQC of *4-(chloromethyl)-2-((4-nitrophenyl)sulfonyl)-10-tosyl-2,10-diazadispiro[4.1.5⁷.1⁵]tridecane* ($CDCl_3$).

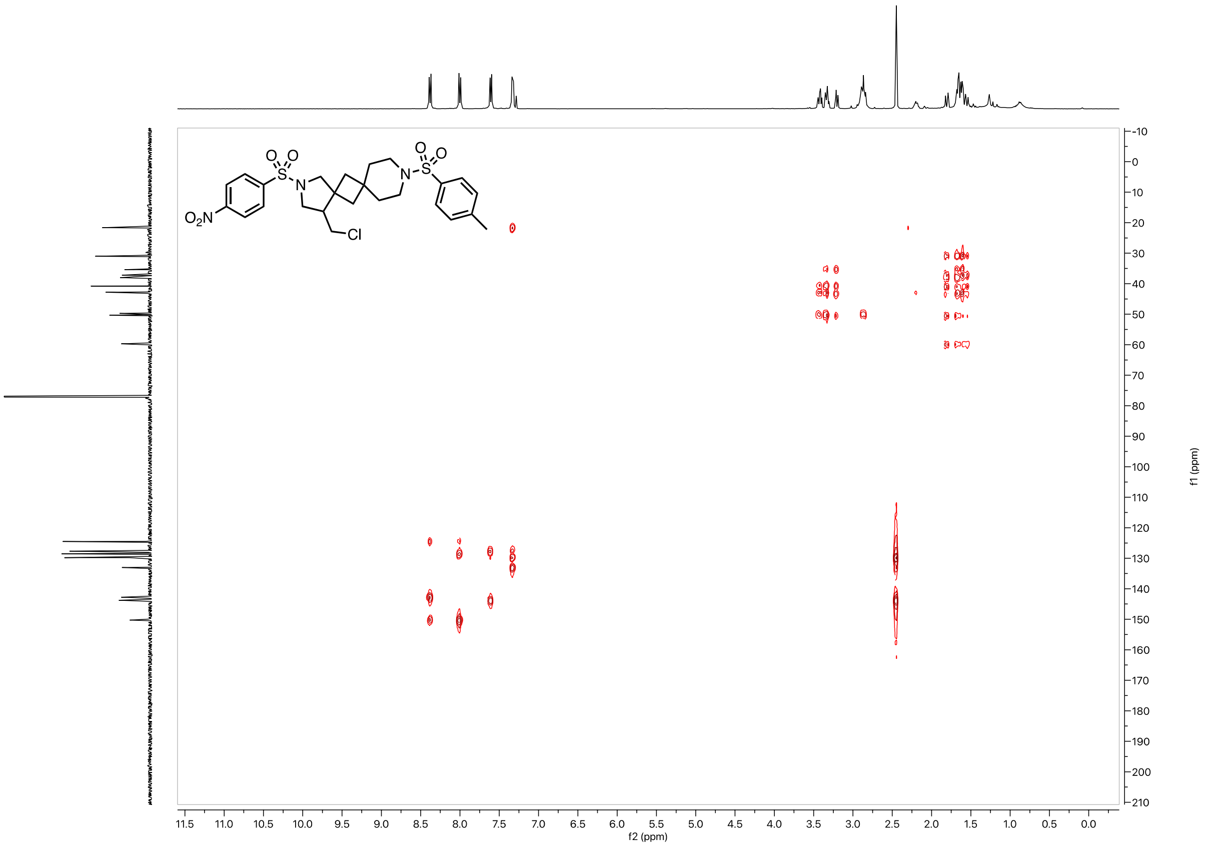

HMBC of *4-(chloromethyl)-2-((4-nitrophenyl)sulfonyl)-10-tosyl-2,10-diazadispiro[4.1.5⁷.1⁵]tridecane* ($CDCl_3$).

4-(bromomethyl)-2-((4-nitrophenyl)sulfonyl)-10-tosyl-2,10-diazadispiro[4.1.5^7.1^5]tridecane (3ah)

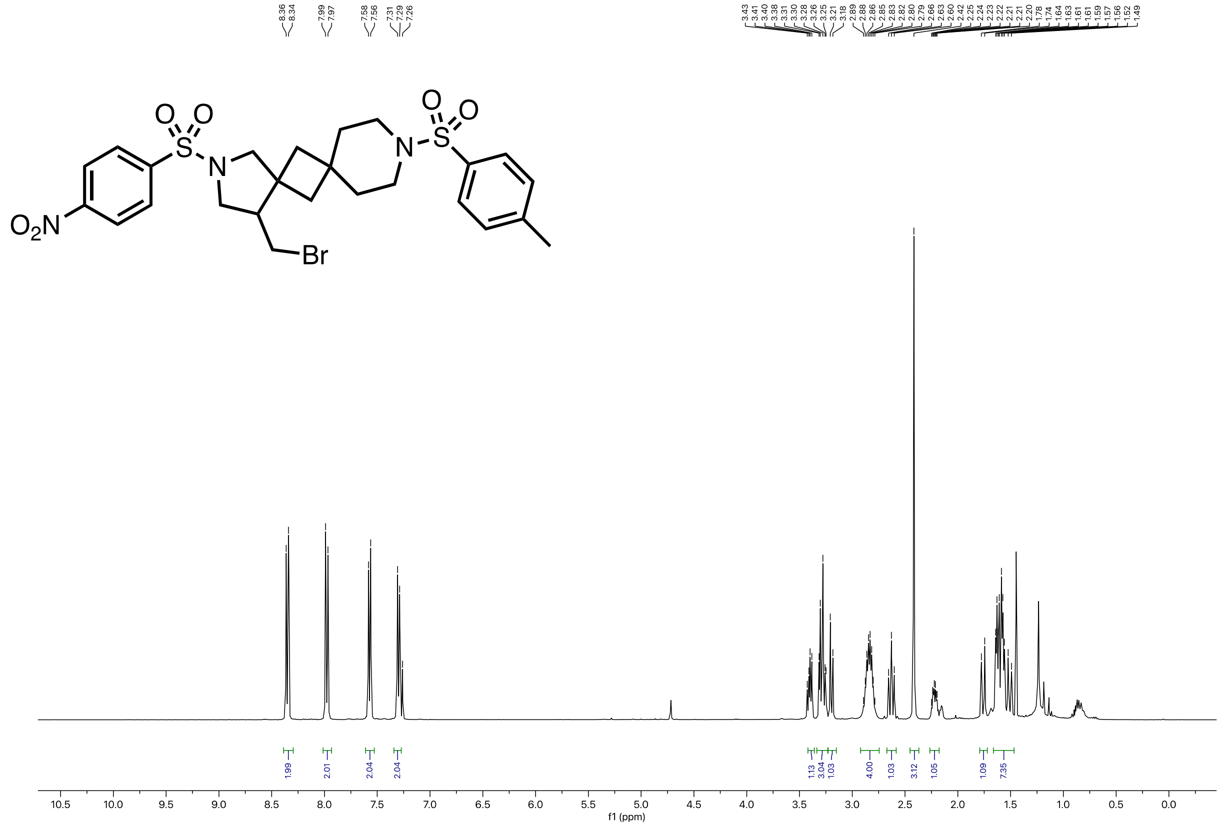

^{1}H NMR of *4-(bromomethyl)-2-((4-nitrophenyl)sulfonyl)-10-tosyl-2,10-diazadispiro[4.1.5^7.1^5]tridecane* (400 MHz, $CDCl_3$).

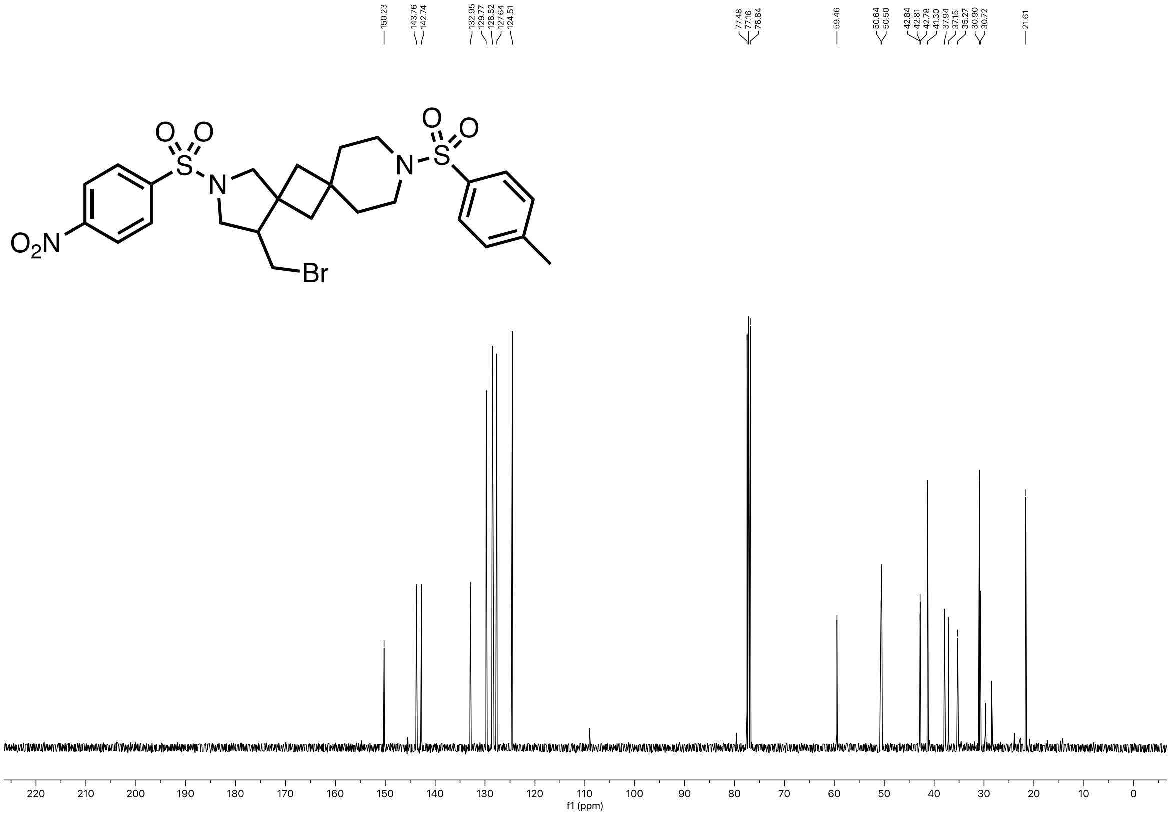

$^{13}C\{^1H\}$ NMR of *4-(bromomethyl)-2-((4-nitrophenyl)sulfonyl)-10-tosyl-2,10-diazadispiro[4.1.5^7.1^5]tridecane* (101 MHz, $CDCl_3$).

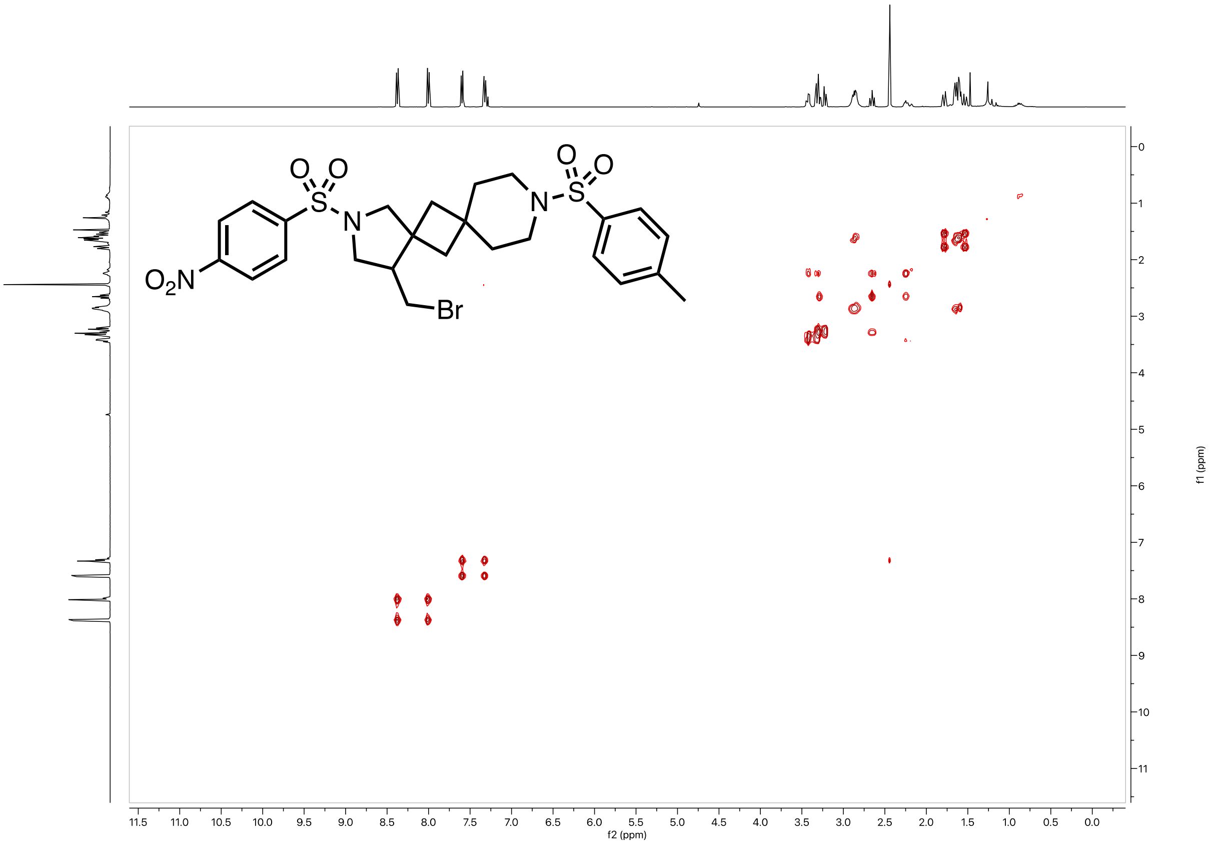

COSY of *4-(bromomethyl)-2-((4-nitrophenyl)sulfonyl)-10-tosyl-2,10-diazadispiro[4.1.5^{7}.1^{5}]tridecane* ($CDCl_3$).

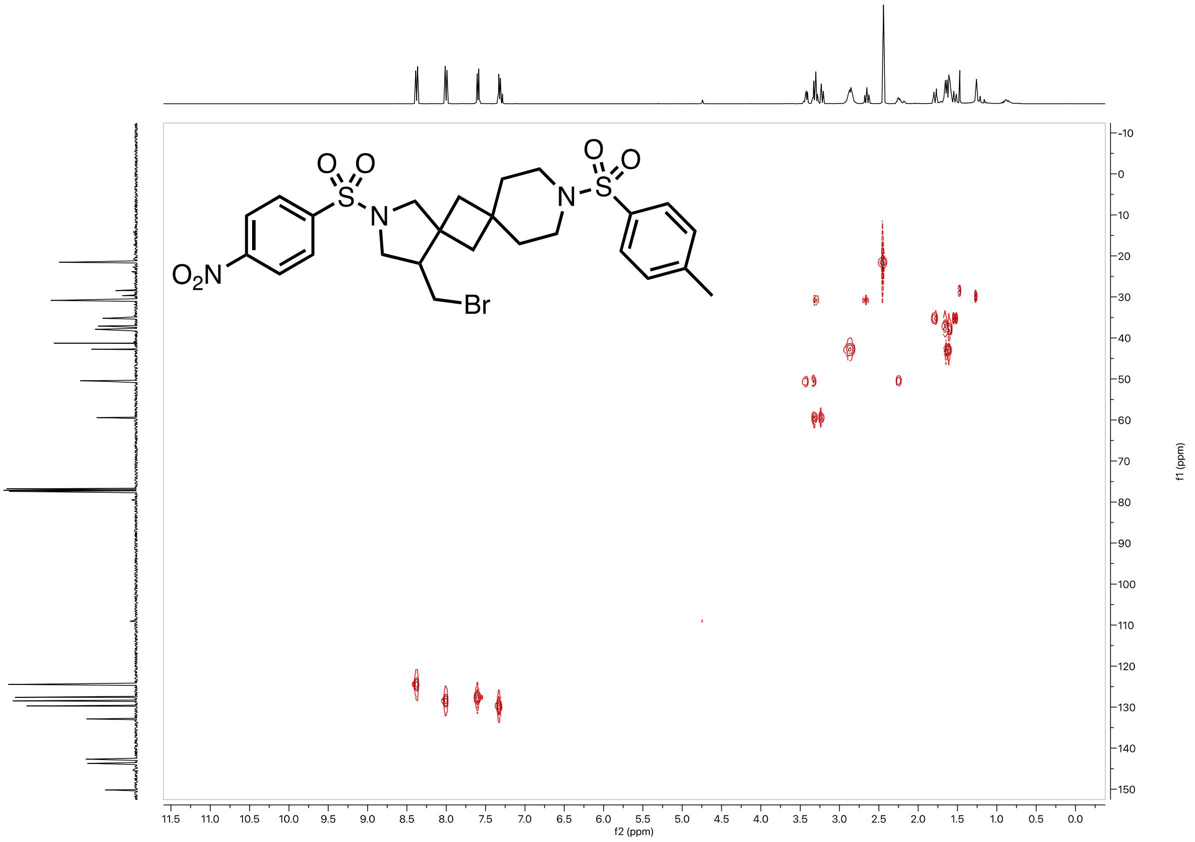

HSQC of *4-(bromomethyl)-2-((4-nitrophenyl)sulfonyl)-10-tosyl-2,10-diazadispiro[4.1.5^{7}.1^{5}]tridecane* ($CDCl_3$).

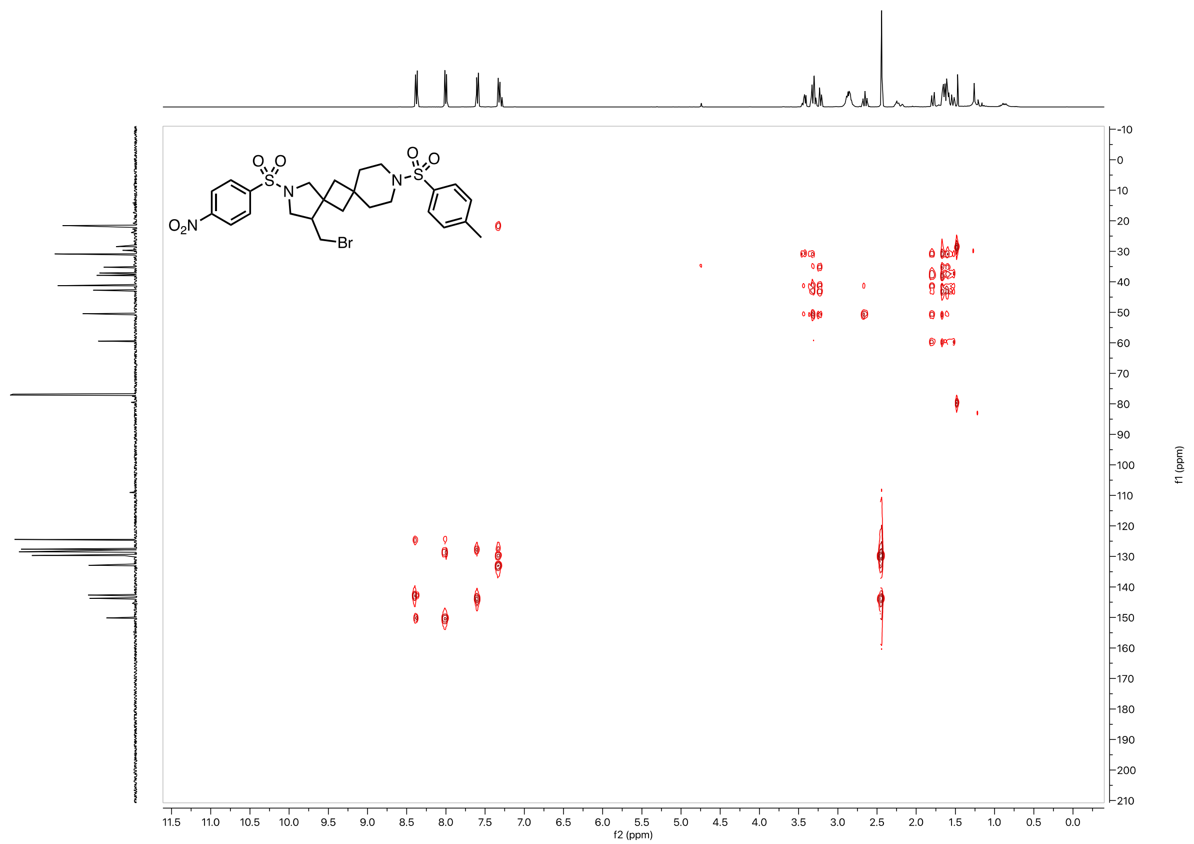

HMBC of *4-(bromomethyl)-2-((4-nitrophenyl)sulfonyl)-10-tosyl-2,10-diazadispiro[4.1.5⁷.1⁵]tridecane* ($CDCl_3$).

4-(chloromethyl)-2-((4-(5-(p-tolyl)-3-(trifluoromethyl)-1H-pyrazol-1-yl)phenyl)sulfonyl)-10-tosyl-2,10-diazadispiro[4.1.5⁷.1⁵]tridecane (3ai)

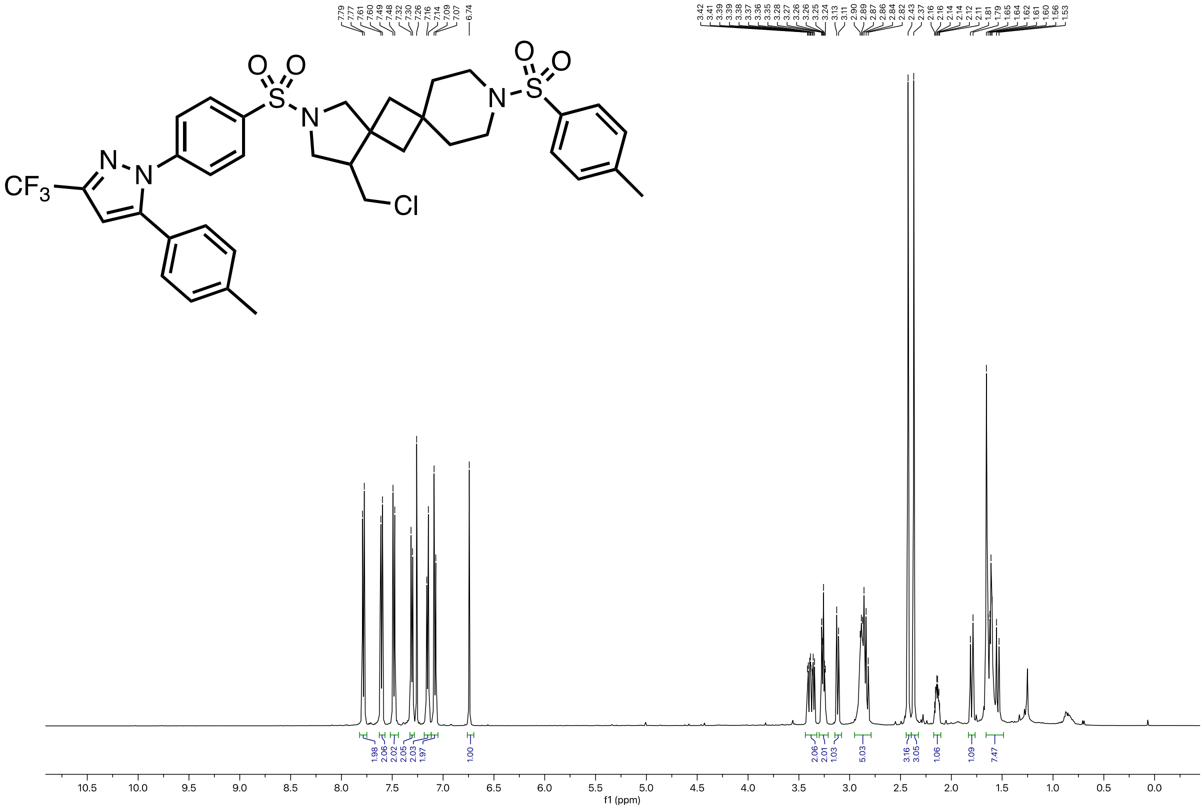

^{1}H NMR of *4-(chloromethyl)-2-((4-(5-(p-tolyl)-3-(trifluoromethyl)-1H-pyrazol-1-yl)phenyl)sulfonyl)-10-tosyl-2,10-diazadispiro[4.1.5⁷.1⁵]tridecane* (500 MHz, $CDCl_3$).

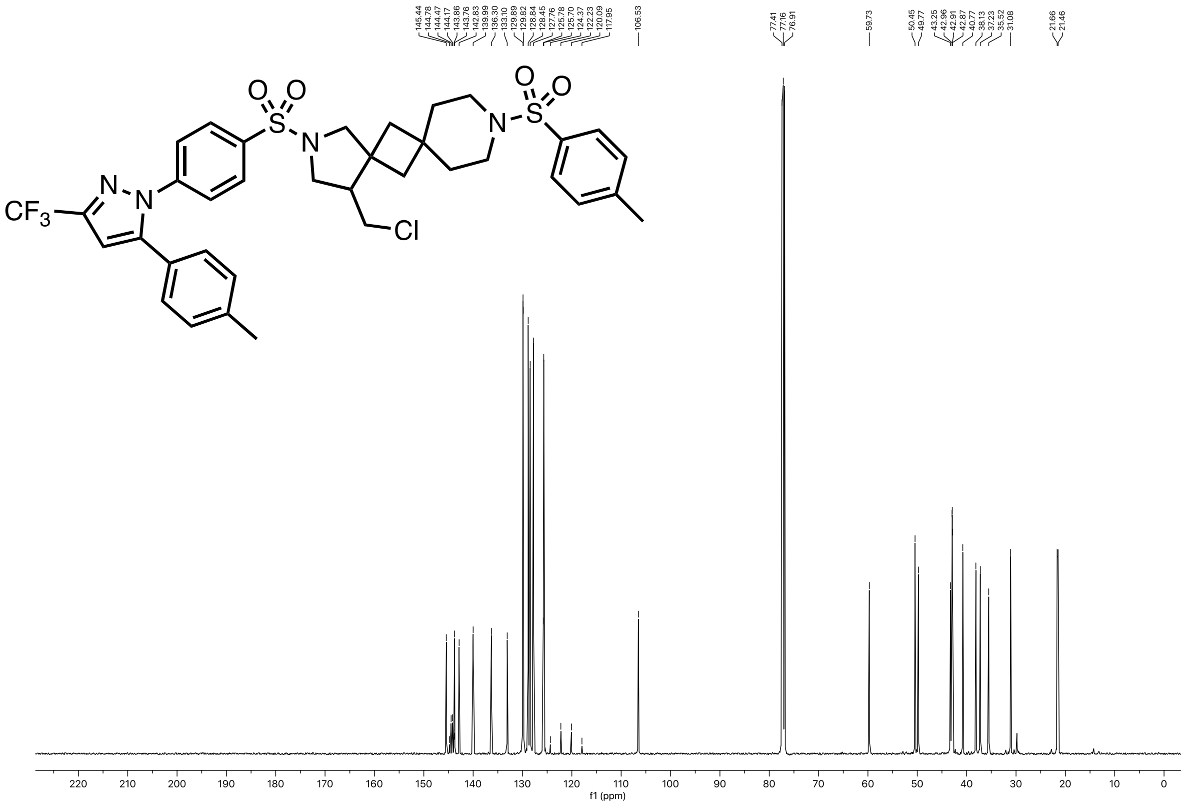

$^{13}C\{^{1}H\}$ NMR of *4-(chloromethyl)-2-((4-(5-(p-tolyl)-3-(trifluoromethyl)-1H-pyrazol-1-yl)phenyl)sulfonyl)-10-tosyl-2,10-diazadispiro[4.1.5^{7}.1^{5}]tridecane* (126MHz, $CDCl_3$).

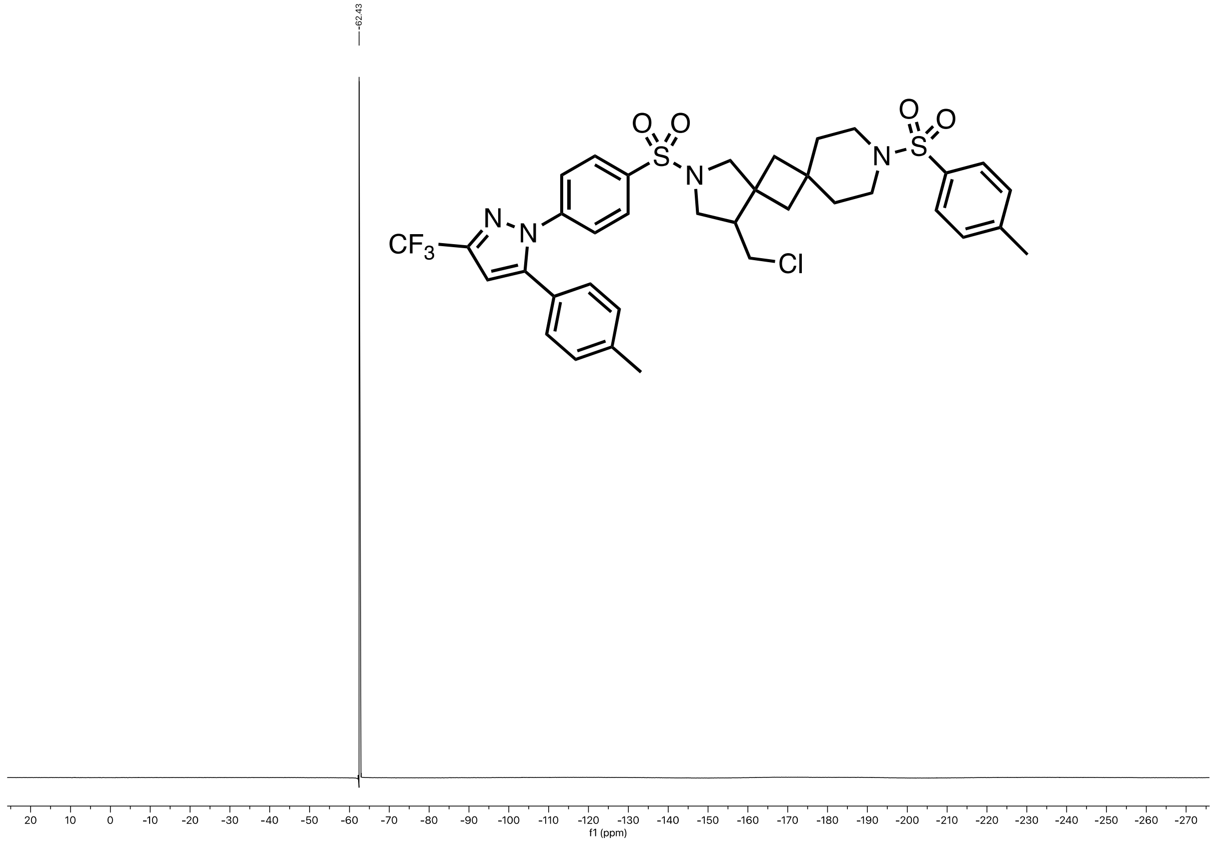

^{19}F NMR of *4-(chloromethyl)-2-((4-(5-(p-tolyl)-3-(trifluoromethyl)-1H-pyrazol-1-yl)phenyl)sulfonyl)-10-tosyl-2,10-diazadispiro[4.1.5^{7}.1^{5}]tridecane* (376 MHz, $CDCl_3$).

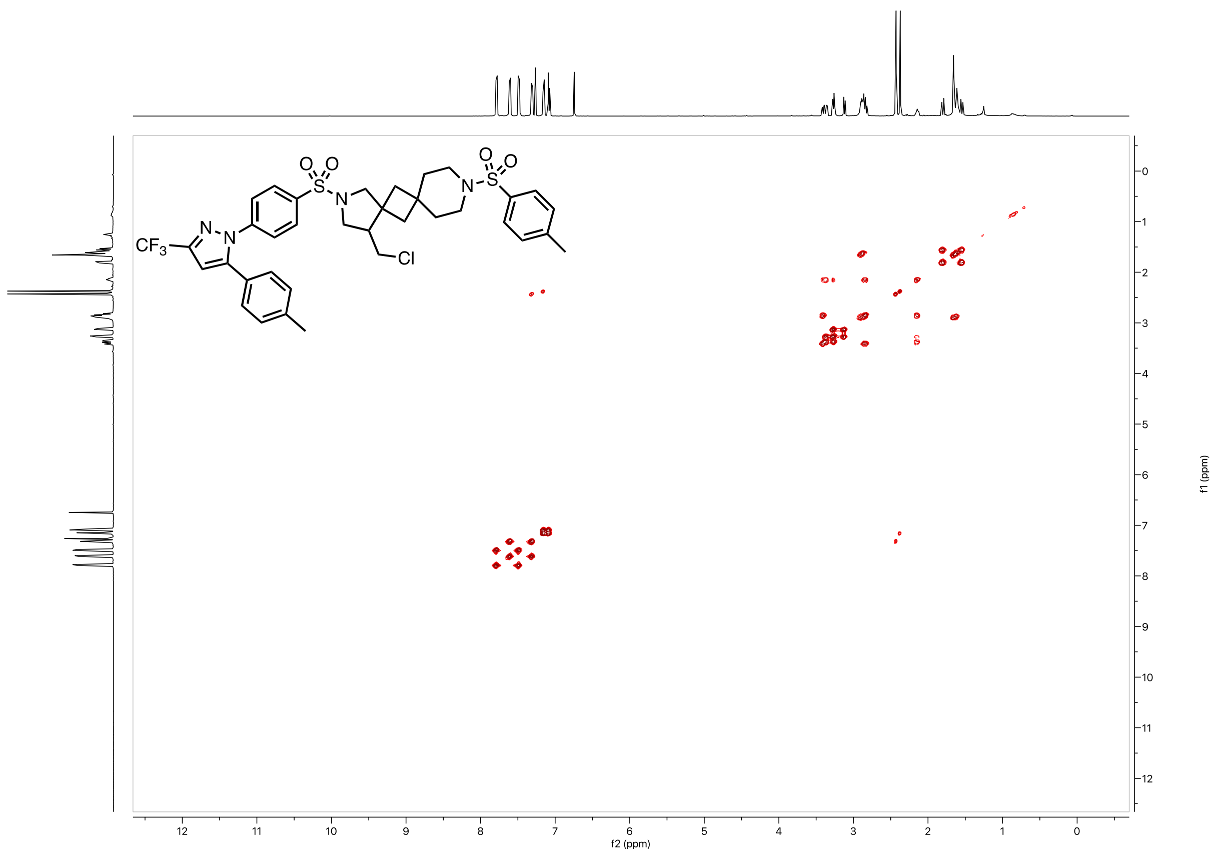

COSY of *4-(chloromethyl)-2-((4-(5-(p-tolyl)-3-(trifluoromethyl)-1H-pyrazol-1-yl)phenyl)sulfonyl)-10-tosyl-2,10-diazadispiro[4.1.5^{7}.1^{5}]tridecane* ($CDCl_3$).

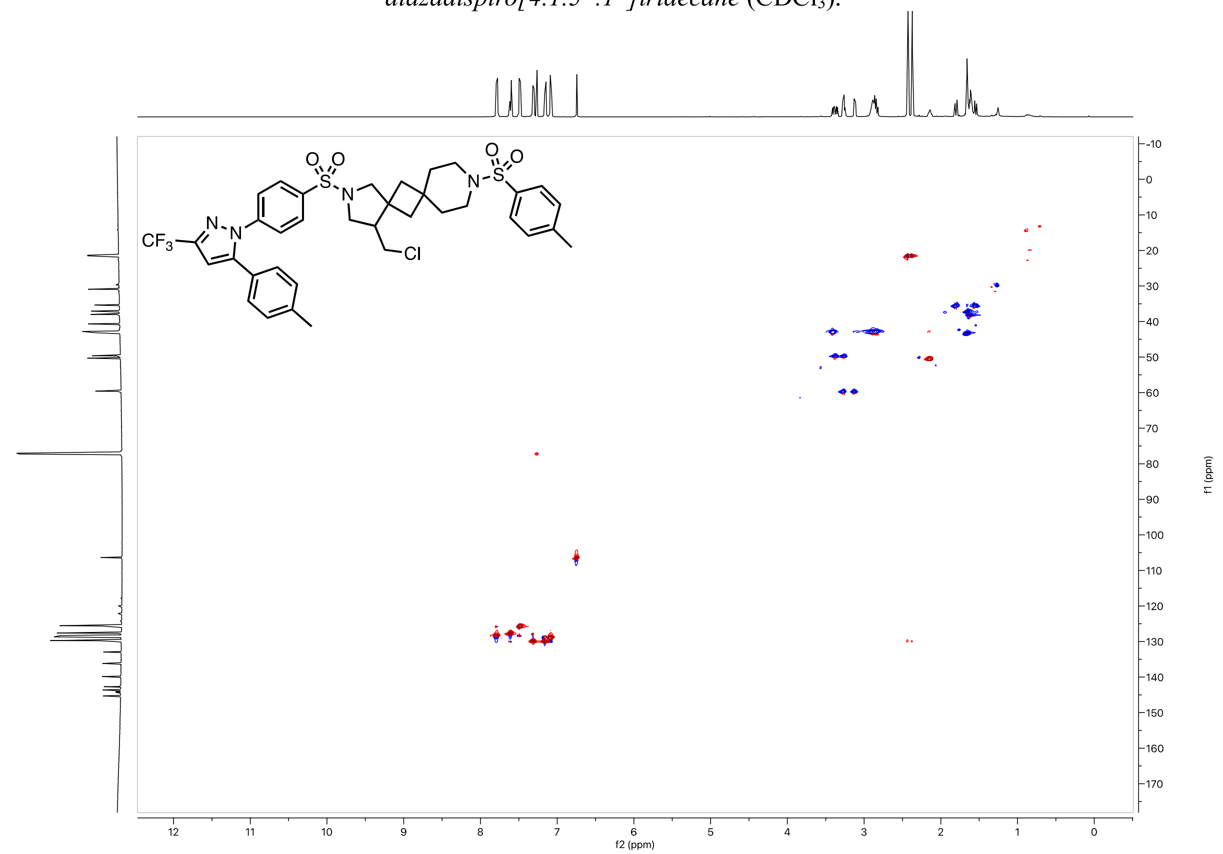

eHSQC of *4-(chloromethyl)-2-((4-(5-(p-tolyl)-3-(trifluoromethyl)-1H-pyrazol-1-yl)phenyl)sulfonyl)-10-tosyl-2,10-diazadispiro[4.1.5^{7}.1^{5}]tridecane* ($CDCl_3$).

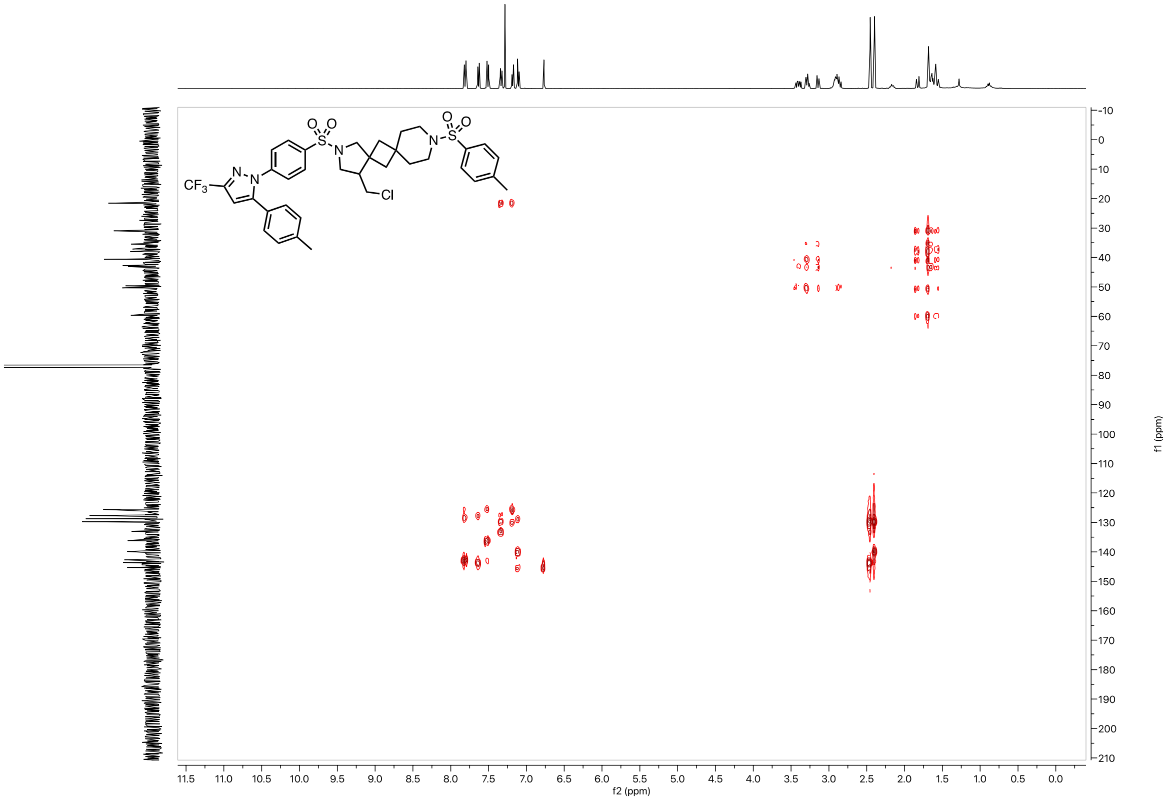

HMBC of *4-(chloromethyl)-2-((4-(5-(p-tolyl)-3-(trifluoromethyl)-1H-pyrazol-1-yl)phenyl)sulfonyl)-10-tosyl-2,10-diazadispiro[4.1.5^7.1^5]tridecane* ($CDCl_3$).

4-(chloromethyl)-2-((4-(5-(p-tolyl)-3-(trifluoromethyl)-1H-pyrazol-1-yl)phenyl)sulfonyl)-2-azaspiro[4.5]decane (3aj)

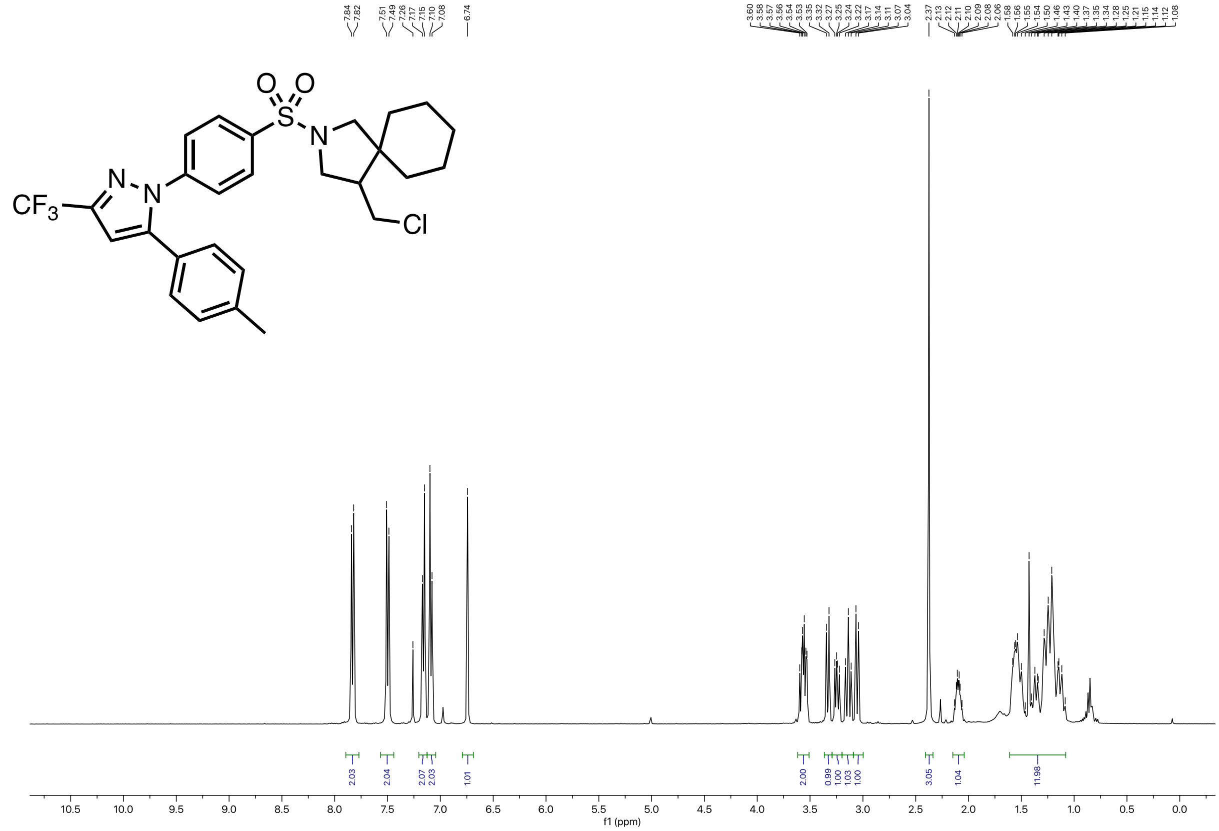

^{1}H NMR of *4-(chloromethyl)-2-((4-(5-(p-tolyl)-3-(trifluoromethyl)-1H-pyrazol-1-yl)phenyl)sulfonyl)-2-azaspiro[4.5]decane* (400 MHz, $CDCl_3$).

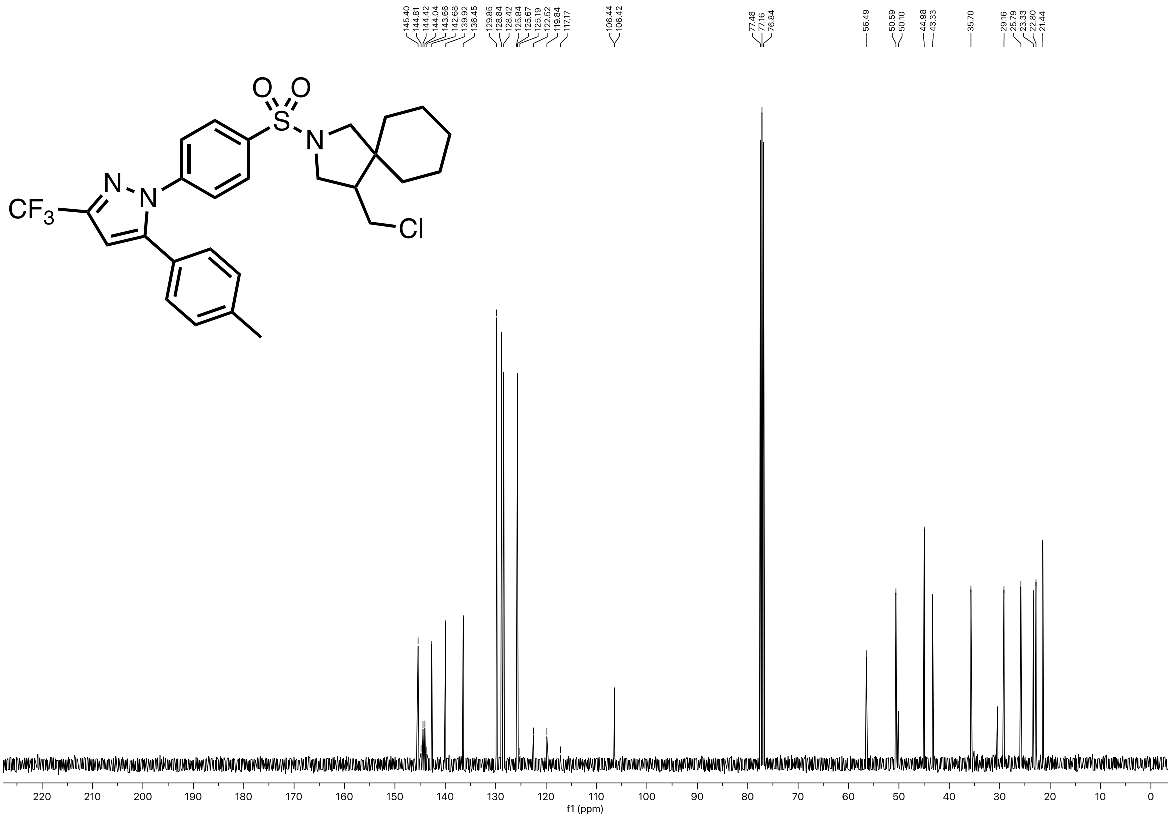

$^{13}C\{^{1}H\}$ NMR of *4-(chloromethyl)-2-((4-(5-(p-tolyl)-3-(trifluoromethyl)-1H-pyrazol-1-yl)phenyl)sulfonyl)-2-azaspiro[4.5]decane* (101 MHz, $CDCl_3$).

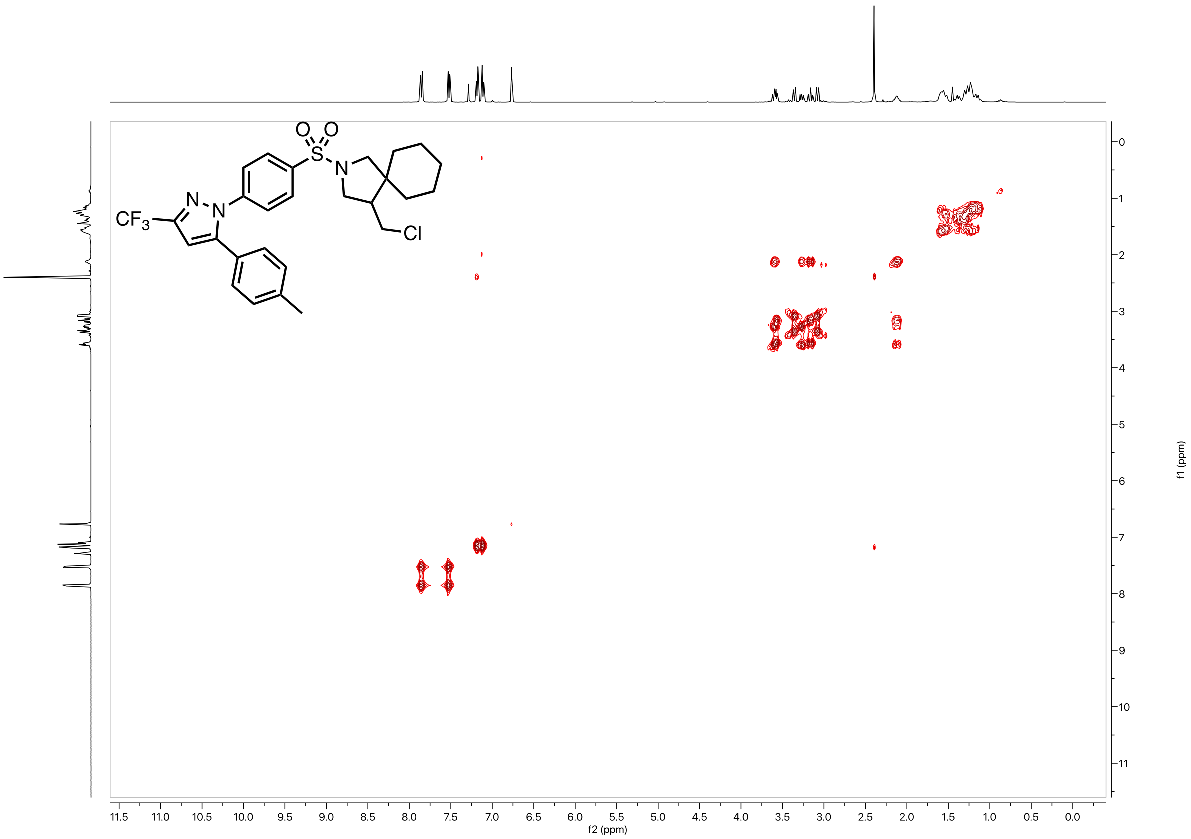

COSY of *4-(chloromethyl)-2-((4-(5-(p-tolyl)-3-(trifluoromethyl)-1H-pyrazol-1-yl)phenyl)sulfonyl)-2-azaspiro[4.5]decane* ($CDCl_3$).

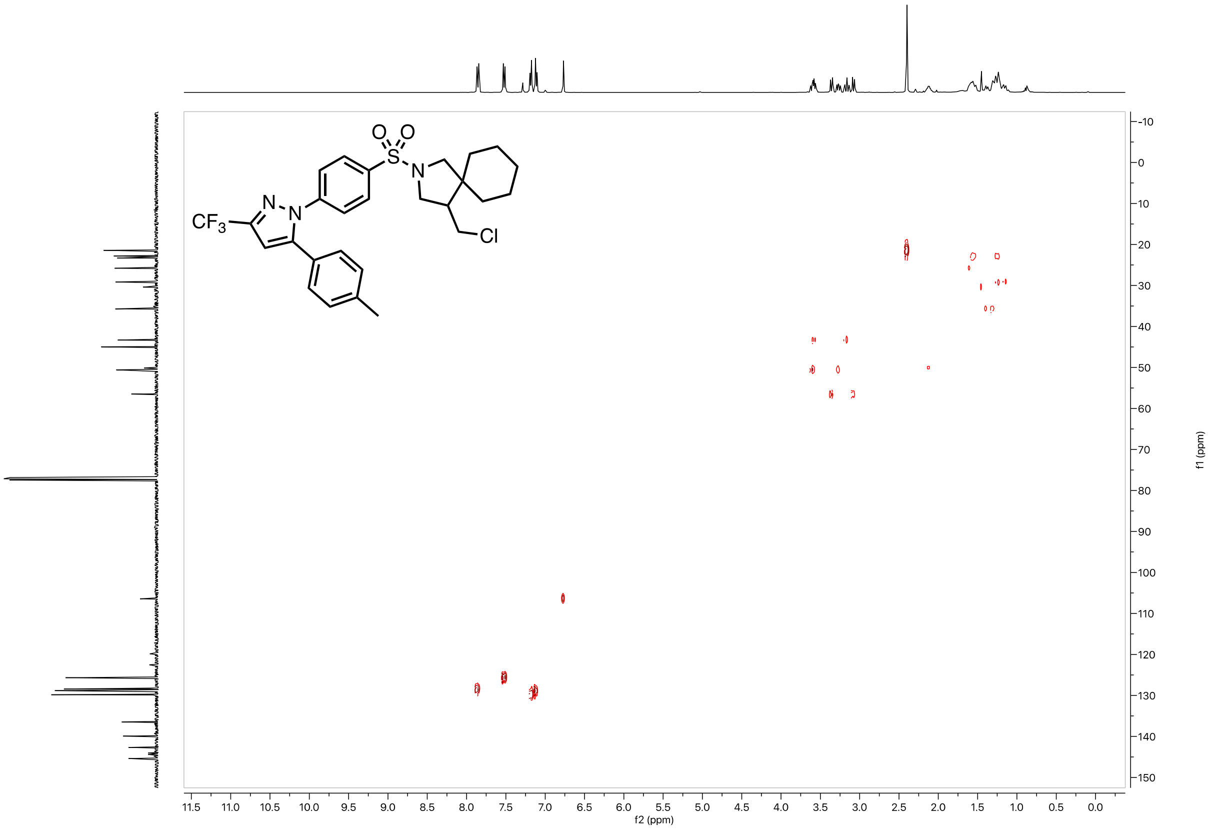

HSQC of *4-(chloromethyl)-2-((4-(5-(p-tolyl)-3-(trifluoromethyl)-1H-pyrazol-1-yl)phenyl)sulfonyl)-2-azaspiro[4.5]decane* ($CDCl_3$).

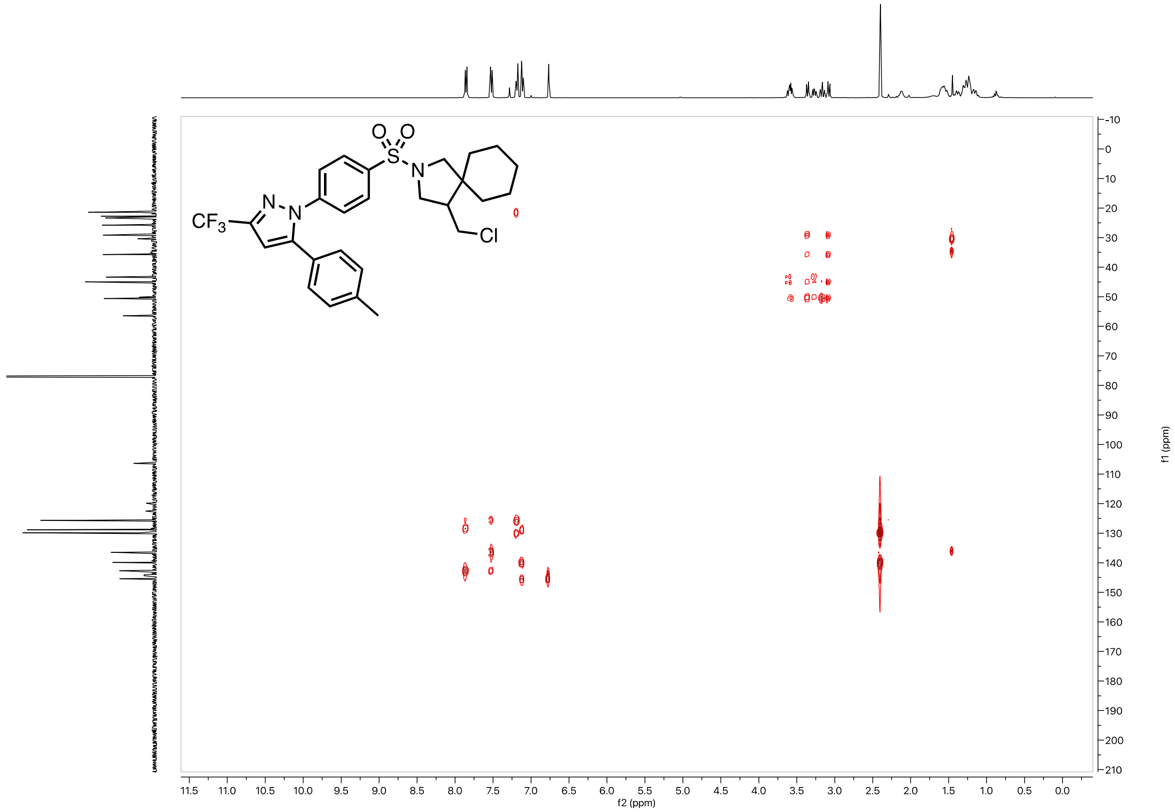

HMBC of *4-(chloromethyl)-2-((4-(5-(p-tolyl)-3-(trifluoromethyl)-1H-pyrazol-1-yl)phenyl)sulfonyl)-2-azaspiro[4.5]decane* ($CDCl_3$).

4-(4-((4-(chloromethyl)-2-azaspiro[4.5]decan-2-yl)sulfonyl)phenyl)-5-methyl-3-phenylisoxazole (3ak)

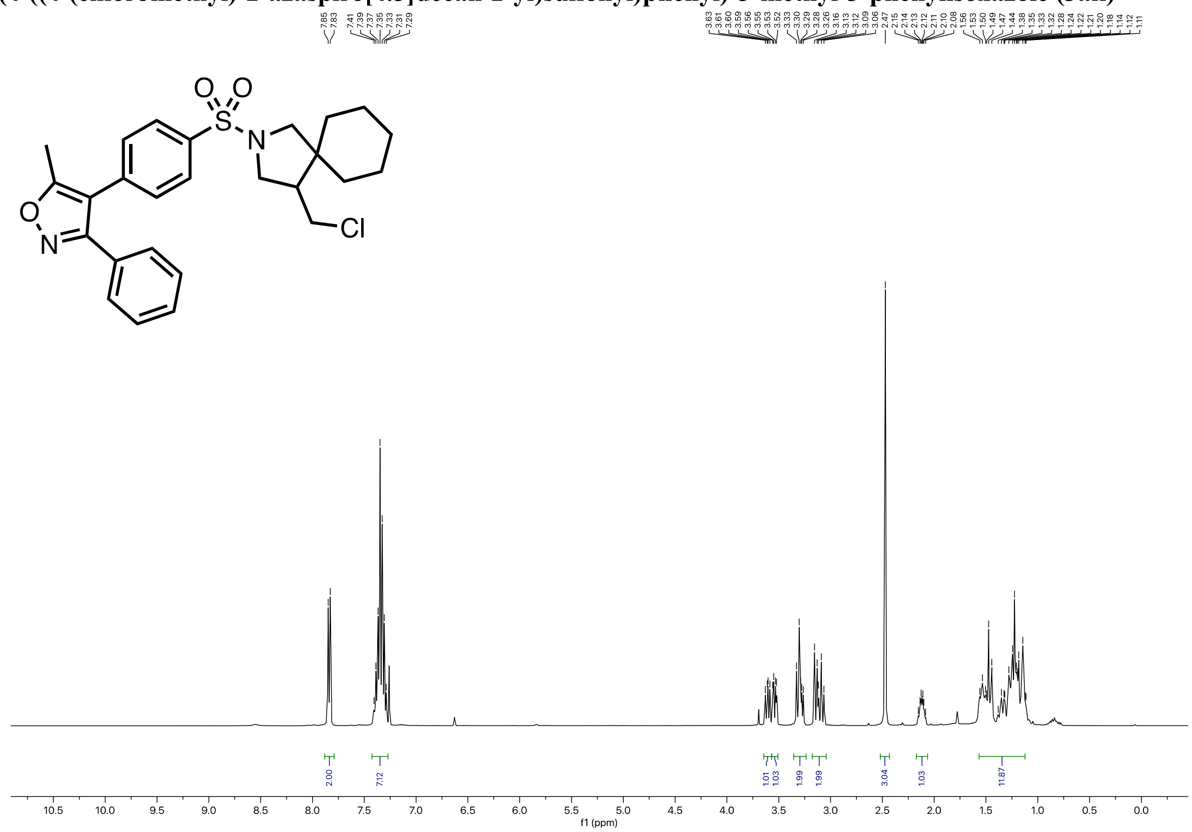

^{1}H NMR of *4-(4-((4-(chloromethyl)-2-azaspiro[4.5]decan-2-yl)sulfonyl)phenyl)-5-methyl-3-phenylisoxazole* (400 MHz, $CDCl_3$).

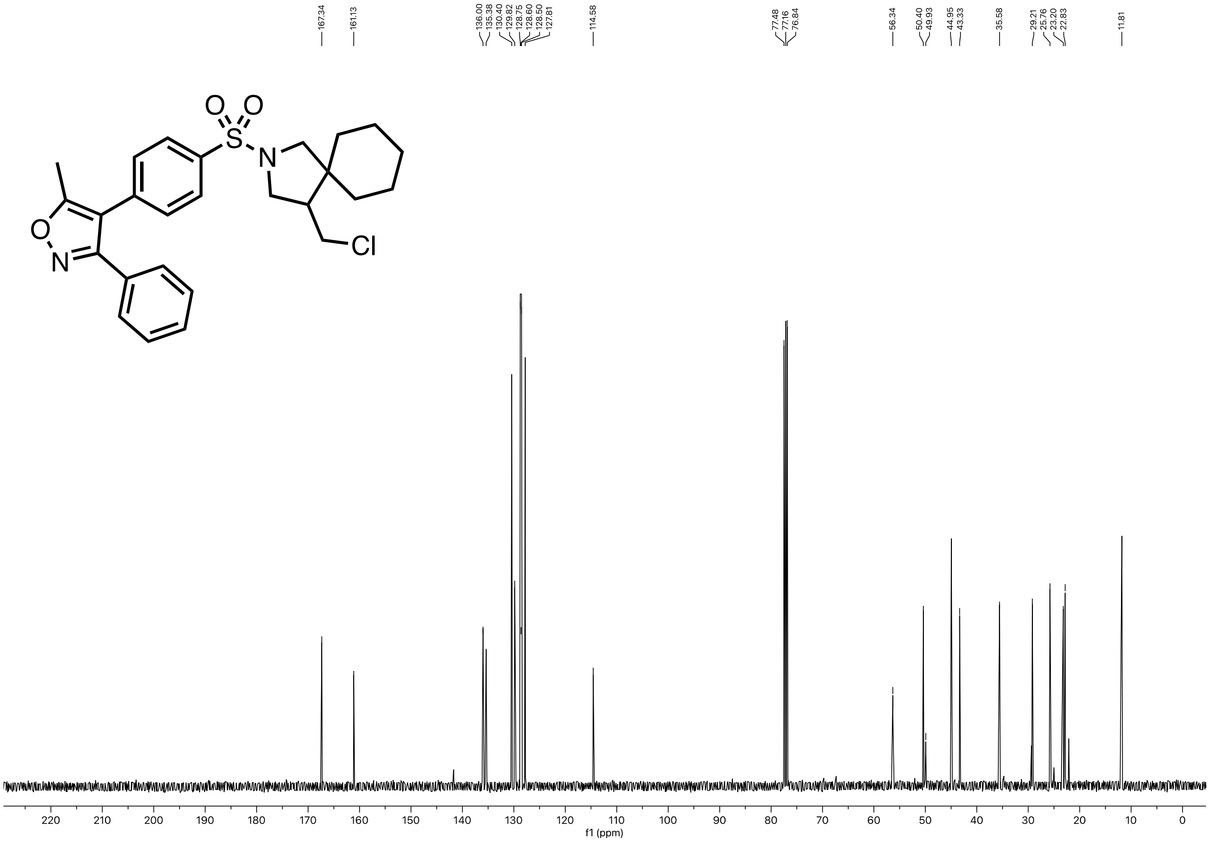

^{13}C{^{1}H} NMR of *4-(4-((4-(chloromethyl)-2-azaspiro[4.5]decan-2-yl)sulfonyl)phenyl)-5-methyl-3-phenylisoxazole* (101 MHz, $CDCl_3$).

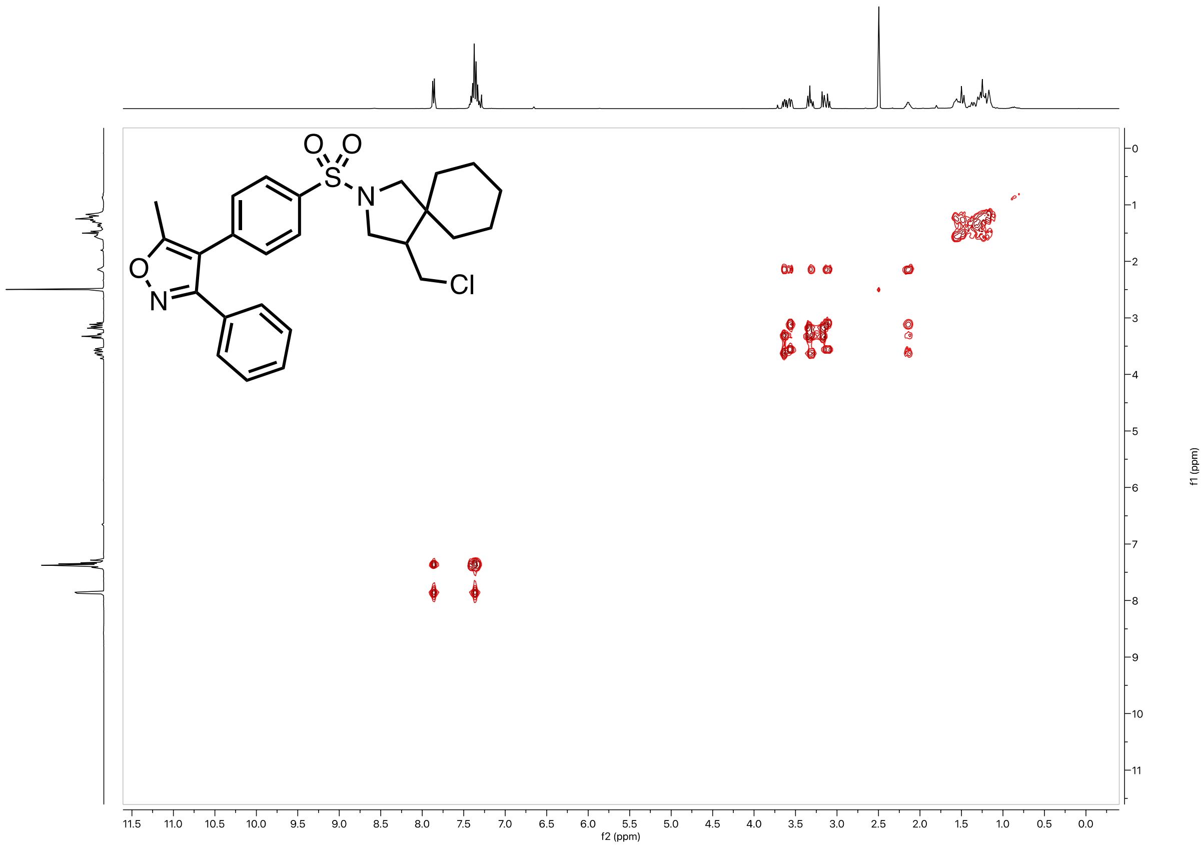

COSY of *4-(4-((4-(chloromethyl)-2-azaspiro[4.5]decan-2-yl)sulfonyl)phenyl)-5-methyl-3-phenylisoxazole* ($CDCl_3$).

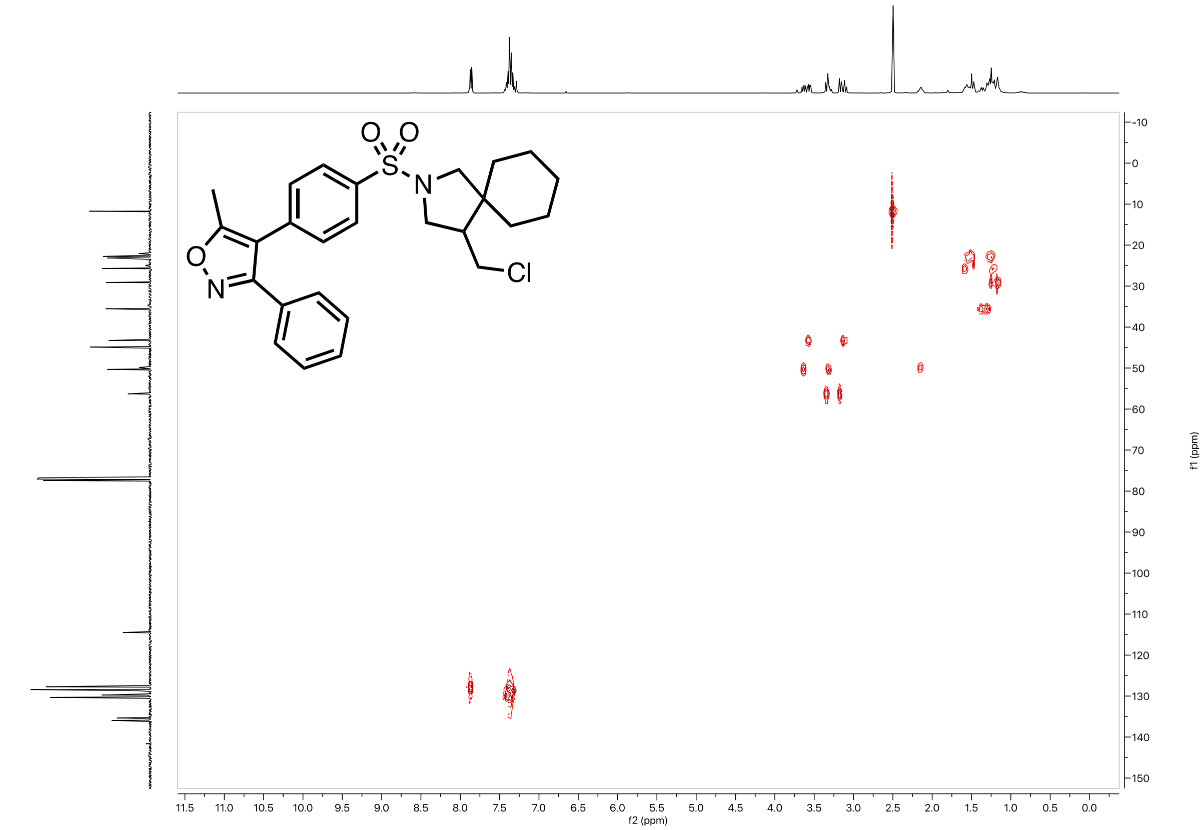

HSQC of *4-(4-((4-(chloromethyl)-2-azaspiro[4.5]decan-2-yl)sulfonyl)phenyl)-5-methyl-3-phenylisoxazole* ($CDCl_3$).

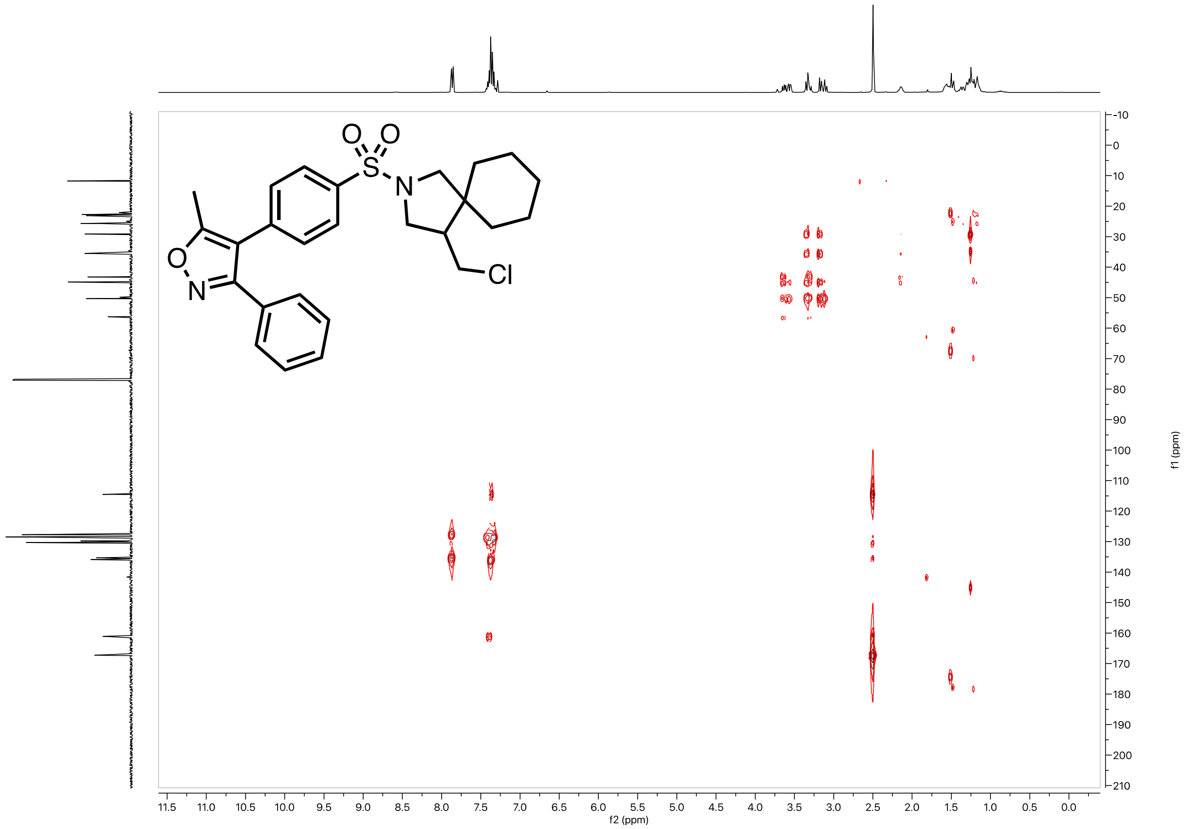

HMBC of *4-(4-((4-(chloromethyl)-2-azaspiro[4.5]decan-2-yl)sulfonyl)phenyl)-5-methyl-3-phenylisoxazole* ($CDCl_3$).

N-(5-((4-(chloromethyl)-2-azaspiro[4.5]decan-2-yl)sulfonyl)-3-methyl-1,3,4-thiadiazol-2(3H)-ylidene)acetamide (3al)

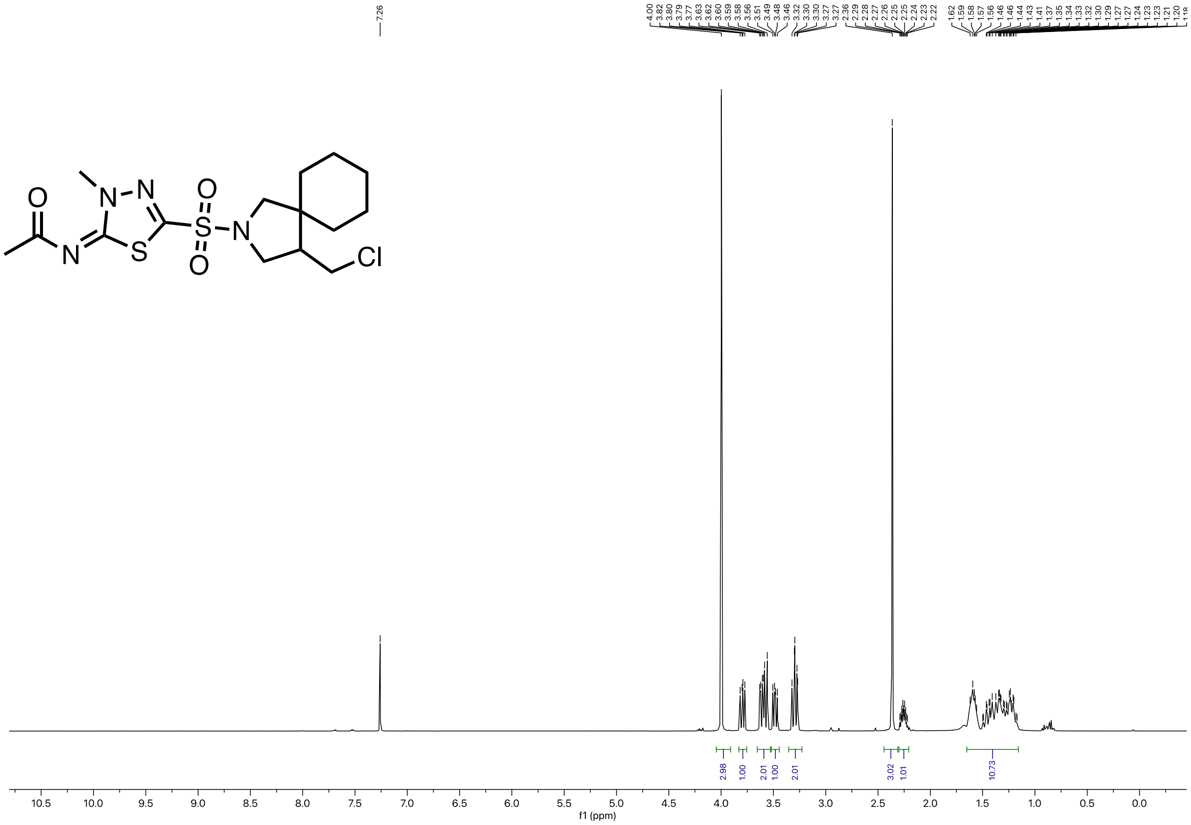

^{1}H NMR of *N-(5-((4-(chloromethyl)-2-azaspiro[4.5]decan-2-yl)sulfonyl)-3-methyl-1,3,4-thiadiazol-2(3H)-ylidene)acetamide* (400 MHz, $CDCl_3$).

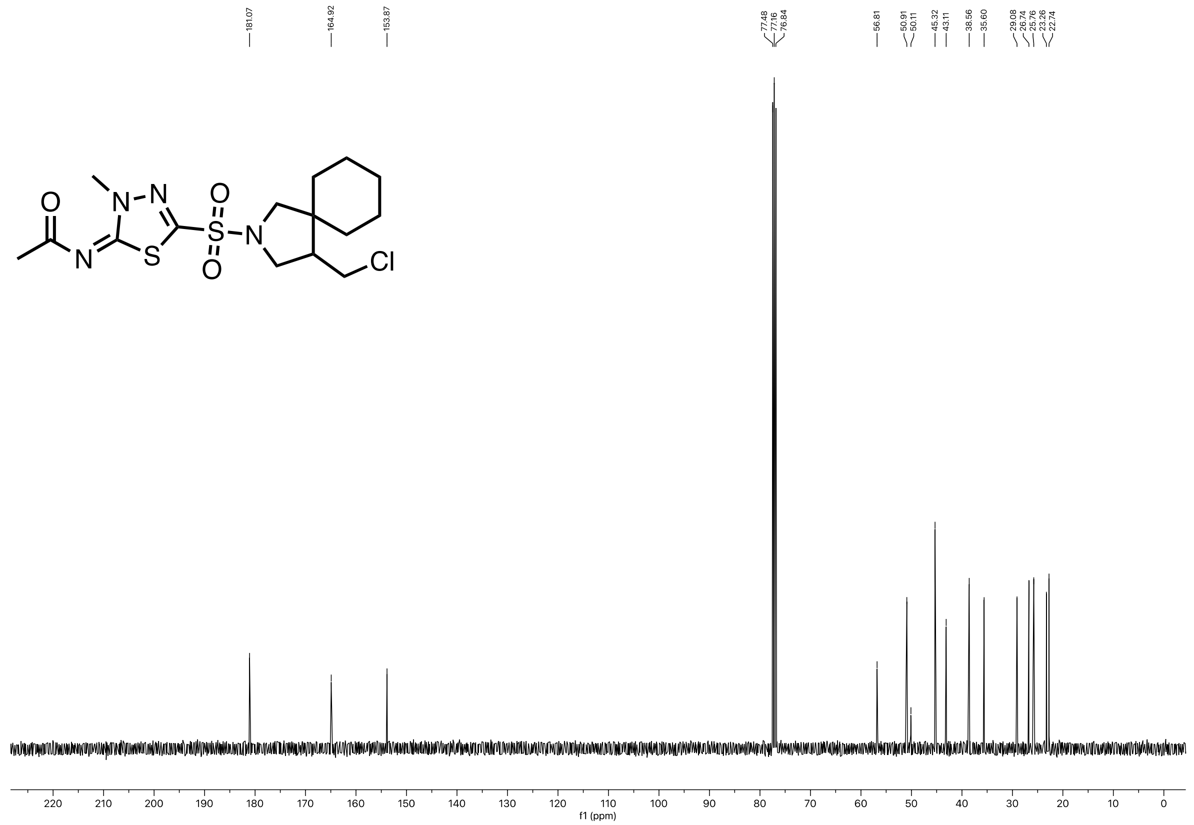

$^{13}C\{^{1}H\}$ NMR of *N-(5-((4-(chloromethyl)-2-azaspiro[4.5]decan-2-yl)sulfonyl)-3-methyl-1,3,4-thiadiazol-2(3H)-ylidene)acetamide* (101 MHz, $CDCl_3$).

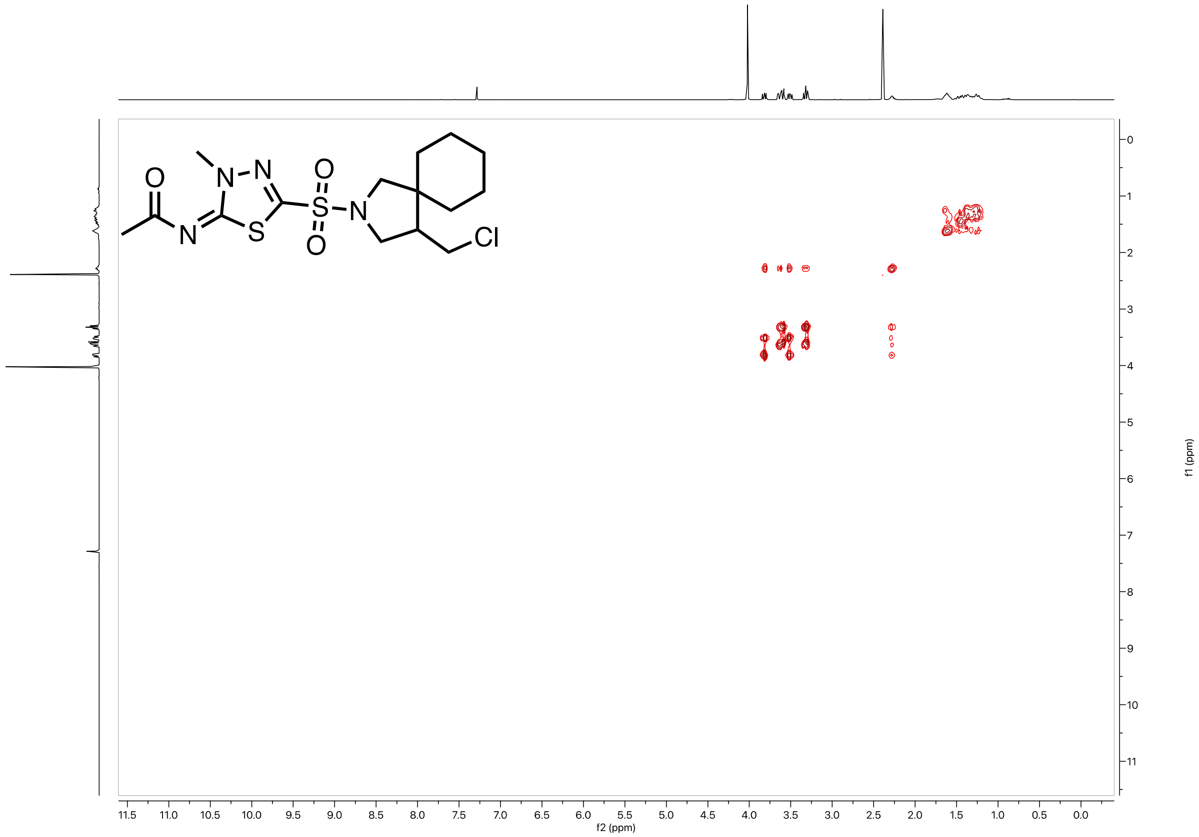

COSY of *N-(5-((4-(chloromethyl)-2-azaspiro[4.5]decan-2-yl)sulfonyl)-3-methyl-1,3,4-thiadiazol-2(3H)-ylidene)acetamide* ($CDCl_3$).

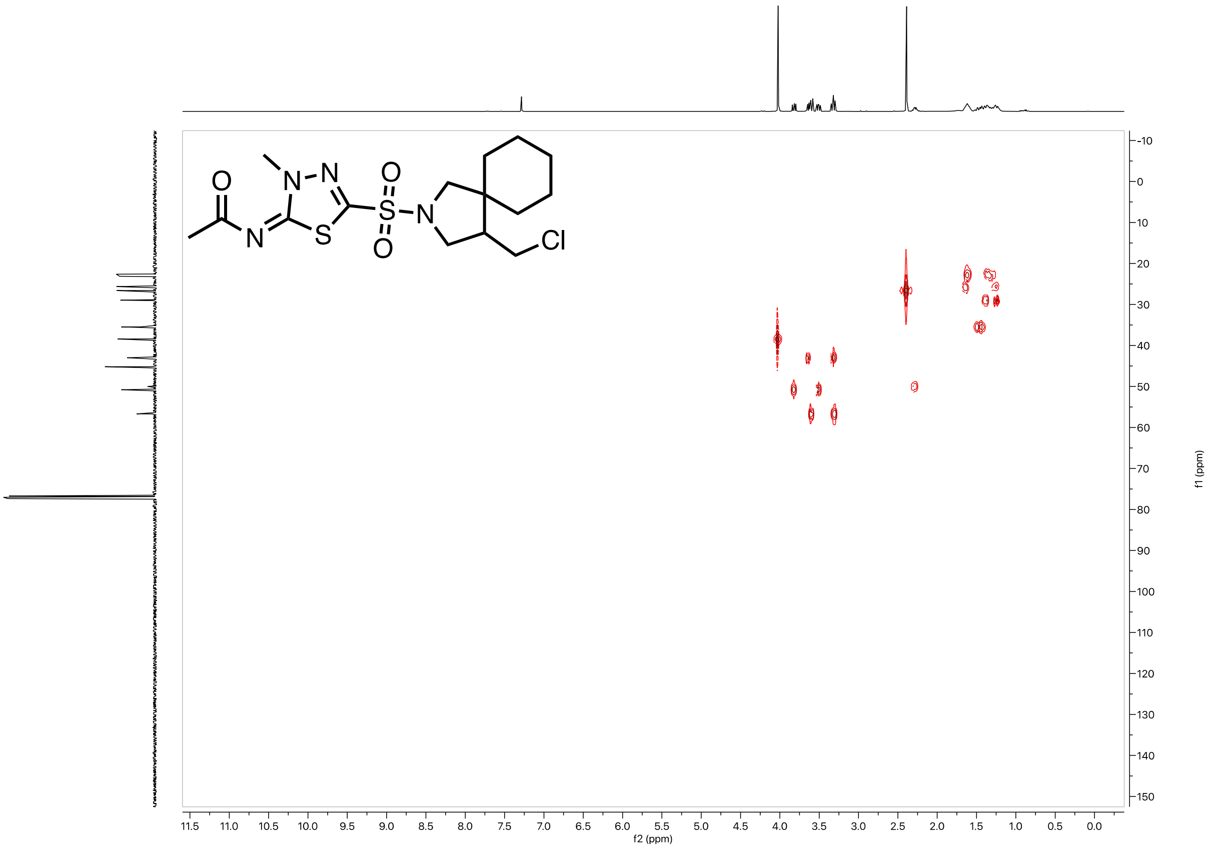

HSQC of *N-(5-((4-(chloromethyl)-2-azaspiro[4.5]decan-2-yl)sulfonyl)-3-methyl-1,3,4-thiadiazol-2(3H)-ylidene)acetamide* ($CDCl_3$).

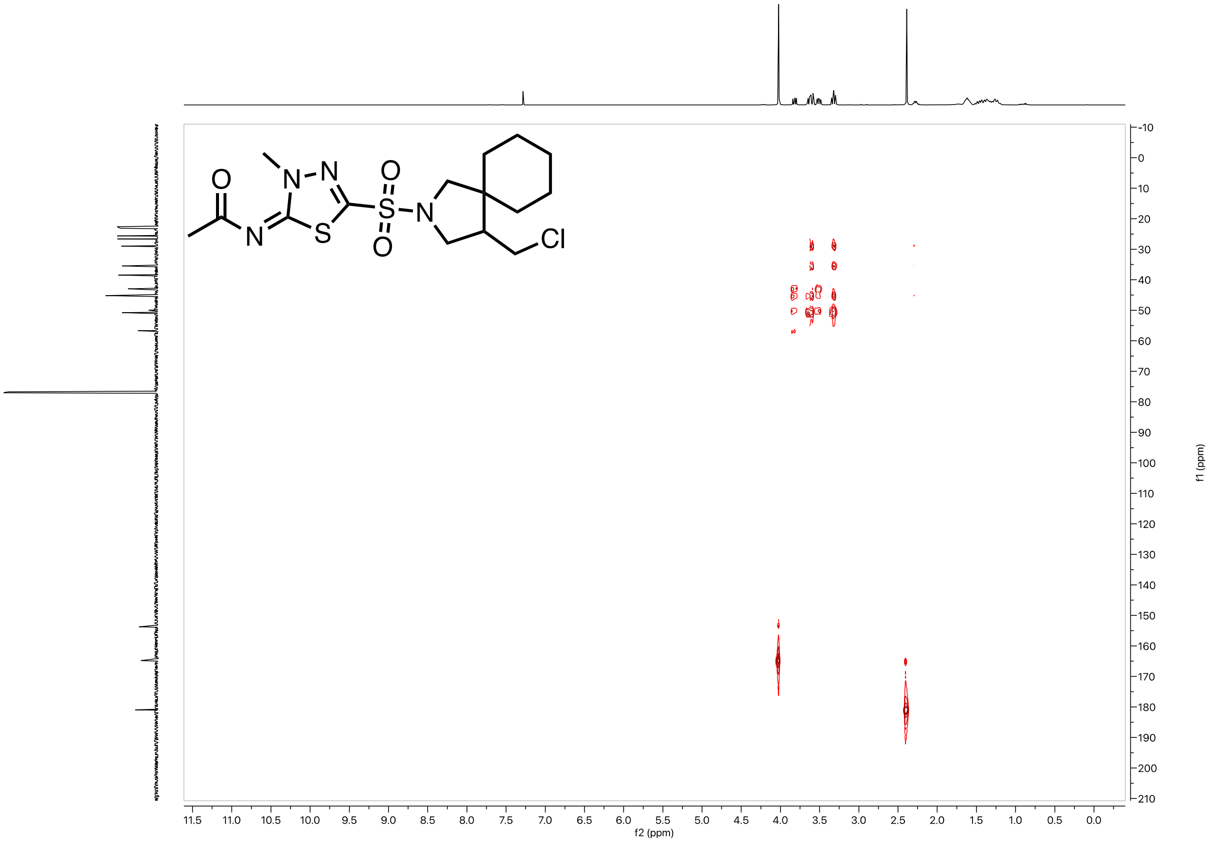

HMBC of *N-(5-((4-(chloromethyl)-2-azaspiro[4.5]decan-2-yl)sulfonyl)-3-methyl-1,3,4-thiadiazol-2(3H)-ylidene)acetamide* ($CDCl_3$).

2-((8-(chloromethyl)-6-azaspiro[3.4]octan-6-yl)sulfonyl)-6-ethoxybenzo[*d*]thiazole (3am)

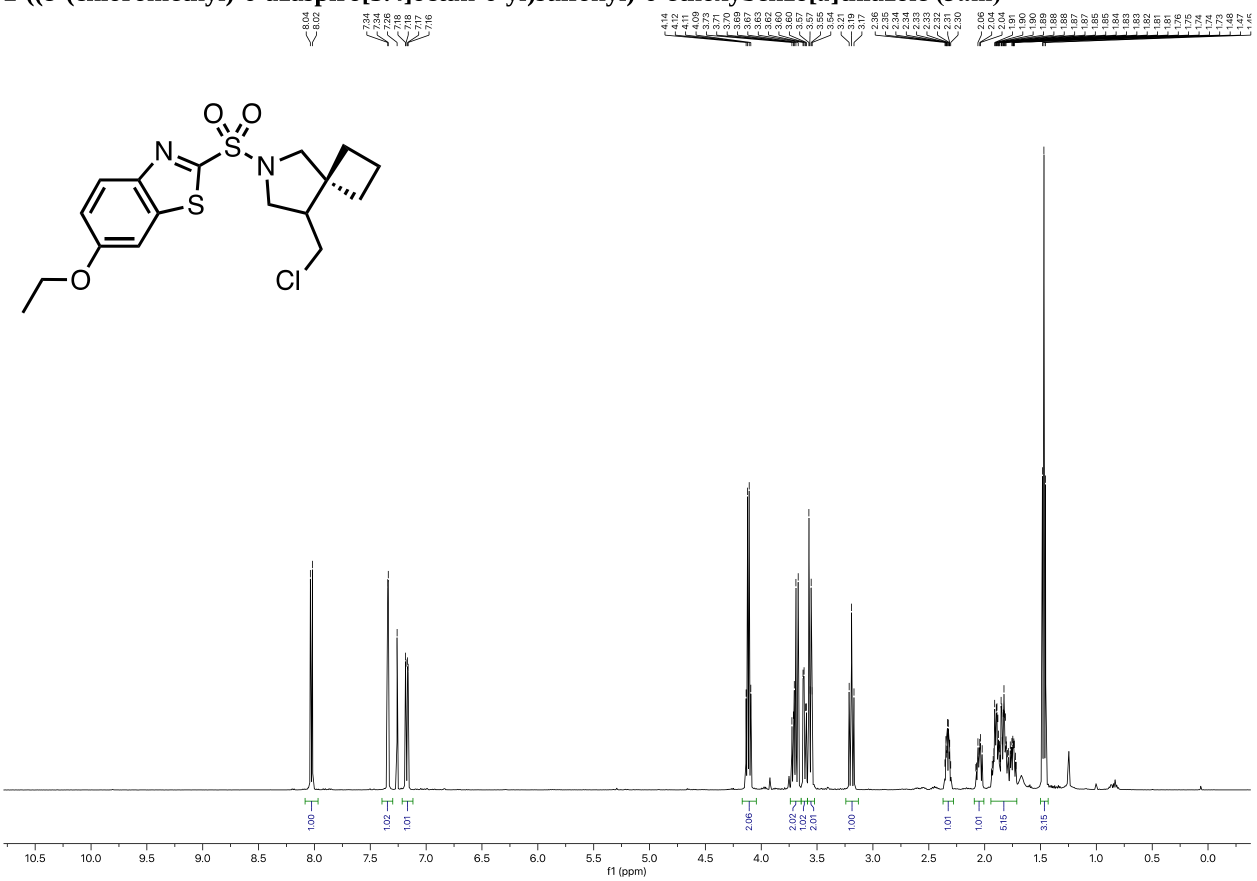

^{1}H NMR of *2-((8-(chloromethyl)-6-azaspiro[3.4]octan-6-yl)sulfonyl)-6-ethoxybenzo[d]thiazole* (500 MHz, $CDCl_3$).

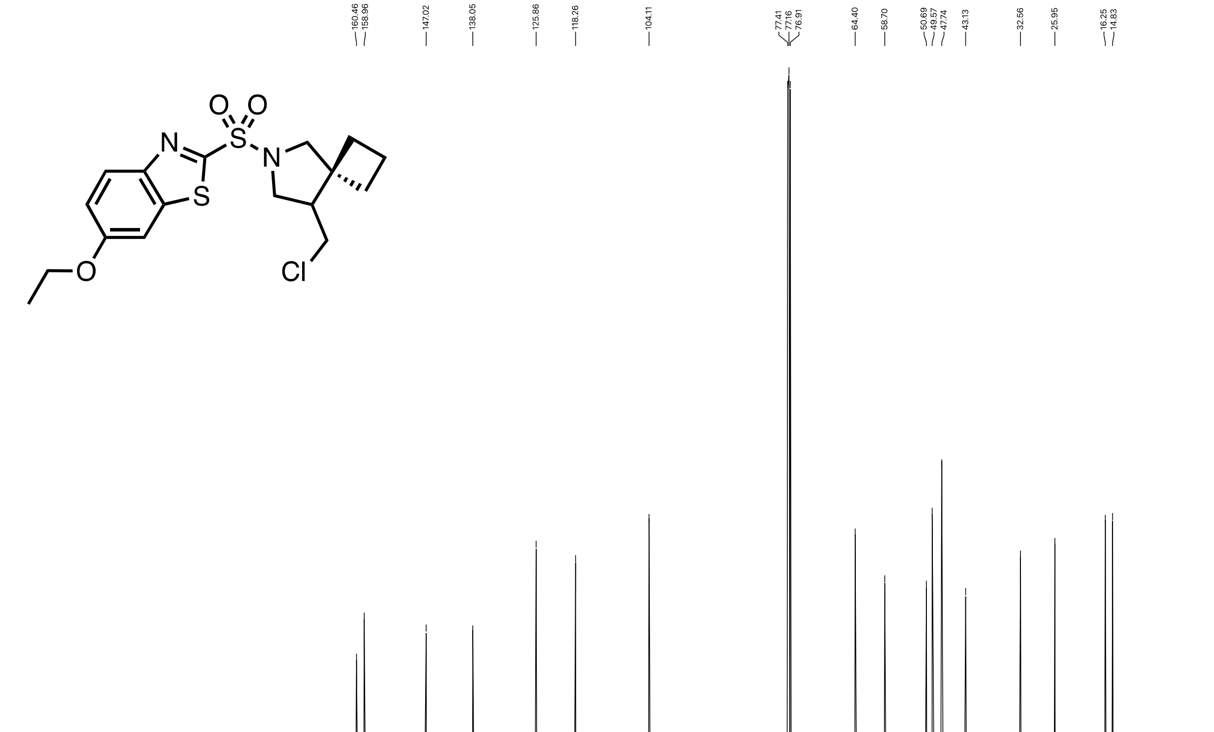

^{13}C{^{1}H} NMR of *2-((8-(chloromethyl)-6-azaspiro[3.4]octan-6-yl)sulfonyl)-6-ethoxybenzo[d]thiazole* (126 MHz, $CDCl_3$).

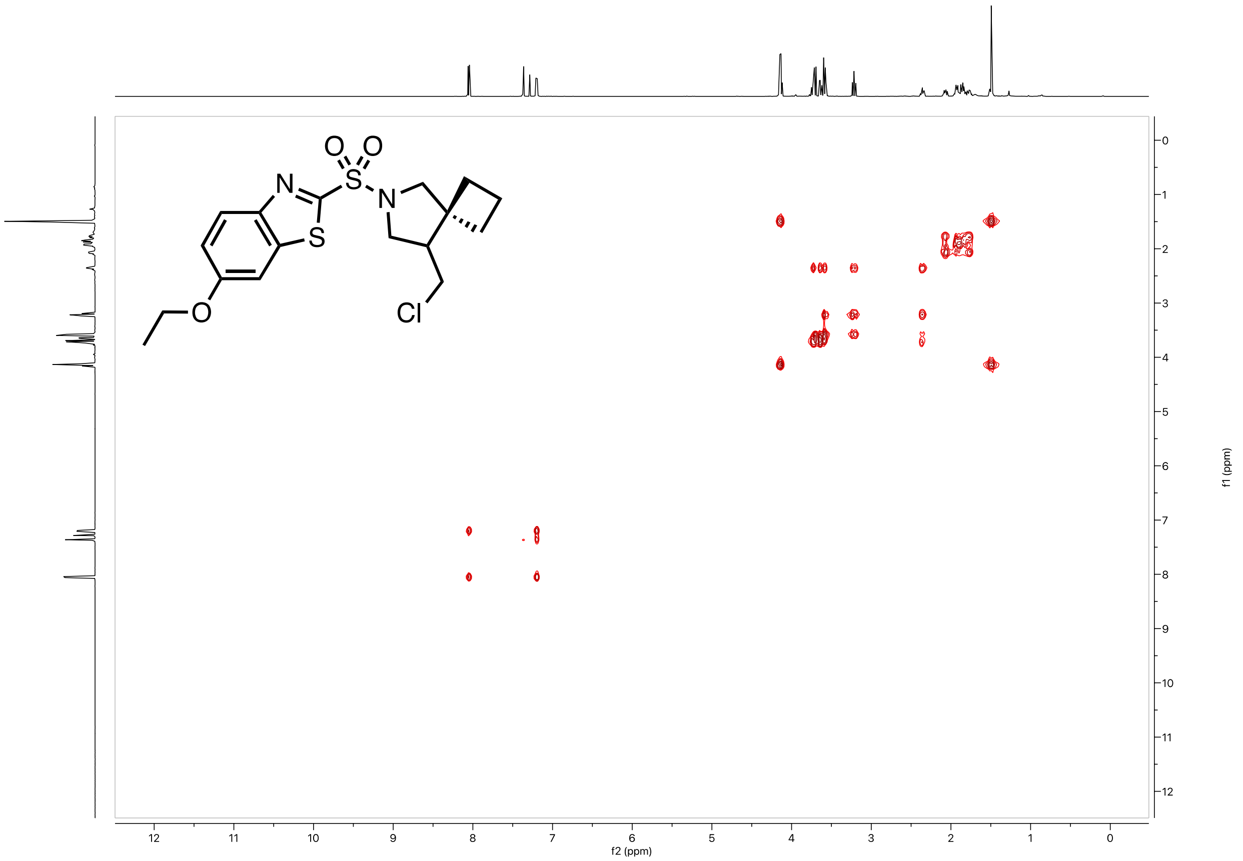

COSY of *2-((8-(chloromethyl)-6-azaspiro[3.4]octan-6-yl)sulfonyl)-6-ethoxybenzo[d]thiazole* ($CDCl_3$).

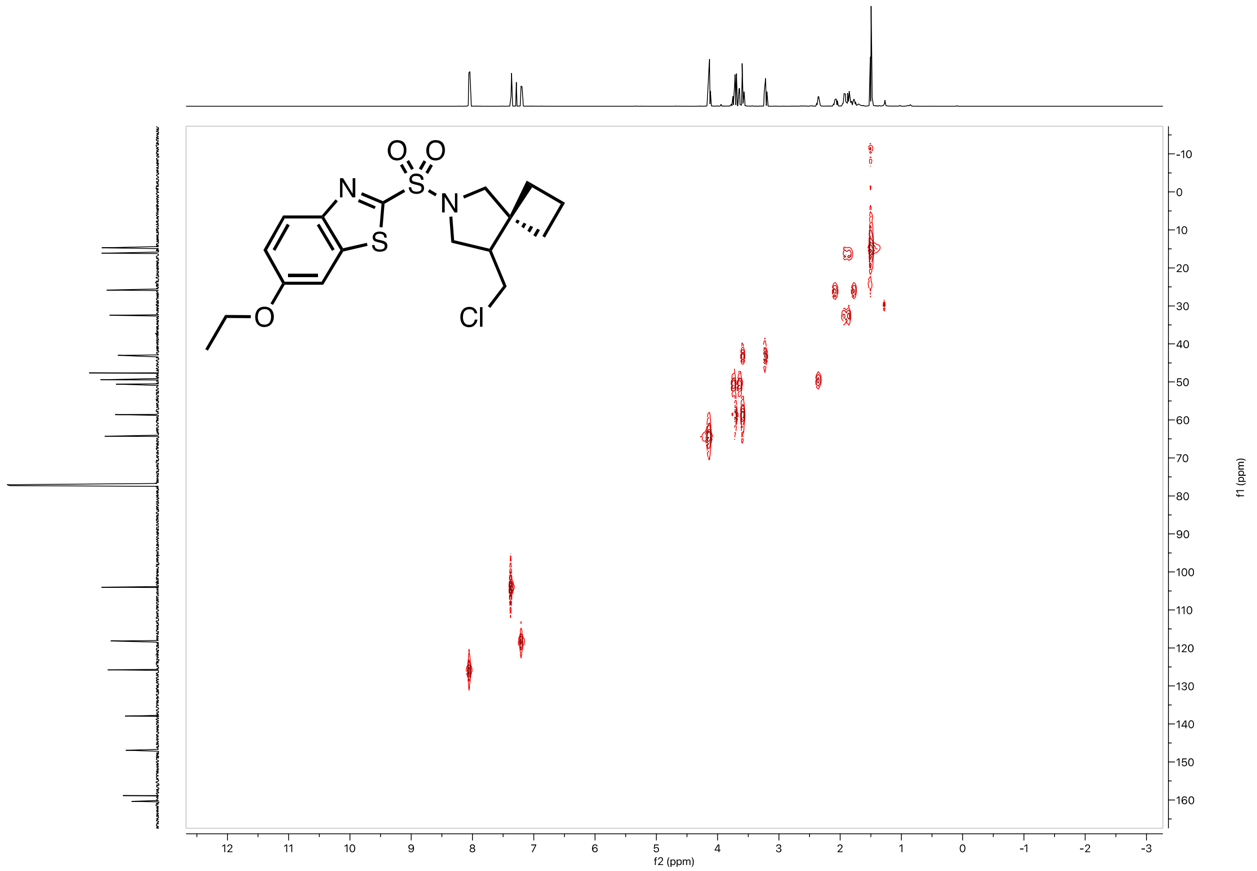

HSQC of *2-((8-(chloromethyl)-6-azaspiro[3.4]octan-6-yl)sulfonyl)-6-ethoxybenzo[d]thiazole* ($CDCl_3$).

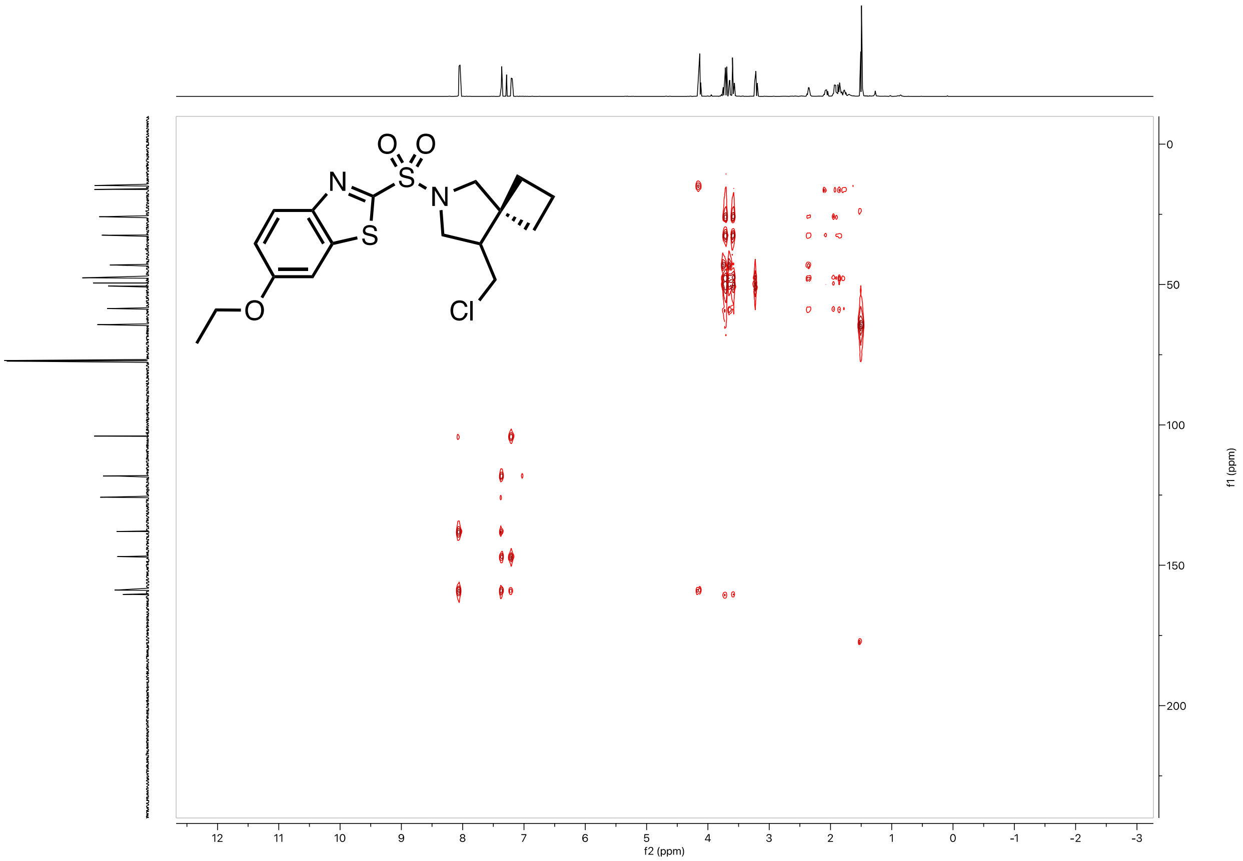

HMBC of *2-((8-(chloromethyl)-6-azaspiro[3.4]octan-6-yl)sulfonyl)-6-ethoxybenzo[d]thiazole* ($CDCl_3$).

4-methyl-2-tosyl-2-azaspiro[4.5]decane (4a)

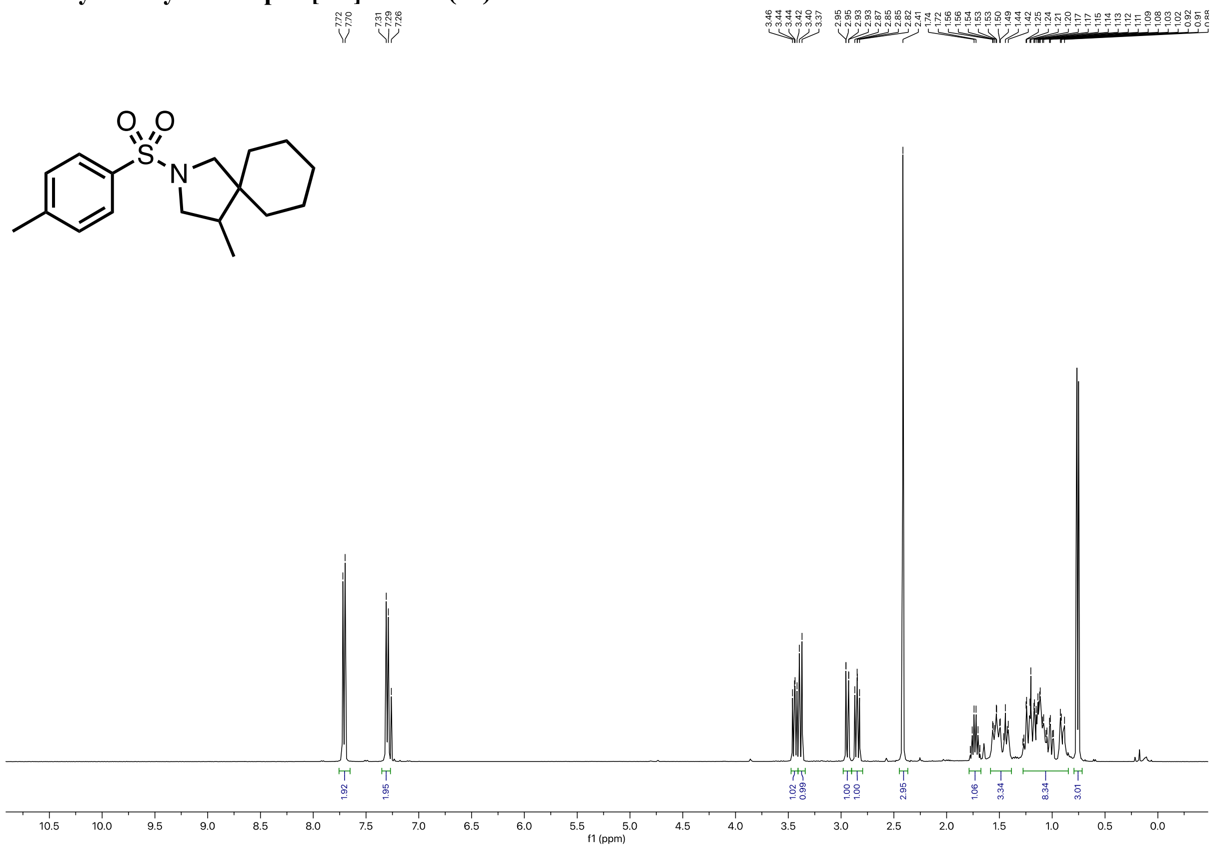

^{1}H NMR of *4-methyl-2-tosyl-2-azaspiro[4.5]decane* (400 MHz, $CDCl_3$).

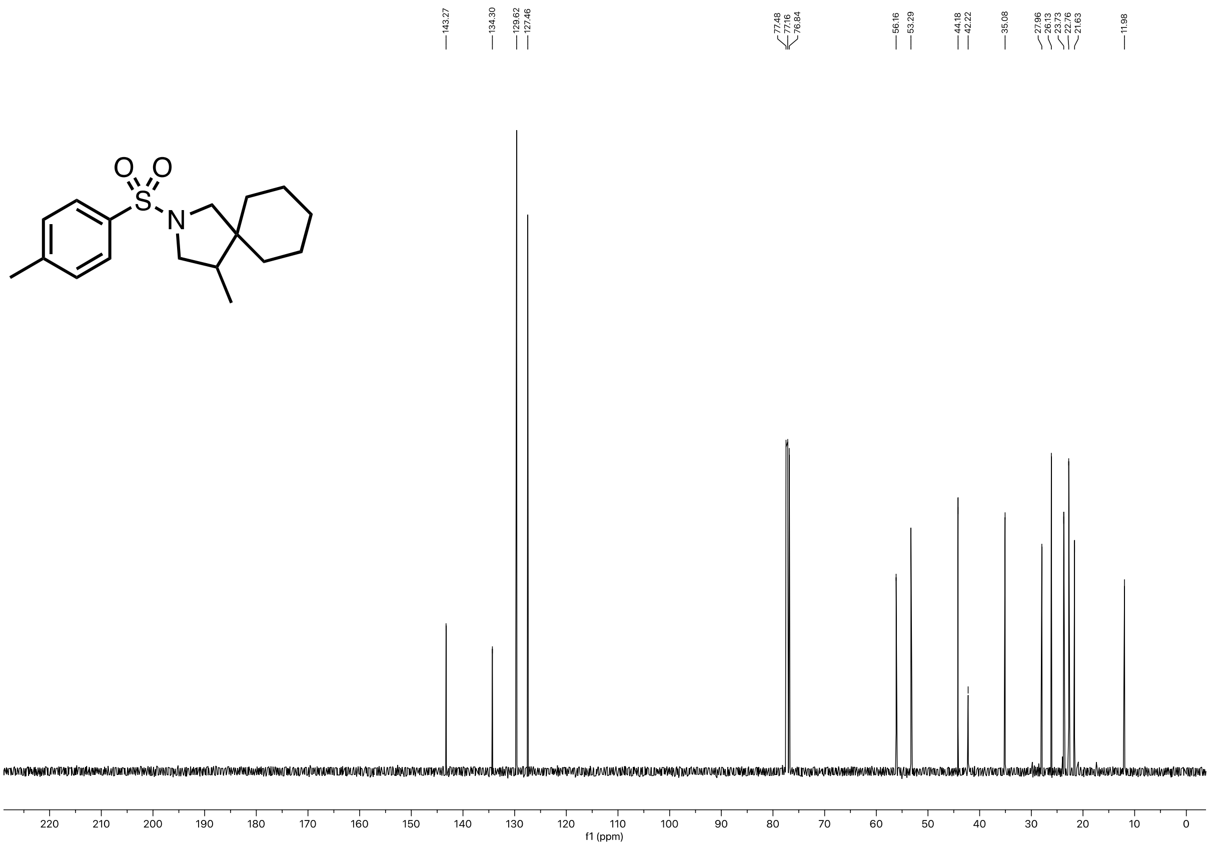

$^{13}C\{^{1}H\}$ NMR of *4-methyl-2-tosyl-2-azaspiro[4.5]decane* (101 MHz, $CDCl_3$).

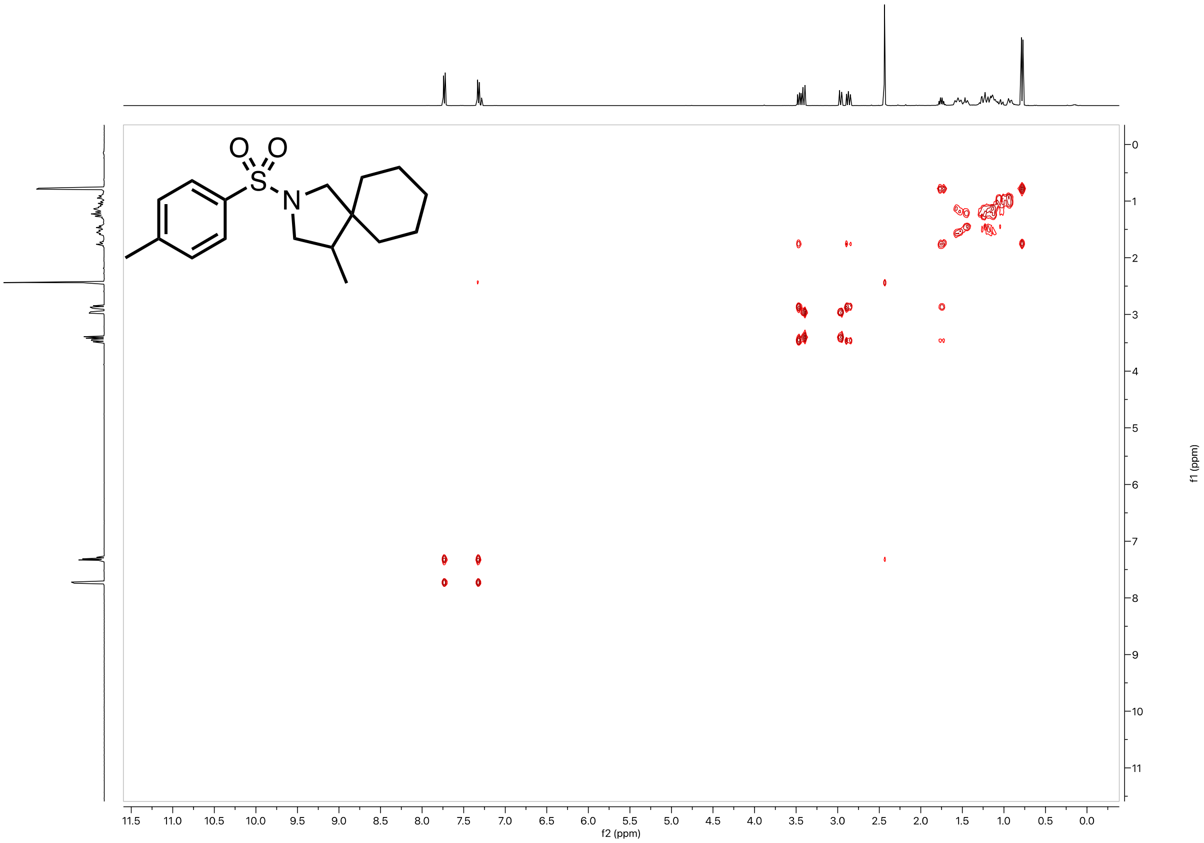

COSY of *4-methyl-2-tosyl-2-azaspiro[4.5]decane* ($CDCl_3$).

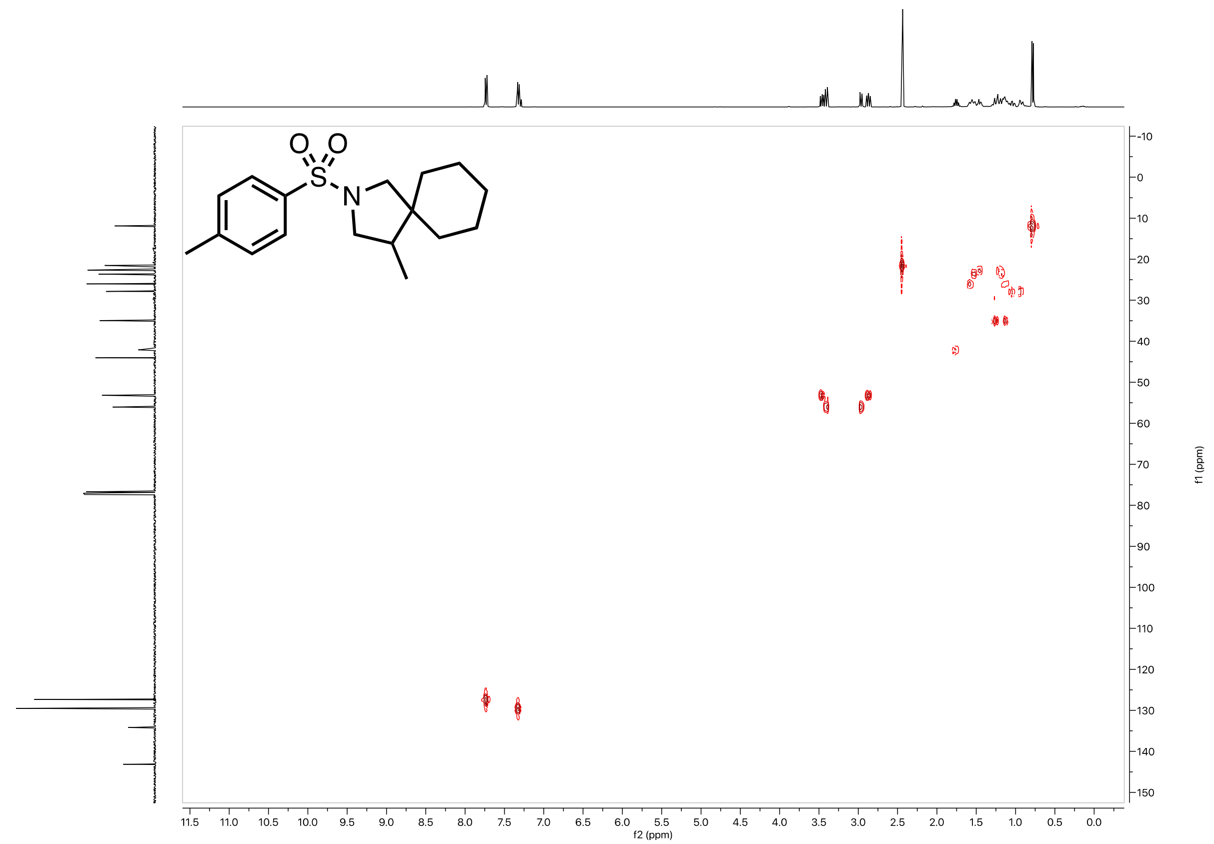

HSQC of *4-methyl-2-tosyl-2-azaspiro[4.5]decane* ($CDCl_3$).

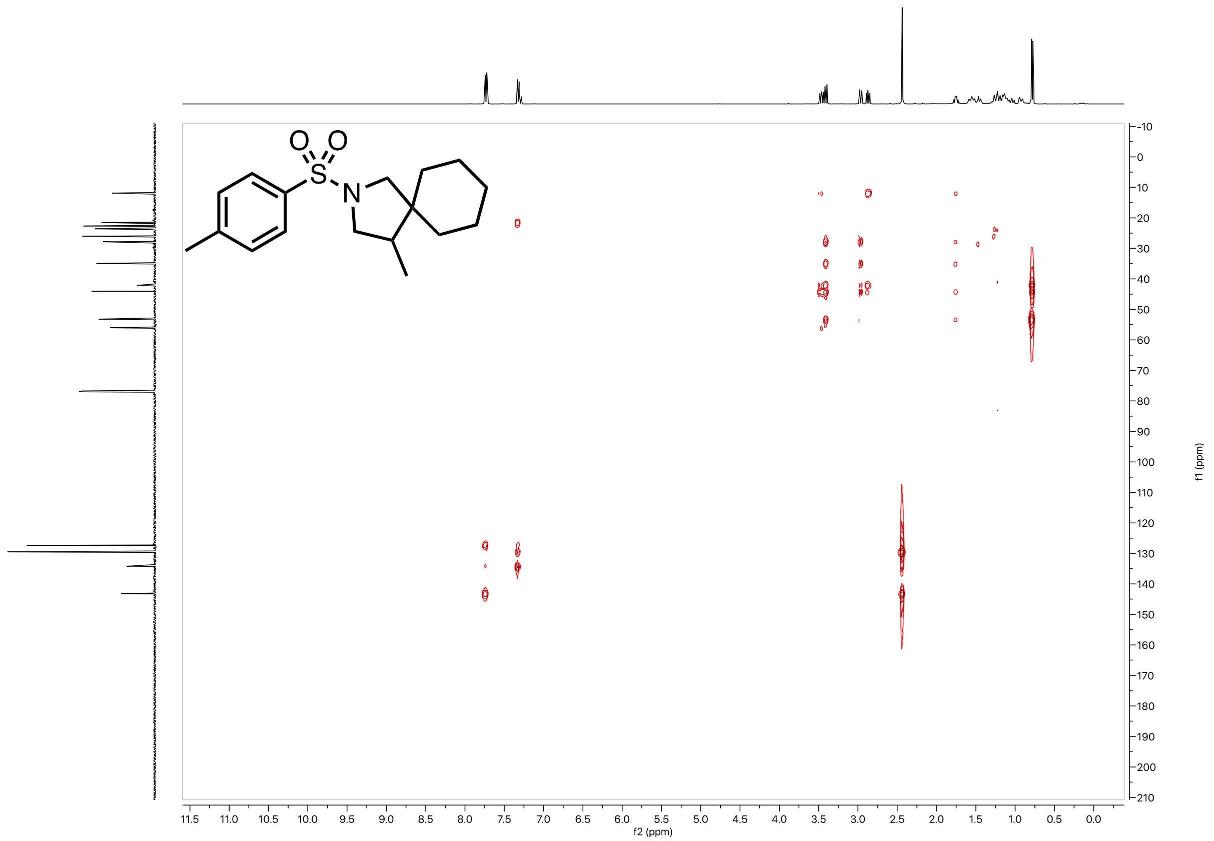

HMBC of *4-methyl-2-tosyl-2-azaspiro[4.5]decane* ($CDCl_3$).

4-methyl-2,8-ditosyl-2,8-diazaspiro[4.5]decane (4b)

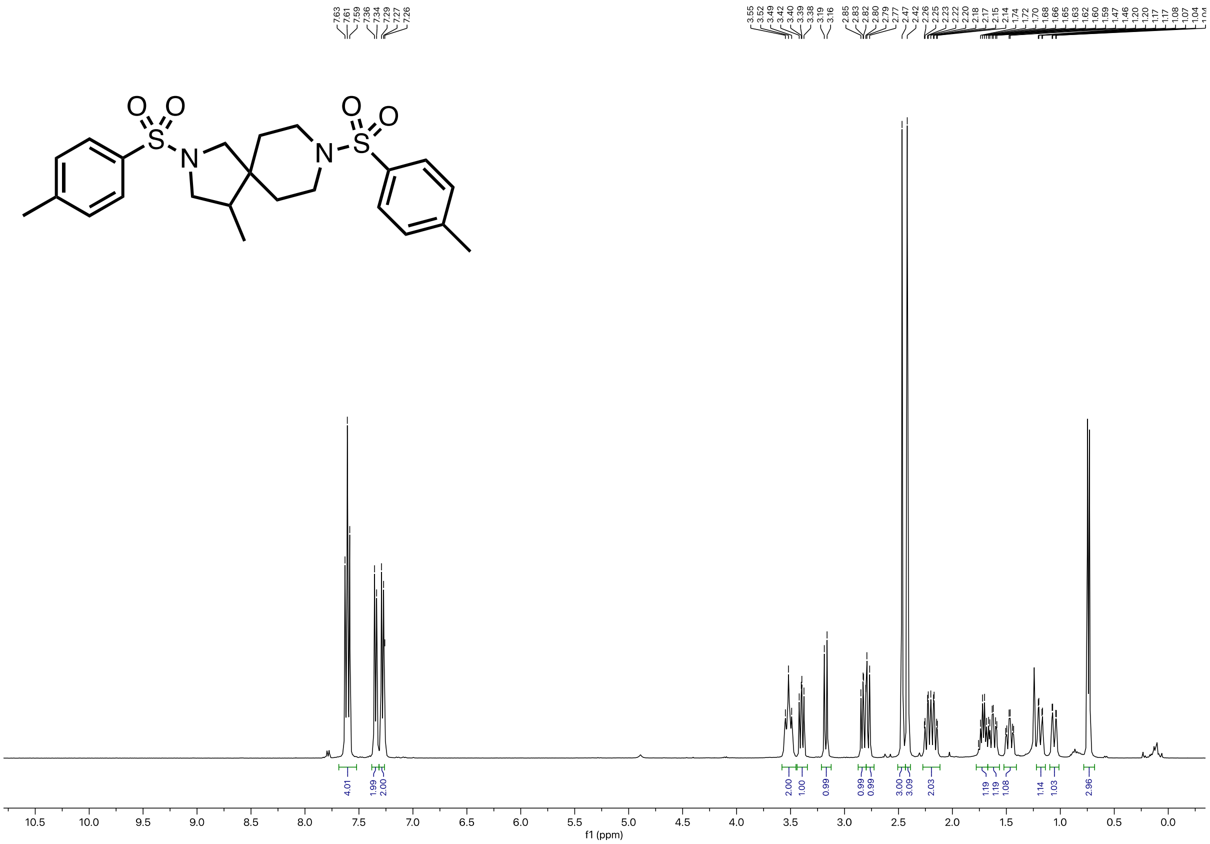

^{1}H NMR of *4-methyl-2,8-ditosyl-2,8-diazaspiro[4.5]decane* (400 MHz, $CDCl_3$).

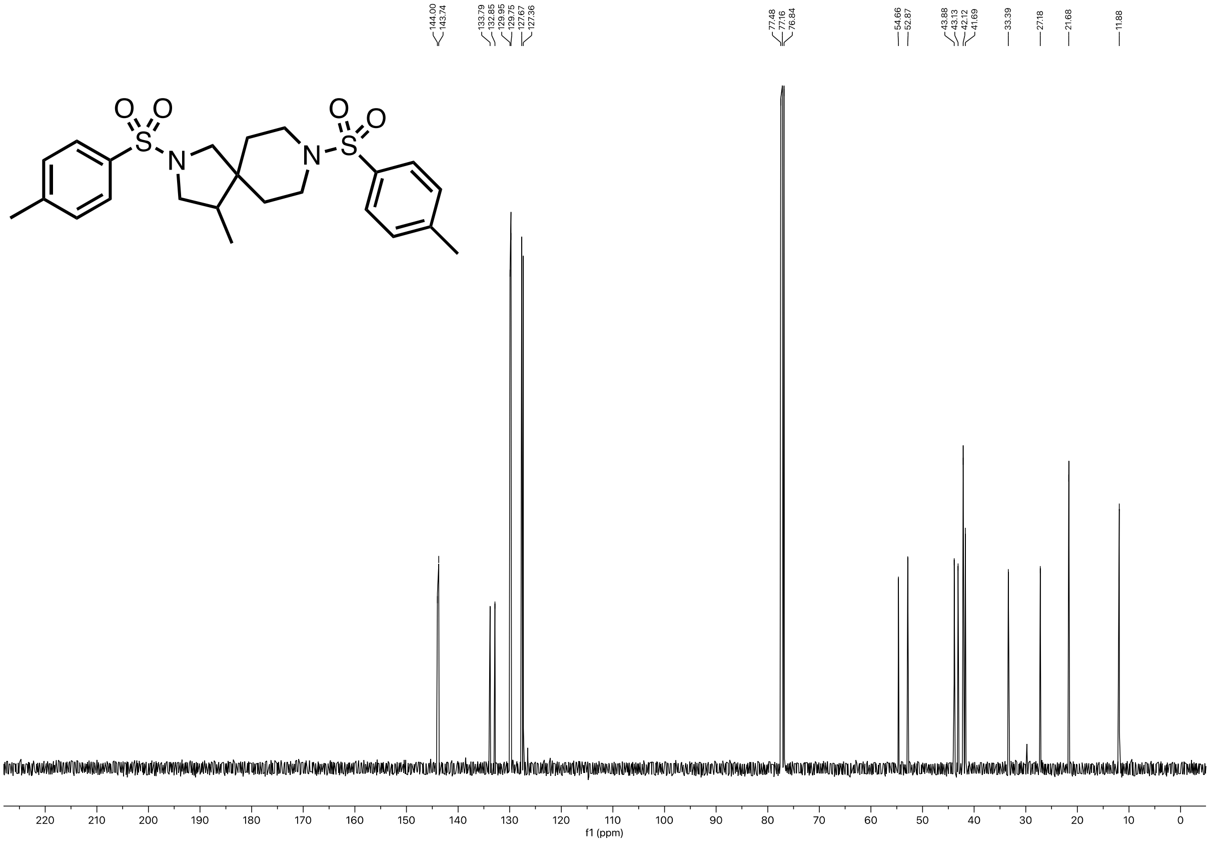

$^{13}C\{^{1}H\}$ NMR of *4-methyl-2,8-ditosyl-2,8-diazaspiro[4.5]decane* (101 MHz, $CDCl_3$).

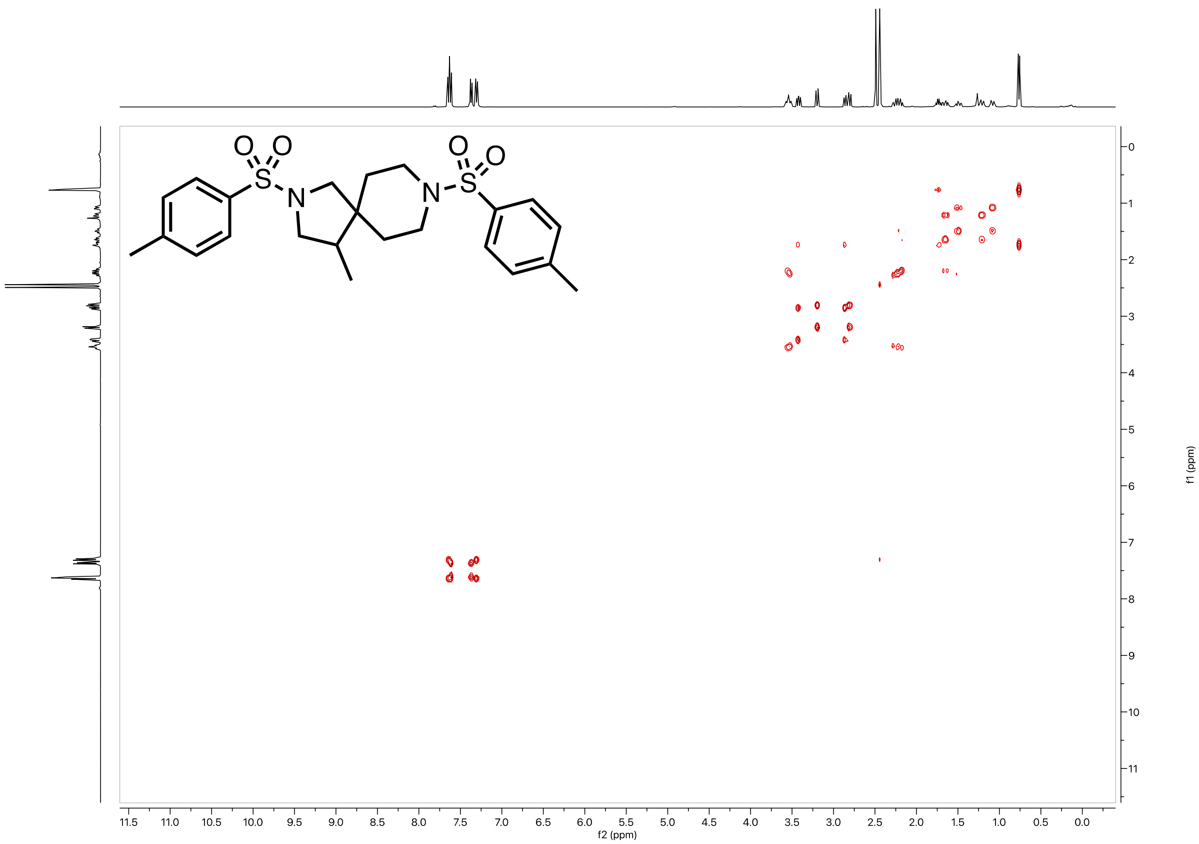

COSY of *4-methyl-2,8-ditosyl-2,8-diazaspiro[4.5]decane* ($CDCl_3$).

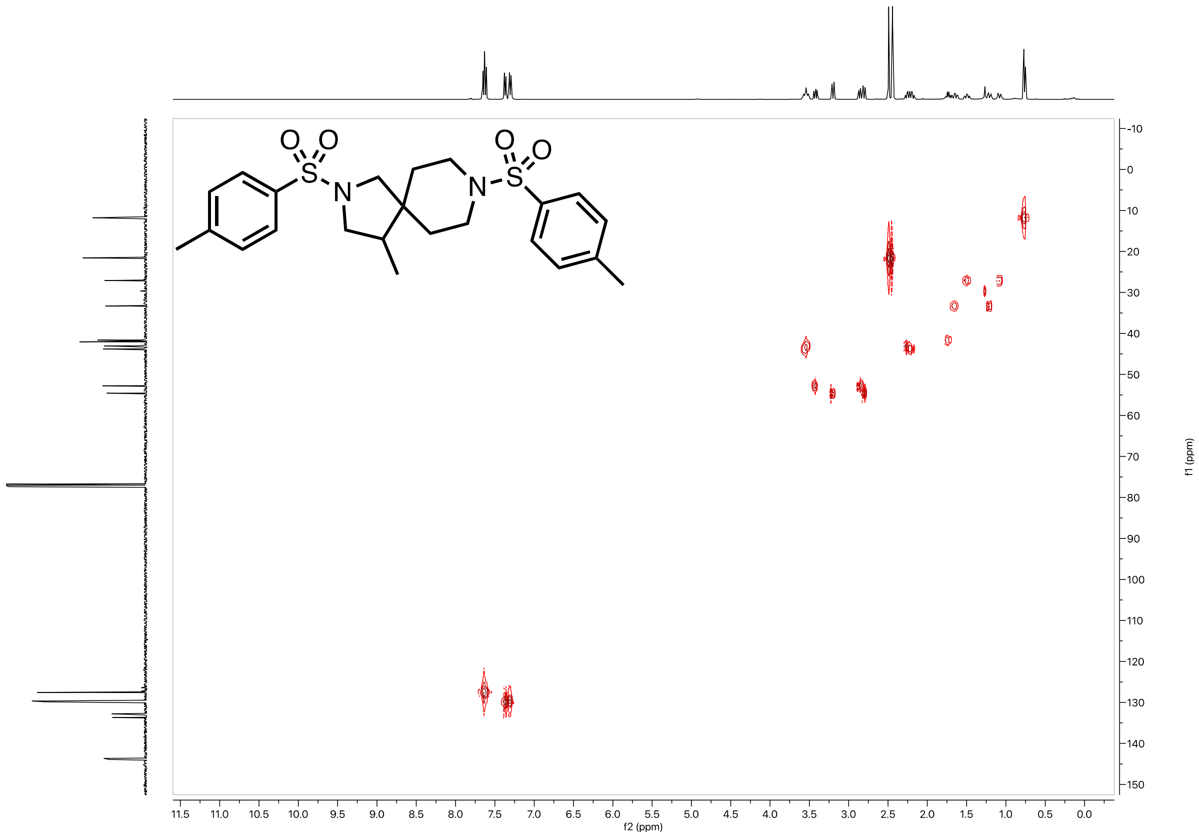

HSQC of *4-methyl-2,8-ditosyl-2,8-diazaspiro[4.5]decane* ($CDCl_3$).

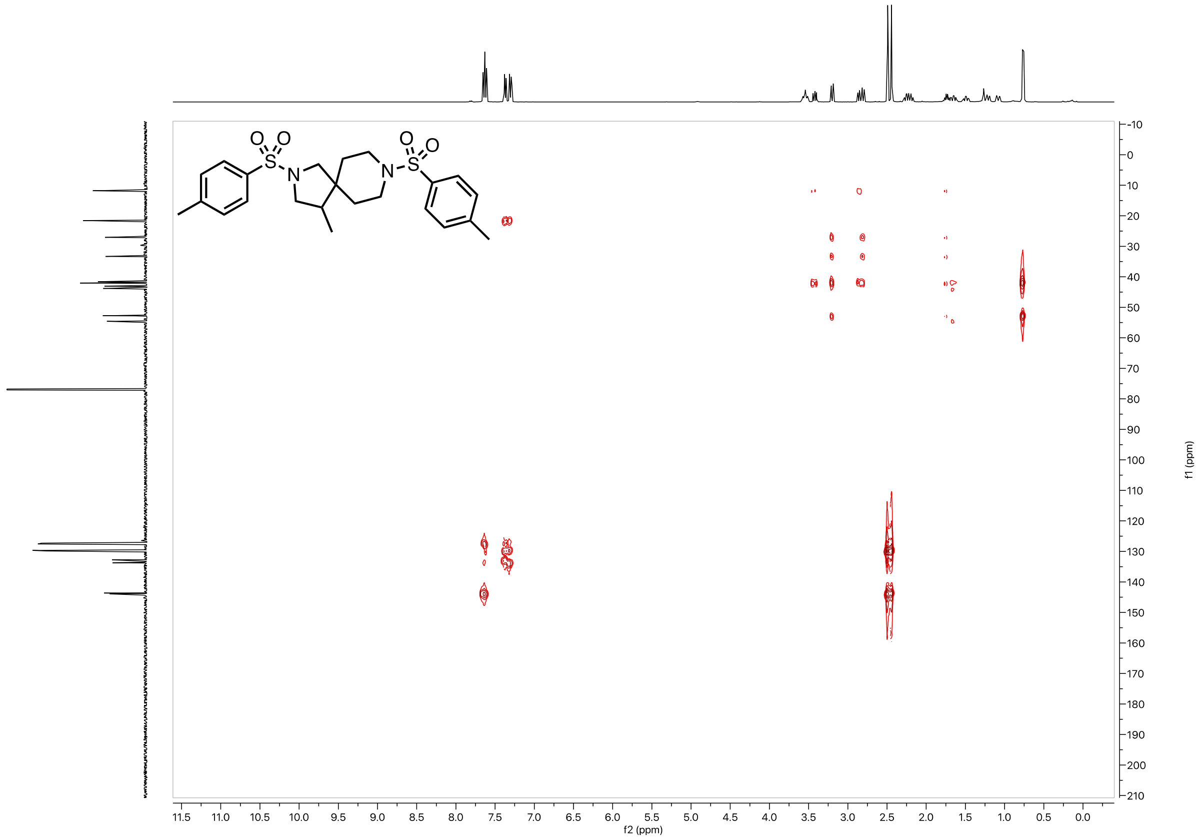

HMBC of *4-methyl-2,8-ditosyl-2,8-diazaspiro[4.5]decane* ($CDCl_3$).

8-methyl-6-((4-(methylsulfonyl)phenyl)sulfonyl)-6-azaspiro[3.4]octane (4c)

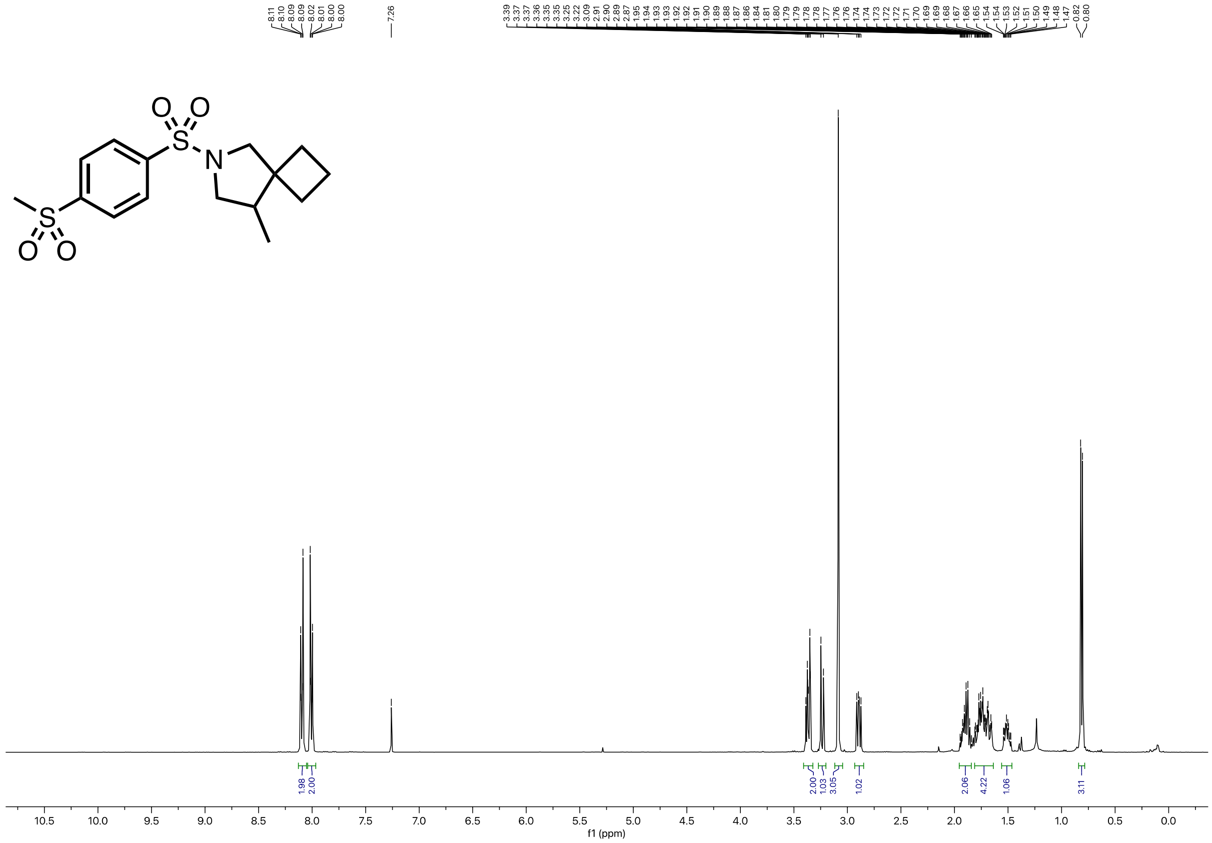

^{1}H NMR of *8-methyl-6-((4-(methylsulfonyl)phenyl)sulfonyl)-6-azaspiro[3.4]octane* (400 MHz, $CDCl_3$).

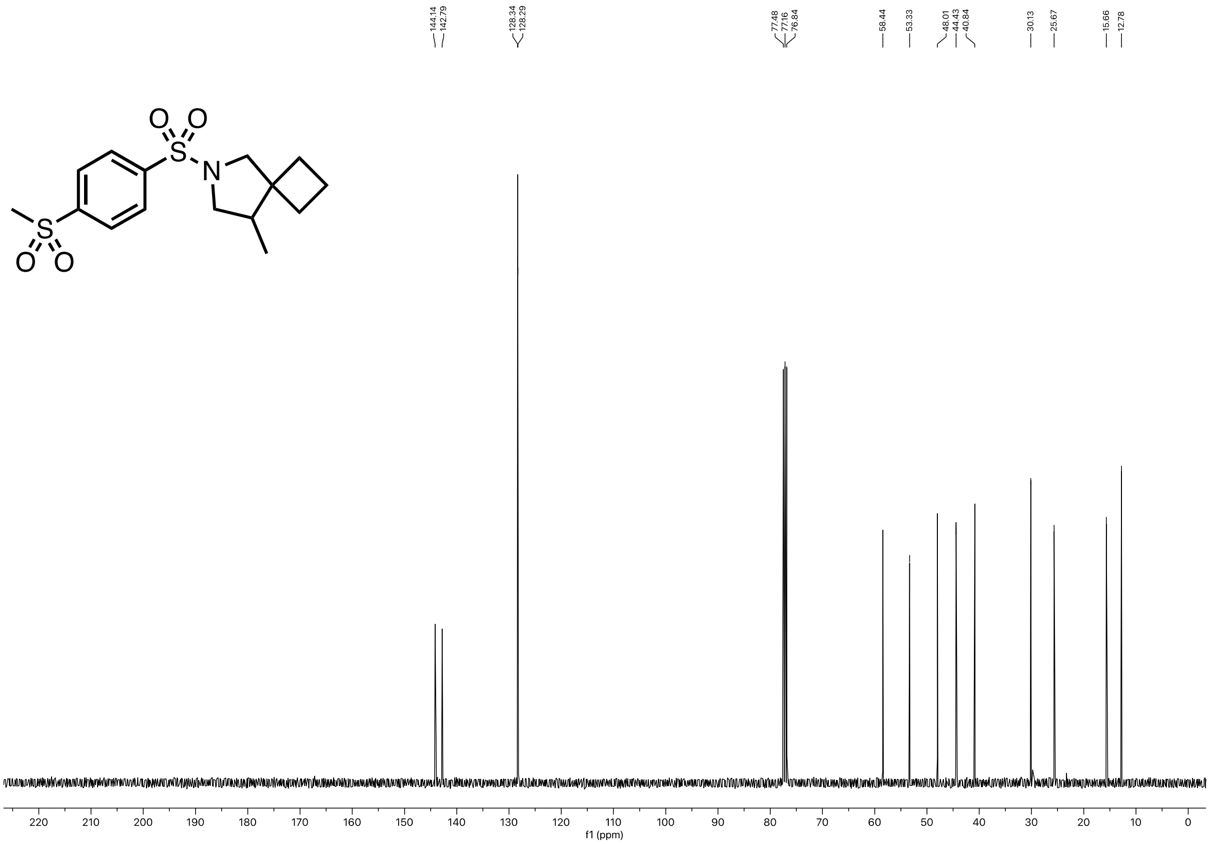

$^{13}C\{^{1}H\}$ NMR of *8-methyl-6-((4-(methylsulfonyl)phenyl)sulfonyl)-6-azaspiro[3.4]octane* (101 MHz, $CDCl_3$).

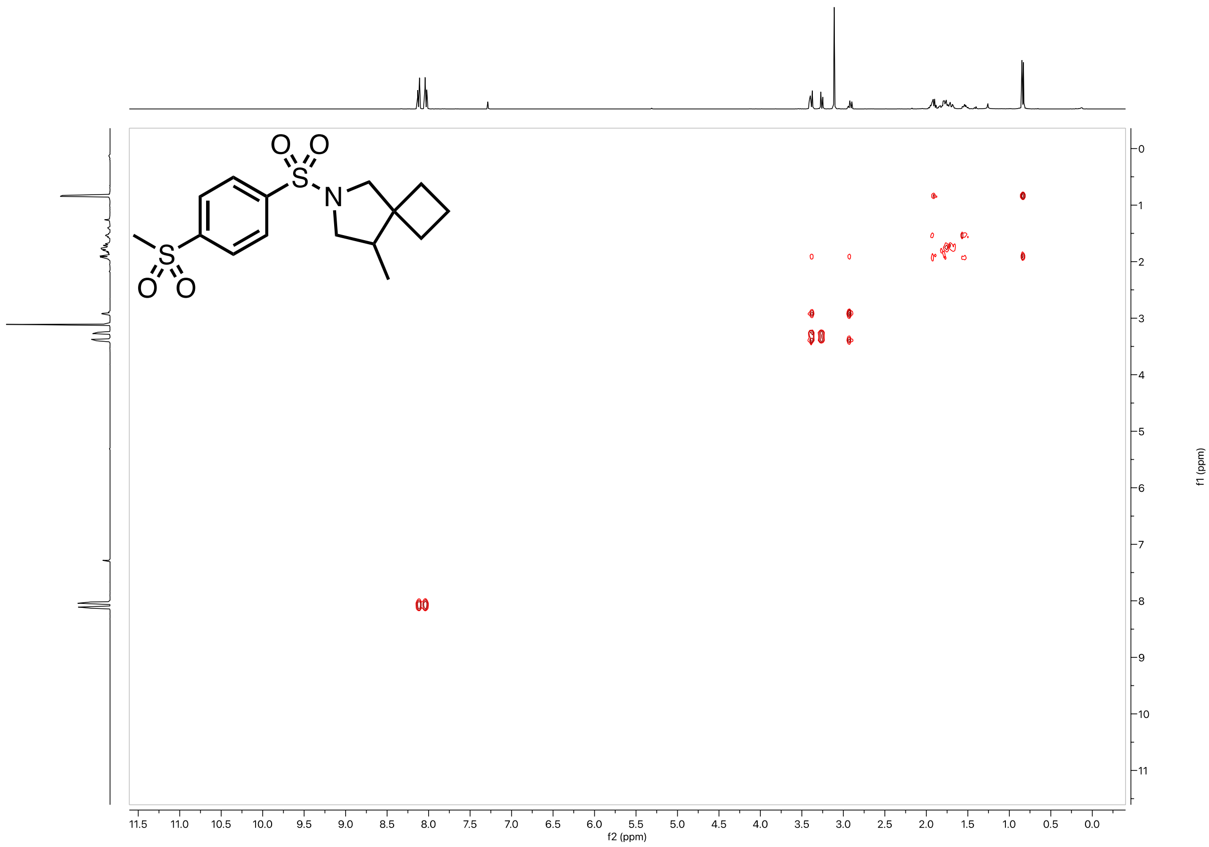

COSY of *8-methyl-6-((4-(methylsulfonyl)phenyl)sulfonyl)-6-azaspiro[3.4]octane* ($CDCl_3$).

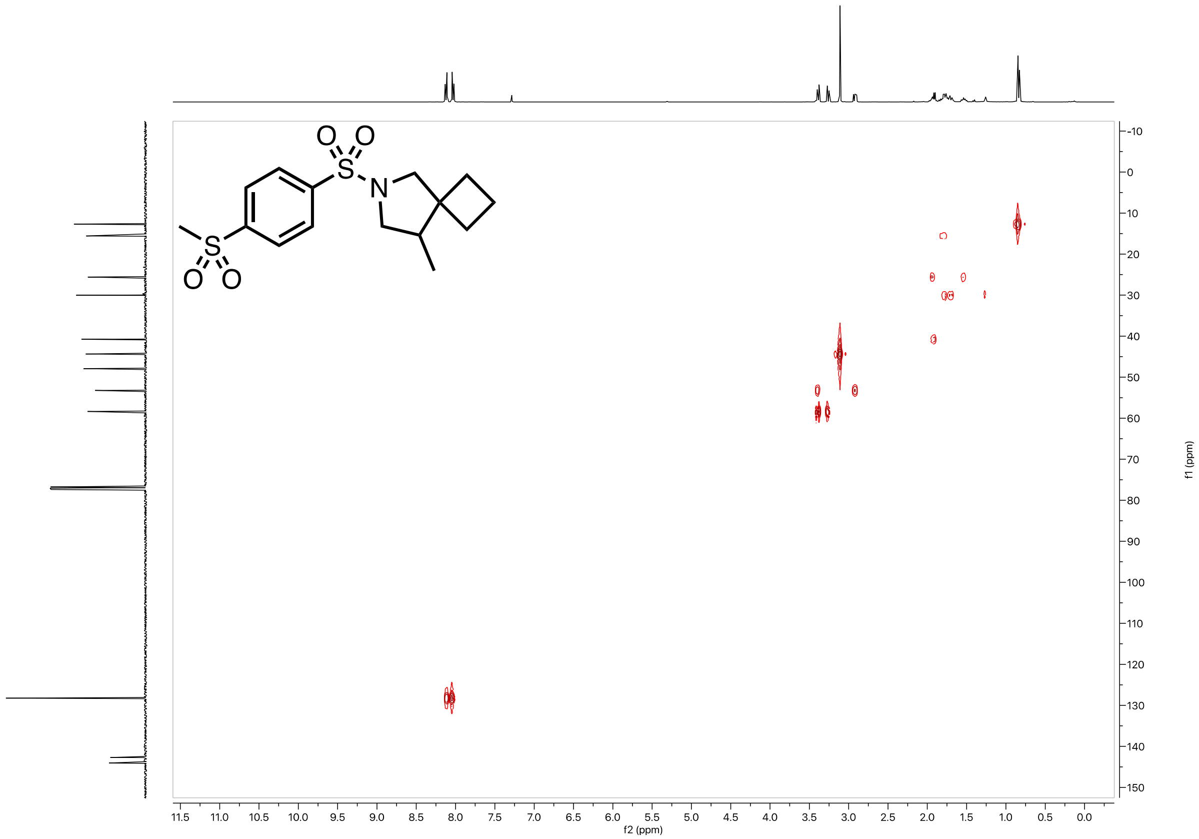

HSQC of *8-methyl-6-((4-(methylsulfonyl)phenyl)sulfonyl)-6-azaspiro[3.4]octane* ($CDCl_3$).

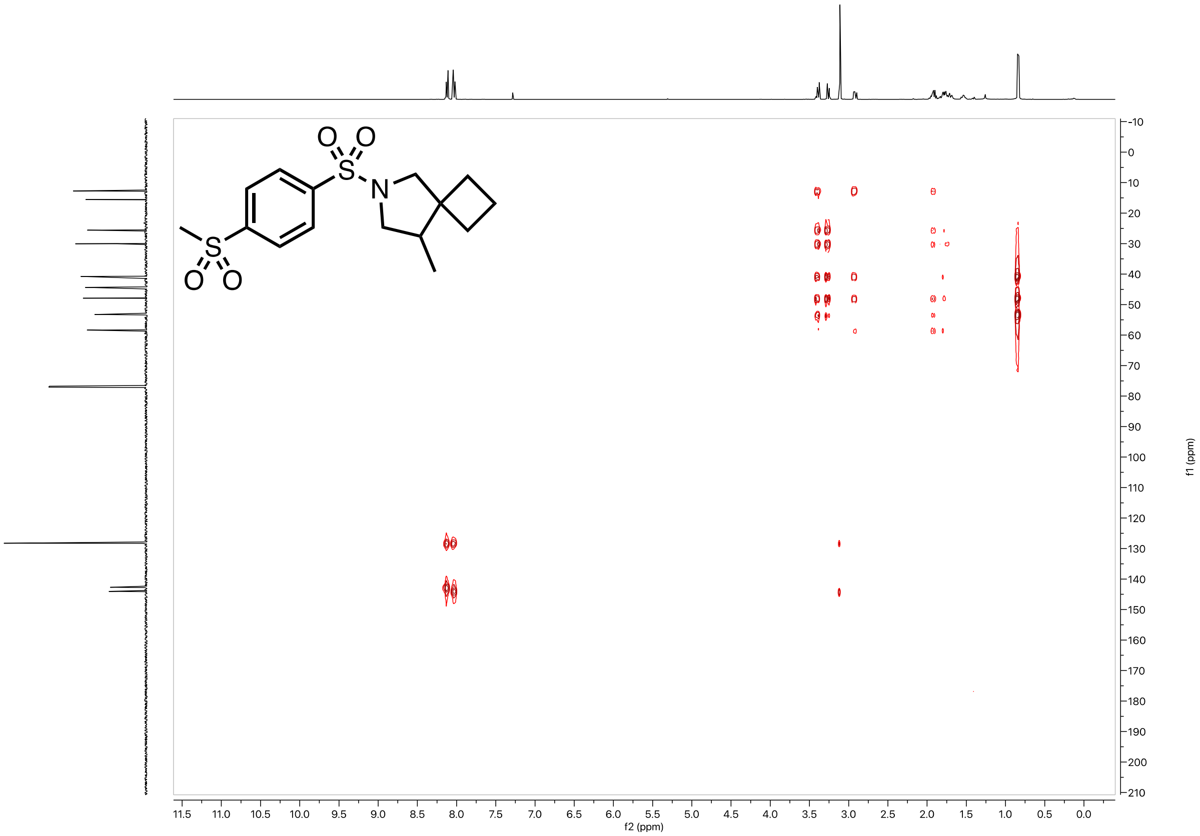

HMBC of *8-methyl-6-((4-(methylsulfonyl)phenyl)sulfonyl)-6-azaspiro[3.4]octane* ($CDCl_3$).

Methyl 4-((8-methyl-6-azaspiro[3.4]octan-6-yl)sulfonyl)benzoate (4d)

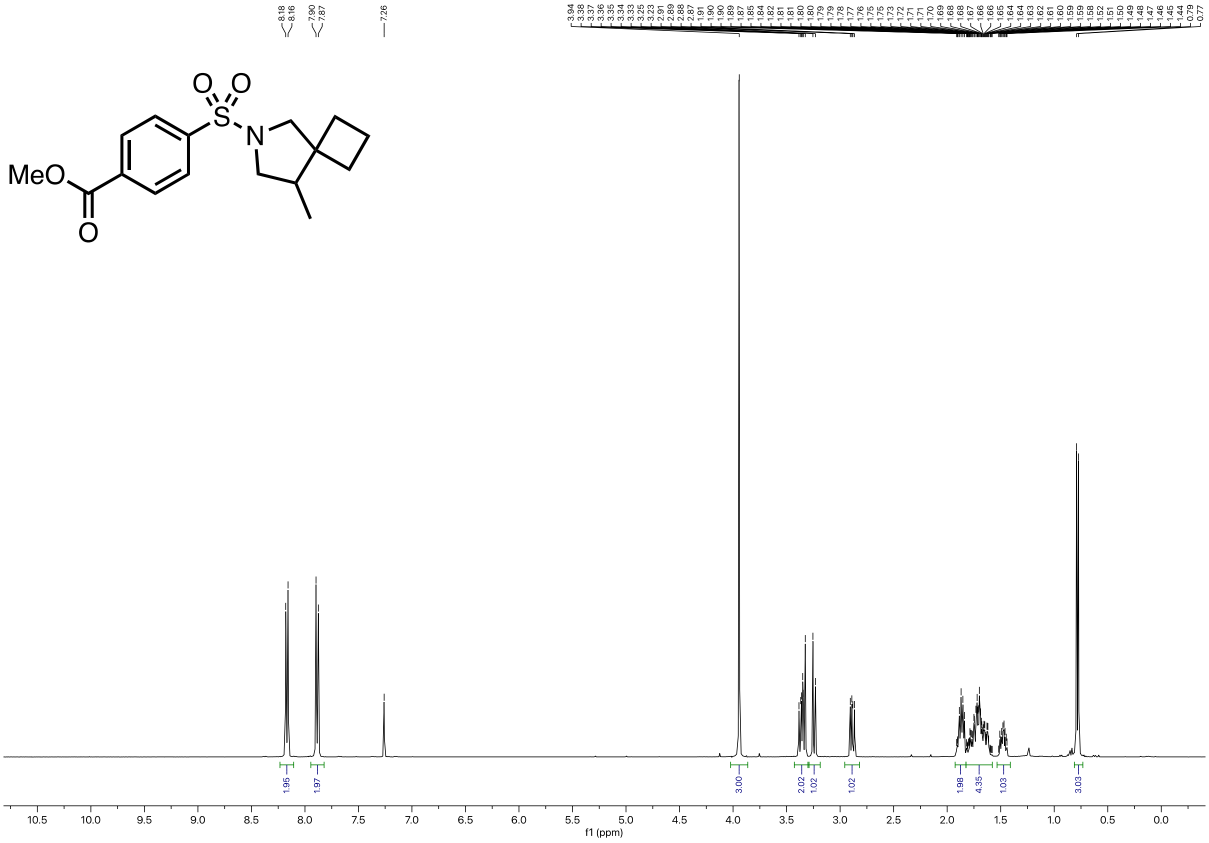

^{1}H NMR of *Methyl 4-((8-methyl-6-azaspiro[3.4]octan-6-yl)sulfonyl)benzoate* (400 MHz, $CDCl_3$).

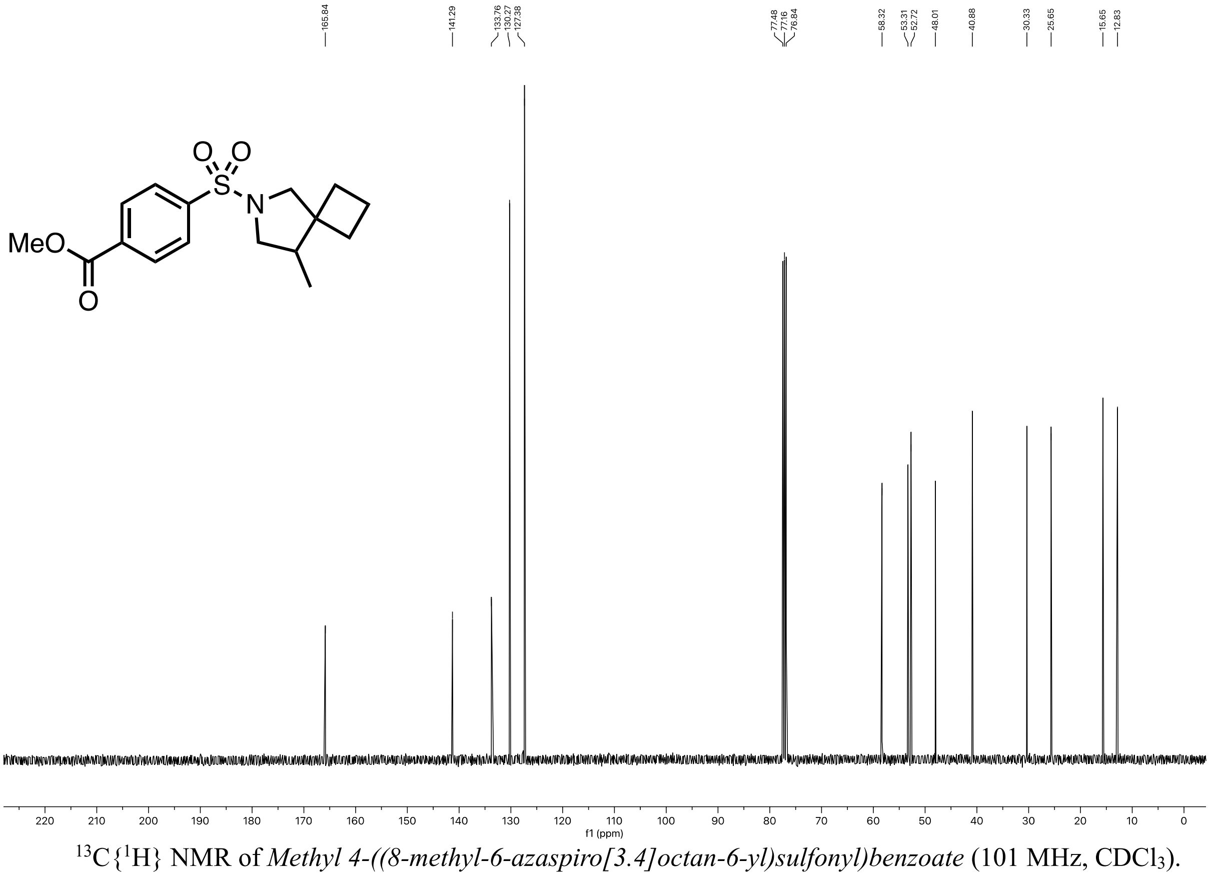

$^{13}C\{^1H\}$ NMR of *Methyl 4-((8-methyl-6-azaspiro[3.4]octan-6-yl)sulfonyl)benzoate* (101 MHz, $CDCl_3$).

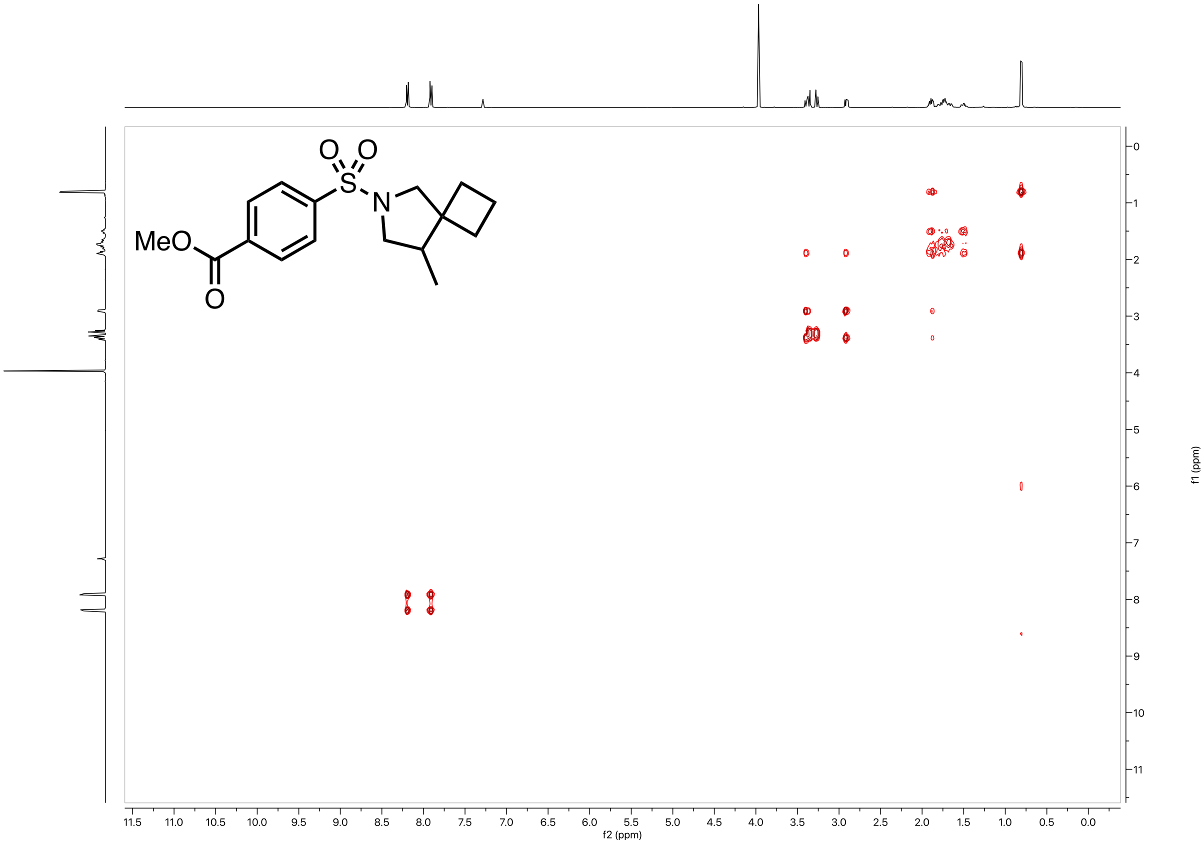

COSY of *Methyl 4-((8-methyl-6-azaspiro[3.4]octan-6-yl)sulfonyl)benzoate* ($CDCl_3$).

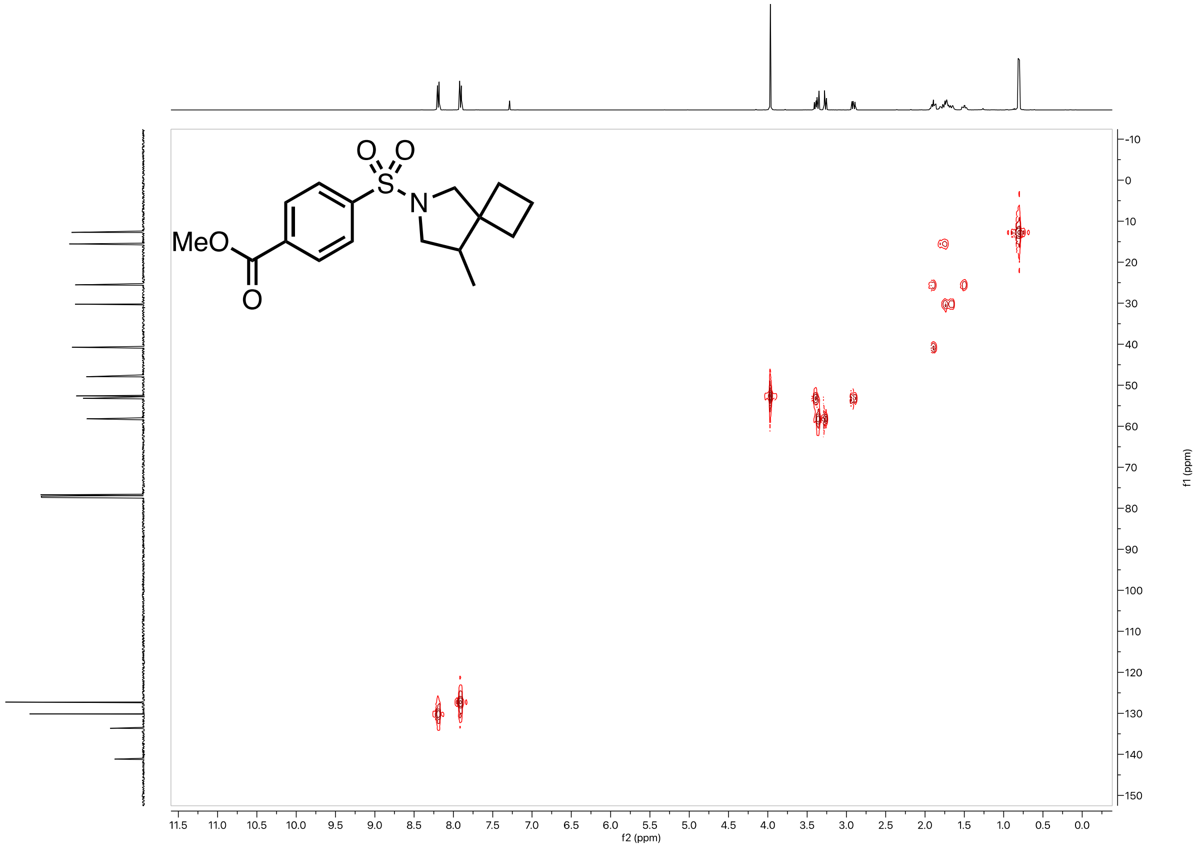

HSQC of *Methyl 4-((8-methyl-6-azaspiro[3.4]octan-6-yl)sulfonyl)benzoate* ($CDCl_3$).

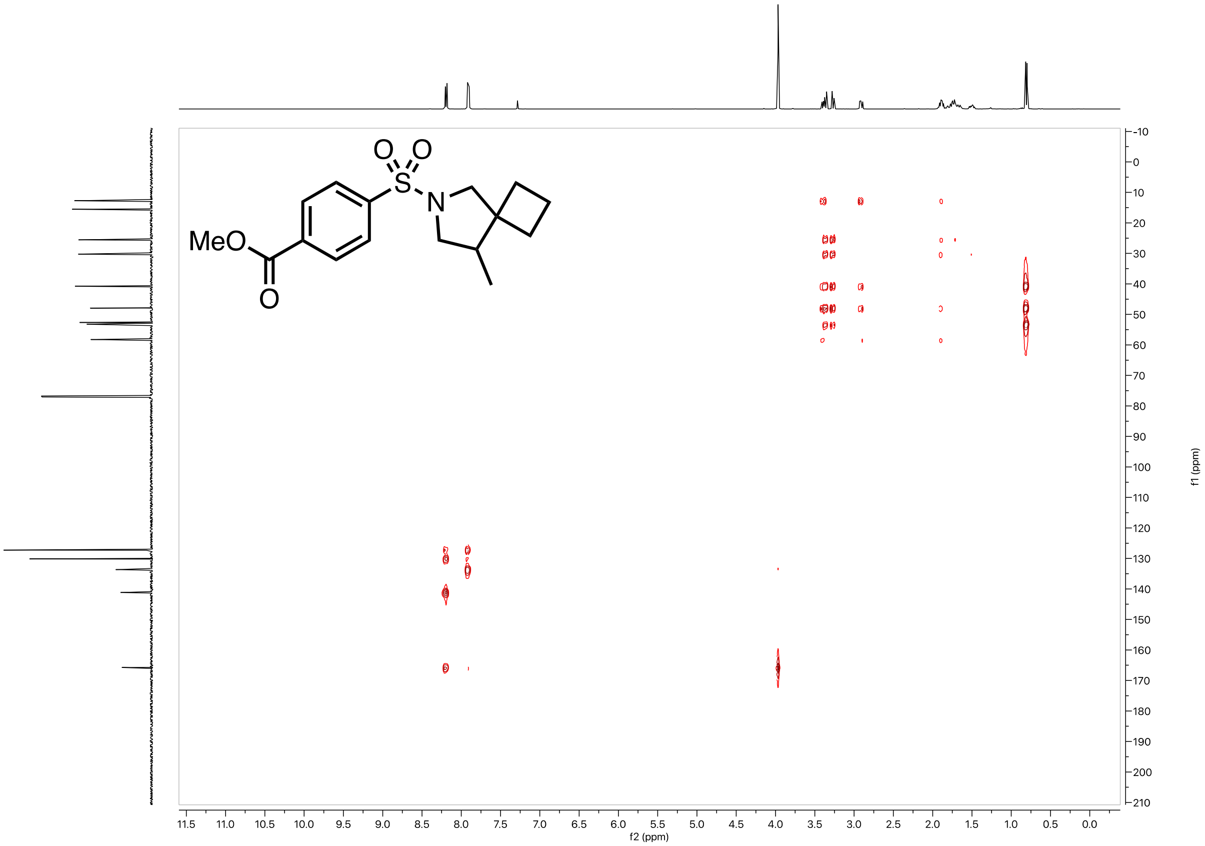

HMBC of *Methyl 4-((8-methyl-6-azaspiro[3.4]octan-6-yl)sulfonyl)benzoate* ($CDCl_3$).

2,8-ditosyl-2,8-diazaspiro[4.5]decane-4-carbaldehyde (5)

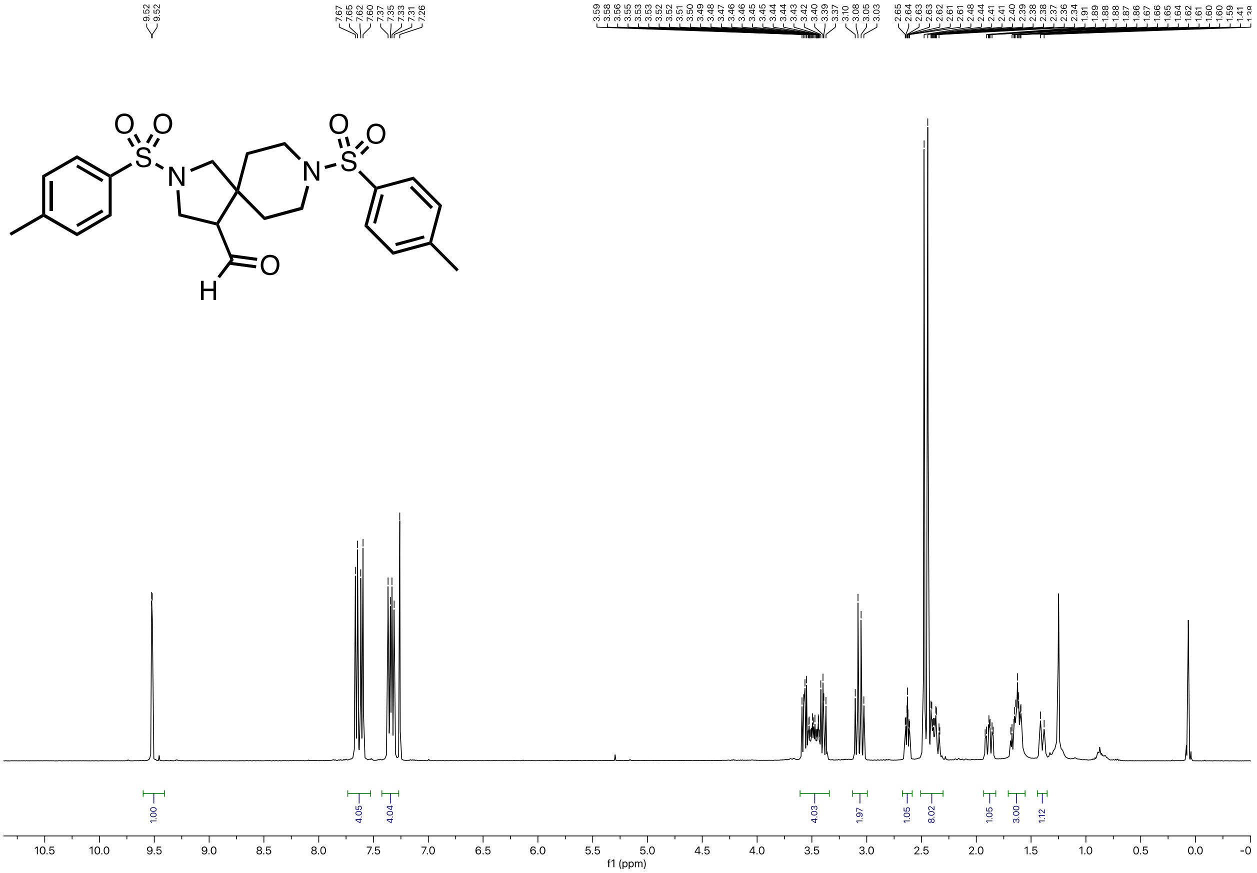

^{1}H NMR of *2,8-ditosyl-2,8-diazaspiro[4.5]decane-4-carbaldehyde* (400 MHz, $CDCl_3$).

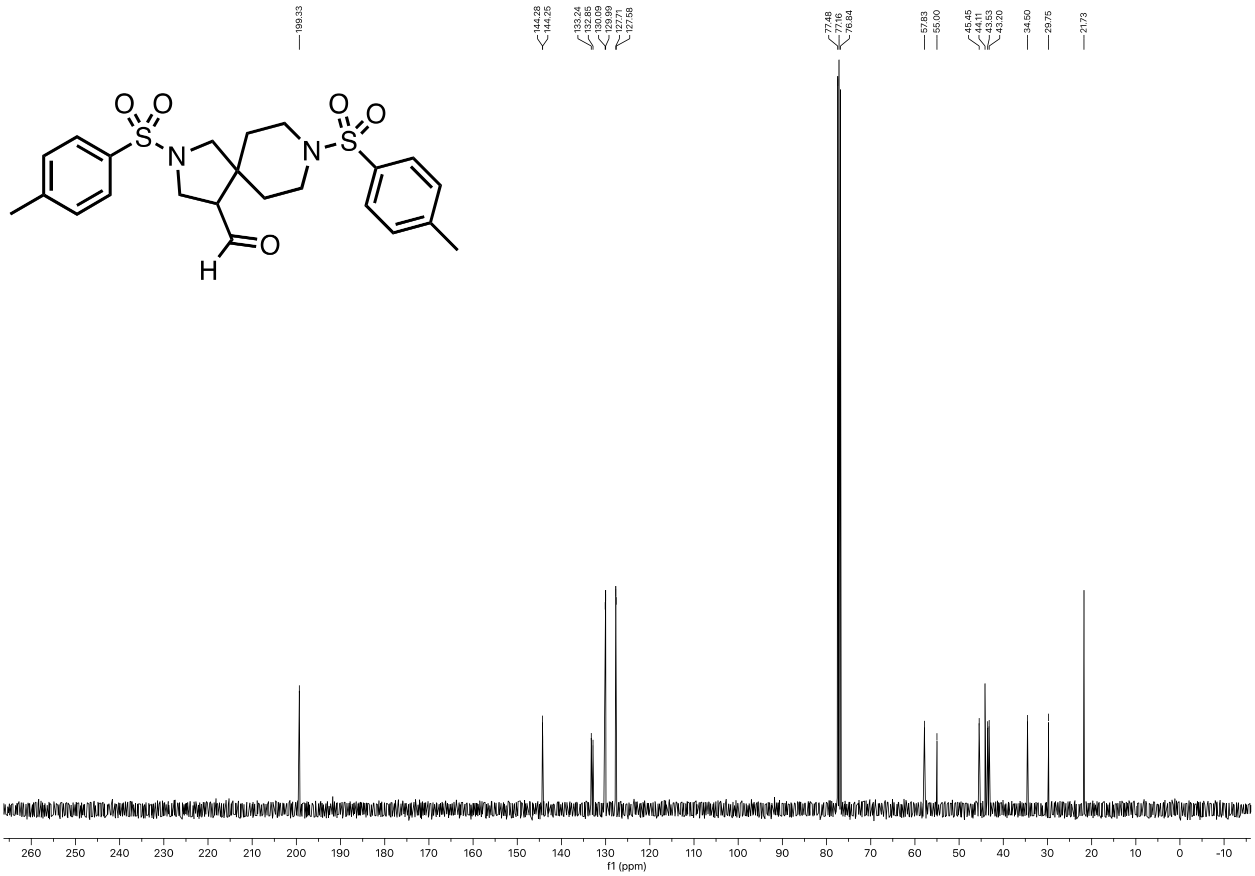

$^{13}C\{^{1}H\}$ NMR of *2,8-ditosyl-2,8-diazaspiro[4.5]decane-4-carbaldehyde* (101 MHz, $CDCl_3$).

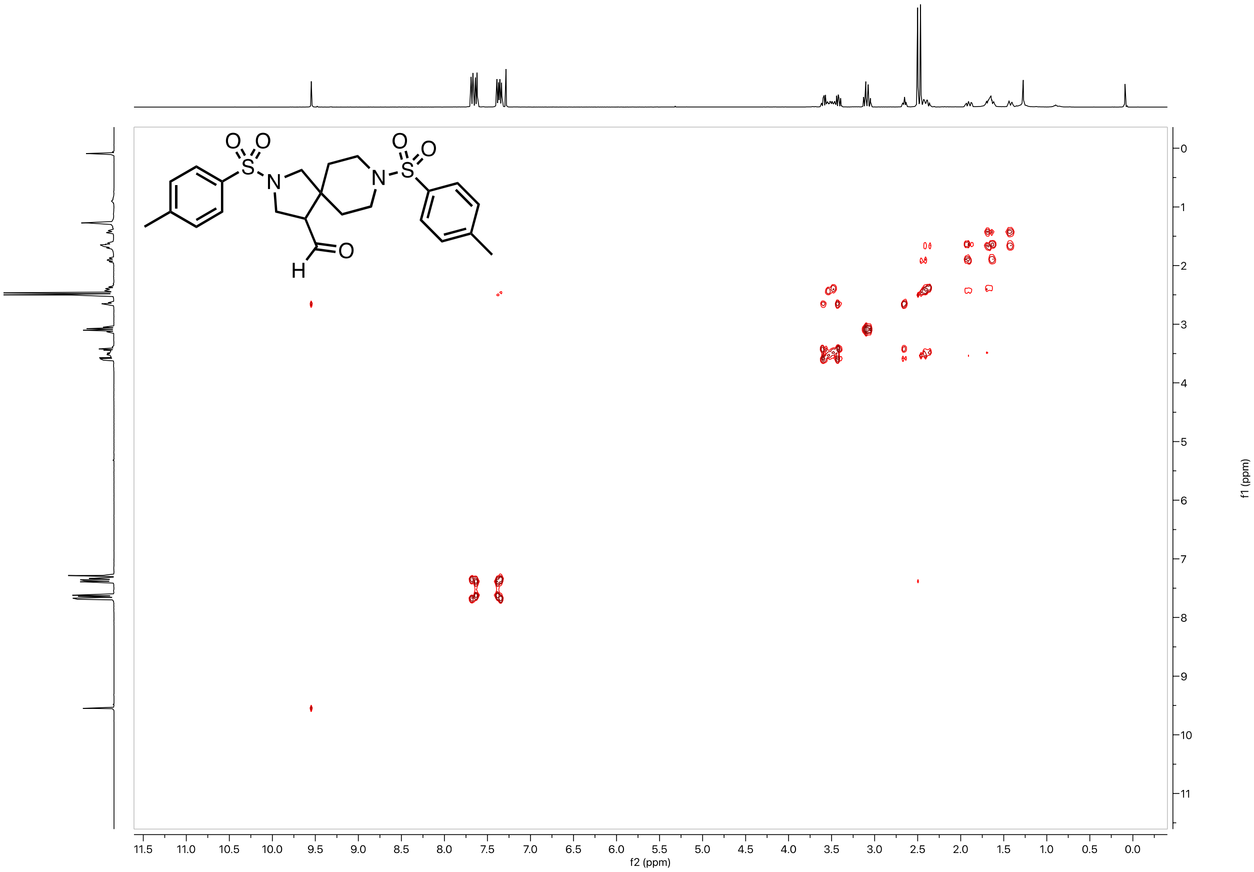

COSY of *2,8-ditosyl-2,8-diazaspiro[4.5]decane-4-carbaldehyde* ($CDCl_3$).

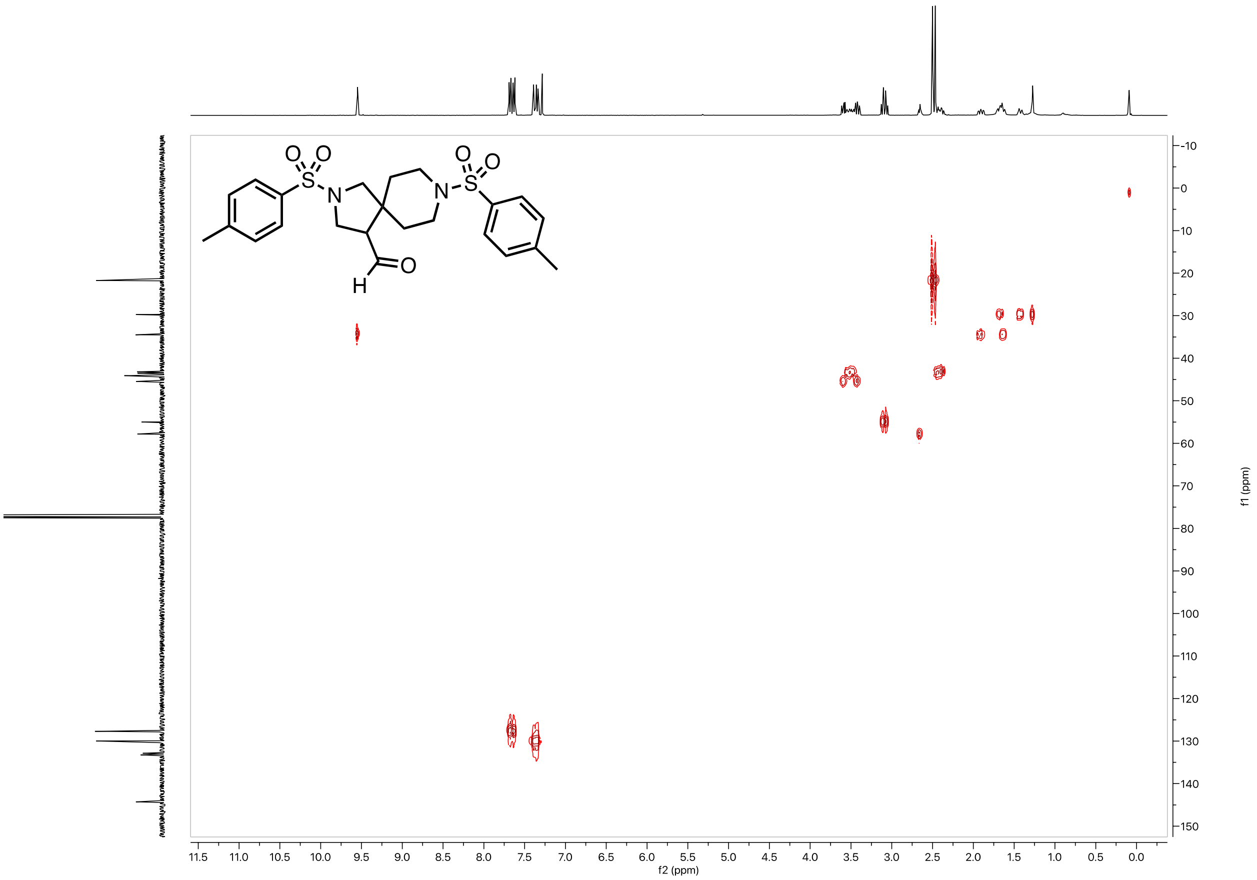

HSQC of *2,8-ditosyl-2,8-diazaspiro[4.5]decane-4-carbaldehyde* ($CDCl_3$).

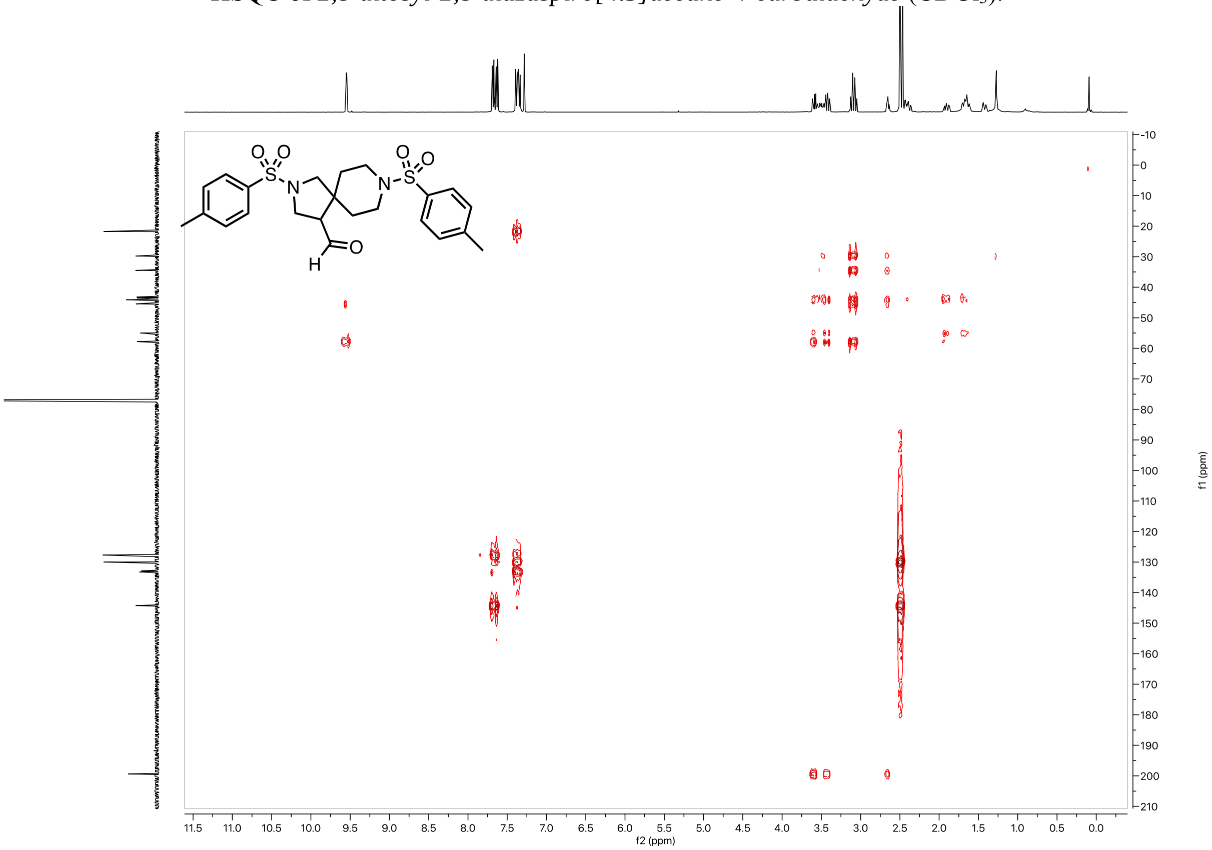

HMBC of *2,8-ditosyl-2,8-diazaspiro[4.5]decane-4-carbaldehyde* ($CDCl_3$).

4-(pyridin-3-ylmethyl)-2,8-ditosyl-2,8-diazaspiro[4.5]decane (6)

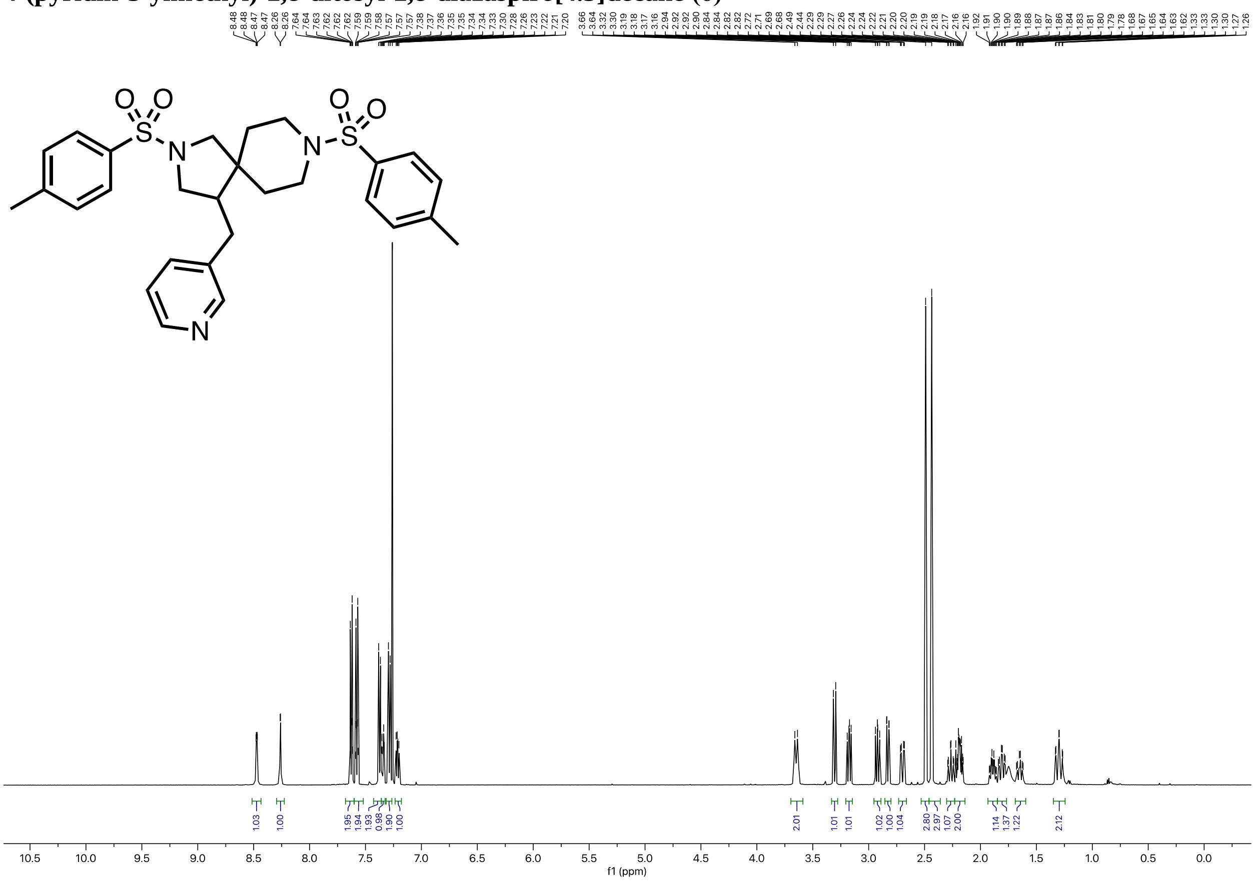

^{1}H NMR of *4-(pyridin-3-ylmethyl)-2,8-ditosyl-2,8-diazaspiro[4.5]decane* (500 MHz, $CDCl_3$).

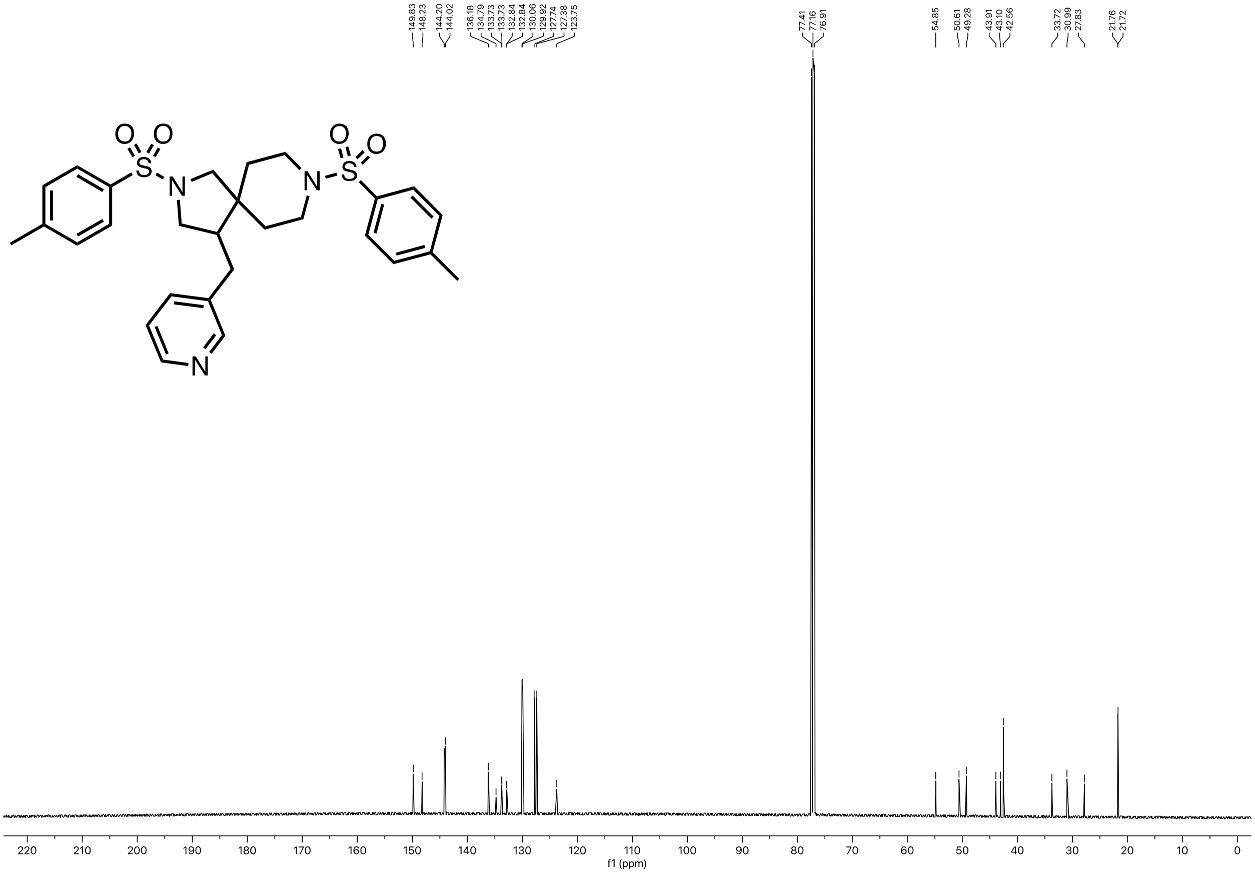

^{13}C{^{1}H} NMR of *4-(pyridin-3-ylmethyl)-2,8-ditosyl-2,8-diazaspiro[4.5]decane* (126 MHz, $CDCl_3$).

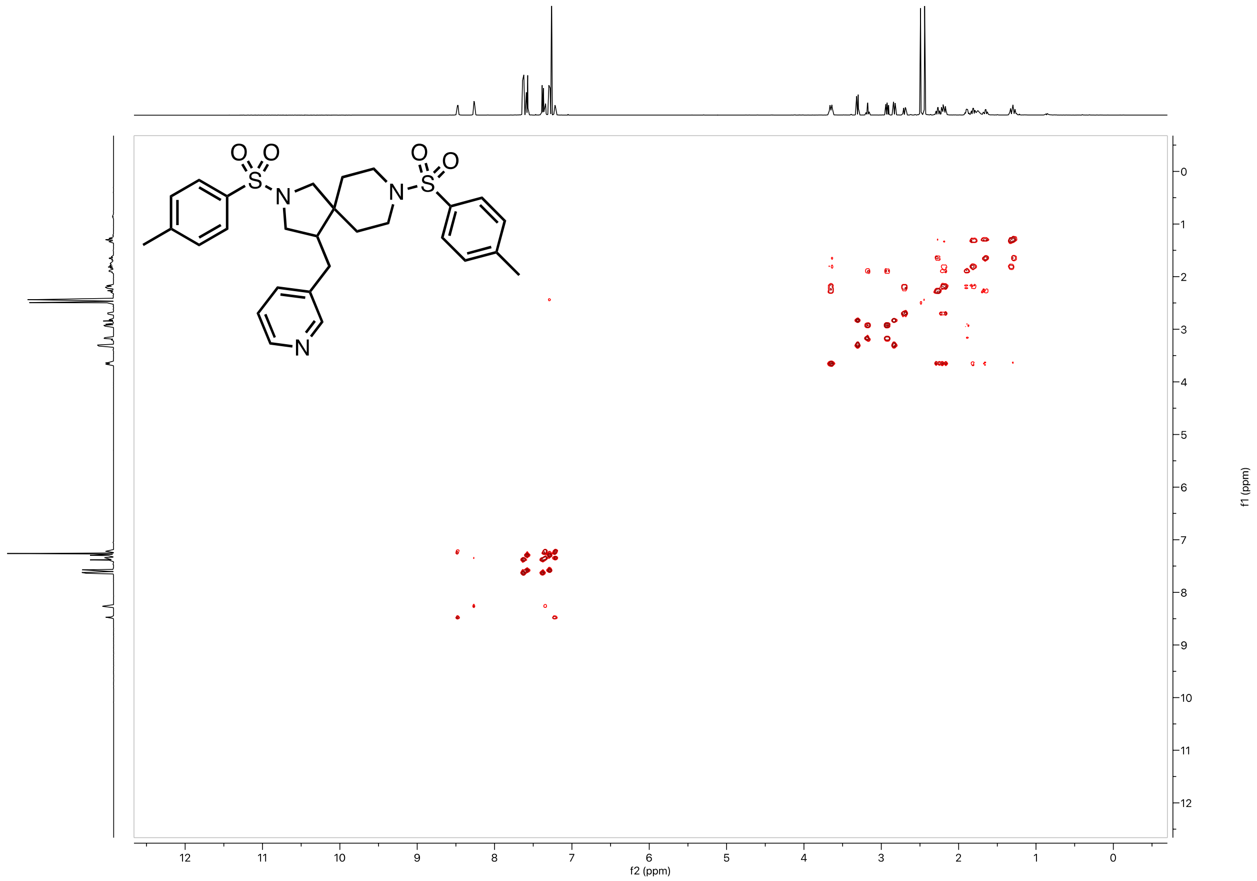

COSY of *4-(pyridin-3-ylmethyl)-2,8-ditosyl-2,8-diazaspiro[4.5]decane* ($CDCl_3$).

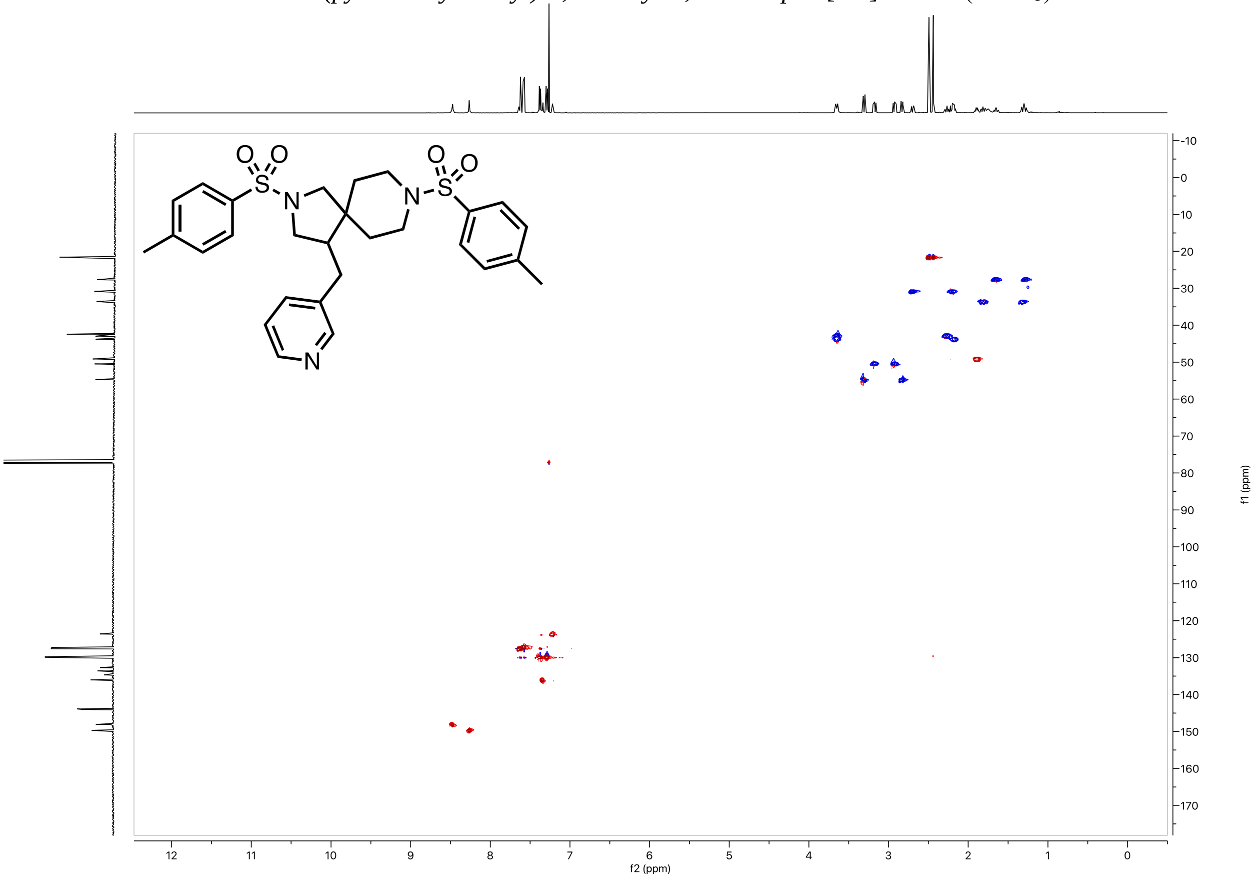

eHSQC of *4-(pyridin-3-ylmethyl)-2,8-ditosyl-2,8-diazaspiro[4.5]decane* ($CDCl_3$).

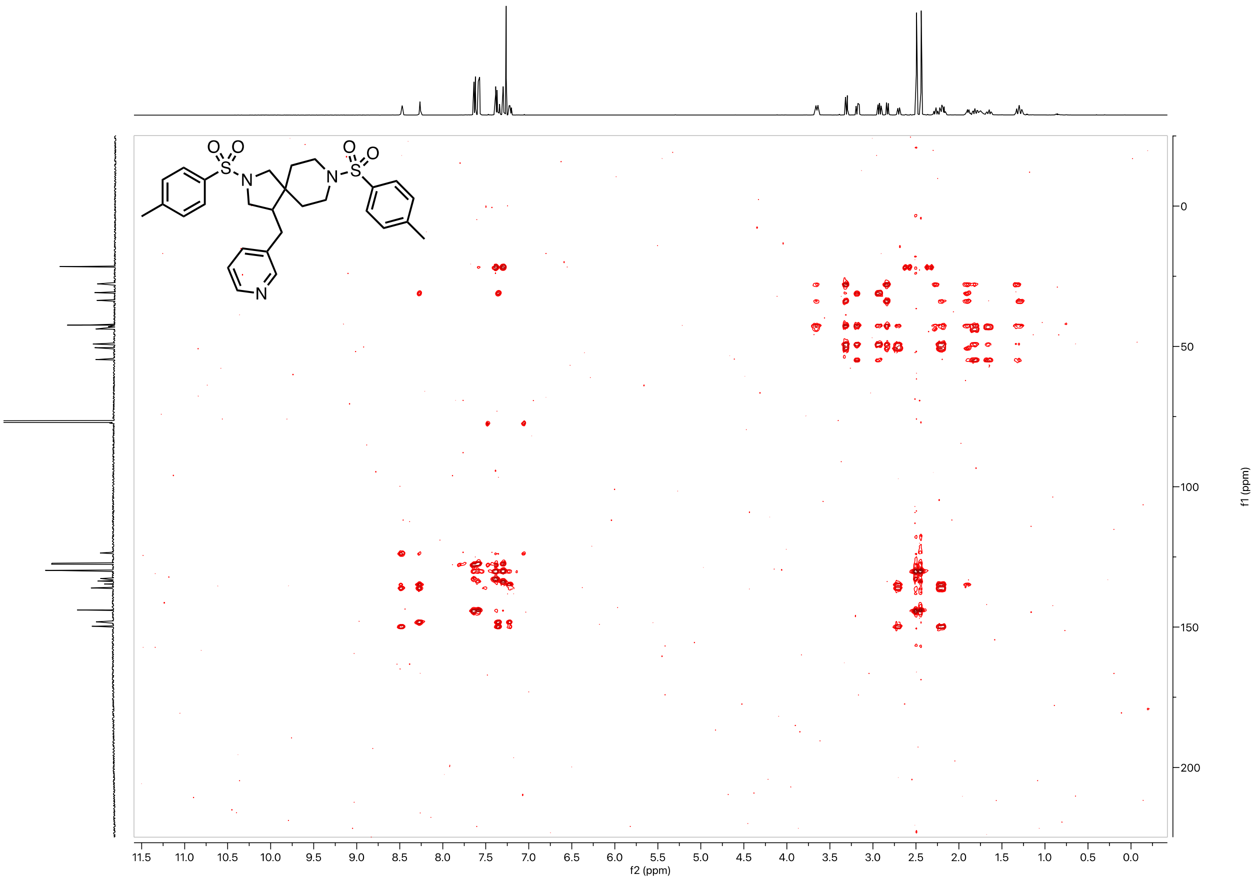

HMBC of *4-(pyridin-3-ylmethyl)-2,8-ditosyl-2,8-diazaspiro[4.5]decane* ($CDCl_3$).

4. Thermal ellipsoid plot/ORTEP diagram for 3aa

Displacement ellipsoids are shown at 50% probability for compound **3aa**.

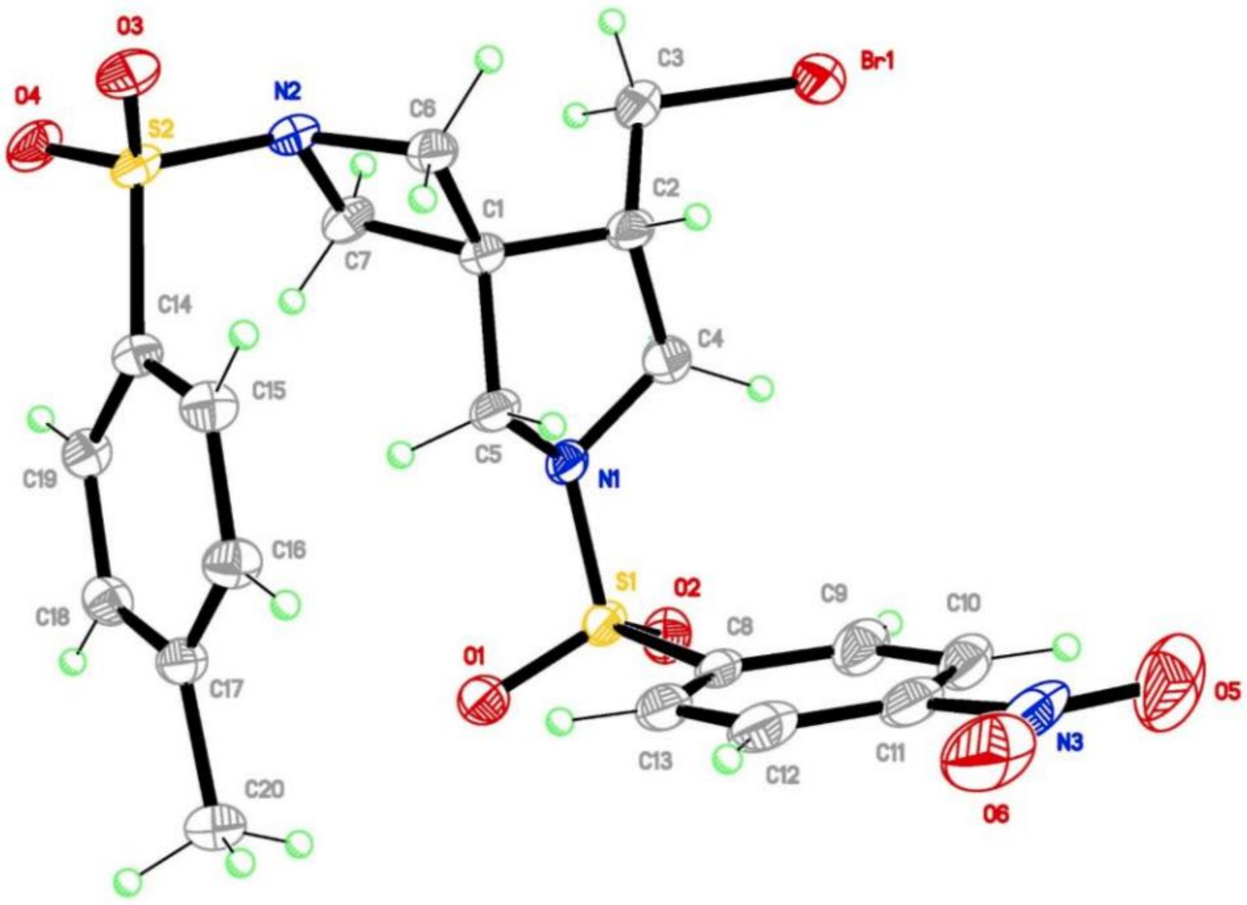

5. X-ray Crystallographic information

Single crystals of compound **3aa** were obtained by slow vapour diffusion of petroleum ether into a solution of **3aa** in DCM. Data collection at 180(2) K using Cu Kα radiation (λ = 1.54178 Å) on a Bruker D8-QUEST PHOTON-III diffractometer. The data acquisition was done with the APEX4 software. SAINT software was implemented for data integration and reduction. Reflections were merged by SHELXL software according to the crystal class for the calculation of statistics and refinement. Absorption corrections were employed to the data using the program SADABS. Structures were solved by direct methods using SHELXT and refined using SHELXL. The structural illustrations were obtained using Mercury. The detailed data collection and structure refinement are summarized in Table S1. CCDC-2190648 (for **3aa**), contained supplementary crystallographic data for this paper.

Table S1: Crystal data and structure refinement for **3aa.**

	CCDC 2190648
Formula	$C_{20}H_{22}BrN_3O_6S_2$
Formula weight	544.44

T/K	180(2)
Crystal system	triclinic
Space group	P 1
a/Å	5.9396(4)
b/Å	12.9121(7)
c/Å	15.0328(9)
α/Å	92.626(3)
β/Å	101.032(4)
γ/Å	98.815(4)
V/Å^3	1114.87(12)
Z	2
ρ_{calc} g/cm^3	1.622
μ/mm^{-1}	4.645
F(000)	556
Crystal size/mm^3	0.060
Radiation	CuKα (λ = 1.54178)
2θ range for data collection/°	3.004 to 66.687
Index ranges	-7 ≥ h ≤ 7, -15 ≥ k ≤ 15, -17 ≥ l ≤ 17
Reflections collected	3898
Data/restraints/parameters	3898/158/399
GOF on F^2	1.056
Final *R* indices [$I>2\sigma(I)$]	R_1 = 0.0423, wR_2 = 0.0901
R indices [all data]	R_1 = 0.0517, wR_2 = 0.0946
